# Altered expression of mitochondrial and extracellular matrix genes in the heart of human fetuses with chromosome 21 trisomy

**DOI:** 10.1186/1471-2164-8-268

**Published:** 2007-08-07

**Authors:** Anna Conti, Floriana Fabbrini, Paola D'Agostino, Rosa Negri, Dario Greco, Rita Genesio, Maria D'Armiento, Carlo Olla, Dario Paladini, Mariastella Zannini, Lucio Nitsch

**Affiliations:** 1 Dipartimento di Biologia e Patologia Cellulare e Molecolare, University Federico II, Napoli, Italy; 2 BIOGEM, Biotechnology and Molecular Genetics, Italy; 3 Institute of Biotechnology, University of Helsinki, Finland; 4 Dipartimento di Scienze Biomorfologiche e Funzionali, University Federico II, Napoli, Italy; 5 Dipartimento di Scienze Ostetriche, Ginecologiche ed Urologiche e Fisiopatologia della Riproduzione, University Federico II, Napoli, Italy; 6 Istituto di Endocrinologia ed Oncologia Sperimentale (IEOS) del CNR, Napoli, Italy

## Abstract

**Background:**

The Down syndrome phenotype has been attributed to overexpression of chromosome 21 (Hsa21) genes. However, the expression profile of Hsa21 genes in trisomic human subjects as well as their effects on genes located on different chromosomes are largely unknown. Using oligonucleotide microarrays we compared the gene expression profiles of hearts of human fetuses with and without Hsa21 trisomy.

**Results:**

Approximately half of the 15,000 genes examined (87 of the 168 genes on Hsa21) were expressed in the heart at 18–22 weeks of gestation. Hsa21 gene expression was globally upregulated 1.5 fold in trisomic samples. However, not all genes were equally dysregulated and 25 genes were not upregulated at all. Genes located on other chromosomes were also significantly dysregulated. Functional class scoring and gene set enrichment analyses of 473 genes, differentially expressed between trisomic and non-trisomic hearts, revealed downregulation of genes encoding mitochondrial enzymes and upregulation of genes encoding extracellular matrix proteins. There were no significant differences between trisomic fetuses with and without heart defects.

**Conclusion:**

We conclude that dosage-dependent upregulation of Hsa21 genes causes dysregulation of the genes responsible for mitochondrial function and for the extracellular matrix organization in the fetal heart of trisomic subjects. These alterations might be harbingers of the heart defects associated with Hsa21 trisomy, which could be based on elusive mechanisms involving genetic variability, environmental factors and/or stochastic events.

## Background

Down syndrome (DS) is the most frequent autosomal aneuploidy that is compatible with post-natal life. It results from complete or partial trisomy of chromosome 21 (Hsa21) and is characterized by a complex phenotype in which over 80 features occur with various degrees of expression and frequency [1]. Down syndrome is a major cause of congenital heart defects (CHD). It is associated mostly with endocardial cushion defects [2,3], the most frequent being atrioventricular canal defects (AVCD) followed by ventricular septal defects (VSD) and tetralogy of Fallot [3].

Attempts to identify the Hsa21 genes that contribute to the DS phenotype have focused on the Down Syndrome Critical Region (DSCR) which spans approximately 5.4 Mb in band 21q22.3 [4-7]. The DSCR hypothesis predicts that a gene, or genes, in this region are sufficient to produce the specific DS features when present in three copies. A narrowed region was also proposed as the candidate region for DS-CHD [8]. The DSCR hypothesis was tested in mice [9,10] and it was found that trisomy for DSCR alone is necessary but not sufficient for brain phenotypes in trisomic mice. These results suggest that the origins of trisomic phenotypes are even more complicated than formerly assumed and that they probably involve multiple gene interactions [10]. It has been proposed that the complex phenotypic alterations of DS could result from an interplay between Hsa21 genes and developmentally regulated genes elsewhere on the genome [11] and that the loss of genetic balance in pivotal processes regulating development might increase susceptibility to genetic and environmental insults [12].

The mechanism by which an extra copy of chromosome 21 produces the DS phenotype is unknown and is possibly complex. It has been postulated that a triplicated chromosome 21 causes a 50% increase in the expression of trisomic genes as a primary dosage effect [13]. With the advent of microarrays and other high-throughput technologies, it became possible to demonstrate this primary dosage effect. By measuring the steady-state-RNA levels in human DS tissues and cells [14-17] and in tissues from mouse models of DS [18-22] it has been established that the trisomy causes an overall 50% increase in the RNA levels of Hsa21 genes. At least some of this RNA increase might result in perturbations of the pathways and cellular processes in which these genes function [23]. This can affect cardiac development and result in CHD [16].

Given the number of candidate genes involved, the number of alternative splice variants of individual genes and the number of pathways in which these genes function, pathway analysis seems the most suitable approach to the study of genotype/phenotype correlations in DS [23]. Data from functional studies suggest that multiple chromosome 21 genes affect protein processing, mitochondrial function and reactive oxygen species production, one-carbon metabolism and cell adhesion [24]. Mitochondrial function and reactive oxygen pathways are already targets in the study of neurodegeneration [25-28].

In this study we determined the transcription profile of Hsa21 genes in the heart of human fetuses at 18–22 weeks of gestation. Our goal was to understand how upregulation of Hsa21 genes may influence the expression of genes mapping on other chromosomes and potentially involved in CHD. We investigated the differential gene expression and pathway dysregulation associated with Hsa21 trisomy in fetal hearts using DNA microarray technology and functional analysis. By comparing heart samples from fetuses with or without Hsa21 trisomy, we observed the upregulation of most, but not all, Hsa21 genes as well as the differential expression of genes located on different chromosomes. We found that downregulation of several mitochondrial genes and upregulation of many extracellular matrix genes was a common feature of all trisomic hearts.

## Results

### General

Cardiac tissue was obtained from fetuses at 18–22 weeks of gestation after therapeutic abortion. Ten samples from fetuses trisomic for Hsa21 and 5 from euploid, control fetuses were studied. At autopsy, the phenotype of the fetuses with Hsa21 trisomy was found to be consistent with DS. The presence of CHD was established by direct examination at the time of tissue explantation and was confirmed by histological analysis of a portion of the heart (see Materials and Methods). The demographic data of the analyzed samples are shown in Table 1.

**Table 1:** Characteristics of the samples analyzed in the study

**Telethon Bank ID**	**Experiment ID**	**Karyotype**	**Age (gw)**	**PMI (h)**	**Heart defects**	**GEO accession**
**NH GROUP**						

**TB 26**	H1	46,XY	20	3	NO	GSM30867
**TB 21**	H2	46,XY	21	3	NO	GSM30868
**TB 30**	H3	46,XY	20	1	NO	GSM30869
**TB 32**	H4	46,XX	21	1	NO	GSM30870
**TB 23**	H5	46,XX	21	4	NO	GSM30871
						
**DSH GROUP**						

**TB 37**	DH1	47,XY,+21	21	2	NO	GSM30862
**TB 43**	DH3	47,XY,+21	20	3	NO	GSM30863
**TB 50**	DH4	47,XY,+21	20	3	NO	GSM30864
**TB 47**	DH5	47,XY,+21	19	4	NO	GSM30865
**TB 48**	DH6	47,XY,+21	21	3	NO	GSM30866
**TB 9**	CDH1	47,XY,+21	22	2	YES	GSM30723
**TB 33**	CDH2	47,XY,+21	20	4	YES	GSM30855
**TB 55**	CDH4	47,XX,+21	21	2	YES	GSM30864
**TB 22**	CDH5	47,XX,+21	18	3	YES	GSM30859
**TB 57**	CDH6	47,XX,+21	20	3	YES	GSM30860

Experiment accession is GSE1789

DSH = Heart samples from fetuses with trisomy of Hsa21

NH = Heart samples from control fetuses

ID = identifier numbers

gw = gestational weeks

PMI(h) = Post-mortem interval (hours)

Heart defects: TB9, Fallot's trilogy; TB33, Ventricular Septal Defect; TB55 Atrio-Ventricular Canal Defect; TB22 and TB57, Fallot's Tetralogy.

GEO = Gene Expression Omnibus

### Analysis of the fetal heart transcriptome

The gene expression profile of the 15 fetal hearts was determined by DNA microarray analysis using Affymetrix HG-U133A oligonucleotide arrays. The 22,283 probe sets represented on the Affymetrix chip corresponded to ~14,500 genes and 500 expressed sequence tags and clones. Affymetrix Microarray Suite (MAS 5.0) software was used to identify presence calls and to quantify gene expression. We first determined the total number of genes expressed in the fetal hearts by assessing the number of presence calls. Approximately 7,200 probe sets, corresponding to ~5,100 individual genes, were called present in all the analyzed samples. We also calculated how many genes were called present in at least 2/3 of the samples, and found that ~10,400 probe sets, corresponding to ~7,500 individual genes, were called present in at least 10 samples. This might represent a more reliable estimate of expressed genes since the MAS 5.0 software underestimates presence calls [29]. Expressed genes and their presence calls are reported (see Additional file 1).

### Expression of Hsa21 genes

**Figure 1:**
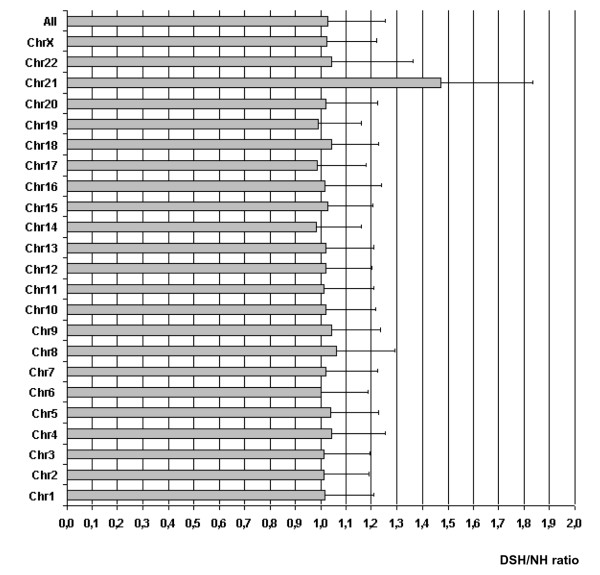
**DSH/NH ratio of gene expression calculated for each chromosome and for all the chromosomes**. Cumulative gene expression (raw data) was calculated for each chromosome and for all chromosomes and expressed as ratio between DSH and NH samples. Differences among chromosomes were evaluated using the ANOVA one-way test with Bonferroni post-hoc multiple comparison test. Only the difference between Hsa21 and any other chromosome is significant (p < 0.0001). Other comparisons among chromosomes are not statistically significant. The mean DSH/NHs ratio is ~1 for all the chromosomes and 1.48 ± 0.35 for Hsa21. DSH = Heart samples from fetuses with Hsa21 trisomy (includes the samples: CDH1, CDH2, CDH4, CDH5, CDH6 from fetuses with heart defects, and the samples: DH1, DH3, DH4, DH5, DH6 from fetuses without heart defects). NH = Heart samples from control non trisomic fetuses (includes the samples: H1, H2, H3, H4, H5).

The Affymetrix HG-U133A chip includes 262 probe sets that correspond to 168 known genes mapping on Hsa21. Eighty-one probe sets, corresponding to 63 individual genes, were called present in all analyzed samples. If we consider genes called present in at least 10 out of 15 samples, 117 probe sets, corresponding to 87 Hsa21 genes, might be regarded as being expressed in the human heart at 18–22 weeks of gestation (see Additional file 2). To investigate whether Hsa21 genes were upregulated in the heart tissue of DS fetuses, we compared the mean raw log-transformed gene expression data from DS heart tissues (DSH group) with the mean control sample values (NH group). The DSH/NH ratio was 1.48 ± 0.35 for Hsa21 genes and ~1 for all chromosomes or for any other chromosome (Figure 1). This difference was highly significant (p < 0.0001; ANOVA test with Bonferroni post-hoc). Scatter plot of DSH versus NH expression data shows that more than 75% of Hsa21 genes had a DSH/NH ratio above 1, whereas genes mapping on all other chromosomes were almost equally distributed above and below the line corresponding to the ratio = 1 (see Additional file 3). The level of variation of expression of Hsa21 genes was not very high, with a fold change ranging from 1.2 to 3. Twenty-five genes had a fold change below 1.2 (see Additional file 2). Quantitative real-time PCR (qRT-PCR) analyses performed on 14 Hsa21 genes, either upregulated or not in DS samples, were in good agreement with the microarray results (Table 2).

**Table 2:** Average expression ratios (DS samples/controls) calculated from microarray and RT-PCR data and Pearson correlation coefficient (r) between microarray and RT-PCR data. Hsa21 genes are in bold.

**Functional Category**	**Probe ID**	**Gene Name**	**GenBank**	**Micro-array Fold change**	**Micro-array p-value**	**RT-PCR ratio**	**r**	**Primers for RT-PCR**
**Oxydative Phospho-rylation**	201226_at	NDUFB8	NM_005004	0.65	0.0160	0.76	0.78	**LEFT**-GCCAAGAAGTATAATATGCGTGTG **RIGHT**-GTCAGGGAGCTTCGGGTAG
	201740_at	NDUFS3	NM_004551	0.76	0.0294	0.60	0.88	**LEFT**-GATTATGGCTTCGAGGGACA **RIGHT**-ACCCGCTTCACTTCATCATC
	201903_at	UQCRC1	NM_003365	0.77	0.0142	0.89	0.84	**LEFT**-CCGAGCAGTCCTCTCAGC **RIGHT**-TGTTCCCTTGAAAGCCAGAT
	201966_at	NDUFS2	NM_004550	0.67	0.0010	0.58	0.89	**LEFT**-GAATGGGCACAGCAGTTTG **RIGHT**-GGCCCAAAGTTCAGGGTAAT
	203606_at	NDUFS6	NM_004553	0.75	0.0464	0.63	0.84	**LEFT**-AGAAGGTCACGCACACTGG **RIGHT**-CACGGGCTGCTCTGCTAT
	203663_s_at	COX5A	NM_004255	0.79	0.0400	1.04	0.79	**LEFT**-AACTGGGCCTTGACAAAGTG **RIGHT**-GGTAACTGTTCACACTCAAGTAGCA
	203858_s_at	COX10	NM_001303	0.77	0.0101	0.66	0.92	**LEFT**-CTTTTGACTGGCCCTGTTTC **RIGHT**-ACCAGCGGTCTGTTCTTTGT
	218160_at	NDUFA8	NM_014222	0.73	0.0445	0.71	0.94	**LEFT**-GTCATGCCGGGGATAGTG **RIGHT**-TTAAGCACAGCAGAACTAATTTTCA
	218190_s_at	UCRC	NM_013387	0.76	0.0017	0.76	0.89	**LEFT**-GACGCTATCTACGACCACATCA **RIGHT**-GGTCCTTCTGGCCTGGAT

**Extra-cellular Matrix**	201069_at	MMP2	NM_004530	1.59	0.0069	1.89	0.93	**LEFT**-TCCACCACCTACAACTTTGAGA **RIGHT**-AACTTGCAGGGCTGTCCTT
	202310_s_at	COL1A1	K01228	1.55	0.0333	5.86	0.90	**LEFT**-TGTTCAGCTTTGTGGACCTC **RIGHT**-CTGTACGCAGGTGATTGGTG
	202403_s_at	COL1A2	NM_000089	1.60	0.0192	3.59	0.77	**LEFT**-CACATGCCGTGACTTGAGAC **RIGHT**-TAGCATCCATAGTGCATCCTTG
	202994_s_at	FBLN1	Z95331	1.72	0.0271	2.38	0.89	**LEFT**-GCCACAAGTGCGAGAACAC **RIGHT**-TAGACGTTGGCACACTCCTG
	209081_s_at	**COL18A1**	AF018081	1.57	0.0079	1.91	0.95	**LEFT**-GTGGCCCTCTACGTGGACT **RIGHT**-TCTGAGTCATCGCCTTCCTC
	213428_s_at	**COL6A1**	AA292373	1.62	0.0493	2.20	0.78	**LEFT**-AGGAGACCCTGGTGAAGCTG **RIGHT**-AGGTCCTGGGGCTCCTCT
	209156_s_at	**COL6A2**	AY029208	2.39	0.0002	2.18	0.79	**LEFT**-GACCTGGTCGCTGAGAAGTT **RIGHT**-GCCTTGTGGAAGTTCTGCTC

**Other genes**	205548_s_at	**BTG3**	NM006806	1.68	0.0134	1.62	0.83	**LEFT**-GAGGCAGTTGAGAGGTTTGC **RIGHT**-GAGTGAGCTCCTTTGGCAAG
	35776_at	**ITSN1**	AF064243	1.72	0.0003	1.57	0.88	**LEFT**-GTGAGCGGCACTGATTTGT **RIGHT**-GATCATGCTTCGCTCTTTCC
	205593_s_at	**PDE9A**	NM_002606	2.05	0.0067	2.05	0.89	**LEFT**-CAGAACGCACTCCGTACAAA **RIGHT**-TGGGCTCTACCTGTCCACTT
	211065_x_at	**PFKL**	BC006422	1.42	0.0088	1.97	0.81	**LEFT**-GGTGGACCTGGAGAAGCTG **RIGHT**-TCCAGGCGGAGTCAATGT
	200642_at	**SOD1**	NM_000454	1.16	>0.05	0.65	0.83	**LEFT**-GCATCATCAATTTCGAGCAG **RIGHT**-CAGCCTCTGTATTATCTCCAA
	203405_at	**DSCR2**	NM_003720	1.15	>0.05	0.85	0.78	**LEFT**-AAAGACTCGGCGTGTTGTC **RIGHT**-GAATTGCTGGGATTTTCCAT
	202671_s_at	**PDXK**	NM_003681	1.12	>0.05	1.00	0.98	**LEFT**-CATACAGAGCCACGTCATCC **RIGHT**-GCATAGCCTGTGTGGTTTGA
	202325_s_at	**ATP5J**	NM_001685	1.00	>0.05	1.17	0.78	**LEFT**-TGTTTGGCTTCTGTCTCACC **RIGHT**-GGCTGACCGAATGACAGAG
	202749_at	**WRB**	NM_004627	1.17	>0.05	1.25	0.85	**LEFT**-CTCAGCTTCGTGTTTGGATG **RIGHT**-ACTGTGGAGAGCTCCTGCTT
	209033_s_at	**DYRK1A**	D86550	1.53	0.0001	1.71	0.84	**LEFT**-GATATCATATGGGTCAGGTCATTTT **RIGHT**-CTGGACTGTAACATAACACAGTATGC
	208370_s_at	**DSCR1**	NM_004414	1.25	>0.05	4.35	0.83	**LEFT**-TTTGCTCAGACCTTACACATAGGA **RIGHT**-GGGAGGGGAGATCAGAAACT
	210555_s_at	NFATC3	U85430	0.45	>0.05	0.54	0.97	**LEFT**-CTTTGCAATGGCAAGAGGA **RIGHT**-GATGAGGCACAGGCAAAGAT
	217526_at	NFATC2	AI478300	0.77	0.019	0.67	0.84	**LEFT**-GAGTTCACATCCCAGAGTCCA **RIGHT**-GAGCACTCGATGGGGTTAGA

### Analysis of differentially expressed genes

**Figure 2:**
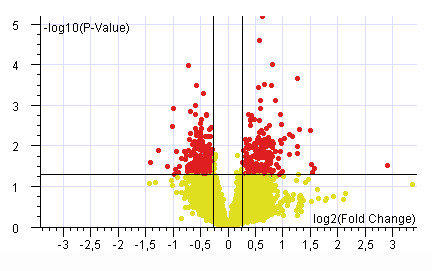
**Volcano plot of genes differentially expressed between trisomic and control samples**. The log2 of fold change between trisomic and control samples is represented on the x-axis and the negative log of p-values from the t-test is represented on the y-axis. Genes upregulated in the trisomic samples are on the right of the horizontal axis 0 value; genes downregulated are on the left. Red dots indicate 473 genes that are significantly up- or down-regulated in the trisomic samples compared to the control samples (p < 0.05). Yellow dots indicate genes with no significant variation.

We compared the gene expression levels in DSH versus NH fetuses. To this aim, raw expression data were normalized and pre-filtered to eliminate unreliable data, thus 8,966 probe sets, corresponding to ~6,300 genes, were considered for further analysis. Reliable gene expression data with fold change DSH vs. NH > |1.2| and p < .05 were filtered. A total of 473 genes were significantly either downregulated (278 genes) or upregulated (195 genes) in the DSH group versus the NH group (Figure 2). Thirty-two of the upregulated genes were located on Hsa21 and thereof 441 dysregulated genes were on different chromosomes (see Additional file 4).

Gene Ontology functional class scoring (GOTM software) was performed by comparing the list of differentially expressed genes to the complete set of genes spotted on the Affymetrix HG-U133A chip, chosen as reference list. Forty-four enriched categories for downregulated genes (Table 3) and 19 enriched categories for upregulated genes (Table 4) were identified. The ratios between observed and expected genes for the most represented GO categories and their p-values are reported (Tables 3 and 4). The most enriched GO cellular component categories were mitochondrial-related, for downregulated genes, and extracellular matrix (ECM)-related, for upregulated genes. At least 65 genes encoding mitochondrial enzymes were downregulated in the DS heart tissue out of the ~650 probe sets for mitochondrial proteins spotted on the HG-U133A chip (530 detected in heart tissue), and 40 genes out of the ~700 probe sets encoding ECM proteins (340 detected in the heart tissue), were upregulated in DSH samples (see Additional file 5).

**Table 3:** Enriched GO categories for down-regulated genes, sorted by p-value of the comparison between observed and expected data

**GO Category**	**Observed**	**Expected**	**Ratio**	**p-value**
**Biological Process**				
gener. of precursor metabolites and energy	30	9.78	3.07	3.31E-08
oxidative phosphorylation	9	1.28	7.03	4.43E-06
ATP synth. Coupled electron transp.	6	0.49	12.24	6.55E-06
coenzyme metabolism	12	2.65	4.53	1.26E-05
mitochondrial electron transport	5	0.41	12.2	3.84E-05
acetyl-CoA metabolism	5	0.56	8.93	0.000193
electron transport	15	5.51	2.72	0.000408
main pathways of carbohydrate metabolism	8	1.92	4.17	0.00065
glucose catabolism	6	1.15	5.22	0.000966
monosaccharide catabolism	6	1.32	4.55	0.001998
hexose catabolism	6	1.32	4.55	0.001998
energy derivation by oxid. of organic comp.	9	2.84	3.17	0.00214
alcohol catabolism	6	1.35	4.44	0.00217
Glycolysis	5	0.94	5.32	0.002329
porphyrin biosynthesis	3	0.28	10.71	0.002341
porphyrin metabolism	3	0.34	8.82	0.004372
group transfer coenzyme metabolism	5	1.11	4.5	0.004859
cellular carbohydrate catabolism	6	1.73	3.47	0.00756
carbohydrate catabolism	6	1.73	3.47	0.00756
glucose metabolism	6	1.77	3.39	0.008491

**Molecular Function**				

electron carrier activity	9	1.14	7.89	1.7E-06
NADH dehydrogenase activity	7	0.69	10.14	4.11E-06
hydrogen ion transporter activity	12	2.39	5.02	4.48E-06
oxidoreductase activity, acting on NADH/NADPH	8	1.02	7.84	6.63E-06
sodium ion transporter activity	7	0.75	9.33	7.61E-06
primary active transporter activity	13	3	4.33	8.97E-06
monovalent inorganic cation transporter activity	12	2.58	4.65	9.83E-06
metal ion transporter activity	9	1.56	5.77	2.34E-05
oxidoreductase activity	23	10.05	2.29	0.000172
electron transporter activity	12	4.37	2.75	0.001446
carrier activity	16	6.91	2.32	0.001523
metal cluster binding	4	0.52	7.69	0.001633
iron-sulfur cluster binding	4	0.52	7.69	0.001633
unfolded protein binding	8	2.62	3.05	0.004727

**Cellular component**				

mitochondrion	48	10.2	4.71	3E-20
mitochondrial envelope	10	2.45	4.08	0.000163
mitochondrial inner membrane	8	1.61	4.97	0.000194
mitochondrial membrane	9	2.07	4.35	0.000217
organelle envelope	13	4.21	3.09	0.000296
envelope	13	4.25	3.06	0.000326
organelle inner membrane	8	1.8	4.44	0.000417
membrane-enclosed lumen	19	8.37	2.27	0.000692
organelle lumen	19	8.37	2.27	0.000692
nucleolus	6	1.82	3.3	0.009655

**Table 4:** Enriched GO categories for upregulated genes, sorted by p-value

**GO categories**	**Observed**	**Expected**	**Ratio**	**p-value**
**Biological process**				
phosphate transport	8	0.98	8.16	5.27E-06
cell adhesion	22	8.15	2.7	1.81E-05
anion transport	9	2.15	4.19	0.000297
inorganic anion transport	8	1.77	4.52	0.000378
axonogenesis	5	0.66	7.58	0.000471
neuron morphogen. during different.	5	0.74	6.76	0.000807
neurite morphogenesis	5	0.74	6.76	0.000807
neuron development	5	0.98	5.1	0.002889
cell development	6	1.45	4.14	0.003197
neuron differentiation	5	1.14	4.39	0.005565
neurogenesis	5	1.19	4.2	0.006627
				
**Molecular Function**				
extracellular matrix struct. const.	10	1.25	8	4.11E-07
integrin binding	4	0.43	9.3	0.000833
copper ion binding	5	0.82	6.1	0.00128
				
**Cellular Component**				
extracellular matrix	22	3.55	6.2	5E-12
collagen	8	0.5	16	2.08E-08
extracellular region	35	14.17	2.47	2.83E-07
fibrillar collagen	4	0.16	25	1.24E-05
actin cytoskeleton	8	2.78	2.88	0.006602

**Figure 3:**
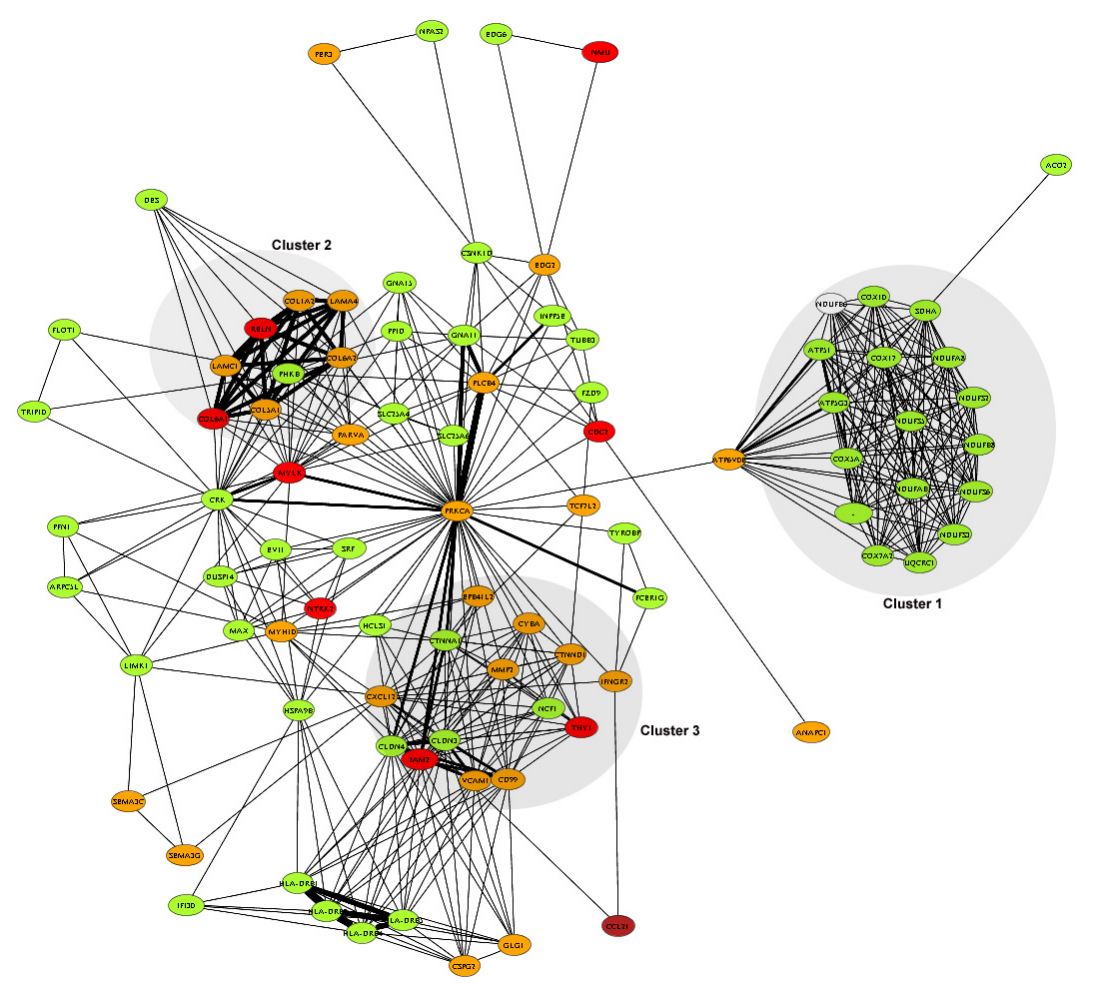
**Genes and gene pathways affected by Hsa21 trisomy**. Pathway analysis was performed with Pathway Miner software on the 473 genes dysregulated in trisomic samples. The most affected pathways are: Oxidative Phosphorylation (cluster 1), containing 16 genes downregulated in trisomic samples, and Focal Adhesion (cluster 2), containing at least 7 genes upregulated in trisomic samples. Cluster 3 is a network of Cell Adhesion genes, mostly upregulated in trisomic samples. Downregulated genes in cluster 1 are all mitochondrial genes; upregulated genes in clusters 2 and 3 are mostly ECM genes. Green indicates downregulated genes (darker green = more downregulated); red indicates upregulated genes (darker red = more upregulated).

Pathway analysis with Pathway Miner software revealed that 'oxidative phosphorylation' (OXPHOS) was the pathway most influenced by Hsa21 trisomy, because at least 16 genes out of the 119 represented on the chip, were downregulated in Hsa21 trisomy and no upregulated oxidative phosphorylation genes were detected (Figure 3). The second most affected pathway was 'focal adhesion', which contained upregulated genes that mostly encode ECM proteins (Figure 3).

To verify that these results were not affected by the methods of data processing and data analysis, a list of differentially expressed genes was generated after a different pre-processing method (gcRMA) and the functional analysis was performed with a different software tool, the Gene Set Enrichment Analysis (GSEA) [30]. A total of 532 genes (49 located on Hsa21), with a fold change higher than |1.2| and p < .05, were found to be differentially expressed in the comparison between DSH and NH samples (see Additional file 6). Using the GSEA to identify gene sets that correlated with the DS condition, we obtained five biologically informative gene sets (see Additional file 7). These included two sets of genes whose upregulation was highly correlated with DS (ECM and Cell Adhesion), and three sets of genes whose downregulation was highly correlated with DS (Mitochondria, Electron Transport Chain and OXPHOS). These results are in very good agreement with functional analysis carried out with MAS 5.0 pre-processed data and the GOTM and Pathway Miner web tools.

Unsupervised classification of samples based on the similarity of expression data across a gene list of ~900 genes, not mapping to Hsa21 and encoding mitochondrial and ECM proteins, yielded a condition tree in which trisomic samples are perfectly separated from control samples (see Additional file 8).

The results of microarray analysis were confirmed by qRT-PCR for 9 OXPHOS genes and for 7 ECM genes (Table 2).

### Expression of DYRK1A, DSCR1 and NFATc genes

**Figure 4:**
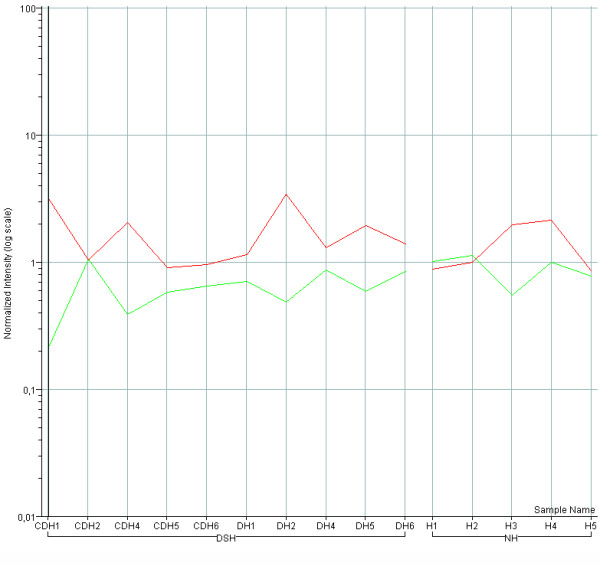
**Expression level of DSCR1 and NFATC3 genes in individual heart samples**. Microarray expression values of DSCR1 (red line) and NFATC3 (green line) genes of individual trisomic hearts (CDH1, CDH2, CDH4, CDH5, CDH6 and DH1, DH3, DH4, DH5, DH6) and individual normal hearts (H1, H2, H3, H4, H5) show an inverse correlation (r = -066) between the two genes.

The calcineurin/NFAT signaling pathway is known to be a critical regulator of organogenesis [31] and the NFATc transcription factors are transiently expressed in the endocardial cushions during heart septation [32]. The DSCR1 and DYRK1A genes, both mapping on Hsa21 within the critical region for DS, act synergistically to prevent nuclear translocation of NFATc transcription factors and may cause their downregulation [33]. We therefore examined the expression of DYRK1A and DSCR1 and of genes of the calcineurin pathway (NFATc1–4) in DSH and NH samples. DYRK1A was upregulated and NFATc2 downregulated in the trisomic heart samples (Table 2, p < .05). NFATc3 was also downregulated (p < .06). DSCR1 expression in fetal hearts varied greatly among individual samples. It was not overall significantly up- or downregulated, but its expression pattern was inversely correlated with that of NFATc3, independently of trisomy or cardiopathy (Figure 4). The Pearson correlation coefficient (r) was equal to -0.66 (r2 = 0.44), p < 0.01.

### Comparison between DS heart samples with and without CHD

**Figure 5:**
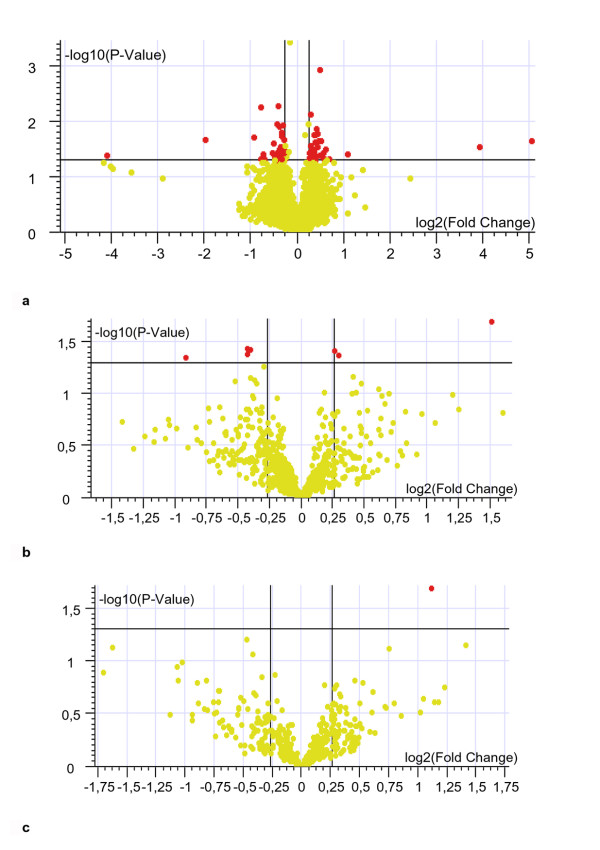
**Volcano plots obtained by comparing DS fetuses with and without cardiac defects**. Forty-two of the 15,000 analyzed genes are differentially expressed in the 5 samples with cardiac anomalies (CDH) and the 5 samples without cardiac anomalies (DH) (6a). Only a few genes of the ~600 encoding mitochondrial proteins (6b) and of the ~700 encoding ECM proteins (6c), are dysregulated in the CDH vs. DH comparison. Red dots indicate genes differentially expressed with fold change >1.2 and p < .05; yellow dots indicate genes with no significant variation.

The differential expression analysis of DS fetuses with (CDH) and without (DH) cardiac defects revealed a strong homogeneity between the 2 groups. Only 42 genes were differentially expressed, 19 upregulated and 23 downregulated in the CDH group, with a fold change ranging from |1.2| to |2|, p < .05 (Figure 5a). None of these genes mapped on Hsa21. The same functional analysis that was used to compare trisomic and control samples did not produce any significant result when genes differentially expressed in CDH vs. DH were considered. Similarly, there were no differences in the expression of mitochondrial and ECM genes between DS fetuses with and without heart defects (Figure 5b–5c).

## Discussion

Using DNA microarray analysis we have obtained information about transcripts that are present in the human fetal heart at 18–22 weeks of gestation. The data indicate that approximately 50% of the 15,000 analyzed genes are expressed in human fetal heart. The percentage of Hsa21 genes expressed in fetal hearts was not very different.

### Upregulation of Hsa21 genes

We found that Hsa21 genes are upregulated in all trisomic fetal hearts. This demonstrates that the concept of increased transcription of Hsa21 genes in Hsa21 trisomy applies to the human heart irrespective of CHD. The level of upregulation is modest. If we consider the mean for all Hsa21 genes analyzed, the fold increase in Hsa21 trisomy hearts versus controls is close to 1.5. This result is highly significant and confirms that microarrays can detect even small differences in gene expression levels.

This modest gene upregulation is in agreement with data obtained in developing human brain and heart [16,17], as well as in the cerebellum [21,22] and in different tissues [18-20] of adult Ts65Dn and Ts1Cje mice, two animal models of Hsa21 trisomy. Despite the different experimental conditions, an overall fold increase of ~1.5, which is consistent with a 3:2 ratio between trisomic and euploid fetuses, was reported in all these studies. However, the global 1.5-fold increase does not simply result from a 1.5-fold upregulation of each single gene. Indeed, whereas the fold increase for most genes is between 1.4 and 1.8, 8 genes are upregulated more than 2-fold in the DSH group and 25 genes are not upregulated in the DSH and NH groups. These results, which have been confirmed by qRT-PCR and are in agreement with another study [16], are indicative of either compensatory effects or heterogeneity in the regulatory mechanisms of Hsa21 genes. The gene expression level of individual Hsa21 genes in the heart of DS individuals is of central relevance in the effort to understand how Hsa21 trisomy causes CHD. Upregulation of a single gene or a combination of dysregulated genes might be at the base of CHD. Measurement of the levels of proteins corresponding to upregulated genes may shed light on the link between Hsa21 trisomy and CHD; a limited amount of information is available on this issue [34-36].

### Downregulation of mitochondrial genes

Our finding that 441 extra-Hsa21 genes were dysregulated in all trisomic samples supports the hypothesis that genes outside of Hsa21 might play a role in determining DS features [37]. Functional analysis of the genes differentially expressed between trisomic and control samples shows global downregulation of genes encoding mitochondrial proteins, especially enzymes involved in the oxidative phosphorylation pathway. Genes from all five complexes were downregulated suggesting that the corresponding proteins and enzymatic activities might be reduced, and that the mitochondrial function could be consequently impaired.

Protein levels of complex I, III and V were found to be decreased in cerebellar and brain regions [38,39], and a reduction of mitochondrial enzyme activity has been reported in platelets from DS patients [40]. Moreover, impaired mitochondrial function, indicated by reduced mitochondrial redox activity and membrane potential, has been observed in DS astrocytes and in primary cultures of DS fibroblasts, [26,27]. More recently it has been reported that the brain of the DS mouse model Ts1Cje has decreased mitochondrial membrane potential and ATP production [25]. These results are indicative of widespread mitochondrial dysfunction in DS. Our data suggest that mitochondrial dysfunction might also occur in DS hearts and that the reduced expression of mitochondrial genes might be the molecular basis of this dysfunction in the heart as well as in other DS tissues. This is probably not a primay effect of gene dosage because genes for mitochondrial function mapping on Hsa21 are either not expressed or not differentially expressed in human fetal hearts.

### Upregulation of ECM genes

Functional analysis of the 473 differentially expressed genes also demonstrates global upregulation of ECM protein genes. This group includes genes encoding adhesion and ECM proteins that map on Hsa21 such as ADAMTS1, ADAMTS5, APP, JAM2, COL6A1, COL6A2 and COL18A1, which are dose-dependently upregulated in trisomic samples, and genes that do not map on Hsa21 such as fibronectin, fibulin, collagen type I, type III, type V, type XV, metalloproteases (MMPs) and several adhesion molecule genes. Overexpression of this gene family is likely to affect cell adhesion properties, possibly determining an increase in adhesiveness. Cells explanted from endocardial cushion derived structures of fetuses with Hsa21 trisomy are more adhesive *in vitro *than those from controls [41]. A stochastic model has been proposed for septal defects in DS by which higher values of adhesiveness result in deficiencies of the atrio-ventricular canal development associated with clinical variability among individuals based on chance alone [42,43].

Several of the ECM genes upregulated in trisomic samples have been investigated for their potential role in DS cardiopathy. These include collagen type VI and MMPs. Collagen type VI is expressed in the endocardial cushions during septation, persists during valve differentiation and is implicated in endocardial cushion differentiation. Its pronounced expression in Hsa21 trisomy might lead to AVSD [44]. MMPs are involved in cardiac organogenesis by regulating cell proliferation, epithelial to mesenchymal transition, ECM remodeling and degradation. MMP2, in particular, coordinates prelooping stages, heart tube formation and selective ECM degradation. It has an important functional role in early cardiogenesis, neural crest cell and cardiac cushion migration and remodeling of the pharyngeal arches and cardiac heart tube [45,46].

It is interesting that DNA microarray analysis from right ventricular biopsies of patients with tetralogy of Fallot demonstrated that genes encoding ECM proteins, such as collagen type I, III, IX, XV and fibronectin, were upregulated versus age-matched controls [47], suggesting that the increase of these ECM proteins has a potential role in CHD.

### How might Hsa21 gene upregulation affect the expression of other genes?

A key issue of DS pathogenesis is to understand how upregulation of Hsa21 genes might dysregulate genes on different chromosomes. Recently, a link has been proposed between two Hsa21 genes, DYRK1A and DSCR1, and the NFATc family of genes. NFATc-null mice show phenotypic anomalies that resemble those observed in human DS and 65% of NFATc1–4-null mice have endocardial cushion defects [33]. Even modest overexpression of DYRK1A decreases NFATc protein activity and levels and may induce vascular and cardiac defects [33]. DSCR1 also encodes a regulatory protein that is expressed in heart tissue [48] and exerts an inhibitory effect on the calcineurin/NFAT signaling pathway [49], although different protein isoforms may have opposite effects [50]. To verify whether these genes affect the heart of DS fetuses, we evaluated their differential expression both by microarray and by qRT-PCR. Our analyses show that DYRK1A is upregulated and that NFATc2 and NFATc3 are downregulated in Hsa21 trisomic samples. Moreover, an increase in DSCR1 expression in individual hearts correlated with a decrease in NFATc3 expression. Recent data show that the enzymatic activity of complex II and IV of the respiratory chain and mitochondrial oxidative activity are reduced in Nfatc3-/-; Nfatc4-/- cardiomyocytes [51] suggesting that the calcineurin/NFAT pathway affects mitochondrial activity during heart development. We propose that upregulation of DIRK1A and/or DSCR1 in DS might affect mitochondrial gene expression, and thereafter mitochondrial function, through the calcineurin/NFAT pathway. Mitochondrial abnormalities and a decrease of COX activity might also be induced by overproduction of beta APP [52], a gene mapping on Hsa21, although the TS1Cje mouse model, in which APP is not triplicated, also shows decreased mitochondrial function and ATP production [25]. The transcription factor GABPalpha, which is encoded by a gene on Hsa21, is another regulator of the expression of genes involved in mitochondrial respiration [28]. However, GABPalpha was not expressed in fetal hearts at 18–22 weeks of gestation, although it cannot be excluded that it is expressed at different embryonic or fetal stages.

Type VI collagen gene upregulation also sheds light on the potential interplay between Hsa21 genes and genes on different chromosomes. Type VI collagen, together with other collagens, is an activator of discoidin domain receptor tyrosine kinases (DDRs) [53,54]. Activated DDRs, in turn, induce the expression of metalloproteases such as MMP1 and MMP2 [53] and of other ECM proteins [55]. Moreover, DDR1 and DDR2 are upregulated in trisomic heart samples (see Additional file 4) and DDR2 expression has been detected by confocal microscopy in developing heart, within the cardiac cushions and eventually within the septum [56].

## Conclusion

The expression of most Hsa21 genes and of many genes located on other chromosomes were dysregulated in the heart of trisomic fetuses at 18–22 weeks of gestation. Genes encoding mitochondrial enzymes were significantly downregulated, whereas genes encoding ECM proteins were upregulated in all trisomic hearts, irrespective of CHD. In fact, gene expression did not differ between DS heart samples with or without cardiopathy. This might be due to some intrinsic limits of our approach (e.g., heart developmental stage, and number of samples and genes analyzed). We nevertheless demonstrate that the expression of several gene categories is dysregulated in the hearts of all DS fetuses, and suggest that this dysregulated expression might be a prelude to heart defects. Other factors, such as differences in the genetic background, different Hsa21 haplotypes, stochastic and/or environmental factors, could play a critical role in determining the final pathogenetic result. Finally, non-coding mRNAs as well as conserved non-genic sequences, which have been described within Hsa21, might be implicated in determining the DS phenotype [57].

## Methods

### Experimental design

Fifteen human fetuses from 18- to 22-week-gestation with and without Hsa21 trisomy were analyzed. Ten were trisomic for Hsa21; 5 non trisomic fetuses served as controls. Diagnosis, gestational age, gender and karyotype are provided in Table 1. Control samples were euploid fetuses without cardiac anomalies. Fetuses H1 and H5 were aborted because of the mother's condition; they did not show any disorder at autopsy examination. Two other fetuses were affected by genetic anomalies: fragile X syndrome (H2) and thalassemia (H4). Fetus H3 was affected by severe hydropia. The experimental design was diseased versus control comparison. The conditions tested were gene expression at 18–22 weeks of gestation and genetic variation.

### Samples

Heart tissue was used for total RNA extraction. All cardiac samples were obtained from the Telethon Bank of Fetal Biological Samples at the University of Naples. We used protocols approved by our Institutional Ethics Committee. The hearts were explanted from fetuses after therapeutic abortion. The posterior half, representative of all four chambers, was dissected by a longitudinal cut and immediately frozen in liquid nitrogen and stored at -135°C for RNA extraction. The anterior half was paraffin-embedded. Fetal karyotype was determined on amniocytes, and was confirmed on cultured fibroblasts, by standard cytogenetic methods with G banding.

### Microarray hybridization procedure

All experiments were performed with Affymetrix HG-U133A oligonucleotide arrays (Affymetrix, Santa Clara, CA), as described at [58]. Total RNA from each sample was extracted using TRIzol reagent (Gibco/BRL Life Technologies, Inc., Gaithersburg, MD) and used to prepare biotinylated target cRNA, according to the Affymetrix recommendations [58]. Purification of PolyA+ mRNA from total RNA was performed with the Oligotex mRNA Kit (QIAGEN GmbH, Hilden, Germany): 1 μg of mRNA was used to generate first-strand cDNA by using a T7-linked oligo(dT) primer; after second-strand synthesis, *in-vitro *transcription was performed with biotinylated UTP and CTP using the Enzo BioArray High Yield RNA Transcript Labeling Kit (Enzo Diagnostics, Farmingdale, NY), resulting in approximately 100-fold amplification of RNA. The target cRNA generated from each sample was processed as recommended by the manufacturer and using an Affymetrix GeneChip Instrument System. Fragmentation of biotinylated cRNA, washing and staining were done according to the instructions provided by Affymetrix. Briefly, spike controls were added to 10 μg fragmented cRNA before overnight hybridization. Arrays were then washed and stained with streptavidin-phycoerythrin, before being scanned on an Affymetrix GeneChip scanner. Quality and amount of starting RNA was confirmed using spectrophotometry and agarose gel electrophoresis. A detailed description of these procedures is available [58].

### Data acquisition and processing

After scanning, array images were assessed by eye to confirm scanner alignment and the absence of significant bubbles or scratches on the chip surface. 3'/5' ratios for GAPDH and beta-actin were confirmed to be within acceptable limits (0.70–1.64), and BioB spike controls were found to be present on all chips, with BioC, BioD and CreX also present in increasing intensity. Expression data were deposited in the Gene Expression Omnibus repository [59] with experiment accession number GSE1789.

Using the Affymetrix Software Microarray Suite (MAS 5.0), each probe set was assigned an "average difference" value corresponding to the expression level of the particular gene it represented. To make comparisons across different chips, data sets on each chip were scaled to a targeted total fluorescence of 100. When scaled to a target intensity of 100 (using Affymetrix MAS 5.0 array analysis software), scaling factors for all arrays were within acceptable limits (0.69–1.51), as were background, Q values and mean intensities. Affymetrix software also assigns each probe set an absolute call (present, absent or marginal), which represents a qualitative indication of whether or not a transcript is detected within a sample.

Array scanning data (CEL files) were also pre-processed using the gcRMA algorithm [60]. Raw data were prefiltered to exclude fluorescence intensities lower than 10, which are indistinguishable from background. Unreliable genes were also discarded using the cross gene error model [61]. This analysis showed an overall 90% agreement with the MAS 5.0 data analysis; 40% of this agreement was for fold change only, but the p-value was higher.

GeneSpring software (Silicon Genetics, Redwood City, CA) was used for data mining. Raw expression data were normalized per gene by dividing each measurement for each gene by the median of that gene's measurements in the corresponding control non trisomic samples. Normalized data were log-transformed. Based on Affymetrix absolute call, we determined the total number of genes that were expressed (i.e. called present) in human fetal heart.

Although the Affymetrix chip HG-U133A can measure the expression of ~15,000 genes, the true transcript level is often affected by a substantial amount of noise and variability induced by many sources including the manufacturing processes and the experimental procedures [62]. We pre-filtered expression data to reduce noise and so discard "unreliable" genes. The Cross-Gene error model [61] was applied to estimate measurement precision by combining variability of gene expression data and assuming that measurements with higher control strength are relatively more precise than measurements with lower control strength. After pre-filtering, genes were considered suitable for differential evaluation if called present in at least 2 out of 15 samples and with a raw signal higher than 10.0. Statistical evaluation of the differential analysis was performed by one way ANOVA. The threshold for statistical significance was set to 0.05.

Unsupervised classification of samples was performed using the hierarchical clustering tool included in the GeneSpring software. A condition tree was generated grouping together DSH or NH samples based on the similarity of their expression data in the specified list.

### Bioinformatics data analyses

Gene ontology functional class scoring was performed using the web-based GOTM software [63,64] which visualizes genes from the list of differentially expressed genes in the GO context, considering as gene sets all the GO categories for biological processes, molecular functions and cellular components. The list of differentially expressed genes was compared to the complete list of genes spotted on Affymetrix HG-U133 chip, in order to identify categories of genes more represented in the list of differentially expressed genes than in the reference gene set.

Pathway analysis was performed by Pathway Miner software [65,66] which catalogs genes of a list based on their role in metabolic, cellular and regulatory pathways from three different pathway databases (GenMap, Encarta and KEGG). A Fisher exact test ranks pathways according to the number of genes of the list which co-occurrs in pathways, considered as gene sets. Furthermore a network is created among genes participating to multiple pathways.

Gene Set Enrichment Analysis (GSEA) was used as described [30] to identify gene sets correlated with the DS condition. The gcRMA output list of differentially expressed genes was ranked per fold change and was submitted to the GSEA tool. Enrichment of functional gene sets from the MSigDB C2 curated database [67] was tested. This database includes sets of genes whose products are involved in specific metabolic and signaling pathways from public databases and sets of genes coregulated in specific conditions. This last group of gene sets was not considered in the analysis. In turn, the Gene Ontology category of Extracellular Matrix genes (GO:31012, cellular component) was added into the MSigDB C2 catalog.

### Quantitative Real-Time PCR

We used the same sources of total RNA for both primary gene expression and validation experiments. cDNA was synthesized with random hexamer primers starting from 1.5 μg of total RNA using the reverse transcription protocol (Taqman Reverse Transcription, Applied Biosystems, Applera, Monza, Italia). Real-time PCR was performed using iQ Supermix SYBR Green 2X [68] on the Bio-Rad iCycler [68] according to the manufacturer's protocols. PCR reactions were performed in triplicate. The primers (MWG Biotech, Ebersberg, Germany) used for amplification are listed in Table 2. Primer pairs were designed using the Primer 3 software [69] to obtain amplicons ranging from 100 to150 base pairs, and specifically designed to span introns or cross intron-exon boundaries. In order to test primer efficiency, serial dilutions of cDNAs generated from selected human fetal hearts, that expressed target genes at a suitable level, were used to generate standard curves for each gene. RPL13A and GAPDH housekeeping genes were chosen as reference genes.

## List of abbreviations used

DS = Down syndrome

Hsa21 = Chromosome 21

CHD = Congenital heart defects

DSH = Heart samples from fetuses with Hsa21 trisomy

NH = Heart samples from non trisomic fetuses

DH = Samples without cardiac anomalies

CDH = Samples with cardiac anomalies

ECM = Extracellular matrix

OXPHOS = Oxidative phosphorylation

## Authors' contributions

AC supervised the molecular studies, performed the data analysis and drafted the manuscript. FF performed the molecular studies. PDA supervised and participated in the tissue collection and sample preparation. RN contributed to writing the manuscript. DG participated in the data analysis. RG participated in the characterization of the samples. MDA and CO were responsible for tissue excision and pathology examination. DP performed fetal heart ultrasound analyses and participated in the sample collection. MZ participated in the design of the study and in the molecular studies. LN was responsible for the coordination and supervision of the entire study.

## Supplementary Material

Additional file 1 Genes expressed in human fetal heart at 18–22 weeks of gestation. Genes are reported if the Affymetrix presence call was 'Present' in at least 10 heart samples. Genes are sorted by alphabetical order. Click here for file

Additional file 2 Hsa21 genes expressed in the human fetal heart at 18–22 weeks of gestation. Genes (probe sets) are sorted according to chromosomal location. Mitochondrial and ECM genes are in bold. Click here for file

Additional file 3 Scatter plot of gene expression data of trisomic samples vs. control samples. Mean raw, log transformed, gene expression data from the 5 control samples (NH) were plotted on the x-axis and data from the 10 trisomic samples (DSH) were plotted on the y-axis. Plots are shown for Hsa21 genes and for genes of all chromosomes excluding Hsa21. In the plot of Hsa21 more than 75% of gene probe sets are above the line, whereas in the plot of all other chromosomes approximately the same number of gene probe sets is above and below the line. Abbreviations for DSH and NH are as in Figure 1. Click here for file

Additional file 4 Genes differentially expressed between trisomic and control samples. The table includes genes with fold change > |1.2| and p < 0.05 (ANOVA test). Genes are sorted according to fold change. Click here for file

Additional file 5 List of downregulated genes encoding mitochondrial proteins, and of upregulated genes encoding extracellular matrix proteins. Hsa21 genes are in bold. Click here for file

Additional file 6 Genes differentially expressed between trisomic and control samples using gc-RMA preprocessed data. The table includes genes with fold change > |1.2| and p < 0.05 (ANOVA test). Genes are sorted according to fold change. Hsa21 genes are in bold. Click here for file

Additional file 7 Enrichment score plots of the five biologically informative sets correlated to the DS condition with an FDR value < 0.05. Extracellular matrix and Cell adhesion gene sets are positively correlated to DS condition whereas Mitochondria, Electron transport chain and Oxidative phosphorylation gene sets are negatively correlated. The enrichment score (ES) represents the degree to which a gene set is enriched at the top (positive ES) or at the bottom (negative ES) of our ranked list. The size indicated for each gene set is the dimension of the leading edge subset that is the subset of members of our list that contribute more to the enrichment score (ES). The nominal p-value and the False Discovery Rate (FDR) value estimate the probability that the enrichment score represents a false positive finding. Click here for file

Additional file 8 Condition tree generated using the hierarchical clustering approach. The tree groups samples together based on the similarity of their expression data across a gene list including ~1000 genes, not mapping to Hsa21, which encode mitochondrial and ECM proteins. The 15 DS samples are clustered together on the left whereas the five control samples are clustered on the right of the image demonstrating that the expression of genes in the specified list can be used to correctly separate DSH from NH samples. Click here for file

## References

[B1] Epstein CJ, Korenberg JR, Anneren G, Antonarakis SE, Ayme S, Courchesne E, Epstein LB, Fowler A, Groner Y, Huret JL (1991). Protocols to establish genotype-phenotype correlations in Down syndrome. Am J Hum Genet.

[B2] Ferencz C, Neill CA, Boughman JA, Rubin JD, Brenner JI, Perry LW (1989). Congenital cardiovascular malformations associated with chromosome abnormalities: an epidemiologic study. J Pediatr.

[B3] Park SC, Mathews RA, Zuberbuhler JR, Rowe RD, Neches WH, Lenox CC (1977). Down syndrome with congenital heart malformation. Am J Dis Child.

[B4] Korenberg JR, Chen XN, Schipper R, Sun Z, Gonsky R, Gerwehr S, Carpenter N, Daumer C, Dignan P, Disteche C (1994). Down syndrome phenotypes: the consequences of chromosomal imbalance. Proc Natl Acad Sci U S A.

[B5] Delabar JM, Theophile D, Rahmani Z, Chettouh Z, Blouin JL, Prieur M, Noel B, Sinet PM (1993). Molecular mapping of twenty-four features of Down syndrome on chromosome 21. Eur J Hum Genet.

[B6] McCormick MK, Schinzel A, Petersen MB, Stetten G, Driscoll DJ, Cantu ES, Tranebjaerg L, Mikkelsen M, Watkins PC, Antonarakis SE (1989). Molecular genetic approach to the characterization of the "Down syndrome region" of chromosome 21. Genomics.

[B7] Rahmani Z, Blouin JL, Creau-Goldberg N, Watkins PC, Mattei JF, Poissonnier M, Prieur M, Chettouh Z, Nicole A, Aurias A (1989). Critical role of the D21S55 region on chromosome 21 in the pathogenesis of Down syndrome. Proc Natl Acad Sci U S A.

[B8] Barlow GM, Chen XN, Shi ZY, Lyons GE, Kurnit DM, Celle L, Spinner NB, Zackai E, Pettenati MJ, Van Riper AJ, Vekemans MJ, Mjaatvedt CH, Korenberg JR (2001). Down syndrome congenital heart disease: a narrowed region and a candidate gene. Genet Med.

[B9] Olson LE, Richtsmeier JT, Leszl J, Reeves RH (2004). A chromosome 21 critical region does not cause specific Down syndrome phenotypes. Science.

[B10] Olson LE, Roper RJ, Sengstaken CL, Peterson EA, Aquino V, Galdzicki Z, Siarey R, Pletnikov M, Moran TH, Reeves RH (2007). Trisomy for the Down syndrome "critical region" is necessary but not sufficient for brain phenotypes of trisomic mice. Hum Mol Genet.

[B11] Reeves RH (2001). Down's syndrome. A complicated genetic insult. Lancet.

[B12] Shapiro BL (1997). Whither Down syndrome critical regions?. Hum Genet.

[B13] Epstein CJ (1986). Developmental genetics. Experientia.

[B14] FitzPatrick DR, Ramsay J, McGill NI, Shade M, Carothers AD, Hastie ND (2002). Transcriptome analysis of human autosomal trisomy. Hum Mol Genet.

[B15] Giannone S, Strippoli P, Vitale L, Casadei R, Canaider S, Lenzi L, D'Addabbo P, Frabetti F, Facchin F, Farina A, Carinci P, Zannotti M (2004). Gene expression profile analysis in human T lymphocytes from patients with Down Syndrome. Ann Hum Genet.

[B16] Mao R, Wang X, Spitznagel EL, Frelin LP, Ting JC, Ding H, Kim JW, Ruczinski I, Downey TJ, Pevsner J (2005). Primary and secondary transcriptional effects in the developing human Down syndrome brain and heart. Genome Biol.

[B17] Mao R, Zielke CL, Ronald Zielke H, Pevsner J (2003). Global up-regulation of chromosome 21 gene expression in the developing down syndrome brain. Genomics.

[B18] Lyle R, Gehrig C, Neergaard-Henrichsen C, Deutsch S, Antonarakis SE (2004). Gene expression from the aneuploid chromosome in a trisomy mouse model of down syndrome. Genome Res.

[B19] Kahlem P, Sultan M, Herwig R, Steinfath M, Balzereit D, Eppens B, Saran NG, Pletcher MT, South ST, Stetten G, Lehrach H, Reeves RH, Yaspo ML (2004). Transcript level alterations reflect gene dosage effects across multiple tissues in a mouse model of down syndrome. Genome Res.

[B20] Amano K, Sago H, Uchikawa C, Suzuki T, Kotliarova SE, Nukina N, Epstein CJ, Yamakawa K (2004). Dosage-dependent over-expression of genes in the trisomic region of Ts1Cje mouse model for Down syndrome. Hum Mol Genet.

[B21] Dauphinot L, Lyle R, Rivals I, Dang MT, Moldrich RX, Golfier G, Ettwiller L, Toyama K, Rossier J, Personnaz L, Antonarakis SE, Epstein CJ, Sinet PM, Potier MC (2005). The cerebellar transcriptome during postnatal development of the Ts1Cje mouse, a segmental trisomy model for Down syndrome. Hum Mol Genet.

[B22] Saran NG, Pletcher MT, Natale JE, Cheng Y, Reeves RH (2003). Global disruption of the cerebellar transcriptome in a Down syndrome mouse model. Hum Mol Genet.

[B23] Gardiner K (2004). Gene-dosage effects in Down syndrome and trisomic mouse models. Genome Biol.

[B24] Gardiner K (2003). Predicting pathway perturbations in Down syndrome. J Neural Transm Suppl.

[B25] Shukkur EA, Shimohata A, Akagi T, Yu W, Yamaguchi M, Murayama M, Chui D, Takeuchi T, Amano K, Subramhanya KH, Hashikawa T, Sago H, Epstein CJ, Takashima A, Yamakawa K (2006). Mitochondrial dysfunction and tau hyperphosphorylation in Ts1Cje, a mouse model for Down syndrome. Hum Mol Genet.

[B26] Arbuzova S, Hutchin T, Cuckle H (2002). Mitochondrial dysfunction and Down's syndrome. Bioessays.

[B27] Busciglio J, Pelsman A, Wong C, Pigino G, Yuan M, Mori H, Yankner BA (2002). Altered metabolism of the amyloid beta precursor protein is associated with mitochondrial dysfunction in Down's syndrome. Neuron.

[B28] O'Leary DA, Pritchard MA, Xu D, Kola I, Hertzog PJ, Ristevski S (2004). Tissue-specific overexpression of the HSA21 gene GABPalpha: implications for DS. Biochim Biophys Acta.

[B29] Irizarry RA, Bolstad BM, Collin F, Cope LM, Hobbs B, Speed TP (2003). Summaries of Affymetrix GeneChip probe level data. Nucleic Acids Res.

[B30] Subramanian A, Tamayo P, Mootha VK, Mukherjee S, Ebert BL, Gillette MA, Paulovich A, Pomeroy SL, Golub TR, Lander ES, Mesirov JP (2005). Gene set enrichment analysis: a knowledge-based approach for interpreting genome-wide expression profiles. Proc Natl Acad Sci U S A.

[B31] Graef IA, Chen F, Crabtree GR (2001). NFAT signaling in vertebrate development. Curr Opin Genet Dev.

[B32] de la Pompa JL, Timmerman LA, Takimoto H, Yoshida H, Elia AJ, Samper E, Potter J, Wakeham A, Marengere L, Langille BL, Crabtree GR, Mak TW (1998). Role of the NF-ATc transcription factor in morphogenesis of cardiac valves and septum. Nature.

[B33] Arron JR, Winslow MM, Polleri A, Chang CP, Wu H, Gao X, Neilson JR, Chen L, Heit JJ, Kim SK, Yamasaki N, Miyakawa T, Francke U, Graef IA, Crabtree GR (2006). NFAT dysregulation by increased dosage of DSCR1 and DYRK1A on chromosome 21. Nature.

[B34] Dowjat WK, Adayev T, Kuchna I, Nowicki K, Palminiello S, Hwang YW, Wegiel J (2006). Trisomy-driven overexpression of DYRK1A kinase in the brain of subjects with Down syndrome. Neurosci Lett.

[B35] Cheon MS, Shim KS, Kim SH, Hara A, Lubec G (2003). Protein levels of genes encoded on chromosome 21 in fetal Down syndrome brain: Challenging the gene dosage effect hypothesis (Part IV). Amino Acids.

[B36] Greber-Platzer S, Schatzmann-Turhani D, Wollenek G, Lubec G (1999). Evidence against the current hypothesis of "gene dosage effects" of trisomy 21: ets-2, encoded on chromosome 21" is not overexpressed in hearts of patients with Down Syndrome. Biochem Biophys Res Commun.

[B37] Reeves RH, Baxter LL, Richtsmeier JT (2001). Too much of a good thing: mechanisms of gene action in Down syndrome. Trends Genet.

[B38] Kim SH, Vlkolinsky R, Cairns N, Lubec G (2000). Decreased levels of complex III core protein 1 and complex V beta chain in brains from patients with Alzheimer's disease and Down syndrome. Cell Mol Life Sci.

[B39] Kim SH, Vlkolinsky R, Cairns N, Fountoulakis M, Lubec G (2001). The reduction of NADH ubiquinone oxidoreductase 24- and 75-kDa subunits in brains of patients with Down syndrome and Alzheimer's disease. Life Sci.

[B40] Prince J, Jia S, Bave U, Anneren G, Oreland L (1994). Mitochondrial enzyme deficiencies in Down's syndrome. J Neural Transm Park Dis Dement Sect.

[B41] Wright TC, Orkin RW, Destrempes M, Kurnit DM (1984). Increased adhesiveness of Down syndrome fetal fibroblasts in vitro. Proc Natl Acad Sci U S A.

[B42] Kurnit DM, Aldridge JF, Neve RL, Matthysse S (1985). Genetics of congenital heart malformations: a stochastic model. Ann N Y Acad Sci.

[B43] Kurnit DM, Aldridge JF, Matsuoka R, Matthysse S (1985). Increased adhesiveness of trisomy 21 cells and atrioventricular canal malformations in Down syndrome: a stochastic model. Am J Med Genet.

[B44] Gittenberger-de Groot AC, Bartram U, Oosthoek PW, Bartelings MM, Hogers B, Poelmann RE, Jongewaard IN, Klewer SE (2003). Collagen type VI expression during cardiac development and in human fetuses with trisomy 21. Anat Rec A Discov Mol Cell Evol Biol.

[B45] Cai DH, Vollberg TM, Hahn-Dantona E, Quigley JP, Brauer PR (2000). MMP-2 expression during early avian cardiac and neural crest morphogenesis. Anat Rec.

[B46] Person AD, Klewer SE, Runyan RB (2005). Cell biology of cardiac cushion development. Int Rev Cytol.

[B47] Sharma HS, Peters TH, Moorhouse MJ, van der Spek PJ, Bogers AJ (2006). DNA microarray analysis for human congenital heart disease. Cell Biochem Biophys.

[B48] Lange AW, Rothermel BA, Yutzey KE (2005). Restoration of DSCR1 to disomy in the trisomy 16 mouse model of Down syndrome does not correct cardiac or craniofacial development anomalies. Dev Dyn.

[B49] Fuentes JJ, Genesca L, Kingsbury TJ, Cunningham KW, Perez-Riba M, Estivill X, de la Luna S (2000). DSCR1, overexpressed in Down syndrome, is an inhibitor of calcineurin-mediated signaling pathways. Hum Mol Genet.

[B50] Qin L, Zhao D, Liu X, Nagy JA, Hoang MV, Brown LF, Dvorak HF, Zeng H (2006). Down Syndrome Candidate Region 1 Isoform 1 Mediates Angiogenesis through the Calcineurin-NFAT Pathway. Mol Cancer Res.

[B51] Bushdid PB, Osinska H, Waclaw RR, Molkentin JD, Yutzey KE (2003). NFATc3 and NFATc4 are required for cardiac development and mitochondrial function. Circ Res.

[B52] Askanas V, McFerrin J, Baque S, Alvarez RB, Sarkozi E, Engel WK (1996). Transfer of beta-amyloid precursor protein gene using adenovirus vector causes mitochondrial abnormalities in cultured normal human muscle. Proc Natl Acad Sci U S A.

[B53] Vogel W, Gish GD, Alves F, Pawson T (1997). The discoidin domain receptor tyrosine kinases are activated by collagen. Mol Cell.

[B54] Vogel WF, Aszodi A, Alves F, Pawson T (2001). Discoidin domain receptor 1 tyrosine kinase has an essential role in mammary gland development. Mol Cell Biol.

[B55] Faraci E, Eck M, Gerstmayer B, Bosio A, Vogel WF (2003). An extracellular matrix-specific microarray allowed the identification of target genes downstream of discoidin domain receptors. Matrix Biol.

[B56] Morales MO, Price RL, Goldsmith EC (2005). Expression of Discoidin Domain Receptor 2 (DDR2) in the developing heart. Microsc Microanal.

[B57] Dermitzakis ET, Reymond A, Scamuffa N, Ucla C, Kirkness E, Rossier C, Antonarakis SE (2003). Evolutionary discrimination of mammalian conserved non-genic sequences (CNGs). Science.

[B58] Affymetrix Homepage. http://www.affymetrix.com/index.affx.

[B59] Gene Expression Omnibus. http://www.ncbi.nlm.nih.gov/projects/geo/.

[B60] Wu Z, Irizarry RA (2004). Preprocessing of oligonucleotide array data. Nat Biotechnol.

[B61] Jornsten R, Yu B (2003). Simultaneous gene clustering and subset selection for sample classification via MDL. Bioinformatics.

[B62] Bassett DE, Eisen MB, Boguski MS (1999). Gene expression informatics--it's all in your mine. Nat Genet.

[B63] Zhang B, Schmoyer D, Kirov S, Snoddy J (2004). GOTree Machine (GOTM): a web-based platform for interpreting sets of interesting genes using Gene Ontology hierarchies. BMC Bioinformatics.

[B64] Gene Ontology Tree Machine. http://bioinfo.vanderbilt.edu/gotm/.

[B65] Pandey R, Guru RK, Mount DW (2004). Pathway Miner: extracting gene association networks from molecular pathways for predicting the biological significance of gene expression microarray data. Bioinformatics.

[B66] Bio Resource for Array Genes. http://www.biorag.org/.

[B67] Gene Set Enrichment Analysis: Overview. http://www.broad.mit.edu/gsea/.

[B68] Bio-Rad. http://www.bio-rad.com.

[B69] Primer 3 Software. http://frodo.wi.mit.edu/cgi-bin/primer3/primer3_www.cgi/.

