# Supplementary material for: Altered expression of mitochondrial and extracellular matrix genes in the heart of human fetuses with chromosome 21 trisomy
Source: BMC Genomics. 2007 Aug 7;8:268. doi: 10.1186/1471-2164-8-268 (PMC1964766; doi:10.1186/1471-2164-8-268)
Supplement: Additional file 1 — Genes expressed in human fetal heart at 18–22 weeks of gestation. Genes are reported if the Affymetrix presence call was 'Present' in at least 10 heart samples. Genes are sorted by alphabetical order. [file 1471-2164-8-268-S1.pdf]

Table S1. – Genes expressed in human fetal heart at 18-22 weeks of gestation

Probe ID	Presence call	Gene Name	GenBank ID	Chromosomal location
208611_s_at	15	(ALPHA)II-SPECTRIN	U83867	9q33-q34
209665_at	13	101F6	AF040704	3p21.3
204072_s_at	15	13CDNA73	NM_023037	13q12.3
214318_s_at	14	13CDNA73	W58342	13q12.3
214319_at	15	13CDNA73	W58342	13q12.3
214711_at	12	15E1.2	BE568184	12q24.31
40640_at	13	384D8-2	U62317	22q13.33
211337_s_at	14	76P	BC000966	15q15
213266_at	15	76P	BF592982	15q15
44702_at	15	7h3	R77097	---
216272_x_at	15	7h3; FLJ13511	AF209931	19p13.13
201180_s_at	15	87U6	J03198	1p13
209447_at	15	8B7	AF043290	6q25
201129_at	15	9G8	L22253	2p22.1
1053_at	13	A1	M87338	7q11.23
202021_x_at	15	A121	AF083441	17q21.31
210632_s_at	11	A2	L35853	17q21
221217_s_at	15	A2BP1	NM_018723	16p13.3
217757_at	15	A2M	NM_000014	12p13.3-p12.3
219488_at	15	A4GALT	NM_017436	22q11.2-q13.2
218434_s_at	14	AACS	NM_023928	12q24.31
201511_at	15	AAMP	NM_001087	2q35
201000_at	15	AARS	NM_001605	16q22
205986_at	14	AATK	NM_004920	---
65493_at	14	ABC1	AA555088	17q23.2-q23.3
203504_s_at	15	ABCA1	AF285167	9q31.1
203505_at	15	ABCA1	AF285167	9q31.1
212772_s_at	14	ABCA2	AL162060	9q34
213353_at	15	ABCA5	BF693921	17q24.3
204719_at	15	ABCA8	NM_007168	17q24
207819_s_at	13	ABCB4	NM_000443	7q21.1
202804_at	15	ABCC1	AI539710	16p13.1
213485_s_at	12	ABCC10	AK000002	6p21.1
203196_at	10	ABCC4	AI948503	13q32
214033_at	12	ABCC6	AI084637	16p13.1
208561_at	13	ABCC9	NM_020297	12p12.1
208562_s_at	10	ABCC9	NM_020297	12p12.1
202850_at	15	ABCD3	NM_002858	1p22-p21
203981_s_at	15	ABCD4	AL574660	14q24.3
201872_s_at	15	ABCE1	NM_002940	4q31
201873_s_at	15	ABCE1	NM_002940	4q31
200045_at	15	ABCF1	NM_001090	6p21.33
207622_s_at	11	ABCF2	NM_005692	7q36
202394_s_at	14	ABCF3	NM_018358	3q27.3
204567_s_at	15	ABCG1	NM_004915	21q22.3
211113_s_at	14	ABCG1	U34919	21q22.3
218633_x_at	15	ABHD10	NM_018394	3q13.13
210006_at	15	ABHD14A	BC002571	3p21.1

205566_at	13	ABHD2	NM_007011	15q26.1
213017_at	14	ABHD3	AL534702	18q11.2
218581_at	14	ABHD4	NM_022060	14q11.2
45288_at	12	ABHD6	AA209239	3p21.2
209027_s_at	15	ABI1	BF673013	10p11.2
207268_x_at	14	ABI2	NM_005759	2q33
202123_s_at	15	ABL1	NM_005157	9q34.1
200965_s_at	15	ABLIM1	NM_006720	10q25
210461_s_at	15	ABLIM1	BC002448	10q25
205730_s_at	15	ABLIM3	NM_014945	5q33.1
208634_s_at	15	abp620	AB029290	1p32-p31
212895_s_at	15	ABR	AL527773	17p13.3
202025_x_at	15	ACAA1	NM_001607	3p23-p22
214274_s_at	15	ACAA1	AI860341	3p23-p22
202003_s_at	15	ACAA2	NM_006111	18q21.1
43427_at	15	ACACB	AI970898	12q24.1
49452_at	15	ACACB	AI057637	12q24.1
219986_s_at	13	ACAD10	NM_025247	12q24.2
221669_s_at	15	ACAD8	BC001964	11q25
206068_s_at	12	ACADL	NM_001608	2q34-q35
202502_at	15	ACADM	NM_000016	1p31
202366_at	15	ACADS	NM_000017	12q22-qter
200710_at	15	ACADVL	NM_000018	17p13-p11
205412_at	15	ACAT1	NM_000019	11q22.3-q23.1
209608_s_at	15	ACAT2	BC000408	6q25.3-q26
222010_at	15	ACAT2	BF224073	6q25.3-q26
222011_s_at	15	ACAT2	BF224073	6q25.3-q26
221641_s_at	15	ACATE2	AF241787	Xp22.13
202323_s_at	11	ACBD3	AI636775	1q42.13
202324_s_at	15	ACBD3	NM_022735	1q42.13
209389_x_at	15	ACBP	M15887	2q12-q21
211070_x_at	15	ACBP	BC006466	2q12-q21
209874_x_at	12	ACDP2	AK023066	10q24.33
219962_at	15	ACE2	NM_021804	Xp22
222257_s_at	15	ACEH; DKFZP434A014	AK026461	Xp22
214894_x_at	15	ACF7	AK023285	1p32-p31
215222_x_at	15	ACF7	AK023406	1p32-p31
209320_at	15	AC-III	AF033861	2p24-p22
209321_s_at	15	AC-III	AF033861	2p24-p22
201715_s_at	15	ACIN1	NM_014977	14q11.2
201127_s_at	13	ACLY	NM_001096	17q12-q21
201128_s_at	15	ACLY	NM_001096	17q12-q21
218981_at	13	ACN9	NM_020186	7q22.1
207071_s_at	15	ACO1	NM_002197	9p22-q32
200793_s_at	15	ACO2	NM_001098	22q11.2-q13.31
202982_s_at	15	ACOT2	NM_006821	14q24.3
208002_s_at	15	ACOT7	NM_007274	1p36.31-p36.11
204212_at	14	ACOT8	NM_005469	20q12-q13.1
204241_at	12	ACOX3	BF055171	4p15.3
201629_s_at	10	ACP1	NM_004300	2p25

201630_s_at	15	ACP1	NM_004300	2p25
215227_x_at	15	ACP1	BG035989	2p25
202767_at	15	ACP2	NM_001610	11p11.2-p11.11
218795_at	13	ACP6	NM_016361	1q21
207969_x_at	14	ACRV1	NM_020109	11p12-q13
203872_at	15	ACTA1	NM_001100	1q42.13-q42.2
200974_at	15	ACTA2	NM_001613	10q23.3
200801_x_at	15	ACTB	NM_001101	7p15-p12
205132_at	15	ACTC	NM_005159	15q11-q14
201550_x_at	15	ACTG1	NM_001614	17q25
211970_x_at	15	ACTG1	BG026805	17q25
211983_x_at	15	ACTG1	BE741683	17q25
211995_x_at	15	ACTG1	AL567820	17q25
212363_x_at	15	ACTG1	AU145192	17q25
212988_x_at	15	ACTG1	AL515810	17q25
213214_x_at	15	ACTG1	AW190090	17q25
221607_x_at	15	ACTG1	BC001920	17q25
202274_at	13	ACTG2	NM_001615	2p13.1
202666_s_at	14	ACTL6A	NM_004301	3q27.1
208636_at	15	ACTN1	AI082078	14q24.1-q24.2
208637_x_at	15	ACTN1	BC003576	14q24.1-q24.2
211160_x_at	12	ACTN1	M95178	14q24.1-q24.2
203861_s_at	15	ACTN2	AU146889	1q42-q43
203862_s_at	15	ACTN2	H16245	1q42-q43
203863_at	15	ACTN2	NM_001103	1q42-q43
203864_s_at	15	ACTN2	NM_001103	1q42-q43
222230_s_at	15	ACTR11; HARP11	AK022248	14q22.3
200720_s_at	15	ACTR1A	AL532341	10q24.33
200721_s_at	15	ACTR1A	NM_005736	10q24.33
202135_s_at	15	ACTR1B	NM_005735	2q11.1-q11.2
200727_s_at	10	ACTR2	AA699583	2p14
200728_at	15	ACTR2	BE566290	2p14
200729_s_at	15	ACTR2	BE566290	2p14
200996_at	15	ACTR3	NM_005721	2q14.1
213101_s_at	15	ACTR3	Z78330	2q14.1
213102_at	15	ACTR3	Z78330	2q14.1
219623_at	15	ACTR5	NM_024855	20q12
218395_at	15	ACTR6	NM_022496	12q23.3
218658_s_at	10	ACTR8	BF967592	---
213198_at	15	ACVR1B	AL117643	12q13
205327_s_at	13	ACVR2A	NM_001616	2q22.2-q23.3
220028_at	11	ACVR2B	NM_001106	3p22
202740_at	15	ACY1	NM_000666	3p21.1
205260_s_at	15	ACYP1	NM_001107	14q24.3
206833_s_at	15	ACYP2	NM_001108	2p16.2
209891_at	14	AD024	AF225416	2q31.1
215273_s_at	11	ADA3	AK024982	3p25.3
202603_at	15	ADAM10	N51370	15q2
202604_x_at	14	ADAM10	NM_001110	15q2
214895_s_at	13	ADAM10	AU135154	15q2

205745_x_at	14	ADAM17	NM_003183	2p25
213532_at	15	ADAM17	AI797833	2p25.2
209765_at	15	ADAM19	AF311317	5q32-q33
207665_at	13	ADAM21	NM_003813	14q24.1
206046_at	15	ADAM23	NM_003812	2q33
213808_at	15	ADAM23	BE674466	---
202381_at	15	ADAM9	NM_003816	8p11.22
222162_s_at	15	ADAMTS1	AK023795	21q21.2
214913_at	14	ADAMTS3	AB002364	4q21.1
219935_at	15	ADAMTS5	NM_007038	21q21.3
220676_at	13	ADAMTS8	NM_007037	11q25
220287_at	14	ADAMTS9	NM_020249	3p14.3-p14.2
201786_s_at	15	ADAR	NM_001111	1q21.1-q21.2
203865_s_at	11	ADARB1	NM_015833	21q22.3
44120_at	14	ADCK2	AI879381	7q32-q34
213245_at	12	ADCY1	AL120173	7p13-p12
209195_s_at	15	ADCY6	AF250226	12q12-q13
204497_at	15	ADCY9	AB011092	16p13.3
208030_s_at	15	ADD1	NM_001119	4p16.3 /// 4p16.3
214726_x_at	14	ADD1	AL556041	4p16.3
214736_s_at	15	ADD1	BE898639	4p16.3
201034_at	15	ADD3	BE545756	10q24.2-q24.3
201752_s_at	15	ADD3	AI763123	10q24.2-q24.3
201753_s_at	15	ADD3	NM_019903	10q24.2-q24.3
205882_x_at	15	ADD3	NM_016824	10q24.2-q24.3
209122_at	15	ADFP	BC005127	9p22.1
208847_s_at	15	ADH5	M29872	4q21-q25
208848_at	15	ADH5	M30471	4q21-q25
217748_at	15	ADIPOR1	NM_015999	1p36.13-q41
201346_at	15	ADIPOR2	NM_024551	12p13.31
204119_s_at	15	ADK	NM_001123	10q22
204120_s_at	12	ADK	NM_001123	10q22
202912_at	15	ADM	NM_001124	11p15.4
201773_at	15	ADNP	NM_015339	20q13.13
222366_at	13	ADNP	W86781	---
205481_at	15	ADORA1	NM_000674	1q32.1
205013_s_at	15	ADORA2A	NM_000675	22q11.23
205891_at	12	ADORA2B	NM_000676	17p12-p11.2
220980_s_at	13	ADPGK	NM_031284	15q22.33
214086_s_at	15	ADPRT2	AK001980	14q11.2-q12
206170_at	13	ADRB2	NM_000024	5q31-q32
201281_at	15	ADRM1	NM_007002	20q13.33
202144_s_at	15	ADSL	NM_000026	22q13.1
210250_x_at	15	ADSL	AF067854	22q13.1
221761_at	15	ADSS	AA628948	1cen-q12
201792_at	14	AEBP1	NM_001129	7p13
221935_s_at	14	AER61	AK023140	3p14.2
217729_s_at	15	AES	NM_001130	19p13.3
211071_s_at	15	AF1Q; RP11-316M1.10	BC006471	1q21 /// 1q21
203563_at	12	AFAP	NM_021638	4p16

201924_at	15	AFF1	NM_005935	4q21
202486_at	15	AFG3L2	NM_006796	18p11
217939_s_at	15	AFTIPHILIN	NM_017657	2p15
214693_x_at	11	AG1	BE732345	1q21.2
215434_x_at	11	AG1	AV684285	---
204333_s_at	12	AGA	NM_000027	4q32-q33
216064_s_at	12	AGA	W27131	4q32-q33
216733_s_at	15	AGAT	X86401	15q15.1
218534_s_at	15	AGGF1	NM_018046	5q14.1
222132_s_at	15	AGK; FLJ10842	AJ278150	7q34
203566_s_at	15	AGL	NM_000645	1p21
205608_s_at	14	AGP1	U83508	8q22.3-q23
218096_at	15	AGPAT5	NM_018361	8p23.1
205401_at	14	AGPS	NM_003659	2q31
212285_s_at	15	AGRN	AI424797	1p36.33
216268_s_at	15	AGS	U77914	20p12.1-p11.23
202834_at	15	AGT	NM_000029	1q42-q43
204500_s_at	15	AGTPBP1	NM_015239	9q22.1
205357_s_at	15	AGTR1	NM_000685	3q21-q25
207294_at	14	AGTR2	NM_000686	Xq22-q23
222321_at	14	AGTR2	AI911273	Xq22-q23
200903_s_at	15	AHCY	NM_000687	20cen-q13.1
200848_at	15	AHCYL1	AA479488	1p12
200849_s_at	15	AHCYL1	AI589266	1p12
200850_s_at	15	AHCYL1	NM_006621	1p12
221569_at	15	AHI1	AL136797	6q23.2
202820_at	13	AHR	NM_001621	7p15
201491_at	15	AHSA1	NM_012111	14q23.3-31
209464_at	14	aik2	AB011446	17p13.1
212543_at	14	AIM1	U83115	6q21
201781_s_at	15	AIP	AL558532	11q13.3
201782_s_at	15	AIP	NM_003977	11q13.3
209737_at	15	AIP1	AB014605	7q21
217097_s_at	13	AIP1	AC004990	---
200611_s_at	15	AIP1; NORI-1	AB010427	4p16.1
210935_s_at	11	AIP1; NORI-1	AF274954	4p16.1
209493_at	15	AIPC	AF338650	5p13.3
219977_at	12	AIPL1	NM_014336	17p13.1
210005_at	12	AIRS	D32051	21q22.1
202587_s_at	15	AK1	BC001116	9q34.1
208967_s_at	15	AK2	U39945	1p34
212174_at	14	AK2	W02312	1p34
212175_s_at	15	AK2	AW277253	1p34
204347_at	14	AK3	AI653169	9pter-p13
204348_s_at	15	AK3L1	NM_013410	9pter-p13
210625_s_at	15	AKAP	U34074	17q21-q23
201674_s_at	15	AKAP1	BC000729	17q21-q23
201675_at	15	AKAP1	NM_003488	17q21-q23
213396_s_at	15	AKAP10	AA456929	17p11.1
203156_at	15	AKAP11	NM_016248	13q13.3

208325_s_at	11	AKAP13	NM_006738	15q24-q25
209534_x_at	15	AKAP13	BF222823	15q24-q25
221718_s_at	15	AKAP13	M90360	15q24-q25
211172_x_at	14	AKAP18	AF161075	6q23
210517_s_at	15	AKAP250	AB003476	6q24-q25
205359_at	15	AKAP6	NM_004274	14q13.1
205771_s_at	13	AKAP7	AL137063	6q23
218064_s_at	14	AKAP8L	NM_014371	19p13.13-p13.12
210962_s_at	15	AKAP9	AB019691	7q21-q22
201900_s_at	15	AKR1A1	NM_006066	1p33-p32
201272_at	15	AKR1B1	NM_001628	7q35
204151_x_at	15	AKR1C1	NM_001353	10p15-p14
202139_at	15	AKR7A2	NM_003689	1p35.1-p36.23
214259_s_at	15	AKR7A2	AI144075	1p35.1-p36.23
216381_x_at	12	AKR7A2	AL035413	---
207163_s_at	15	AKT1	NM_005163	14q32.32
212607_at	15	AKT3	N32526	1q43-q44
212609_s_at	14	AKT3	U79271	1q43-q44
218487_at	10	ALAD	BC000977	9q34
205633_s_at	15	ALAS1	NM_000688	3p21.1
211560_s_at	12	ALAS2	AF130113	Xp11.21
201951_at	15	ALCAM	BF242905	3q13.1
201952_at	15	ALCAM	AA156721	3q13.1
214687_x_at	15	ALDA	AK026577	16q22-q24
212224_at	13	ALDH1A1	NM_000689	9q21.13
201425_at	15	ALDH2	NM_000690	12q24.2
202053_s_at	15	ALDH3A2	NM_000382	17p11.2
202054_s_at	14	ALDH3A2	NM_000382	17p11.2
210544_s_at	13	ALDH3A2	BC002430	17p11.2
211004_s_at	13	ALDH3B1	BC002553	11q13
203722_at	13	ALDH4A1	NM_003748	1p36
204290_s_at	15	ALDH6A1	NM_005589	14q24.3
221588_x_at	15	ALDH6A1	AW612403	14q24.3
221589_s_at	15	ALDH6A1	AW612403	14q24.2
221590_s_at	15	ALDH6A1	AW612403	14q24.2
208950_s_at	15	ALDH7A1	BC002515	5q31
208951_at	15	ALDH7A1	BC002515	5q31
201612_at	15	ALDH9A1	NM_000696	1q23.1
200966_x_at	15	ALDOA	NM_000034	16q22-q24
202022_at	15	ALDOC	NM_005165	17cen-q12
213413_at	15	ALF	BG434174	2p16.3
215306_at	11	ALF	AL049443	---
210169_at	15	ALG1	AB007880	16p13.3
218203_at	15	ALG5	NM_013338	13q13.2
219649_at	15	ALG6	NM_013339	1p31.3
203545_at	15	ALG8	NM_024079	11pter-p15.5
219374_s_at	14	ALG9	NM_024740	11q23
203935_at	15	ALK2	L02911	2q23-q24
205621_at	15	ALKBH	NM_006020	14q24
214220_s_at	15	ALMS1	AW003635	2p13

214707_x_at	15	ALMS1	AB002326	2p13
204446_s_at	13	ALOX5	NM_000698	10q11.2
209621_s_at	15	ALP	AF002280	4q35
216377_x_at	14	ALPG	X07247	---
204659_s_at	12	ALR	AF124604	16p13.3-p13.12
219834_at	11	ALS2CR8	NM_024744	2q33.3
201964_at	12	ALS4	N64643	9q34.3
202550_s_at	15	ALS8	AF160212	20q13
209424_s_at	11	AMACR	AI796120	5p13.2-q11.1
209425_at	14	AMACR	AA888589	5p13.2-q11.1
201196_s_at	14	AMD2	M21154	6q21-q22
201197_at	15	AMD2	M21154	6q21-q22
222108_at	14	AMIGO2	AC004010	12q13.11
204976_s_at	13	AMMERC1	AK023637	Xq22.3
203002_at	15	AMOTL2	NM_016201	3q21-q22
207755_at	13	AMOTL2	NM_025017	---
212360_at	15	AMPD2	AI916249	1p13.3
205257_s_at	15	AMPH	NM_001635	7p14-p13
210202_s_at	13	AMPH2	U87558	2q14
214439_x_at	14	AMPH2	AF043899	2q14
204294_at	15	AMT	NM_000481	3p21.2-p21.1
203360_s_at	10	AMY-1	D50692	1p33-p32.2
208498_s_at	15	AMY1A	NM_004038	1p21 /// 1p21
218167_at	15	AMZ2	NM_016627	17q24.3
215425_at	14	ANA	AL049332	21q21.1-q21.2
207845_s_at	15	ANAPC10	NM_014885	4q31
209001_s_at	15	ANAPC13	BC005398	3q22.1
200098_s_at	15	ANAPC5	T33068	12q24.31
208721_s_at	14	ANAPC5	BF967271	12q24.31
208722_s_at	15	ANAPC5	BC001081	12q24.31
211036_x_at	15	ANAPC5	BC006301	12q24.31
209957_s_at	15	ANF	M30262	1p36.21
205609_at	15	ANGPT1	NM_001146	8q22.3-q23
205572_at	10	ANGPT2	NM_001147	8p23.1
213001_at	15	ANGPTL2	AF007150	9q34
213004_at	15	ANGPTL2	AI074333	9q34
219514_at	11	ANGPTL2	NM_012098	9q34
208353_x_at	14	ANK1	NM_020480	8p11.1
202920_at	15	ANK2	NM_001148	4q25-q27
202921_s_at	15	ANK2	NM_001148	4q25-q27
206385_s_at	15	ANK3	NM_020987	10q21
209442_x_at	15	ANK3	AL136710	10q21
208772_at	11	ANKHD1	AU160676	5q31.3
214919_s_at	15	ANKHD1	R39094	---
219081_at	15	ANKHD1	NM_024668	5q31.3
218769_s_at	15	ANKRA2	NM_023039	5q12-q13
206029_at	14	ANKRD1	NM_014391	10q23.33
218093_s_at	15	ANKRD10	NM_017664	13q34
212286_at	12	ANKRD12	AW572909	18p11.22
212289_at	13	ANKRD12	AW572909	18p11.22

216563_at	10	ANKRD12	X80821	18p11.22
213005_s_at	15	ANKRD15	D79994	9p24.3
212211_at	15	ANKRD17	AI986295	4q21.1-q21.21
218418_s_at	15	ANKRD25	NM_015493	19p13.2
213035_at	13	ANKRD28	AI081194	3p25.1
211717_at	15	ANKRD40	BC005853	17q21.33
219069_at	14	ANKRD49	NM_017704	11q21
204671_s_at	12	ANKRD6	BE677131	6q14.2-q16.1
204672_s_at	11	ANKRD6	NM_014942	6q14.2-q16.1
212747_at	14	ANKS1	AI990523	6p21.31
201038_s_at	14	ANP32A	T67821	15q22.3-q23
201043_s_at	13	ANP32A	NM_006305	15q22.3-q23
201051_at	15	ANP32A	BE560202	15q22.3-q23
201305_x_at	15	ANP32B	NM_006401	9q22.32
201306_s_at	15	ANP32B	NM_006401	9q22.32
208103_s_at	15	ANP32E	NM_030920	1q21.3 /// 1q21.3
221505_at	15	ANP32E	AW612574	1q21.3
32625_at	13	ANPa	X15357	1q21-q22
209369_at	15	ANX3	M63310	4q13-q22
201012_at	15	ANXA1	NM_000700	9q12-q21.2
206200_s_at	15	ANXA11	NM_001157	10q23
214783_s_at	11	ANXA11	BG177920	---
201590_x_at	15	ANXA2	NM_004039	15q21-q22
210427_x_at	15	ANXA2	BC001388	15q21-q22
213503_x_at	15	ANXA2	BE908217	15q21-q22
201301_s_at	15	ANXA4	NM_001153	2p13
201302_at	15	ANXA4	NM_001153	2p13
200782_at	15	ANXA5	NM_001154	4q26-q28
200982_s_at	15	ANXA6	NM_001155	5q32-q34
201366_at	15	ANXA7	NM_004034	10q21.1-q21.2
203074_at	15	ANXA8	NM_001630	10q11.2
208614_s_at	15	AOI	M62994	3p14.3
209766_at	13	AOP1	AF118073	10q25-q26
209600_s_at	14	AOX	S69189	17q24-q25
201464_x_at	15	AP1	BC002646	1p32-p31
201466_s_at	15	AP1	BC002646	1p32-p31
64418_at	14	AP1GBP1	AI472320	17q21.1
209635_at	15	AP1S1	BC003561	7q22.1
203300_x_at	15	AP1S2	NM_003916	Xp22.31
211779_x_at	15	AP2A2	BC006155	11p15.5 /// 11p15.5
212159_x_at	15	AP2A2	AI125280	11p15.5
215764_x_at	14	AP2A2	AA877641	11p15.5
200612_s_at	15	AP2B1	NM_001282	17q11.2-q12
200615_s_at	15	AP2B1	AL567295	17q11.2-q12
200613_at	15	AP2M1	NM_004068	3q28
202120_x_at	15	AP2S1	NM_004069	19q13.2-q13.3
208074_s_at	15	AP2S1	NM_021575	19q13.2-q13.3
211047_x_at	15	AP2S1	BC006337	19q13.2-q13.3
203142_s_at	15	AP3B1	NM_003664	5q14.1
206592_s_at	15	AP3D1	NM_003938	19p13.3

203410_at	15	AP3M2	NM_006803	8p11.2
202442_at	15	AP3S1	NM_001284	5q22
202398_at	12	AP3S2	BC002785	15q26.1
202399_s_at	15	AP3S2	NM_005829	15q26.1
210277_at	15	AP47B	AF155159	14q12
210278_s_at	13	AP47B	AF155159	14q12
204844_at	15	APA; gp160	L12468	4q25
209944_at	15	APA1; APA-1	BC000330	14q24.3
204859_s_at	14	APAF1	NM_013229	12q23
215148_s_at	15	APBA3	AI141541	19p13.3
202652_at	15	APBB1	NM_001164	11p15
212970_at	13	APBB2	AI694303	4p14
212985_at	14	APBB2	BF115739	4p14
204650_s_at	15	APBB3	NM_006051	5q31
203525_s_at	14	APC	AI375486	5q21-q22
203526_s_at	14	APC	M74088	5q21-q22
210027_s_at	15	APE	M80261	14q11.2-q12
205265_s_at	15	APEG1	NM_005876	2q36.1
201284_s_at	11	APEH	NM_001640	3p21.31
204408_at	11	APEX2	NM_014481	Xp11.22
213026_at	15	APG12L	BE965998	5q21-q22
213930_at	15	APG12L	N51708	5q21-q22
208815_x_at	15	apg-2	AB023420	5q31.1-q31.2
221492_s_at	15	APG3	AF202092	3q13.2
213115_at	15	APG4A	AL031177	Xq22.1-q22.3
204903_x_at	15	APG4B	AL080168	2q37.3
212280_x_at	15	APG4B	AA532726	2q37.3
210639_s_at	15	APG5L	AF293841	6q21
218389_s_at	15	APH1A	NM_016022	1p36.13-q31.3
208759_at	14	APH2	AF240468	1q22-q23
206536_s_at	13	API3	U32974	Xq25
201686_x_at	12	API5	AF229254	11p12-q12
201687_s_at	15	API5	NM_006595	11p12-q12
218698_at	15	APIP	NM_015957	11p13
32042_at	15	APK1 antigen	S72904	Xq25-q26.2
208248_x_at	15	APLP2	NM_001642	11q23-q25
208702_x_at	15	APLP2	BC000373	11q23-q25
208703_s_at	15	APLP2	BG427393	11q23-q25
208704_x_at	15	APLP2	BC000373	11q23-q25
211404_s_at	15	APLP2	BC004371	11q23-q25
214875_x_at	15	APLP2	AW001847	11q23-q25
203571_s_at	10	APM2	BC004471	10q23.31
204450_x_at	13	APOA1	NM_000039	11q23-q24
206160_at	15	APOBEC2	NM_006789	6p21
206632_s_at	10	APOBEC3B	NM_004900	22q13.1-q13.2
204205_at	15	APOBEC3G	NM_021822	22q13.1-q13.2
206738_at	11	APOC4	NM_001646	19q13.2
201525_at	15	APOD	NM_001647	3q26.2-qter
203381_s_at	15	APOE	NM_000041	19q13.2
203382_s_at	15	APOE	NM_000041	19q13.2

212884_x_at	15	APOE	AI358867	19q13.2
221087_s_at	15	APOL3	NM_014349	22q13.1
205682_x_at	12	APOM	NM_019101	6p21.31
200602_at	15	APP	NM_000484	21q21.2
211277_x_at	15	APP	BC004369	21q21.2
214953_s_at	15	APP	X06989	21q21.2
202268_s_at	15	APPBP1	NM_003905	16q22
202629_at	15	APPBP2	AV681579	17q21-q23
202630_at	14	APPBP2	AA046411	17q21-q23
202631_s_at	14	APPBP2	NM_006380	17q21-q23
209788_s_at	10	APPILS	AF183569	5q15
218158_s_at	13	APPL	NM_012096	3p21.1-p14.3
209500_x_at	13	APRIL	AF114012	17p13.1
207956_x_at	15	APRIN	NM_015928	13q12.3
215888_at	14	APRIN	AK026889	13q12.3
203219_s_at	15	APRT	NM_000485	16q24
213892_s_at	15	APRT	AA927724	16q24
205367_at	14	APS	NM_020979	7q22
216252_x_at	11	APT1	Z70519	10q24.1
215566_x_at	14	APT-2; DJ886K2.4	AK024724	1p36.12-p35.1
218527_at	15	APTX	NM_017692	9p13.3
207542_s_at	15	AQP1	NM_000385	7p14
209047_at	15	AQP1	AL518391	7p14
39248_at	15	AQP3	N74607	9p13
212584_at	15	AQR	BG260519	15q13.3
35846_at	15	AR7	M24899	17q11.2
208736_at	15	ARC21; p21-Arc	AF004561	12q24.11
208679_s_at	15	ARC34	AF279893	2q36.1
201176_s_at	15	ARCN1	NM_001655	11q23.3
218185_s_at	15	Arcp; FLJ10511	AK024053	8q12.3
210995_s_at	10	ARD1; ARFD1; RNF46	AF230399	5q12.3
203025_at	15	ARD1A	NM_003491	Xq28
200065_s_at	15	ARF1	AF052179	1q42
208750_s_at	15	ARF1	AF052179	1q42
200011_s_at	15	ARF3	NM_001659	12q13
200734_s_at	15	ARF3	BG341906	12q13
201096_s_at	15	ARF4	AL537042	3p21.2-p21.1
201097_s_at	15	ARF4	NM_001660	3p21.2-p21.1
203586_s_at	15	ARF4L	U25771	17q12-q21
201526_at	15	ARF5	NM_001662	7q31.3
203312_x_at	15	ARF6	NM_001663	7q22.1
214182_at	14	ARF6	AA243143	7q22.1
217888_s_at	15	ARFGAP1	AL039370	20q13.33
202211_at	15	ARFGAP3	BC005122	22q13.2-q13.3
202956_at	15	ARFGEF1	NM_006421	8q13
218230_at	15	ARFIP1	AL044651	4q31.3
202109_at	10	ARFIP2	NM_012402	11p15
203174_s_at	14	ARFRP1	NM_003224	20q13.3
215984_s_at	14	ARFRP1	AL121845	20q13.3
206177_s_at	11	ARG1	NM_000045	6q23

203945_at	14	ARG2	NM_001172	14q24.1-q24.3
203946_s_at	15	ARG2	NM_001172	14q24.1-q24.3
57082_at	15	ARH	AA169780	1p36-p35
212724_at	15	ARHE	BG054844	2q23.3
202117_at	15	ARHGAP1	BG468434	11p12-q12
219431_at	10	ARHGAP10	NM_024605	4q31.22
207606_s_at	15	ARHGAP12	NM_018287	---
218076_s_at	15	ARHGAP17	NM_018054	16p12.2
37577_at	14	ARHGAP19	U79256	10q24.2
221030_s_at	10	ARHGAP24	NM_031305	4q22.1 /// 4q22.1
205068_s_at	15	ARHGAP26	BE671084	5q31
203910_at	15	ARHGAP29	NM_004815	1p22.1
217936_at	15	ARHGAP5	AW044631	14q12
206167_s_at	14	ARHGAP6	NM_001174	Xp22.3
47069_at	15	ARHGAP8	AA533284	22q13.31
211716_x_at	15	ARHGDIA	BC005851	17q25.3
201288_at	15	ARHGDIB	NM_001175	12p12.3
213867_x_at	15	ARHGEF10	AA809056	---
201334_s_at	15	ARHGEF12	AB002380	11q23.3
205507_at	14	ARHGEF15	NM_014958	17p13.1
203756_at	11	ARHGEF17	NM_014786	11q13.3
207629_s_at	12	ARHGEF2	NM_004723	1q21-q22
209435_s_at	15	ARHGEF2	BC000265	1q21-q22
218501_at	13	ARHGEF3	NM_019555	3p21-p13
202547_s_at	15	ARHGEF7	NM_003899	13q34
202548_s_at	15	ARHGEF7	NM_003899	13q34
203264_s_at	15	ARHGEF9	NM_015185	Xq11.2
215506_s_at	14	ARHI; NOEY2	AK021882	1p31
221789_x_at	14	ARHT2	AK024450	16p13.3
222131_x_at	13	ARHT2	BC004327	16p13.3
212152_x_at	15	ARID1A	AI679080	1p35.3
218917_s_at	15	ARID1A	NM_018450	1p35.3
205062_x_at	15	ARID4A	NM_002892	14q22.3-q23.1
214139_at	10	ARID4B	AI051476	1q42.1-q43
221230_s_at	15	ARID4B	NM_016374	1q42.1-q43
212614_at	15	ARID5B	BG285011	10q21.3
201878_at	15	ARIH1	NM_005744	---
201879_at	15	ARIH1	AI694332	15q24
201880_at	15	ARIH1	AL040708	15q24
201881_s_at	15	ARIH1	NM_005744	15q24
201228_s_at	12	ARIH2	NM_006321	3p21.2-p21.3
201229_s_at	15	ARIH2	BC000422	3p21.2-p21.3
201230_s_at	15	ARIH2	NM_006321	3p21.2-p21.3
201657_at	15	ARL1	BE890745	12q23.3
201658_at	15	ARL1	AU151560	12q23.3
201659_s_at	15	ARL1	NM_001177	12q23.3
202564_x_at	15	ARL2	NM_001667	11q12
202092_s_at	14	ARL2BP	NM_012106	16q13
202641_at	15	ARL3	NM_004311	10q23.3
205020_s_at	13	ARL4A	NM_005738	7p21-p15.3

218150_at	15	ARL5A	NM_012097	2q24.1
211935_at	15	ARL6IP	D31885	16p12-p11.2
218216_x_at	15	ARL6IP4	NM_016638	12q24.31
220597_s_at	15	ARL6IP4	NM_018694	12q24.31
200760_s_at	15	ARL6IP5	N92494	3p14
200761_s_at	15	ARL6IP5	NM_006407	3p14
202207_at	12	ARL7	BG435404	2q37.2
217852_s_at	15	ARL8B	NM_018184	3p26.1
217755_at	15	ARM2; HN1A	AF060925	17q25.2
203486_s_at	15	ARMC8	NM_015396	3q22.3
203487_s_at	15	ARMC8	NM_015396	3q22.3
218694_at	15	ARMCX1	NM_016608	Xq21.33-q22.2
203404_at	15	ARMCX2	NM_014782	Xq21.33-q22.2
217858_s_at	15	ARMCX3	NM_016607	Xq21.33-q22.2
219335_at	15	ARMCX5	NM_022838	Xq22.1-q22.3
202655_at	15	ARMET	NM_006010	3p21.1
218221_at	14	ARNT	NM_001668	1q21
209121_x_at	14	ARP-1	M64497	15q26
200950_at	15	ARPC1A	NM_006409	7q22.1
201954_at	15	ARPC1B	NM_005720	7q22.1
207988_s_at	15	ARPC2	NM_005731	2q36.1
213513_x_at	15	ARPC2	BG034239	2q36.1
211963_s_at	15	ARPC5	AL516350	1q25.2
220966_x_at	15	ARPC5L	NM_030978	9q34.11
214553_s_at	15	ARPP-19	NM_006628	15q21.1
221482_s_at	15	ARPP-19	AF084555	15q21.1
221483_s_at	15	ARPP-19	AF084555	15q21.1
201680_x_at	15	ARS2	NM_015908	7q21
222047_s_at	15	ARS2	AI523895	7q21
217775_s_at	14	ARSDR1	AF167438	14q24.1
217776_at	15	ARSDR1	AF167438	14q24.1
201837_s_at	15	ART1	AF197954	2pter-p25.1
210147_at	15	ART3	U47054	4p15.1-p14
210385_s_at	11	ARTS-1	AF106037	5q15
210980_s_at	15	ASAH1	U47674	8p22-p21.3
213702_x_at	15	ASAH1	AI934569	8p22-p21.3
213902_at	15	ASAH1	AI379338	8p22-p21.3
215178_x_at	13	ASAHL	AV724215	---
212818_s_at	13	ASB-1	AF055024	2q37
212819_at	11	ASB-1	AF055024	2q37
218841_at	15	ASB8	NM_024095	12q13.12
205673_s_at	14	ASB9	NM_024087	---
215684_s_at	13	ASC1p100	AL096741	22q12.1
219336_s_at	15	ASCC1	NM_015947	10pter-q25.3
212815_at	15	ASCC3	AA156961	6q16
200058_s_at	15	ASCC3L1	BC001417	2q11.2
201855_s_at	14	ASCIZ	NM_015251	16q23.2
214468_at	15	ASD3	D00943	14q12
201742_x_at	15	ASF; SF2; SRp30a	M69040	17q21.3-q22
203427_at	15	ASF1A	NM_014034	6q22.31

218115_at	12	ASF1B	NM_018154	19p13.13
215075_s_at	15	ASH	L29511	17q24-q25
218554_s_at	13	ASH1L	NM_018489	1q22
209517_s_at	15	ASH2L	AB020982	8p11.2
210094_s_at	10	ASIP	AF196186	10p11.22
221526_x_at	12	ASIP	AF196185	10p11.22
221527_s_at	14	ASIP	AF196185	10p11.22
203836_s_at	10	ASK1	D84476	6q22.33
204608_at	14	ASL	NM_000048	7cen-q11.2
209394_at	13	ASMTL	BC002508	Xp22.3
36553_at	15	ASMTL	AA669799	Xp22.3
36554_at	15	ASMTL	Y15521	Xp22.3
202024_at	15	ASNA1	NM_004317	19q13.3
205047_s_at	14	ASNS	NM_001673	7q21.3
202511_s_at	15	ASP; APG5; APG5L; hAPG5	AK001899	6q21
202512_s_at	14	ASP; APG5; APG5L; hAPG5	AK001899	6q21
207284_s_at	14	ASPH	NM_020164	8q12.1
219918_s_at	14	ASPM	NM_018123	1q31
219087_at	15	ASPN	NM_017680	9q22
207076_s_at	15	ASS	NM_000050	9q34.1
213528_at	15	AsTP2	AL035369	1q23.3
212234_at	11	ASXL1	AL034550	20q11.1
212237_at	15	ASXL1	N64780	20q11.1
212238_at	15	ASXL1	AL117518	20q11.1
218659_at	15	ASXL2	NM_018263	2p24.1
211509_s_at	15	ASY	AB015639	2p14-p13
210968_s_at	15	ASY	AF333336	2p14-p13
214629_x_at	15	ASY	AF320999	2p14-p13
218782_s_at	13	ATAD2	NM_014109	8q24.13
222103_at	15	ATF1	AI434345	12q13
205446_s_at	14	ATF2	NM_001880	2q32
212984_at	15	ATF2	BE786164	2q32
202672_s_at	15	ATF3	NM_001674	1q32.3
200779_at	15	ATF4	NM_001675	22q13.1
204998_s_at	15	ATF5	BC005174	19q13.3
218987_at	15	ATF7IP	NM_018179	12p13.2
219870_at	15	ATF7IP2	NM_024997	16p13.2
204833_at	13	ATG12	NM_004707	5q21-q22
214699_x_at	11	Atg21	AK024279	7p22.2
202492_at	15	ATG9A	NM_024085	2q36.1
210858_x_at	15	ATM	U26455	11q22-q23
212672_at	15	ATM	U82828	11q22-q23
203454_s_at	15	ATOX1	NM_004045	5q32
213238_at	15	ATP10D	AI478147	4p12
213582_at	11	ATP11A	BF439472	13q34
212536_at	15	ATP11B	AB023173	3q27
218052_s_at	14	ATP13A1	NM_020410	19p13.11
212297_at	15	ATP13A3	BF218804	3q29
220948_s_at	15	ATP1A1	NM_000701	1p13
203296_s_at	15	ATP1A2	NM_000702	1q21-q23

201242_s_at	15	ATP1B1	BC000006	1q24
201243_s_at	15	ATP1B1	NM_001677	1q24
204311_at	12	ATP1B2	NM_001678	17p13.1
212361_s_at	15	ATP2A2	AA805753	12q23-q24.1
212362_at	15	ATP2A2	AA805753	12q23-q24.1
209281_s_at	15	ATP2B1	L14561	12q21-q23
212930_at	12	ATP2B1	AW576457	12q21-q23
215716_s_at	14	ATP2B1	L14561	12q21-q23
212135_s_at	15	ATP2B4	AW517686	1q25-q32
212136_at	15	ATP2B4	AW517686	1q25-q32
209934_s_at	14	ATP2C1	AF225981	3q21-q24
211137_s_at	14	ATP2C1A	AF189723	3q21-q24
213738_s_at	15	ATP5A1	AI587323	18q12-q21
201322_at	15	ATP5B	NM_001686	12p13-qter
205711_x_at	15	ATP5C1	NM_005174	10q22-q23
208870_x_at	15	ATP5C1	BC000931	10q22-q23
213366_x_at	15	ATP5C1	AV711183	10q22-q23
214132_at	15	ATP5C1	BG232034	10q22-q23
203926_x_at	15	ATP5D	NM_001687	19p13.3
213041_s_at	15	ATP5D	BE798517	19p13.3
217801_at	15	ATP5E	NM_006886	20q13.3
211755_s_at	15	ATP5F1	BC005960	1p13.2 /// 1p13.2
208972_s_at	15	ATP5G1	AL080089	17q21.33
207507_s_at	15	ATP5G3	NM_001689	2q31.2
207508_at	15	ATP5G3	NM_001689	2q31.2
207335_x_at	15	ATP5I	NM_007100	4p16.3
209492_x_at	15	ATP5I	BC003679	4p16.3
202325_s_at	15	ATP5J	NM_001685	21q21.1
202961_s_at	15	ATP5J2	NM_004889	7q22.1
208746_x_at	15	ATP5JG	AF070655	11q23
207573_x_at	15	ATP5L	NM_006476	11q23
208745_at	15	ATP5L	AA917672	11q23
210453_x_at	15	ATP5L	AL050277	11q23
200818_at	15	ATP5O	NM_001697	21q22.1-q22.2
216954_x_at	15	ATP5O	S77356	21q22.1-q22.2
206992_s_at	14	ATP5S	NM_015684	14q22.1
206993_at	15	ATP5S	NM_015684	14q22.1
213995_at	15	ATP5S	AW195882	14q22.1
207809_s_at	15	ATP6AP1	NM_001183	Xq28
201443_s_at	15	ATP6AP2	AF248966	Xq21
201444_s_at	15	ATP6AP2	NM_005765	Xq21
212383_at	15	ATP6V0A1	AL096733	17q21
200078_s_at	15	ATP6V0B	BC005876	1p32.3 /// 1p32.3
212041_at	15	ATP6V0D1	AL566172	16q22
200096_s_at	15	ATP6V0E	AV717561	5q35.2
201172_x_at	15	ATP6V0E	NM_003945	5q35.2
214149_s_at	14	ATP6V0E	AI252582	5q35.2
214150_x_at	15	ATP6V0E	AI252582	5q35.2
201971_s_at	12	ATP6V1A	NM_001690	3q13.31
201089_at	15	ATP6V1B2	NM_001693	8p22-p21

202872_at	15	ATP6V1C1	AW024925	8q22.3
202874_s_at	15	ATP6V1C1	NM_001695	8q22.3
208638_at	15	ATP6V1C2	BE910010	---
208678_at	15	ATP6V1E1	BC004443	22pter-q11.2
201527_at	15	ATP6V1F	NM_004231	7q32
208737_at	15	ATP6V1G1	BC003564	9q33.1
214762_at	15	ATP6V1G2	BF340635	6p21.3
205197_s_at	12	ATP7A	BE567813	Xq13.2-q13.3
205198_s_at	15	ATP7A	NM_000052	Xq13.2-q13.3
204624_at	15	ATP7B	NM_000053	13q14.2-q21
213106_at	15	ATP8A1	AI769688	4p14-p12
219660_s_at	11	ATP8A2	NM_016529	13q12-13
214594_x_at	14	ATP8B1	BG252666	18q21-q22
216873_s_at	11	ATP8B2	AL137537	1q22
212062_at	13	ATP9A	AB014511	20q13.11-q13.2
213057_at	11	ATPAF2	AW118608	---
208836_at	15	ATPB-3; FLJ29027	U51478	3q22-q23
218799_at	15	ATPBD1B	NM_018066	1p35.3
218461_at	15	ATPBD1C	NM_016301	12q24.13
218671_s_at	15	ATPIF1	NM_016311	---
36994_at	15	ATPL	M62762	16p13.3
210149_s_at	15	ATPQ; ATP5JD	AF061735	17q25
212517_at	15	ATRN	AL132773	20p13
208859_s_at	10	ATRX	AI650257	Xq13.1-q21.1
208861_s_at	15	ATRX	U72937	Xq13.1-q21.1
209392_at	14	ATX	L35594	8q24.1
210839_s_at	15	ATX	D45421	8q24.1
203231_s_at	14	ATXN1	AW235612	6p23
203232_s_at	15	ATXN1	NM_000332	6p23
208832_at	15	ATXN10	AW241832	22q13.31
202622_s_at	11	ATXN2	NM_002973	12q24
205415_s_at	10	ATXN3	AI888099	14q24.3-q32.2
205416_s_at	15	ATXN3	NM_004993	14q24.3-q32.2
204516_at	13	ATXN7	BG390306	3p21.1-p12
212114_at	15	ATXN7L3	BE967207	12q15
205052_at	15	AUH	NM_001698	9q22.31
220525_s_at	15	AUP1	NM_012103	2p13
218580_x_at	15	AURKAIP1	NM_017900	1p36.33
212599_at	15	AUTS2	AK025298	7q11.23
219366_at	15	AVEN	NM_020371	15q13.1
205539_at	11	AVIL	NM_006576	12q13.2
214331_at	11	AVIL	AI796813	---
218631_at	15	AVPI1	NM_021732	10q24.2
221613_s_at	15	AWP1	AL136598	15q24.3
202686_s_at	13	AXL	NM_021913	19q13.1
40472_at	15	AYTL3	AF007155	15q13.2
218043_s_at	14	AZI2	NM_022461	3p23
201772_at	15	AZIN1	NM_015878	8q22.3
211382_s_at	15	AZU-1; ECTACC	AF220152	10q26
221691_x_at	15	B23; NPM; MGC104254	AB042278	5q35 /// 5q35

201891_s_at	15	B2M	NM_004048	15q21-q22.2
216231_s_at	15	B2M	AW188940	15q21-q22.2
211379_x_at	15	B3GALT3	AB050855	3q25
211812_s_at	15	B3GALT3	AB050856	3q25
210205_at	11	B3GALT4	AB026730	6p21.3
203188_at	15	B3GNT6	NM_006876	11q13.1
209034_at	15	B4-2	AF279899	6q16.1
209413_at	12	B4GALT2	BC002431	1p34-p33
210243_s_at	15	B4GALT3	AF038661	1q21-q23
212876_at	12	B4GALT4	BF223021	3q13.3
221484_at	15	B4GALT5	AB004550	20q13.1-q13.2
221485_at	15	B4GALT5	AB004550	20q13.1-q13.2
53076_at	14	B4GALT7	AI040029	5q35.2-q35.3
217200_x_at	11	B561	U06715	---
210527_x_at	15	bA408E5.3	L11645	13q11
217904_s_at	15	BACE1	AI653425	11q23.2-q23.3
217867_x_at	15	BACE2	AF178532	21q22.3
204194_at	15	BACH1	NM_001186	21q22.11
221234_s_at	15	BACH2	NM_021813	6q15 /// 6q15
1861_at	15	BAD	U66879	11q13.1
209364_at	14	BAD	U66879	11q13.1
210649_s_at	13	BAF250a	AF231056	1p35.3
202387_at	15	BAG1	NM_004323	9p12
211475_s_at	15	BAG1	AF116273	9p12
209406_at	15	BAG-2; KIAA0576; dJ417I1.2	AF095192	6p12.3-p11.2
217911_s_at	15	BAG3	NM_004281	10q25.2-q26.2
202985_s_at	13	BAG5	NM_004873	14q32.33
209135_at	15	BAH	AF289489	8q12.1
210896_s_at	15	BAH	AF306765	8q12.1
203051_at	14	BAHD1	NM_014952	15q14
205638_at	12	BAI3	NM_001704	6q12
215372_x_at	13	BAIAP1	AU146794	---
203304_at	15	BAMBI	NM_012342	10p12.3-p11.2
219966_x_at	15	BANP	NM_017869	16q24
201419_at	12	BAP1	NM_004656	3p21.31-p21.2
205345_at	15	BARD1	NM_000465	2q34-q35
202391_at	15	BASP1	NM_006317	5p15.1-p14
200041_s_at	15	BAT1	NM_004640	6p21.3
208132_x_at	14	BAT2	NM_004638	6p21.3
212081_x_at	15	BAT2	AF129756	6p21.3
214201_x_at	12	BAT2	AA742237	6p21.3
201255_x_at	15	BAT3	NM_004639	6p21.3
210208_x_at	15	BAT3	BC003133	6p21.3
213318_s_at	15	BAT3	BG028844	6p21.3
208478_s_at	13	BAX	NM_004324	19q13.3-q13.4
211833_s_at	13	BAX	U19599	19q13.3-q13.4
217985_s_at	11	BAZ1A	AA102574	14q12-q13
217986_s_at	15	BAZ1A	AA102574	14q12-q13
208445_s_at	14	BAZ1B	NM_023005	7q11.23
201353_s_at	14	BAZ2A	AI653126	12q24.3-qter

203080_s_at	15	BAZ2B	NM_013450	2q23-q24
211703_s_at	15	BBP	AF353990	1p32.1
213882_at	14	BBP	AA012917	1p32.1
213883_s_at	15	BBP	AA012917	1p32.1
218471_s_at	15	BBS1	NM_024649	11q13.1
212744_at	14	BBS4	AI813772	15q22.3-q23
213015_at	13	BBX	BF448315	3q13.1
213016_at	15	BBX	AA573805	3q13.1
31837_at	10	BC002942	U62317	22q13.33
205084_at	14	BCAP29	NM_018844	7q22-q31
200837_at	15	BCAP31	NM_005745	Xq28
204032_at	13	BCAR3	NM_003567	1p22.1
203053_at	15	BCAS2	NM_005872	1p21-p13.3
220488_s_at	12	BCAS3	NM_017679	17q23
203576_at	15	BCAT2	NM_001190	19q13
218264_at	14	BCCIP	NM_016567	10q26.1
219798_s_at	12	BCDIN3	NM_019606	7q22.1
208682_s_at	15	BCG1	AF126181	Xp11.2
205433_at	15	BCHE	NM_000055	3q26.1-q26.2
202331_at	15	BCKDHA	NM_000709	19q13.1-q13.2
210653_s_at	11	BCKDHB	M55575	6q13-q15
202030_at	15	BCKDK	NM_005881	16p11.2
208712_at	15	bcl-1	M73554	11q13
205263_at	15	BCL10	AF082283	1p22
219497_s_at	12	BCL11A	NM_018014	2p16.1
203685_at	15	BCL2	NM_000633	18q21.33
217955_at	15	BCL2L13	NM_015367	22q11
209311_at	15	BCL2L2	D87461	14q11.2-q12
203140_at	15	BCL6	NM_001706	3q27
203795_s_at	10	BCL7A	NM_020993	---
210679_x_at	15	BCL7A	BC002629	---
202518_at	15	BCL7B	NM_001707	7q11.23
219072_at	13	BCL7C	NM_004765	16p11
201083_s_at	11	BCLAF1	AA740754	6q22-q23
201084_s_at	15	BCLAF1	NM_014739	6q22-q23
216870_x_at	15	BCMSUN	AF264787	13q14.3
219433_at	15	BCOR	NM_017745	Xp11.4
219444_at	15	BCORL1	NM_021946	Xq25-q26.1
210125_s_at	15	BCRG1	AF044773	11q13.1
209735_at	15	BCRP	AF098951	4q22
207618_s_at	15	BCS1L	NM_004328	2q33
212655_at	15	BDG29	AB011151	16q24.2
202514_at	15	BDH	AW139131	3q29
202515_at	15	BDH	BG251175	3q29
211715_s_at	14	BDH1	BC005844	3q29 /// 3q29
208945_s_at	15	BECN1	AF139131	17q21
208946_s_at	15	BECN1	AF139131	17q21
203319_s_at	15	BERF-1	L04282	3q21
202710_at	15	BET1	BC000899	7q21.1-q22
203511_s_at	15	BET3	AF041432	1p34.3

216063_at	13	beta globin pseudogene	N55205	---
218332_at	15	BEX1	NM_018476	Xq21-q23
215440_s_at	15	BEXL1	AL523320	Xq22.1-q22.3
218056_at	15	BFAR	NM_016561	16p13.13
214763_at	13	BFIT	AK023937	1p32.3
206956_at	15	BGLAP	NM_000711	1q25-q31
201261_x_at	15	BGN	BC002416	Xq28
213905_x_at	15	BGN	AA845258	18q22.3
201169_s_at	13	BHLHB2	BG326045	3p26
201170_s_at	15	BHLHB2	NM_003670	3p26
210273_at	12	BHPCDH; BH-Pcdh	AB006757	4p15
220580_at	10	BICC1	NM_025044	10q21.3
209203_s_at	15	BICD2	BC002327	8q22
212702_s_at	15	BICD2	N45111	9q22.32
213154_s_at	15	BICD2	AI934125	9q22.32
204493_at	12	BID	NM_001196	22q11.1
211725_s_at	15	BID	BC005884	---
209091_s_at	15	Bif-1	AF263293	1p22
210101_x_at	15	Bif-1	AF257318	1p22
202955_s_at	13	BIG1	AF084520	8q13
216266_s_at	15	BIG1	AK025637	8q13
209267_s_at	10	BIGM103	AB040120	4q22-q24
222199_s_at	15	BIN3	AK001289	8p21.2
211936_at	15	BIP; MIF2; GRP78	AF216292	9q33-q34.1
202076_at	15	BIRC2	NM_001166	11q22
202095_s_at	15	BIRC5	NM_001168	17q25
202896_s_at	14	Bit	AB023430	20p13 /// 20p13
202897_at	15	Bit	AB023430	20p13
218732_at	12	BIT1	NM_016077	17q23.2
219657_s_at	10	BKLF; MGC48279	AF285837	4p14
212447_at	15	BKLHD1	AF161402	7p14.3
216834_at	14	BL34	S59049	1q31
201032_at	15	BLCAP	NM_006698	20q11.2-q12
221534_at	12	Bles03; P5326	AF073483	11q13.1
202179_at	13	BLMH	NM_000386	17q11.2
202592_at	15	BLOC1S1	NM_001487	12q13-q14
221702_s_at	15	BLP2	AF353992	15q26.3 /// 15q26.3
203773_x_at	15	BLVRA	NM_000712	7p14-cen
211729_x_at	15	BLVRA	BC005902	7p14-cen
202201_at	15	BLVRB	NM_000713	19q13.1-q13.2
219555_s_at	11	BM039	NM_018455	16q23.2
35617_at	15	BMK1	U29725	17p11.2
205430_at	15	BMP5	NM_021073	6p12.1
205431_s_at	15	BMP5	NM_021073	6p12.1
209590_at	15	BMP7	AL157414	20q13
204832_s_at	10	BMPR1A	NM_004329	10q22.3
213578_at	15	BMPR1A	AI678679	10q22.3
203082_at	15	BMS1L	NM_014753	10q11.21
204930_s_at	14	BNIP1	NM_013979	5q33-q34
207829_s_at	12	BNIP1	NM_013978	5q33-q34

37226_at	15	BNIP1	U15172	5q33-q34
209308_s_at	15	BNIP2	BC002461	15q21.3
201848_s_at	15	BNIP3	U15174	10q26.3
201849_at	15	BNIP3	NM_004052	10q26.3
221478_at	15	BNIP3L	AL132665	8p21
221479_s_at	15	BNIP3L	AL132665	8p21
219345_at	15	BOLA1	NM_016074	1p36.13-q31.3
218775_s_at	13	BOMB	NM_024949	4q35.1
203502_at	15	BPGM	NM_001724	7q31-q34
205750_at	11	BPHL	NM_004332	6p25
209271_at	14	BPTF	AB032251	17q24.3
218522_s_at	13	BPY2IP1	NM_018174	19p13.12
206044_s_at	15	BRAF	NM_004333	7q34
213473_at	12	BRAP	AL042733	12q24.12
204520_x_at	15	BRD1	NM_014577	22q13.33
215460_x_at	13	BRD1	AL080149	22q13.33
208685_x_at	15	BRD2	AA902767	6p21.3
208686_s_at	15	BRD2	AA902767	6p21.3
203825_at	15	BRD3	NM_007371	9q34
202102_s_at	15	BRD4	BF718610	19p13.1
202103_at	15	BRD4	AI991631	19p13.1
221776_s_at	15	BRD7	AI885109	16q12
202227_s_at	15	BRD8	NM_006696	5q31
220155_s_at	13	BRD9	NM_023924	5p15.33
205550_s_at	15	BRE	NM_004899	2p23.3
218955_at	15	BRF2	NM_018310	8p11.23
208794_s_at	15	BRG1	D26156	19p13.2
214728_x_at	12	BRG1	AK026573	19p13.2
202427_s_at	15	BRP44	NM_015415	1q24
218024_at	15	BRP44L	NM_016098	6q27
202157_s_at	15	BRUNOL3	U69546	10p13
218090_s_at	15	BRWD2	NM_018117	10q26
222024_s_at	15	BRX	AK022014	15q24-q25
208908_s_at	15	BS-17; MGC9402	AF327443	5q15-q21
32837_at	15	BSCL	U56418	9q34.3
208906_at	12	BSCL2	BC004911	11q12-q13.5
218004_at	15	BSDC1	NM_018045	1p34.3
210993_s_at	15	BSP1	U54826	4q28
211256_x_at	10	BT2.1	U90142	6p22.1
217945_at	15	BTBD1	NM_025238	15q24
212993_at	15	BTBD14A	AA114166	9q34.3
202946_s_at	15	BTBD3	NM_014962	20p12.1
214499_s_at	12	BTF	AF249273	6q22-q23
221540_x_at	13	BTF2	AF078847	5q12.2-q13.3
208517_x_at	15	BTF3	NM_001207	5q13.3
214800_x_at	15	BTF3	R83000	5q13.3
38241_at	12	BTF3	U90548	6p21.3
200920_s_at	15	BTG1	AL535380	12q22
200921_s_at	15	BTG1	NM_001731	12q22
201236_s_at	14	BTG2	BG339064	1q32

205548_s_at	15	BTG3	NM_006806	21q21.1-q21.2
213134_x_at	15	BTG3	AI765445	21q21.1-q21.2
210970_s_at	15	BTKI	AF235049	6q14.3
203944_x_at	15	BTN2A1	NM_007049	6p22.1
215493_x_at	15	BTN2A2	AL121936	6p22.1
209846_s_at	12	BTN3A2	BC002832	6p22.1
204820_s_at	14	BTN3A3	NM_006994	6p21.3
217207_s_at	11	BTNLR	AK025267	5q35.3
209642_at	14	BUB1A; BUB1L; hBUB1	AF043294	2q14
203755_at	15	BUB1B	NM_001211	15q15
201456_s_at	15	BUB3	NM_004725	10q26
201457_x_at	15	BUB3	NM_004725	10q26
201458_s_at	15	BUB3	NM_004725	10q26
209974_s_at	15	BUB3	AF047473	10q26
218462_at	15	BXDC5	NM_025065	1p22.3
205839_s_at	11	BZRAP1	NM_004758	17q22-q23
202096_s_at	15	BZRP	NM_000714	22q13.31
200776_s_at	15	BZW1	AL518328	---
200777_s_at	15	BZW1	NM_014670	2q33
217809_at	15	BZW2	NM_014038	7p21.2
209183_s_at	14	C10orf10	AL136653	10q11.21
217905_at	13	C10orf119	NM_024834	10q26.13
218331_s_at	15	C10orf18	NM_017782	10p15.1
212500_at	15	C10orf22	AL049319	10q21.3
212502_at	15	C10orf22	AV713053	10q21.3
212771_at	15	C10orf38	AU150943	10p13
212419_at	15	C10orf56	AA131324	10q23.1
212423_at	15	C10orf56	AK024784	10q23.1
203481_at	15	C10orf6	AI655902	10q24.32
203482_at	14	C10orf6	AL133215	10q24.32
212121_at	15	C10orf61	BE962354	10q24.1
201725_at	15	C10orf7	NM_006023	10p13
55662_at	15	C10orf76	H27225	10q24.32
211376_s_at	15	C10orf86	BC005212	10q26.13
219067_s_at	15	C10orf86	NM_017615	10q26.13
219240_s_at	12	C10orf88	NM_024942	10q26.13
218297_at	14	C10orf97	NM_024948	10p13
218213_s_at	15	C11orf10	NM_014206	11q12-q13.1
217969_at	15	C11orf2	NM_013265	11q13
220560_at	13	C11orf21	NM_014144	11p15.5
52164_at	15	C11orf24	AA065185	11q13
43934_at	10	C11ORF4	AA479495	11cen-q22.3
202409_at	15	C11orf43	X07868	---
215692_s_at	10	C11orf8	BE645386	11p13
218220_at	15	C12orf10	NM_021640	12q13
221652_s_at	14	C12orf11	AF274950	12p12.1
221260_s_at	15	C12orf22	NM_030809	12q13.11-q13.12
218374_s_at	15	C12orf4	NM_020374	12p13.3
219099_at	13	C12orf5	NM_020375	12p13.3
218422_s_at	14	C13orf10	T79937	13q22.2

217769_s_at	15	C13orf12	BC003390	13q12.3
219303_at	15	C13orf7	NM_024546	13q22.2
202562_s_at	15	C14orf1	AL136658	14q24.3
218852_at	15	C14orf10	NM_017917	14q13.2
219757_s_at	15	C14orf101	NM_017799	14q22.3
219164_s_at	12	C14orf103	H96715	14q32.31
219166_at	14	C14orf104	NM_018139	14q21.3
218139_s_at	15	C14orf108	NM_018229	14q22.3
213246_at	15	C14orf109	AI346504	14q32.13
202623_at	15	C14orf11	NM_018453	14q13.1
217645_at	15	C14orf112	AW088547	14q24.1
218363_at	15	C14orf114	NM_018199	14q24.1
213794_s_at	15	C14orf120	AI269117	14q11.2
219203_at	12	C14orf122	NM_016049	14q11.2
212355_at	14	C14orf124	AI075450	14q11.2
213398_s_at	15	C14orf124	AI347090	14q11.2
218108_at	15	C14orf130	NM_018108	14q32.13
219086_at	14	C14orf131	NM_018335	14q32.33
218820_at	12	C14orf132	NM_020215	14q32.2
218431_at	15	C14orf133	NM_022067	14q24.3-q31
219972_s_at	15	C14orf135	NM_022495	14q23.1
218940_at	15	C14orf138	NM_024558	14q22.1
219563_at	12	C14orf139	NM_024633	14q32.2
214264_s_at	15	C14orf143	AI656610	14q32.11
212460_at	15	C14orf147	BE738425	14q13.1
213508_at	15	C14orf147	AA142942	14q13.1
212465_at	15	C14orf154	AA524500	14q32.32
221434_s_at	15	C14orf156	NM_031210	14q24.3 /// 14q24.3
218298_s_at	15	C14orf159	NM_024952	14q32.12
215548_s_at	15	C14orf163	AB020724	14q12
217768_at	15	C14orf166	NM_016039	14q22.1
219526_at	14	C14orf169	NM_024644	14q24.2
214659_x_at	13	C14orf170	AC007956	14q24.3
52741_at	12	C14orf172	AI962879	14q32.33
218144_s_at	12	C14orf173	NM_022489	14q32.33
202279_at	15	C14orf2	NM_004894	14q32.33
212643_at	15	C14orf32	AI671747	14q22.2
212644_s_at	15	C14orf32	AI671747	14q22.2
220173_at	15	C14orf45	NM_025057	14q24.2
221932_s_at	15	C14orf87	AA133341	14q32.2
201683_x_at	12	C14orf92	BE783632	14q11.2
201684_s_at	15	C14orf92	BF001668	14q11.2
201685_s_at	14	C14orf92	NM_014828	14q11.2
217448_s_at	11	C14orf92	AL117508	4q26
219009_at	11	C14orf93	NM_021944	14q11.2
218383_at	15	C14orf94	NM_017815	14q11.2
221688_s_at	15	C15orf12	AL136913	15q24
217898_at	15	C15orf24	NM_020154	15q13.2
218791_s_at	15	C15orf29	NM_024713	15q13.2
204495_s_at	13	C15orf39	NM_015492	15q23

221064_s_at	10	C16orf28	NM_023076	16p13.3
219315_s_at	15	C16orf30	NM_024600	16p13.3
218493_at	15	C16orf33	NM_024571	16p13.3
212109_at	15	C16orf34	AI590869	16p13.3
212115_at	15	C16orf34	AK023154	16p13.3
214273_x_at	15	C16orf35	AV704353	16p13.3
212736_at	15	C16orf45	BE299456	16p13.13
218183_at	15	C16orf5	NM_013399	16p13.3
204676_at	15	C16orf51	NM_015421	16p13.13-p12.3
218300_at	12	C16orf53	NM_024516	16p12.1
205781_at	15	C16orf7	NM_004913	16q24
218715_at	15	C17orf40	NM_018428	17q11.2
219146_at	14	C17orf42	NM_024683	---
218514_at	15	C17orf71	NM_018149	17q23.2
207996_s_at	13	C18orf1	NM_004338	18p11.2
209573_s_at	10	C18orf1	AF009424	18p11.2
209574_s_at	15	C18orf1	AI349506	18p11.2
212055_at	15	C18orf10	BF689173	18q12.2
213616_at	15	C18orf10	BF063896	18q12.2
213617_s_at	13	C18orf10	BF063896	18q12.2
219419_at	15	C18orf22	NM_024805	18q23
221190_s_at	15	C18orf8	NM_013326	18q11.2
52285_f_at	13	C18orf9	AW002970	18p11.21
221739_at	15	C19orf10	AL524093	19p13.3
212131_at	15	C19orf13	BG054966	19q13.12
222099_s_at	15	C19orf13	AW593859	19q13.12
214173_x_at	15	C19orf2	AW514900	19q12
222266_at	15	C19orf2	BF796940	19q12
55705_at	15	C19orf22	W07773	19p13.3
221587_s_at	12	C19orf24	BC000890	19p13.3
221267_s_at	15	C19orf27	NM_031213	19p13.3
213390_at	13	C19orf7	AB028987	19q13.33
200056_s_at	15	C1D	NM_006333	2p13-p12
219439_at	15	C1GALT1	NM_020156	7p14-p13
219283_at	15	C1GALT1C1	NM_014158	Xq25
213938_at	10	C1orf1	Z38645	3p21.31
220235_s_at	12	C1orf103	NM_018372	1p13.2
204699_s_at	13	C1orf107	NM_014388	1q32.2
204700_x_at	15	C1orf107	NM_014388	1q32.2
217893_s_at	14	C1orf108	NM_024595	1p34.2
206721_at	12	C1orf114	NM_021179	1q24
218546_at	15	C1orf115	NM_024709	1q42.11
218165_at	15	C1orf149	NM_022756	1p35.3-p33
201794_s_at	15	C1orf16	NM_014837	1q25
219988_s_at	15	C1orf164	NM_018150	1p34.1
219670_at	15	C1orf165	NM_024603	1p33
209883_at	15	C1orf17; KIAA0584	AF288389	1q25
218932_at	15	C1orf181	NM_017953	1p22.3
203550_s_at	14	C1orf2	NM_006589	1q21
220342_x_at	13	C1orf22	NM_017992	1q24-q25

220926_s_at	13	C1orf22	NM_025191	1q24-q25
220992_s_at	15	C1orf25	NM_030934	1q25.2 /// 1q25.2
218721_s_at	15	C1orf27	NM_017847	1q25
219063_at	12	C1orf35	NM_024319	1q42.13
212164_at	13	C1orf37	AL522296	1q32.1
212165_at	15	C1orf37	AF070537	1q32.1
207571_x_at	11	C1orf38	NM_004848	1p35.3
203960_s_at	15	C1orf41	NM_016126	1p32.1-p33
215691_x_at	15	C1orf41	AV702994	---
219406_at	15	C1orf50	NM_024097	1p34.1
219506_at	15	C1orf54	NM_024579	1q21.3
202809_s_at	15	C1orf60	NM_023015	1q22
202559_x_at	15	C1orf77	NM_015607	1q22
202560_s_at	15	C1orf77	NM_015607	1q22
220134_x_at	15	C1orf78	NM_018166	1p34.3
221710_x_at	15	C1orf78	BC006241	1p34.3
220199_s_at	15	C1orf80	NM_022831	1q42.12
203429_s_at	15	C1orf9	NM_016227	1q24
218232_at	14	C1QA	NM_015991	1p36.3-p34.1
202953_at	13	C1QB	NM_000491	1p36.3-p34.1
208910_s_at	15	C1QBP	L04636	17p13.3
214214_s_at	15	C1QBP	AU151801	17p13.3
218456_at	15	C1QDC1	NM_023925	12p11
205575_at	11	C1QL1	NM_006688	17q21
202877_s_at	13	C1QR1	NM_012072	20p11.22
202878_s_at	15	C1QR1	NM_012072	20p11.22
220975_s_at	13	C1QTNF1	NM_030968	17q25.3
220988_s_at	15	C1QTNF3	NM_030945	5p13-p12
212067_s_at	15	C1R	AL573058	---
208747_s_at	15	C1S	M18767	12p13
218448_at	15	C20orf11	NM_017896	20q13.33
221954_at	15	C20orf111	AA160474	20q13.11
218159_at	14	C20orf116	NM_023935	20p13
78047_s_at	12	C20orf127	AW001777	20q11.2
218010_x_at	15	C20orf149	NM_024299	20q13.33
219512_at	13	C20orf172	NM_024918	20q11.23
207713_s_at	14	C20orf18	NM_006462	20p13
221827_at	15	C20orf18	BE788439	20p13
218586_at	15	C20orf20	NM_018270	20q13.33
221741_s_at	15	C20orf21	AL096828	20q13.33
219570_at	12	C20orf23	NM_024704	20p11.23
217835_x_at	15	C20orf24	NM_018840	20q11.23
50314_i_at	14	C20orf27	AI761506	20p13
219706_at	12	C20orf29	NM_018347	20p13
220477_s_at	15	C20orf30	NM_014145	20p13
218094_s_at	15	C20orf35	NM_018478	20q13.12
219310_at	15	C20orf39	NM_024893	20p11.21
218089_at	12	C20orf4	NM_015511	20pter-q12
217737_x_at	15	C20orf43	NM_016407	20q13.31
217935_s_at	14	C20orf44	NM_018244	20q11.23

217851_s_at	15	C20orf45	NM_016045	20q13.32
218859_s_at	15	C20orf6	NM_016649	20p12.1
89948_at	14	C20orf67	AI743331	20q13.12
214812_s_at	14	C21orf106	D80006	21q22.3
215529_x_at	12	C21orf106	AI590053	21q22.3
214820_at	12	C21orf107	AJ002572	21q22.2
212996_s_at	11	C21orf108	AI803485	21q22.11
220311_at	12	C21orf127	NM_013240	21q21.3
212875_s_at	11	C21orf25	AP001745	21q22.3
213989_x_at	10	C21orf27	AB004853	21q22.13
202217_at	15	C21orf33	NM_004649	21q22.3
219004_s_at	15	C21orf45	NM_018944	21q22.11
218123_at	15	C21orf59	NM_017835	21q22.1
218515_at	15	C21orf66	NM_016631	21q21.3
221158_at	15	C21orf66	NM_013329	21q21.3
220941_s_at	10	C21orf91	NM_017447	21q21.1
209418_s_at	15	C22orf19	BC003615	22q12
204402_at	15	C22orf3	NM_012265	22q12.1-q12.2
33778_at	15	C22orf4; HSC79E021	AL096779	22q13.3
202027_at	15	C22orf5	NM_012264	22q12
219629_at	11	C22orf8	NM_017911	22q13
212421_at	13	C22orf9	AW468871	22q13.31
217118_s_at	10	C22orf9	AK025608	22q13.31
209233_at	15	C2f	U72514	12p13
214823_at	15	C2H2 zinc finger protein	AF033199	---
218037_at	15	C2orf17	NM_024293	2q36.1
221983_at	14	C2orf17	AL040896	2q36.1
221984_s_at	15	C2orf17	AL040896	2q36.1
222129_at	11	C2orf17	AK026155	2q36.1
204364_s_at	15	C2orf23	BE535746	2p11.2
204365_s_at	15	C2orf23	NM_022912	2p11.2
200070_at	15	C2orf24	BC001393	2q36.1
217883_at	15	C2orf25	NM_015702	2q23.3
219496_at	13	C2orf26	NM_023016	2q13
219329_s_at	15	C2orf28	NM_016085	2p23.3
216305_s_at	15	C2orf3	AC005034	2p11.2-p11.1
219137_s_at	15	C2orf33	NM_020194	2q36.3
219065_s_at	15	C2orf4	NM_015955	2p23.2
217767_at	15	C3	NM_000064	19p13.3-p13.2
219288_at	15	C3orf14	NM_020685	3p21.1
219114_at	15	C3orf18	NM_016210	3p21.3
220942_x_at	15	C3orf28	NM_014367	3q21.1
208925_at	15	C3orf4	AF161522	3p11-q11
214428_x_at	14	C4A	K02403	6p21.3
208451_s_at	10	C4B	NM_000592	6p21.3
210054_at	15	C4orf15	BC003648	4p16.3
219023_at	14	C4orf16	NM_018569	4q26
203600_s_at	13	C4orf8	NM_003704	4p16.3
214661_s_at	15	C4orf9	R06783	4p16.3
201309_x_at	15	C5orf13	NM_004772	5q22.2

201310_s_at	15	C5orf13	NM_004772	5q22.2
222344_at	13	C5orf13	AW972765	5q22.2
220495_s_at	15	C5orf14	NM_024715	5q31.2
203024_s_at	15	C5orf15	NM_020199	5q31.1
208872_s_at	15	C5orf18	AA814140	5q22-q23
208873_s_at	15	C5orf18	BC000232	5q22-q23
218588_s_at	15	C5orf3	NM_018691	5q31-q33
220751_s_at	10	C5orf4	NM_016348	5q31-q32
48030_i_at	12	C5orf4	H93077	5q31-q32
48031_r_at	14	C5orf4	H93077	5q31-q32
218518_at	15	C5orf5	NM_016603	5q31
220088_at	10	C5R1	NM_001736	19q13.3-q13.4
216521_s_at	10	c6.1A-TCRC	S72931	---
205457_at	11	C6orf106	NM_024294	6p21.31
217925_s_at	14	C6orf106	AL523965	6p21.31
212176_at	15	C6orf111	AA902326	6q16.3
212177_at	15	C6orf111	AW081113	6q16.3
212179_at	15	C6orf111	AW081113	6q16.3
213322_at	15	C6orf130	AL031778	6p21.1
212760_at	15	C6orf133	AB002347	6p21.1
218874_s_at	11	C6orf134	NM_024909	6p21.32
219294_at	10	C6orf139	NM_018132	6p12.3
212923_s_at	15	C6orf145	AK024828	6p25.1
218561_s_at	15	C6orf149	NM_020408	6p25.1
213312_at	15	C6orf162	NM_020425	6q15-q16.1
213314_at	15	C6orf162	NM_020425	6q15-q16.1
42361_g_at	11	C6orf18	AI588986	6p21.3
219307_at	15	C6orf210	NM_020381	6q21
218195_at	15	C6orf211	NM_024573	6q25.1
206707_x_at	14	C6orf32	NM_015864	6p22.3-p21.32
209829_at	15	C6orf32	AB002384	6p22.3-p21.32
220755_s_at	15	C6orf48	NM_016947	6p21.3
218233_s_at	15	C6orf49	NM_017601	6p21.31
208809_s_at	15	C6orf62	AL136632	6p22.1
213872_at	14	C6orf62	BE465032	6p22.1
219006_at	15	C6orf66	NM_014165	6q16.3
215207_x_at	13	C6orf68	BF695847	---
218877_s_at	15	C6orf75	NM_021820	6q11.1-q22.33
220094_s_at	15	C6orf79	NM_022102	6p24.3-p23
209479_at	15	C6orf80	BC000758	6q23.1-q24.1
206006_s_at	11	C6orf84	NM_014895	6q15
220329_s_at	15	C6orf96	NM_017909	6q25.1
32094_at	15	C6ST; C6ST1	AB017915	10q22.2
202992_at	15	C7	NM_000587	5p13
204215_at	15	C7orf23	NM_024315	7q21.1-q21.2
47083_at	12	C7orf26	AI280108	7p22.1
201973_s_at	15	C7orf28B	AL550875	---
213587_s_at	15	C7orf32	AI884867	7q36.1
221542_s_at	14	C8orf2	AL442077	8p11.2
221543_s_at	15	C8orf2	AL442077	8p11.2 /// 8p11.2

218777_at	11	C8orf20	NM_025232	8p21.3
219071_x_at	15	C8orf30A	NM_016458	8q24.3
219060_at	15	C8orf32	NM_018024	8q24.13
218187_s_at	15	C8orf33	NM_023080	8q24.3
218541_s_at	15	C8orf4	NM_020130	8p11.2
219124_at	12	C8orf41	NM_025115	8p12
220216_at	10	C8orf44	NM_019607	8q13.1
218905_at	15	C8orf52	NM_017864	8q22.1
205308_at	15	C8orf70	NM_016010	8q21.11
200767_s_at	12	C9orf10	NM_014612	9q22.32
200774_at	15	C9orf10	BE963765	9q22.32
213386_at	15	C9orf125	AV726900	---
204480_s_at	15	C9orf16	NM_024112	9q34.1
41047_at	15	C9orf16	AI885170	9q34.1
212848_s_at	15	C9orf3	BG036668	9q22.33
218992_at	15	C9orf46	NM_018465	9p24.1
205684_s_at	14	C9orf55	NM_017925	9p21.3
218998_at	15	C9orf6	NM_017832	9q32
61874_at	15	C9orf7	AL042496	9q34
208101_s_at	15	C9orf74	NM_030914	9q34.13
218979_at	14	C9orf76	NM_024945	9q22.1
218116_at	15	C9orf78	NM_016520	9q34.2
219276_x_at	15	C9orf82	NM_024828	9p21.1
220050_at	13	C9orf9	NM_018956	9q34
221865_at	13	C9orf91	BF969986	9q33.1
219147_s_at	15	C9orf95	NM_017881	9q21.31
219464_at	15	CA14	NM_012113	1q21
209301_at	15	CA2	M36532	8q22
206209_s_at	15	CA4	NM_000717	17q23
217873_at	15	CAB39	NM_016289	2q37.1
221972_s_at	15	Cab45	AL571362	1p36.33
218168_s_at	14	CABC1	NM_020247	1q42.13
202624_s_at	13	CABIN1	NM_012295	22q11.23
37652_at	14	CABIN1	AB002328	22q11.23
214933_at	13	CACNA1A	AA769818	19p13.2-p13.1
204811_s_at	13	CACNA2D2	NM_006030	3p21.3
207776_s_at	15	CACNB2	NM_000724	10p12
213714_at	15	CACNB2	AI040163	10p12
211592_s_at	15	CACNL1A1	L29536	12p13.3
34726_at	15	CACNLB3	U07139	12q13
211761_s_at	15	CACYBP	BC005975	1q24-q25
219572_at	15	CADPS2	NM_017954	---
208779_x_at	15	CAK	L20817	6p21.3
1007_s_at	15	CAK	U48705	---
210749_x_at	15	CAK	L11315	6p21.3
211297_s_at	11	CAK1	L20320	5q12.1
216903_s_at	15	CALC	AK022697	10q22.2
32541_at	15	CaM-PrP catalytic subunit	S46622	8p21.2
209002_s_at	15	CALCOCO1	BC003177	12q13.13
206331_at	15	CALCRL	NM_005795	2q32.2

201616_s_at	15	CALD1	AL577531	7q33
201617_x_at	15	CALD1	AI685060	7q33
205525_at	15	CALD1	NM_018495	7q33 /// 7q33
212077_at	15	CALD1	AL583520	7q33
202162_s_at	14	CALIF	AF180476	5q31-q33
202163_s_at	15	CALIF	AF180476	5q31-q33
202164_s_at	15	CALIF	AF180476	5q31-q33
200655_s_at	15	CALM1	NM_006888	14q24-q31
209563_x_at	15	CALM1	BC000454	14q24-q31
211984_at	15	CALM1	AI653730	14q24-q31
211985_s_at	15	CALM1	AI653730	14q24-q31
213688_at	12	CALM1	N25325	14q24-q31
207243_s_at	15	CALM2	NM_001743	2p21
200622_x_at	15	CALM3	AV685208	19q13.2-q13.3
200623_s_at	15	CALM3	NM_005184	19q13.2-q13.3
209817_at	14	CALNB; CALNA2	M29550	10q21-q22
200935_at	10	CALR	NM_004343	19p13.3-p13.2
212952_at	15	CALR	AA910371	19p13.3-p13.2
212953_x_at	14	CALR	BE251303	19p13.3-p13.2
214315_x_at	15	CALR	AI348935	19p13.3-p13.2
214316_x_at	14	CALR	AI378706	19p13.3-p13.2
200755_s_at	15	CALU	BF939365	7q32
200757_s_at	15	CALU	NM_001219	7q32
214845_s_at	14	CALU	AF257659	7q32
216713_at	12	CAM; CCM1	AL049325	---
209956_s_at	15	CAM2	U23460	22q12
210404_x_at	11	CAM2	AF078803	22q12
200653_s_at	15	CAMI	M27319	14q24-q31
204392_at	14	CAMK1	NM_003656	3p25.3
208095_s_at	15	CAMK2G	NM_001222	10q22
212669_at	13	CAMK2G	AI093569	10q22
212757_s_at	15	CAMK2G	BF111268	10q22
218309_at	15	CAMK2N1	NM_018584	1p36.13
213812_s_at	13	CAMKK	AK024748	12q24.2
212252_at	13	CAMKK2	AA181179	12q24.2
203538_at	15	CAMLG	NM_001745	5q23
212710_at	14	CAMSAP1	AL043774	9q34.3
212711_at	15	CAMSAP1	AA019977	9q34.3
212712_at	15	CAMSAP1	AL043774	9q34.3
212948_at	15	CAMTA2	AB020716	17p13.3
207483_s_at	15	CAND1	NM_018448	12q14
208683_at	15	CANP	M23254	1q41-q42
46323_at	15	CANT1	AL120741	17q25.3
200068_s_at	15	CANX	AI761759	5q35
208852_s_at	15	CANX	AI761759	5q35
200625_s_at	15	CAP1	NM_006367	1p34.2
213798_s_at	15	CAP1	AA806142	1p34.2
210844_x_at	15	CAP102; FLJ36832	D14705	5q31
212551_at	15	CAP2	NM_006366	6p22.3
212554_at	15	CAP2	N90755	6p22.3

204373_s_at	15	CAP350	NM_014810	1p36.13-q41
213165_at	15	CAP350	AI041204	1p36.13-q41
37012_at	15	CAPB	U03271	1p36.1
215623_x_at	11	CAPC; hCAP-C	AK002200	3q26.1
201850_at	14	CAPG	NM_001747	2cen-q24
200752_s_at	11	CAPN1	NM_005186	11q13
210944_s_at	15	CAPN3	BC003169	15q15.1-q21.1
203356_at	15	CAPN7	BE349584	3p24
203357_s_at	13	CAPN7	NM_014296	3p24
200001_at	15	CAPNS1	NM_001749	19q13.13
208374_s_at	15	CAPZA1	NM_006135	1p13.1
201237_at	15	CAPZA2	AV685920	7q31.2-q31.3
201238_s_at	15	CAPZA2	BC005338	7q31.2-q31.3
201949_x_at	15	CAPZB	AL572341	1p36.1
201950_x_at	15	CAPZB	NM_004930	1p36.1
200603_at	15	CAR	AL050038	17q23-q24
210026_s_at	15	CARD10	AW205153	22q13.1
204950_at	15	CARD8	NM_014959	19q13.33
218929_at	15	CARF	NM_017632	4q35.1
218384_at	15	CARHSP1	NM_014316	16p13.2
212512_s_at	15	CARM1	AA551784	19p13.2
202402_s_at	14	CARS	NM_001751	11p15.5
212971_at	15	CARS	AI769685	11p15.5
207842_s_at	15	CASC3	NM_007359	17q11-q21.3
210563_x_at	15	CASH	U97075	2q33-q34
210564_x_at	14	CASH	AF009619	2q33-q34
211316_x_at	15	CASH	AF009616	2q33-q34
211862_x_at	15	CASH	AF015451	2q33-q34
214486_x_at	10	CASH	AF041459	2q33-q34
211208_s_at	15	CASK	AB039327	Xp11.4
61297_at	12	CASKIN2	AL037338	17q25.3
202150_s_at	15	CASL	U64317	6p25-p24
202763_at	15	CASP3	NM_004346	4q34
213596_at	13	CASP4	AL050391	11q22.2-q22.3
209790_s_at	15	CASP6	BC000305	4q25
211464_x_at	10	CASP6	U20537	4q25
207181_s_at	14	CASP7	NM_001227	10q25
222201_s_at	15	CASP8AP2	AB037736	6q16.1
203984_s_at	15	CASP9	U60521	1p36.3-p36.1
219645_at	15	CASQ1	NM_001231	1q21
207317_s_at	15	CASQ2	NM_001232	1p13.3-p11
207467_x_at	15	CAST	NM_001750	5q15-q21
209701_at	12	CAST	D16217	5q15-q21
212586_at	15	CAST	AA195244	5q15-q21
220015_at	14	CASZ1	NM_017766	1p36.22
201432_at	15	CAT	NM_001752	11p13
211922_s_at	15	CAT	AY028632	11p13 /// 11p13
209746_s_at	15	CAT5; CLK-1	AF032900	16p13.11-p12.3
203065_s_at	15	CAV1	NM_001753	7q31.1
212097_at	15	CAV1	AU147399	7q31.1

203323_at	15	CAV2	BF197655	7q31.1
203324_s_at	15	CAV2	NM_001233	7q31.1
208204_s_at	11	CAV3	NM_001234	3p25
201478_s_at	13	CBF5	U59151	Xq28
207625_s_at	14	CBFA2T2	NM_005093	20q11
208056_s_at	12	CBFA2T3	NM_005187	16q24
202370_s_at	15	CBFB	NM_001755	16q22.1
213626_at	15	CBR4; FLJ14431	AL049442	4q32.3
212816_s_at	12	CBS	BE613178	21q22.3
220175_s_at	15	CBWD1	NM_020667	---
201518_at	15	CBX1	NM_006807	17q
200037_s_at	15	CBX3	NM_016587	7p15.2
201091_s_at	15	CBX3	NM_016587	7p15.2
212126_at	13	CBX5	BG391282	---
202047_s_at	14	CBX6	AI458128	22q13.1
212914_at	12	CBX7	AV648364	22q13.1
208720_s_at	15	CC1.4	L10911	20q11.23
215717_s_at	13	CCA	X62009	5q23-q31
206037_at	15	CCBL1	NM_004059	9q34.13
220466_at	12	CCDC15	NM_025004	11q24.2
206016_at	12	CCDC22	NM_014008	Xp11.23
219644_at	15	CCDC41	NM_016122	12q22
221069_s_at	15	CCDC44	NM_016360	17q24.2
217814_at	15	CCDC47	NM_020198	17q24.2
218655_s_at	15	CCDC49	NM_017748	17q21.2
218722_s_at	14	CCDC51	NM_024661	3p21.31
204716_at	12	CCDC6	NM_005436	10q21
206257_at	15	CCDC9	NM_015603	19q13.33
204606_at	14	CCL21	NM_002989	9p13
209101_at	15	CCN2	M92934	6q23.1
203418_at	15	CCNA2	NM_001237	4q25-q31
214710_s_at	15	CCNB1	BE407516	5q12
217988_at	15	CCNB1IP1	NM_021178	14q11.2
202705_at	15	CCNB2	NM_004701	15q21.3
201955_at	15	CCNC	AL137784	6q21
208711_s_at	13	CCND1	BC000076	11q13
200951_s_at	13	CCND2	AW026491	12p13
200953_s_at	15	CCND2	NM_001759	12p13
201700_at	15	CCND3	NM_001760	6p21
205034_at	12	CCNE2	NM_004702	8q22.1
208796_s_at	15	CCNG1	BC000196	5q32-q34
202769_at	15	CCNG2	AW134535	4q21.22
202770_s_at	14	CCNG2	NM_004354	4q21.22
211559_s_at	13	CCNG2	L49506	4q21.22
204093_at	15	CCNH	NM_001239	5q13.3-q14
208655_at	15	CCNI	BG530368	4q21.22
208656_s_at	15	CCNI	BG530368	4q21.22
219470_x_at	13	CCNJ	NM_019084	10pter-q26.12
220046_s_at	15	CCNL1	NM_020307	3q25.32
204645_at	15	CCNT2	NM_001241	2q21.3

213743_at	15	CCNT2	BE674119	2q21.3
221156_x_at	14	CCPG1	NM_004748	15q21.1
220671_at	12	CCRN4L	NM_012118	4q28.3
203522_at	15	CCS	NM_005125	11q13
201946_s_at	15	CCT2	AL545982	12q14.3
201947_s_at	15	CCT2	NM_006431	12q14.3
200910_at	15	CCT3	NM_005998	1q23
200877_at	15	CCT4	NM_006430	2p15
201326_at	15	CCT6A	BE737030	7p11.2
201327_s_at	15	CCT6A	NM_001762	7p11.2
200812_at	15	CCT7	NM_006429	2p13.2
200873_s_at	15	CCT8	NM_006585	21q22.11
208696_at	15	CCTE	AF275798	5p15.31
211676_s_at	15	CD119; IFNGR	AF056979	6q23-q24
201743_at	15	CD14	NM_000591	5q22-q32
209087_x_at	15	CD146; MUC18	AF089868	11q23.3
210869_s_at	15	CD146; MUC18	M29277	11q23.3
211340_s_at	15	CD146; MUC18	M28882	11q23.3
204306_s_at	15	CD151	NM_004357	11p15.5
204007_at	10	CD16	J04162	1q23
203645_s_at	14	CD163	NM_004244	12p13.3
208405_s_at	15	CD164	NM_006016	6q21
205789_at	14	CD1D	NM_001766	1q22-q23
209582_s_at	14	CD200	H23979	3q12-q13
209771_x_at	12	CD24	AA761181	6q21
216379_x_at	12	CD24	AK000168	---
219025_at	15	CD248	NM_020404	11q13
203593_at	15	CD2AP	NM_012120	6p12
202256_at	10	CD2BP2	BF793888	16p12.1
203799_at	15	CD302	NM_014880	2q24.2
208982_at	15	CD31	M37780	17q23
208983_s_at	14	CD31	M37780	17q23
218529_at	11	CD320	NM_016579	19p13.3-p13.2
209543_s_at	15	CD34	M81104	1q32
206488_s_at	15	CD36	NM_000072	7q11.2
209555_s_at	15	CD36	BE968792	7q11.2
35150_at	15	CD40	X60592	20q12-q13.2
204490_s_at	11	CD44	M24915	11p13
209835_x_at	13	CD44	BC004372	11p13
212014_x_at	14	CD44	AI493245	11p13
212063_at	15	CD44	BE903880	11p13
213857_s_at	15	CD47	BG230614	3q13.1-q13.2
203416_at	15	CD53	NM_000560	1p13
205173_x_at	15	CD58	NM_001779	1p13
211744_s_at	15	CD58	BC005930	1p13 /// 1p13
200983_x_at	15	CD59	BF983379	11p13
200985_s_at	15	CD59	NM_000611	11p13
212463_at	15	CD59	BE379006	---
200663_at	15	CD63	NM_001780	12q12-q13
200675_at	15	CD81	NM_004356	11p15.5

204440_at	12	CD83	NM_004233	6p23
210845_s_at	13	CD87	U08839	19q13
201005_at	15	CD9	NM_001769	12p13.3
201028_s_at	15	CD99	U82164	Xp22.32
201029_s_at	15	CD99	NM_002414	Xp22.32
221449_s_at	15	CDA08	NM_030790	16q11.2
217436_x_at	15	CDA12	M80469	---
208022_s_at	14	CDC14B	NM_003671	9q22.33
221555_x_at	15	CDC14B	BF792631	9q22.33
221556_at	15	CDC14B	BF792631	9q22.33
202717_s_at	15	CDC16	NM_003903	13q34
209658_at	15	CDC16	AF164598	13q34
209659_s_at	15	CDC16	AF164598	13q34
203213_at	15	CDC2	AL524035	10q21.1
203214_x_at	15	CDC2	NM_001786	10q21.1
202870_s_at	15	CDC20	NM_001255	1p34.1
202892_at	15	CDC23	NM_004661	5q31
201853_s_at	15	CDC25B	NM_021873	20p13
217878_s_at	10	CDC27	NM_001256	17q12-17q23.2
217879_at	15	CDC27	NM_001256	17q12-17q23.2
217880_at	15	CDC27	NM_001256	---
217881_s_at	14	CDC27	NM_001256	17q12-17q23.2
211289_x_at	11	CDC2L2	AF067524	1p36
212401_s_at	15	CDC2L2	AI767436	1p36.3
207318_s_at	15	CDC2L5	AJ297710	7p13
212897_at	11	CDC2L6	AI738802	6q22.1
212540_at	15	CDC34	BG476661	19p13.3
219343_at	15	CDC37L1	NM_017913	---
203376_at	15	CDC40	BG528818	6q22.1
208727_s_at	12	CDC42	BC003682	1p36.1
208728_s_at	15	CDC42	BC003682	1p36.1
214230_at	10	CDC42	R37664	1p36.1
203794_at	15	CDC42BPA	NM_014826	1q42.11
214464_at	10	CDC42BPA	NM_003607	1q42.11
217849_s_at	15	CDC42BPB	NM_006035	14q32.3
204693_at	11	CDC42EP1	NM_007061	22q13.1
209286_at	14	CDC42EP3	AI754416	2p21
209287_s_at	15	CDC42EP3	AI754416	2p21
209288_s_at	15	CDC42EP3	AL136842	2p21
218062_x_at	15	CDC42EP4	NM_012121	17q24-q25
218157_x_at	14	CDC42SE1	NM_020239	1q21.3
209055_s_at	14	CDC5L	AW268817	6p21
209056_s_at	15	CDC5L	NM_001253	6p21
209057_x_at	14	CDC5L	AB007892	6p21
203968_s_at	11	CDC6	NM_001254	17q21.3
204510_at	15	CDC7	NM_003503	1p22
221436_s_at	11	CDCA3	NM_031299	12p13 /// 12p13
218399_s_at	14	CDCA4	NM_017955	14q32.33
208834_x_at	15	CDH1	BC001865	17q11
220115_s_at	14	CDH10	NM_006727	5p14-p13

204726_at	10	CDH13	NM_001257	16q24.2-q24.3
206898_at	15	CDH19	NM_021153	18q22-q23
203441_s_at	15	CDH2	NM_001792	18q11.2
204677_at	15	CDH5	NM_001795	16q22.1
201253_s_at	15	CDIPT	NM_006319	16p12.1
210559_s_at	15	CDK1	D88357	10q21.1
203468_at	10	CDK10	NM_003674	16q24
212899_at	15	CDK11	AB028951	6q22.1
204252_at	15	CDK2	M68520	12q13
201938_at	15	CDK2AP1	NM_004642	12q24.31
203252_at	14	CDK2AP2	NM_005851	11q13
202246_s_at	15	CDK4	NM_000075	12q14
218315_s_at	14	CDK5RAP1	NM_016408	20pter-q11.23
218740_s_at	15	CDK5RAP3	NM_025197	17q21.32
204831_at	15	CDK8	R59697	13q12
219831_at	10	CDKL3	NM_016508	5q31
202284_s_at	15	CDKN1A	NM_000389	6p21.2
209112_at	15	CDKN1B	BC001971	12p13.1-p12
213182_x_at	15	CDKN1C	R78668	11p15.5
213183_s_at	15	CDKN1C	N95363	11p15.5
213348_at	15	CDKN1C	N33167	11p15.5
216894_x_at	15	CDKN1C	D64137	---
219534_x_at	15	CDKN1C	NM_000076	11p15.5
204159_at	15	CDKN2C	NM_001262	1p32
214880_x_at	12	CDM	D90453	7q33
204154_at	15	CDO1	NM_001801	5q22-q23
216751_at	10	CDRT4	AK024879	---
205709_s_at	13	CDS	U65887	4q21.23
212862_at	15	CDS2	AL568982	20p13
212864_at	15	cds2	Y16521	20p13
203098_at	14	CDYL	AL050164	6p25.1
211657_at	10	CEACAM6	M18728	19q13.2
212501_at	15	CEBPB	AL564683	20q13.1
213006_at	11	CEBPD	AV655640	8q11.21
204203_at	13	CEBPG	NM_001806	19q13.12
203341_at	15	CEBPZ	NM_005760	2p22.3
219505_at	11	CECR1	NM_017424	22q11.2
218592_s_at	15	CECR5	NM_017829	---
204235_s_at	15	CED6	AF200715	2q32.3-q33
41660_at	12	CELSR1	AL031588	22q13.3
204029_at	15	CELSR2	NM_001408	1p21
36499_at	12	CELSR2	D87469	1p21
209172_s_at	15	CENF; PRO1779	U30872	1q32-q41
204962_s_at	14	CENPA	NM_001809	2p24-p21
204739_at	15	CENPC1	NM_001812	4q12-q13.3
207828_s_at	15	CENPF	NM_005196	1q32-q41
212476_at	15	CENTB2	D26069	3q29
214102_at	13	CENTD1	AK023737	---
212516_at	11	CENTD2	AB018325	11q13.3
34206_at	14	CENTD2	AB018325	11q13.3

218950_at	15	CENTD3	NM_022481	5q31.3
204066_s_at	15	CENTG2	NM_014914	---
221971_x_at	15	CENTG2	BE672818	10q11.22
204251_s_at	14	CEP164	NM_014956	11q23.3
220071_x_at	15	CEP27	NM_018097	15q14
214721_x_at	15	CEP4	AL162074	17q24-q25
218542_at	14	CEP55	NM_018131	10q23.33
203493_s_at	15	CEP57	NM_014679	11q21
203494_s_at	15	CEP57	NM_014679	11q21
209862_s_at	15	CEP57	BC001233	11q21
219242_at	11	CEP63	NM_025180	3q22.1
207971_s_at	12	CEP68	NM_015147	2p14
219375_at	14	CEPT1	NM_006090	1p13.2
218421_at	15	CERK	NM_022766	22q13.31
209667_at	15	CES2	BF033242	16q22.1
209194_at	15	CETN2	BC005334	Xq28
209662_at	15	CETN3	BC005383	5q14.3
212610_at	15	CFC	U79291	12q24
203166_at	15	CFDP1	NM_006324	16q22.2-q22.3
200021_at	15	CFL1	NM_005507	11q13
208485_x_at	15	CFLAR	NM_003879	2q33-q34
214730_s_at	15	CFR-1	AK025457	16q22-q23
213375_s_at	13	CG018	N80918	13q12-q13
206861_s_at	15	CGGBP1	NM_003663	3p12-p11.1
212405_s_at	14	CGI-01	AK001172	1q24-q25.3
212407_at	12	CGI-01	AL049669	1q24-q25.3
218036_x_at	15	CGI-07	AL520719	3q26.1
202194_at	15	CGI-100	AL117354	1pter-q31.3
209404_s_at	15	CGI-109; FLJ90481	AF151867	5q23.1
219037_at	15	CGI-115	NM_016052	1q41
218628_at	15	CGI-116	NM_016053	12q23.3
219030_at	15	CGI-121	NM_016058	2p24.3-p24.1
203259_s_at	15	CGI-130	BC001671	6q13-q24.3
222360_at	11	CGI-30	AI291720	1p21.2
210076_x_at	15	CGI-55	AF151813	1p31-p22
222133_s_at	15	CGI-72; MGC64923	AK022280	8q24.22
204097_s_at	15	CGI-79	AF078865	Xq26.1
218549_s_at	15	CGI-90; FLJ20665	AK000672	8q21.2
33307_at	15	CGI-96	AL022316	22q13.2-q13.31
204605_at	15	CGRRF1	NM_006568	14q22.2
214785_at	13	CHAC	AB023203	9q21
214426_x_at	12	CHAF1A	BF062223	19p13.3
217720_at	15	CHCHD2	NM_016139	7p11.2
211927_x_at	15	CHCHD3	BE963164	---
217972_at	15	CHCHD3	NM_017812	7q33
218642_s_at	15	CHCHD7	NM_024300	8q11.23
220647_s_at	14	CHCHD8	NM_016565	11q13.3
204258_at	15	CHD1	NM_001270	5q15-q21
212539_at	15	CHD1L	AI422099	1q12
201183_s_at	15	CHD4	NM_001273	12p13

218829_s_at	14	CHD7	AI475906	8q12.1
212571_at	15	CHD8	U00955	14q11.2
212615_at	15	CHD9	AI742305	16q12.2
212616_at	15	CHD9	BF668950	16q12.2
205393_s_at	11	CHEK1	NM_001274	11q24-q24
205394_at	11	CHEK1	NM_001274	11q24-q24
202230_s_at	15	CHERP	NM_006387	19p13.1
205022_s_at	15	CHES1	NM_005197	14q24.3-q31
218031_s_at	15	CHES1	NM_018589	14q32.11
218803_at	15	CHFR	NM_018223	12q24.33
219049_at	15	ChGn	NM_018371	8p21.3
219492_at	15	CHIC2	NM_012110	4q11
204233_s_at	10	CHKA	AI991328	11q13.1
204193_at	15	CHKB	NM_005198	22q13.33
216594_x_at	15	chlordecone reductase homolog	S68290	10p15-p14
209160_at	13	c-hluPGFS	AB018580	10p15-p14
218177_at	10	CHMP1B	NM_020412	18p11.22-p11.21
218178_s_at	13	CHMP1B	NM_020412	18p11.22-p11.21
202536_at	15	CHMP2.5	AK002165	3p12.1
202121_s_at	15	CHMP2A	NM_014453	19q
202537_s_at	15	CHMP2B	NM_014043	3p12.1
202538_s_at	15	CHMP2B	NM_014043	3p12.1
218571_s_at	15	CHMP4A	NM_014169	14q11.2
218572_at	12	CHMP4A	NM_014169	14q11.2
218085_at	15	CHMP5	NM_015961	9p13.3
219356_s_at	15	CHMP5	NM_016410	9p13.3
212313_at	15	CHMP7	BC004344	8p21.2
216979_at	14	CHN	X89894	9q22
212624_s_at	15	CHN1	BF339445	2q31-q32.1
207486_x_at	13	CHN2	NM_004067	7p15.3
213385_at	15	CHN2	AK026415	7p15.3
218566_s_at	15	CHORDC1	NM_012124	11q14.3
214665_s_at	15	CHP; SLC9A1BP	AK000095	---
202175_at	15	CHPF	NM_024536	2q36.1
203207_s_at	15	CHPPR	BF214329	8q12.3
203208_s_at	15	CHPPR	BF214329	8q12.3
207859_s_at	10	CHRNB3	NM_000749	8p11.2
214246_x_at	15	CHRNE	AI859060	17p13-p12
205567_at	15	CHST1	NM_003654	11p11.2-p11.1
204065_at	12	CHST10	NM_004854	2q12.1
218927_s_at	15	CHST12	BC002918	7p22
203921_at	14	CHST2	NM_004267	3q24
220446_s_at	11	CHST4	NM_005769	16q22.2
219182_at	15	CHST5	NM_024533	16q22.3
64900_at	11	CHST5	AA401703	16q22.3
221059_s_at	13	CHST6	NM_021615	16q22
203044_at	15	CHSY1	NM_014918	15q26.3
212832_s_at	15	ch-TOG	AI143124	11p11.2
203428_s_at	15	CIA	AB028628	6q22.31
208424_s_at	15	CIAPIN1	NM_020313	16q13-q21

208968_s_at	15	CIAPIN1	BC002568	16q13-q21
201953_at	15	CIB1	NM_006384	15q25.3-q26
205007_s_at	15	CIB2	NM_006383	15q24
205008_s_at	15	CIB2	NM_006383	15q24
212784_at	13	CIC	AB002304	19q13.2
221188_s_at	14	CIDEB	NM_014430	14q11.2
219398_at	13	CIDEC	NM_022094	3p25.3
216591_s_at	14	CII-3	AF080579	---
206227_at	14	CILP	NM_003613	15q22
218267_at	15	CINP	NM_016550	14q32.33 /// 14q32.33
200810_s_at	15	CIRBP	NM_001280	19p13.3
200811_at	15	CIRBP	NM_001280	19p13.3
207980_s_at	15	CITED2	NM_006079	6q23.3
205516_x_at	13	CIZ1	NM_012127	9q34.1
201804_x_at	15	CKAP1	NM_001281	19q13.11-q13.12
211759_x_at	15	CKAP1	BC005969	19q13.11-q13.12
216194_s_at	15	CKAP1	AD001527	19q13.11-q13.12
218252_at	15	CKAP2	NM_018204	13q14
200998_s_at	15	CKAP4	AW029619	12q24.11
200999_s_at	15	CKAP4	NM_006825	12q24.11
200884_at	15	CKB	NM_001823	14q32
209211_at	11	CKLF	AF132818	13q21.33
209212_s_at	11	CKLF	AF132818	13q21.33
219161_s_at	14	CKLF	NM_016951	16q22.1
221058_s_at	15	CKLF	NM_016326	16q22.1
204810_s_at	15	CKM	NM_001824	19q13.2-q13.3
205295_at	15	CKMT2	NM_001825	5q13.3
201897_s_at	15	CKS1B	BC001425	1q21.2
204170_s_at	15	CKS2	NM_001827	9q22
213379_at	15	CL640; FLJ26072	AF091086	4q21.3
209508_x_at	15	clarp	AF005774	2q33-q34
209939_x_at	15	clarp	AF005775	2q33-q34
212752_at	15	CLASP1	AA176798	2q14.2-q14.3
212306_at	15	CLASP2	AI741784	3p22.3
212308_at	14	CLASP2	AL137636	3p22.3
212309_at	14	CLASP2	AI741784	3p22.3
38069_at	14	CLC7; CLC-7; OPTA2	Z67743	16p13
207855_s_at	14	CLCC1	NM_015127	1p13.3
209143_s_at	15	CLCI	AF005422	11q13.5-q14
201734_at	15	CLCN3	AI760629	4q33
201735_s_at	15	CLCN3	NM_001829	4q33
203950_s_at	11	CLCN6	NM_001286	1p36
207047_s_at	12	CLCNKA	NM_004070	1p36
205328_at	15	CLDN10	NM_006984	13q31-q34
214135_at	10	CLDN18	BE551219	3q22.3
204482_at	15	CLDN5	NM_003277	22q11.21
212351_at	15	CLE	U23028	3q27.3
211709_s_at	13	CLEC11A	BC005810	19q13.3 /// 19q13.3
219761_at	15	CLEC1A	NM_016511	12p13.31

209732_at	15	CLEC2B	BC005254	12p13-p12
205200_at	15	CLEC3B	NM_003278	3p22-p21.3
205830_at	15	CLGN	NM_004362	4q28.3-q31.1
208791_at	15	CLI	M25915	8p21-p12
208792_s_at	15	CLI	M25915	8p21-p12
213415_at	14	CLIC2	AI768628	Xq28
201559_s_at	15	CLIC4	AF109196	1p36.11
201560_at	15	CLIC4	NM_013943	1p36.11
221881_s_at	14	CLIC4	AI638420	1p36.11
213317_at	15	CLIC5	AL049313	---
217628_at	12	CLIC5	BF032808	---
219866_at	15	CLIC5	NM_016929	6p12.1-21.1
35160_at	13	CLIM2	AF064491	10q24-q25
210716_s_at	12	CLIP	M97501	12q24.3
214683_s_at	15	CLK1	AI251890	2q33
210346_s_at	15	CLK1; STY; CLK4	AF212224	2q33
202140_s_at	15	CLK3	NM_003992	15q24
209275_s_at	11	CLN3	AF015593	16p12.1
210859_x_at	14	CLN3	AF077973	16p12.1
204084_s_at	15	CLN5	NM_006493	13q21.1-q32
204085_s_at	11	CLN5	NM_006493	13q21.1-q32
214252_s_at	14	CLN5	AV700514	13q21.1-q32
204980_at	13	CLOCK	NM_004898	4q12
202799_at	15	CLPP	NM_006012	19p13.3
201640_x_at	11	CLPTM1	NM_001294	19q13.2-q13.3
211136_s_at	14	CLPTM1	BC004865	19q13.2-q13.3
204809_at	15	CLPX	NM_006660	15q22.2-q22.3
201561_s_at	15	CLSTN1	NM_014944	1p36.22
200960_x_at	15	CLTA	NM_007096	9p13
204050_s_at	15	CLTA	NM_001833	9p13
216295_s_at	15	CLTA	X81636	9p13
205172_x_at	15	CLTB	NM_007097	4q2-q3
206284_x_at	15	CLTB	NM_001834	4q2-q3
211043_s_at	15	CLTB	BC006332	4q2-q3 /// 4q2-q3
200614_at	15	CLTC	NM_004859	17q11-qter
222043_at	14	CLU	AI982754	8p21-p12
204576_s_at	15	CLUAP1	AA207013	16p13.3
204577_s_at	12	CLUAP1	NM_024793	16p13.3
205518_s_at	11	CMAH	NM_003570	--- /// ---
218111_s_at	15	CMAS	NM_018686	12p12.2
212977_at	15	CMKOR1	AI817041	2q37.3
217870_s_at	15	CMPK	NM_016308	---
208693_s_at	15	CMT2D	D30658	7p15
217947_at	15	CMTM6	NM_017801	3p22.3
201774_s_at	14	CNAP1; KIAA0159	AK022511	12p13.3
217752_s_at	13	CNDP2	NM_018235	18q22.3
201653_at	15	CNIH	NM_005776	14q22.2
203951_at	15	CNN1	NM_001299	19p13.2-p13.1
201605_x_at	11	CNN2	NM_004368	21q11.1
201445_at	15	CNN3	NM_001839	1p22-p21

206818_s_at	14	CNNM2	NM_017649	10q24.33
220739_s_at	15	CNNM3	NM_017623	2p12-p11.2
200860_s_at	15	CNOT1	BC000779	16q13
200861_at	14	CNOT1	NM_016284	16q13
217798_at	15	CNOT2	NM_014515	12q14.3-q15
222182_s_at	15	CNOT2	BG105204	12q14.3-q15
203239_s_at	14	CNOT3	NM_014516	19q13.4
203291_at	15	CNOT4	NM_013316	7q22-qter
217970_s_at	13	CNOT6	NM_015455	5q35.3
218250_s_at	15	CNOT7	NM_013354	8p22-p21.3
219400_at	11	CNTNAP1	NM_003632	17q21
219301_s_at	10	CNTNAP2	NM_014141	7q35-q36
208853_s_at	15	CNX; P90; IP90	L18887	5q35
201913_s_at	11	COASY	NM_025233	17q12-q21
203642_s_at	15	COBLL1	NM_014900	2q24.3
202757_at	15	COBRA1	NM_015456	9q34
212189_s_at	14	COD1	AK022874	16q22.1
203073_at	15	COG2	NM_007357	1q42.2
203629_s_at	15	COG5	AU152134	7q22-q31
203630_s_at	15	COG5	NM_006348	7q22-q31
213190_at	11	COG7	R61519	16p12.3
213243_at	15	COH1	AI052003	8q22.2
203654_s_at	14	COIL	NM_004645	17q22-q23
204320_at	15	COL11A1	NM_001854	1p21
37892_at	15	COL11A1	J04177	1p21
213870_at	10	COL11A2	AL031228	6p21.3
216993_s_at	15	COL11A2	U32169	---
212865_s_at	15	COL14A1	BF449063	8q23
203477_at	15	COL15A1	NM_001855	9q21-q22
204345_at	15	COL16A1	NM_001856	1p35-p34
209081_s_at	15	COL18A1	AF018081	21q22.3
209082_s_at	15	COL18A1	AF018081	21q22.3
202310_s_at	15	COL1A1	K01228	17q21.3-q22.1
202311_s_at	15	COL1A1	AI743621	17q21.3-q22.1
217430_x_at	14	COL1A1	Y15916	---
202403_s_at	15	COL1A2	NM_000089	7q22.1
202404_s_at	15	COL1A2	NM_000089	7q22.1
208096_s_at	15	COL21A1	NM_030820	6p12.3-p11.2
201852_x_at	15	COL3A1	NM_000090	2q31
211161_s_at	15	COL3A1	AF130082	---
215076_s_at	15	COL3A1	AU144167	2q31
211980_at	15	COL4A1	AI922605	13q34
211981_at	15	COL4A1	NM_001845	13q34
211343_s_at	13	COL4A2	M33653	10q22
211964_at	15	COL4A2	X05610	13q34
211966_at	15	COL4A2	AA909035	13q34
222073_at	10	COL4A3	AI694562	2q36-q37
213110_s_at	15	COL4A5	AW052179	Xq22
213992_at	15	COL4A6	AI889941	Xq22
203325_s_at	15	COL5A1	AI130969	9q34.2-q34.3

212488_at	15	COL5A1	N30339	9q34.2-q34.3
212489_at	15	COL5A1	AI983428	9q34.2-q34.3
221729_at	15	COL5A2	AL575735	2q14-q32
221730_at	15	COL5A2	AL575735	2q14-q32
218975_at	10	COL5A3	NM_015719	19p13.2
52255_s_at	15	COL5A3	AI984221	19p13.2
212091_s_at	10	COL6A1	AI141603	21q22.3
213428_s_at	15	COL6A1	AA292373	21q22.3
209156_s_at	15	COL6A2	AY029208	21q22.3
213290_at	15	COL6A2	AL531750	21q22.3
201438_at	15	COL6A3	NM_004369	2q37
204136_at	13	COL7A1	NM_000094	3p21.1
213622_at	13	COL9A2	AI733465	1p33-p32
219873_at	15	COLEC11	NM_024027	2p25.3
221019_s_at	15	COLEC12	NM_030781	18pter-p11.3
209230_s_at	15	COM1	AF135266	16p11.2
218439_s_at	15	COMMD10	BC005179	5q23.1
218048_at	15	COMMD3	NM_012071	10pter-q22.1
209132_s_at	15	COMMD4	BE313890	15q23
218351_at	15	COMMD8	NM_017845	4p12
218072_at	14	COMMD9	NM_014186	11p13
208817_at	15	COMT	BC000419	22q11.21-q11.23
208818_s_at	15	COMT	BC000419	22q11.21-q11.23
208684_at	15	COPA	U24105	1q23-q25
214336_s_at	12	COPA	AI621079	1q23-q25
201358_s_at	15	COPB	NM_016451	11p15.2
201359_at	15	COPB	NM_016451	11p15.2
201098_at	15	COPB2	NM_004766	3q23
201264_at	12	COPE	NM_007263	19p13.11
217749_at	15	COPG	NM_016128	3q21.3
202467_s_at	15	COPS2	NM_004236	15q21.2
209838_at	13	COPS2	AA496247	15q21.2
202078_at	15	COPS3	NM_003653	17p11.2
218042_at	15	COPS4	NM_016129	4q21.3
201652_at	15	COPS5	NM_006837	8q13.1
201405_s_at	15	COPS6	NM_006833	7q22.1
213504_at	15	COPS6	W63732	7q22.1
202141_s_at	15	COPS8	BC003090	2q37.3
202142_at	14	COPS8	BC003090	2q37.3
202143_s_at	15	COPS8	BC003090	2q37.3
214260_at	12	COPS8	AI079287	2q37.3
217726_at	15	COPZ1	NM_016057	12q13.2-q13.3
221227_x_at	13	COQ3	NM_017421	6q16.3
218328_at	13	COQ4	NM_016035	9q34.13
218760_at	15	COQ6	NM_015940	14q24.2
210820_x_at	15	COQ7	AL136647	16p13.11-p12.3
220356_at	15	CORIN	NM_006587	4p13-p12
64486_at	15	CORO1B	AI341234	11q13.1
221676_s_at	15	CORO1C	BC002342	12q24.1
209789_at	13	CORO2B	BF939649	15q22.31

213454_at	14	CORT	AL578583	---
203858_s_at	15	COX10	NM_001303	17p12-17p11.2
203551_s_at	15	COX11	NM_004375	17q22
211727_s_at	15	COX11	BC005895	17q22 /// 17q22
219547_at	15	COX15	NM_004376	10q24
221550_at	11	COX15	BC002382	10q24
203880_at	15	COX17	NM_005694	3q13.33
200086_s_at	15	COX4I1	AA854966	16q22-qter
202698_x_at	15	COX4I1	NM_001861	16q22-qter
212312_at	15	COX4I2	AL117381	20q11.21
218057_x_at	15	COX4NB	BC001472	16q24
203663_s_at	15	COX5A	NM_004255	15q25
202343_x_at	15	COX5B	NM_001862	2cen-q13
211025_x_at	15	COX5B	BC006229	2cen-q13
213735_s_at	15	COX5B	AI557312	2cen-q13
200925_at	15	COX6A1	NM_004373	12q24.2
206353_at	15	COX6A2	NM_005205	16p
201441_at	15	COX6B1	NM_001863	19q13.1
201754_at	15	COX6C	NM_004374	8q22-q23
217191_x_at	15	COX6CP1	AF042163	---
204570_at	15	COX7A1	NM_001864	19q13.1
201597_at	15	COX7A2	NM_001865	6q12
201256_at	15	COX7A2L	NM_004718	2p22.1
202110_at	15	COX7B	NM_001866	Xq13.3
217329_x_at	15	COX7BP1	AF042164	---
201134_x_at	15	COX7C	NM_001867	5q14
213846_at	15	COX7C	AA382702	5q14
217491_x_at	15	COX7CP1	AF042165	---
201119_s_at	15	COX8A	NM_004074	11q12-q13
204662_at	14	CP110	NM_014711	16p13.11
209338_at	15	CP2	U03494	12q13
205624_at	10	CPA3	NM_001870	3q21-q25
201940_at	14	CPD	AA897514	17p11.1-q11.2
201941_at	15	CPD	BE349147	17p11.1-q11.2
201116_s_at	13	CPE	NM_001873	4q32.3
201117_s_at	14	CPE	NM_001873	4q32.3
205773_at	12	CPEB3	NM_014912	10q23.33
210145_at	14	cPLA2	M68874	1q25
206918_s_at	15	CPNE1	NM_003915	20q11.23
212170_at	12	CPNE1	BF447705	20q11.23
202118_s_at	15	CPNE3	AA541758	8q21.2
202119_s_at	15	CPNE3	NM_003909	8q21.2
204172_at	15	CPOX	NM_000097	3q12
221511_x_at	15	CPR8; KIAA1254	AF212228	15q21.1
222156_x_at	14	CPR8; KIAA1254	AK022459	15q21.1
201639_s_at	12	CPSF1	NM_013291	8q24.23
33132_at	15	CPSF160	U37012	8q24.23
217994_x_at	15	CPSF3L	NM_017871	1p36.33
206688_s_at	12	CPSF4	NM_006693	7q22.1
213461_at	13	CPSF5	AI800983	16q13

202469_s_at	15	CPSF6	NM_007007	12q14.3
221675_s_at	15	CPT; CPT1	AF195624	12q
203633_at	14	CPT1A	BF001714	11q13.1-q13.2
210069_at	15	CPT1-M	U62733	22q13.33
210070_s_at	15	CPT1-M	U62733	22q13.33
208146_s_at	15	CPVL	NM_031311	7p15-p14
209522_s_at	15	CRAT	BC000723	9q34.1
218142_s_at	15	CRBN	NM_016302	3p26.2
204312_x_at	11	CREB1	AI655737	2q34
204313_s_at	15	CREB1	AA161486	2q34
204314_s_at	15	CREB1	NM_004379	2q34
214513_s_at	11	CREB1	M34356	2q34
212345_s_at	15	CREB3L2	BE675139	7q34
205931_s_at	12	CREB5	NM_004904	7p15
202160_at	15	CREBBP	NM_004380	16p13.3
201988_s_at	15	CREBL2	BF438056	12p13
201989_s_at	15	CREBL2	NM_001310	12p13
201990_s_at	15	CREBL2	NM_001310	12p13
201200_at	15	CREG1	NM_003851	1q24
203368_at	15	CRELD1	NM_015513	3p25.3
218358_at	15	CRELD2	NM_024324	22p13
207630_s_at	15	CREM	NM_001881	10p11.21
209967_s_at	15	CREM	D14826	10p11.21
214508_x_at	15	CREM	U44836	10p11.21
202551_s_at	15	CRIM1	BG546884	2p21
202552_s_at	15	CRIM1	NM_016441	2p21
205081_at	15	CRIP1	NM_001311	7q11.23
218643_s_at	15	CRIPT	BF540954	2p21
202224_at	15	CRK	NM_016823	---
202225_at	15	CRK	AW612311	---
212180_at	15	CRKL	AK000311	22q11
219226_at	10	CRKRS	NM_016507	17q21.2
205474_at	15	CRLF3	NM_015986	17q11.2
210815_s_at	14	CRLR; CGRPR	U17473	2q32.2
208775_at	15	CRM1	D89729	2p16
202517_at	14	CRMP1	NM_001313	4p16.1-p15
219913_s_at	15	CRNKL1	NM_016652	20p11.2
203804_s_at	15	CROP	NM_006107	17q21
208835_s_at	15	CROP	AW089673	17q21
220044_x_at	15	CROP	NM_016424	17q21
204573_at	15	CROT	NM_021151	7q21.1
202611_s_at	11	CRSP2	NM_004229	Xp11.4-p11.2
215167_at	14	CRSP2	BE567032	Xp11.4-p11.2
218846_at	15	CRSP3	NM_004830	6q22.33-q24.1
51176_at	15	CRSP8	AA131335	9q34.1-q34.3
204349_at	11	CRSP9	BC005250	5q33.3
204350_s_at	15	CRSP9	NM_004270	5q33.3
221204_s_at	12	CRTAC1	NM_018058	10q22
201380_at	15	CRTAP	NM_006371	3p22
207159_x_at	12	CRTC1	NM_025021	19p13.11

218648_at	15	CRTC3	NM_022769	15q26.1
209283_at	15	CRYA2	AF007162	11q22.3-q23.1
220753_s_at	15	CRYL1	NM_015974	13q12.11
205489_at	14	CRYM	NM_001888	16p13.11-p12.3
202950_at	15	CRYZ	NM_001889	1p31-p22
219767_s_at	15	CRYZL1	NM_005111	21q21.3
208660_at	15	CS	BC000105	12q13.2-q13.3
211476_at	15	CS-1; C4orf5	AY013295	4q26-q27
200066_at	15	CSA2	AF182645	2p15-p14
221139_s_at	15	CSAD	NM_015989	12q13.11-q14.3
201160_s_at	15	CSDA	AL556190	12p13.1
201161_s_at	15	CSDA	AL556190	12p13.1
219939_s_at	15	CSDE1	NM_007158	1p22
201111_at	15	CSE1	AF053641	20q13
201112_s_at	15	CSE1	AF053641	20q13
210766_s_at	15	CSE1	AF053640	20q13
203104_at	13	CSF1R	NM_005211	5q33-q35
205159_at	12	CSF2RB	AV756141	22q13.1
212737_at	15	CSH2	AL513583	5q31.3-q33.1
213750_at	14	CSIG	AA928506	16p13.13
202329_at	14	CSK	NM_004383	15q23-q25
206562_s_at	15	CSNK1A1	NM_001892	5q32
208865_at	15	CSNK1A1	BG534245	5q32
208866_at	15	CSNK1A1	BF510713	5q32
213086_s_at	15	CSNK1A1	BF341845	---
213860_x_at	15	CSNK1A1	AW268585	5q32
208867_s_at	14	CSNK1A1	AF119911	---
207945_s_at	15	CSNK1D	NM_001893	17q25
208774_at	15	CSNK1D	AV700224	17q25
202332_at	15	CSNK1E	NM_001894	22q13.1
221673_s_at	12	CSNK1G1L	AB042563	15q22.1-q22.31
202573_at	15	CSNK1G2	AL530441	19p13.3
220768_s_at	14	CSNK1G3	NM_004384	5q23
206075_s_at	12	CSNK2A1	NM_001895	20p13
212072_s_at	15	CSNK2A1	AL049761	20p13
212073_at	12	CSNK2A1	AI631874	20p13
212075_s_at	15	CSNK2A1	AI161318	20p13
203575_at	15	CSNK2A2	NM_001896	16p13.3-p13.2
201390_s_at	15	CSNK2B	NM_001320	6p21-p12
211623_s_at	15	CSNK2B	M30448	---
204619_s_at	15	CSPG2	BF590263	5q14.3
204620_s_at	15	CSPG2	NM_004385	5q14.3
215646_s_at	14	CSPG2	R94644	5q14.3
221731_x_at	15	CSPG2	BF218922	5q14.3
209257_s_at	10	CSPG6	BF795297	10q25
209258_s_at	10	CSPG6	AI373676	10q25
202610_s_at	15	CSRP	AF135802	Xp11.4-p11.2
200621_at	15	CSRP1	NM_004078	1q32
207030_s_at	15	CSRP2	NM_001321	12q21.1
211126_s_at	15	CSRP2	U46006	12q21.1

205553_s_at	15	CSRP3	NM_003476	11p15.1
201360_at	15	CST3	NM_000099	20p11.21
201201_at	15	CSTB	NM_000100	21q22.3
204459_at	12	CSTF2	NM_001325	Xq22.1
212901_s_at	10	CSTF2T	BF732638	10q11
212905_at	14	CSTF2T	AI872408	10q11
203947_at	14	CSTF3	NM_001326	11p13
32723_at	14	CstF-50; CstFp50	L02547	20q13.31
210854_x_at	15	CT1; CRTR; MGC87396	U17986	Xq28
218898_at	15	CT120	NM_024792	17p13.3
203392_s_at	15	CTBP1	NM_001328	4p16
212863_x_at	15	CTBP1	BF337195	4p16
213979_s_at	13	CTBP1	BF984434	4p16
213980_s_at	15	CTBP1	AA053830	4p16
201218_at	15	CTBP2	N23018	10q26.2
201219_at	14	CTBP2	AW269836	10q26.2
201220_x_at	15	CTBP2	NM_001329	10q26.2
210554_s_at	15	CTBP2	BC002486	10q26.2
210835_s_at	15	CTBP2	AF222711	10q26.2
218923_at	10	CTBS	AW304174	1p22.3
218924_s_at	11	CTBS	AW304174	1p22
202521_at	15	CTCF	NM_006565	16q21-q22.3
217844_at	15	CTDSP1	NM_021198	2q35
203445_s_at	15	CTDSP2	NM_005730	12q13-q15
201904_s_at	12	CTDSPL	BF031714	3p21.3
201906_s_at	15	CTDSPL	NM_005808	3p21.3
221850_x_at	14	CTGLF1	AI826075	---
217127_at	13	CTH	AL354872	1p31.1
200764_s_at	15	CTNNA1	AI826881	5q31
200765_x_at	15	CTNNA1	NM_001903	5q31
202468_s_at	15	CTNNAL1	NM_003798	9q31.2
201533_at	15	CTNNB1	NM_001904	3p21 /// 3p21
203081_at	15	CTNNBIP1	NM_020248	1p36.22
221021_s_at	15	CTNNBL1	NM_030877	20q11.23-q12
208407_s_at	15	CTNND1	NM_001331	11q11
208862_s_at	15	CTNND1	AW073672	11q11
211240_x_at	15	CTNND1	AB002382	11q11
36566_at	12	CTNS	AJ222967	17p13
210010_s_at	15	CTP; SLC20A3	U25147	22q11.21
202613_at	15	CTPS	NM_001905	1p34.1
219080_s_at	15	CTPS2	NM_019857	Xp22
214377_s_at	11	CTRL	BF508685	1p32.3-p31.3
200838_at	15	CTSB	NM_001908	8p22
200839_s_at	15	CTSB	NM_001908	8p22
213274_s_at	15	CTSB	AA020826	8p22
213275_x_at	15	CTSB	W47179	8p22
201487_at	15	CTSC	NM_001814	11q14.1-q14.3
200766_at	12	CTSD	NM_001909	11p15.5
205927_s_at	10	CTSE	NM_001910	1q31
203657_s_at	15	CTSF	NM_003793	11q13

202295_s_at	15	CTSH	NM_004390	15q24-q25
202450_s_at	15	CTSK	NM_000396	1q21
202087_s_at	15	CTSL	NM_001912	9q21-q22
203758_at	15	CTSO	AV729484	4q31-q32
201059_at	15	CTTN	NM_005231	11q13
214074_s_at	12	CTTN	BG475299	11q13
219468_s_at	10	CUEDC1	NM_017949	17q23.2
218097_s_at	15	CUEDC2	NM_024040	10q24.32
209489_at	15	CUGBP1	N25915	11p11
221742_at	14	CUGBP1	AI472139	11p11
221743_at	15	CUGBP1	AI472139	11p11
202156_s_at	15	CUGBP2	N36839	10p13
202158_s_at	14	CUGBP2	N36839	10p13
207614_s_at	15	CUL1	NM_003592	7q36.1
203079_s_at	15	CUL2	NM_003591	10p11.22
201370_s_at	14	CUL3	AU145232	2q36.3
201371_s_at	15	CUL3	AU145232	2q36.3
201423_s_at	15	CUL4A	AL037208	13q34
201424_s_at	15	CUL4A	NM_003589	13q34
202213_s_at	13	CUL4B	AI650819	Xq23
202214_s_at	15	CUL4B	NM_003588	Xq23
210257_x_at	15	CUL4B	AF212995	Xq23
203531_at	15	CUL5	BF435809	11q22-q23
203533_s_at	13	CUL5	NM_003478	11q22-q23
221488_s_at	15	CUTA; MGC111154	AF230924	6pter-p21.31
218970_s_at	15	CUTC	NM_015960	10q24.31
202367_at	15	CUTL1	NM_001913	7q22.1
214743_at	15	CUTL1	BE046521	7q22.1
203917_at	15	CXADR	NM_001338	21q21.1
823_at	15	CXC3	U84487	16q13
203666_at	15	CXCL12	NM_000609	10q11.1
217028_at	15	CXCR4	AJ224869	---
204340_at	13	CXorf12	NM_003492	Xq28
220252_x_at	11	CXorf21	NM_025159	Xp21.3
213315_x_at	15	CXorf40	L43577	Xq28
214112_s_at	15	CXorf40	AA543076	Xq28
212961_x_at	15	CXorf40; EOLA1	L43578	Xq28
205583_s_at	15	CXorf45	NM_024810	Xq23
205584_at	12	CXorf45	NM_024810	Xq23
221196_x_at	11	CXorf53	NM_024332	Xq28
205088_at	14	CXorf6	NM_005491	Xq28
204923_at	15	CXorf9	AL023653	Xq26
201828_x_at	15	CXX1	NM_003928	Xq26
217948_at	14	CXX1	NM_015582	Xq26.3
48580_at	15	CXXC1	U55777	18q12
207843_x_at	15	CYB5	NM_001914	18q23
215726_s_at	15	CYB5	M22976	18q23
207986_x_at	15	CYB561	NM_001915	17q11-qter
210816_s_at	10	CYB561	BC000021	17q11-qter
201633_s_at	14	CYB5-M	AW235051	16q22.1

201634_s_at	13	CYB5-M	NM_030579	16q22.1
202263_at	15	CYB5R1	NM_016243	1p36.13-q41
201885_s_at	15	CYB5R3	NM_000398	22q13.31-qter
219079_at	14	CYB5R4	NM_016230	6pter-q22.33
203028_s_at	13	CYBA	NM_000101	16q24
203922_s_at	12	CYBB	AI308863	Xp21.1
203923_s_at	11	CYBB	AI308863	Xp21.1
210131_x_at	15	CYBL	D49737	1q21
201066_at	15	CYC1	NM_001916	8q24.3
208905_at	15	CYCS	BC005299	7p15.2
208923_at	15	CYFIP1	BC005097	15q11
215785_s_at	15	CYFIP2	AL161999	5q33.3
220999_s_at	12	CYFIP2	NM_030778	5q33.3 /// 5q33.3
213072_at	13	CYHR1	AI928387	8q24.3
213681_at	14	CYHR1	AW512817	---
213295_at	15	CYLD	AA555096	16q12-q13
39582_at	12	CYLD	AL050166	16q12-q13
207063_at	10	CYorf14	NM_018542	Yq11.222
208993_s_at	15	CYP; SRCyp; CARS-Cyp	U40763	2q31.1
207608_x_at	13	CYP1A2	NM_000761	15q22-qter
202435_s_at	10	CYP1B1	AU154504	2p21
202436_s_at	15	CYP1B1	AU144855	2p21
202437_s_at	15	CYP1B1	NM_000104	2p21
219565_at	10	CYP20A1	NM_020674	2q33.3
216025_x_at	13	CYP2C	M21940	10q24
214421_x_at	12	CYP2C9	AV652420	10q24
217558_at	10	CYP2C9	BE971373	10q24
205073_at	15	CYP2J2	NM_000775	1p31.3-p31.2
210502_s_at	15	CYP-33	AF042386	1p32
220432_s_at	12	CYP39A1	NM_016593	6p21.1-p11.2
202314_at	15	CYP51A1	NM_000786	7q21.2-q21.3
216607_s_at	15	CYP51P2	U40053	---
207386_at	10	CYP7B1	NM_004820	8q21.3
208928_at	12	CYPOR	AF258341	7q11.2
201289_at	15	CYR61	NM_001554	1p31-p22
210764_s_at	15	CYR61	AF003114	1p31-p22
212527_at	15	D15Wsu75e	BF057059	22q13.2
212528_at	10	D15Wsu75e	AI348009	---
212012_at	15	D2S448	BF342851	2p25
212013_at	15	D2S448	D86983	2p25
209569_x_at	15	D4S234E	BC001745	4p16.3
208812_x_at	15	D6S204	BC004489	6p21.3
216526_x_at	15	D6S204	AK024836	6p21.3
216060_s_at	15	DAAM1	AK021890	14q23.1
212793_at	14	DAAM2	BF513244	6p21.1
201278_at	15	DAB2	N21202	---
201279_s_at	13	DAB2	BC003064	5p13
201280_s_at	15	DAB2	NM_001343	5p13
210757_x_at	15	DAB2	AF188298	5p13
221519_at	15	DAC	AF281859	10q24

205471_s_at	15	DACH1	NM_004392	13q22
219179_at	15	DACT1	NM_016651	14q23.1
200046_at	15	DAD1	NM_001344	14q11-q12
201925_s_at	15	DAF	NM_000574	1q32
201926_s_at	15	DAF	BC001288	1q32
205417_s_at	15	DAG1	NM_004393	3p21
221934_s_at	15	DALRD3	BF941492	3p21.31
206878_at	10	DAO	NM_001917	12q24
201095_at	15	DAP	NM_004394	5p15.2
38703_at	15	DAP; ASPEP	AF005050	2q36.1
208822_s_at	15	DAP-3	U18321	1q21-q22
209323_at	15	DAP4	AF081567	11q13.5
203139_at	15	DAPK1	NM_004938	9q34.1
203890_s_at	12	DAPK3	BF686824	19p13.3
203891_s_at	15	DAPK3	NM_001348	19p13.3
201623_s_at	15	DARS	BC000629	2q22.1
201624_at	15	DARS	NM_001349	2q22.1
218365_s_at	13	DARS2	NM_018122	1q24.2
213213_at	15	DATF1	AL035669	---
201763_s_at	15	DAXX	NM_001350	6p21.3
218443_s_at	15	DAZAP1	NM_018959	19p13.3
200794_x_at	15	DAZAP2	NM_014764	2q33.2
212595_s_at	15	DAZAP2	AL534321	2q33.2
214334_x_at	15	DAZAP2	N34846	2q33.2
204244_s_at	12	DBF4	NM_006716	7q21.3
202428_x_at	15	DBI	NM_020548	2q12-q21
202806_at	15	DBN1	NM_004395	5q35.3
217025_s_at	15	DBN1	AL110225	5q35.3
201386_s_at	15	DBP1	AF279891	4p15.3
214988_s_at	15	DBP-5	X63071	21q22.1-q22.2
205370_x_at	15	DBT	NM_001918	1p31
201677_at	15	DC12	AI937543	3q21.3
201678_s_at	14	DC12	AI937543	3q21.3
218447_at	15	DC13	NM_020188	16q23.2
203299_s_at	15	DC22	AF251295	Xp22.31
221570_s_at	15	DC3	AF201938	2q31.1
209484_s_at	15	DC8	AF201941	1q41
205399_at	11	DCAMKL1	NM_004734	13q13-q14.1
222101_s_at	15	DCHS1	BF222893	11p15.4
209759_s_at	15	DCI	BC002746	16p13.3
203302_at	15	DCK	NM_000788	4q13.3-q21.1
219678_x_at	15	DCLRE1C	NM_022487	10p13
201894_s_at	15	DCN	NM_001920	12q13.2
209335_at	15	DCN	BC005322	12q13.2
218508_at	14	DCP1A	NM_018403	3p21.31
212919_at	15	DCP2	AV715578	5q22.3
218774_at	13	DCPS	NM_014026	11q24.2
208661_s_at	15	DCRR1	D84294	21q22.2
210645_s_at	15	DCRR1	D83077	21q22.2
203124_s_at	15	DCT1	AF046997	12q13

201571_s_at	15	DCTD	AI656493	4q35.1
201572_x_at	15	DCTD	NM_001921	4q35.1
210137_s_at	15	DCTD	BC001286	4q35.1
201082_s_at	15	DCTN1	NM_004082	2p13
211780_x_at	11	DCTN1	BC006163	2p13 /// 2p13
200932_s_at	15	DCTN2	NM_006400	12q13.2-q13.3
204246_s_at	15	DCTN3	NM_007234	9p13
218013_x_at	11	DCTN4	NM_016221	5q31-q32
209232_s_at	15	DCTN5	BC004191	16p12.3
203261_at	15	DCTN6	NM_006571	8p12-p11
218583_s_at	15	DCUN1D1	NM_020640	3q26.3
219116_s_at	15	DCUN1D2	NM_018185	13q34
217973_at	15	DCXR	NM_016286	17q25.3
211653_x_at	15	DD	M33376	10p15-p14
218260_at	13	DDA1	NM_024050	19p13.12
201896_s_at	15	DDA3	BC001425	1q21.2
209094_at	15	DDAH1	AL078459	1p22
202262_x_at	15	DDAH2	NM_013974	6p21.3
215537_x_at	15	DDAH2	AJ012008	6p21.3
203409_at	13	DDB2	NM_000107	11p12-p11
221039_s_at	13	DDEF1	NM_018482	8q24.1-q24.2
206414_s_at	15	DDEF2	NM_003887	2p25
209383_at	10	DDIT3	BC003637	12q13.2
202887_s_at	15	DDIT4	NM_019058	10pter-q26.12
208674_x_at	15	DDOST	D29643	1p36.1
208675_s_at	15	DDOST	D29643	1p36.1
207169_x_at	15	DDR1	NM_001954	6p21.3
202929_s_at	15	DDT	NM_001355	22q11.23
201241_at	15	DDX1	NM_004939	2p24
204977_at	15	DDX10	NM_004398	11q22-q23
208149_x_at	10	DDX11	NM_030653	12p11 /// 12p11
213378_s_at	11	DDX11	AI983033	12p13.31
208151_x_at	15	DDX17	NM_030881	22q13.1 /// 22q13.1
208718_at	15	DDX17	Z97056	22q13.1
213998_s_at	13	DDX17	AW188131	22q13.1
205763_s_at	15	DDX18	NM_006773	2q14.2
208895_s_at	15	DDX18	BG530850	2q14.2
208897_s_at	15	DDX18	BG530850	2q14.2
202577_s_at	13	DDX19A	BC005162	16q22.1
208152_s_at	15	DDX21	NM_004728	10q21 /// 10q21
200694_s_at	15	DDX24	NM_020414	14q32
200702_s_at	13	DDX24	BG421209	14q32
218819_at	14	DDX26	NM_012141	13q14.12-q14.2
222239_s_at	15	DDX26	AL117626	13q14.12-q14.2
219108_x_at	13	DDX27	NM_017895	20q13.13
40255_at	12	DDX28	AC004531	---
212648_at	15	DDX29	AL079292	5q11.2
212649_at	11	DDX29	AL079292	5q11.2
211787_s_at	15	DDX2A	BC006210	17p13 /// 17p13
212674_s_at	15	DDX30	AK002076	3p21.31

201584_s_at	15	DDX39	NM_005804	19p13.13
201210_at	15	DDX3X	NM_001356	Xp11.3-p11.23
212514_x_at	14	DDX3X	R60068	Xp11.3-p11.23
212515_s_at	15	DDX3X	BG492602	Xp11.3-p11.23
205000_at	10	DDX3Y	NM_004660	Yq11
217840_at	15	DDX41	NM_016222	5q35.3
201788_at	15	DDX42	NM_007372	17q24.2
220890_s_at	15	DDX47	NM_016355	12p13.2
201303_at	15	DDX48	NM_014740	17q25.3
31807_at	15	DDX49	AC002985	19p12
200033_at	15	DDX5	NM_004396	17q21
213637_at	14	DDX52	BE503392	---
219111_s_at	14	DDX54	NM_024072	12q24.21
217754_at	14	DDX56	NM_019082	7p13
202906_s_at	13	DECR; NADPH	AF049895	8q21
202447_at	15	DECR1	NM_001359	8q21.3
219664_s_at	14	DECR2	NM_020664	16p13.3
209165_at	14	DED; CHE1; CHE-1	AF083208	17q11.2-q12
202480_s_at	14	DEDD	NM_004216	1q23.1
205033_s_at	15	DEFA1	NM_004084	8p23.2-p23.1
207269_at	14	DEFA4	NM_001925	8p23
211255_x_at	10	DEFT	AF064605	1q23.1
215158_s_at	15	DEFT	AK022531	1q23.1
207431_s_at	15	DEGS1	NM_003676	1q42.12
209250_at	15	DEGS1	BC000961	1q42.12
200934_at	15	DEK	NM_003472	6p23
200047_s_at	15	DELTA	Z14077	14q
201901_s_at	15	DELTA	Z14077	14q
220295_x_at	13	DEPDC1	NM_017779	1p31.2
218858_at	11	DEPDC6	NM_022783	8q24.12
218102_at	15	DERA	NM_015954	12p13.1
218172_s_at	15	DERL1	NM_018630	8q24.13
219402_s_at	15	DERL1	NM_024295	8q24.13
218333_at	15	DERL2	NM_016041	---
219260_s_at	14	DERP6	NM_015362	17p13.2
202222_s_at	15	DES	NM_001927	2q35
214027_x_at	15	DES	AA889653	2q35
209146_at	15	DESP4	U93162	4q32-q34
219641_at	15	DET1	NM_017996	15q25.3
203733_at	15	DEXI	NM_014015	16p13.2
203277_at	15	DFFA	NM_004401	1p36.3-p36.2
203695_s_at	15	DFNA5	NM_004403	7p15
203669_s_at	15	DGAT1	NM_012079	8q24.3
214198_s_at	14	DGCR2	AU150824	22q11.21
208024_s_at	15	DGCR6	NM_005675	22q11.21
203385_at	15	DGKA	NM_001345	12q13.3
208072_s_at	15	DGKD	NM_003648	2q37.1
206806_at	15	DGKI	NM_004717	7q32.3-q33
207556_s_at	14	DGKZ	NM_003646	11p11.2
203816_at	13	DGUOK	NM_001929	2p13

209549_s_at	15	DGUOK	BC001121	2p13
200862_at	10	DHCR24	NM_014762	1p33-p31.1
201791_s_at	12	DHCR7	NM_001360	11q13.2-q13.5
202532_s_at	14	DHFR	NM_000791	5q11.2-q13.2
202534_x_at	15	DHFR	NM_000791	5q11.2-q13.2
48808_at	14	DHFR	AI144299	5q11.2-q13.2
209619_at	15	DHLAG	K01144	5q32
213632_at	12	DHOdehase	M94065	16q22
202802_at	15	DHPS	NM_001930	19p13.2-p13.1
207831_x_at	15	DHPS	NM_013407	19p13.2-p13.1
213279_at	15	DHRS1	AL050217	14q11.2
202481_at	15	DHRS3	NM_004753	1p36.1
218021_at	15	DHRS4	NM_021004	14q11.2
218285_s_at	15	DHRS6	NM_020139	4q24
217989_at	15	DHRS8	NM_016245	4q22.1
209916_at	12	DHTKD1	BC002477	10p14
201385_at	15	DHX15	NM_001358	4p15.3
203694_s_at	15	DHX16	NM_003587	6p21.3
204355_at	14	DHX30	NM_014966	3p21.31
218198_at	15	DHX32	NM_018180	10q26.2
218277_s_at	15	DHX40	BF431360	17q23.2
202420_s_at	15	DHX9	NM_001357	1q25
219350_s_at	15	DIABLO	NM_019887	12q24.31
205603_s_at	12	DIAPH2	NM_007309	Xq22
205726_at	15	DIAPH2	NM_006729	Xq22
206061_s_at	15	DICER1	NM_030621	14q32.2
212888_at	14	DICER1	BG109746	14q32.2
213229_at	15	DICER1	BF590131	14q32.2
221471_at	15	Diff33	U49188	20q13.1-13.3
221472_at	15	Diff33	U49188	20q13.1-13.3
221473_x_at	15	Diff33	U49188	20q13.1-13.3
210052_s_at	10	DIL2	AF098158	20q11.2
218218_at	15	DIP13B	NM_018171	12q24.1
204610_s_at	15	DIPA	NM_006848	11q12.1
221579_s_at	15	DIPP; DIPP1	AF062530	6p21.2
206090_s_at	15	DISC1	NM_018662	1q42.1
214724_at	10	DIXDC1	AF070621	---
221311_x_at	13	DJ122O8.2	NM_020466	6q14.2-q16.1
205002_at	14	DJ159A19.3	NM_015699	1p36.13
217256_x_at	15	dJ507I15.1	Z98950	---
201580_s_at	15	DJ971N18.2	AL544094	20p12
201581_at	15	DJ971N18.2	AL544094	20p12
201479_at	15	DKC1	NM_001363	Xq28
221541_at	15	DKFZP434B044	AL136861	16q24.1
212886_at	15	DKFZP434C171	AL080169	5q33.1
212804_s_at	15	DKFZP434C212	AI797397	9q34.11
212132_at	15	DKFZP434D1335	AL117499	19q13.12
221245_s_at	14	DKFZP434E2135	NM_030804	---
221535_at	15	DKFZp434E248	AL136897	3q29
221536_s_at	15	DKFZp434E248	AL136897	3q29

221031_s_at	15	DKFZP434F0318	NM_030817	12p13.2
214679_x_at	15	DKFZp434J194	AL110227	19p13.3
212228_s_at	15	DKFZP434K046	AC004382	16q13
221522_at	15	DKFZP434L0718	AL136784	19q13.12
219872_at	10	DKFZp434L142	NM_016613	4q32.1
213701_at	15	DKFZp434N2030	AW299245	12q21.33
212505_s_at	14	DKFZp434P101	AL110250	19p13.11
213737_x_at	15	DKFZp434P162	AI620911	---
212300_at	12	DKFZp451J0118	AL049795	1p34.3
213657_s_at	13	DKFZp547K1113	BE858194	15q26.1
213658_at	15	DKFZp547K1113	BE858194	15q26.1
37590_g_at	14	DKFZp547K1113	AL109698	15q26.1
216028_at	13	DKFZP564C152	AL049980	11
52078_at	15	DKFZP564D0478	AI828080	1p35.3
212123_at	15	DKFZP564D116	AL050022	10q24.1
212936_at	15	DKFZP564D172	AI927701	5q15
212333_at	15	DKFZp564F0522	AL049943	2p23.1
212202_s_at	15	DKFZP564G2022	BG493972	15q14
212204_at	15	DKFZp564G2022	AL049944	15q14
212893_at	14	DKFZp564I052	AL080063	1p31.1
209596_at	15	DKFZp564I1922	AF245505	Xp22.33
209177_at	15	DKFZP564J0123	BC002873	3p21.31
217794_at	11	DKFZP564J157	NM_018457	12q12
221553_at	12	DKFZp564K142	AL136636	Xq13.3
210596_at	12	DKFZp564K142	AF130104	Xq13.3
204218_at	15	DKFZP564M082	NM_014042	11q13.3
212018_s_at	15	DKFZP564M182	AK025446	16p13.13
221596_s_at	14	DKFZp564O0523	AL136619	7q21.3
204687_at	15	DKFZP564O0823	NM_015393	4q13.3-q21.3
212002_at	14	DKFZp566C0424	BG171020	1p36.13
212004_at	15	DKFZp566C0424	AL050028	1p36.13
215172_at	13	DKFZP566K0524	AL050040	10q11.22
217713_x_at	15	DKFZP566N034	AA126763	---
207761_s_at	15	DKFZP586A0522	NM_014033	12q13.13
209703_x_at	14	DKFZP586A0522	BC004492	12q13.13
213861_s_at	15	DKFZP586D0919	N67741	12q13.2
221636_s_at	15	DKFZp586G2122	AL136931	1q42.11
209760_at	11	DKFZp586H1322	AL136932	4q31.3
213661_at	12	DKFZP586H2123	AI671186	11p13
213546_at	11	DKFZp586I1420	AL050378	7p15.1
212212_s_at	15	DKFZP586J0619	BF055496	7p22.3
221970_s_at	15	DKFZP586L0724	AU158148	17q24.3
36552_at	12	DKFZp586P0123	AL080220	11q13.3
213189_at	15	DKFZp667G2110	AI823896	---
54970_at	14	DKFZp761I2123	AA868332	7p13
204602_at	12	DKK1	NM_012242	10q11.2
219908_at	12	DKK2	NM_014421	4q25
202196_s_at	15	DKK3	NM_013253	11p15.2
214247_s_at	15	DKK3	AU148057	11p15.2
211150_s_at	13	DLAT	J03866	---

212568_s_at	15	DLAT	BF978872	11q23.1
213149_at	15	DLAT	AW299740	11q23.1
220511_s_at	13	DLC1	NM_024767	8p22
220512_at	11	DLC1	NM_024767	8p22
205677_s_at	15	DLEU1	NM_005887	13q14.3
202516_s_at	10	DLG1	NM_004087	3q29
212729_at	15	DLG3	AI916274	Xq13.1
201681_s_at	15	DLG5	AB011155	10q23
203764_at	15	DLG7	NM_014750	14q22.2
202572_s_at	15	DLGAP4	NM_014902	20q11.23
209560_s_at	15	dlk	U15979	14q32
217066_s_at	12	DM	M87313	19q13.3
208250_s_at	10	DMBT1	NM_004406	10q25.3-q26.1
208386_x_at	15	DMC1	NM_007068	22q13.1
203881_s_at	15	DMD	NM_004010	Xp21.2
207660_at	12	DMD	NM_004019	Xp21.2
208086_s_at	11	DMD	M92650	Xp21.2
37996_s_at	15	DMPK	L08835	19q13.3
203301_s_at	15	DMTF1	NM_021145	7q21
33768_at	15	DMWD	L19267	19q13.3
203791_at	15	DMXL1	NM_005509	5q22
220725_x_at	10	DNAH3	NM_025095	16p13.11-p12.3
200880_at	15	DNAJA1	AL534104	9p13-p12
200881_s_at	15	DNAJA1	AL534104	9p13-p12
205963_s_at	15	DNAJA3	NM_005147	16p13.3
200664_s_at	14	DNAJB1	NM_006145	19p13.2
200666_s_at	15	DNAJB1	NM_006145	19p13.2
202867_s_at	15	DNAJB12	NM_017626	10q22.2
219237_s_at	12	DNAJB14	NM_024920	4q23
202500_at	15	DNAJB2	NM_006736	2q32-q34
203810_at	15	DNAJB4	BG252490	1p31.1
203811_s_at	14	DNAJB4	NM_007034	1p31.1
209015_s_at	15	DNAJB6	BC002446	7q36.3
202842_s_at	15	DNAJB9	AL080081	7q31
202843_at	14	DNAJB9	NM_012328	7q31
218409_s_at	11	DNAJC1	NM_022365	10p12.33-p12.32
221781_s_at	12	DNAJC10	BG168666	2q32.1
221782_at	15	DNAJC10	BG168666	2q32.1
218435_at	15	DNAJC15	NM_013238	13q14.1
206782_s_at	14	DNAJC4	NM_005528	11q13
213919_at	13	DNAJC4	AW024467	11q13
204720_s_at	15	DNAJC6	AV729634	1pter-q31.3
202416_at	15	DNAJC7	NM_003315	17q11.2
205545_x_at	15	DNAJC8	NM_014280	1p35.2
212491_s_at	15	DNAJC8	AA843895	1p35.2
213088_s_at	15	DNAJC9	BE551340	10q22.3
204008_at	15	DNAL4	NM_005740	22q13.1
208694_at	15	DNA-PKcs	U47077	8q11
222154_s_at	15	DNAPTP6	AK002064	2q33.2
203912_s_at	14	DNASE1L1	NM_006730	Xq28

211928_at	15	DNCH1	AB002323	14q32.3-qter
219469_at	10	DNCH2	NM_024606	11q21-q22.1
57739_at	14	DND1	AI949010	5q31.3
209157_at	15	DNJ3/CPR3	AF011793	16q11.1-q11.2
203105_s_at	15	DNM1L	NM_012062	12p11.21
201697_s_at	12	DNMT1	NM_001379	19p13.2
201937_s_at	12	DNPEP	NM_012100	2q36.1
217604_at	15	DNPEP	AI086530	---
202776_at	15	DNTTIP2	NM_014597	1p22.1
204135_at	15	DOC1	NM_014890	3q12.3
203187_at	15	DOCK1	NM_001380	10q26.13-q26.3
205003_at	15	DOCK4	NM_014705	7q31.1
221794_at	15	DOCK6	AI198543	19p13.2
55583_at	14	DOCK6	AI198543	19p13.2
212538_at	15	DOCK9	AL576253	13q32.3
214263_x_at	15	DOK4	AI192781	16q13
214844_s_at	15	DOK5	AL050069	20q13.2
221677_s_at	15	DONSON	AF232674	21q22.1
209509_s_at	15	DPAGT1	BC000325	11q23.3
211302_s_at	15	DPDE4	L20966	1p31
202116_at	15	DPF2	NM_006268	11q13
219746_at	15	DPF3	NM_012074	14q24.3-q31.1
202632_at	14	DPH1	NM_001383	--- /// ---
222041_at	15	DPH2L1	BG235929	---
213853_at	15	Dph4	AL050199	---
219590_x_at	15	DPH5	NM_015958	1p21.2
202673_at	15	DPM1	NM_003859	20q13.13
219373_at	15	DPM3	NM_018973	1q22
220939_s_at	15	DPP8	NM_017743	15q22
207977_s_at	15	DPT	NM_001937	1q12-q23
213068_at	15	DPT	AI146848	1q12-q23
213071_at	15	DPT	AL049798	1q12-q23
204646_at	15	DPYD	NM_000110	1p22
200762_at	15	DPYSL2	NM_001386	8p22-p21
201431_s_at	15	DPYSL3	NM_001387	5q32
205493_s_at	15	DPYSL4	NM_006426	10q26
207654_x_at	15	DR1	NM_001938	1p22.1
209187_at	15	DR1	AW516932	1p22.1
209188_x_at	15	DR1	AW516932	1p22.1
216652_s_at	15	DR1	AL137673	1p22.1
209295_at	15	DR5	AF016266	8p22-p21
203258_at	15	DRAP1	NM_006442	11q13.3
208931_s_at	15	DRBF	AF147209	19p13.2
211375_s_at	15	DRBF	AF141870	19p13.2
206590_x_at	12	DRD2	NM_016574	11q23
208486_at	14	DRD5	NM_000798	4p16.1
221985_at	15	DRE1	AW006750	3q27.3
221986_s_at	15	DRE1	AW006750	3q27.3
217868_s_at	15	DREV1	BC000195	16p13-p12
202810_at	15	DRG1	NM_004147	22q12.2 /// 22q12.2

221517_s_at	15	DRIP80	AF105421	11q14
221643_s_at	12	DRPLA	AF016005	1p36.1-p36.2
204751_x_at	12	DSC2	NM_004949	18q12.1
206032_at	13	DSC3	AI797281	18q12.1
206033_s_at	10	DSC3	NM_001941	18q12.1
208370_s_at	15	DSCR1	NM_004414	21q22.1-q22.2
215253_s_at	11	DSCR1	AL049369	21q22.1-q22.2
203498_at	15	DSCR1L1	NM_005822	6p21.1
203405_at	15	DSCR2	NM_003720	21q22.3
203635_at	15	DSCR3	NM_006052	21q22.2
221689_s_at	15	DSCR5b	AB035745	21q22.2
217901_at	15	DSG2	NM_001943	18q12.1
208763_s_at	15	DSIPI	AL110191	Xq22.3
200606_at	15	DSP	NM_004415	6p24
212253_x_at	12	DST	BG253119	6p12-p11
212254_s_at	15	DST	AI798790	6p12-p11
215016_x_at	15	DST	BC004912	6p12-p11
201021_s_at	15	DSTN	BF697964	20p11.23
201022_s_at	15	DSTN	BF697964	20p11.23
213079_at	15	DT1P1A10	AA223871	Xp11.22
205741_s_at	15	DTNA	NM_001392	18q12
210611_s_at	13	DTNA	U26744	18q12
38037_at	15	DTR	M60278	5q23
200035_at	15	DULLARD	NM_015343	17p13
217912_at	14	DUS1L	NM_022156	17q25.3
209457_at	15	DUSP; HVH3	U16996	10q25
201041_s_at	15	DUSP1	NM_004417	5q34
221563_at	14	DUSP10	N36770	1q41
202703_at	15	DUSP11	NM_003584	2p13.1
218576_s_at	15	DUSP12	NM_007240	1q21-q22
219963_at	13	DUSP13	NM_016364	10q22.3
203367_at	15	DUSP14	NM_007026	17q12
218845_at	15	DUSP22	NM_020185	6p25.3
219144_at	15	DUSP26	NM_024025	8p12
201536_at	15	DUSP3	AL048503	17q21
201537_s_at	15	DUSP3	AL048503	17q21
201538_s_at	14	DUSP3	AL048503	17q21
208891_at	15	DUSP6	BC003143	12q22-q23
208892_s_at	15	DUSP6	BC003143	12q22-q23
213848_at	15	DUSP7	AI655015	3p21
206374_at	15	DUSP8	NM_004420	11p15.5
208955_at	15	DUT	AB049113	15q15-q21.1
208956_x_at	15	DUT	AB049113	15q15-q21.1
209932_s_at	15	DUT	U90223	15q15-q21.1
203230_at	15	DVL1	AF006011	1p36
57532_at	15	DVL2	AW016304	17p13.2
201908_at	15	DVL3	NM_004423	3q27
209821_at	15	DVS27	AB024518	9p24.1
219061_s_at	15	DXS9879E	NM_006014	Xq28
222338_x_at	14	DXS9879E	AI375965	9p13.3

203624_at	14	DXYS155E	NM_005088	--- /// ---
217976_s_at	15	DYNC1LI1	NM_016141	3p22.3
203590_at	15	DYNC1LI2	NM_006141	16q22.1
203762_s_at	10	DYNC2LI1	NM_016008	2p25.1-p24.1
203763_at	15	DYNC2LI1	NM_016008	2p25.1-p24.1
200703_at	15	DYNLL1	NM_003746	12q24.23
217917_s_at	15	DYNLRB1	NM_014183	20q11.21
217918_at	15	DYNLRB1	NM_014183	20q11.21
201999_s_at	15	DYNLT1	NM_006519	6q25.2-q25.3
203303_at	15	DYNLT3	NM_006520	Xp21
209033_s_at	15	DYRK1A	D86550	21q22.13
202968_s_at	15	DYRK2	Y09216	12q14.3
202969_at	15	DYRK2	AI216690	12q14.3
202970_at	13	DYRK2	AI192838	12q14.3
202971_s_at	10	DYRK2	Y09216	12q14.3
212954_at	15	DYRK4	AF263541	12p13.32
204556_s_at	13	DZIP1	AL568422	13q32.1
204557_s_at	11	DZIP1	NM_014934	13q32.1
207231_at	14	DZIP3	NM_014648	3q13.13
213186_at	15	DZIP3	BG502305	3q13.13
222146_s_at	15	E2-2	AK026674	18q21.1
205371_s_at	12	E2B	M27093	1p31
203692_s_at	10	E2F3	AI640363	6p22
203693_s_at	13	E2F3	NM_001949	6p22
203957_at	13	E2F6	NM_001952	2p25.1
219990_at	14	E2F8	NM_024680	11p15.1
215210_s_at	15	E2k	S72422	14q24.3
209095_at	15	E3; LAD; DLDH; PHE3	J03620	7q31-q32
209478_at	15	E3; MGC14480	U95006	17q25.3
209028_s_at	15	E3B1	AF006516	10p11.2
215533_s_at	11	E4	AF091093	1p36.3
208833_s_at	15	E46L; SCA10; FLJ37990	AF119662	22q13.31
218524_at	12	E4F1	NM_004424	16p13.3
208768_x_at	15	EAP; HBP15; HBP15/L22	D17652	1p36.3-p36.2
204274_at	15	EBAG9	AA812215	8q23
204278_s_at	13	EBAG9	NM_004215	8q23
220392_at	15	EBF2	NM_022659	8p21.2
205419_at	13	EBI2	NM_004951	13q32.3
201323_at	15	EBNA1BP2	NM_006824	1p35-p33
213787_s_at	15	EBP	AV702405	Xp11.23-p11.22
208676_s_at	15	EBP1	U87954	12q13
221475_s_at	15	EC45	AF279903	3p24.1
221476_s_at	15	EC45	AF279903	3p24.1
201749_at	15	ECE1	BF969352	---
216449_x_at	15	ECGP; GP96; GRP94	AK025862	12q24.2-q24.3
200789_at	15	ECH1	NM_001398	19q13.1
219974_x_at	15	ECHDC1	NM_018479	6q22.33
218552_at	15	ECHDC2	NM_018281	1p32.3
219298_at	15	ECHDC3	NM_024693	10p14
201135_at	15	ECHS1	NM_004092	10q26.2-q26.3

209365_s_at	15	ECM1	U65932	1q21
206101_at	15	ECM2	NM_001393	9q22.3
208091_s_at	15	ECOP	NM_030796	7p11.2 /// 7p11.2
219787_s_at	14	ECT2	NM_018098	3q26.1-q26.2
206217_at	13	EDA	NM_001399	Xq12-q13.1
208882_s_at	15	EDD	U69567	8q22
208883_at	15	EDD	BF515424	---
208884_s_at	15	EDD	AF006010	8q22
203279_at	10	EDEM1	NM_014674	3p26.1
204642_at	15	EDG1	NM_001400	1p21
204036_at	14	EDG2	AW269335	9q32
204037_at	15	EDG2	BF055366	9q32
204038_s_at	13	EDG2	NM_001401	9q32
204463_s_at	15	EDNRA	NM_001957	4q31.22
204464_s_at	15	EDNRA	NM_001957	4q31.22
204271_s_at	15	EDNRB	NM_000115	13q22
204273_at	15	EDNRB	NM_000115	13q22
206701_x_at	15	EDNRB	NM_003991	13q22
213410_at	15	EDRF1	AL050102	10q26.2
204892_x_at	15	EEF1A1	NM_001402	6q14.1
206559_x_at	15	EEF1A1	NM_001403	---
213477_x_at	15	EEF1A1	AL515273	6q14.1
213583_x_at	15	EEF1A1	BE964125	6q14.1
213614_x_at	15	EEF1A1	BE786672	---
204540_at	15	EEF1A2	NM_001958	20q13.3
200705_s_at	15	EEF1B2	NM_001959	2q33-q34
203113_s_at	15	EEF1D	NM_001960	8q24.3
214394_x_at	15	EEF1D	AI613383	8q24.3
204905_s_at	15	EEF1E1	NM_004280	6p24.3-p25.1
200689_x_at	15	EEF1G	NM_001404	11q12.3
211345_x_at	15	EEF1G	AF119850	---
200094_s_at	15	EEF2	AI004246	---
204102_s_at	15	EEF2	NM_001961	19pter-q12
212400_at	15	EEIG1	AL043266	---
211200_s_at	12	EFCAB2	BC002836	---
201842_s_at	15	EFEMP1	AI826799	2p16
201843_s_at	12	EFEMP1	NM_004105	2p16
206580_s_at	15	EFEMP2	NM_016938	11q13
212410_at	15	EFHA1	AI346431	13q12.11
219833_s_at	15	EFHC1	NM_018100	6p12.3
220591_s_at	15	EFHC2	NM_025184	Xp11.4
217992_s_at	13	EFHD2	AW664179	1p36.13
202023_at	15	EFNA1	NM_004428	1q21-q22
202668_at	15	EFNB2	BF001670	13q33
205031_at	13	EFNB3	NM_001406	17p13.1-p11.2
204400_at	15	EFS	NM_005864	14q11.2-q12
212656_at	14	EF-TS; EF-Tsmt	AF110399	12q13.2
218973_at	15	EFTUD1	NM_024580	15q25.1
206254_at	15	EGF	NM_001963	4q25
212830_at	14	EGFL5	W68084	9q32-q33.3

219454_at	11	EGFL6	NM_015507	Xp22
218825_at	15	EGFL7	NM_016215	9q34.3
208469_s_at	14	EGFL8	NM_030652	6p21.3
211551_at	10	EGFR	K03193	7p12
221497_x_at	15	EGLN1	BC005369	1q42.1
220956_s_at	15	EGLN2	NM_017555	19q13.2
219232_s_at	15	EGLN3	NM_022073	14q13.1
201693_s_at	14	EGR1	NM_001964	5q31.1
201694_s_at	15	EGR1	NM_001964	5q31.1
206115_at	10	EGR3	NM_004430	8p23-p21
91703_at	11	EHBP1L1	AA149545	11q13.1
208112_x_at	15	EHD1	NM_006795	11q13
45297_at	14	EHD2	AI417917	19q13.3
218935_at	15	EHD3	NM_014600	2p21
209536_s_at	15	EHD4	AF320070	15q11.1
208289_s_at	14	EI24	NM_004879	11q24
216396_s_at	15	EI24	AF131850	11q24
208669_s_at	15	EID1	AF109873	15q21.1-q21.2
208670_s_at	12	EID1	AF109873	15q21.1-q21.2
211698_at	15	EID1	AF349444	15q21.1-q21.2
201016_at	15	EIF1AX	BE542684	Xp22.13
201017_at	15	EIF1AX	BG149698	Xp22.13
201018_at	15	EIF1AX	BE542684	Xp22.13
201019_s_at	15	EIF1AX	NM_001412	Xp22.13
204409_s_at	10	EIF1AY	BC005248	Yq11.222
204410_at	10	EIF1AY	NM_004681	Yq11.222
217736_s_at	15	EIF2AK1	AW007368	7p22
204211_x_at	14	EIF2AK2	NM_002759	2p22-p21
218696_at	15	EIF2AK3	NM_004836	2p12
209429_x_at	15	EIF2B	AF112207	2p23.3
201632_at	14	EIF2B1	NM_001414	12q24.31
202461_at	15	EIF2B2	NM_014239	14q24.3
218488_at	15	EIF2B3	NM_020365	1p34.1
215482_s_at	15	EIF2B4	AJ011307	2p23.3
218287_s_at	14	EIF2C1	NM_012199	1p35-p34
213310_at	13	EIF2C2	AI613483	8q24
219426_at	14	EIF2C3	NM_024852	1p34.3
219190_s_at	10	EIF2C4	NM_017629	1p34.3
201142_at	15	EIF2S1	BC002513	14q24.1
201143_s_at	14	EIF2S1	BC002513	14q24.1
201144_s_at	15	EIF2S1	NM_004094	14q24.1
208726_s_at	15	EIF2S2	BC000461	20pter-q12
208688_x_at	15	eIF3	U78525	7p22.3
210501_x_at	15	eIF3k	AF119846	19q13.2
208264_s_at	15	EIF3S1	NM_003758	15q15.1
208985_s_at	15	EIF3S1	BC002719	15q15.1
217364_x_at	11	EIF3S1	AL031313	---
200595_s_at	15	EIF3S10	NM_003750	10q26
200596_s_at	15	EIF3S10	BE614908	10q26
212716_s_at	15	EIF3S12	AW083133	19q13.2

201592_at	15	EIF3S3	NM_003756	8q24.11
214328_s_at	15	EIF3S3	R01140	---
208887_at	15	EIF3S4	BC000733	19p13.2
200023_s_at	15	EIF3S5	AI001896	11p15.4
208697_s_at	15	EIF3S6	BC000734	8q22-q23
217719_at	15	EIF3S6IP	NM_016091	22q
200005_at	15	EIF3S7	NM_003753	22q13.1
200647_x_at	15	EIF3S8	NM_003752	16p11.2
210949_s_at	15	EIF3S8	BC000533	16p11.2
215230_x_at	15	EIF3S8	AA679705	16p11.2
203462_x_at	15	EIF3S9	NM_003751	7p22.3
211501_s_at	14	EIF3S9	BC001173	7p22.3
201530_x_at	15	EIF4A1	NM_001416	17p13
214805_at	15	EIF4A1	U79273	17p13
200912_s_at	15	EIF4A2	NM_001967	3q28
211937_at	15	EIF4B	NM_001417	12q13.13
211938_at	15	EIF4B	BF247371	12q13.13
201435_s_at	15	EIF4E	AW268640	4q21-q25
201436_at	14	EIF4E	AI742789	4q21-q25
201437_s_at	14	EIF4E	NM_001968	4q21-q25
213571_s_at	15	EIF4E2	BF516289	2q37.1
208770_s_at	15	EIF4EBP2	BC005057	10q21-q22
209393_s_at	11	EIF4EL3	AF047695	2q37.1
218626_at	15	EIF4ENIF1	NM_019843	22q11.2
208624_s_at	13	EIF4G1	BE966878	3q27-qter
208625_s_at	15	EIF4G1	BE966878	3q27-qter
200004_at	15	EIF4G2	NM_001418	11p15
201935_s_at	15	EIF4G3	AI768122	1p36.12
201936_s_at	10	EIF4G3	NM_003760	1p36.12
208290_s_at	15	EIF5	NM_001969	14q32.33
208705_s_at	15	EIF5	BG481972	14q32.33
208708_x_at	15	EIF5	AL080102	14q32.33
201122_x_at	15	EIF5A	BC000751	17p13-p12
201123_s_at	15	EIF5A	NM_001970	17p13-p12
213757_at	15	EIF5A	AA393940	17p13-p12
208706_s_at	15	EIF-5A	AK026933	14q32.33
201024_x_at	14	EIF5B	NM_015904	2p11.1-q11.1
201027_s_at	15	EIF5B	NM_015904	2p11.1-q11.1
214314_s_at	12	EIF5B	BE138647	2p11.1-q11.1
210213_s_at	15	eIF6	AF022229	20q12
210504_at	14	EKLF	U65404	19p13.13-p13.12
201767_s_at	13	ELAC2	NM_018127	17p11.2
201726_at	15	ELAVL1	BC003376	19p13.2
201727_s_at	13	ELAVL1	NM_001419	19p13.2
203822_s_at	15	ELF2	NM_006874	4q28
210361_s_at	15	ELF2	AF256223	4q28
212680_x_at	15	ELF2	BE305165	11q13
203617_x_at	10	ELK1	NM_005229	Xp11.2
221773_at	15	ELK3	AW575374	12q23
215606_s_at	14	ELKS	AB029004	12p13.3

204513_s_at	12	ELMO1	NM_014800	7p14.1
55692_at	15	ELMO2	W22924	20q13
212670_at	15	ELN	AA479278	7q11.23
57163_at	15	ELOVL1	H93026	1p34.1
208788_at	15	ELOVL5	AL136939	6p21.1-p12.1
215082_at	12	ELOVL5	BF973387	6p21.1-p12.1
204256_at	15	ELOVL6	NM_024090	4q25
221094_s_at	11	ELP3	NM_018091	8p21.1
203829_at	15	ELP4	NM_019040	11p13
219134_at	15	ELTD1	NM_022159	1p33-p32
219436_s_at	15	EMCN; EMCN2	AF205940	4q23
209477_at	15	EMD	BC000738	Xq28
204163_at	10	EMILIN1	NM_007046	2p23.3-p23.2
204796_at	15	EML1	NM_004434	14q32
204797_s_at	15	EML1	NM_004434	14q32
204398_s_at	14	EML2	NM_012155	19q13.32
213063_at	15	EML5	BF970253	14q31.3
201324_at	15	EMP1	NM_001423	12p12.3
201325_s_at	15	EMP1	NM_001423	12p12.3
213895_at	12	EMP1	BF445047	12p12.3
204975_at	15	EMP2	NM_001424	16p13.2
203729_at	15	EMP3	NM_001425	19q13.3
211339_s_at	15	EMT	D13720	5q31-q32
217820_s_at	15	ENAH	NM_018212	1q42.13
201341_at	13	ENC1	NM_003633	5q12-q13.3
204824_at	14	ENDOG	NM_004435	9q34.1
205521_at	14	ENDOGL1	BF511976	3p21.3
201809_s_at	15	ENG	NM_000118	9q33-q34.1
201231_s_at	15	ENO1	NM_001428	1p36.3-p36.2
217294_s_at	15	ENO1L1	U88968	1p36.3-p36.2
201313_at	15	ENO2	NM_001975	12p13
204483_at	15	ENO3	NM_001976	17pter-p11
204142_at	15	ENOSF1	NM_017512	18p11.32
204143_s_at	15	ENOSF1	NM_017512	18p11.32
204845_s_at	15	ENPEP	NM_001977	4q25
204160_s_at	15	ENPP4	NM_014936	6p21.1
204161_s_at	11	ENPP4	NM_014936	6p21.1
202596_at	15	ENSA	BC000436	1q21.3
221486_at	15	ENSA	AF067170	1q21.3
221487_s_at	11	ENSA	AF067170	1q21.3
204717_s_at	13	ENT2	AF034102	11q13
201768_s_at	15	ENTH	BC004467	5q23.1-q33.3
201769_at	15	ENTH	NM_014666	5q23.1-q33.3
207691_x_at	13	ENTPD1	NM_001776	10q24
209473_at	15	ENTPD1	AV717590	10q24
209474_s_at	10	ENTPD1	AV717590	10q24
219731_at	15	ENTPD1	NM_024343	10q24
201704_at	13	ENTPD6	NM_001247	20p11.2-p11.22
218482_at	15	ENY2	NM_020189	8q23.2
210374_x_at	14	EP3	D38300	1p31.2

202221_s_at	15	EP300	AV727101	22q13.2
213579_s_at	15	EP300	AI459462	22q13.2
212375_at	11	EP400	BE880591	12q24.33
212336_at	14	EPB41L1	AL121895	20q11.2-q12
201718_s_at	14	EPB41L2	BF511685	6q23
201719_s_at	15	EPB41L2	NM_001431	6q23
206710_s_at	15	EPB41L3	NM_012307	18p11.32
211776_s_at	15	EPB41L3	BC006141	18p11.32 /// 18p11.32
212681_at	14	EPB41L3	AI770004	18p11.32
204505_s_at	15	EPB49	NM_001978	8p21.1
216437_at	12	EPC1	AK024949	---
203499_at	14	EPHA2	NM_004431	1p36
202894_at	15	EPHB4	NM_004444	7q22
216680_s_at	14	EPHB4	BC004264	7q22
204718_at	13	EPHB6	NM_004445	7q33-q35
202017_at	12	EPHX1	NM_000120	1q42.1
213434_at	15	EPIM	H95263	12q24.33
202669_s_at	15	EPLG5	U16797	13q33
217892_s_at	15	EPLIN	BC001247	12q13
205231_s_at	15	EPM2A	NM_005670	6q24
202909_at	15	EPM2AIP1	NM_014805	3p22.1
203464_s_at	15	EPN2	NM_014964	17p11.2
209962_at	10	EPOR	M34986	19p13.3-p13.2
209963_s_at	15	EPOR	M34986	19p13.3-p13.2
215054_at	14	EPOR	H16758	19p13.3-p13.2
37986_at	10	EPOR	M60459	19p13.3-p13.2
210534_s_at	15	EPPB9	BC002944	17p11.2
200842_s_at	15	EPRS	AI475965	1q41-q42
200843_s_at	15	EPRS	NM_004446	1q41-q42
217886_at	15	EPS15	BF213575	1p32
217887_s_at	15	EPS15	BF213575	1p32
222113_s_at	15	EPS15L1	AV710549	19p13.12
202609_at	15	EPS8	NM_004447	12q23-q24
217053_x_at	13	ER81	X87175	7p22
219672_at	15	ERAF	NM_016633	16p11.2
212087_s_at	15	ERAL1	AL562733	17q11.2
217941_s_at	15	ERBB2IP	NM_018695	5q13.1
202454_s_at	15	ERBB3	NM_001982	12q13
214053_at	15	ERBB4	AW772192	---
203719_at	15	ERCC1	NM_001983	19q13.2-q13.3
203720_s_at	15	ERCC1	NM_001983	19q13.2-q13.3
202176_at	12	ERCC3	NM_000122	2q21
202414_at	13	ERCC5	NM_000123	13q22
207347_at	15	ERCC6	NM_000124	10q11
205162_at	10	ERCC8	NM_000082	5q12.1
203643_at	15	ERF	NM_006494	19q13
213541_s_at	15	ERG	AI351043	21q22.3
209218_at	15	ERG1	AF098865	8q24.1
200043_at	15	ERH	NM_004450	14q24.1

212046_x_at	13	ERK1	X60188	16p12-p11.2
218498_s_at	13	ERO1L	NM_014584	14q22.1
201216_at	15	ERP29	NM_006817	12q24.13
216032_s_at	15	Erv46	AF091085	20pter-q12
32032_at	15	ES2	L77566	22q11.21
209009_at	15	ESD	BC001169	13q14.1-q14.2
215095_at	11	ESD	AU145746	13q14.1-q14.2
215096_s_at	15	ESD	AU145746	13q14.1-q14.2
208394_x_at	10	ESM1	NM_007036	5q11.2
38158_at	13	ESPL1	D79987	8
203193_at	14	ESRRA	NM_004451	11q13
207981_s_at	15	ESRRG	NM_001438	1q41
209966_x_at	13	ESRRG	AB020639	1q41
34868_at	15	EST1B	AB029012	1q21.2
219216_at	13	ETAA16	NM_019002	2p13-p15
201573_s_at	15	ETF1	NM_004730	5q31.1
201574_at	15	ETF1	NM_004730	5q31.1
201931_at	15	ETFA	NM_000126	15q23-q25
202942_at	15	ETFB	NM_001985	19q13.3
205530_at	15	ETFDH	NM_004453	4q32-q35
33494_at	15	ETFDH	S69232	4q32-q35
204034_at	15	ETHE1	NM_014297	19q13.32
219017_at	15	ETNK1	NM_018638	12p12.2
205528_s_at	13	ETO	X79990	8q22
201328_at	15	ETS2	AL575509	21q22.3
201329_s_at	15	ETS2	NM_005239	21q22.3
222303_at	14	ETS2	AV700891	21q22.3
206501_x_at	15	ETV1	NM_004956	7p22
217061_s_at	10	ETV1	AC004857	---
221911_at	15	ETV1	BE881590	7p22
203348_s_at	12	ETV5	BF060791	3q28
203349_s_at	13	ETV5	NM_004454	3q28
221884_at	15	EVI1	BE466525	3q24-q28
204774_at	15	EVI2A	NM_014210	17q11.2
210347_s_at	11	EVI9	AF080216	2p16.1
217838_s_at	15	EVL	NM_016337	14q32.32
209214_s_at	15	EWSR1	BC004817	22q12.2
210011_s_at	15	EWSR1	BC000527	22q12.2
212034_s_at	15	EXOC7	AI817079	17q25.3
207541_s_at	15	EXOSC10	NM_002685	1p36.22
58696_at	12	EXOSC4	AL039469	8q24.3
91684_g_at	14	EXOSC4	AI571298	8q24.3
212627_s_at	15	EXOSC7	AL581473	3p21.32
213648_at	10	EXOSC7	AW614427	3p21.32
205061_s_at	15	EXOSC9	NM_005033	4q27
213226_at	15	EXOSC9	AI346350	4q27
201995_at	15	EXT1	NM_000127	8q24.11-q24.13
202012_s_at	15	EXT2	AA196245	11p12-p11
202013_s_at	15	EXT2	NM_000401	11p12-p11
209537_at	15	EXTL2	AF000416	1p21

220266_s_at	11	EZF; GKLF	AF105036	9q31
203249_at	13	EZH1	AB002386	17q21.1-q21.3
32259_at	15	EZH1	AB002386	17q21.1-q21.3
203358_s_at	12	EZH2	NM_004456	7q35-q36
214715_x_at	15	F11	AK024789	19q13.42
203305_at	15	F13A1	NM_000129	6p25.3-p24.3
213526_s_at	14	F25965	BF215644	19q13.1
203989_x_at	15	F2R	NM_001992	5q13
207300_s_at	14	F7	NM_000131	13q34
205756_s_at	15	F8	NM_000132	Xq28
203274_at	15	F8A1	NM_012151	Xq28
205738_s_at	15	FABP3	NM_004102	1p33-p32
214285_at	15	FABP3	AI041520	1p33-p32
203980_at	15	FABP4	NM_001442	8q21
202345_s_at	15	FABP5	NM_001444	8q21.13
201963_at	15	FACL2	NM_021122	4q34-q35
207275_s_at	15	FACL2	NM_001995	4q34-q35
201660_at	15	FACL3	AL525798	2q34-q35
201661_s_at	15	FACL3	D89053	2q34-q35
201662_s_at	15	FACL3	D89053	2q34-q35
202422_s_at	14	FACL4	NM_022977	Xq22.3-q23
202535_at	10	FADD	NM_003824	11q13.3
208963_x_at	15	FADS1	AL512760	11q12.2-q13.1
208964_s_at	15	FADS1	AL512760	11q12.2-q13.1
202218_s_at	15	FADS2	NM_004265	11q12-q13.1
204257_at	15	FADS3	NM_021727	11q12-q13.1
218080_x_at	15	FAF1	NM_007051	1p33
202862_at	15	FAH	NM_000137	15q23-q25
218504_at	15	FAHD2A	NM_016044	2p24.3-p11.2
222056_s_at	15	FAHD2A	AA723370	2p24.3-p11.2
220643_s_at	15	FAIM	NM_018147	3q22.3
207186_s_at	13	FALZ	NM_004459	17q24.3
219253_at	15	FAM11B	NM_024121	2q14.2
202972_s_at	15	FAM13A1	NM_014883	4q22.1
202973_x_at	15	FAM13A1	NM_014883	4q22.1
202915_s_at	15	FAM20B	NM_014864	1p36.13-q41
202916_s_at	14	FAM20B	NM_014864	1p36.13-q41
211068_x_at	15	FAM21C	BC006456	10q11.1 /// 10q11.1
57715_at	15	FAM26B	W72694	10pter-q26.12
201863_at	15	FAM32A	NM_014077	19pter-p13.3
220547_s_at	15	FAM35A	NM_019054	10q23.31
202771_at	15	FAM38A	NM_014745	16q24.3
209405_s_at	14	FAM3A	BC002934	Xq28
38043_at	15	FAM3A	X55448	Xq28
201889_at	15	FAM3C	NM_014888	7q22.1-q31.1
221804_s_at	15	FAM45A	AI697488	Xq26.1
221766_s_at	15	FAM46A	AW246673	6q14
220408_x_at	15	FAM48A	NM_017569	13q13.2
221774_x_at	15	FAM48A	AW003334	13q13.2
217916_s_at	15	FAM49B	NM_016623	8q24.21

203262_s_at	15	FAM50A	NM_004699	Xq28
205775_at	13	FAM50B	NM_012135	6p25-pter
203206_at	15	FAM53B	NM_014661	10q26.2
218023_s_at	15	FAM53C	NM_016605	5q31
221591_s_at	15	FAM64A	BC005004	17p13.2
218029_at	11	FAM65A	NM_024519	16q22.1
218126_at	15	FAM82C	NM_018145	15q14
203420_at	15	FAM8A1	NM_016255	6p22-p23
218074_at	15	FAM96B	NM_016062	16q22.1-q22.3
205189_s_at	15	FANCC	NM_000136	9q22.3
220255_at	15	FANCE	NM_021922	6p21-p22
203564_at	14	FANCG	NM_004629	9p13
218397_at	15	FANCL	NM_018062	2p16.1
201910_at	15	FARP1	BF213279	13q32.2-q32.3
201911_s_at	15	FARP1	NM_005766	13q32.2-q32.3
204282_s_at	14	FARS2	NM_006567	6p25.1
204283_at	14	FARS2	NM_006567	6p25.1
202159_at	13	FARSLA	NM_004461	19p13.2
204780_s_at	12	FAS	NM_000043	10q24.1
214114_x_at	15	FAST	AK023141	7q35 /// 7q35
202676_x_at	12	FASTK	NM_006712	7q35
210975_x_at	15	FASTK	BC000377	7q35
201579_at	15	FAT	NM_005245	4q34-q35
219427_at	15	FAT4	NM_024582	4q28.1
200019_s_at	15	FAU	NM_001997	11q13
211919_s_at	14	FB22	AF348491	2q21 /// 2q21
214436_at	12	FBL2	AF176518	3p22.3
209943_at	14	FBL4; FBL5	AF176699	6q16.1-q16.3
201787_at	11	FBLN1	NM_001996	22q13.31
202994_s_at	14	FBLN1	Z95331	22q13.31
202995_s_at	15	FBLN1	NM_006486	22q13.31
203886_s_at	15	FBLN2	NM_001998	3p25-p24
203088_at	10	FBLN5	NM_006329	14q32.1
202765_s_at	10	FBN1	AI264196	15q21.1
202766_s_at	15	FBN1	NM_000138	15q21.1
203184_at	15	FBN2	NM_001999	5q23-q31
211074_at	13	FBP	AF000381	---
211503_s_at	15	FBP; RAB-14	AF112206	9q32-q34.11
203598_s_at	15	FBP21; MGC117310	AK000979	13q13.3
212824_at	15	FBP3	U69127	9q34.2
218255_s_at	15	FBS1	NM_022452	16p11.2
212229_s_at	15	FBX21	AK001699	12q24.23
210638_s_at	15	FBX9	AF176704	6p12.3-p11.2
208988_at	15	FBXL11	BE675843	11q13.1
220127_s_at	15	FBXL12	NM_017703	19p13.2
213145_at	14	FBXL14	BF001666	12p13.33
213249_at	15	FBXL7	AU145127	5p15.1
203255_at	14	FBXO11	NM_018693	2p21
222119_s_at	15	FBXO11	AL117620	2p21
212231_at	15	FBXO21	AB020682	12q24.23

202272_s_at	15	FBXO28	NM_015176	1q42.12
218432_at	15	FBXO3	NM_012175	11p13
218539_at	15	FBXO34	NM_017943	14q22.2
205310_at	15	FBXO34L	NM_012066	19q13.3
221257_x_at	15	FBXO38	NM_030793	5q33.1 /// 5q33.1
220164_s_at	13	FBXO40	NM_016298	3q21.1
47773_at	13	FBXO42	AA836114	1p36.23-p36.11
218875_s_at	13	FBXO5	NM_012177	6q25-q26
201178_at	15	FBXO7	NM_012179	22q12-q13
212987_at	15	FBXO9	AL031178	6p12.3-p11.2
209455_at	15	FBXW11	BE963245	5q35.1
215600_x_at	15	FBXW12	AK022174	3p21.31
209630_s_at	15	FBXW2	U87460	9q34
218941_at	15	FBXW2	NM_012164	9q34
218751_s_at	15	FBXW7	NM_018315	4q31.3
204232_at	15	FCER1G	NM_004106	1q23
203561_at	10	FCGR2A	NM_021642	1q23
218831_s_at	15	FCGRT	NM_004107	19q13.3
203620_s_at	13	FCHSD2	NM_014824	11q13.3
205283_at	10	FCMD	NM_006731	9q31-q33
208647_at	15	FDFT1	AA872727	8p23.1-p22
210950_s_at	15	FDFT1	BC003573	8p23.1-p22
201275_at	15	FDPS	NM_002004	1q22
203646_at	14	FDX1	NM_004109	11q22
203647_s_at	15	FDX1	NM_004109	11q22
203116_s_at	15	FECH	NM_000140	18q21.3
212367_at	15	FEM1B	NM_015322	15q22
212373_at	15	FEM1B	AW139179	15q22
213341_at	14	FEM1C	AI862658	5q22
37384_at	15	FEM-2	D86995	22q11.22
204767_s_at	13	FEN1	BC000323	11q12
201798_s_at	15	FER1L3	NM_013451	10q24
211864_s_at	15	FER1L3	AF207990	10q24
203562_at	15	FEZ1	NM_005103	11q24.2
202305_s_at	14	FEZ2	NM_005102	2p21
204819_at	12	FGD1	NM_004463	Xp11.21
219901_at	13	FGD6	NM_018351	12q23.1
205117_at	15	fgf gene	X59065	5q31
208240_s_at	11	FGF1	NM_013394	5q31 /// 5q31
207501_s_at	15	FGF12	NM_004113	3q28
214589_at	15	FGF12	AL119322	3q28
205110_s_at	15	FGF13	NM_004114	Xq26.3
206987_x_at	15	FGF18	NM_003862	5q34
211029_x_at	15	FGF18	BC006245	5q34 /// 5q34
214284_s_at	12	FGF18	AA022949	5q34
205782_at	14	FGF7	NM_002009	15q15-q21.1
206404_at	15	FGF9	NM_002010	13q11-q12
222164_at	13	FGFR1	AU145411	---
205588_s_at	13	FGFR1OP	NM_007045	6q27
214124_x_at	15	FGFR1OP	AL043487	6q27

205305_at	15	FGL1	NM_004467	8p22-p21.3
204834_at	15	FGL2	NM_006682	7q11.23
203033_x_at	15	FH	NM_000143	1q42.1
214170_x_at	15	FH	AA669797	1q42.1
213800_at	15	FH	X04697	1q32
206492_at	15	FHIT	NM_002012	3p14.2
201540_at	15	FHL1	NM_001449	Xq26
210298_x_at	15	FHL1	AF098518	Xq26
214505_s_at	15	FHL1	AF220153	Xq26
201539_s_at	15	FHL-1	U29538	Xq26
210299_s_at	15	FHL1B	AF063002	Xq26
202949_s_at	15	FHL2	NM_001450	2q12-q14
218530_at	15	FHOD1	NM_013241	16q22
218980_at	15	FHOD3	NM_025135	18q12
202041_s_at	15	FIBP	NM_004214	11q13.1
215032_at	14	FINB	AK022442	---
212343_at	10	FinGER6	AL117461	Xq12
214879_x_at	14	FIP	AY007087	19q13
221007_s_at	15	FIP1L1	NM_030917	4q12 /// 4q12
209899_s_at	15	FIR	AF217197	8q24.2-qtel
222130_s_at	10	FJH1	AK024635	7p22
219522_at	12	FJX1	NM_014344	11p13
219117_s_at	15	FKBP11	NM_016594	12q13.12
219118_at	15	FKBP11	NM_016594	12q13.12
219390_at	14	FKBP14	NM_017946	7p15.1
200709_at	15	FKBP1A	NM_000801	20p13
210186_s_at	15	FKBP1A	BC005147	20p13
210187_at	15	FKBP1A	BC005147	20p13
214119_s_at	15	FKBP1A	AI936769	20p13
206857_s_at	14	FKBP1B	NM_004116	2p24.1
203391_at	15	FKBP2	NM_004470	11q13.1-q13.3
218003_s_at	15	FKBP25	M90820	14q21.3
200894_s_at	12	FKBP4	NM_002014	12p13.33
200895_s_at	15	FKBP4	NM_002014	12p13.33
212169_at	15	FKBP9	AL050187	7p11.1
208789_at	15	FKSG13	BC004295	17q21.31
208790_s_at	15	FKSG13	AF312393	17q21.31
211445_x_at	15	FKSG17	AF315951	8q22.3
209593_s_at	15	FKSG18	AF317129	9q34
208588_at	13	FKSG2	NM_021631	8p11.2
208120_x_at	15	FKSG49	NM_031221	---
211454_x_at	14	FKSG51	AF336878	---
205661_s_at	14	FLAD1	NM_025207	1q22
209046_s_at	15	FLC3A	AB030710	16q22.3-q24.1
204236_at	13	FLI1	NM_002017	11q24.1-q24.3
210786_s_at	11	FLI1	M93255	11q24.1-q24.3
212024_x_at	13	FLII	U80184	17p11.2
222065_s_at	15	FLII	AI830227	17p11.2
218349_s_at	12	FLJ10036	AA824298	15q22.2
218008_at	15	FLJ10099	NM_017994	7q11.22

218067_s_at	15	FLJ10154	NM_018011	13q33.2
218974_at	15	FLJ10159	NM_018013	6q21
45828_at	15	FLJ10241	AI768100	19q13.31
222200_s_at	15	FLJ10276	AK021440	1p34.3
219130_at	15	FLJ10287	NM_019083	1pter-q31.3
58780_s_at	15	FLJ10357	R42449	14q11.2
208933_s_at	15	FLJ10359	AI659005	1q42.3
44563_at	15	FLJ10385	AI858000	17p13.2
218920_at	15	FLJ10404	NM_019057	5q35.3
221656_s_at	15	FLJ10521	BC003073	1p36.13
218155_x_at	15	FLJ10534; KIAA1401	AK026565	17p13.3
218156_s_at	15	FLJ10534; KIAA1401	AK026565	17p13.3
63009_at	15	FLJ10539	AI188402	3p14.2
201918_at	15	FLJ10618	AI927944	3q23
201919_at	15	FLJ10618	AL049246	3q23
212326_at	15	FLJ10619	AB007922	1p36.21
218614_at	15	FLJ10652	NM_018169	12p11.22
221806_s_at	15	FLJ10707	BF590997	3p25.3
218314_s_at	15	FLJ10726	AA024582	11q23.2
215792_s_at	11	FLJ10737	AL109978	1p36.23
217940_s_at	15	FLJ10769	NM_018210	13q34
217884_at	15	FLJ10774	NM_024662	11p13
218824_at	15	FLJ10781	NM_018215	19q13.33
209445_x_at	15	FLJ10803	BC001743	7p13
209446_s_at	10	FLJ10803	BC001743	7p13
218340_s_at	12	FLJ10808	NM_018227	4q13.3
56821_at	12	FLJ10815	AI963454	16q13
212370_x_at	15	FLJ10824	AL080183	10q11.23
221090_s_at	12	FLJ10826	NM_018233	16q13
219044_at	10	FLJ10916	NM_018271	2p11.2
219718_at	13	FLJ10986	NM_018291	1p32.1
209688_s_at	15	FLJ10996	BC005078	2q14.2
209689_at	12	FLJ10996	BC005078	2q14.2
219774_at	14	FLJ10996	NM_019044	2q14.2
218521_s_at	14	FLJ11011	AK024050	8q13.3
219754_at	11	FLJ11016	NM_018301	Xq22.1-24
202301_s_at	15	FLJ11021	NM_023012	12q24.31
202302_s_at	15	FLJ11021	NM_023012	12q24.31
218545_at	15	FLJ11088	NM_018318	12p11.23
218513_at	14	FLJ11184	NM_018352	4q32.2
203738_at	15	FLJ11193	AI421192	5p13.3
218449_at	15	FLJ11200	NM_018359	4q35.1
213228_at	15	FLJ11212	AK023913	5q14.1
218627_at	12	FLJ11259	NM_018370	12q23.3
218930_s_at	15	FLJ11273	AV705186	7p21.3
218429_s_at	11	FLJ11286	NM_018381	19p13.2
53720_at	15	FLJ11286	AI862559	19p13.2
219940_s_at	15	FLJ11305	NM_018386	13q34
219791_s_at	15	FLJ11539	NM_024748	4q34.1
211433_x_at	15	FLJ11560	AL583909	9p13.2

219056_at	15	FLJ11712	NM_024570	13q14.13
218214_at	15	FLJ11773	NM_021934	12q13.13
204216_s_at	13	FLJ11806	NM_024824	14q31.3
213064_at	15	FLJ11806	N64802	14q31.3
214107_x_at	15	FLJ11822	AW340850	17
218153_at	15	FLJ12118	NM_024537	13q34
48659_at	15	FLJ12438	W60802	1p36.21
218051_s_at	15	FLJ12442	NM_022908	3p21.31
219022_at	10	FLJ12448	NM_022895	12q
217866_at	15	FLJ12529	NM_024811	11q12.3
215029_at	12	FLJ12666	AL117451	---
212766_s_at	15	FLJ12671	AW294587	1q23.1
46142_at	11	FLJ12681	AI003763	16p13.3
219627_at	14	FLJ12700	NM_024910	7q36.1
218179_s_at	14	FLJ12716	NM_021942	4q35.1
218838_s_at	13	FLJ12788	NM_022492	2p13.1
214700_x_at	15	FLJ12870	AK000323	2q24.1
222341_x_at	11	FLJ12892	AW973235	---
217593_at	12	FLJ12895	AI375002	19q13.43
218879_s_at	11	FLJ12998	NM_022764	16q24.1
208918_s_at	15	FLJ13052	BE674658	1p36.33-p36.21
208919_s_at	15	FLJ13052	BC001709	1p36.33-p36.21
219016_at	13	FLJ13149	NM_021826	---
219871_at	12	FLJ13197	NM_024614	4p14
218884_s_at	12	FLJ13220	AK025248	4p13
203579_s_at	12	FLJ13291	AI660619	16q22.1
222151_s_at	15	FLJ13386	AK023738	3q22.1
219397_at	15	FLJ13448	NM_025147	2q33.1
44617_at	15	FLJ13491	AI431902	12q24.31
218962_s_at	15	FLJ13576	NM_022484	7q31.32
218674_at	13	FLJ13611	NM_024941	5q12.3
204800_s_at	14	FLJ13639	NM_024705	13q14.2
220321_s_at	10	FLJ13646	NM_024584	2p23.3
45749_at	15	FLJ13725	AA400206	16q22.1
218734_at	15	FLJ13848	NM_024771	11q13.1
222369_at	15	FLJ13848	AW971254	11q13.1
217750_s_at	15	FLJ13855	BE544096	17q21.33
217891_at	14	FLJ13868	NM_022744	16p11.2
212482_at	15	FLJ13910	BF671894	2p11.2
212479_s_at	15	FLJ13910	AK022815	2p11.2
45633_at	11	FLJ13912	AI421812	16q13
213679_at	13	FLJ13946	AL049329	2q31.3
219054_at	15	FLJ14054	NM_024563	5p13.3
45526_g_at	15	FLJ14154	AI246641	16p13.3
212995_x_at	15	FLJ14346	BG255188	---
213166_x_at	15	FLJ14346	BG332462	2q21.2
211185_s_at	15	FLJ14753	AF130099	9q22.32
44065_at	11	FLJ14827	AI937468	12q24.21
200979_at	15	FLJ16518	BF739979	Xp22.13
208246_x_at	15	FLJ20006	NM_017618	16q22.1

218789_s_at	15	FLJ20010	NM_019021	11q14.2-q14.3
218986_s_at	14	FLJ20035	NM_017631	4q32.3
219696_at	14	FLJ20054	NM_019049	1q31.3
219467_at	15	FLJ20125	NM_017676	5q21.2
220520_s_at	11	FLJ20130	NM_017681	Xq22.3
218510_x_at	15	FLJ20152	NM_019000	5p15.1
218532_s_at	15	FLJ20152	NM_019000	5p15.1
202808_at	15	FLJ20154; FLJ20367	AK000161	10q24.33
219646_at	15	FLJ20186	NM_017702	16q24.3
219846_at	10	FLJ20203	NM_025174	1q22
204593_s_at	15	FLJ20232	AA046752	22q13
204594_s_at	14	FLJ20232	NM_013298	22q13
221516_s_at	15	FLJ20232	BC002587	22q13
58994_at	15	FLJ20241	AI689402	19p13.13
219620_x_at	14	FLJ20245	NM_017723	---
217899_at	15	FLJ20254	NM_017727	2p23.3
218710_at	15	FLJ20272	NM_017735	2p23.2-p23.1
219717_at	12	FLJ20280	NM_017741	4p15.33
208773_s_at	15	FLJ20288	AL136943	5q31.3
207856_s_at	15	FLJ20297	NM_017951	2q21.2
206860_s_at	15	FLJ20323	NM_019005	7p22-p21
211724_x_at	15	FLJ20323	BC005883	7p22-p21
218630_at	13	FLJ20345	NM_017777	17q23.2
218692_at	13	FLJ20366	NM_017786	8q23.2
218460_at	15	FLJ20397	NM_017802	7p22.3
47105_at	10	FLJ20399	AA886893	16q22.1
218745_x_at	10	FLJ20422	NM_017814	19p13.11
43977_at	14	FLJ20422	AI660497	19p13.11
220838_at	12	FLJ20433	NM_017820	9
221821_s_at	15	FLJ20436; FLJ12670	AK022732	12q13.12
218984_at	15	FLJ20485	NM_019042	7q22.2
48106_at	13	FLJ20489	H14241	12q13.11
219219_at	12	FLJ20512	NM_017854	19q13.33
218646_at	15	FLJ20534	NM_017867	4q33
217961_at	15	FLJ20551	NM_017875	3p21.33
219128_at	15	FLJ20558	NM_017880	2p13.3
203197_s_at	15	FLJ20580	AW157077	1p32.3
212337_at	15	FLJ20618	AI687738	22q12.2
221229_s_at	15	FLJ20628	NM_017910	2p23.3 /// 2p23.3
220060_s_at	11	FLJ20641	NM_017915	12q23.3
217872_at	15	FLJ20643	NM_017916	19q13.33
220137_at	11	FLJ20674	NM_019086	12q24.23
207730_x_at	15	FLJ20700	NM_017932	19p13.3
219093_at	13	FLJ20701	NM_017933	2q37.1
219289_at	14	FLJ20718	NM_017939	16q12.1
213612_x_at	15	FLJ20719	AI800419	1q21.2
217895_at	15	FLJ20758	NM_017952	2p11.2
214749_s_at	15	FLJ20811	AK000818	Xq21.33-q22.3
218958_at	13	FLJ20850	NM_017967	19p13.11
51200_at	15	FLJ20850	AI744084	19p13.11

218366_x_at	15	FLJ20859	NM_022734	14q11.2
218844_at	14	FLJ20920	NM_025149	17q21.33
219980_at	14	FLJ21106	NM_025097	4q28.2
219751_at	14	FLJ21148	NM_024860	16q13
203513_at	15	FLJ21439	NM_025137	15q14
219377_at	15	FLJ21610	NM_022751	18q12.1
219269_at	13	FLJ21616	NM_024567	8p21.1
219029_at	11	FLJ21657	NM_022483	5p12
218531_at	15	FLJ21749	NM_025124	11q13.1
219008_at	12	FLJ21820	NM_021925	2p24.2
218483_s_at	13	FLJ21827	NM_020153	11q23.3
220349_s_at	14	FLJ21865	NM_022759	17q25.3
65635_at	15	FLJ21865	AL044097	17q25.3
219002_at	15	FLJ21901	NM_024622	2q31
218842_at	15	FLJ21908	NM_024604	12q13.11
219616_at	15	FLJ21963	NM_024560	12q21.31
220032_at	13	FLJ21986	NM_024913	7q31.32
212918_at	15	FLJ22028	AI962943	12p12.3
219802_at	15	FLJ22028	NM_024854	12p12.3
222209_s_at	15	FLJ22104	AK000684	11q14.1
221800_s_at	13	FLJ22175	AL514147	17q25.3
53071_s_at	15	FLJ22222	AI885411	17q25.3
218262_at	15	FLJ22318	NM_022762	5q35.3
218394_at	15	FLJ22386	NM_024589	16p13.3
222143_s_at	14	FLJ22405; FLJ90311	AY007098	3p26
218175_at	15	FLJ22471	NM_025140	12q24.31
221925_s_at	13	FLJ22490	BE044503	8q13.1
219176_at	15	FLJ22555	NM_024520	2q33.1
219458_s_at	15	FLJ22609	AK025762	3q11.2
219544_at	15	FLJ22624	NM_024808	13q21.33
218454_at	15	FLJ22662	NM_024829	12p13.2
220486_x_at	15	FLJ22679	NM_017698	Xq22.3
218248_at	15	FLJ22794	NM_022074	11q12.2
220602_s_at	11	FLJ22795	NM_025084	15q25.1
219880_at	15	FLJ23053	NM_022907	5q35.3
221958_s_at	15	FLJ23091	AA775681	1p31.2
219747_at	13	FLJ23191	NM_024574	4q27
221208_s_at	15	FLJ23342	NM_024631	11q24.2
218916_at	15	FLJ23436	NM_024671	16p11.2
219217_at	15	FLJ23441	NM_024678	11q13.4
218647_s_at	14	FLJ23476	BE464161	1p34.2
216044_x_at	15	FLJ23493	AK027146	1p22.1
219487_at	15	FLJ23560	NM_024685	12q21.1
215046_at	13	FLJ23861	AL133053	2q34
211034_s_at	15	FLJ30092	BC006270	12q24.13
212532_s_at	15	FLJ30656	AW873564	---
218017_s_at	13	FLJ32731	NM_025070	8p11.1
216791_at	10	FLJ33318	AK026449	---
219221_at	15	FLJ35036	NM_024724	3q23
212547_at	15	FLJ35348	N34842	9q34.3

215143_at	15	FLJ36166	AL049437	7q22.1
217608_at	14	FLJ36754	AW408767	5q12.3
213294_at	15	FLJ38348	AV755522	2p22.3
212791_at	15	FLJ38984	AL042729	1p34.3
219817_at	10	FLJ39616	NM_016534	12q24.13
64432_at	15	FLJ39616	W05463	12q24.13
222052_at	15	FLJ41131	AA001552	19q13.2
214902_x_at	14	FLJ42393	AL080232	---
217419_x_at	15	FLJ45064	AK021586	1p36.33
208904_s_at	15	FLJ46061	BF431363	19p13.2
213715_s_at	14	FLJ46061	AF070591	19p13.3
35254_at	15	fln29	AB007447	12q
200859_x_at	15	FLNA	NM_001456	Xq28
213746_s_at	15	FLNA	AW051856	Xq28
214752_x_at	15	FLNA	AI625550	Xq28
207876_s_at	15	FLNC	NM_001458	7q32-q35
208749_x_at	15	FLOT1	AA507012	6p21.3
210142_x_at	15	FLOT1	AF117234	6p21.3
201350_at	15	FLOT2	NM_004475	17q11-q12
209004_s_at	15	FLR1	AF142481	4p15.33
204358_s_at	14	FLRT2	NM_013231	14q24-q32
204359_at	15	FLRT2	NM_013231	14q24-q32
219250_s_at	12	FLRT3	NM_013281	20p11
222033_s_at	15	FLT1	AA058828	13q12
211726_s_at	15	FMO2	BC005894	1q23-q25
206263_at	14	FMO4	NM_002022	1q23-q25
202709_at	15	FMOD	NM_002023	1q32
203689_s_at	14	FMR1	AI743037	Xq27.3
215245_x_at	14	FMR1	AA830884	Xq27.3
210495_x_at	15	FN1	AF130095	2q34
211719_x_at	15	FN1	BC005858	2q34 /// 2q34
212464_s_at	15	FN1	X02761	2q34
214701_s_at	10	FN1	AJ276395	2q34
216442_x_at	15	FN1	AK026737	2q34
218210_at	14	FN3KRP	NM_024619	17q25.3
219806_s_at	15	FN5	NM_020179	11q13.3-q23.3
212288_at	15	FNBP1	AB011126	9q34
213940_s_at	12	FNBP1	AU145053	9q34
215017_s_at	15	FNBP1L	AW270932	1p22.1
213729_at	15	FNBP3	Z78308	2q24.1
218053_at	15	FNBP3	NM_017892	2q24.1
212232_at	15	FNBP4	AB023231	11p11.2-p11.12
202304_at	15	FNDC3A	NM_014923	13q14.12
218618_s_at	15	FNDC3B	NM_022763	3q26.31
200090_at	15	FNTA	BG168896	8p22-q11
209189_at	15	FOS	BC004490	14q24.3
218880_at	11	FOSL2	NM_024530	2p23-p22
213260_at	15	FOXC1	AU145890	6p25
203734_at	15	FOXJ2	NM_018416	12p13.31
206015_s_at	15	FOXJ3	NM_014947	1pter-q31.3

217310_s_at	10	FOXJ3	AK027075	1pter-q31.3
203064_s_at	13	FOXK2	NM_004514	17q25
202580_x_at	14	FOXM1	NM_021953	12p13
202724_s_at	15	FOXO1A	NM_002015	13q14.1
204131_s_at	15	FOXO3A	N25732	---
204132_s_at	15	FOXO3A	NM_001455	6q21
215221_at	14	FOXP1	AK025064	---
205140_at	15	FPGT	NM_003838	1p31.1
212086_x_at	15	FPL	AK026584	1q21.2-q21.3
209471_s_at	15	FPTA	L00634	8p22-q11
41858_at	10	FRAG1; MGC799	AL049261	11p15.5
219889_at	13	FRAT1	NM_005479	10q24.2
209864_at	15	FRAT2	AB045118	10q24.1
203697_at	15	FRE	U91903	2qter
203698_s_at	15	FRE	U91903	2qter
204145_at	15	FRG1	NM_004477	4q35
215160_x_at	15	FRG1	AL441988	---
210220_at	12	frizzled	L37882	17q21.1
213056_at	15	FRMD4B	AU145019	3p14.2
209903_s_at	14	FRP1	U49844	3q22-q24
201564_s_at	13	FSCN1	NM_003088	7p22
200601_at	15	FSGS; FSGS1	U48734	19q13 /// 19q13
208310_s_at	15	FSTL1	NM_007085	3q13.33
208782_at	15	FSTL1	BC000055	3q13.33
200748_s_at	15	FTH1	NM_002032	11q13
214211_at	15	FTH1	AA083483	11q13
211628_x_at	15	FTHP1	J04755	--- /// ---
212788_x_at	15	FTL	BG537190	19q13.3-q13.4
213187_x_at	15	FTL	BG538564	19q13.3-q13.4
209702_at	15	FTO	U79260	16q12.2
218373_at	15	FTS	NM_022476	16q12.2
216304_x_at	15	FTSH	AJ295618	10p14
205324_s_at	15	FTSJ1	NM_012280	Xp11.23
218356_at	12	FTSJ2	NM_013393	7p22
218103_at	15	FTSJ3	NM_017647	17q23
203091_at	15	FUBP1	NM_003902	1p31.1
212847_at	15	FUBP1	AL036840	1p31.1
214093_s_at	15	FUBP1	AA156865	1p31.1
202838_at	15	FUCA1	NM_000147	1p34
201945_at	15	FURIN	NM_002569	15q26.1
200959_at	15	FUS	NM_004960	16p11.2
215744_at	10	FUS	AW514140	16p11.2
217370_x_at	12	FUS	S75762	16p11.2
204299_at	15	FUSIP1	NM_021993	---
206095_s_at	15	FUSIP1	NM_006625	1p36.11
213594_x_at	15	FUSIP1	AU130523	1p36.11
209578_s_at	15	FUT13; C21orf80	BC000626	21q22.3
202419_at	15	FVT1	NM_002035	18q21.3
216091_s_at	10	FWD1	AF101784	10q24.32
36936_at	15	FX; P35B	U58766	8q24.3

217981_s_at	15	FXC1	W67995	11p15.5-p15.3
205565_s_at	10	FXN	NM_000144	9q13-q21.1
201635_s_at	15	FXR1	NM_005087	3q28
201636_at	15	FXR1	BG025078	3q28
201637_s_at	15	FXR1	NM_005087	3q28
35265_at	15	FXR2	AF044263	17p13.1
205384_at	15	FXYD1	NM_005031	19q13.1
202488_s_at	15	FXYD3	NM_005971	19q13.13
218084_x_at	15	FXYD5	NM_014164	19q12-q13.1
217897_at	15	FXYD6	NM_022003	11q23.3
218204_s_at	15	FYCO1	NM_024513	3p21.32
212486_s_at	14	FYN	N20923	6q21
209414_at	10	FYR	AF083810	19p13.3
209416_s_at	15	FYR	AF083810	19p13.3
204451_at	15	FZD1	NM_003505	7q21
218665_at	15	FZD4	NM_012193	11q14.2
203987_at	15	FZD6	NM_003506	8q22.3-q23.1
203706_s_at	14	FZD7	NM_003507	2q33
213524_s_at	15	G0S2	NM_015714	1q32.2-q41
205690_s_at	15	G10	NM_003910	7q22.1
215535_s_at	15	G15	AF007145	6p21.3
32836_at	15	G15	U56417	6p21.3
205483_s_at	15	G1P2	NM_005101	1p36.33
204415_at	14	G1P3	NM_022873	1p35
214772_at	12	G2	H08993	11p13
208643_s_at	15	G22P1	J04977	2q35
214626_s_at	13	G2AN; GluII; KIAA0088	AK026548	11q12.3
201503_at	15	G3BP	BG500067	5q33.1
206383_s_at	11	G3BP2	NM_012297	4q21.21
208840_s_at	15	G3BP2	AB014560	4q21.21
208841_s_at	15	G3BP2	AB014560	4q21.21
217398_x_at	15	G3PD	AK026525	12p13
37278_at	15	G4.5	X92762	Xq28
208659_at	15	G6; NCC27	AF034607	6p22.1-p21.2
214909_s_at	15	G6a	AK026191	6p21.3
221453_at	10	G6PC2	NM_021176	2q24-q31
44654_at	15	G6PC3	AI669655	17q21.31
209449_at	15	G7b	AF196468	6p21.3
204332_s_at	14	GA; AGU; ASRG	M64073	4q32-q33
219158_s_at	12	Ga19	AF327722	4q31.1
202812_at	15	GAA	NM_000152	17q25.2-q25.3
207112_s_at	14	GAB1	NM_002039	4q31.1
214987_at	15	GAB1	AL049449	4q31.1
200645_at	15	GABARAP	NM_007278	17p13.2
208868_s_at	15	GABARAPL1	BF125756	12p13.31
208869_s_at	15	GABARAPL1	BF125756	12p13.31
211458_s_at	15	GABARAPL3	AF180519	15q26.1
209459_s_at	15	GABAT	AF237813	16p13.2
209460_at	15	GABAT	AF237813	16p13.2
203146_s_at	15	GABBR1	NM_001470	6p21.31

204618_s_at	15	GABPB2	NM_005254	15q21.2
205510_s_at	12	GABPB2	NM_017976	15q21.2
208463_at	13	GABRA4	NM_000809	4p12
207010_at	14	GABRB1	NM_000812	4p12
204537_s_at	14	GABRE	NM_004961	Xq28
37028_at	15	GADD34	U83981	19q13.2
203725_at	15	GADD45A	NM_001924	1p31.2-p31.1
207574_s_at	15	GADD45B	NM_015675	19p13.3
209304_x_at	15	GADD45B	AF087853	19p13.3
204121_at	14	GADD45G	NM_006705	9q22.1-q22.2
212891_s_at	15	GADD45GIP1	BF972185	19p13.2
210879_s_at	14	GAF1	AF334812	2p13-p12
204417_at	15	GALC	NM_000153	14q31
202528_at	10	GALE	NM_000403	1p36-p35
205219_s_at	13	GALK2	NM_002044	15q15.3
203066_at	15	GALNAC4S-6ST	NM_014863	10q26
218871_x_at	14	GALNACT-2	NM_018590	10q11.21
222235_s_at	14	GALNACT-2	AL139812	---
201722_s_at	15	GALNT1	NM_020474	18q12.1
201723_s_at	15	GALNT1	NM_020474	18q12.1
201724_s_at	15	GALNT1	NM_020474	18q12.1
219013_at	15	GALNT11	NM_022087	7q34-q36
217788_s_at	15	GALNT2	NM_004481	1q41-q42
218313_s_at	15	GALNT7	NM_017423	4q31.1
203179_at	15	GALT	NM_000155	9p13
205354_at	15	GAMT	NM_000156	19p13.3
211934_x_at	13	GANAB	W87689	11q12.3
210621_s_at	15	GAP	M23612	5q13.3
212581_x_at	15	GAPD	BE561479	12p13
213453_x_at	15	GAPD	BF689355	12p13
31874_at	14	GAR22; MGC17243	Y07846	22q12.2
213049_at	15	GARNL1	BG436400	14q13.2
214855_s_at	15	GARNL2	AL050050	9q31
212378_at	15	GART	NM_000819	21q22.1
204457_s_at	15	GAS1	NM_002048	9q21.3-q22
205848_at	15	GAS2	NM_005256	11p14.3-p15.2
210139_s_at	15	GAS3	L03203	17p12-p11.2
1598_g_at	15	GAS6	L13720	13q34
202177_at	15	GAS6	NM_000820	13q34
202191_s_at	15	GAS7	BE439987	17p
202192_s_at	13	GAS7	NM_005890	17p
209984_at	12	GASC-1	AB037901	9p24-p23
209710_at	15	GATA2	AL563460	3q21.3
209604_s_at	11	GATA3	AI796169	10p15
205517_at	15	GATA4	AV700724	8p23.1-p22
210002_at	15	GATA6	D87811	18q11.1-q11.2
208503_s_at	15	GATAD1	NM_021167	7q21-q22
203178_at	14	GATM	NM_001482	15q15.1
201816_s_at	15	GBAS	NM_001483	7p12
203282_at	15	GBE1	NM_000158	3p12.3

214835_s_at	15	G-BETA	AF131748	3p14.3
208961_s_at	14	GBF	AB017493	10p15
201439_at	15	GBF1	NM_004193	10q24
220587_s_at	14	GBL	NM_022372	16p13.3
208631_s_at	15	GBP	U04627	2p23
202269_x_at	15	GBP1	NM_002053	1p22.2
201738_at	15	GC20	NM_005875	3p21.33
203765_at	15	GCA	NM_012198	2q24.3
36475_at	15	GCAT	Z97630	22q13.1
202832_at	15	GCC2	NM_014635	2q12.3
203500_at	13	GCDH	NM_000159	19p13.2
208369_s_at	15	GCDH	NM_013976	19p13.2
204224_s_at	15	GCH1	NM_000161	14q22.1-q22.2
204867_at	15	GCHFR	NM_005258	15q15
202922_at	15	GCLC	BF676980	6p12
202923_s_at	12	GCLC	NM_001498	6p12
203925_at	15	GCLM	NM_002061	1p22.1
212139_at	15	GCN1L1	D86973	12q24.2
202182_at	15	GCN5L2	NM_021078	17q21
211555_s_at	14	GC-S-beta-1	AF020340	4q31.3-q33
213133_s_at	15	GCSH	AW237404	1q23.3
200008_s_at	15	GDI2	NM_001494	10p15
200009_at	15	GDI2	NM_001494	10p15
201864_at	15	GDIL	D45021	Xq28
216620_s_at	15	GEF10	AF009205	8p23
204472_at	15	GEM	NM_005261	8q13-q21
219539_at	12	GEMIN6	NM_024775	2p22.3
203986_at	15	GENX-3414	NM_003943	4q24-q25
216041_x_at	15	GEP	AK023348	17q21.32
210300_at	15	GES	AF152863	20q11.21
219821_s_at	10	GFOD1	NM_018988	6pter-p22.1
202722_s_at	15	GFPT1	NM_002056	2p13
205100_at	15	GFPT2	NM_005110	5q34-q35
45572_s_at	15	GGA1	AW009695	22q13.31
50277_at	15	GGA1	AW001443	22q13.31
208913_at	15	GGA2	AA868560	16p12
208914_at	12	GGA2	AW874641	16p12
208915_s_at	11	GGA2	AF190863	16p12
210658_s_at	15	GGA2	BC000284	16p12
213772_s_at	15	GGA2	BF196572	16p12
209411_s_at	12	GGA3	AW008018	17q25.2
205351_at	13	GGCX	NM_000821	2p12
214005_at	15	GGCX	BE326952	2p12
214006_s_at	12	GGCX	BE326952	2p12
203560_at	15	GGH	NM_003878	8q12.2
202321_at	14	GGPS1	AW299507	1q43
202322_s_at	15	GGPS1	AW299507	1q43
207131_x_at	14	GGT1	NM_013430	22q11.23
209180_at	15	GGTB	U49245	1p31
209181_s_at	15	GGTB	U49245	1p31

209248_at	15	GHITM	AL136713	10q23.2
209249_s_at	15	GHITM	AF131820	10q23.2
205498_at	15	GHR	NM_000163	5p13-p12
219243_at	13	GIMAP4	NM_018326	7q36.1
218805_at	11	GIMAP5	NM_018384	7q36.1
64064_at	14	GIMAP5	AI435089	7q36.1
219777_at	15	GIMAP6	NM_024711	7q36.1
207525_s_at	15	GIPC1	NM_005716	19p13.1
218030_at	13	GIT1	NM_014030	17p11.2
201667_at	15	GJA1	NM_000165	6q21-q23.2
204904_at	13	GJA4	NM_002060	1p35.1
40687_at	15	GJA4	M96789	1p35.1
214430_at	15	GLA	NM_000169	Xq22
201576_s_at	13	GLB1	NM_000404	3p21.33
213552_at	14	GLCE	W87398	15q22.31
211600_at	15	GLEPP1	U20489	12p13.3-p13.2
207966_s_at	15	GLG1	NM_012201	16q22-q23
212045_at	14	GLG1	N32761	16q22-q23
207034_s_at	11	GLI2	NM_030379	2q14
207153_s_at	13	GLMN	NM_007070	1p22.1
200681_at	15	GLO1	NM_006708	6p21.3-p21.1
205279_s_at	15	GLRB	AF094754	4q31.3
205280_at	13	GLRB	NM_000824	4q31.3
206662_at	15	GLRX	NM_002064	5q14
219933_at	15	GLRX2	NM_016066	1q31.2-q31.3
203159_at	15	GLS	AB020645	2q32-q34
221510_s_at	15	GLS	AI828035	2q32-q34
218473_s_at	15	GLT25D1	NM_024656	19p13.12
219015_s_at	15	GLT28D1	BC005336	Xq23
218146_at	15	GLT8D1	NM_018446	3p21.31
218147_s_at	15	GLT8D1	NM_018446	3p21.31
221447_s_at	11	GLT8D2	NM_031302	12q /// 12q
217807_s_at	15	GLTSCR2	NM_015710	19q13.3
200946_x_at	15	GLUD1	AI339331	10q23.3
200947_s_at	15	GLUD1	NM_005271	10q23.3
200648_s_at	13	GLUL	NM_002065	1q31
215001_s_at	15	GLUL	AL161952	1q31
217202_s_at	15	GLUL	U08626	---
214864_s_at	15	GLXR	AK024386	9q12
216308_x_at	15	GLXR	AK026752	9q12
35436_at	15	GM130; MGC20672	L06147	9q34.13
208798_x_at	15	GM88	AF204231	15q11.2
218458_at	14	GMCL1L	NM_022471	5q35.3
204875_s_at	13	GMDS	NM_001500	6p25
214106_s_at	11	GMDS	AI762113	6p25
222251_s_at	11	GMEB2	AL133646	20q13.33
44146_at	14	GMEB2	AA045183	20q13.33
202543_s_at	15	GMFB	BC005359	14q22.2
202544_at	15	GMFB	NM_004124	14q22.2
204220_at	15	GMFG	NM_004877	19q13.2

218350_s_at	15	GMNN	NM_015895	6p22.1
208761_s_at	15	GMP1	U83117	2q33
218070_s_at	15	GMPPA	NM_013335	2q36.1
204187_at	15	GMPR	NM_006877	6p23
217990_at	15	GMPR2	NM_016576	14q11.2
214431_at	15	GMPS	NM_003875	3q24
213766_x_at	15	GNA11	N36926	19p13.3
213944_x_at	15	GNA11	BG236220	19p13.3
40562_at	15	GNA11	AF011499	19p13.3
564_at	15	GNA11	M69013	19p13.3
201179_s_at	14	GNAI1	J03005	1p13
209576_at	15	GNAI1	AL049933	7q21
201040_at	11	GNAI2	NM_002070	3p21
201181_at	14	GNAI3	NM_006496	1p13
204762_s_at	13	GNAO1	NM_020988	16q13
202615_at	15	GNAQ	BF222895	---
200780_x_at	15	GNAS	NM_000516	20q13.2-q13.3
200981_x_at	15	GNAS	NM_016592	20q13.2-q13.3
212273_x_at	15	GNAS	AI591100	20q13.2-q13.3
214157_at	12	GNAS	AA401492	20q13.2-q13.3
217673_x_at	15	GNAS	AA650558	---
200744_s_at	15	GNB1	NM_002074	1p36.33
200745_s_at	15	GNB1	AF070603	1p36.33
200746_s_at	15	GNB1	NM_002074	1p36.33
200852_x_at	15	GNB2	NM_005273	7q21.3-q22.1
200651_at	15	GNB2L1	NM_006098	5q35.3
222034_at	15	GNB2L1	AA443762	5q35.3
204000_at	14	GNB5	NM_016194	15q21.1
205042_at	15	GNE	NM_005476	9p13.1
204115_at	15	GNG11	NM_004126	7q31-q32
212294_at	15	GNG12	BG111761	1p31.2
207157_s_at	15	GNG5	NM_005274	1p22
206896_s_at	15	GNG7	NM_005145	19p13.3
217629_at	11	GNGT2	AA365670	---
201948_at	15	GNL2	NM_013285	1p34.2
217850_at	15	GNL3	NM_014366	3p21.31
201956_s_at	15	GNPAT	NM_014236	1q42.11-42.3
202382_s_at	15	GNPDA1	NM_005471	5q21
213852_at	15	GNRHR2	BG289199	1q12
212334_at	15	GNS	AW167793	12q14
212335_at	15	GNS	AW167793	12q14
207447_s_at	12	GNTIVH	NM_013244	12q21
203384_s_at	14	GOLGA1	NM_002077	9q34.11
202106_at	15	GOLGA3	NM_005895	12q24.33
201567_s_at	15	GOLGA4	NM_002078	3p22-p21.3
215203_at	11	GOLGA4	AW438464	3p22-p21.3
210425_x_at	15	GOLGA5	AF164622	15q11.2
218241_at	15	GOLGA5	NM_005113	14q32.12-q32.13
217819_at	15	GOLGA7	NM_016099	8p11.21
201056_at	15	GOLGB1	N53479	---

201057_s_at	15	GOLGB1	NM_004487	3q13
213650_at	14	GOLGIN-67	AW006438	15q11.2
217771_at	12	GOLPH2	NM_016548	9q21.33
217803_at	15	GOLPH3	NM_022130	5p13.3
204324_s_at	13	GOLPH4	NM_014498	3q26.2
218873_at	15	GON4	NM_017710	---
207812_s_at	15	GORASP2	NM_015530	2q31.1-q31.2
208842_s_at	15	GORASP2	W93787	2q31.1-q31.2
204630_s_at	15	GOSR1	NM_004871	17q11
213020_at	13	GOSR1	AI814252	17q11
213021_at	15	GOSR1	AI741876	17q11
210009_s_at	12	GOSR2	AF229796	17q21
213180_s_at	15	GOSR2	BE895285	17q21
208813_at	15	GOT1	BC000498	10q24.1-q25.1
200708_at	15	GOT2	NM_002080	16q21
207389_at	12	GP1BA	NM_000173	17pter-p12
206655_s_at	13	GP1BB	NM_000407	22q11.21-q11.23
201618_x_at	14	GPAA1	NM_003801	8q24.3
211060_x_at	15	GPAA1	BC006383	8q24.3 /// 8q24.3
215690_x_at	15	GPAA1	AL157437	8q24.3
209433_s_at	14	GPAT	U00238	4q12
209434_s_at	10	GPAT	U00238	4q12
219078_at	14	GPATC2	NM_018040	1q41
218895_at	13	GPATC3	NM_022078	1p35.3-p35.1
202756_s_at	15	GPC1	NM_002081	2q35-q37
209220_at	15	GPC3	L47125	Xq26.1
204983_s_at	14	GPC4	AF064826	Xq26.1
212510_at	15	GPD1L	AA135522	3p22.3
211821_x_at	13	GPErik	U00178	4q28.2-q31.1
220773_s_at	15	GPHN	NM_020806	14q23.3-q24.1
208308_s_at	15	GPI	NM_000175	19q13.1
218652_s_at	14	GPI7	NM_017733	4p16.3
209707_at	15	GPI8; MGC22559	AF022913	1p31.1
200723_s_at	15	GPIAP1	NM_005898	11p13
203776_at	15	GPKOW	NM_015698	Xp11.23
209167_at	15	GPM6B	AI419030	Xp22.2
209168_at	15	GPM6B	AW148844	Xp22.2
209169_at	15	GPM6B	N63576	Xp22.2
209170_s_at	15	GPM6B	AI419030	Xp22.2
201141_at	15	GPNMB	NM_002510	7p15
211977_at	15	GPR107	AK024651	9q34.2
211979_at	13	GPR107	AB046844	9q34.2
212950_at	15	GPR116	BF941499	6p21.1
212951_at	15	GPR116	N95226	6p21.1
65718_at	15	GPR124	AI655903	8p11.23
210473_s_at	14	GPR125	M37712	---
213094_at	14	GPR126	AL033377	---
221902_at	10	GPR153	AL567940	---
214104_at	15	GPR161	AI703188	1q23.3
218855_at	12	GPR175	NM_016372	3q21.2

221288_at	14	GPR22	NM_005295	7q22-q31.1
206960_at	13	GPR23	NM_005296	Xq13-q21.1
221306_at	11	GPR27	NM_018971	3p21-p14
212070_at	13	GPR56	AL554008	16q13
220642_x_at	15	GPR89	NM_016334	1p36.13-q31.3
204793_at	15	GPRASP1	NM_014710	Xq22.1
203632_s_at	15	GPRC5B	NM_016235	16p12
217782_s_at	15	GPS1	NM_004127	17q25.3
209350_s_at	15	GPS2	AL157493	17p13
213628_at	15	GPSM2	AA883493	1p13.3
221922_at	12	GPSM2	AW195581	1p13.3
208336_s_at	10	GPSN2	NM_004868	19p13.13
200736_s_at	15	GPX1	NM_000581	3p21.3
201348_at	15	GPX3	NM_002084	5q23
214091_s_at	15	GPX3	AW149846	5q23
201106_at	15	GPX4	NM_002085	19p13.3
213170_at	15	GPX7	AA406605	1p32
216321_s_at	15	GR	X03348	5q31
218706_s_at	12	GRAMD3	NM_023927	5q23.3
209409_at	15	GRB10	D86962	7p12-p11.2
206204_at	13	GRB14	NM_004490	2q22-q24
201347_x_at	15	GRHPR	NM_012203	9q12
212090_at	14	GRINA	AL571424	8q24.3
212241_at	15	GRINL1A	AI632774	15q22.1
212243_at	15	GRINL1A	BE645501	15q22.1
212244_at	15	GRINL1A	AL050091	15q22.1
204395_s_at	11	GRK5	NM_005308	10q24-qter
204396_s_at	15	GRK5	NM_005308	10q24-qter
200678_x_at	15	GRN	NM_002087	17q21.32
211284_s_at	15	GRN	BC000324	17q21.32
209774_x_at	11	gro-beta	M57731	4q21
212432_at	13	GRPEL1	AL542571	4p16
212434_at	15	GRPEL1	AI984421	4p16
201501_s_at	15	GRSF1	NM_002092	4q13
201520_s_at	15	GRSF1	BF034561	4q13
215030_at	15	GRSF1	AK023187	4q13
209276_s_at	15	GRX	AF162769	5q14
209025_s_at	15	GRY-RBP	AF037448	6q14-q15
209515_s_at	11	GS2	U38654	15q15-q21.1
210951_x_at	10	GS2	AF125393	15q15-q21.1
218154_at	13	GSDMDC1	NM_024736	8q24.3
211630_s_at	15	gsh-s	L42531	---
632_at	15	GSK3A	L40027	19q13.31
209945_s_at	15	GSK3B	BC000251	3q13.3
200696_s_at	15	GSN	NM_000177	9q33
201912_s_at	15	GSPT1	NM_002094	16p13.1
215438_x_at	15	GSPT1	BE906054	16p13.1
205541_s_at	14	GSPT2	NM_018094	Xp11.23-p11.21
201415_at	14	GSS	NM_000178	20q11.2
210912_x_at	15	GST	M99422	1p13.3

215766_at	15	GSTA1	AL096729	6p12.1
202967_at	15	GSTA4	NM_001512	6p12.1
217751_at	15	GSTK1	NM_015917	7q35
204550_x_at	15	GSTM1	NM_000561	1p13.3
204418_x_at	15	GSTM2	NM_000848	1p13.3
202554_s_at	15	GSTM3	AL527430	1p13.3
204149_s_at	10	GSTM4	NM_000850	1p13.3
205752_s_at	14	GSTM5	NM_000851	1p13.3
201470_at	15	GSTO1	NM_004832	10q25.1
200824_at	15	GSTP1	NM_000852	11q13
203815_at	13	GSTT1	NM_000853	22q11.23
210088_x_at	15	GT1	M36172	17q21-qter
201883_s_at	14	GT1	D29805	9p13
209617_s_at	15	GT24; NPRAP	AF035302	5p15.2
211450_s_at	10	GTBP	D89646	2p16
219770_at	15	GTDC1	NM_024659	2q22.3-q23.1
220853_at	12	GTDC1	NM_014118	2q22.3-q23.1
202678_at	15	GTF2A2	NM_004492	15q21.3
208066_s_at	15	GTF2B	NM_001514	1p22-p21
205930_at	15	GTF2E1	NM_005513	3q21-q24
202680_at	15	GTF2E2	NM_002095	8p21-p12
202355_s_at	15	GTF2F1	BC000120	19p13.3
202356_s_at	15	GTF2F1	NM_002096	19p13.3
209595_at	14	GTF2F2	BC001771	13q14
202451_at	15	GTF2H1	BC000365	11p15.1-p14
202453_s_at	15	GTF2H1	BC000365	11p15.1-p14
222104_x_at	12	GTF2H3	AI569458	12q24.31
203577_at	15	GTF2H4	NM_001517	6p21.3
213357_at	15	GTF2H5	AV701318	---
201065_s_at	15	GTF2I	NM_001518	7q11.23
210891_s_at	15	GTF2I	AF035737	7q11.23
218412_s_at	10	GTF2IRD1	NM_016328	7q11.23
201338_x_at	15	GTF3A	NM_002097	13q12.3-q13.1
215091_s_at	15	GTF3A	BE542815	13q12.3-q13.1
204366_s_at	14	GTF3C2	NM_001521	2p23.3
212429_s_at	14	GTF3C2	AW194657	2p23.3
218343_s_at	15	GTF3C3	NM_012086	2q33.1
210794_s_at	15	GTL2	AF119863	14q32
217957_at	15	GTL3	NM_013242	16q13
219357_at	11	GTPBP1	NM_014027	22q13.1
221050_s_at	12	GTPBP2	NM_019096	6p21-p12
213835_x_at	15	GTPBP3	AL524262	19p13.12
218238_at	15	GTPBP4	NM_012341	10p15-p14
218239_s_at	15	GTPBP4	NM_012341	10p15-p14
203314_at	15	GTPBP6	NM_012227	Xp22.33
212767_at	11	GTPBP7	BC004409	10q26.3
218088_s_at	15	GTR2	AK023373	1p34
204318_s_at	14	GTSE1	NM_016426	22q13.2-q13.3
211040_x_at	15	GTSE1	BC006325	22q13.2-q13.3
215942_s_at	14	GTSE1	BF973178	22q13.2-q13.3

221699_s_at	15	GU2	AF334103	10q22.1 /// 10q22.1
221942_s_at	15	GUCY1A3	AI719730	4q31.3-q33
203817_at	15	GUCY1B3	W93728	4q31.3-q33
200075_s_at	15	GUK1	BC006249	1q32-q41 /// 1q32-q41
204237_at	15	GULP1	NM_016315	2q32.3-q33
202605_at	15	GUSB	NM_000181	7q21.11
215938_s_at	11	GVI	AK001290	22q13.1
211275_s_at	15	GYG	AF087942	3q24-q25.1
201554_x_at	15	GYG1	NM_004130	3q24-q25.1
214407_x_at	11	GYPA	AI240545	4q28-q31
207459_x_at	10	GYPB	NM_002100	4q28-q31
202947_s_at	15	GYPC	NM_002101	2q14-q21
201673_s_at	15	GYS1	NM_002103	19q13.3
215388_s_at	15	H 36-2	X56210	1q32
221667_s_at	15	H11	AF133207	12q24.23
208886_at	15	H1F0	BC000145	22q13.1
204805_s_at	15	H1FX	NM_006026	3q21.3
211535_s_at	15	H2	M60485	8p11.2-p11.1
215404_x_at	13	H2	AK024388	8p11.2-p11.1
214500_at	15	H2A.y	AF044286	5q31.3-q32
214501_s_at	15	H2A.y	AF044286	5q31.3-q32
220936_s_at	11	H2AFJ	NM_018267	12p12
202487_s_at	15	H2AFV	NM_012412	7p13
212205_at	15	H2AFV	AA534860	7p13
205436_s_at	15	H2AFX	NM_002105	11q23.2-q23.3
213344_s_at	15	H2AFX	H51429	11q23.2-q23.3
207168_s_at	15	H2AFY	NM_004893	5q31.3-q32
220375_s_at	13	H2AFY	NM_024752	5q31.3-q32
200853_at	15	H2AFZ	NM_002106	4q24
213911_s_at	15	H2AFZ	BF718636	4q24
216323_x_at	14	H2-ALPHA	K03460	---
208579_x_at	13	H2BFS	NM_017445	21q22.3
216885_s_at	14	H326	AK026481	1q22-q23
200080_s_at	15	H3F3A	AI955655	1q41
208755_x_at	15	H3F3A	BF312331	1q41
211940_x_at	15	H3F3A	BE869922	1q41
213826_s_at	15	H3F3A	AA292281	1q41
213828_x_at	15	H3F3A	AA477655	1q41
209069_s_at	15	H3F3B	BC001124	17q25
211997_x_at	15	H3F3B	NM_005324	17q25
211998_at	15	H3F3B	Z48950	17q25
211999_at	15	H3F3B	Z48950	17q25
212010_s_at	15	H41	AK025647	3q22.1
213554_s_at	14	H41	AI928407	3q22.1
209620_s_at	15	hABC7	AB005289	Xq12-q13
202282_at	15	HADH2	NM_004493	Xp11.2
208629_s_at	14	HADHA	BG472176	2p23
208630_at	15	HADHA	AI972144	2p23
201007_at	15	HADHB	NM_000183	2p23

201035_s_at	15	HADHSC	BC000306	4q22-q26
201036_s_at	15	HADHSC	NM_005327	4q22-q26
205012_s_at	15	HAGH	NM_005326	16p13.3
220491_at	15	HAMP	NM_021175	19q13.1
221744_at	15	HAN11	AW575465	17q24.2
221745_at	14	HAN11	AW575465	17q24.2
220138_at	15	HAND1	NM_004821	5q33
217020_at	13	HAP; RRB2; NR1B2	X04014	---
202042_at	15	HARS	NM_002109	5q31.3
203138_at	15	HAT1	NM_003642	2q31.2-q33.1
201145_at	15	HAX1	NM_006118	1q22
200077_s_at	15	hAZ-brain	D87914	19p13.3 /// 19p13.3
214438_at	15	HB24	M60721	1q41-q42.1
204018_x_at	15	HBA1	NM_000558	16p13.3
209458_x_at	15	HBA1	AF105974	16p13.3
211699_x_at	15	HBA1	AF349571	16p13.3 /// 16p13.3
214414_x_at	15	HBA1	T50399	16p13.3
217414_x_at	15	HBA1	V00489	---
211745_x_at	15	HBA2	BC005931	16p13.3 /// 16p13.3
211696_x_at	15	HBB	AF349114	11p15.5 /// 11p15.5
217232_x_at	15	HBB	AF059180	---
209116_x_at	15	HBD	M25079	11p15.5
205919_at	14	HBE1	NM_005330	11p15.5
203821_at	12	HBEGF	NM_001945	5q23
204848_x_at	15	HBG1	NM_000559	11p15.5
204419_x_at	15	HBG2	NM_000184	11p15.5
213515_x_at	15	HBG2	AI133353	11p15.5
210973_s_at	10	HBGF-R	M63889	8p11.2-p11.1
209273_s_at	15	HBLD2	BG387555	9q22.1
209274_s_at	15	HBLD2	BC002675	9q22.1
221425_s_at	15	HBLD2	NM_030940	9q22.1 /// 9q22.1
209466_x_at	15	HBNF-1	M57399	7q33-q34
209102_s_at	15	HBP1	AF019214	7q22-q31
209316_s_at	15	HBS1L	BC001465	6q23-q24
215786_at	10	HBXAP	AK022170	---
218166_s_at	14	HBXAP	NM_016578	11q13.4
202299_s_at	15	HBXIP	NM_006402	1p13.2
202300_at	15	HBXIP	NM_006402	1p13.2
209092_s_at	15	HC71; CGI-150	AF061730	17p13.3
218345_at	15	HCA112	NM_018487	7q36.1
209259_s_at	15	HCAP	AF020043	10q25
204207_s_at	10	hCAP1a	AB012142	6q16
218662_s_at	15	HCAP-G	NM_022346	4p16-p15
218663_at	14	HCAP-G	NM_022346	4p16-p15
203745_at	15	HCCS	AI801013	Xp22.3
203746_s_at	15	HCCS	NM_005333	Xp22.3
210657_s_at	15	hcdcrel2b	U88870	17q22-q23
202474_s_at	13	HCFC1	NM_005334	Xq28
45714_at	15	HCFC1R1	AA436930	16p13.3
219484_at	14	HCFC2	NM_013320	12q23.3

201534_s_at	15	HCG-1	AF044221	13q12-q13
215985_at	10	hcgVIII	X92110	---
208018_s_at	12	HCK	NM_002110	20q11-q12
202957_at	15	HCLS1	NM_005335	3q13
217965_s_at	15	HCNGP	NM_013260	17q25.3
37424_at	12	HCR	AB029343	6p21.3
37425_g_at	15	HCR	AB029343	6p21.3
208978_at	15	hCRP2	U36190	14q32.3
201209_at	15	HDAC1	NM_004964	1p34
201833_at	15	HDAC2	NM_001527	6q21
216326_s_at	15	HDAC3	AF059650	---
206846_s_at	15	HDAC6	NM_006044	Xp11.23
217937_s_at	10	HDAC7A	NM_016596	12q13.1
205659_at	15	HDAC9	NM_014707	7p21.1
212785_s_at	15	HDCMA18P	AA160181	4q26
203260_at	11	HDDC2	NM_016063	6q13-q24.3
200896_x_at	15	HDGF	NM_004494	Xq25
216484_x_at	15	HDGF	L24521	Xq25
216693_x_at	15	HDGFRP3	AL133102	15q11.2
209524_at	15	HDGFRP3	AK001280	15q11.2
203974_at	15	HDHD1A	NM_012080	Xp22.32
209190_s_at	11	HDIA1	AF051782	5q31
200643_at	14	HDLBP	NM_005336	2q37
221767_x_at	15	HDLBP	AA515560	2q37
208846_s_at	15	HD-VDAC3	U90943	8p11.2
209606_at	13	HE	L06633	2q11.2
204370_at	15	HEAB	NM_006831	11q12
209657_s_at	15	heat shock factor 2	M65217	6q22.32
218594_at	15	HEATR1	NM_018072	1q42.3
218450_at	15	HEBP1	NM_015987	12p13.2
203430_at	15	HEBP2	NM_014320	6q24
218603_at	15	HECA	NM_016217	6q23-q24
218632_at	15	HECTD3	NM_024602	---
209572_s_at	15	HEED; WAIT1	AF080227	11q14.2-q22.3
212822_at	10	HEG	AA121502	3q21.2
213069_at	14	HEG	AI148659	3q21.2
203674_at	15	HELZ	NM_014877	17q24.3
218620_s_at	12	HEMK1	NM_016173	3p21.3
52159_at	15	HEMK1	W93807	3p21.3
203903_s_at	15	HEPH	NM_014799	Xq11-q12
218306_s_at	15	HERC1	NM_003922	15q22
217902_s_at	15	HERC2	NM_004667	15q13
219863_at	15	HERC5	NM_016323	4q22.1-q23
210036_s_at	15	HERG	AB044806	7q35-q36
1487_at	15	hERRa	L38487	11q13
203394_s_at	15	HES1	BE973687	3q28-q29
203395_s_at	15	HES1	NM_005524	3q28-q29
215933_s_at	15	HEX	Z21533	10q23.33
201765_s_at	15	HEXA	AL523158	15q23-q24
201944_at	15	HEXB	NM_000521	5q13

202814_s_at	15	HEXIM1	NM_006460	17q21.31
44783_s_at	15	HEY1	R61374	8q21
219743_at	15	HEY2	NM_012259	6q22.2-q22.33
201824_at	15	HFB30	AB022663	5q23.3-q31.1
206087_x_at	13	HFE	NM_000410	6p21.3
211327_x_at	12	HFE	AF149804	6p21.3
40148_at	11	hFE65L	U62325	4p14
202231_at	15	hfl-B5	NM_006360	11p13
202232_s_at	15	hfl-B5	NM_006360	11p13
213082_s_at	14	hfrc	AJ005866	9q22.33
213083_at	13	hfrc	AJ005866	9q22.33
211801_x_at	10	hfzo1	AF329637	3q27.1
213592_at	15	HG11	X89271	11q12
217457_s_at	11	hGDS	X63465	4q23-q25
209410_s_at	14	hGrb10zeta	AF000017	7p12-p11.2
217812_at	15	HGRG8	BC002559	14q12-q21
208615_s_at	15	HH13	AF208850	1p35
208617_s_at	15	HH13	AF208850	1p35
210695_s_at	15	HHCMA56	U13395	16q23.3-q24.1
212255_s_at	15	HHD	AK001684	3q21-q24
204689_at	15	HHEX	NM_001529	10q23.33
220387_s_at	10	HHLA3	NM_007071	1p31.2
203711_s_at	15	HIBCH	NM_014362	2q32.3
213374_x_at	15	HIBCH	AW000964	2q32.3
211675_s_at	15	HIC	AF054589	7q31.2 /// 7q31.2
212964_at	11	HIC2	AI912206	22q11.21
212966_at	13	HIC2	AL043112	22q11.21
200989_at	15	HIF1A	NM_001530	14q21-q24
59999_at	15	HIF1AN	W37897	10q24
219319_at	15	HIF3A	NM_022462	19q13.33
222123_s_at	14	HIF3A	AK021881	19q13.33
221896_s_at	15	HIG1	BE739519	3p21.33
218507_at	15	HIG2	NM_013332	7q32.2
217845_x_at	15	HIGD1A	NM_014056	3p21.33
209107_x_at	11	Hin-2	U19179	2p23
208826_x_at	15	HINT	U27143	5q31.2
200093_s_at	15	HINT1	N32864	5q31.2
207721_x_at	15	HINT1	NM_005340	5q31.2
205425_at	12	HIP1	NM_005338	7q11.23
202346_at	15	HIP2	NM_005339	4p14
212291_at	11	HIPK1	AI393355	1p13.1
212293_at	14	HIPK1	BG111260	1p13.1
219028_at	13	HIPK2	NM_022740	7q32-q34
209687_at	15	hIRH	U19495	10q11.1
218946_at	15	HIRIP5	NM_015700	2p15-p13
203253_s_at	14	HISPPD1	NM_015216	5q21.2
209398_at	15	HIST1H1C	BC002649	6p21.3
215071_s_at	15	HIST1H2AC	AL353759	---
209911_x_at	15	HIST1H2BD	BC002842	6p21.3
222067_x_at	10	HIST1H2BD	AL353759	6p21.3

210387_at	12	HIST1H2BG	BC001131	6p21.3
209806_at	15	HIST1H2BK	BC000893	6p21.33
205967_at	13	HIST1H4C	NM_003542	6p21.3
208180_s_at	10	HIST1H4H	NM_003543	6p21.3
214290_s_at	15	HIST2H2AA	AI313324	1q21.3
218280_x_at	15	HIST2H2AA	BC001629	1q21.3
202708_s_at	15	HIST2H2BE	NM_003528	1q21-q23
221582_at	15	HIST3H2A	BC001193	1q42.13
204512_at	15	HIVEP1	NM_002114	6p24-p22.3
212641_at	13	HIVEP2	AL023584	6q23-q24
212642_s_at	15	HIVEP2	AL023584	6q23-q24
209098_s_at	12	HJ1	U73936	20p12.1-p11.23
209099_x_at	15	HJ1	U73936	20p12.1-p11.23
200697_at	15	HK1	NM_000188	10q22
209186_at	15	HK1	M23114	12q23-q24.1
202934_at	15	HK2	AI761561	2p13
222029_x_at	15	HKE2	NM_014260	6p21.3
215313_x_at	15	HLA-A	AA573862	6p21.3
208729_x_at	15	HLA-B	D83043	6p21.3
211911_x_at	15	HLA-B	L07950	6p21.3 /// 6p21.3
213932_x_at	15	HLA-B	AI923492	6p21.3
217456_x_at	15	HLA-B	M31183	6p21.3
209140_x_at	15	HLA-B39	L42024	6p21.3
214459_x_at	15	HLA-C	M12679	6p21.3
211799_x_at	15	HLA-Cw*1701	U62824	6p21.3
217478_s_at	15	HLA-DMA	X76775	---
203932_at	15	HLA-DMB	NM_002118	6p21.3
211990_at	15	HLA-DPA1	M27487	6p21.3
211991_s_at	15	HLA-DPA1	M27487	6p21.3
201137_s_at	15	HLA-DPB1	NM_002121	6p21.3
212671_s_at	10	HLA-DQA1	BG397856	6p21.3
211654_x_at	11	HLA-DQB1	M17565	6p21.3 /// 6p21.3
208894_at	14	HLA-DRA	M60334	6p21.3
210982_s_at	13	HLA-DRA	M60333	6p21.3
209312_x_at	15	HLA-DRB1	U65585	6p21.3
215193_x_at	15	HLA-DRB1	AJ297586	6p21.3
221491_x_at	10	HLA-DRB3	U66825	6p21.3
208306_x_at	15	HLA-DRB4	NM_021983	6p21.3
204670_x_at	15	HLA-DRB5	NM_002125	6p21.3
200904_at	15	HLA-E	X56841	6p21.3
200905_x_at	15	HLA-E	NM_005516	6p21.3
204806_x_at	15	HLA-F	NM_018950	6p21.3
221875_x_at	15	HLA-F	AW514210	6p21.3
221978_at	14	HLA-F	BE138825	6p21.3
222279_at	15	HLA-F	AI669379	6p21.3
211529_x_at	15	HLA-G	M90684	6p21.3
211528_x_at	15	HLA-G2.2	M90685	6p21.3
200878_at	15	HLF	AF052094	2p21-p16
208141_s_at	15	HLRC1	NM_031304	19p13.3
211593_s_at	15	hMAST205	AB047005	1p34.1 /// 1p34.1

203040_s_at	15	HMBS	NM_000190	11q23.3
208668_x_at	15	HMG17	BC003689	1p36.1
208980_s_at	15	HMG20	M26880	12q24.3
218152_at	15	HMG20A	NM_018200	15q24
210719_s_at	15	HMG20B	BC002552	19p13.3
65133_i_at	12	HMGA1L4	AI862454	2p13.1
208025_s_at	14	HMGA2	NM_003483	12q15 /// 12q15
213486_at	10	HMGA2	BF435376	12q15
200679_x_at	15	HMGB1	NM_002128	13q12
200680_x_at	15	HMGB1	NM_002128	13q12
214938_x_at	15	HMGB1	AF283771	13q12
208808_s_at	15	HMGB2	BC000903	4q31
203744_at	15	HMGB3	NM_005342	Xq28
212596_s_at	15	HMGBCG; THC211630	AJ010070	22q13.1
212597_s_at	15	HMGBCG; THC211630	AL079310	22q13.1
202772_at	15	HMGCL	NM_000191	1p36.1-p35
202539_s_at	12	HMGCR	NM_000859	5q13.3-q14
202540_s_at	13	HMGCR	NM_000859	5q13.3-q14
221750_at	15	HMGCS1	BG035985	---
200943_at	15	HMGN1	NM_004965	21q22.3
200944_s_at	15	HMGN1	NM_004965	21q22.3
202579_x_at	15	HMGN4	NM_006353	6p21.3
209786_at	15	HMGN4	BC001282	6p21.3
209787_s_at	15	HMGN4	BC001282	6p21.3
209035_at	15	hMK-1	M69148	11p11.2
207165_at	15	HMMR	NM_012485	5q33.2-qter
203665_at	14	HMOX1	NM_002133	22q12
218120_s_at	15	HMOX2	NM_002134	16p13.3
209421_at	15	hMSH2	U04045	2p22-p21
204112_s_at	15	HNMT	NM_006895	2q22.1
211732_x_at	13	HNMT	BC005907	2q22.1 /// 2q22.1
214280_x_at	15	HNRNPA1; MGC102835	X79536	12q13.1
211930_at	15	hnRNPA3	AW080932	2q31.2
201054_at	15	HNRPA0	BE966599	5q31
201055_s_at	15	HNRPA0	NM_006805	5q31
200016_x_at	15	HNRPA1	NM_002136	12q13.1
213356_x_at	15	HNRPA1	AL568186	12q13.1
221919_at	15	HNRPA1	AW450929	12q13.1
222040_at	15	HNRPA1	AI144007	12q13.1
205292_s_at	15	HNRPA2B1	NM_002137	7p15
206809_s_at	15	HNRPA3	NM_005758	---
211929_at	15	HNRPA3	BF195526	2q31.2
211931_s_at	15	HNRPA3	BG505670	Xq27.1
211932_at	15	HNRPA3	BF195526	2q31.2
211933_s_at	15	HNRPA3	BF195526	2q31.2
201277_s_at	15	HNRPAB	NM_004499	5q35.3
200014_s_at	15	HNRPC	NM_004500	14q11.2
200751_s_at	15	HNRPC	BE898861	14q11.2
212626_x_at	15	HNRPC	AA664258	14q11.2
214737_x_at	15	HNRPC	AV725195	14q11.2

200073_s_at	15	HNRPD	M94630	4q21.1-q21.2
209330_s_at	15	HNRPD	D55674	4q21.1-q21.2
213359_at	15	HNRPD	W74620	4q21.1-q21.2
221480_at	13	HNRPD	D55672	4q21.1-q21.2
221481_x_at	15	HNRPD	D55672	4q21.1-q21.2
201993_x_at	15	HNRPDL	NM_005463	4q13-q21
209067_s_at	15	HNRPDL	D89092	4q13-q21
209068_at	13	HNRPDL	D89678	4q13-q21
212454_x_at	15	HNRPDL	AI762552	4q13-q21
201376_s_at	12	HNRPF	NM_004966	10q11.21-q11.22
201031_s_at	15	HNRPH1	NM_005520	5q35.3
213619_at	15	HNRPH1	AV753392	5q35.3
201132_at	15	HNRPH2	NM_019597	Xq22
207127_s_at	15	HNRPH3	NM_021644	10q22
208990_s_at	15	HNRPH3	AI912352	10q22
210110_x_at	15	HNRPH3	AF132363	10q22
210588_x_at	15	HNRPH3	L32610	10q22
200097_s_at	15	HNRPK	AI701949	9q21.32-q21.33
200775_s_at	15	HNRPK	BC000355	9q21.32-q21.33
221860_at	15	HNRPL	AL044078	19q13.2
35201_at	15	HNRPL	X16135	19q13.2
208765_s_at	15	HNRPR	NM_005826	1p36.11
208766_s_at	15	HNRPR	BC001449	1p36.11
200593_s_at	15	HNRPU	BC003621	1q44
200594_x_at	15	HNRPU	NM_004501	1q44
208713_at	15	HNRPUL1	BF724216	19q13.31
209675_s_at	10	HNRPUL1	BC004242	19q13.31
209252_at	14	HO3	U18937	5q31.3
201972_at	15	HO68	AF113129	3q13.31
213793_s_at	15	HOMER1	BE550452	5q14.2
204647_at	15	HOMER3	NM_004838	19p13.11
215489_x_at	15	HOMER3	AI871287	19p13.11
209721_s_at	15	HOM-TES-103	BC002857	12p13.3
36030_at	15	HOM-TES-103	AL080214	12p13.3
211597_s_at	15	HOP	AB059408	4q11-q12
216071_x_at	15	HOPA	AF132033	Xq13
206848_at	13	HOXA7	NM_006896	7p15-p14
206194_at	10	HOXC4	AW299598	12q13.3
210762_s_at	15	HP	AF026219	8p22
209715_at	15	HP1	L07515	12q13.13
209039_x_at	14	HPAST	AF001434	11q13
205462_s_at	10	HPCAL1	NM_002149	2p25.1
212552_at	14	HPCAL1	BE617588	2p25.1
221645_s_at	15	HPF1	M27877	19q13.3
203914_x_at	10	HPGD	NM_000860	4q34-q35
217485_x_at	15	hPMS3	D38435	7q22.1
216525_x_at	15	hPMS5	D38437	7q11.23
216843_x_at	10	hPMSR2	U38964	---
216111_x_at	14	hPMSR3	U38979	---
179_at	10	hPMSR6	U38980	---

210707_x_at	13	hPMSR6	U38980	7
202854_at	15	HPRT1	NM_000194	Xq26.1
203309_s_at	11	HPS1	NM_000195	10q23.1-q23.3
204544_at	15	HPS5	NM_007216	11p14
212983_at	15	HRAS	NM_005343	11p15.5
219983_at	15	HRASLS	NM_020386	3q29
219984_s_at	15	HRASLS	NM_020386	3q29
209581_at	14	HRASLS3	BC001387	11q13.1
218091_at	15	HRB	NM_004504	2q36.3
218092_s_at	15	HRB	NM_004504	2q36.3
203202_at	15	HRB2	AI950314	12q21.1
207066_at	15	HRC	NM_002152	19q13.3
37170_at	10	HRIHFB2017	AB015331	4q21.23
216210_x_at	15	HRIHFB2122	AA046650	22q13.1
206864_s_at	12	HRK	NM_003806	12q24.23
202098_s_at	15	HRMT1L1	NM_001535	21q22.3
221564_at	15	HRMT1L1	AL570294	21q22.3
206445_s_at	15	HRMT1L2	NM_001536	19q13.3
207563_s_at	15	HRNT1	U77413	Xq13
209240_at	15	HRNT1	AF070560	Xq13
209526_s_at	15	HRP-3	AB029156	15q11.2
210428_s_at	15	HRS	AF260566	17q25
203790_s_at	15	HRSP12	N54448	8q22
216976_s_at	15	H-RYK	X96588	3q22
219020_at	15	HS1BP3	NM_022460	2p24.2
203284_s_at	15	HS2ST1	AW151887	1p31.1-p22.1
203285_s_at	15	HS2ST1	NM_012262	1p31.1-p22.1
205466_s_at	11	HS3ST1	BF000296	4p16
218896_s_at	15	HSA277841	NM_018553	17p13.3
204405_x_at	15	HSA9761	NM_014473	5q11-q14
217106_x_at	15	HSA9761	AF091078	5q11-q14
200942_s_at	15	HSBP1	NM_001537	16q23.3
212817_at	15	Hsc40; KIAA1045	AK023253	9p13.2
210338_s_at	15	HSC54	AB034951	11q24.1
208687_x_at	15	HSC70	AF352832	11q24.1
219304_s_at	15	hSCDGF-B	AB033832	11q22.3
205404_at	11	HSD11B1	NM_005525	1q32-q41
217869_at	15	HSD17B12	NM_016142	11p11.2
201413_at	15	HSD17B4	NM_000414	5q21
220081_x_at	14	HSD17B7	NM_016371	1q23
213540_at	15	HSD17B8	AL031228	6p21.3
209512_at	15	HSDL2	BC004331	9q33.1
209513_s_at	15	HSDL2	BC004331	9q33.1
202344_at	15	HSF1	NM_005526	8q24.3
211220_s_at	10	HSF2	BC005329	6q22.32
216205_s_at	15	HSG	AK021947	1p36.21
203013_at	15	HSGT1	NM_007265	10q22.3
209339_at	15	hSiah2	U76248	3q25
208810_at	15	HSJ2	AF080569	7q36.3
208811_s_at	15	HSJ2	AF080569	7q36.3

211251_x_at	15	HSM	U78774	1p32
211797_s_at	13	HSM	U62296	1p32
221771_s_at	14	HSMPP8	BC003542	13q12.11
208739_x_at	15	HSMT3	L76416	17q25
208738_x_at	15	HSMT3	AK024823	17q25
209481_at	15	HSNFRK	AF226044	3p21.32
200064_at	15	HSP90BETA	AF275719	6p12
219212_at	14	HSPA14	NM_016299	10p14
200799_at	15	HSPA1A	NM_005345	6p21.3
200800_s_at	15	HSPA1A	NM_005345	6p21.3
202581_at	15	HSPA1B	NM_005346	6p21.3
211538_s_at	15	HSPA2	U56725	---
211015_s_at	15	HSPA4	L12723	5q31.1-q31.2
213564_x_at	15	HSPA8	BE042354	12p12.2-p12.1
221891_x_at	15	HSPA8	AA704004	11q24.1
200690_at	14	HSPA9B	AA927701	5q31.1
200691_s_at	15	HSPA9B	NM_004134	5q31.1
200692_s_at	15	HSPA9B	NM_004134	5q31.1
201841_s_at	15	HSPB1	NM_001540	7q11.23
205824_at	15	HSPB2	NM_001541	11q22-q23
206375_s_at	15	HSPB3	NM_006308	5q11.2
214767_s_at	12	HSPB6	AL551046	19q13.13
218934_s_at	15	HSPB7	NM_014424	1p36.23-p34.3
219284_at	11	HSPBAP1	NM_024610	3q21.1
202415_s_at	15	HSPBP1	NM_012267	19q13.42
218026_at	15	HSPC009	NM_014019	17q21
221791_s_at	15	HSPC016	BG167522	3p21.31
217926_at	15	HSPC023	NM_014047	19p13.13
211406_at	15	HSPC039; PRO2309	AF119875	18q12
218971_s_at	11	HSPC049	AK001606	7q33
203023_at	15	HSPC111	NM_016391	5q35.3
214011_s_at	15	HSPC111	BE314601	5q35.3
210829_s_at	15	HSPC116	AF077048	5q14.1
200042_at	15	HSPC117	NM_014306	22q12
218936_s_at	15	HSPC128	NM_014167	12q21.31
221046_s_at	15	HSPC135	NM_014170	3q13.2
219979_s_at	15	HSPC138	NM_016401	11q14.1
221711_s_at	15	HSPC142	BC006244	19p13.12
217774_s_at	15	HSPC152	NM_016404	11q13.1
218728_s_at	15	HSPC163	AK024569	1q42.12
221597_s_at	15	HSPC171	BC003080	16q22.1
218354_at	15	HSPC176	NM_016209	16q24.3
209020_at	15	HSPC207	AF217514	20q13.11
213031_s_at	15	HSPC264; FLJ14888	AF161382	15q25.2
211968_s_at	15	HSPCA	AI962933	14q32.33
211969_at	15	HSPCA	BG420237	14q32.33
214359_s_at	15	HSPCB	AI218219	6p12
200806_s_at	15	HSPD1	BE256479	---
200807_s_at	15	HSPD1	NM_002156	2q33.1
205133_s_at	15	HSPE1	NM_002157	2q33.1

201655_s_at	15	HSPG2	AI991033	1p36.1-p35
206976_s_at	15	HSPH1	NM_006644	13q12.3
208744_x_at	15	HSPH1	D86956	13q12.3
211089_s_at	12	HSPK36	Z25434	13q14.13
210211_s_at	15	HSPN	AF028832	14q32.33
204521_at	15	HSU79274	NM_013300	12q24.13
221622_s_at	15	HT007; MGC111203	AF246240	11q14.1
220703_at	15	HT009	NM_018470	10p15.3
206689_x_at	13	HTATIP	NM_006388	11q13
214258_x_at	11	HTATIP	AA886971	11q13
209448_at	11	HTATIP2	BC002439	11p15.1
202602_s_at	15	HTATSF1	NM_014500	Xq26.1-q27.2
209586_s_at	15	HTCD37; DRES-17	AF123539	1q21
210988_s_at	15	HTCD37; DRES-17	AF123538	1q21
200072_s_at	15	HTGR1	AF061832	19p13.3-p13.2
215000_s_at	15	HUM3CL	AL117593	2p21 /// 2p21
213951_s_at	13	HUMGT198A	BE964655	---
204883_s_at	10	HUS1	NM_004507	7p13-p12
207783_x_at	15	HUWE1	NM_017627	13q12-q14
208598_s_at	15	HUWE1	NM_005703	---
210849_s_at	15	HVPS41	AF135593	7p14-p13
206855_s_at	15	HYAL2	NM_003773	3p21.3
221435_x_at	15	HYI	NM_031207	1p34.1 /// 1p34.1
200825_s_at	15	HYOU1	NM_006389	11q23.1-q23.3
212493_s_at	15	HYPB	AI761110	3p21.31
215038_s_at	15	HYPB	AF049103	3p21.31
219910_at	14	HYPE	NM_007076	12q24.1
218680_x_at	15	HYPK	NM_016400	15q15.1
211009_s_at	15	HZF7; ZNFEB	AF159567	18q12
211075_s_at	15	IAP	Z25521	3q13.1-q13.2
204744_s_at	15	IARS	NM_013417	9q21
217900_at	15	IARS2	NM_018060	1q42.11
36564_at	14	IBRDC3	W27419	1p34.3
211684_s_at	15	IC2	AF250307	2q31.1
38918_at	14	ICA12	AF083105	1q32
210547_x_at	14	ICA69; ICAp69	L21181	7p22
202638_s_at	12	ICAM1	NM_000201	19p13.3-p13.2
204683_at	15	ICAM2	NM_000873	17q23-q25
213620_s_at	15	ICAM2	AA126728	17q23-q25
204949_at	14	ICAM3	NM_002162	19p13.3-p13.2
210785_s_at	13	ICB-1	AB035482	1p35.3
212959_s_at	15	ICD	AK001821	12q23.3
209668_x_at	15	iCE; CE-2	D50579	16q22.1
219122_s_at	15	ICF45	NM_017872	5q33.3
201609_x_at	15	ICMT	AL578502	1p36.21
201611_s_at	15	ICMT	AL578502	1p36.21
204868_at	15	ICT1	NM_001545	17q25.2
208937_s_at	15	ID1	D13889	20q11
201565_s_at	15	ID2	NM_002166	2p25
201566_x_at	15	ID2	NM_002166	2p25

213931_at	15	ID2	AI819238	---
207826_s_at	15	ID3	NM_002167	1p36.13-p36.12
209291_at	15	ID4	AW157094	6p22-p21
209292_at	14	ID4	AL022726	6p22-p21
209293_x_at	15	ID4	U16153	6p22-p21
203327_at	15	IDE	N22903	10q23-q25
203328_x_at	12	IDE	NM_004969	10q23-q25
201193_at	15	IDH1	NM_005896	2q33.3
210045_at	11	IDH2	AU151428	15q26.1
210046_s_at	15	IDH2	U52144	15q26.1
202069_s_at	15	IDH3A	NM_005530	15q25.1-q25.2
202070_s_at	15	IDH3A	NM_005530	15q25.1-q25.2
201509_at	15	IDH3B	NM_006899	20p13
210014_x_at	15	IDH3B	AF023266	20p13
210418_s_at	15	IDH3B	AF023265	20p13 /// 20p13
202471_s_at	15	IDH3G	NM_004135	Xq28
204615_x_at	15	IDI1	NM_004508	10p15.3
208881_x_at	15	IDI1	BC005247	10p15.3
212483_at	15	IDN3	AB019494	5p13.2
202439_s_at	15	IDS	NM_000202	Xq28
206342_x_at	14	IDS	NM_006123	Xq28
212221_x_at	15	IDS	AV703259	Xq28
212223_at	15	IDS	AV703259	Xq28
217432_s_at	13	IDS	AF179281	Xq28
202081_at	15	IER2	NM_004907	19p13.13
201631_s_at	15	IER3	NM_003897	6p21.3
218611_at	15	IER5	NM_016545	1q25.1
203854_at	15	IF	NM_000204	4q25
201025_at	15	IF2	AB018284	2p11.1-q11.1
206332_s_at	15	IFI16	NM_005531	1q22
208965_s_at	15	IFI16	BG256677	1q22
208966_x_at	15	IFI16b	AF208043	1q22
202411_at	15	IFI27	NM_005532	14q32
201422_at	15	IFI30	NM_006332	19p13.1
209417_s_at	13	IFI35	BC001356	17q21
214453_s_at	14	IFI44	NM_006417	1p31.1
203153_at	15	IFIT1	NM_001548	10q25-q26
204747_at	13	IFIT3	NM_001549	10q24
201601_x_at	15	IFITM1	NM_003641	11p15.5
214022_s_at	15	IFITM1	AA749101	11p15.5
201315_x_at	15	IFITM2	NM_006435	11p15.5
212203_x_at	15	IFITM3	BF338947	11p15.5
216565_x_at	15	IFITM3	AL121994	---
208448_x_at	13	IFNA16	NM_002173	9p22
204786_s_at	10	IFNAR2	L41944	21q22.1
202727_s_at	15	IFNGR1	NM_000416	6q23-q24
201642_at	15	IFNGR2	NM_005534	21q22.11
202146_at	15	IFRD1	AA747426	7q22-q31
209100_at	15	IFRD2	BC001327	3p21.3
210312_s_at	15	IFT20	BC002640	17q11.2

218709_s_at	15	IFT52	NM_016004	---
218100_s_at	15	IFT57	NM_018010	3q13.13
219174_at	13	IFT74	NM_025103	9p21.1
219372_at	15	IFT81	NM_014055	12q24.13
204703_at	15	IFT88	NM_006531	13q12.1
202105_at	15	IGBP1	NM_001551	Xq13.1-q13.3
209540_at	11	IGF1	M29644	12q22-q23
209541_at	15	IGF1	AI972496	12q22-q23
203627_at	15	IGF1R	AI830698	15q25-q26
203628_at	14	IGF1R	H05812	15q25-q26
202410_x_at	14	IGF2	NM_000612	11p15.5
210881_s_at	10	IGF2	M17863	11p15.5
201392_s_at	15	IGF2R	BG031974	6q26
201393_s_at	15	IGF2R	NM_000876	6q26
210095_s_at	15	IGFBP1	M31159	7p13-p12
202718_at	15	IGFBP2	NM_000597	2q33-q34
212143_s_at	15	IGFBP3	BF340228	7p13-p12
201508_at	15	IGFBP4	NM_001552	17q12-q21.1
203425_s_at	15	IGFBP5	M65062	2q33-q36
211958_at	15	IGFBP5	R73554	2q33-q36
211959_at	15	IGFBP5	AW007532	2q33-q36
203851_at	15	IGFBP6	NM_002178	12q13
201162_at	15	IGFBP7	NM_001553	4q12
201163_s_at	15	IGFBP7	NM_001553	4q12
213910_at	12	IGFBP7	AW770896	4q12
202421_at	15	IGSF3	AB007935	1p13
209030_s_at	15	IGSF4	NM_014333	11q23.2
209031_at	15	IGSF4	AL519710	11q23.2
209818_s_at	14	IHABP4	AF241831	9q22.3-q31
212439_at	13	IHPK1	BE614199	3p21.31
218192_at	15	IHPK2	NM_016291	3p21.31
202491_s_at	15	IKBKAP	NM_003640	9q31
209341_s_at	13	IKBKB	AU153366	8p11.2
209666_s_at	12	IKK-alpha	AF080157	10q24-q25
209575_at	15	IL10RB	BC001903	21q22.1-q22.2
204773_at	15	IL11RA	NM_004512	9p13
201887_at	15	IL13RA1	NM_001560	Xq24
211612_s_at	14	IL13RA1	U62858	---
205992_s_at	15	IL15	NM_000585	4q31
64440_at	12	IL17RC	AI560217	3p25.3
209970_x_at	15	IL1BCE	M87507	11q23
211366_x_at	15	IL1BCE	U13698	11q23
202948_at	15	IL1R1	NM_000877	2q12
220663_at	13	IL1RAPL1	NM_014271	Xp22.1-p21.3
216483_s_at	15	IL25	AC005339	---
203828_s_at	14	IL32	NM_004221	16p13.3
205207_at	13	IL6	NM_000600	7p21
212195_at	15	IL6ST	AL049265	5q11
212196_at	15	IL6ST	AW242916	5q11
202859_x_at	13	IL8	NM_000584	4q13-q21

200052_s_at	15	ILF2	NM_004515	1q22
208930_s_at	12	ILF3	BG032366	19p13.2
217804_s_at	15	ILF3	NM_004516	19p13.2
217805_at	15	ILF3	NM_004516	19p13.2
201234_at	15	ILK	NM_004517	11p15.5-p15.4
202993_at	15	ILVBL	NM_006844	19p13.1
210624_s_at	15	ILVBL	BC000109	19p13.1
211952_at	14	IMB3	AF339834	13q32.2
200955_at	15	IMMT	NM_006839	2p11.2
218847_at	15	IMP-2	NM_006548	3q28
203819_s_at	15	IMP-3	AU160004	7p11
203820_s_at	15	IMP-3	NM_006547	7p11
212411_at	13	IMP4	BE747342	2q21.2
203011_at	15	IMPA1	NM_005536	8q21.13-q21.3
203126_at	14	IMPA2	NM_014214	18p11.2
218637_at	11	IMPACT	NM_018439	18q11.2-q12.1
201892_s_at	15	IMPDH2	NM_000884	3p21.2
208415_x_at	12	ING1	NM_005537	13q34
209808_x_at	14	ING1	AW193656	13q34
205981_s_at	15	ING2	NM_001564	4q35.1
205070_at	15	ING3	NM_019071	7q31
48825_at	14	ING4	AA887083	12p13.31
207687_at	11	INHBC	NM_005538	12q13.1
207688_s_at	15	INHBC	NM_005538	12q13.1
202794_at	15	INPP1	NM_002194	2q32
204552_at	13	INPP4A	AA355179	2q11.2
205376_at	15	INPP4B	NM_003866	4q31.1
203006_at	15	INPP5A	NM_005539	10q26.3
204706_at	13	INPP5E	NM_019892	9q34.3
203607_at	15	INPP5F	NM_014937	10q26.13
210612_s_at	11	INPP5H	AF318616	6q25.3
201598_s_at	13	INPPL1	NM_001567	11q23
201625_s_at	11	INSIG1	BE300521	7q36
201626_at	15	INSIG1	BG292233	7q36
201627_s_at	15	INSIG1	BE300521	7q36
213792_s_at	15	INSR	AA485908	19p13.3-p13.2
210114_at	10	INVS	AF039217	9q31
203546_at	15	IPO13	NM_014652	1p34.1
218305_at	10	IPO4	NM_024658	14q11.2
200992_at	15	IPO7	AL137335	11p15.3
200993_at	15	IPO7	AA939270	11p15.3
200994_at	15	IPO7	BG291787	11p15.3
200995_at	15	IPO7	AI741392	11p15.3
217885_at	15	IPO9	NM_018085	1q32.1
216841_s_at	15	IPO-B	X15132	6q25.3
219092_s_at	13	IPPK	NM_022755	9q21.33-q22.31
211707_s_at	12	IQCB1	BC005806	3q21.1
204202_at	11	IQCE	NM_017604	7p22.3
214739_at	13	IQCG	AI357539	3q29
221185_s_at	12	IQCG	NM_025111	3q29

200791_s_at	15	IQGAP1	NM_003870	15q26.1
210840_s_at	15	IQGAP1	D29640	15q26.1
213446_s_at	11	IQGAP1	AI679073	15q26.1
203474_at	12	IQGAP2	NM_006633	5q13.3-q14.1
203906_at	13	IQSEC1	AI652645	3p25.2
203907_s_at	14	IQSEC1	AI652645	3p25.2
213129_s_at	15	IQWD1	AI970157	---
217908_s_at	15	IQWD1	NM_018442	1q23.3
201587_s_at	15	IRAK1	NM_001569	Xq28
202531_at	11	IRF1	NM_002198	5q31.1
203275_at	15	IRF2	NM_002199	4q34.1-q35.1
202621_at	15	IRF3	NM_001571	19q13.3-q13.4
204562_at	11	IRF4	NM_002460	6p25-p23
202597_at	13	IRF6	AU144284	1q32.3-q41
208436_s_at	15	IRF7	NM_004030	11p15.5
204057_at	11	IRF8	AI073984	16q24.1
204686_at	15	IRS1	NM_005544	2q36
209184_s_at	14	IRS2	AF073310	13q34
209185_s_at	15	IRS2	AF073310	13q34
210239_at	14	IRX-2a	U90304	16q11.2-q13
220225_at	15	IRX4	NM_016358	5p15.3
209075_s_at	15	ISCU	AY009128	12q24.1
208114_s_at	15	ISG20L2	NM_030980	1q23.1
203882_at	15	ISGF3G	NM_006084	14q11.2
207191_s_at	15	ISLR	NM_005545	15q23-q24
218170_at	15	ISOC1	NM_016048	5q22.1-q33.3
222240_s_at	15	ISYNA1	AL137749	19p13.11
209744_x_at	14	Itch	AB056663	20q11.22-q11.23
206766_at	15	ITGA10	AF112345	1q21
206493_at	14	ITGA2B	NM_000419	17q21.32
201389_at	15	ITGA5	NM_002205	12q11-q13
201656_at	15	ITGA6	NM_000210	2q31.1
215177_s_at	11	ITGA6	AV733308	2q31.1
209663_s_at	15	ITGA7	AF072132	12q13
216331_at	15	ITGA7	AK022548	12q13
205055_at	15	ITGAE	NM_002208	17p13
202351_at	15	ITGAV	AI093579	2q31-q32
211945_s_at	15	ITGB1	BG500301	10p11.2
216178_x_at	15	ITGB1	AA215854	10p11.2
203336_s_at	15	ITGB1BP1	AL548363	2p25.2
203337_x_at	12	ITGB1BP1	NM_004763	2p25.2
219829_at	15	ITGB1BP2	NM_012278	Xq12-q13.1
221051_s_at	15	ITGB1BP3	NM_014446	19p13.3
202803_s_at	13	ITGB2	NM_000211	21q22.3
204628_s_at	15	ITGB3	NM_000212	17q21.32
216261_at	10	ITGB3	AI151479	17q21.32
205176_s_at	15	ITGB3BP	NM_014288	1p31.3
201124_at	15	ITGB5	AL048423	3q21.2
201125_s_at	15	ITGB5	NM_002213	3q21.2
214020_x_at	11	ITGB5	AI335208	3q21.2

202746_at	15	ITM2A	AL021786	Xq13.3-Xq21.2
202747_s_at	15	ITM2A	NM_004867	Xq13.3-Xq21.2
217731_s_at	15	ITM2B	NM_021999	13q14.3
217732_s_at	15	ITM2B	NM_021999	13q14.3
221004_s_at	15	ITM2C	NM_030926	2q37 /// 2q37
209171_at	13	ITPA	AF219116	20p
203723_at	14	ITPKB	NM_002221	1q42.13
213076_at	15	ITPKC	D38169	19q13.1
203710_at	15	ITPR1	NM_002222	3p26-p25
216944_s_at	11	ITPR1	U23850	3p26-p25
210740_s_at	15	ITRPK1	AF279372	14q31
209297_at	11	ITSN	AF114488	21q22.1-q22.2
209298_s_at	13	ITSN	AF114488	21q22.1-q22.2
35776_at	15	ITSN	AF064243	21q22.1-q22.2
209907_s_at	15	ITSN2	AF182198	2pter-p25.1
203682_s_at	12	IVD	NM_002225	15q14-q15
216958_s_at	10	IVD	AK022777	15q14-q15
201363_s_at	15	IVNS1ABP	AB020657	1q25.1-q31.1
206245_s_at	15	IVNS1ABP	NM_006469	1q25.1-q31.1
209784_s_at	15	JAG2	AF029778	14q32
32137_at	15	jag2	Y14330	14q32 /// 14q32
201648_at	15	JAK1	AL039831	1p31.3
205841_at	14	JAK2	NM_004972	9p24
205842_s_at	14	JAK2	AF001362	9p24
219213_at	15	JAM2	NM_021219	21q21.2
212813_at	15	JAM3	AA149644	11q25
202040_s_at	15	JARID1A	NM_005056	12p11
215698_at	13	JARID1A	AF007135	12p11
201548_s_at	15	JARID1B	W02593	1q32.1
201549_x_at	15	JARID1B	NM_006618	1q32.1
203297_s_at	15	JARID2	BG029530	6p24-p23
216309_x_at	10	JH8	AF072467	8q24.3
37872_at	12	JH8	AF072468	8q24.3
213971_s_at	14	JJAZ1	AI924660	---
212689_s_at	15	JMJD1A	AA524505	2p11.2
201643_x_at	15	JMJD1B	NM_016604	5q31
210878_s_at	15	JMJD1B	BC001202	5q31
221763_at	15	JMJD1C	AI694023	10q22.1
203205_at	13	JMJD2A	NM_014663	1p34.1
212492_s_at	15	JMJD2B	AW237172	19p13.3
212495_at	10	JMJD2B	AW237172	19p13.3
212496_s_at	11	JMJD2B	AW237172	19p13.3
213146_at	10	JMJD3	AA521267	17p13.1
204813_at	14	JNK3A2	U34819	4q22.1-q23
201751_at	15	JOSD1	NM_014876	22q13.1
218750_at	15	JOSD3	NM_024116	11q21
221580_s_at	15	JOSD3	BC001972	11q21
206734_at	15	JRKL	NM_003772	11q21
200048_s_at	15	JTB	NM_006694	1q21
210927_x_at	15	JTB	BC004239	1q21

210754_s_at	14	JTK8	M79321	8q13
202138_x_at	15	JTV1	NM_006303	7p22
209971_x_at	14	JTV1	AI928526	7p22
213281_at	15	JUN	BE327172	1p32-p31
201473_at	15	JUNB	NM_002229	19p13.2
203752_s_at	15	JUND	NM_005354	19p13.2
201015_s_at	15	JUP	NM_021991	17q21
213287_s_at	15	K10	X14487	17q21
209008_x_at	15	K8	U76549	12q13
207719_x_at	15	KAB	NM_014812	1q44
212746_s_at	15	KAB	AA126789	1q44
205206_at	15	KAL1	NM_000216	Xp22.32
201090_x_at	15	K-ALPHA-1	NM_006082	12q13.12
211058_x_at	15	K-ALPHA-1	BC006379	12q13.12
211072_x_at	15	K-ALPHA-1	BC006481	12q13.12
212639_x_at	15	K-ALPHA-1	AL581768	---
213646_x_at	15	K-ALPHA-1	BE300252	12q13.12
209714_s_at	15	KAP	AF213033	14q22
200840_at	15	KARS	NM_005548	16q23-q24
200079_s_at	15	KARS2; KIAA0070	AF285758	16q23-q24
209472_at	15	KAT3	BC000819	1p22.2
205526_s_at	15	KATNA1	NM_007044	6q24.3
203162_s_at	15	KATNB1	NM_005886	16q13
219106_s_at	15	KBTBD10	AI126808	2q31.1
218570_at	14	KBTBD4	NM_018095	11p11.2
217938_s_at	15	KCMF1	AI743396	2p11.2
207248_at	10	KCNA4	NM_002233	11p14
206762_at	14	KCNA5	NM_002234	12p13
210078_s_at	11	KCNAB1	L39833	3q26.1
210471_s_at	13	KCNAB1	U33428	3q26.1
208514_at	12	KCNE1	NM_000219	21q22.1-q22.2
205262_at	14	KCNH2	NM_000238	7q35-q36
206765_at	15	KCNJ2	AF153820	17q23.1-q24.2
208359_s_at	14	KCNJ4	NM_004981	22q13.1
208404_x_at	10	KCNJ5	NM_000890	11q24
205303_at	15	KCNJ8	BF514158	12p11.23
205304_s_at	15	KCNJ8	BF514158	12p11.23
204679_at	14	KCNK1	NM_002245	1q42-q43
205952_at	11	KCNK3	NM_002246	2p23
219287_at	15	KCNMB4	AF160967	12q
220116_at	12	KCNN2	NM_021614	5q22.3
212188_at	15	KCTD12	AA551075	13q22.1
212192_at	15	KCTD12	AI718937	13q22.1
218553_s_at	14	KCTD15	NM_024076	19q13.12
34858_at	15	KCTD2	D79998	17q25.2
217894_at	15	KCTD3	NM_016121	1q41
218474_s_at	13	KCTD5	NM_018992	16p13.3
213474_at	15	KCTD7	AI890903	7q11.21
218823_s_at	15	KCTD9	NM_017634	8p21.1
219479_at	14	KDELC1	NM_024089	13q33

200922_at	15	KDELR1	NM_006801	19q13.3
200698_at	15	KDELR2	AL542253	7p22.2
200699_at	15	KDELR2	AL542253	7p22.2
200700_s_at	15	KDELR2	NM_006854	7p22.2
204017_at	15	KDELR3	NM_006855	22q13.1
207265_s_at	12	KDELR3	NM_016657	22q13.1
203934_at	15	KDR	NM_002253	4q11-q12
202417_at	13	KEAP1	NM_012289	19p13.2
221838_at	14	KELCHL	N38751	22q11.21
49329_at	15	KELCHL	N38751	22q11.21
209863_s_at	12	KET	AF091627	3q27-q29
200040_at	15	KHDRBS1	NM_006559	1p32
201488_x_at	15	KHDRBS1	NM_006559	1p32
214185_at	15	KHDRBS1	AW592227	1p32
204372_s_at	15	KHSRP	AI933301	19p13.3
212303_x_at	12	KHSRP	BG026366	19p13.3
209539_at	15	KIAA0006	D25304	Xq26
214662_at	14	KIAA0007	D26488	2p23.3
32062_at	12	KIAA0014	D25216	8q24.3
212622_at	15	KIAA0033	AU153138	11p15.3
212623_at	15	KIAA0033	AU153138	11p15.3
212896_at	15	KIAA0052	D29641	5q11.2
212789_at	15	KIAA0056	AI796581	11q25
213483_at	15	KIAA0073	AK025679	5q12.3
36084_at	15	KIAA0076	D38548	6p21.1
212380_at	15	KIAA0082	D43949	6p21.2
201729_s_at	13	KIAA0100	NM_014680	17q11.2
202503_s_at	15	KIAA0101	NM_014736	15q22.1
211713_x_at	13	KIAA0101	BC005832	15q22.1
203584_at	15	KIAA0103	NM_014673	8q23.2
212591_at	15	KIAA0117	AA887480	---
212399_s_at	15	KIAA0121	D50911	3p25.2
212414_s_at	15	KIAA0128	D50918	Xq25
201977_s_at	15	KIAA0141	AI539425	5q31.3
212149_at	15	KIAA0143	AW470003	8q24.22
212150_at	15	KIAA0143	AA805651	8q24.22
212523_s_at	13	KIAA0146	D63480	8q11.21
200616_s_at	14	KIAA0152	BC000371	12q24.31
200617_at	15	KIAA0152	NM_014730	12q24.31
216251_s_at	15	KIAA0153	BF965437	22q13.31
212835_at	13	KIAA0157	AA777641	10q26.2
212837_at	14	KIAA0157	D63877	10q26.2
200851_s_at	15	KIAA0174	NM_014761	16q22.2
212846_at	14	KIAA0179	AA811192	21q22.3
212056_at	15	KIAA0182	D80004	16q24.1
212057_at	15	KIAA0182	AA206161	16q24.1
34221_at	15	KIAA0194	D83778	5q33.1
202650_s_at	15	KIAA0195	NM_014738	17q25.3
201985_at	15	KIAA0196	NM_014846	8p22
208999_at	15	KIAA0202	D86957	5q31

208952_s_at	15	KIAA0217	AU154358	10p15.3
208953_at	15	KIAA0217	AU154358	10p15.3
221992_at	10	KIAA0220	AI925734	16q22.3
212733_at	12	KIAA0226	AI798908	3q29
212735_at	14	KIAA0226	AI798908	3q29
212441_at	15	KIAA0232	D86985	4p16.1
38892_at	13	KIAA0240	D87077	6p21.1
212474_at	14	KIAA0241	D87682	7p14.3
202181_at	15	KIAA0247	NM_014734	14q24.1
212053_at	15	KIAA0251; LP8165	AK025504	16p13.13
212301_at	15	KIAA0252	AI192081	15q14
212302_at	15	KIAA0252	AI192081	15q14
212450_at	15	KIAA0256	D87445	15q15.3
212451_at	13	KIAA0256	N52532	15q15.3
203169_at	10	KIAA0258	NM_014785	9pter-p22.1
212264_s_at	15	KIAA0261	BE645850	10q23.31
212267_at	15	KIAA0261	D87450	10q23.31
212145_at	15	KIAA0264	D87453	5q13.2
209254_at	13	KIAA0265	AI808625	7q32.3
209255_at	15	KIAA0265	D87454	7q32.3
210111_s_at	15	KIAA0265	AF277175	7q32.3
212310_at	11	KIAA0268	D87742	1q42.12
203656_at	15	KIAA0274	NM_014845	6q21
212851_at	15	KIAA0276	AA194584	4q12
212855_at	14	KIAA0276	D87466	4q12
212621_at	14	KIAA0286	AB006624	12q13.2
210958_s_at	10	KIAA0303	BC003646	5q13.1
40016_g_at	15	KIAA0303	AB002301	5q13.1
215696_s_at	15	KIAA0310	BC001404	9q34.3
204308_s_at	15	KIAA0329	NM_014844	14q32.33
41386_i_at	15	KIAA0346	AB002344	17p13.1
41387_r_at	13	KIAA0346	AB002344	17p13.1
203288_at	15	KIAA0355	NM_014686	19q13.12
212805_at	10	KIAA0367	AL138349	9q21.31
212428_at	15	KIAA0368	AW001101	9q32
214356_s_at	15	KIAA0368	AI272899	9q32
203048_s_at	15	KIAA0372	NM_014639	5q15
203049_s_at	15	KIAA0372	NM_014639	5q15
205250_s_at	15	KIAA0373	NM_014684	12q21.33
212480_at	10	KIAA0376	AB002374	22q11.23
202713_s_at	15	KIAA0391	AA129755	14q13.2
212319_at	11	KIAA0397	AB007857	17p13.3
36129_at	15	KIAA0397	AB007857	17p13.3
212601_at	14	KIAA0399	AB007859	17p13.3
213300_at	15	KIAA0404	AW168132	11q13.1
212898_at	14	KIAA0406	AB007866	20q12
203171_s_at	14	KIAA0409	NM_015324	11p15.4
213304_at	15	KIAA0423	AI823592	14q21.3
204303_s_at	12	KIAA0427	NM_014772	18q21.1
201854_s_at	14	KIAA0431	AI744148	16q23.2

212215_at	15	KIAA0436	AB007896	2p22.1
32091_at	15	KIAA0446	AB007915	1q23.1
213216_at	12	KIAA0459	AL537463	1p36.13
212553_at	12	KIAA0460	AK026120	1q21.3
203900_at	13	KIAA0467	NM_024547	---
214663_at	12	KIAA0472	AB007941	1q32.1
202860_at	15	KIAA0476	NM_014856	1p36.13-q31.3
203958_s_at	14	KIAA0478	AI557467	1pter-q31.3
202271_at	15	KIAA0483	AB007952	1q42.12
215109_at	10	KIAA0492	R02172	---
201775_s_at	14	KIAA0494	AA676790	1pter-p22.1
201776_s_at	15	KIAA0494	NM_014774	1pter-p22.1
201778_s_at	15	KIAA0494	NM_014774	1pter-p22.1
213340_s_at	15	KIAA0495	AI073551	---
214954_at	10	KIAA0527	BF977837	3p22.3
212943_at	15	KIAA0528	AB011100	12p12.2
36545_s_at	14	KIAA0542	AB011114	22q12.2
212485_at	15	KIAA0553	AU146596	17q21.31
212946_at	15	KIAA0564	AK025432	13q13.3
212675_s_at	15	KIAA0582	AB011154	2p14
205631_at	13	KIAA0586	NM_014749	14q23.1
37232_at	13	KIAA0586	AB011158	14q23.1
212929_s_at	15	KIAA0592	W68158	10q11.1
212348_s_at	15	KIAA0601	AB011173	1p36.11
34406_at	15	KIAA0602	AB011174	14q32.33
206003_at	14	KIAA0635	NM_014645	4q12
212134_at	14	KIAA0638	AB014538	11q23.3
205150_s_at	15	KIAA0644	AV724192	7p15.1
205151_s_at	10	KIAA0644	AV724192	7p15.1
212138_at	15	KIAA0648	AB014548	4p14
212140_at	13	KIAA0648	AB014548	4p14
203955_at	14	KIAA0649	NM_014811	9q34.3
212569_at	14	KIAA0650	AV699744	18p11.31
212577_at	14	KIAA0650	AA868754	18p11.31
212579_at	14	KIAA0650	AB014550	18p11.31
203363_s_at	13	KIAA0652	NM_014741	11p11.2
203364_s_at	14	KIAA0652	NM_014741	11p11.2
212775_at	15	KIAA0657	AI978623	2q36.1
212776_s_at	13	KIAA0657	AI978623	2q36.1
213946_s_at	13	KIAA0657	AI633851	2q36.1
214928_at	14	KIAA0657	AI915513	2q36.1
205787_x_at	13	KIAA0663	AI803216	1q32.1
212456_at	15	KIAA0664	AB014564	17p13.3
215232_at	10	KIAA0672	AK023797	17p12
31826_at	15	KIAA0674	AB014574	9q33.1
212052_s_at	15	KIAA0676	AB014576	5q35.3
34260_at	14	KIAA0683	AB014583	16p13.3
212200_at	15	KIAA0692	AW274877	12q24.33
212201_at	13	KIAA0692	AW274877	12q24.33
213118_at	10	KIAA0701	AL136821	12q23.2-q23.3

212690_at	15	KIAA0725	AB018268	8p11.23
204403_x_at	14	KIAA0738	NM_014719	7q35
210529_s_at	12	KIAA0738	BC000609	7q35
212979_s_at	15	KIAA0738	AW293343	7q35
212981_s_at	14	KIAA0738	BF030508	7q35
212311_at	14	KIAA0746	AA522514	4p15.31
212314_at	15	KIAA0746	AB018289	4p15.31
213099_at	15	KIAA0759	AB018302	14q24.3
36865_at	12	KIAA0759	AB018302	14q24.3
212856_at	11	KIAA0767	AB018310	22q13.31
214961_at	15	KIAA0774	AI818409	13q12.3
212633_at	15	KIAA0776	AL132776	6q16.3
212634_at	15	KIAA0776	AW298092	6q16.3
212840_at	15	KIAA0794	BG339560	3q29
217100_s_at	11	KIAA0794	AK026451	3q29
212882_at	10	KIAA0795	AB018338	3p21.31
213358_at	12	KIAA0802	AB018345	18p11.22
209553_at	15	KIAA0804	BC001001	3q28
212715_s_at	14	KIAA0819	AB020626	22q11.21
209839_at	12	KIAA0820	AL136712	1q24.1
212546_s_at	15	KIAA0826	AI126634	4p12
212548_s_at	14	KIAA0826	BF515124	4p12
212814_at	10	KIAA0828	AB020635	7q32.3
212573_at	15	KIAA0830	AF131747	11q21
204568_at	15	KIAA0831	NM_014924	14q22.2
36888_at	15	KIAA0841	AB020648	19q13.13
212146_at	12	KIAA0842	AB020649	1p36.13
212402_at	14	KIAA0853	BE895685	13q14.11
203321_s_at	15	KIAA0863	AK022688	18q23
203322_at	14	KIAA0863	AU145934	18q23
212975_at	14	KIAA0870	AB020677	8q24.3
215616_s_at	12	KIAA0876	AB020683	19p13.3
212792_at	15	KIAA0877	AB020684	7p14.3-p14.2
212956_at	15	KIAA0882	AI348094	4q31.1
213424_at	13	KIAA0895	AB020702	7p14.1
212653_s_at	15	KIAA0903	AB020710	2p15
210616_s_at	15	KIAA0905	AB020712	4q21.3
202220_at	15	KIAA0907	NM_014949	1q22
215170_s_at	10	KIAA0912	AB020719	15q15.3
212359_s_at	15	KIAA0913	W89120	10q22.3
212503_s_at	15	KIAA0934	N22859	10p15.3
212504_at	13	KIAA0934	N31807	10p15.3
209654_at	15	KIAA0947	BC004902	5p15.32
212908_at	13	KIAA0962	AK022530	1p36.1
212911_at	13	KIAA0962	AB023179	1p36.1
216996_s_at	15	KIAA0971	AK021557	2q34
207705_s_at	15	KIAA0980	NM_025176	20p11.22-p11.1
211383_s_at	14	KIAA0982	AL136827	10p15.3
200907_s_at	15	KIAA0992	AU157932	4q32.3
214672_at	15	KIAA0998	AB023215	14q24.3

204155_s_at	13	KIAA0999	AA044154	11q23.3
213034_at	15	KIAA0999	AB023216	11q23.3
203831_at	15	KIAA1002	NM_014925	12q13.2
213959_s_at	14	KIAA1005	BF515597	16q12.2
212838_at	13	KIAA1010	AB023227	10q24.31
207305_s_at	15	KIAA1012	NM_014939	18q12.1
203678_at	14	KIAA1018	NM_014967	15q13.1
212209_at	15	KIAA1025	AL133033	12q24.22
213478_at	15	KIAA1026	AB028949	1p36.13
212794_s_at	15	KIAA1033	AK001728	12q24.11
212795_at	15	KIAA1033	AL137753	12q24.11
215936_s_at	10	KIAA1033	AK001657	12q24.11
212754_s_at	15	KIAA1040	BG548738	12q14.1
215146_s_at	15	KIAA1043	AB028966	22q12.1
213311_s_at	15	KIAA1049	BF000251	16q24.3
221495_s_at	15	KIAA1049	AF322111	16q24.3
212796_s_at	14	KIAA1055	BF195608	15q24.1
201270_x_at	13	KIAA1068	AB028991	7p13-p12
213153_at	13	KIAA1076	AB028999	12q24.31
212763_at	15	KIAA1078	AW593213	1q32.1
212765_at	15	KIAA1078	AB029001	1q32.1
217196_s_at	14	KIAA1078	AL110158	1q32.1
212325_at	15	KIAA1102	AK026815	4p14
212327_at	15	KIAA1102	AK026815	4p14
212328_at	15	KIAA1102	AB029025	4p14
214098_at	13	KIAA1107	AB029030	1p22.1
212779_at	15	KIAA1109	AB029032	4q28.1
213271_s_at	13	KIAA1117	AB029040	6q15
221868_at	15	KIAA1155	AB032981	2p13.2
222111_at	14	KIAA1164	AU145293	15q21.3
212904_at	15	KIAA1185	AB033011	1p36.32
44822_s_at	14	KIAA1193	AW003889	19p13.3
55872_at	14	KIAA1196	AI493119	20q13.33
212942_s_at	13	KIAA1199	AB033025	15q
221736_at	15	KIAA1219	BG236163	20q12
213974_at	15	KIAA1233	AB033059	15q25.2
213387_at	15	KIAA1240	AB033066	2p24.1
53991_at	13	KIAA1277	AA127623	7q34
212453_at	15	KIAA1279	AB033105	10q22.1
219520_s_at	15	KIAA1280	NM_018458	Xp22.32
220911_s_at	10	KIAA1305	NM_025081	---
222270_at	10	KIAA1387	BG540048	2p16.2
221952_x_at	15	KIAA1393	AB037814	14q23.1
213316_at	15	KIAA1462	AL050154	---
212802_s_at	15	KIAA1521	AK023841	9q34.11
214869_x_at	15	KIAA1521	AK021533	9q34.11
207765_s_at	12	KIAA1539	NM_025182	9p13.2
65438_at	11	KIAA1609	AA195124	16q24.1
220940_at	12	KIAA1641	NM_025190	---
212854_x_at	15	KIAA1693	AB051480	1p31

53968_at	14	KIAA1698	AI869988	11q12.3
221778_at	15	KIAA1718	BE217882	7q33-q35
218503_at	15	KIAA1797	NM_017794	9p21
215629_s_at	13	KIAA1799	AA905286	---
220368_s_at	12	KIAA2010	NM_017936	14q32.12
212162_at	15	KIDINS220	AK022873	2p24
212163_at	15	KIDINS220	AB033076	2p24
204444_at	13	KIF11	NM_004523	10q24.1
202962_at	14	KIF13B	NM_015254	8p21.1
206364_at	15	KIF14	NM_014875	1pter-q31.3
219306_at	15	KIF15	NM_020242	3p21.32
209234_at	10	KIF1B	BF939474	1p36.2
203087_s_at	15	KIF2	NM_004520	5q12-q13
218755_at	14	KIF20A	NM_005733	5q31
202183_s_at	15	KIF22	NM_007317	16p11.2
204709_s_at	14	KIF23	NM_004856	12q13.12
213623_at	14	KIF3A	NM_007054	5q31
203943_at	14	KIF3B	NM_004798	20q11.21
218355_at	15	KIF4	AF179308	Xq13.1
201991_s_at	15	KIF5B	BF223224	10pter-q22.1
201992_s_at	14	KIF5B	NM_004521	10pter-q22.1
203333_at	15	KIFAP3	NM_014970	1q23.3
205664_at	15	KIN	NM_012311	10p15-p14
205051_s_at	15	KIT	NM_000222	4q11-q12
205978_at	12	KL	NM_004795	13q12
204176_at	14	KLEIP	AA808694	1q24.1-q24.3
202393_s_at	15	KLF10	NM_005655	8q22.2
218486_at	15	KLF11	AA149594	2p25
219371_s_at	15	KLF2	NM_016270	19p13.13-p13.11
221841_s_at	15	KLF4	BF514079	9q31
204334_at	15	KLF7	AA488672	2q32
203543_s_at	12	KLF9	NM_001206	9q13
217906_at	15	KLHDC2	NM_014315	14q21.3-q22.1
208784_s_at	15	KLHDC3	BC001793	6p21.1
214383_x_at	15	KLHDC3	BF063121	6p21.1
221219_s_at	14	KLHDC4	NM_017566	16q24.3
219931_s_at	10	KLHL12	NM_021633	1q32.1
219157_at	15	KLHL2	NM_007246	4q21.2
204177_s_at	14	KLHL20	NM_014458	1q24.1-q24.3
210635_s_at	13	KLHL20	BC005253	1q24.1-q24.3
203068_at	14	KLHL21	NM_014851	1p36.23
206551_x_at	14	KLHL24	NM_017644	3q27.3
221221_s_at	15	KLHL3	NM_017415	5q31
220238_s_at	15	KLHL7	NM_018846	7p15.3
220239_at	15	KLHL7	NM_018846	7p15.3
213117_at	13	KLHL9	AW138594	9p22
213233_s_at	15	KLHL9	AA460694	9p22
216338_s_at	15	KLIP1	AK021433	6p21.1
210667_s_at	15	KNP-I	D86062	21q22.3
212877_at	15	KNS2	AA284075	14q32.3

212878_s_at	15	KNS2	AA284075	14q32.3
213656_s_at	15	KNS2	BF593594	14q32.3
206316_s_at	10	KNTC1	NM_014708	12q24.31
204162_at	14	KNTC2	NM_006101	18p11.31
210715_s_at	15	kop	AF027205	19q13.1
209538_at	15	KOX30	U69645	10q22-q25
202055_at	10	KPNA1	AW051311	3q21
202056_at	13	KPNA1	AW051311	3q21
202058_s_at	14	KPNA1	BC002374	3q21
202059_s_at	13	KPNA1	NM_002264	3q21
213741_s_at	14	KPNA1	BF575685	3q21
201088_at	15	KPNA2	NM_002266	17q23.1-q23.3
211762_s_at	15	KPNA2	BC005978	17q23.1-q23.3
221502_at	15	KPNA3	AL120704	13q14.3
221503_s_at	15	KPNA3	AL120704	13q14.3
212102_s_at	11	KPNA6	BG403834	1p35.1-p34.3
208974_x_at	15	KPNB1	BC003572	17q21.32
208975_s_at	10	KPNB1	BC003572	17q21.32
213507_s_at	15	KPNB1	BG249565	17q21.32
213573_at	15	KPNB1	AA861608	17q21.32
213574_s_at	15	KPNB1	AA861608	17q21.32
213803_at	15	KPNB1	BG545463	17q21.32
204009_s_at	15	KRAS	NM_004985	12p12.1
204010_s_at	13	KRAS	NM_004985	12p12.1
214352_s_at	15	KRAS2	BF673699	12p12.1
34031_i_at	15	Krit1	U90269	7q21-q22
205900_at	11	KRT1	NM_006121	12q12-q13
207023_x_at	15	KRT10	NM_000421	17q21
210633_x_at	15	KRT10	M19156	17q21
201596_x_at	15	KRT18	NM_000224	12q13
215189_at	11	KRTHB6	X99142	12q13
200914_x_at	15	KTN1	NM_004986	14q22.1
200915_x_at	15	KTN1	NM_004986	14q22.1
214709_s_at	15	KTN1	Z22551	14q22.1
217915_s_at	15	L30	AF165521	15q21
213837_at	14	L3MBTL	Z98752	20q13.12
210306_at	12	L3MBTL1	U89358	20q13.12
209387_s_at	15	L6	M90657	3q21-q25
216804_s_at	15	L9; ENH; LIM	AK027217	4q22
211207_s_at	12	LACS5	AF129166	5q31
218701_at	13	LACTB2	NM_016027	8p22-q22.3
205116_at	15	LAMA2	NM_000426	6q22-q23
213519_s_at	15	LAMA2	AI078169	6q22-q23
202202_s_at	15	LAMA4	NM_002290	6q21
210089_s_at	13	LAMA4	BC004241	6q21
210150_s_at	13	LAMA5	BC003355	20q13.2-q13.3
201505_at	15	LAMB1	NM_002291	7q22
211651_s_at	15	LAMB1	M20206	7q22 /// 7q22
216264_s_at	15	LAMB2	X79683	3p21 /// 3p21
200770_s_at	15	LAMC1	NM_002293	1q31

200771_at	15	LAMC1	NM_002293	1q31
216840_s_at	15	LAMM	AK026829	6q22-q23
201551_s_at	13	LAMP1	NM_005561	13q34
201552_at	15	LAMP1	NM_005561	13q34
201553_s_at	15	LAMP1	NM_005561	13q34
200821_at	15	LAMP2	NM_013995	Xq24
203041_s_at	15	LAMP2	NM_002294	Xq24
203042_at	14	LAMP2	NM_002294	Xq24
213801_x_at	15	LAMR1	AW304232	3p21.3
202019_s_at	11	LANCL1	AI935255	2q33-q35
202020_s_at	15	LANCL1	NM_006055	2q33-q35
212408_at	15	LAP1B	AK023204	1q24.2
212409_s_at	14	LAP1B	AK021613	1q24.2
217933_s_at	15	LAP3	NM_015907	4p15.33
200673_at	15	LAPTM4A	NM_014713	2p24.3
208029_s_at	15	LAPTM4B	NM_018407	8q22.1
208767_s_at	15	LAPTM4B	AW149681	8q22.1
214039_s_at	15	LAPTM4B	T15777	8q22.1
201720_s_at	10	LAPTM5	AI589086	1p34
201721_s_at	15	LAPTM5	NM_006762	1p34
212137_at	15	LARP	AV746402	---
208954_s_at	15	LARP5	BC003381	10p15.3
218651_s_at	15	LARP6	NM_018357	15q22.32
204016_at	13	LARS2	NM_015340	3p21.3
34764_at	15	LARS2	D21851	3p21.3
200618_at	15	LASP1	NM_006148	17q11-q21.3
222212_s_at	15	LASS2	AK001105	1q21.3
212442_s_at	15	LASS6	BG289001	2q31.1
212446_s_at	15	LASS6	AI658534	2q31.1
208118_x_at	11	LAT1-3TM	NM_031211	16p12
211768_at	12	LAT2	BC006080	7q11.23
213261_at	14	LBA1	AA035414	3p22.1
221011_s_at	15	LBH	NM_030915	2p23.3
201795_at	15	LBR	NM_002296	1q42.1
212588_at	12	LCA	Y00062	1q31-q32
216203_at	14	LCB2	U15555	14q24.3-q31
209827_s_at	13	LCF	M90391	15q26.3
221515_s_at	15	LCMT1	BC001214	16p12.3-16p12.1
204012_s_at	10	LCMT2	AL529189	15q15.1
206481_s_at	15	LDB2	NM_001290	4p16
213371_at	15	LDB3	AI803302	10q22.3-q23.2
213717_at	15	LDB3	AA211481	10q22.3-q23.2
200650_s_at	15	LDHA	NM_005566	11p15.4
201030_x_at	15	LDHB	NM_002300	12p12.2-p12.1
202068_s_at	15	LDLR	NM_000527	19p13.3
209866_s_at	13	LEC3	AF307080	4q13.1
221558_s_at	12	LEF1	AF288571	4q23-q25
206012_at	14	LEFTY2	NM_003240	1q42.1
218604_at	15	LEMD3	NM_014319	12q14
202377_at	15	LEPR	AW026535	1p31

209894_at	15	LEPR	U50748	1p31
220750_s_at	14	LEPRE1	NM_022356	1p34.1
218717_s_at	12	LEPREL1	NM_018192	3q29
202378_s_at	15	LEPROT	NM_017526	1p31
202594_at	15	LEPROTL1	NM_015344	8p21.2-p21.1
202595_s_at	15	LEPROTL1	BC000642	8p21.2-p21.1
201593_s_at	15	LEREPO4	AV716798	2q32.2
201595_s_at	15	LEREPO4	NM_018471	2q32.2
207170_s_at	15	LETMD1	NM_015416	12q13.13
217810_x_at	15	LEUS	D84223	5q32
216942_s_at	15	LFA3; LFA-3	D28586	1p13
201105_at	15	LGALS1	NM_002305	22q13.1
220158_at	10	LGALS14	NM_020129	19q13.2
208949_s_at	15	LGALS3	BC001120	14q21-q22
200923_at	14	LGALS3BP	NM_005567	17q25
208934_s_at	15	LGALS8	AI659005	1q42-q43
208936_x_at	15	LGALS8	AI659005	1q42-q43
210731_s_at	15	LGALS8	AL136105	1q42-q43
210732_s_at	14	LGALS8	AL136105	1q42-q43
218326_s_at	15	LGR4	NM_018490	11p14-p13
218253_s_at	15	LGTN	NM_006893	1q31-q32
218656_s_at	15	LHFP	NM_005780	13q12
212658_at	15	LHFPL2	N66633	5q14.1
219884_at	13	LHX6	NM_014368	9q34.11
214045_at	11	LIAS	BF056778	4p14
205876_at	13	LIFR	NM_002310	5p13-p12
202347_s_at	15	LIG	AB022435	4p14
206235_at	10	LIG4	NM_002312	13q33-q34
208987_s_at	10	LILINA	AF179221	11q13.1
218850_s_at	13	LIMD1	NM_014240	3p21.3
202193_at	14	LIMK2	NM_005569	22q12.2
210582_s_at	10	LIMK2	AL117466	22q12.2
207198_s_at	15	LIMS1	NM_004987	2q12.3
212687_at	15	LIMS1	AL110164	2q12.3
220765_s_at	15	LIMS2	NM_017980	2q21.1
206440_at	11	LIN7A	NM_004664	12q21
219760_at	15	LIN7B	NM_022165	19q13.33
219399_at	13	LIN7C	NM_018362	11p14 /// 11p14
221568_s_at	13	LIN7C	N63709	11p14
201847_at	15	LIPA	NM_000235	10q23.2-q23.3
205571_at	15	LIPT1	NM_015929	2q11.2
200815_s_at	15	LIS1	L13386	17p13.3
200706_s_at	14	LITAF	NM_004862	16p13.3-p12
202386_s_at	15	LKAP	NM_019081	16p13.13
210852_s_at	13	LKRSDH	AF229180	7q31.3
214829_at	12	LKRSDH	AK023446	7q31.3
203713_s_at	15	LLGL2	NM_004524	17q24-q25
216387_x_at	12	LLT1	AL353580	---
200805_at	15	LMAN2	NM_006816	5q35.3
221274_s_at	15	LMAN2L	NM_030805	2q11.2 /// 2q11.2

220036_s_at	15	LMBR1L	NM_018113	12q13.12
218191_s_at	15	LMBRD1	NM_018368	6q13
218574_s_at	15	LMCD1	NM_014583	3p26-p24
203411_s_at	15	LMNA	NM_005572	1q21.2-q21.3
214213_x_at	14	LMNA	AA063189	1q21.2-q21.3
203276_at	13	LMNB1	NM_005573	5q23.3-q31.1
216952_s_at	12	LMNB2	M94363	---
204249_s_at	15	LMO2	NM_005574	11p13
209204_at	10	LMO4	AI824831	1p22.3
209205_s_at	15	LMO4	BC003600	1p22.3
202674_s_at	12	LMO7	NM_005358	13q21.33
203320_at	15	LNK	NM_005475	12q24
216187_x_at	15	LNX1	AF222691	---
48117_at	11	LOC112869	AA160496	16p12.1
212714_at	15	LOC113251	AL050205	12q13.12
216547_at	15	LOC127406	AL353681	---
212017_at	15	LOC130074	BF677404	2q21.2
212934_at	14	LOC137886	AI245523	8q12.1
212080_at	13	LOC143941	AV714029	11q23
216559_x_at	15	LOC144983	AL050348	---
213527_s_at	15	LOC146542	AC002310	16p11.2
222039_at	14	LOC146909	AA292789	17q21.31
212155_at	15	LOC149603	AA085748	1q42.13
213703_at	15	LOC150759	W95043	2q11.2
221973_at	14	LOC150759	AI983904	---
212098_at	15	LOC151162	AL134724	---
215978_x_at	14	LOC152719	AK021514	---
212731_at	15	LOC157567	U79297	8q22.3
213212_x_at	14	LOC161527	AI632181	---
212697_at	15	LOC162427	AL515874	17q21.31
211325_x_at	14	LOC171220	U72518	12p13.31
212866_at	10	LOC203069	AI081543	8p21.2
35156_at	15	LOC203069	AL050297	8p21.2
213510_x_at	15	LOC220594	AW194543	17p11.2
214920_at	14	LOC221981	R33964	7p21.3
213078_x_at	11	LOC254531	AI889513	15q13.2
65630_at	13	LOC283232	AI742455	11p15.5
214719_at	11	LOC283537	AK026720	13q12.3
217520_x_at	12	LOC283683	BG396614	15q12
210242_x_at	15	LOC283687	AF249277	15q24.3
213391_at	15	LOC286148	AI669947	8q22.1
59433_at	14	LOC286434	N32185	---
209733_at	10	LOC286440	AL034399	---
214035_x_at	15	LOC339047	AA308853	---
214870_x_at	15	LOC339047	AC002045	---
221501_x_at	15	LOC339047; KIAA0220	AF229069	16p13.12
50374_at	15	LOC339229	AA150503	---
212708_at	15	LOC339287	AV721987	17q21.2
222000_at	15	LOC339448	AI915947	1p36.32
213360_s_at	15	LOC340318	AA514622	7q11.23

217365_at	11	LOC343070	AL022101	---
213408_s_at	15	LOC375133	AK024034	22q11.21
216505_x_at	15	LOC376693	AL118502	---
214946_x_at	15	LOC387680	AV728658	10q11.23
213689_x_at	15	LOC388650	AL137958	1p22.1
65588_at	15	LOC388796	AA827892	20q12
222370_x_at	13	LOC388906	N57781	---
65472_at	10	LOC388969	AI161338	2p11.2
222368_at	14	LOC389634	AW972351	---
51228_at	14	LOC389677	N36928	8q22.1
213556_at	13	LOC390940	BE673445	19q13.32
51158_at	10	LOC400451	AI801973	---
215283_at	15	LOC400642	U79248	18p11.31
217506_at	15	LOC400642	H49382	18p11.31
210396_s_at	15	LOC440354	AF271775	16p12.1
222207_x_at	15	LOC441258	AK024602	---
201871_s_at	15	LOC51035	NM_015853	11q12.3
218303_x_at	15	LOC51315	NM_016618	2p11.2
210718_s_at	15	LOC51326	AF119889	17q21.32
220014_at	14	LOC51334	NM_016644	5q23.2
51774_s_at	15	LOC51619	AW014299	7p13
58900_at	15	LOC51619	AW025284	7p13
65521_at	14	LOC51619	W74577	---
213142_x_at	15	LOC54103	AV700415	7q21.11
222150_s_at	15	LOC54103	AK026747	7q21.11
201921_at	15	LOC552891	NM_004125	9q32
218484_at	15	LOC56901	NM_020142	12q13.2
203622_s_at	11	LOC56902	NM_020143	2p13.3
213272_s_at	15	LOC57146	AF070596	16p12
203897_at	15	LOC57149	BE963444	16p11.2
209679_s_at	14	LOC57228	BC003379	12q13.13
218263_s_at	15	LOC58486	NM_021211	11p15.3
220770_s_at	15	LOC63920	NM_022090	5q34
220020_at	11	LOC63929	NM_022098	22q13.31-q13.33
44669_at	15	LOC644096	N31716	---
217653_x_at	12	LOC653471	AW150065	---
221249_s_at	15	LOC81558	NM_030802	17q21.33
208107_s_at	14	LOC81691	NM_030941	16p13.11
215215_s_at	14	LOC81691	AC004381	16p13.11
214748_at	15	LOC88523	U50529	13q12.3
221823_at	13	LOC90355	AL565741	5q21.2
91952_at	14	LOC90379	AI363375	19p13.13
221825_at	15	LOC90806	BE671941	1q32.3
221826_at	11	LOC90806	BE671941	1q32.3
213502_x_at	10	LOC91316	AA398569	22q11.23
212957_s_at	14	LOC92249	AU154785	Xq11.2
213220_at	15	LOC92482	AV706096	---
213224_s_at	15	LOC92482	AK025724	---
213455_at	15	LOC92689	W87466	4p14
213346_at	15	LOC93081	BE748563	13q33.1

221740_x_at	15	LOC9884	AI140364	17q24.2
205011_at	13	LOH11CR2A	NM_014622	11q23
210102_at	12	LOH11CR2A	BC001234	11q23
220244_at	14	LOH3CR2A	NM_013343	3p24-26
204298_s_at	15	LOX	NM_002317	5q23.2
215446_s_at	15	LOX	L16895	---
203570_at	13	LOXL1	NM_005576	15q24-q25
202998_s_at	15	LOXL2	AF117949	8p21.3-p21.2
212047_s_at	12	LP2254	AK025329	17p13.3
208816_x_at	11	LPC2B	M62898	---
202651_at	15	LPGAT1	NM_014873	1p36.13-q42.3
47560_at	10	LPHN1	AI525402	19p13.2
206953_s_at	15	LPHN2	NM_012302	1p31.1
212276_at	15	LPIN1	D80010	2p25.1
202459_s_at	15	LPIN2	NM_014646	18p11.31
202460_s_at	12	LPIN2	NM_014646	18p11.31
203548_s_at	15	LPL	BF672975	8p22
203549_s_at	15	LPL	NM_000237	8p22
203007_x_at	15	LPL1	AF077198	8q11.23
215280_s_at	14	LPNA3	AK023850	19q13.33
202822_at	15	LPP	BF221852	3q27-q28
209147_s_at	15	LPP1	AB000888	5q11
209355_s_at	15	LPP3	AB000889	1pter-p22.1
213496_at	13	LPPR4	AW592563	1p21.3
216250_s_at	13	LPXN	X77598	11q12.1
220532_s_at	15	LR8	NM_014020	7q36.1
212692_s_at	15	LRBA	W60686	4q31.23
214109_at	15	LRBA	AI659561	4q31.23
36907_at	10	LRBP; MVLK	M88468	12q24
37796_at	12	LRCH4	AF053356	7q22
219491_at	12	LRFN4	NM_024036	11q13.1
211596_s_at	14	LRIG1	AB050468	--- /// ---
205953_at	15	LRIG2	NM_014813	1p13.1
201412_at	15	LRP10	NM_014045	14q11.2
219631_at	15	LRP12	NM_024937	8q22.2-q23.1
219188_s_at	15	LRP16	BC000270	11q11
34697_at	12	LRP6	AF074264	12p11-p13
201186_at	15	LRPAP1	NM_002337	4p16.3
211615_s_at	15	LRPPRC	M92439	2p22.1 /// 2p22.1
211971_s_at	15	LRPPRC	AI653608	2p22.1
205381_at	15	LRRC17	NM_005824	7q22.1
219949_at	15	LRRC2	NM_024512	3p21.31
218550_s_at	12	LRRC20	NM_018205	10q22.2
203835_at	14	LRRC32	NM_005512	11q13.5-q14
218577_at	15	LRRC40	NM_017768	1p31.2
201932_at	13	LRRC41	NM_006369	1p34.1
219338_s_at	15	LRRC49	NM_017691	15q22.32
218245_at	12	LRRC54	NM_015516	11q13.3
206483_at	10	LRRC6	NM_012472	8q24.22
218684_at	14	LRRC8D	NM_018103	1p22.2

201861_s_at	15	LRRFIP1	BF965566	---
201862_s_at	15	LRRFIP1	NM_004735	2q37.3
211452_x_at	15	LRRFIP1	AF130054	2q37.3
218364_at	15	LRRFIP2	NM_017724	3p22.1
220610_s_at	15	LRRFIP2	NM_006309	3p22.1
209841_s_at	15	LRRN3	AI221950	7q31.1
203534_at	15	LSM1	NM_014462	8p11.2
202209_at	15	LSM3	NM_014463	3p25.1
202736_s_at	15	LSM4	AA112507	19p13.11
202737_s_at	15	LSM4	AA112507	19p13.11
202904_s_at	13	LSM5	AU153477	7p14.3
211747_s_at	15	LSM5	BC005938	7p14.3 /// 7p14.3
205036_at	15	LSM6	NM_007080	4q31.21
204559_s_at	15	LSM7	NM_016199	19p13.3
219119_at	15	LSM8	NM_016200	7q31.1-q31.3
214110_s_at	15	LSP1	BF195104	---
220171_x_at	11	LSR7	NM_018559	13q13-q14
202245_at	15	LSS	AW084510	21q22.3
208771_s_at	15	LTA4H	J02959	12q22
202728_s_at	14	LTBP1	AI986120	2p22-p21
202729_s_at	15	LTBP1	AI986120	2p22-p21
204682_at	14	LTBP2	NM_000428	14q24
219922_s_at	15	LTBP3	NM_021070	11q12
204442_x_at	15	LTBP4	NM_003573	19q13.1-q13.2
40093_at	15	LU	X83425	19q13.2
220143_x_at	14	LUC7L	NM_018032	16p13.3
217842_at	14	LUC7L2	NM_016019	7q34
220099_s_at	15	LUC7L2	NM_016007	7q34
201394_s_at	15	LUCA15	U23946	3p21.3
201395_at	15	LUCA15	U23946	3p21.3
201744_s_at	15	LUM	NM_002345	12q21.3-q22
221832_s_at	15	LUZP1	AV741657	1p36.11
219588_s_at	13	LUZP5	NM_017760	7q36.3
218729_at	15	LXN	NM_020169	3q25.32
219860_at	15	LY6G5C	NM_025262	6p21.31
206773_at	15	LY6H	NM_002347	8q24.3
206584_at	15	LY96	NM_015364	8q13.3
52169_at	14	LYK5	AI302185	17q24.2
202625_at	15	LYN	AI356412	8q13
202626_s_at	15	LYN	AI356412	8q13
212449_s_at	15	LYPLA1	BG288007	8q11.23
202292_x_at	15	LYPLA2	NM_007260	1p36.12-p35.1
216606_x_at	15	LYPLA2	AL050332	---
204458_at	15	LYPLA3	AL110209	16q22.1
212248_at	13	LYRIC	AI886796	8q22.1
212250_at	15	LYRIC	AV700332	8q22.1
212251_at	15	LYRIC	AI972475	8q22.1
202169_s_at	15	LYS2	AF302110	11q22
202170_s_at	15	LYS2	AF151057	11q22
204076_at	15	LYSAL1	AB002390	8p21.2

203518_at	13	LYST	NM_000081	1q42.1-q42.2
210943_s_at	13	LYST	U84744	1q42.1-q42.2
213975_s_at	12	LYZ	AV711904	12q14.3
218437_s_at	15	LZTFL1	AA843132	3p21.3
203412_at	15	LZTR1	NM_006767	22q11.21
200722_s_at	15	M11S1	BG258784	11p13
215049_x_at	15	M130; MM130	Z22969	12p13.3
201383_s_at	11	M17S2	AL044170	---
209026_x_at	15	M40	AF141349	6p21.32
212320_at	15	M40	BC001002	6p21.32
208677_s_at	15	M6	L20471	19p13.3
200900_s_at	15	M6PR	AI583537	12p13
200901_s_at	15	M6PR	AI583537	12p13
202122_s_at	14	M6PRBP1	NM_005817	19p13.3
221494_x_at	15	M9	AF085358	19q13.2
203345_s_at	15	M96	AI566096	1p22.1
203346_s_at	14	M96	AF072814	1p22.1
209704_at	13	M96	AL523380	1p22.1
209705_at	15	M96	BG033764	1p22.1
212279_at	15	MAC30	BE779865	17q11.2
212281_s_at	15	MAC30	BF038366	17q11.2
212282_at	15	MAC30	BE779865	17q11.2
207358_x_at	15	MACF1	NM_012090	1p32-p31
208633_s_at	15	MACF1	W61052	1p32-p31
201670_s_at	15	MACS	M68956	6q22.2
204857_at	14	MAD1L1	NM_003550	7p22
203362_s_at	15	MAD2L1	NM_002358	4q27
203094_at	15	MAD2L1BP	NM_014628	6p21.1
212346_s_at	11	MAD4	AK026392	4p16.3
210752_s_at	12	MAD7	AF213666	17q21.1
217909_s_at	15	MAD7	AF213668	17q21.1
217910_x_at	15	MAD7	AF213668	---
208037_s_at	14	MADCAM1	NM_007164	19p13.3
38398_at	15	MADD	AB002356	11p11.2
203076_s_at	15	MADR2	U65019	18q21.1
207922_s_at	15	MAEA	NM_005882	4p16.3
209348_s_at	15	MAF	BF508646	16q22-q23
36711_at	15	MAFF	AL021977	22q13.1
213627_at	15	MAGED2	AI924630	Xp11.2
210882_s_at	10	MAGED3	U04811	Xp11.22-p11.21
221261_x_at	15	MAGED4	NM_030801	
218176_at	15	MAGEF1	NM_022149	3q13
218573_at	15	MAGEH1	NM_014061	Xp11.22
206144_at	14	MAGI1	NM_004742	3p14.1
218969_at	15	Magmas	NM_016069	16p13.3
210092_at	15	MAGOH	AF067173	1p34-p33
210093_s_at	15	MAGOH	AF067173	1p34-p33
219362_at	13	MAK10	NM_024635	---
220925_at	14	MAK10	NM_021929	9q22.1
217745_s_at	15	MAK3	NM_025146	3q13.31

204777_s_at	14	MAL	NM_002371	2cen-q13
208309_s_at	14	MALT1	NM_006785	18q21
210017_at	14	MALT1	AF070528	18q21
210018_x_at	15	MALT1	AB026118	18q21
202360_at	15	MAML1	NM_014757	5q35
208116_s_at	10	MAN1A1	NM_005907	6q22
221760_at	15	MAN1A1	BG287153	6q22
217921_at	13	MAN1A2	BE543064	1p13
217922_at	15	MAN1A2	BE543064	1p13
65884_at	15	MAN1B1	AA631254	9q34
214180_at	14	MAN1C1	AW340588	1p35
218918_at	15	MAN1C1	NM_020379	1p35
205105_at	13	MAN2A1	NM_002372	5q21-q22
202032_s_at	15	MAN2A2	NM_006122	15q26.1
219999_at	15	MAN2A2	NM_018621	15q26.1
214703_s_at	15	MAN2B2	AW954107	4p16.2
203668_at	15	MAN2C1	NM_006715	15q11-q13
209166_s_at	15	MANB; LAMAN	U68567	19cen-q13.1
203778_at	15	MANBA	NM_005908	4q22-q25
220945_x_at	14	MANSC1	NM_018050	12p13.2
212741_at	14	MAOA	AA923354	Xp11.4-p11.3
204041_at	15	MAOB	NM_000898	Xp11.23
212508_at	15	MAP-1; PNMA4	AK024029	14q32
208786_s_at	15	MAP1A/1BLC3	AF183417	16q24.2
212233_at	15	MAP1B	AL523076	5q13
208785_s_at	15	MAP1LC3B	BE893893	---
202670_at	15	MAP2K1	AI571419	15q22.1-q22.33
217971_at	15	MAP2K1IP1	NM_021970	4q23
202424_at	15	MAP2K2	NM_030662	7q32
207667_s_at	12	MAP2K3	NM_002756	17q11.2
215498_s_at	15	MAP2K3	AA780381	17q11.2
215499_at	14	MAP2K3	AA780381	17q11.2
203265_s_at	11	MAP2K4	NM_003010	17p11.2
203266_s_at	15	MAP2K4	NM_003010	17p11.2
203652_at	15	MAP3K11	NM_002419	11q13.1-q13.3
205448_s_at	13	MAP3K12	NM_006301	12q13
203514_at	10	MAP3K3	BF971923	17q24.2
204089_x_at	14	MAP3K4	NM_006724	6q25.3
216199_s_at	13	MAP3K4	AL109942	6q25.3
203837_at	14	MAP3K5	NM_005923	6q22.33
206853_s_at	14	MAP3K7	AL121964	6q16.1-q16.3
206854_s_at	15	MAP3K7	NM_003188	6q16.1-q16.3
210284_s_at	14	MAP3K7IP2	AB018276	6q25.1-q25.3
212184_s_at	15	MAP3K7IP2	AL117407	6q25.1-q25.3
212566_at	15	MAP4	AL523310	3p21
212567_s_at	15	MAP4	AL523310	3p21
243_g_at	15	MAP4	M64571	3p21
33850_at	15	MAP4	W28892	3p21
218311_at	15	MAP4K3	NM_003618	2p22.3
206571_s_at	15	MAP4K4	NM_004834	2q11.2-q12

218181_s_at	15	MAP4K4	AL561281	2q12.1
203552_at	15	MAP4K5	AW298170	14q11.2-q21
203553_s_at	15	MAP4K5	NM_006575	14q11.2-q21
218291_at	15	MAPBPIP	NM_014017	1q22
208351_s_at	14	MAPK1	NM_002745	22q11.2
212271_at	15	MAPK1	AA195999	22q11.2
206106_at	12	MAPK12	AL022328	22q13.33
210058_at	13	MAPK13	BC000433	6p21.31
202530_at	15	MAPK14	NM_001315	6p21.3-p21.2
211561_x_at	14	MAPK14	L35253	---
207121_s_at	15	MAPK6	NM_002748	15q21
213178_s_at	15	MAPK8IP3	AB028989	16p13.3
203218_at	15	MAPK9	W37431	5q35
217808_s_at	14	MAPKAP1	NM_024117	9q34.12
201460_at	15	MAPKAPK2	AI141802	1q32
201461_s_at	14	MAPKAPK2	NM_004759	1q32
215050_x_at	11	MAPKAPK2	BG325734	1q32
202787_s_at	15	MAPKAPK3	NM_004635	3p21.3
202788_at	15	MAPKAPK3	NM_004635	3p21.3
212871_at	14	MAPKAPK5	NM_003668	12q24.13
200712_s_at	15	MAPRE1	NM_012325	20q11.1-11.23
200713_s_at	15	MAPRE1	NM_012325	20q11.1-11.23
202501_at	15	MAPRE2	NM_014268	18q12.2
213489_at	13	MAPRE2	BE671156	18q12.2
218582_at	15	MARCH5	NM_017824	10q23.33
201737_s_at	15	MARCH6	NM_005885	5p15.2
202653_s_at	15	MARCH7	BC003404	2q24.2
202654_x_at	15	MARCH7	NM_022826	2q24.2
213256_at	15	MARCH-III	AW593996	5q23.3
201736_s_at	15	MARCH-VI	BF000409	5p15.2
212498_at	15	MARCH-VI	AF056433	---
201668_x_at	14	MARCKS	AW163148	6q22.2
201669_s_at	15	MARCKS	NM_002356	6q22.2
213002_at	15	MARCKS	AA770596	6q22.2
200644_at	15	MARCKSL1	NM_023009	1p34.3
221047_s_at	15	MARK1	NM_018650	1q42.11
202568_s_at	15	MARK3	AI745639	14q32.3
202569_s_at	15	MARK3	NM_002376	14q32.3
55065_at	15	MARK4	AL120554	19q13.3
201475_x_at	15	MARS	NM_004990	12q13.2
213671_s_at	15	MARS	AA621558	12q13.2
217956_s_at	15	MASA	NM_021204	4q21.3
218499_at	13	MASK	NM_016542	Xq26.2
213749_at	15	MASP1	AV686235	3q27-q28
215903_s_at	15	MAST2	BE786598	1p34.1
200768_s_at	15	MAT2A	BC001686	2p11.2
213705_at	10	MAT2A	AW301861	2p11.2
217993_s_at	15	MAT2B	NM_013283	5q34-q35.1
202350_s_at	15	MATN2	NM_002380	8q22
200624_s_at	15	MATR3	AA577695	5q31.3

200626_s_at	15	MATR3	NM_018834	5q31.3
214363_s_at	15	MATR3	AA129420	5q31.3
219543_at	12	MAWBP	NM_022129	10pter-q25.3
209331_s_at	15	MAX	AA723514	14q23
209332_s_at	15	MAX	AA723514	14q23
212064_x_at	14	MAZ	AI471665	16p11.2
204179_at	15	MB	NM_005368	22q13.1
208858_s_at	11	MBC2	BC004998	12q13.13
203353_s_at	14	MBD1	NM_015846	18q21
202484_s_at	15	MBD2	AF072242	18q21
41160_at	15	MBD3	AC005943	19p13.3
209579_s_at	15	MBD4	AL556619	3q21-q22
209580_s_at	15	MBD4	AL556619	3q21-q22
214047_s_at	15	MBD4	AI913365	3q21-q22
214048_at	10	MBD4	AI913365	3q21-q22
220195_at	12	MBD5	NM_018328	2q23.3
209058_at	15	MBF1	AB002282	9q34.3
209059_s_at	15	MBF1	AB002282	9q34.3
218411_s_at	13	MBIP	NM_016586	14q13.2
201151_s_at	13	MBNL1	BF512200	3q25
201152_s_at	15	MBNL1	N31913	3q25
201153_s_at	15	MBNL1	N31913	3q25
203640_at	15	MBNL2	BE328496	13q32.2
210136_at	13	MBP	AW070431	18q23
209356_x_at	15	MBP1	AB030655	11q13
201620_at	15	MBTPS1	NM_003791	
217543_s_at	14	MBTPS1	BE890314	
209408_at	15	MCAK; KNSL6	U63743	1p34.1
211519_s_at	13	MCAK; KNSL6	AY026505	1p34.1
209086_x_at	15	MCAM	BE964361	11q23.3
211042_x_at	14	MCAM	BC006329	---
204656_at	11	MCART1	AL138752	9p12-p11
209366_x_at	15	MCB5	M22865	18q23
206132_at	14	MCC	NM_002387	5q21-q22
218440_at	15	MCCC1	NM_020166	3q27
209623_at	15	MCCC2	AW439494	5q12-q13
35147_at	15	MCF2L	AB002360	13q34
212245_at	15	MCFD2	AL567779	2p21
212246_at	13	MCFD2	BE880828	2p21
205716_at	13	MCFP	NM_018843	7q21.13
210775_x_at	13	MCH6	AB015653	1p36.3-p36.1
200797_s_at	15	MCL1	AI275690	1q21
200798_x_at	15	MCL1	NM_021960	1q21
214057_at	11	MCL1	H71805	1q21
217274_x_at	15	MCL1	X52005	---
202107_s_at	15	MCM2	NM_004526	3q21
201555_at	15	MCM3	NM_002388	6p12
212269_s_at	15	MCM3AP	AJ010089	21q22.3
220459_at	14	MCM3APAS	NM_018118	21q22.3
222036_s_at	15	MCM4	AI859865	8q11.2

222037_at	12	MCM4	AI859865	8q11.2
216237_s_at	15	MCM5	AA807529	22q13.1
201930_at	15	MCM6	NM_005915	2q21
219952_s_at	11	MCOLN1	NM_020533	19p13.3-p13.2
207549_x_at	11	MCP	NM_002389	1q32
208783_s_at	15	MCP	AL570661	1q32
216598_s_at	10	MCP-1	S69738	17q11.2-q21.1
202556_s_at	15	MCRS1	NM_006337	12q13.12
210807_s_at	13	MCT2	AF049608	12q13
218163_at	15	MCTS1	NM_014060	Xq22-q24
208825_x_at	15	MDA20; FLJ27455	U43701	17q11
203062_s_at	15	MDC1	NM_014641	6pter-p21.31
215543_s_at	15	MDC1D; KIAA0609	BF057493	22q12.3
200978_at	15	MDH1	NM_005917	2p13.3
209036_s_at	15	MDH2	BC001917	7p12.3-q11.2
213333_at	15	MDH2	AL520774	7p12.3-q11.2
213761_at	15	MDM1	AW664850	12q14.3
212693_at	15	MDN1	BE670928	---
209994_s_at	15	mdr1	AF016535	7q21.1
210317_s_at	15	MDS	U28936	17p13.3
210996_s_at	15	MDS	U43430	17p13.3
218288_s_at	15	MDS025	NM_021825	11q14.1
218597_s_at	15	MDS029	NM_018464	10q21.3
219348_at	15	MDS032	NM_018467	19p13.12
221706_s_at	15	MDS032	BC006005	19p13.12
214042_s_at	15	MDS1	AW071997	---
221775_x_at	15	MDS1	BG152979	---
204058_at	14	ME1	AL049699	6q12
204059_s_at	15	ME1	NM_002395	6q12
209397_at	15	ME2	BC000147	6p25-p24
204663_at	15	ME3	NM_006680	11cen-q22.3
218061_at	15	MEA1	NM_014623	6p21.3-p21.1
203506_s_at	12	MED12	NM_005120	Xq13
212208_at	15	MED13L	AK023837	12q24.22
218438_s_at	15	MED28	NM_025205	4p16
217843_s_at	15	MED4	NM_014166	13q14.12
207079_s_at	12	MED6	NM_005466	14q24.1
210104_at	13	MED6	AF074723	14q24.1
213127_s_at	15	MED8	BG230758	1p34.1
218372_at	15	MED9	NM_018019	17p11.2
31845_at	14	MEF	U32645	Xq26
208328_s_at	15	MEF2A	NM_005587	15q26
212535_at	15	MEF2A	AA142929	15q26
209199_s_at	15	MEF2C	L08895	5q14
209200_at	15	MEF2C	AL536517	5q14
203003_at	15	MEF2D	AL530331	1q12-q23
211678_s_at	15	MEG3	AF090934	14q32 /// 14q32
203812_at	13	MEGF5	AB011538	5q35
203813_s_at	13	MEGF5	AB011538	5q35
214778_at	11	MEGF8	AB011541	19q12

204069_at	15	MEIS1	NM_002398	2p14-p13
207480_s_at	15	MEIS2	NM_020149	15q13.3
214077_x_at	15	MEIS4	H15129	17p11.2
221695_s_at	12	MEKK2; MEKK2B	AF239798	2q21.1
204825_at	15	MELK	NM_014791	9p13.1
202645_s_at	15	MEN1	NM_000244	11q13
205619_s_at	15	MEOX1	NM_004527	17q21
206201_s_at	15	MEOX2	NM_005924	7p22.1-p21.3
206028_s_at	14	MERTK	NM_006343	2q14.1
202016_at	15	MEST	NM_002402	7q32
212673_at	15	METAP1	D42084	4q23
219051_x_at	14	METRN	NM_024042	16p13.3
209265_s_at	15	METTL3	BC001650	14q11.1
219698_s_at	14	METTL4	NM_022840	18p11.31
203406_at	15	MFAP1	NM_005926	15q15-q21
203417_at	15	MFAP2	NM_017459	1p36.1-p35
213123_at	15	MFAP3	BE222709	5q32-q33.2
212713_at	14	MFAP4	R72286	17p11.2
213764_s_at	15	MFAP5	AW665892	12p13.1-p12.3
213765_at	15	MFAP5	AW665892	12p13.1-p12.3
210605_s_at	15	MFGE8	BC003610	15q25
213457_at	13	MFHAS1	BF739959	8p23.1
207098_s_at	15	MFN1	NM_017927	3q27.1
217043_s_at	15	MFN1	U95822	3q27.1
201155_s_at	15	MFN2	NM_014874	1p36.21
204153_s_at	13	MFNG	AI738965	22q12
218109_s_at	15	MFSD1	NM_022736	3q25.32
222138_s_at	15	MG21; FLJ20563	AF158978	Xp11.23 /// Xp11.23
212945_s_at	14	MGA	BE502432	15q15
201126_s_at	15	MGAT1	NM_002406	5q35
203102_s_at	15	MGAT2	NM_002408	14q21
211061_s_at	14	MGAT2	BC006390	14q21 /// 14q21
220189_s_at	15	MGAT4B	NM_014275	5q35
215528_at	14	MGAT5	AL049390	---
205740_s_at	15	MGC10433	NM_024321	19q13.13
208094_s_at	14	MGC10471	NM_030818	19p13.13
217047_s_at	15	MGC105131	AK027138	4q22.1
212068_s_at	12	MGC10526	AB011087	9q34.3
218897_at	13	MGC10993	NM_030577	2q14.2
209029_at	15	MGC110877	AF193844	12p13.31
212861_at	14	MGC11308	BF690150	12q13.13
221912_s_at	15	MGC1203	AL049795	1p36.11-p34.2
202783_at	15	MGC126502; MGC126503	U40490	5p13.1-5cen
202784_s_at	15	MGC126502; MGC126503	U40490	5p13.1-5cen
211038_s_at	15	MGC12760	BC006312	1p36.13
221864_at	15	MGC13024	AW517464	16p11.2
57516_at	12	MGC13138	AA746290	16p11.2
214696_at	12	MGC14376	AF070569	17p13.3
212890_at	11	MGC15523	BF663461	17q25.3
203173_s_at	15	MGC16824	NM_020314	16p13.11

211159_s_at	14	MGC2134; MGC8949	AB000635	6p21.1
212340_at	15	MGC21416	BE673723	Xq12
212341_at	15	MGC21416	AA195936	Xq12
212342_at	13	MGC21416	BG500611	Xq12
209391_at	12	MGC21559; MGC111193	AF061729	9q34.13
214061_at	14	MGC21654	AI017564	8q24.13
221904_at	15	MGC21688	AI141670	3q27.3
209328_x_at	11	MGC2198	BC000587	5q35.3
209329_x_at	15	MGC2198	BC000587	5q35.3
213065_at	15	MGC23401	AB011118	12q15
208653_s_at	14	MGC-24	AF299343	6q21
208654_s_at	15	MGC-24	AF299343	6q21
213104_at	15	MGC24381	AI799802	16p13.3
221637_s_at	14	MGC2477	BC001434	11q12.3
203119_at	13	MGC2574	NM_024098	11q12.2
213610_s_at	15	MGC2610	BE326381	2q31.1
209566_at	15	MGC26273	AL080184	2q14.2
218945_at	15	MGC2654	NM_024109	16p13.2
218903_s_at	15	MGC2731	NM_024068	12q13.13
222064_s_at	15	MGC2744	AI093187	17q21.31
219097_x_at	15	MGC2747	NM_024104	19p13.12
221988_at	15	MGC2747	AA463853	---
200076_s_at	15	MGC2749	BC006479	19p13.11
218641_at	15	MGC3032	NM_023941	11q13
215380_s_at	15	MGC3077; FLJ11717	AK021779	7p15-p14
218714_at	12	MGC3121	NM_024031	16p11.2
45687_at	15	MGC3121	AA161130	16p11.2
218419_s_at	15	MGC3123	NM_024107	17q21.31
220934_s_at	15	MGC3196	NM_024084	---
220305_at	12	MGC3260	NM_024030	20p13
218953_s_at	15	MGC3265	NM_024028	5q33.1
213644_at	15	MGC33887	AI979276	17q24.2
213392_at	15	MGC35048	AW070229	16p13.11
219324_at	13	MGC3731	NM_024313	22q13.1
212178_s_at	15	MGC3792	AK022555	7q11.23
214773_x_at	15	MGC3794	AI983505	1q23.2
221959_at	15	MGC39325	BE672313	8q12.1
216126_at	13	MGC39821	AK022418	19p13.11
214051_at	15	MGC39900	BF677486	Xq22.2
218756_s_at	12	MGC4172	NM_024308	17q21.1
220352_x_at	13	MGC4278	NM_024305	---
218130_at	12	MGC4368	NM_024510	17q25.3
210933_s_at	10	MGC4655	BC004908	16q22.1
203257_s_at	14	MGC4707	NM_024113	11p11.2
221620_s_at	15	MGC4825	AF061264	Xp22.13
221548_s_at	15	MGC4846	AY024365	2q37.3
211416_x_at	11	MGC50550; dJ831C21.2	L20492	20p11.1
202365_at	15	MGC5139	BC004815	12q24.31
220949_s_at	15	MGC5242	NM_024033	7q33
219200_at	13	MGC5297	NM_024091	5p15.3-p15.2

211986_at	15	MGC5395	BG287862	11q12.2
221477_s_at	15	MGC5618	BC001980	6q25.3
211996_s_at	15	MGC70907	BG256504	16p12.3
211685_s_at	15	MGC74858	AF251061	8q22-q23
214023_x_at	14	MGC8685	AL533838	6p25
221593_s_at	15	MGC88191	BC001663	2q12.1
215084_s_at	12	MGC8974	AL031427	1p33-p32.1
62212_at	15	MGC955	W37846	1p34.1
40225_at	15	MGC99654	D88435	4p16
200898_s_at	14	MGEA5	AK002091	10q24.1-q24.3
200899_s_at	15	MGEA5	AK002091	10q24.1-q24.3
211026_s_at	15	MGLL	BC006230	3q21.3 /// 3q21.3
204880_at	15	MGMT	NM_002412	10q26
202291_s_at	15	MGP	NM_000900	12p13.1-p12.3
212576_at	15	MGRN1	AB011116	16p13.3
204168_at	15	MGST2	NM_002413	4q28.3
201403_s_at	15	MGST3	NM_004528	1q23
205904_at	14	MICA	NM_000247	6p21.3
205905_s_at	11	MICA	NM_000247	6p21.3
206275_s_at	14	MICAL2	NM_014632	11p15.3
212472_at	15	MICAL2	BE965029	11p15.3
212473_s_at	15	MICAL2	BE965029	11p15.3
55081_at	14	MICAL-L1	W46406	22q13.1-q13.2
203636_at	15	MID1	BE967532	Xp22
203637_s_at	15	MID1	NM_000381	Xp22
210694_s_at	14	MID1	AF041209	Xp22
218251_at	15	MID1IP1	NM_021242	Xp11.4
208384_s_at	13	MID2	NM_012216	Xq22
214846_s_at	15	MIDORI	AB037751	15q25.2
217871_s_at	15	MIF	BC000447	22q11.23
211558_s_at	15	MIG13	U26266	19p13.2-p13.1
209210_s_at	15	MIG2	Z24725	14q22.1
200984_s_at	15	MIN1	X16447	11p13
213188_s_at	14	MINA53	AI823896	3q12.1-q12.2
214030_at	15	MINA53	BE501352	3q12.1-q12.2
209959_at	10	MINOR	U12767	9q22
209226_s_at	15	MIP	U72069	5q13.2
209751_s_at	15	MIP-2A	AF291676	19q13.43
36830_at	13	MIPEP	U80034	13q12
209585_s_at	15	MIPP	AF084943	10q23
221824_s_at	15	MIR	AA770170	10q11.22
202593_s_at	15	MIR16	NM_016641	16p12-p11.2
221559_s_at	14	MIS12	BC000229	17p13.3
212499_s_at	15	MISS	AK025580	14q22.2
207233_s_at	14	MITF	NM_000248	3p14.2-p14.1
37433_at	11	MIZ1	AF077954	18q21.1
212020_s_at	12	MKI67	AU152107	10q25-qter
205698_s_at	15	MKK6	U39657	17q25.1
218138_at	15	MKKS	NM_018848	20p12
212748_at	15	MKL1	AB037859	22q13

218259_at	15	MKL2	NM_014048	16p13.13
209467_s_at	15	MKNK1	BC002755	1p34.1
218205_s_at	15	MKNK2	NM_017572	19p13.3
215501_s_at	12	MKP5; MKP-5	AK022513	1q41
201285_at	15	MKRN1	NM_013446	7q34
218071_s_at	14	MKRN2	NM_014160	3p25
208082_x_at	15	MKRN4	NM_030757	Xp21.1 /// Xp21.1
204173_at	15	MLC1SA	NM_002475	12q13.13
209742_s_at	15	MLC2; CMH10	AF020768	12q23-q24.3
204783_at	15	MLF1	AI911434	3q25.1
204784_s_at	15	MLF1	NM_022443	3q25.1
218883_s_at	15	MLF1IP	NM_024629	4q35.1
200948_at	15	MLF2	NM_005439	12p13
202520_s_at	15	MLH1	NM_000249	3p21.3
204838_s_at	15	MLH3	NM_014381	14q24.3
214525_x_at	10	MLH3	AB039667	14q24.3
217216_x_at	11	MLH3	AC006530	---
212076_at	12	MLL	AA715041	11q23
203419_at	13	MLL4	NM_014727	19q13.1
205408_at	14	MLLT10	NM_004641	10p12
204917_s_at	14	MLLT3	AV756536	9p22
204918_s_at	13	MLLT3	AV756536	9p22
218211_s_at	14	MLPH	NM_024101	2q37.3
220615_s_at	10	MLSTD1	NM_018099	12p11.23
202519_at	13	MLXIP	NM_014938	12q21.31
218869_at	15	MLYCD	NM_012213	16q23.3
210908_s_at	15	mm-1	AB055804	12q12
217492_s_at	15	MMAC1	AF023139	9p21
203414_at	15	MMD	NM_012329	17q
209700_x_at	15	MMGL	AB042555	1q12
210305_at	15	MMGL	AB042557	1q12
203365_s_at	14	MMP15	NM_002428	16q13-q21
201069_at	15	MMP2	NM_004530	16q13-q21
207118_s_at	13	MMP23A	NM_004659	1p36.3
203936_s_at	15	MMP9	NM_004994	20q11.2-q13.1
160020_at	15	MMP-X1	Z48481	14q11-q12
207012_at	10	MMP-X2	AB009303	8q21
205612_at	11	MMRN1	NM_007351	4q22
219091_s_at	15	MMRN2	NM_024756	10q23.31
202167_s_at	15	MMS19L	NM_022362	10q24-q25
209096_at	15	MMS2	U62136	8q11.21
205330_at	13	MN1	NM_002430	22q11
217317_s_at	15	MN7	AB002391	15q11-q13
220201_at	14	MNAB	NM_018835	9q34
220202_s_at	15	MNAB	NM_018835	9q34
219703_at	15	MNS1	NM_018365	15q21.2
204206_at	15	MNT	NM_020310	17p13.3
201297_s_at	13	MOB1	AK023321	2p13.1
201298_s_at	15	MOBK1B	BC003398	2p13.1
218212_s_at	15	MOCS2	NM_004531	5q11

221820_s_at	15	MOF; hMOF; FLJ14040	AK024102	16p11.2
214650_x_at	11	MOG	AL050328	6p22-p21.3
203956_at	15	MORC2	NM_014941	22q12.2
219038_at	15	MORC4	NM_024657	Xq22.3
221381_s_at	15	MORF4	NM_006792	4q33-q34.1
217982_s_at	15	MORF4L1	NM_006791	15q24
201994_at	15	MORF4L2	NM_012286	Xq22
219527_at	15	MOSC2	NM_017898	1q42.11
218853_s_at	15	MOSPD1	NM_019556	Xq26.3
64883_at	15	MOSPD2	AI744083	Xp22.31
219070_s_at	14	MOSPD3	NM_023948	7q22
209708_at	15	MOX	AY007239	6q23.1-23.3
209758_s_at	15	MP25; MAGP2	U37283	12p13.1-p12.3
210532_s_at	15	MP68; PLPM	AF116639	14q32.33
209149_s_at	13	MP70; HMP70	U94831	14q11.2
209150_s_at	15	MP70; HMP70	U94831	14q11.2
205079_s_at	15	MPDZ	NM_003829	9p24-p22
213306_at	15	MPDZ	AA917899	9p24-p22
212611_at	15	MPEG1	AV728526	11q12.2
203686_at	15	MPG	NM_002434	16p13.3
205235_s_at	12	MPHOSPH1	NM_016195	10q23.32
212885_at	15	MPHOSPH10	AL545921	2p13.2
203740_at	14	MPHOSPH6	NM_005792	16q23.3
221965_at	14	MPHOSPH9	AI990326	12q24.31
203949_at	11	MPO	NM_000250	17q23.1
202974_at	15	MPP1	NM_002436	Xq28
207984_s_at	12	MPP2	NM_005374	17q12-q21
213270_at	11	MPP2	AW450911	17q12-q21
206186_at	14	MPP3	NM_001932	17q12-q21
219321_at	15	MPP5	NM_022474	14q24.1
205413_at	15	MPPED2	NM_001584	11p13
203524_s_at	15	MPST	NM_021126	22q13.1
203466_at	14	MPV17	NM_002437	2p23-p21
201874_at	15	MPZL1	BF978611	1q23.3
201875_s_at	15	MPZL1	NM_024569	1q23.3
206538_at	15	MRAS	NM_012219	3q22.3
209583_s_at	15	MRC	AF063591	3q12-q13
204438_at	14	MRC1	NM_002438	10p13
37408_at	15	MRC2	AB014609	17q24.1
201318_s_at	15	MRCL3	NM_006471	18p11.31
201319_at	15	MRCL3	NM_006471	18p11.31
208896_at	15	MrDb; FLJ33908	X98743	2q14.2
213138_at	11	MRF-1	M62324	2q11.2
209357_at	15	MRG1	AF109161	6q23.3
212197_x_at	15	M-RIP	AB020671	17p11.2
214771_x_at	15	M-RIP	AK025604	17p11.2
221474_at	15	MRLC2; MLC-B	U26162	18p11.31
209380_s_at	15	MRP5	AF146074	3q27
204386_s_at	15	MRP63	NM_024026	---
204387_x_at	15	MRP63	NM_024026	---

221995_s_at	13	MRP63	BF195165	---
219162_s_at	15	MRPL11	NM_016050	11q13.3
203931_s_at	15	MRPL12	NM_002949	17q25
218049_s_at	15	MRPL13	NM_014078	8q22.1-q22.3
218027_at	15	MRPL15	NM_014175	8q11.2-q13
217980_s_at	15	MRPL16	NM_017840	---
217907_at	15	MRPL18	NM_014161	6q25.3
203465_at	15	MRPL19	NM_014763	2q11.1-q11.2
218887_at	15	MRPL2	NM_015950	6p21.3
220526_s_at	15	MRPL20	NM_017971	1p36.3-p36.2
218339_at	15	MRPL22	NM_014180	5q33.1-q33.3
213897_s_at	15	MRPL23	AI832239	11p15.5-p15.4
218270_at	10	MRPL24	NM_024540	1q21-q22
204599_s_at	15	MRPL28	NM_006428	16p13.3
208787_at	15	MRPL3	BC003375	3q21-q23
203781_at	15	MRPL33	NM_004891	2p21
221692_s_at	15	MRPL34	AB049652	19p13.1 /// 19p13.1
218890_x_at	15	MRPL35	NM_016622	2p11.2
218558_s_at	15	MRPL39	NM_017446	21q21.3
218105_s_at	11	MRPL4	NM_015956	---
203152_at	15	MRPL40	NM_003776	22q11.21
217919_s_at	15	MRPL42	AL136659	12q22
218202_x_at	15	MRPL44	NM_022915	2q36.3
219244_s_at	15	MRPL46	NM_022163	15q24-q25
218281_at	15	MRPL48	NM_016055	11q13.3
201717_at	15	MRPL49	NM_004927	11q13
209609_s_at	15	MRPL9	BC004517	---
211594_s_at	15	MRPL9	AB049636	--- /// ---
218106_s_at	15	MRPS10	NM_018141	6p21.1-p12.1
211595_s_at	15	MRPS11	AB049944	15q25 /// 15q25
204331_s_at	15	MRPS12	AA587905	19q13.1-q13.2
203800_s_at	15	MRPS14	BG254653	1q23-1q25
203801_at	15	MRPS14	BG254653	1q24-q25
221437_s_at	15	MRPS15	NM_031280	1p35-p34.1
213092_x_at	13	MRPS16	AW241779	10q22.2
218982_s_at	15	MRPS17	NM_015969	7p11
218385_at	15	MRPS18A	NM_018135	6p21.3
221693_s_at	15	MRPS18a	AB049952	6p21.3 /// 6p21.3
208907_s_at	14	MRPS18B	BC005373	6p21.3
217408_at	15	MRPS18B	AL050361	6p21.3
218001_at	15	MRPS2	NM_016034	9q34
219220_x_at	15	MRPS22	NM_020191	3q23
219819_s_at	15	MRPS28	NM_014018	8q21.1-q21.2
218398_at	15	MRPS30	NM_016640	5q11
222275_at	15	MRPS30	AI039469	5q11
212603_at	15	MRPS31	NM_005830	13q13.3
212604_at	15	MRPS31	AI937794	13q13.3
218654_s_at	15	MRPS33	NM_016071	7q32-q34
218112_at	14	MRPS34	NM_023936	16p13.3
217942_at	15	MRPS35	NM_021821	12p11

212944_at	15	MRPS6	AK024896	21q21.3-q22.1
213164_at	15	MRPS6	AI867198	21q21.3-q22.1
217932_at	15	MRPS7	NM_015971	17q25
218536_at	15	MRS2L	AF052167	6p22.3-p22.1
218538_s_at	14	MRS2L	AF052167	6p22.3-p22.1
219607_s_at	13	MS4A4A	NM_024021	11q12
211364_at	11	MSAP; c86fus	AF109294	9p21
218136_s_at	15	MSCP	NM_018579	8p21.2
221920_s_at	15	MSCP	BE677761	8p21.2
41220_at	15	MSF	AB023208	17q25
208657_s_at	12	MSF	AF142408	17q25
205887_x_at	13	MSH3	NM_002439	5q11-q12
210947_s_at	11	MSH3	J04810	5q11-q12
202911_at	15	MSH6	NM_000179	2p16
204633_s_at	12	MSK1	AF074393	14q31-q32.1
207551_s_at	15	MSL3L1	NM_006800	Xp22.3
200600_at	15	MSN	NM_002444	Xq11.2-q12
219281_at	15	MSRA	NM_012331	8p23.1
218773_s_at	15	MSRB2	NM_012228	10p12
219451_at	12	MSRB2	NM_016064	10p12
209741_x_at	15	MSTP063; KIAA1454	AF119814	15q24
216399_s_at	15	MSTP063; KIAA1454	AK025663	15q24
205932_s_at	14	MSX1	NM_002448	4p16.3-p16.1
213132_s_at	13	MT	AL022237	22q13.31
212859_x_at	15	MT1E	BF217861	16q13
216336_x_at	15	MT1E	AL031602	---
210524_x_at	13	MT1F	AF078844	16q13
213629_x_at	15	MT1F	BF246115	16q13
217165_x_at	13	MT1F	M10943	---
204745_x_at	15	MT1G	NM_005950	16q13
211456_x_at	15	MT1H	AF333388	---
204326_x_at	15	MT1X	NM_002450	16q13 /// 16q13
208581_x_at	15	MT1X	NM_005952	16q13
212185_x_at	15	MT2A	NM_005953	16q13
202247_s_at	14	MTA1	NM_004689	14q32.3
211783_s_at	15	MTA1	BC006177	14q32.3
217761_at	15	MTCBP-1	NM_018269	2p25.3
217772_s_at	15	MTCH2	NM_014342	11p11.2
210212_x_at	15	MTCP1	BC002600	Xq28
216862_s_at	15	MTCP1	Z24459	---
219363_s_at	15	MTERFD1	NM_015942	8q22.1
205323_s_at	11	MTF1	NM_005955	1p33
202309_at	14	MTHFD1	NM_005956	14q24
201761_at	15	MTHFD2	NM_006636	2p13.1
220346_at	15	MTHFD2L	NM_025001	4q21.21
203433_at	15	MTHFS	NM_006441	15q24.3
203095_at	15	MTIF2	NM_002453	2p14-p16
213511_s_at	15	MTMR1	AI167164	Xq28
216095_x_at	15	MTMR1	AF057354	Xq28
205076_s_at	10	MTMR11	NM_006697	1q12-q21

203211_s_at	15	MTMR2	NM_016156	11q22
202197_at	15	MTMR3	NM_021090	22q12.2
212277_at	14	MTMR4	AB014547	17q22-q23
214268_s_at	15	MTMR4	AL042220	17q22-q23
214429_at	15	MTMR6	U47635	13q12
204837_at	15	MTMR9	AL080178	8p23-p22
213278_at	15	MTMR9	AW014788	8p23-p22
218716_x_at	15	MTO1	NM_012123	6q14.1
222014_x_at	15	MTO1	AI249752	6q14.1
203774_at	15	MTR	NM_000254	1q43
203200_s_at	15	MTRR	N29717	5p15.3-p15.2
212096_s_at	15	MTSG1	AL096842	8p22
203037_s_at	15	MTSS1	NM_014751	8p22
212093_s_at	15	MTUS1	AI695017	8p22
212095_s_at	13	MTUS1	BE552421	8p22
212484_at	10	MTVR1	BF974389	11q23
32209_at	15	MTVR1	AF052151	11q23
210386_s_at	15	MTX1	BC001906	1q21
203517_at	15	MTX2	NM_006554	2q31.2
215333_x_at	15	MU	X08020	1p13.3
217117_x_at	12	MUC3	AF007194	7q22
213009_s_at	15	MUL	AK022701	17q22-q23
218568_at	15	MULK	NM_018238	7q34
221290_s_at	12	MUM1	NM_016473	19p13.3
218463_s_at	15	MUS81	NM_025128	11q13
202959_at	15	MUT	AI433712	6p21
202960_s_at	15	MUT	NM_000255	6p21
207727_s_at	15	MUTYH	NM_012222	1p34.3-p32.1
202086_at	12	MX1	NM_002462	21q22.3
204994_at	15	MX2	NM_002463	21q22.3
210778_s_at	10	MXD4	BC002713	4p16.3
202364_at	15	MXI1	NM_005962	10q24-q25
213422_s_at	15	MXRA8	AW888223	1p36.33
221498_at	15	MY014	AF060509	1q21.3
209836_x_at	15	My016	AF060511	16p12.1
212781_at	13	MY038	AK026954	16p12.2
212783_at	15	MY038	AK026954	16p12.2
213906_at	15	MYBL1	AW592266	8q22
208040_s_at	15	MYBPC3	NM_000256	11p11.2 /// 11p11.2
203359_s_at	15	MYCBP	NM_012333	1p33-p32.2
201960_s_at	15	MYCBP2	NM_015057	13q22
209757_s_at	15	MYCN	BC002712	2p24.1
220471_s_at	12	MYCT1	NM_025107	6q25.1
209124_at	15	MYD88	U70451	3p22
201497_x_at	10	MYH11	NM_022844	16p13.13-p13.12
215795_at	15	MYH14	AK000947	20q11.23
208148_at	10	MYH4	NM_017533	17p13.1
204737_s_at	15	MYH7	NM_000257	14q12
216265_x_at	15	MYH7	AI292276	14q12
211926_s_at	15	MYH9	AI827941	22q13.1

211014_s_at	15	MYL	AF230410	15q22
205589_at	15	MYL3	NM_000258	3p21.3-p21.2
216054_x_at	15	MYL4	X58851	17q21-qter
210395_x_at	15	MYL4	AF116676	17q21-qter
205144_at	15	MYL5	L03785	4p16.3
205145_s_at	11	MYL5	NM_002477	4p16.3
212082_s_at	15	MYL6	BE734356	12q13.13
214002_at	15	MYL6	AA419227	12q13.13
219942_at	15	MYL7	NM_021223	7p21-p11.2
201058_s_at	15	MYL9	NM_006097	20q11.23
221659_s_at	14	MYLC2PL	BC002778	7q22.1
220319_s_at	14	MYLIP	NM_013262	6p23-p22.3
202555_s_at	14	MYLK	NM_005965	3q21
218926_at	15	MYNN	NM_018657	3q26.31
201976_s_at	14	MYO10	NM_012334	5p15.1-p14.3
59375_at	13	MYO15B	AI825877	---
212364_at	15	MYO1B	BF432550	2q12-q34
212365_at	15	MYO1B	BF215996	2q12-q34
214656_x_at	10	MYO1C	BE790157	17p13
32811_at	14	MYO1C	X98507	17p13
203072_at	11	MYO1E	NM_004998	15q21-q22
204527_at	13	MYO5A	NM_000259	15q21
218966_at	15	MYO5C	NM_018728	15q21
203216_s_at	14	MYO6	NM_004999	6q13
208962_s_at	15	MYO6	BE540552	11q12.2-q13.1
206656_s_at	15	MYOD1	BC000353	11p15.4
205610_at	15	MYOM1	NM_003803	18p11.31-p11.32
205826_at	14	MYOM2	NM_003970	8p23.3
219728_at	15	MYOT	NM_006790	5q31
219509_at	13	MYOZ1	NM_021245	10q22.1
207148_x_at	15	MYOZ2	NM_016599	4q26-q27
213782_s_at	15	MYOZ2	BF939176	4q26-q27
201957_at	15	MYPT2; MGC87886	AF324888	1q32.1
214780_s_at	12	MYR5; myosin	AK002201	19p13.1
200049_at	15	MYST2	NM_007067	17q21.32
202423_at	15	MYST3	NM_006766	8p11
212462_at	15	MYST4	AU144267	10q22.2
214496_x_at	15	MYST4	NM_012330	10q22.2
212406_s_at	15	MYT1	AB028973	20q13.33
209228_x_at	15	N33	U42349	8p22
221867_at	10	N4BP1	BF436315	16q12.1
32069_at	15	N4BP1	AB014515	16q12.1
48612_at	15	N4BP1	AA225490	16q12.1
214775_at	14	N4BP3	AW139448	5q35.3
209272_at	15	NAB1	AF045451	2q32.3-q33
211139_s_at	15	NAB1	AF045452	2q32.3-q33
212803_at	13	NAB2	BF337329	12q13
200735_x_at	15	NACA	NM_005594	12q23-q24.1
208635_x_at	15	NACA	BF976260	12q23-q24.1
222018_at	14	NACA	AI992187	12q23-q24.1

211939_x_at	15	NACB	X74070	5q13.3
211546_x_at	15	NACP	L36674	4q21
210153_s_at	15	NAD(P)+ -dependent malic enz	M55905	6p25-p24
202926_at	15	NAG	NM_015909	2p24
202943_s_at	10	NAGA	NM_000262	22q13-qter
218231_at	15	NAGK	NM_017567	2p13.2
204360_s_at	14	NAGLU	NM_000263	17q21
218380_at	14	NALP1	NM_021730	17p13
200597_at	15	NANOS1	AI123320	10q26
218189_s_at	15	NANS	NM_018946	9p24.1-p23
47530_at	15	NAP1	AA748492	9q31.1
204528_s_at	15	NAP1L1	NM_004537	12q21.1
208752_x_at	15	NAP1L1	AI888672	12q21.1
208753_s_at	15	NAP1L1	BC002387	12q21.1
208754_s_at	15	NAP1L1	AI888672	12q21.1
212967_x_at	15	NAP1L1	AW148801	12q21.1
213864_s_at	15	NAP1L1	AI985751	12q21.1
204749_at	15	NAP1L3	NM_004538	Xq21.3-q22
201414_s_at	15	NAP1L4	NM_005969	11p15.5
206491_s_at	15	NAPA	NM_003827	19q13.33
208751_at	15	NAPA	BC001165	19q13.33
210048_at	15	NAPG	BC001889	18p11.21
219862_s_at	15	NARF	NM_012336	17q25.3
219378_at	15	NARG1L	NM_024561	13q13.3
218713_at	15	NARG2	NM_024611	15q21.3
200027_at	15	NARS	NM_004539	18q21.2-q21.3
201969_at	15	NASP	AW003362	1p34.1
201970_s_at	15	NASP	NM_002482	1p34.1
214911_s_at	15	NAT	S78771	6p21.3
214440_at	15	NAT1	NM_000662	8p23.1-p21.3
204382_at	14	NAT9	NM_015654	17q25.2
218330_s_at	15	NAV2	NM_018162	11p15.1
37005_at	15	NB	D28124	1p36.13-p36.11
210739_x_at	15	NBC	AF069510	4q21
221207_s_at	15	NBEA	NM_015678	13q13
217987_at	15	NBLA00058	NM_019048	2p24.3-q21.3
219342_at	15	NBLA04196	NM_022900	7q21.3
202907_s_at	15	NBN	NM_002485	8q21
201103_x_at	15	NBPF14	NM_015383	---
201104_x_at	15	NBPF14	NM_015383	1q21.2
201384_s_at	15	NBR1	NM_005899	17q21.1
202905_x_at	13	NBS1	AI796269	8q21
217299_s_at	11	NBS1	AK001017	8q21
203440_at	15	NCAD	M34064	18q11.2
217359_s_at	15	NCAM	M22094	11q23.1
212843_at	15	NCAM1	AA126505	11q23.1
214952_at	13	NCAM1	BF348061	11q23.1
205669_at	11	NCAM2	NM_004540	21q21.1
209520_s_at	15	NCBP1	BC001450	9q34.1
201517_at	15	NCBP2	BC001255	3q29

201521_s_at	12	NCBP2	NM_007362	3q29
209556_at	13	NCDN	AB011179	1p34.3
204725_s_at	15	NCK1	AI591085	3q21
211063_s_at	15	NCK1	BC006403	3q21 /// 3q21
203315_at	10	NCK2	BC000103	2q12
207738_s_at	15	NCKAP1	NM_013436	2q32
218697_at	15	NCKIPSD	NM_016453	3p21
200610_s_at	15	NCL	NM_005381	2q12-qter
209105_at	14	NCOA1	AI672428	2p23
209106_at	15	NCOA1	BF576458	2p23
212867_at	15	NCOA2	AI040324	8q13.2
207700_s_at	15	NCOA3	NM_006534	20q12
209060_x_at	14	NCOA3	AI438999	20q12
209061_at	15	NCOA3	AI761748	20q12
210774_s_at	15	NCOA4	AL162047	10q11.2
219231_at	14	NCOA6IP	NM_024831	8q11
200854_at	15	NCOR1	AB028970	17p11.2
200855_at	12	NCOR1	AW771910	17p11.2
200856_x_at	14	NCOR1	BF437948	17p11.2
200857_s_at	15	NCOR1	NM_006311	17p11.2
207760_s_at	15	NCOR2	NM_006312	12q24
217045_x_at	11	NCR2	AL136967	6p21.1
211805_s_at	10	NCX1	AF108389	2p23-p22
201362_at	15	ND1	AF205218	1q25.1-q31.1
218414_s_at	14	NDE1	NM_017668	16p13.11
208093_s_at	15	NDEL1	NM_030808	17p13.1 /// 17p13.1
217800_s_at	15	NDFIP1	NM_030571	5q31.3
209550_at	15	NDN	U35139	15q11.2-q12
210817_s_at	15	NDP52	BC004130	17q21.33
200632_s_at	15	NDRG1	NM_006096	8q24.3
206453_s_at	15	NDRG2	NM_016250	14q11.2
217286_s_at	15	NDRG3	BC001805	---
209159_s_at	15	NDRG4	AV724216	16q21-q22.1
202607_at	15	NDST1	AL526632	5q33.1
202298_at	15	NDUFA1	NM_004541	Xq25-q26
217860_at	15	NDUFA10	NM_004544	2q37.3
220864_s_at	15	NDUFA13	NM_015965	19p13.2
209224_s_at	15	NDUFA2	BC003674	5q31
218563_at	15	NDUFA3	NM_004542	19q13.42
217773_s_at	15	NDUFA4	NM_002489	7p21.3
201304_at	15	NDUFA5	NM_005000	7q32
202000_at	15	NDUFA6	BC002772	22q13.2-q13.31
202001_s_at	15	NDUFA6	BC002772	22q13.2-q13.31
202785_at	14	NDUFA7	NM_005001	19p13.2
218160_at	15	NDUFA8	NM_014222	9q33.2-q34.11
202077_at	15	NDUFAB1	NM_005003	16p12.3
204125_at	15	NDUFAF1	NM_016013	15q11.2-q21.3
206790_s_at	15	NDUFB1	NM_004545	14q32.13
218320_s_at	15	NDUFB11	NM_019056	Xp11.3
218200_s_at	15	NDUFB2	NM_004546	7q34

218201_at	15	NDUFB2	NM_004546	7q34
203371_s_at	15	NDUFB3	NM_002491	2q31.3
218226_s_at	15	NDUFB4	NM_004547	3q13.33
203621_at	15	NDUFB5	NM_002492	3q27.1
203613_s_at	15	NDUFB6	NM_002493	9p13.3
202839_s_at	15	NDUFB7	NM_004146	19p13.12-p13.11
201226_at	15	NDUFB8	NM_005004	10q23.2-q23.33
201227_s_at	15	NDUFB8	NM_005004	10q23.2-q23.33
214241_at	15	NDUFB8	AA723057	10q23.2-q23.33
203478_at	15	NDUFC1	NM_002494	4q28.2-q31.1
206936_x_at	15	NDUFC2	NM_022335	11q13.4
218101_s_at	15	NDUFC2	NM_004549	11q13.4
203039_s_at	15	NDUFS1	NM_005006	2q33-q34
201966_at	15	NDUFS2	NM_004550	1q23
208969_at	15	NDUFS2L	AF050641	12p13.3
201740_at	15	NDUFS3	NM_004551	11p11.11
209303_at	15	NDUFS4	BC005270	5q11.1
201757_at	15	NDUFS5	NM_004552	1p34.2-p33
203606_at	15	NDUFS6	NM_004553	5p15.33
211752_s_at	15	NDUFS7	BC005954	19p13.3 /// 19p13.3
203189_s_at	15	NDUFS8	NM_002496	11q13
203190_at	15	NDUFS8	NM_002496	11q13
202941_at	15	NDUFV2	NM_021074	18p11.31-p11.2
203961_at	15	NEBL	AL157398	10p12
203962_s_at	15	NEBL	NM_006393	10p12
207279_s_at	15	NEBL	NM_016365	10p12 /// 10p12
216882_s_at	15	NEBL	AL157398	10p12
217585_at	10	NEBL	BE502910	10p12
209300_s_at	15	NECAP1	BC002888	12p13.31
220731_s_at	11	NECAP2	NM_018090	1p36.13
209032_s_at	15	NECL2	AF132811	11q23.2
213012_at	15	NEDD4	D42055	15q
212445_s_at	15	NEDD4L	AI357376	18q21
201840_at	15	NEDD8	NM_006156	14q11.2
202149_at	15	NEDD9	AL136139	6p25-p24
219502_at	13	NEIL3	NM_018248	4q34.3
213331_s_at	15	NEK1	AV700007	4q33
219542_at	10	NEK11	NM_024800	3q22.1
204641_at	15	NEK2	NM_002497	1q32.2-q41
213116_at	15	NEK3	AI191920	13q14.13
204634_at	14	NEK4	NM_003157	3p21.1
212530_at	15	NEK7	AL080111	1q31.3
212299_at	15	Nek8	AL117502	14q24.2
221214_s_at	15	NELF	NM_015537	9q34.3
218407_x_at	15	NENF	NM_013349	1q32.3
204321_at	15	NEO1	NM_002499	15q22.3-q23
214934_at	13	NEO1L	AW411030	18q23
218678_at	15	NES	NM_024609	1q23.1
201829_at	15	NET1	AW263232	10p15
201830_s_at	15	NET1	NM_005863	10p15

209890_at	15	NET-4	AF065389	4q23
218888_s_at	14	NETO2	AI335263	16q11
216836_s_at	15	NEU	X03363	17q11.2-q12
208926_at	15	NEU; SIAL1	U84246	6p21.3
212676_at	14	NF1	AW293356	17q11.2
212678_at	13	NF1	AW054826	17q11.2
213438_at	11	NFASC	AA995925	---
210555_s_at	12	NFAT4; NFATX	U85430	16q22.2
208003_s_at	10	NFAT5	NM_006599	16q22.1
211105_s_at	12	NF-ATc/C	U80918	18q23
212808_at	14	NFATC2IP	AI884627	16p12.1
212809_at	14	NFATC2IP	AA152202	16p12.1
217526_at	14	NFATC2IP	AI478300	16p12.1
217527_s_at	15	NFATC2IP	AI478300	16p12.1
200758_s_at	15	NFE2L1	NM_003204	17q21.3
200759_x_at	15	NFE2L1	NM_003204	17q21.3
214179_s_at	15	NFE2L1	H93013	17q21.3
201146_at	15	NFE2L2	NM_006164	2q31
209289_at	15	NFIB	AI700518	9p24.1
209290_s_at	15	NFIB	BC001283	9p24.1
213029_at	15	NFIB	BG478428	9p24.1
213032_at	14	NFIB	AI186739	9p24.1
203574_at	15	NFIL3	NM_005384	9q22
203973_s_at	15	NF-IL6-beta	M83667	8q11.21
209239_at	13	NFKB1	M55643	4q24
201502_s_at	15	NFKBIA	NM_020529	14q13
206968_s_at	14	NFRKB	NM_006165	11q24-q25
218455_at	15	NFS1	NM_021100	20q11.23
218127_at	15	NFYB	BC005316	12q22-q23
218128_at	15	NFYB	BC005316	12q22-q23
218129_s_at	15	NFYB	BC005316	12q22-q23
202215_s_at	15	NFYC	NM_014223	1p32
202216_x_at	15	NFYC	BC005003	1p32
39966_at	15	NGC; MGC44034	AF059274	3p21.3
217963_s_at	15	NGFRAP1	NM_014380	Xq22.2
220742_s_at	15	NGLY1	NM_018297	3p24.1
217722_s_at	15	NGRN	NM_016645	15q26.1
208829_at	14	NGS-17	AF029750	6p21.3
219353_at	15	NHLRC2	NM_017687	10q26.11
201076_at	15	NHP2L1	NM_005008	22q13.2-q13.31
201077_s_at	15	NHPX; OTK27	AF155235	22q13.2-q13.31
217966_s_at	15	NIBAN	AF288391	1q25
217967_s_at	15	NIBAN	AF288391	1q25
202007_at	15	NID	BF940043	1q43
202008_s_at	15	NID1	NM_002508	1q43
204114_at	15	NID2	NM_007361	14q21-q22
218133_s_at	13	NIF3L1	NM_021824	2q33
218334_at	15	NIF3L1BP1	NM_025075	3p21.1
202475_at	15	NIFIE14	NM_006326	19q13.1 /// 19q13.1
217896_s_at	10	NIP30	NM_024946	16q13

212129_at	15	NIPA2	AI589507	15q11.2
207108_s_at	12	NIPBL	NM_015384	5p13.2
213918_s_at	15	NIPBL	BF221673	5p13.2
201708_s_at	14	NIPSNAP1	AW083371	22q12.2
201709_s_at	15	NIPSNAP1	NM_003634	22q12.2
221104_s_at	15	NIPSNAP3B	NM_018376	9q31.3
201591_s_at	15	NISCH	NM_007184	3p21.1
202891_at	15	NIT1	NM_005600	1q21-q22
218557_at	15	NIT2	NM_020202	3q12.3
203830_at	13	NJMU-R1	NM_022344	17q12
208680_at	15	NKEFA	L19184	1p34.1
211658_at	15	NKEFB	L19185	19p13.2
39729_at	15	NKEFB	L19185	19p13.2
218240_at	15	NKIRAS2	NM_017595	17q21.31
222105_s_at	15	NKIRAS2	AA452565	---
205004_at	12	NKRF	NM_017544	Xq25
202379_s_at	15	NKTR	NM_005385	3p23-p21
202380_s_at	15	NKTR	NM_005385	3p23-p21
215338_s_at	13	NKTR	AI688640	3p23-p21
206578_at	15	NKX2-5	NM_004387	5q34
209706_at	12	NKX3A; NKX3.1	AF247704	8p21
205893_at	15	NLGN1	NM_014932	3q26.32
218318_s_at	15	NLK	NM_016231	17q11.2
201577_at	15	NME1	NM_000269	17q21.3
201268_at	15	NME2	NM_002512	17q21.3
212739_s_at	15	NME4	AL523860	16p13.3
205851_at	14	NME6	BC001808	3p21
219553_at	15	NME7	NM_013330	1q24
203964_at	13	NMI	NM_004688	2p24.3-q21.3
212372_at	15	NMMHCB	AK026977	17p13
201157_s_at	15	NMT	AF020500	17q21.31
205005_s_at	14	NMT2	AW293531	10p13
205006_s_at	15	NMT2	NM_004808	10p13
206023_at	12	NMU	NM_006681	4q12
202237_at	13	NNMT	NM_006169	11q23.1
202238_s_at	11	NNMT	NM_006169	11q23.1
218889_at	15	NOC3L	NM_022451	10q23.33
214427_at	10	NOL1	NM_006170	12p13
200874_s_at	15	NOL5A	NM_006392	20p13
200875_s_at	15	NOL5A	NM_006392	20p13
202882_x_at	15	NOL7	NM_016167	6p23
210097_s_at	15	NOL7	AF130102	6p23 /// 6p23
218244_at	15	NOL8	NM_017948	9q22.32
218754_at	15	NOL9	NM_024654	1p36.23
219110_at	14	NOLA1	NM_018983	4q25
209104_s_at	15	NOLA2	BC000009	5q35.3
217962_at	15	NOLA3	NM_018648	15q14-q15
205895_s_at	15	NOLC1	NM_004741	10q24.32
211951_at	15	NOLC1	AI355279	10q24.32
221853_s_at	15	NOMO3	N39536	16p13.12

200057_s_at	15	NONO	NM_007363	Xq13.1
208698_s_at	15	NONO	BC002364	Xq13.1
210470_x_at	13	NONO	BC003129	Xq13.1
217950_at	15	NOSIP	NM_015953	19q13.33
218902_at	15	NOTCH1	NM_017617	9q34.3
202443_x_at	15	NOTCH2	NM_024408	1p13-p11
212377_s_at	15	NOTCH2	AU158495	1p13-p11
214722_at	15	NOTCH2NL	AW516297	1q21.2
203238_s_at	15	NOTCH3	NM_000435	19p13.2-p13.1
205247_at	15	NOTCH4	NM_004557	6p21.3
204501_at	12	NOV	NM_002514	8q24.1
214321_at	15	NOV	BF440025	8q24.1
205794_s_at	15	NOVA1	NM_002515	14q
219773_at	14	NOX4	NM_016931	11q14.2-q21
201695_s_at	15	NP	NM_000270	14q13.1
211358_s_at	15	NP94	AF234161	9q34.1
218506_x_at	15	N-PAC	NM_018459	16p13.3
222115_x_at	14	N-PAC	BC003693	16p13.3
218294_s_at	14	NPAP60	AF267865	22q13.31
205459_s_at	10	NPAS2	NM_002518	2q11.2
213462_at	11	NPAS2	AW000928	2q11.2
39548_at	14	NPAS2	U77970	2q11.2
39549_at	15	NPAS2	AI743090	2q11.2
209798_at	15	NPAT	D83243	11q22-q23
202679_at	15	NPC1	NM_000271	18q11-q12
200701_at	15	NPC2	NM_006432	14q24.3
200941_at	15	NPC-A-13	AK026575	16q23.3
218446_s_at	15	NPD008	AF223467	17p11.2
210574_s_at	15	NPD011	AF241788	1p35-p34
209378_s_at	15	NPD012	AF241785	10q23.2
209379_s_at	15	NPD012	AF241785	10q23.2
209006_s_at	14	NPD014	AF247168	1p36.13-p35.1
209007_s_at	15	NPD014	AF247168	1p36.13-p35.1
218086_at	13	NPDC1	NM_015392	9q34.3
89476_r_at	14	NPEPL1	AA398062	20q13.32
201454_s_at	15	NPEPPS	NM_006310	17q21
214101_s_at	10	NPEPPS	BG153399	17q21
213471_at	11	NPHP4	AB014573	1p36.22
204538_x_at	15	NPIP	NM_006985	16p13.13
217796_s_at	15	NPL4	NM_017921	17qter
200063_s_at	15	NPM1	BC002398	5q35
221923_s_at	15	NPM1	AA191576	5q35
206801_at	15	NPPB	NM_002521	1p36.2
214066_x_at	10	NPR2	AA565715	9p21-p12
219789_at	12	NPR3	NM_000908	5p14-p13
209201_x_at	13	NPYR	L01639	2q21
201468_s_at	13	NQO1	NM_000903	16q22.1
210519_s_at	12	NQO1	BC000906	16q22.1
203814_s_at	15	NQO2	NM_000904	6pter-q12
209750_at	12	NR1D2	N32859	3p24.1

218215_s_at	15	NR1H2	NM_007121	19q13.3-19q13.3
203920_at	15	NR1H3	NM_005693	11p11.2
204791_at	15	NR2C1	NM_003297	12q23.1
209505_at	11	NR2F1	AI951185	5q14
209120_at	15	NR2F2	AL037401	15q26
215073_s_at	14	NR2F2	AL554245	15q26
209262_s_at	11	NR2F6	BC002669	19p13.1
201865_x_at	15	NR3C1	AI432196	---
201866_s_at	12	NR3C1	NM_000176	5q31
211671_s_at	15	NR3C1	U01351	5q31 /// 5q31
205259_at	15	NR3C2	NM_000901	4q31.1
210904_s_at	15	NR4; IL-13Ra	U81380	Xq24
202340_x_at	15	NR4A1	NM_002135	12q13
204622_x_at	11	NR4A2	NM_006186	2q22-q23
207978_s_at	11	NR4A3	NM_006981	9q22
209014_at	15	NRAGE; DLXIN-1	AF217963	Xp11.23
202647_s_at	15	NRAS	NM_002524	1p13.2
221803_s_at	14	NRBF2	AA883074	10q22.1
217765_at	15	NRBP1	NM_013392	2p23
208979_at	15	NRC	AF128458	20q11
208709_s_at	15	NRD1	U64898	1p32.2-p32.1
204651_at	15	NRF1	AW003022	7q32
204652_s_at	11	NRF1	NM_005011	7q32
202599_s_at	15	NRIP1	NM_003489	21q11.2
202600_s_at	13	NRIP1	AI824012	21q11.2
219557_s_at	10	NRIP3	AJ400877	11p15.3
218625_at	15	NRN1	NM_016588	6p25.1
210510_s_at	14	NRP	AF145712	10p12
212298_at	15	NRP1	BE620457	10p12
214632_at	11	NRP2	AA295257	2q33.3
221238_at	10	NSBP1	NM_030763	Xq13.3 /// Xq13.3
209279_s_at	12	NSDHL	BC000245	Xq28
215093_at	14	NSDHL	U82671	Xq28
208627_s_at	15	NSEP1	BE966374	1p34
217830_s_at	15	NSFL1C	NM_016143	20p13
220248_x_at	15	NSFL1C	NM_018839	20p13
203269_at	15	NSMAF	NM_003580	8q12-q13
210178_x_at	14	NSSR	AF047448	1p36.11
203802_x_at	15	NSUN5	NM_018044	7q11.23
213773_x_at	15	NSUN5	AW248552	7q11.23
209155_s_at	15	NT5C2	BC001595	10q24.33
203939_at	12	NT5E	NM_002526	6q14-q21
213061_s_at	15	NTAN1	AA643304	16p13.13
213062_at	15	NTAN1	AA643304	16p13.13
203718_at	13	NTE	NM_006702	19p13.3-p13.2
207152_at	14	NTRK2	NM_006180	9q22.1
221795_at	10	NTRK2	AA707199	9q22.1
221796_at	14	NTRK2	AA707199	9q22.1
206291_at	14	NTS	NM_006183	12q21
204589_at	15	NUAK1	NM_014840	12q24.11

220987_s_at	10	NUAK2	NM_030952	1q32.1 /// 1q32.1
203978_at	15	NUBP1	NM_002484	16p13.2
218227_at	14	NUBP2	NM_012225	16p13.3
220176_at	13	NUBPL	NM_025152	14q12
200646_s_at	14	NUCB1	NM_006184	19q13.2-q13.4
200649_at	15	NUCB1	BC002356	19q13.2-q13.4
203675_at	15	NUCB2	NM_005013	11p15.1-p14
217802_s_at	15	NUCKS1	NM_022731	1q32.1
201173_x_at	15	NUDC	NM_006600	1p35-p34
214136_at	14	NUDT13	W80642	10q22.3
219347_at	15	NUDT15	NM_018283	13q14.12
218609_s_at	10	NUDT2	NM_001161	9p13
202697_at	15	NUDT21	NM_007006	16q13
211973_at	11	NUDT3	AW341200	---
212605_s_at	13	NUDT3	AK025759	---
206302_s_at	15	NUDT4	NM_019094	---
206303_s_at	15	NUDT4	NM_019094	---
212181_s_at	15	NUDT4	AF191654	---
212183_at	15	NUDT4	AB007956	---
218375_at	15	NUDT9	NM_024047	4q22.1
200747_s_at	15	NUMA1	NM_006185	11q13
218768_at	15	NUP107	NM_020401	12q14.3
202184_s_at	15	NUP133	NM_018230	1q42.13
202097_at	15	NUP153	NM_005124	6p22.3
212709_at	14	Nup160	D83781	11p11.12
212247_at	15	NUP205	AW008531	7q33
202155_s_at	13	NUP214	NM_005085	9q34.1
218622_at	15	NUP37	NM_024057	12q23.3
219007_at	15	NUP43	NM_024647	6q24.3
213682_at	15	NUP50	AL036344	22q13.31
218256_s_at	15	NUP54	NM_017426	4q21.21
202153_s_at	14	NUP62	NM_016553	19q13.33
218014_at	15	NUP85	NM_024844	17q25.2
202900_s_at	15	NUP88	NM_002532	17p13.2
203195_s_at	15	NUP98	NM_005387	11p15.5
210793_s_at	14	NUP98	U41815	11p15.5
204435_at	14	NUPL1	NM_014778	13q12.13
204003_s_at	10	NUPL2	NM_007342	7p15
218039_at	15	NUSAP1	NM_016359	15q14
219978_s_at	13	NUSAP1	NM_018454	15q14
202397_at	15	NUTF2	NM_005796	16q22.1
208922_s_at	15	NXF1	BC004904	11q12-q13
218708_at	15	NXT1	NM_013248	20p12-p11.2
213241_at	15	NY-REN-58	AF035307	12q23.3
213288_at	15	OACT2	AI761250	2p25.2
201599_at	15	OAT	NM_000274	10q26
215947_s_at	15	OAZ1	AF090094	19p13.3
215952_s_at	15	OAZ1	AF090094	19p13.3
201364_s_at	15	OAZ2	AF242521	15q22.1
201365_at	13	OAZ2	NM_002537	15q22.1

212461_at	15	OAZIN	BF793951	8q22.3
219100_at	15	OBFC1	NM_024928	10q25.1
211355_x_at	12	OBR	U52914	1p31
211356_x_at	11	OBR	U66495	1p31
222324_at	14	OBSCN	AI344415	---
208274_at	11	OCLM	NM_022375	---
203446_s_at	14	OCRL	NM_000276	Xq25-q26.1
205074_at	14	OCTN2	AB015050	5q31
209731_at	15	OCTS3	U79718	16p13.3
213018_at	15	ODAG	AI337901	7q21-q22
214718_at	15	ODAG	AK026142	7q21-q22
200790_at	15	ODC1	NM_002539	2p25
203569_s_at	15	OFD1	NM_003611	Xp22.2-p22.3
210135_s_at	12	OG12	AF022654	3q25-q26.1
209003_at	15	OGC; SLC20A4	AF070548	17p13.3
201282_at	15	OGDH	NM_002541	7p14-p13
219277_s_at	12	OGDHL	NM_018245	10q11.23
210443_x_at	10	OGFR	AF172452	20q13.3
205301_s_at	12	OGG1	NM_016820	3p26.2
205760_s_at	15	OGG1	NM_016821	3p26.2
218730_s_at	15	OGN	AF202167	9q22
207564_x_at	15	OGT	NM_003605	Xq13
212307_s_at	15	OGT	BF001665	Xq13
209129_at	15	OIP1	AF000974	7q22
214924_s_at	15	OIP106	AK000754	3p25.3-p24.1
215136_s_at	15	OIP2	AL050353	13q13.1
213599_at	14	OIP5	BE045993	15q14
213131_at	11	OLFM1	R38389	9q34.3
217525_at	15	OLFML1	AW305097	11p15.4
213075_at	15	OLFML2A	AL050002	9q34.11
213125_at	15	OLFML2B	AW007573	1q23.1
218162_at	15	OLFML3	NM_020190	1p13.1
205907_s_at	12	OMD	AI765819	9q22.32
211152_s_at	15	OMI	AF184911	2p12
212213_x_at	15	OPA1	AB011139	3q28-q29
212214_at	10	OPA1	BF439570	3q28-q29
214306_at	15	OPA1	AA209332	3q28-q29
206323_x_at	15	OPHN1	NM_002547	Xq12
222025_s_at	14	OPLAH	AI991887	8q24.3
209875_s_at	15	OPN	M83248	4q21-q25
219032_x_at	15	OPN3	NM_014322	1q43
201692_at	14	OPRS1	NM_005866	9p13.2
202073_at	15	OPTN	AV757675	10p14
202074_s_at	15	OPTN	AV757675	10p14
221464_at	10	OR1D2	NM_002548	17p13-p12
221460_at	10	OR2C1	NM_012368	16p13.3
222327_x_at	10	OR7E156P	AA459867	---
217499_x_at	15	OR7E76	AW874308	---
210028_s_at	15	ORC3	AF125507	6q14.3-q16.1
203351_s_at	15	ORC4L	NM_002552	2q22-q23

203352_at	10	ORC4L	NM_002552	2q22-q23
204957_at	15	ORC5L	NM_002553	7q22.1
211212_s_at	11	ORC5L	AF081459	7q22.1
219105_x_at	14	ORC6L	NM_014321	16q12
218556_at	15	ORMDL2	NM_014182	12q13.13
209485_s_at	15	ORP1	AF274714	18q11.1
212582_at	15	ORP8	AL049923	12q14
208735_s_at	15	OS4; PSR2; SCP2	AF022231	12q13-q15
200714_x_at	15	OS9	NM_006812	12q13
215399_s_at	15	OS-9	AI683900	12q13
201800_s_at	15	OSBP1	AF185696	11q12-q13
219073_s_at	14	OSBPL10	NM_017784	3p22.3
218304_s_at	15	OSBPL11	NM_022776	3q21
208158_s_at	15	OSBPL1A	NM_018030	18q11.1
209221_s_at	15	OSBPL2	AI753638	20q13.3
209222_s_at	15	OSBPL2	AI753638	20q13.3
212585_at	15	OSBPL8	BF970829	12q14
218047_at	15	OSBPL9	NM_024586	1p32.3
210809_s_at	15	osf-2	D13665	13q13.3
207173_x_at	15	osf-4	D21254	16q22.1
209450_at	12	OSGEP	AB050442	14q11.2
205729_at	10	OSMR	NM_003999	5p13.2
219421_at	15	OSRF	NM_012382	5p15.2-p12
218196_at	15	OSTM1	NM_014028	6q21
201245_s_at	15	OTUB1	AL523776	11q13.1
38710_at	15	OTUB1	AL096714	11q13.1
203480_s_at	15	OTUD4	NM_014928	4q31.21
208717_at	15	OXA1L	BC001669	14q11.2
202780_at	15	OXCT1	NM_000436	5p13
218197_s_at	15	OXR1	NM_018002	8q23
219133_at	15	OXSM	NM_017897	3p24.1
202696_at	15	OXSR1	NM_005109	3p22-p21.3
206825_at	12	OXTR	NM_000916	3p25
221539_at	15	P/OKcl.6	AB044548	8p12
203879_at	15	p110D	U86453	1p36.2
213039_at	10	P114-RHO-GEF	AB011093	19p13.3
209175_at	15	P125; MSTP053	AK001135	10q25-q26
212331_at	15	p130	X76061	16q12.2
212932_at	12	P130	AK022494	2q21.3
202373_s_at	15	p150	AF255648	1q42.11
218209_s_at	14	P15RS	NM_018170	18q12.2
211792_s_at	11	p18; INK4C; p18-INK4C	U17074	1p32
210240_s_at	13	p19	U20498	19p13
208795_s_at	15	P1cdc47	D55716	7q21.3-q22.1
208764_s_at	15	P2	D13119	12q13.13
204088_at	14	P2RX4	NM_002560	12q24.32
221466_at	12	P2RY4	NM_002565	Xq13
218589_at	15	P2RY5	NM_005767	13q14
207543_s_at	15	P4HA1	NM_000917	10q21.3-q23.1
202733_at	15	P4HA2	NM_004199	5q31

200654_at	15	P4HB	J02783	17q25
200656_s_at	15	P4HB	NM_000918	17q25
209953_s_at	12	P50CDC37	U63131	19p13.2
209337_at	15	p52	AF063020	9p22.2
210758_at	10	p52	AF098482	9p22.2
218403_at	15	P53CSV	NM_016399	12q24.31
210241_s_at	15	P53TG1	AB007458	7q21.1
210886_x_at	15	P53TG1	AB007457	7q21.1
209917_s_at	14	P53TG1	BC002709	7q21.1
208612_at	15	P58	D83485	15q15
208843_s_at	15	p59	BC001408	2q31.1-q31.2
211552_s_at	12	P5CDh	U24267	1p36
217791_s_at	15	P5CSL	U76542	10q24.3
216835_s_at	15	P62DOK; MGC117395	AF035299	2p13
215749_s_at	15	P65	AK001574	3p22-p21.33
209861_s_at	15	p67; MNPEP	U13261	12q23.1
208719_s_at	14	P72	U59321	22q13.1
216347_s_at	14	p85	AK023188	14q32.33
208649_s_at	15	p97	AF100752	9p13-p12
214794_at	15	PA2G4	BF669264	12q13
215823_x_at	15	PABP	U64661	Xq13-q21
215157_x_at	15	PABPC1	AI734929	8q22.2-q23
208113_x_at	15	PABPC3	NM_030979	13q12-q13
201064_s_at	15	PABPC4	NM_003819	1p32-p36
201544_x_at	15	PABPN1	BF675004	14q11.2-q13
201545_s_at	15	PABPN1	NM_004643	14q11.2-q13
213046_at	15	PABPN1	AI130920	14q11.2-q13
41329_at	13	PACE-1	AI458463	1q23.3
214204_at	15	PACRG	BF224076	6q26
212778_at	15	PACS1L	AL583340	14q32.33
201651_s_at	15	PACSIN2	NM_007229	22q13.2-13.33
218744_s_at	14	PACSIN3	NM_016223	11p12-p11.12
202093_s_at	15	PAF1	NM_019088	19q13.1
320_at	13	PAF-2	D83703	6p21.1
200813_s_at	11	PAFAH1B1	BE256969	17p13.3
200816_s_at	15	PAFAH1B1	NM_000430	17p13.3
210160_at	14	PAFAH1B2	BC000398	11q23
203228_at	11	PAFAH1B3	NM_002573	19q13.1
201013_s_at	15	PAICS	AA902652	4pter-q21
201014_s_at	15	PAICS	NM_006452	4pter-q21
209063_x_at	15	PAIP1	BF248165	---
210283_x_at	15	PAIP1	BC005295	5p12
213754_s_at	15	PAIP1	AW613203	5p12
217724_at	15	PAI-RBP1	BE730433	1p31-p22
217725_x_at	15	PAI-RBP1	BE730433	1p31-p22
218886_at	11	PAK1IP1	NM_017906	6p24.1
208875_s_at	14	PAK2	AF092132	3q29
208877_at	15	PAK2	W74494	3q29
208878_s_at	14	PAK2	AF092132	3q29
33814_at	15	PAK4	AF005046	19q13.2

200897_s_at	15	PALLD	NM_016081	4q32.3
200906_s_at	15	PALLD	NM_016081	4q32.3
203859_s_at	11	PALM	NM_002579	19p13.3
202759_s_at	15	PALM2-AKAP2	BE879367	9q31-q33
202760_s_at	15	PALM2-AKAP2	NM_007203	9q31-q33
218736_s_at	14	PALMD	NM_017734	1p22-p21
202336_s_at	15	PAM	NM_000919	5q14-q21
212958_x_at	15	PAM	AI022882	5q14-q21
214620_x_at	15	PAM	BF038548	5q14-q21
222203_s_at	15	PAN2	AK023625	2p24.3
218809_at	15	PANK2	NM_024960	20p13
218433_at	14	PANK3	NM_024594	5q34
221751_at	15	PANK3	AL565516	5q34
218771_at	15	PANK4	NM_018216	1p36.32
204715_at	14	PANX1	NM_015368	11q21
50400_at	10	PAOX	AI743990	10q26.3
210946_at	15	PAP2-a2	AF014403	5q11
218947_s_at	14	PAPD1	NM_018109	10p12.1
222282_at	11	PAPD4	AV761453	---
209388_at	15	PAPOLA	BC000927	14q32.31
212718_at	15	PAPOLA	BF797555	14q32.31
212720_at	14	PAPOLA	AI670847	14q32.31
222035_s_at	15	PAPOLA	AI984479	14q32.31
222273_at	14	PAPOLG	AI419423	---
203058_s_at	15	PAPSS2	AW299958	10q23-q24
203059_s_at	14	PAPSS2	NM_004670	10q23-q24
203060_s_at	15	PAPSS2	NM_004670	10q23-q24
213372_at	14	PAQR3	AW173157	4q21.23
219236_at	10	PAQR6	NM_024897	1q23.1
210434_x_at	15	PAR	AF151056	1q21
205245_at	13	PARD6A	NM_016948	16q22.1
205060_at	11	PARG	NM_003631	10q11.23
200006_at	15	PARK7	NM_007262	1p36.33-p36.12
203905_at	11	PARN	NM_002582	16p13
218543_s_at	15	PARP12	NM_022750	7q34
219034_at	13	PARP16	NM_017851	15q22.2
204752_x_at	12	PARP2	NM_005484	14q11.2-q12
215773_x_at	15	PARP2	AJ236912	14q11.2-q12
202239_at	12	PARP4	NM_006437	13q11
219639_x_at	15	PARP6	NM_020213	15q22.33
219033_at	12	PARP8	NM_024615	5q11.2
217890_s_at	14	PARVA	NM_018222	11p15.3
216253_s_at	14	PARVB	N73272	22q13.2-q13.33
37965_at	15	PARVB	AA181053	22q13.2-q13.33
37966_at	15	PARVB	AA187563	22q13.2-q13.33
222221_x_at	12	PAST	AY007161	11q13
204004_at	14	PAWR	AI336206	12q21
204005_s_at	14	PAWR	NM_002583	12q21
121_at	15	PAX8	X69699	2q12-q14
212825_at	15	PAXIP1L	AI357401	7q36

220355_s_at	15	PB1	AF177387	3p21
217738_at	15	PBEF1	NM_005746	7q22.2
217739_s_at	15	PBEF1	NM_005746	7q22.2
209584_x_at	14	PBI	AF165520	22q13.1-q13.2
219148_at	15	PBK	NM_018492	8p21.2
205353_s_at	15	PBP	NM_002567	12q24.23
211941_s_at	15	PBP	BE969671	12q24.23
205253_at	14	PBX1	NM_002585	1q23
212148_at	15	PBX1	AL049381	1q23
212151_at	14	PBX1	BF967998	1q23
202875_s_at	15	PBX2	BE397715	6p21.3
202876_s_at	14	PBX2	NM_002586	6p21.3
204082_at	15	PBX3	NM_006195	9q33-q34
214177_s_at	14	PBXIP1	AI935162	1q22
212857_x_at	15	PC4	BG231551	5p13.3
221727_at	15	PC4	AA456973	5p13.3
203845_at	15	PCAF	AV727449	3p24
203557_s_at	15	PCBD1	NM_000281	10q22
208620_at	15	PCBP1	U24223	2p13-p12
204031_s_at	15	PCBP2	NM_005016	12q12
213517_at	15	PCBP2	AW103422	12q12
209361_s_at	15	PCBP4	BC004153	3p21
203860_at	15	PCCA	NM_000282	13q32
212694_s_at	15	PCCB	NM_000532	3q21-q22
219656_at	14	PCDH12	NM_016580	5q31
218892_at	15	PCDH16	NM_024542	11p15.4
205656_at	15	PCDH17	NM_014459	13q14.3
219737_s_at	11	PCDH9	NM_020403	---
205717_x_at	15	PCDHGC3	NM_002588	5q31
209079_x_at	15	PCDHGC3	AF152318	5q31
211066_x_at	15	PCDHGC3	BC006439	5q31 /// 5q31
215836_s_at	15	PCDHGC3	AK026188	5q31
203378_at	15	PCF11	AB020631	11q13
203792_x_at	11	PCGF2	NM_007144	17q21.2
203793_x_at	15	PCGF2	NM_007144	17q21.2
213551_x_at	15	PCGF2	AI744229	---
214239_x_at	15	PCGF2	AI560455	---
204564_at	15	PCGF3	NM_006315	4p16.3
212753_at	15	PCGF3	AI692203	4p16.3
202265_at	15	PCGF4	NM_005180	10p11.23
208715_at	15	PCIA3	AB020980	1q22-q25
208716_s_at	15	PCIA3	AB020980	1q22-q25
210768_x_at	15	PCIA3	AF274935	1q22-q25
211098_x_at	14	PCIA3	AF277194	1q22-q25
210335_at	10	PCIP1	AF056209	12q21.32
202174_s_at	15	PCM1	NM_006197	8p22-p21.3
209997_x_at	10	PCM1	BC000453	8p22-p21.3
214118_x_at	15	PCM1	AI205598	8p22-p21.3
205202_at	15	PCMT1	NM_005389	6q24-q25
208857_s_at	15	PCMT1	M93008	6q24-q25

210156_s_at	15	PCMT1	D25547	6q24-q25
201202_at	15	PCNA	NM_002592	20pter-p12
217816_s_at	15	PCNP	NM_020357	3q12.3
219295_s_at	15	PCOLCE2	NM_013363	3q21-q24
201933_at	15	PCOLN3	NM_002768	16q24.3
205549_at	15	PCP4	NM_006198	21q22.2
222175_s_at	15	PCQAP	AK000003	22q11.2
205559_s_at	13	PCSK5	NM_006200	9q21.3
213652_at	10	PCSK5	AU152579	9q21.3
207414_s_at	14	PCSK6	NM_002570	15q26
208935_s_at	15	pcta-1	L78132	1q42-q43
207239_s_at	15	PCTK1	NM_006201	Xp11.3-p11.23
208823_s_at	14	PCTK1	BE787860	Xp11.3-p11.23
208824_x_at	15	PCTK1	BC001048	Xp11.3-p11.23
221918_at	15	PCTK2	AI742210	12q23.1
214797_s_at	15	PCTK3	BC000281	1q31-q32
218676_s_at	15	PCTP	NM_021213	17q21-q24
203803_at	12	PCYOX1	N45309	2p13.3
204209_at	14	PCYT1A	AI638771	---
209577_at	15	PCYT2	BC000351	17q25.3
202290_at	15	PDAP1	NM_014891	7q22.1
210907_s_at	15	PDCD10	BC002506	3q26.2
212422_at	12	PDCD11	AL547263	10q24.33
204025_s_at	11	PDCD2	NM_002598	6q27
213581_at	15	PDCD2	BF446180	6q27
202730_s_at	12	PDCD4	NM_014456	10q24
202731_at	15	PDCD4	NM_014456	10q24
212593_s_at	15	PDCD4	AI185160	10q24
212594_at	14	PDCD4	AI185160	10q24
219275_at	15	PDCD5	NM_004708	19q12-q13.1
203415_at	15	PDCD6	NM_013232	5pter-p15.2
217746_s_at	15	PDCD6IP	NM_013374	3p22.3
205512_s_at	15	PDCD8	NM_004208	Xq25-q26
219043_s_at	15	PDCL3	NM_024065	2q12.1
205501_at	15	PDE10A	AI143879	---
208396_s_at	14	PDE1A	NM_005019	2q32.1
207303_at	15	PDE1C	NM_005020	7p15.1-p14.3
208591_s_at	11	PDE3B	NM_000922	11p15.1 /// 11p15.1
214582_at	15	PDE3B	NM_000753	11p15.1 /// 11p15.1
204735_at	12	PDE4A	NM_006202	19p13.2
203708_at	15	PDE4B	NM_002600	1p31
206792_x_at	15	PDE4C	NM_000923	19p13.11
204491_at	15	PDE4D	R40917	5q12
210837_s_at	14	PDE4D	AF012074	5q12
205872_x_at	15	PDE4DIP	NM_022359	1q12
211751_at	11	PDE4DIP	BC005949	1q12 /// 1q12
212390_at	15	PDE4DIP	AB007923	1q12
213388_at	15	PDE4DIP	H15535	---
214129_at	15	PDE4DIP	AI821791	1q12
204091_at	14	PDE6D	NM_002601	2q35-q36

212521_s_at	15	PDE8A	BE568219	15q25.2
212522_at	15	PDE8A	W73272	15q25.2
219575_s_at	15	PDF	NM_022341	16q22.1
205463_s_at	11	PDGFA	NM_002607	7p22
204200_s_at	13	PDGFB	NM_002608	22q12.3-q13.1
216061_x_at	15	PDGFB	AK022920	22q12.3-q13.1
218718_at	15	PDGFC	NM_016205	4q32
203131_at	15	PDGFRA	NM_006206	4q11-q13
202273_at	10	PDGFRB	NM_002609	5q31-q32
205226_at	12	PDGFRL	NM_006207	8p22-p21.3
200980_s_at	15	PDHA1	BF739979	Xp22.2-p22.1
208911_s_at	15	PDHB	M34055	3p21.1-p14.2
211023_at	15	PDHB	AL117618	3p21.1-p14.2
203067_at	15	PDHX	NM_003477	11p13
208658_at	15	PDIA4	BC000425	7q35
211048_s_at	15	PDIA4	BC006344	7q35 /// 7q35
203857_s_at	15	PDIA5	NM_006810	3q21.1
207668_x_at	15	PDIA6	NM_005742	2p25.1
208639_x_at	15	PDIA6	BC001312	2p25.1
202590_s_at	15	PDK2	AL574319	17q21.33
221957_at	14	PDK3	BF939522	Xp22.13
205960_at	15	PDK4	NM_002612	7q21.3-q22.1
208690_s_at	15	PDLIM1	BC000915	10q22-q26.3
219165_at	15	PDLIM2	NM_021630	8p21.2
210170_at	13	PDLIM3	BC001017	4q35
211564_s_at	15	PDLIM4	BC003096	5q31.1
203242_s_at	15	PDLIM5	BG054550	4q22
203243_s_at	15	PDLIM5	NM_006457	4q22
212412_at	15	PDLIM5	AV715767	4q22
213684_s_at	15	PDLIM5	BF671400	4q22
221994_at	15	PDLIM5	AA196325	4q22
211681_s_at	11	PDLIM5; L9; ENH	AF116705	4q22 /// 4q22
203370_s_at	13	PDLIM7	NM_005451	5q35.3
214121_x_at	15	PDLIM7	AA086229	5q35.3
214266_s_at	10	PDLIM7	AW206786	5q35.3
220236_at	14	PDPR	NM_017990	16q22.1
211663_x_at	15	PDS	M61900	9q34.2-q34.3
202671_s_at	15	PDXK	NM_003681	21q22.3
218018_at	15	PDXK	AW449022	21q22.3
218019_s_at	15	PDXK	AW449022	21q22.3
212915_at	15	PDZRN3	AL569804	3p14.1
37950_at	15	PE	X74496	6q22
200787_s_at	15	PEA15	BC002426	1q21.1
200788_s_at	15	PEA15	NM_003768	1q21.1
208981_at	15	PECAM1	AA702701	17q23
218025_s_at	15	PECI	NM_006117	6p24.3
217923_at	15	PEF1	NM_012392	1p34
212092_at	13	PEG10	BE858180	7q21
212094_at	15	PEG10	BE858180	7q21
209242_at	15	PEG3	AL042588	19q13.4

209243_s_at	13	PEG3	AF208967	19q13.4
218319_at	15	PELI1	NM_020651	2p13.3
218472_s_at	15	PELO	NM_015946	5q11.2
215354_s_at	14	PELP1	BC002875	17p13.3
207621_s_at	15	PEMT	NM_007169	17p11.2
218302_at	15	PEN2	NM_018468	19q13.13
213791_at	15	PENK	NM_006211	8q23-q24 /// 8q23-q24
202108_at	15	PEPD	NM_000285	19q12-q13.2
202861_at	14	PER1	NM_002616	17p13.1-17p12
205251_at	15	PER2	NM_022817	2q37.3
221811_at	10	PERLD1	BF033007	17q21.2
55616_at	15	PERLD1	AI703342	17q21.2
202212_at	10	PES1	NM_014303	22q12.1
204300_at	14	PET112L	NM_004564	4q27-q28
204873_at	11	PEX1	NM_000466	7q21-q22
215023_s_at	12	PEX1	AC000064	7q21-q22
213296_at	15	PEX10	BF339133	1pter-q24
202658_at	15	PEX11B	NM_003846	1q21.2
205094_at	15	PEX12	NM_000286	17q21.1
203503_s_at	15	PEX14	NM_004565	1p36.22
33760_at	14	Pex14	AB017546	1p36.22
49878_at	15	PEX16	AA523441	11p11.2
201706_s_at	15	PEX19	BC000496	1q22
201707_at	13	PEX19	NM_002857	1q22
219180_s_at	12	PEX26	AI817074	22q11.21
203970_s_at	15	PEX3	NM_003630	6q23-q24
203972_s_at	15	PEX3	AB035307	6q23-q24
203244_at	15	PEX5	NM_000319	12p13.3
211033_s_at	15	PEX7	BC006268	6q21-q22.2
206390_x_at	12	PF4	NM_002619	4q12-q21
202259_s_at	15	PFAAP5	NM_014887	13q12-q13
221899_at	14	PFAAP5	AI809961	13q12-q13
202258_s_at	15	PFAAP5	U50532	13q12-q13
213302_at	10	PFAS	AL044326	17p13.1
201507_at	15	PFDN1	NM_002622	5q31
218336_at	15	PFDN2	NM_012394	1q23.1
205361_s_at	15	PFDN4	AI718295	20q13
205362_s_at	14	PFDN4	NM_002623	20q13
207132_x_at	15	PFDN5	NM_002624	12q12
202464_s_at	15	PFK2; IPFK2	D49817	10p14-p15
209992_at	13	PFKFB2	AB044805	1q31
201102_s_at	15	PFKL	NM_002626	21q22.3
211065_x_at	15	PFKL	BC006422	21q22.3 /// 21q22.3
210976_s_at	15	PFKM	U24183	12q13.3
201037_at	15	PFKP	NM_002627	10p15.3-p15.2
200634_at	15	PFN1	NM_005022	17p13.3
204992_s_at	15	PFN2	NM_002628	3q25.1-q25.2
221521_s_at	14	Pfs2	BC003186	16q24.1
204604_at	15	PFTK1	NM_012395	7q21-q22

201893_x_at	15	PG40	AF138300	12q13.2
211813_x_at	15	PG40	AF138303	12q13.2
211896_s_at	15	PG40	AF138302	12q13.2
200886_s_at	15	PGAM1	NM_002629	10q25.3
205736_at	15	PGAM2	NM_000290	7p13-p12
213469_at	13	PGAP1	AV705244	2q33.1
203501_at	14	PGCP	NM_006102	8q22.2 /// 8q22.2
208454_s_at	14	PGCP	NM_016134	8q22.2
201118_at	14	PGD	NM_002631	1p36.3-p36.13
206726_at	13	PGDS	NM_014485	4q22.3
203450_at	15	PGEA1	NM_015373	22q12
215179_x_at	15	PGF	AK023843	14q24-q31
211571_s_at	14	pgH3	D32039	5q14.3
200737_at	15	PGK1	NM_000291	Xq13
200738_s_at	15	PGK1	NM_000291	Xq13
217356_s_at	15	PGK1	S81916	Xq13
218387_s_at	15	PGLS	NM_012088	19p13.2
218388_at	15	PGLS	NM_012088	19p13.2
220944_at	15	PGLYRP4	NM_020393	1q21
201968_s_at	15	PGM1	NM_002633	1p31
221788_at	15	PGM3	AI433464	6q14.1-q15
219891_at	15	PGPEP1	NM_017712	19p13.11
201120_s_at	15	PGRMC1	NM_006667	Xq22-q24
201121_s_at	15	PGRMC1	NM_006667	Xq22-q24
201701_s_at	15	PGRMC2	NM_006320	4q26
213227_at	15	PGRMC2	BE879873	4q26
219394_at	13	PGS1	NM_024419	17q25.3
222125_s_at	15	PH-4	BC000580	3p21.31
204048_s_at	15	PHACTR2	AA551142	6q24.1
204049_s_at	15	PHACTR2	NM_014721	6q24.1
200658_s_at	15	PHB	NM_002634	17q21
200659_s_at	15	PHB	NM_002634	17q21
201600_at	15	PHB2	NM_007273	12p13
218338_at	14	PHC1	NM_004426	12p13
200919_at	15	PHC2	NM_004427	1p34.3
202928_s_at	13	PHF1	NM_024165	6p21.3
40446_at	15	PHF1	AL021366	6p21.3
219126_at	15	PHF10	NM_018288	6q27
221786_at	15	PHF10	BF431618	6q27
221787_at	15	PHF10	BF431618	6q27
221816_s_at	15	PHF11	BF055474	13q14.12
212660_at	14	PHF15	AI735639	5q31.2
218517_at	14	PHF17	NM_024900	4q26-q27
212726_at	14	PHF2	AB014562	9q22.31
206567_s_at	10	PHF20	NM_016436	20q11.22-q11.23
203278_s_at	12	PHF21A	NM_016621	11p11.2
218616_at	15	PHF22	NM_020395	4q25
215718_s_at	11	PHF3	AI949220	6q12
217951_s_at	11	PHF3	NM_015153	6q12
217952_x_at	15	PHF3	NM_015153	6q12

217954_s_at	15	PHF3	NM_015153	6q12
212916_at	14	PHF8	AW249934	Xp11.22
201397_at	15	PHGDH	NM_006623	1p12
212542_s_at	15	PHIP	BF224151	6q14
213074_at	15	PHIP	BG545769	6q14-q15
209439_s_at	14	PHK	D38616	Xp22.2-p22.1
209438_at	12	PHKA2	AL096700	Xp22.2-p22.1
202738_s_at	15	PHKB	BG149218	16q12-q13
202739_s_at	15	PHKB	NM_000293	16q12-q13
217996_at	15	PHLDA1	NM_007350	12q15
217997_at	15	PHLDA1	NM_007350	12q15
209674_at	15	PHLL1	D83702	12q23-q24.1
217226_s_at	15	PHOX1	M95929	1q24
210191_s_at	12	PHTF1	BC002447	1p13
209780_at	15	PHTF2	AL136883	7q11.23-q21
203335_at	15	PHYH	NM_006214	10pter-p11.2
211429_s_at	13	PI	AF119873	---
212688_at	15	PI3K	BC003393	3q22.3
210417_s_at	13	PI4Kb	U81802	1q21
209345_s_at	15	PI4KII	AL561930	10q24
217862_at	15	PIAS1	NM_016166	15q
217863_at	15	PIAS1	NM_016166	15q
217864_s_at	15	PIAS1	NM_016166	15q
203035_s_at	10	PIAS3	NM_006099	1q21
212881_at	15	PIASY	AK022481	19p13.3
213239_at	15	PIBF1	NM_006346	13q21.33
212506_at	15	PICALM	AL135735	11q14
212511_at	14	PICALM	AI766247	11q14
215236_s_at	10	PICALM	AV721177	11q14
215832_x_at	15	PICALM	AV722190	11q14
209080_x_at	15	PICOT	AF118652	6p25.3
214205_x_at	15	PICOT	AK022131	6p25.3
210367_s_at	15	PIG12	AF010316	9q34.3
219501_at	15	PIG38	NM_017993	13q14.11
203491_s_at	12	PIG8	AI123527	11q21
203492_x_at	15	PIG8	AA918224	11q21
205281_s_at	13	PIGA	NM_002641	Xp22.1
205452_at	15	PIGB	NM_004855	15q21-q22
214152_at	12	PIGB	AU144243	15q21.1
202846_s_at	13	PIGC	NM_002642	1q23-q25 /// 1q23-q25
216593_s_at	10	PIGCP1	AB000359	---
205077_s_at	15	PIGF	NM_002643	2p21-p16
205078_at	15	PIGF	NM_002643	2p21-p16
212117_at	15	PIGF	BF978689	2p21
212120_at	15	PIGF	BE897886	2p21-p16
212122_at	15	PIGF	AW771590	2p21
209625_at	14	PIGH	BC004100	14q11-q24
205873_at	14	PIGL	NM_004278	17p12-p11.2
219048_at	12	PIGN	NM_012327	18q21.33

209998_at	13	PIGO	BC001030	9p13.2
213070_at	15	PIK3C2A	AV682436	11p15.5-p14
204484_at	13	PIK3C2B	NM_002646	1q32
204297_at	15	PIK3C3	NM_002647	18q12.3
204369_at	15	PIK3CA	NM_006218	3q26.3
212239_at	15	PIK3R1	AI680192	5q13.1
212240_s_at	15	PIK3R1	AI679268	5q13.1
212249_at	13	PIK3R1	AI679268	5q13.1
207105_s_at	15	PIK3R2	NM_005027	19q13.2-q13.4
202743_at	15	PIK3R3	BE622627	1p34.1
212740_at	15	PIK3R4	BF740111	3q22.1
220566_at	13	PIK3R5	NM_014308	17p13.1
207081_s_at	15	PIK4CA	NM_002650	22q11.21
206138_s_at	12	PIK4CB	NM_002651	1q21
206139_at	15	PIK4CB	NM_002651	1q21
220954_s_at	15	PILRB	NM_013440	7q22.1
209193_at	15	PIM1	M24779	6p21.2
202927_at	13	PIN1	NM_006221	19p13
204571_x_at	15	PIN4	BE797213	Xq13
214224_s_at	15	PIN4	BE674061	Xq13
214225_at	13	PIN4	BE674061	Xq13
209018_s_at	15	PINK1	BF432478	1p36
209019_s_at	15	PINK1	BF432478	1p36
207391_s_at	15	PIP5K1A	NM_003557	1q22-q24
205632_s_at	15	PIP5K1B	NM_003558	9q13
212518_at	15	PIP5K1C	AB011161	19p13.3
205570_at	11	PIP5K2A	NM_005028	10p12.32
201080_at	15	PIP5K2B	BF338509	---
213111_at	15	PIP5K3	AB023198	2q34
207469_s_at	15	PIR	NM_003662	Xp22.31
201190_s_at	15	PITPNA	H15647	17p13.3
201192_s_at	15	PITPNA	NM_006224	17p13.3
202522_at	15	PITPNB	AL031591	22q12.1
219155_at	15	PITPNC1	NM_012417	17q24.3
205273_s_at	14	PITRM1	NM_014968	10p15.3
207558_s_at	10	PITX2	NM_000325	4q25-q27
218667_at	15	PJA1	NM_022368	Xq13.1
201133_s_at	15	PJA2	AA142966	5q22.1
202328_s_at	15	PKD1	NM_000296	16p13.3
203688_at	15	PKD2	NM_000297	4q21-q23
204612_at	15	PKIA	NM_006823	8q21.11
202732_at	15	PKIG	NM_007066	20q12-q13.1
201251_at	15	PKM2	NM_002654	15q22
213700_s_at	15	PKM2	AA554945	15q22
204267_x_at	15	PKMYT1	NM_004203	16p13.3
202161_at	12	PKN1	NM_002741	19p13.1-p12
212628_at	15	PKN2	BG292065	1p22.2
212629_s_at	13	PKN2	AI633689	1p22.2
54051_at	13	PKNOX1	H59033	21q22.3
63305_at	14	PKNOX2	D81792	---

207717_s_at	15	PKP2	NM_004572	12p11
201928_at	15	PKP4	AA194254	2q23-q31
201929_s_at	15	PKP4	NM_003628	2q23-q31
216267_s_at	10	PL6	BF034906	3p21.3
221027_s_at	14	PLA2G12A	NM_030821	4q25 /// 4q25
203649_s_at	13	PLA2G2A	NM_000300	1p35
219095_at	14	PLA2G4B	NM_005090	15q11.2-q21.3
60528_at	15	PLA2G4B	N71116	15q11.2-q21.3
206178_at	15	PLA2G5	NM_000929	1p36-p34
215870_s_at	15	PLA2G5	AL158172	1p36-p34
204691_x_at	14	PLA2G6	NM_003560	22q13.1
210647_x_at	15	PLA2G6	AF102988	22q13.1
206214_at	13	PLA2G7	NM_005084	6p21.2-p12
209533_s_at	14	PLA2P	AF145020	9p21
219702_at	10	PLAC1	NM_021796	Xq26
219014_at	15	PLAC8	NM_016619	4q21.3
205372_at	15	PLAG1	NM_002655	8q12
207002_s_at	15	PLAGL1	NM_002656	6q24-q25
207943_x_at	14	PLAGL1	NM_006718	6q24-q25
209318_x_at	15	PLAGL1	BG547855	6q24-q25
202924_s_at	12	PLAGL2	AF006005	20q11.21
201860_s_at	15	PLAT	NM_000930	8p12
205479_s_at	12	PLAU	NM_002658	10q24
211668_s_at	14	PLAU	K03226	10q24 /// 10q24
204938_s_at	15	PLB	M60411	6q22.1
213222_at	15	PLCB1	AL049593	20p12
203895_at	15	PLCB4	AL535113	20p12
203896_s_at	15	PLCB4	AL535113	20p12
205111_s_at	14	PLCE1	NM_016341	10q23
205112_at	13	PLCE1	NM_016341	10q23
214159_at	10	PLCE1	AW771015	10q23
216218_s_at	15	PLCE2	AK023546	3p25.1-p24.3
204613_at	15	PLCG2	NM_002661	16q24.1
205934_at	15	PLCL1	NM_006226	2q33
213309_at	15	PLCL2	AL117515	3p25.1-p24.3
218951_s_at	13	PLCXD1	NM_018390	Xp22.33
177_at	13	PLD1	U38545	3q26
201050_at	15	PLD3	NM_012268	19q13.2
201373_at	12	PLEC1	NM_000445	8q24
219024_at	14	PLEKHA1	NM_021622	10q26.13
214989_x_at	15	PLEKHA5	BC000969	12p12
220952_s_at	15	PLEKHA5	NM_019012	12p12
201410_at	15	PLEKHB2	AI983043	2q21.2
201411_s_at	13	PLEKHB2	NM_017958	2q21.2
209209_s_at	15	PLEKHC1	AW469573	14q22.1
214212_x_at	15	PLEKHC1	AI928241	14q22.1
212719_at	15	PLEKHE1	AB011178	18q21.33
218640_s_at	15	PLEKHF2	BF439250	8q22.1
218290_at	15	PLEKHJ1	NM_018049	19p13.3
218223_s_at	15	PLEKHO1	NM_016274	1q21.3

202240_at	10	PLK1	NM_005030	16p12.3
201939_at	15	PLK2	NM_006622	5q12.1-q13.2
204887_s_at	10	PLK4	NM_014264	4q27-q28
204939_s_at	15	PLN	NM_002667	6q22.1
204940_at	15	PLN	NM_002667	6q22.1
200827_at	15	PLOD1	NM_000302	1p36.3-p36.2
202619_s_at	15	PLOD2	AI754404	3q23-q24
202620_s_at	15	PLOD2	NM_000935	3q23-q24
202185_at	15	PLOD3	NM_001084	7q22
210198_s_at	15	PLP1	BC002665	Xq22
201136_at	15	PLP2	NM_002668	Xp11.23
201215_at	15	PLS3	NM_005032	Xq24
202430_s_at	15	PLSCR1	NM_021105	3q23
202446_s_at	15	PLSCR1	AI825926	3q23
56197_at	15	PLSCR3	AI783924	---
218901_at	15	PLSCR4	NM_020353	3q24
202075_s_at	15	PLTP	NM_006227	20q12-q13.1
214081_at	14	PLXDC1	AF070526	17q21.1
219700_at	15	PLXDC1	NM_020405	17q21.1
221537_at	14	PLXNA1	T16388	3q21.3
221538_s_at	11	PLXNA1	T16388	3q21.3
213030_s_at	13	PLXNA2	AI688418	1q32.2
208890_s_at	13	PLXNB2	BC004542	22q13.33
38671_at	15	PLXND1	AB014520	3q21.3
217225_x_at	15	PM5	AL512687	16p13.11
49077_at	15	PME-1	AL040538	11q13.3
202337_at	15	PMF1	NM_007221	1q12
209640_at	14	PML	M79462	15q22
203467_at	15	PMM1	NM_002676	22q13.2
203201_at	11	PMM2	NM_000303	16p13.3-p13.2
212088_at	15	PMPCA	BF570122	9q34.3
201682_at	15	PMPCB	NM_004279	7q22-q32
213677_s_at	15	PMS1	BG434893	2q31-q33
215667_x_at	15	PMS2L1	AI375694	7q22.1
214756_x_at	15	PMS2L13	AB017004	7q22.1
215412_x_at	14	PMS2L16	AB017007	7q22.1
214473_x_at	15	PMS2L3	NM_005395	7q11.23
203515_s_at	15	PMVK	NM_006556	1p13-q23
222158_s_at	15	PNAS-4	AF229834	1q44
212371_at	15	PNAS-4	AL049397	1q44
218961_s_at	15	PNKP	NM_007254	19q13.3-q13.4
218224_at	15	PNMA1	NM_006029	14q24.2
209598_at	12	PNMA2	AB020690	8p21.1
210183_x_at	15	PNN	AF112222	15q15.1
212036_s_at	15	PNN	AW152664	14q13.3
212037_at	15	PNN	BF508848	14q13.3
212705_x_at	11	PNPLA2	BF570210	11p15.5
217779_s_at	15	PNRC2	NM_017761	1p36.11
201578_at	15	PODXL	NM_005397	7q32-q33
212349_at	11	POFUT1	AL045513	20q11

218229_s_at	14	POGK	NM_017542	1q23.3
212153_at	15	POGZ	AB007930	1q21.3
204835_at	12	POLA	NM_016937	Xp22.1-p21.3
203616_at	15	POLB	NM_002690	8p11.2
201115_at	15	POLD2	NM_006230	7p13
212836_at	15	POLD3	D26018	11q14
202996_at	10	POLD4	NM_021173	11q13
217806_s_at	15	POLDIP2	NM_015584	17q11.2
212100_s_at	15	POLDIP3	Z93241	22q13.31
215357_s_at	13	POLDIP3	Z93241	22q13.31
205909_at	11	POLE2	NM_002692	14q21-q22
208828_at	15	POLE3	BC004170	9q33
203366_at	13	POLG	NM_002693	15q25
213007_at	14	POLG	W74442	15q25
205811_at	15	POLG2	NM_007215	17q
219317_at	15	POLI	NM_007195	18q21.1
220113_x_at	11	POLR1B	NM_019014	2q13
207515_s_at	15	POLR1C	NM_004875	6p21.1
218258_at	15	POLR1D	NM_015972	13q12.2
202725_at	14	POLR2A	NM_000937	17p13.1
201803_at	15	POLR2B	NM_000938	4q12
208996_s_at	15	POLR2C	BC000409	16q13-q21
203664_s_at	14	POLR2D	NM_004805	2q21
214144_at	11	POLR2D	BF432147	2q21
213887_s_at	15	POLR2E	AI554759	19p13.3
217854_s_at	15	POLR2E	BC004441	19p13.3
209511_at	15	POLR2F	BC003582	22q13.1
202306_at	15	POLR2G	NM_002696	11q13.1
209302_at	15	POLR2H	U37689	3q28
212955_s_at	15	POLR2I	AL037557	19q12
212782_x_at	15	POLR2J	BG335629	7q11.2
216242_x_at	15	POLR2J2	AW402635	7
202634_at	15	POLR2K	AL558030	8q22.3
202635_s_at	15	POLR2K	NM_005034	8q22.3
211730_s_at	15	POLR2L	BC005903	11p15 /// 11p15
219459_at	15	POLR3B	NM_018082	12q24.11
210573_s_at	15	POLR3C	BC004424	1q21.2
208361_s_at	15	POLR3D	NM_001722	8q21
218016_s_at	15	POLR3E	NM_018119	16p12.3
205218_at	12	POLR3F	NM_006466	20p11.23
206653_at	10	POLR3G	BF062139	5q14.3
218866_s_at	15	POLR3K	AF060223	16p13.3
202466_at	15	POLS	NM_006999	5p15
213893_x_at	15	POM121	AA161026	---
217944_at	15	POMGNT1	NM_017739	1p34.1
218476_at	15	POMT1	NM_007171	9q34.1
210910_s_at	15	POMZP3	BC000487	7q11.23
201876_at	15	PON2	NM_000305	7q21.3
210830_s_at	15	PON2	AF001602	7q21.3
202868_s_at	15	POP4	NM_006627	19q13.11

204839_at	15	POP5	NM_015918	12q24.31
209482_at	15	POP7	BC001430	7q22
219647_at	15	POPDC2	NM_022135	3q13.33
219926_at	15	POPDC3	NM_022361	6q21
211967_at	15	PORIMIN	BG538627	11q22.1
204353_s_at	15	POT1	BC002923	7q31.33
204354_at	15	POT1	NM_015450	7q31.33
32502_at	14	PP1665	AL041124	11q13.3
210444_at	13	PP2; Y2B; NPY1RL	U59431	5q31
213305_s_at	14	PP2A	L42375	14q32
635_s_at	12	PP2A	L42374	11q12-q13
209296_at	15	PP2CB	AF136972	2p22.1
205761_s_at	13	PP35	AW242981	7q22-q31
209278_s_at	12	PP5; TFPI-2	L27624	7q22
212541_at	13	PP591	AL562282	1q22
209927_s_at	15	pp7704	AF261137	1q22
212199_at	15	PP784	AL566962	4p16.1
217848_s_at	15	PPA1	NM_021129	10q11.1-q24
220741_s_at	15	PPA2	NM_006903	4q25
212226_s_at	15	PPAP2B	AA628586	1pter-p22.1
212230_at	15	PPAP2B	AV725664	1pter-p22.1
203497_at	15	PPARBP	NM_004774	17q12-q21.1
219195_at	15	PPARGC1A	NM_013261	4p15.1
214146_s_at	15	PPBP	R64130	4q12-q13
218341_at	15	PPCS	NM_024664	1p34.1
202065_s_at	11	PPFIA1	NM_003626	11q13.2
202066_at	15	PPFIA1	AA195259	11q13.2
210235_s_at	14	PPFIA1	U22815	11q13.2
210236_at	10	PPFIA1	U22815	11q13.2
214978_s_at	12	PPFIA4	AK023365	1q32.1
203735_x_at	15	PPFIBP1	N35896	12p12.1
214375_at	13	PPFIBP1	AI962377	12p12.1
200661_at	15	PPGB	NM_000308	20q13.1
201293_x_at	15	PPIA	NM_021130	7p13-p11.2
211378_x_at	15	PPIA	BC001224	---
211765_x_at	15	PPIA	BC005982	7p13-p11.2
211978_x_at	15	PPIA	AI708767	7p13-p11.2
217346_at	13	PPIA	AL021395	---
212661_x_at	15	PPIAL3	BE731738	21q21.1
200967_at	15	PPIB	NM_000942	15q21-q22
200968_s_at	15	PPIB	NM_000942	15q21-q22
204517_at	15	PPIC	BE962749	5q23.2
204518_s_at	15	PPIC	NM_000943	5q23.2
204185_x_at	15	PPID	NM_005038	4q31.3
204186_s_at	15	PPID	AI014573	4q31.3
202494_at	14	PPIE	NM_006112	1p32
201489_at	15	PPIF	BC005020	10q22-q23
201490_s_at	13	PPIF	NM_005729	10q22-q23
208994_s_at	15	PPIG	AI638762	2q31.1
204228_at	15	PPIH	NM_006347	1p34.1

203966_s_at	15	PPM1A	NM_021003	14q23.1
213225_at	15	PPM1B2	AJ271832	2p22.1
204566_at	15	PPM1D	NM_003620	17q23.3
203063_at	15	PPM1F	NM_014634	22q11.22
200913_at	13	PPM1G	NM_002707	2p23.3
200885_at	15	PPM1J	NM_005167	1p13.1
218273_s_at	13	PPM2C	NM_018444	8q22.1
217841_s_at	14	PPME1	NM_016147	11q13.3
208644_at	15	PPOL	M32721	1q41-q42
204788_s_at	15	PPOX	NM_000309	1q22
200846_s_at	15	PPP1CA	NM_002708	11q13
201407_s_at	15	PPP1CB	AI186712	2p23
201408_at	15	PPP1CB	W67887	2p23
201409_s_at	15	PPP1CB	W67887	2p23
200726_at	15	PPP1CC	NM_002710	12q24.1-q24.2
201703_s_at	10	PPP1R10	NM_002714	6p21.3
201500_s_at	15	PPP1R11	NM_021959	6p21.3
201602_s_at	14	PPP1R12A	NM_002480	12q15-q21
201603_at	15	PPP1R12A	AI817061	12q15-q21
201604_s_at	15	PPP1R12A	NM_002480	12q15-q21
201958_s_at	12	PPP1R12B	NM_002481	1q32.1
218849_s_at	11	PPP1R13L	NM_006663	19q13.32
202014_at	15	PPP1R15A	NM_014330	19q13.2
205478_at	15	PPP1R1A	NM_006741	12q13.13
202165_at	15	PPP1R2	BF966540	3q29
202166_s_at	15	PPP1R2	NM_006241	3q29
204284_at	15	PPP1R3C	N26005	10q23-q24
201214_s_at	15	PPP1R7	NM_002712	2q37.3
213465_s_at	15	PPP1R7	BF718769	2q37.3
207830_s_at	15	PPP1R8	NM_002713	1p35
221088_s_at	13	PPP1R9A	NM_017650	7q21.3
208652_at	15	PPP2CA	BC000400	5q23-q31
215628_x_at	15	PPP2CA	AL049285	---
201375_s_at	15	PPP2CB	NM_004156	8p12-p11.2
200695_at	15	PPP2R1A	NM_014225	19q13.41
202313_at	15	PPP2R2A	NM_002717	8p21.1
213849_s_at	14	PPP2R2B	AA974416	5q31-5q32
207749_s_at	15	PPP2R3A	NM_002718	3q22.1
209632_at	14	PPP2R3A	AI760130	3q22.1
219264_s_at	15	PPP2R3B	NM_013239	Xp22.33
206452_x_at	14	PPP2R4	NM_021131	9q34
208874_x_at	15	PPP2R4	BC002545	9q34
216105_x_at	14	PPP2R4	X86428	9q34
202187_s_at	15	PPP2R5A	NM_006243	1q32.2-q32.3
201877_s_at	15	PPP2R5C	NM_002719	14q32
214083_at	15	PPP2R5C	AW772123	14q32
202513_s_at	15	PPP2R5D	NM_006245	6p21.1
203338_at	15	PPP2R5E	NM_006246	14q23.1
202425_x_at	14	PPP3CA	NM_000944	4q21-q24
202429_s_at	15	PPP3CA	AL353950	4q21-q24

202457_s_at	15	PPP3CA	AA911231	4q21-q24
202432_at	15	PPP3CB	NM_021132	10q21-q22
207000_s_at	14	PPP3CC	NM_005605	8p21.2
32540_at	13	PPP3CC	AI762547	8p21.2
204506_at	14	PPP3R1	AL544951	2p15
204507_s_at	10	PPP3R1	NM_000945	2p15
201594_s_at	15	PPP4R1	NM_005134	18p11.22
222288_at	13	PPP4R2	AI004009	---
203529_at	15	PPP6C	NM_016294	9q34.11 /// 9q34.11
206174_s_at	15	PPP6C	NM_002721	9q34.11
200975_at	15	PPT1	NM_000310	1p32
209490_s_at	14	PPT2	AF020543	6p21.3
209826_at	15	PPT2	AF020544	6p21.3
207769_s_at	15	PQBP1	NM_005710	Xp11.23
218208_at	15	PQLC1	NM_025078	18q23
209633_at	15	PR72	AL389975	3q22.1
218997_at	15	PRAF1	NM_022490	9p13.1
203456_at	14	PRAF2	NM_007213	Xp11.23
218009_s_at	15	PRC1	NM_003981	15q26.1
208938_at	14	PRCC	BC004913	1q21.1
201494_at	15	PRCP	NM_005040	11q14
219515_at	13	PRDM10	NM_020228	11q25
203057_s_at	15	PRDM2	AV724783	1p36
205277_at	15	PRDM2	NM_012231	1p36
218329_at	14	PRDM4	NM_012406	12q23-q24.1
49485_at	15	PRDM4	W22625	12q23-q24.1
201619_at	15	PRDX3	NM_006793	10q25-q26
201923_at	15	PRDX4	NM_006406	Xp22.13
200844_s_at	15	PRDX6	BE869583	1q24.2
200845_s_at	15	PRDX6	BE869583	1q24.2
217861_s_at	15	PREB	NM_013388	2p23.3
202918_s_at	15	PREI3	AF151853	2q33.1
202919_at	15	PREI3	NM_015387	2q33.1
37022_at	11	PRELP	U41344	1q32
204117_at	11	PREP	NM_002726	6q22
221883_at	12	PREP1; pkonx1c	AA133342	21q22.3
201858_s_at	15	PRG1	J03223	10q22.1
201859_at	15	PRG1	NM_002727	10q22.1
205053_at	15	PRIM1	NM_000946	12q13
215336_at	11	PRKA11	AK002166	13q13.3
202801_at	15	PRKACA	NM_002730	19p13.1
202741_at	15	PRKACB	AA130247	1p36.1
202742_s_at	15	PRKACB	NM_002731	1p36.1
201805_at	15	PRKAG1	NM_002733	12q12-q14
218292_s_at	15	PRKAG2	AF087875	7q35-q36
200604_s_at	15	PRKAR1A	M18468	17q23-q24
200605_s_at	15	PRKAR1A	NM_002734	17q23-q24
204842_x_at	14	PRKAR2A	BC002763	3p21.3-p21.2
213052_at	14	PRKAR2A	BF246917	3p21.3-p21.2
203680_at	15	PRKAR2B	NM_002736	7q22-q31.1

213093_at	14	PRKCA	AI471375	17q22-q23.2
209685_s_at	11	PRKCB1	M13975	16p11.2
209048_s_at	15	PRKCBP1	AB032951	20q13.13
209049_s_at	15	PRKCBP1	BC001004	20q13.13
213010_at	15	PRKCDBP	AI088622	11p15.4
206099_at	12	PRKCH	NM_006255	14q22-q23
218764_at	15	PRKCH	NM_024064	14q22-q23
209678_s_at	15	PRKCI	L18964	3q26.3
213518_at	15	PRKCI	AI689429	3q26.3
200707_at	14	PRKCSH	NM_002743	19p13.1-p13.2
214080_x_at	15	PRKCSH	AI815793	19p13.1-p13.2
205880_at	15	PRKD1	NM_002742	14q11
38269_at	15	PRKD2	AL050147	19q13.2
218236_s_at	15	PRKD3	NM_005813	2p21
218378_s_at	14	PRKRIP1	NM_024653	7q22.1
39313_at	14	PRKWNK1	AB002342	12p13.3
206279_at	10	PRKY	NM_002760	Yp11.2
221443_x_at	12	PRLH	NM_015893	2q37.3
213320_at	14	PRMT3	AL551971	11p15.1
222106_at	14	PRND	AL133396	20pter-p12
201300_s_at	15	PRNP	NM_000311	20pter-p12
215707_s_at	15	PRNP	AV725328	20pter-p12
208973_at	13	PRNPIP	BC001072	1p32
212872_s_at	15	PRO0213	AK023092	6p21.1
219599_at	12	PRO1843	NM_018507	12q13.13
220943_s_at	12	PRO1853	NM_018607	2p22.3
222231_s_at	15	PRO1855	AK025328	17q21.33
201934_at	15	PRO2730	N92524	3p21.31
203650_at	15	PROCR	NM_006404	20q11.2
204304_s_at	15	PROM1	NM_006017	4p15.33
207808_s_at	15	PROS1	NM_000313	3p11-q11.2
210759_s_at	15	pros-30	M64992	11p15.1
209384_at	15	PROSC	AA176833	8p11.2
209385_s_at	15	PROSC	AA176833	8p11.2
209535_s_at	12	proto-LBC	AF127481	15q24-q25
207401_at	12	PROX1	NM_002763	1q32.2-q32.3
40465_at	14	prp28	AF026402	12q13.12
221547_at	15	PRPF18	BC000794	10p14
203103_s_at	13	PRPF19	NM_014502	11q12.2
202251_at	15	PRPF3	NM_004698	1q21.1
202408_s_at	15	PRPF31	NM_015629	19q13.42
218040_at	15	PRPF38B	NM_018061	1p13.3
220553_s_at	14	PRPF39	NM_018333	14q21.3
209161_at	14	PRPF4	AI184802	9q31-q33
209162_s_at	12	PRPF4	AI184802	9q31-q33
202126_at	15	PRPF4B	AA156948	6p25.1
202127_at	15	PRPF4B	AB011108	6p25.1
200000_s_at	15	PRPF8	NM_006445	17p13.3
208447_s_at	15	PRPS1	NM_002764	Xq21-q27
209440_at	15	PRPS1	BC001605	Xq21-q27

203401_at	14	PRPS2	NM_002765	Xp22.3-p22.2
202529_at	15	PRPSAP1	NM_002766	17q24-q25
203537_at	15	PRPSAP2	NM_002767	17p11.2-p12
219392_x_at	13	PRR11	NM_018304	17q23.2
205618_at	15	PRRG1	NM_000950	Xp21.1
205991_s_at	15	PRRX1	NM_006902	1q24
201185_at	15	PRSS11	NM_002775	10q26.3
209017_s_at	15	PRSS15	U02389	19p13.2
202458_at	13	PRSS23	NM_007173	11q14.1
203089_s_at	15	PRSS25	NM_013247	2p12
217269_s_at	11	PRSS7	AP001672	---
209599_s_at	11	PRUNE	AI762105	1q21
201455_s_at	15	PSA; MP100	AJ132583	17q21
200866_s_at	15	PSAP	M32221	10q21-q22
200871_s_at	15	PSAP	NM_002778	10q21-q22
221619_s_at	15	PSAP; CGI-64	AF189289	6pter-p24.1
218271_s_at	15	PSARL	NM_018622	3q27.3
202880_s_at	14	PSCD1	NM_004762	17q25
209158_s_at	15	PSCD2	BC004361	19q13.3
219183_s_at	11	PSCD4	NM_013385	22q12.3-q13.1
203354_s_at	12	PSD3	NM_015310	8pter-p23.3
203355_s_at	15	PSD3	NM_015310	8pter-p23.3
218613_at	15	PSD3	NM_018422	8p22
203460_s_at	15	PSEN1	NM_007318	14q24.3
217368_at	14	pseudog 1 for mitoch ATP synthase c subunit (P2)	X69909	---
206102_at	15	PSF1	NM_021067	20p11.21
205961_s_at	15	PSIP1	NM_004682	9p22.2 /// 9p22.2
222286_at	10	PSIP1	R43279	9p22.2
201676_x_at	15	PSMA1	NM_002786	11p15.1
211746_x_at	15	PSMA1	BC005932	11p15.1 /// 11p15.1
201316_at	15	PSMA2	AL523904	7p13
201317_s_at	15	PSMA2	NM_002787	7p13
201532_at	15	PSMA3	NM_002788	14q23
203396_at	15	PSMA4	NM_002789	15q24.1
201274_at	15	PSMA5	NM_002790	1p13
208805_at	15	PSMA6	BC002979	14q13
201114_x_at	15	PSMA7	NM_002792	20q13.33
216088_s_at	15	PSMA7	AL078633	20q13.33
200876_s_at	15	PSMB1	NM_002793	6q27
214288_s_at	15	PSMB1	W86293	6q27
202659_at	15	PSMB10	NM_002801	16q22.1
200039_s_at	15	PSMB2	BC000268	1p34.2
201400_at	15	PSMB3	NM_002795	17q12
202243_s_at	15	PSMB4	NM_002796	1q21
202244_at	15	PSMB4	NM_002796	1q21
208799_at	15	PSMB5	BC004146	14q11.2 /// 14q11.2
208827_at	15	PSMB6	BC000835	17p13
200786_at	15	PSMB7	NM_002799	9q34.11-q34.12
204279_at	15	PSMB9	NM_002800	6p21.3

204219_s_at	15	PSMC1	NM_002802	14q32.11
201067_at	10	PSMC2	BF215487	7q22.1-q22.3
201068_s_at	15	PSMC2	NM_002803	7q22.1-q22.3
201267_s_at	14	PSMC3	AL545523	11p12-p13
201699_at	15	PSMC6	NM_002806	14q22.1
201198_s_at	15	PSMD1	AI860431	2q37.1
201199_s_at	15	PSMD1	NM_002807	2q37.1
219485_s_at	15	PSMD10	NM_002814	Xq22.3
208776_at	11	PSMD11	BF432873	17q12
208777_s_at	15	PSMD11	BF432873	17q12
202352_s_at	15	PSMD12	AI446530	17q24.3
202353_s_at	15	PSMD12	NM_002816	17q24.3
201232_s_at	15	PSMD13	NM_002817	11p15.5
201233_at	15	PSMD13	NM_002817	11p15.5
212296_at	15	PSMD14	NM_005805	2q24.3
212509_s_at	15	PSMD14	BF968134	---
200830_at	15	PSMD2	NM_002808	3q27.3
201388_at	15	PSMD3	NM_002809	17q21.2
200882_s_at	15	PSMD4	NM_002810	1q21.3
210460_s_at	15	PSMD4	AB033605	1q21.3
211609_x_at	15	PSMD4	U51007	---
203447_at	15	PSMD5	AU157008	9q34.11
202753_at	15	PSMD6	NM_014814	3p21.1
201705_at	15	PSMD7	NM_002811	16q23-q24
200820_at	15	PSMD8	NM_002812	19q13.13
207805_s_at	15	PSMD9	NM_002813	12q24.31-q24.32
200814_at	15	PSME1	NM_006263	14q11.2
201762_s_at	15	PSME2	NM_002818	14q11.2
200987_x_at	13	PSME3	AA758755	17q21
209852_x_at	14	PSME3	BC001423	17q21
209853_s_at	14	PSME3	BC001423	17q21
212219_at	14	PSME4	D38521	2p16.3
212222_at	15	PSME4	AU143855	2p16.3
201052_s_at	15	PSMF1	BG029917	20p13
201053_s_at	15	PSMF1	NM_006814	20p13
211961_s_at	15	PSN	AK000826	3q21.3
218371_s_at	15	PSPC1	AA969958	13q12.11
205048_s_at	10	PSPH	NM_003832	---
205194_at	15	PSPH	NM_004577	7p15.2-p15.1
212722_s_at	15	PSR	AK021780	17q25
212723_at	15	PSR	AK021780	17q25
216306_x_at	15	PTB-1	X62006	19p13.3
202189_x_at	15	PTBP1	NM_002819	19p13.3
211270_x_at	15	PTBP1	BC002397	19p13.3
211271_x_at	15	PTBP1	BC004383	19p13.3
212015_x_at	15	PTBP1	BF690062	19p13.3
212016_s_at	14	PTBP1	AA679988	19p13.3
218683_at	15	PTBP2	NM_021190	1p22.1-p21.3
214937_x_at	15	PTC4	AI924817	8p22-p21.3
210266_s_at	13	PTC7	AF220137	1p13.1

209815_at	14	PTCH	BG054916	---
215492_x_at	13	PTCRA	AL035587	6p21.3
219293_s_at	15	PTD004	NM_013341	2q31.1
217780_at	15	PTD008	NM_016145	19p13.2
221599_at	15	PTD015	BC002752	11q13.4
221600_s_at	15	PTD015	BC002752	11q13.4
215233_at	14	PTDSR	AA351360	17q25
201433_s_at	15	PTDSS1	NM_014754	8q22
221005_s_at	10	PTDSS2	NM_030783	11p15 /// 11p15
204054_at	14	PTEN	NM_000314	10q23.3
211711_s_at	15	PTEN	BC005821	10q23.3 /// 10q23.3
218967_s_at	14	PTER	BF112019	10p12
211748_x_at	15	PTGDS	BC005939	9q34.2-q34.3
212187_x_at	15	PTGDS	NM_000954	9q34.2-q34.3
213933_at	15	PTGER3	AW242315	---
204897_at	15	PTGER4	AA897516	5p13.1
218083_at	15	PTGES2	NM_025072	9q34.13
200627_at	15	PTGES3	BC003005	12q13.13
208131_s_at	15	PTGIS	NM_000961	20q13.11-q13.13
205128_x_at	14	PTGS1	NM_000962	9q32-q33.3
37547_at	15	PTHB1	U85995	7p14
37549_g_at	15	PTHB1	U87408	7p14
205911_at	12	PTHR1	NM_000316	3p22-p21.1
207821_s_at	15	PTK2	NM_005607	8q24-qter
208820_at	15	PTK2	AL037339	8q24-qter
207011_s_at	14	PTK7	NM_002821	6p21.1-p12.2
201745_at	15	PTK9	NM_002822	12q12 /// 12q12
214007_s_at	14	PTK9	AW665024	12q12
202009_at	15	PTK9L	NM_007284	3p21.1 /// 3p21.1
200772_x_at	15	PTMA	NM_002823	2q35-q36
200773_x_at	15	PTMA	NM_002823	2q35-q36
216384_x_at	15	PTMA	AF257099	---
216515_x_at	15	PTMA	AL121585	---
208549_x_at	15	PTMAP7	NM_016171	2q35-q36 /// 2q35-q36
218045_x_at	10	PTMS	M24398	12p13
209465_x_at	15	PTN	AL565812	7q33-q34
211737_x_at	15	PTN	BC005916	7q33-q34 /// 7q33-q34
212032_s_at	15	PTOV1	AL046054	19q13.33
200730_s_at	15	PTP4A1	BF576710	6q12
200731_s_at	15	PTP4A1	AW165960	6q12
200732_s_at	14	PTP4A1	AL578310	6q12
200733_s_at	15	PTP4A1	BF576710	6q12
208616_s_at	15	PTP4A2	U48297	1p35
206574_s_at	15	PTP4A3	NM_007079	8q24.3
209695_at	15	PTP4A3	BC003105	8q24.3
40524_at	12	PTPD1	X79510	14q31.3
216988_s_at	15	ptp-IV1r	L48722	---
219654_at	15	PTPLA	NM_014241	10p14-p13

212640_at	15	PTPLB	AV712602	3q21.1
202716_at	15	PTPN1	NM_002827	20q13.1-q13.2
209896_s_at	15	PTPN11	AF119855	12q24
202006_at	15	PTPN12	NM_002835	7q11.23
204201_s_at	13	PTPN13	NM_006264	4q21.3
205503_at	13	PTPN14	NM_005401	1q32.2
213521_at	13	PTPN18	AW575379	2q21.2
213136_at	15	PTPN2	AI828880	18p11.3-p11.2
213137_s_at	12	PTPN2	AI828880	18p11.3-p11.2
206687_s_at	14	PTPN6	NM_002831	12p13
202958_at	14	PTPN9	NM_002833	15q23
213795_s_at	15	PTPRA	AL121905	20p13
205846_at	14	PTPRB	NM_002837	12q15-q21
213362_at	14	PTPRD	N73931	9p23-p24.3
214043_at	15	PTPRD	BF062299	9p23-p24.3
221840_at	14	PTPRE	AA775177	10q26
204944_at	15	PTPRG	NM_002841	3p21-p14
203038_at	15	PTPRK	NM_002844	6q22.2-23.1
203329_at	15	PTPRM	NM_002845	18p11.2
210675_s_at	12	PTPRQ	U77917	12q15
204469_at	15	PTPRZ1	NM_002851	7q31.3
209694_at	15	PTS	M97655	11q22.3-q23.3
203554_x_at	15	PTTG1	NM_004219	5q35.1
200677_at	15	PTTG1IP	NM_004339	21q22.3
218135_at	15	PTX1	NM_016570	12p11.23
206157_at	14	PTX3	NM_002852	3q25
201164_s_at	15	PUM1	BG474429	1p35.2
201165_s_at	15	PUM1	BE670915	1p35.2
201166_s_at	15	PUM1	NM_014676	1p35.2
201493_s_at	15	PUM2	BE778078	2p22-p21
216221_s_at	15	PUM2	D87078	2p22-p21
206308_at	13	PuMet; M.HsaIIP	AJ223333	10p15.1
204020_at	15	PURA	BF739943	5q31
208758_at	15	PURH	D89976	2q35
221277_s_at	12	PUS3	NM_031307	11q24.2 /// 11q24.2
203149_at	14	PVRL2	NM_002856	19q13.2-q13.4
213325_at	14	PVRL3	AA129716	3q13
221974_at	15	PWCR1	AW770748	---
201606_s_at	15	PWP1	NM_007062	12q24.11
201608_s_at	15	PWP1	NM_007062	12q24.11
219076_s_at	15	PXMP2	NM_018663	12q24.33
210296_s_at	15	PXMP3	BC005375	8q21.1
219428_s_at	15	PXMP4	NM_007238	20q11.22
201087_at	15	PXN	NM_002859	12q24.31
202148_s_at	14	PYCR1	NM_006907	17q25.3
201481_s_at	15	PYGB	NM_002862	20p11.2-p11.1
202990_at	15	PYGL	NM_002863	14q21-q22
205577_at	12	PYGM	NM_005609	11q12-q13.2
217846_at	15	QARS	NM_005051	3p21.3-p21.1
209123_at	15	QDPR	BC000576	4p15.31

211874_s_at	11	MORF	AF119230	10q22.2
212452_x_at	15	MORF	AF113514	10q22.2
212262_at	15	QKI	AA149639	6q26-27
212263_at	15	QKI	AI114716	6q26-27
212265_at	15	QKI	AL031781	6q26-27
212636_at	15	QKI	AL031781	6q26-27
205174_s_at	14	QPCT	NM_012413	2p22.3
204044_at	15	QPRT	NM_014298	16p12.1
209174_s_at	15	QRICH1	BC000978	3p21.31
218949_s_at	15	QRSL1	AL136679	6q21
201482_at	14	QSCN6	NM_002826	1q24
219178_at	12	QTRTD1	NM_024638	3q13.31
214543_x_at	10	QUAKING	AF142421	6q26-27
202754_at	15	R3HDM1	NM_015361	2q21.3
200863_s_at	15	RAB11A	AI215102	15q21.3-q22.31
200864_s_at	15	RAB11A	NM_004663	15q21.3-q22.31
217793_at	15	RAB11B	NM_004218	19p13.3
219681_s_at	12	RAB11FIP1	NM_025151	8p11.22
203883_s_at	15	RAB11FIP2	BG249608	10q26.12
203884_s_at	10	RAB11FIP2	NM_014904	10q26.12
203933_at	14	RAB11FIP3	NM_014700	16p13.3
202252_at	15	RAB13	NM_002870	1q21.2
200927_s_at	15	RAB14	AA919115	9q32-q34.11
221810_at	10	RAB15	AA631242	---
59697_at	15	RAB15	AA582932	---
207791_s_at	15	RAB1A	NM_004161	2p14
208724_s_at	15	RAB1A	BC000905	2p14
212677_s_at	15	RAB1A	BG530481	2p14
213440_at	15	RAB1A	AL530264	2p14
220964_s_at	13	RAB1B	NM_030981	11q12 /// 11q12
208730_x_at	15	RAB2	AA535244	8q12.1
208731_at	15	RAB2	AU158062	8q12.1
208732_at	15	RAB2	AU158062	8q12.1
208733_at	13	RAB2	AW301641	8q12.1
208734_x_at	15	RAB2	M28213	8q12.1
221960_s_at	10	RAB2	AI189609	8q12.1
219622_at	15	RAB20	NM_017817	13q34
203885_at	15	RAB21	NM_014999	12q15
213405_at	14	RAB22A	N95443	20q13.32
218360_at	15	RAB22A	NM_020673	20q13.32
217762_s_at	15	Rab22B	AF183421	18p11.3
217763_s_at	15	Rab22B	AF183421	18p11.3
217764_s_at	15	Rab22B	AF183421	18p11.3
209514_s_at	15	RAB27A	BE502030	15q15-q21.1
222294_s_at	13	RAB27A	AW971415	15q15-q21.1
207495_at	10	RAB28	NM_004249	4p16.1
209110_s_at	15	RAB2L	AL050259	6p21.3
204214_s_at	11	RAB32	NM_006834	6q24.2
206039_at	11	RAB33A	NM_004794	Xq26.1
221014_s_at	14	RAB33B	NM_031296	4q28 /// 4q28

221819_at	15	RAB35	BF791960	12q24.31
213530_at	15	RAB3GAP	AI040009	2q21.3
213531_s_at	15	RAB3GAP	AI040009	2q21.3
202372_at	15	RAB3-GAP150	BF240652	1q42.11
204547_at	15	RAB40B	NM_006822	17q25.3
203581_at	15	RAB4A	BC002438	1q42-q43
203582_s_at	15	RAB4A	NM_004578	1q42-q43
206113_s_at	15	RAB5A	NM_004162	3p24-p22
209089_at	15	RAB5A	BC001267	3p24-p22
201276_at	15	RAB5B	AF267863	12q13
201140_s_at	15	RAB5C	AF141304	17q21.2
201156_s_at	11	RAB5C	AF141304	17q21.2
214552_s_at	13	RAB5EP; RABPT5	AF098638	17p13.3
201047_x_at	15	RAB6A	BC003617	11q13.3
221792_at	14	RAB6B	AW118072	---
210406_s_at	15	RAB6C	AL136727	2q31
212561_at	15	RAB6IP1	AA349595	11p15.3
211960_s_at	15	RAB7	BG261416	3q21.3
218699_at	15	RAB7L1	NM_003929	1q32
218700_s_at	14	RAB7L1	NM_003929	1q32
208819_at	15	RAB8A	BC002977	19p13.1
221808_at	15	RAB9A	NM_004251	Xp22.2
203136_at	15	RABAC1	NM_006423	19q13.31
74694_s_at	14	RABEP2	AA907940	16p12.1
77508_r_at	14	RABEP2	AW001436	16p12.1
203150_at	15	RABEPK	NM_005833	9q34.11
204028_s_at	15	RABGAP1	NM_012197	9q34.11
213313_at	15	RABGAP1	AI922519	9q34.11
203020_at	15	RABGAP1L	NM_014857	1q24
213982_s_at	15	RABGAP1L	BG107203	1q24
218310_at	15	RABGEF1	NM_014504	7q11.21
203573_s_at	15	RABGGTA	NM_004581	14q11.2
213704_at	15	RABGGTB	AA129753	1p31
220500_s_at	14	RABL2A	NM_007082	2q13
219151_s_at	15	RABL2B	NM_007081	22q13.33
205037_at	15	RABL4	NM_006860	22q13.1
208640_at	15	RAC1	BG292367	7p22
208641_s_at	15	RAC1	BC004247	7p22
213603_s_at	15	RAC2	BE138888	22q13.1
209426_s_at	10	RACE	AF047020	5p13.2-q11.1
222077_s_at	15	RACGAP1	AU153848	12q13.12
204460_s_at	15	RAD1	NM_002853	5p13.2
204461_x_at	15	RAD1	NM_002853	5p13.2
207405_s_at	15	RAD17	NM_002873	5q13
210826_x_at	14	RAD17	AF098533	5q13
211228_s_at	15	RAD17	AF085736	5q13
200607_s_at	15	RAD21	BG289967	8q24
200608_s_at	15	RAD21	NM_006265	8q24
201039_s_at	15	RAD23A	NM_005053	19p13.2
201046_s_at	15	RAD23A	NM_005053	19p13.2

201222_s_at	15	RAD23B	AL527365	9q31.2
201223_s_at	15	RAD23B	NM_002874	9q31.2
208393_s_at	12	RAD50	NM_005732	5q31
204146_at	14	RAD51AP1	BE966146	12p13.2-p13.1
206066_s_at	14	RAD51C	NM_002876	17q22-q23
209849_s_at	15	RAD51C	AF029669	17q22-q23
37793_r_at	13	RAD51D	AF034956	17q11
211994_at	15	RAD52	AI742553	12p13.3
208860_s_at	14	RAD54L	U09820	Xq13.1-q21.1
204828_at	14	RAD9A	NM_004584	11q13.1-q13.2
201558_at	15	RAE1	NM_003610	20q13.31
211318_s_at	15	RAE1	U85943	---
201244_s_at	15	RAF1	NM_002880	3p25
212646_at	15	RAFTLIN	D42043	3p25.1
219125_s_at	12	RAG1AP1	NM_018845	1q22
205130_at	15	RAGE	NM_014226	14q32
202052_s_at	15	RAI14	NM_015577	5p13.3-p13.2
218337_at	12	RAI16	NM_022749	8p21.3
212124_at	15	RAI17	AF070622	10q23.1
219440_at	12	RAI2	NM_021785	Xp22
209833_at	15	RAIDD	U79115	12q21.33-q23.1
214435_x_at	15	RALA	NM_005402	7p15-p13
202100_at	15	RALB	BG169673	2cen-q13
202101_s_at	11	RALB	NM_002881	2cen-q13
202844_s_at	13	RALBP1	NM_006788	18p11.3
202845_s_at	15	RALBP1	NM_006788	18p11.3
207016_s_at	15	RALDH2	AB015228	15q21.2
209050_s_at	15	RALGDS	AI421559	9q34.3
210552_s_at	11	RALGEF2	AF221098	9q34.13
204199_at	15	RALGPS1	NM_014636	9q34.13
201271_s_at	15	RALY	NM_016732	20q11.21-q11.23
218585_s_at	15	RAMP; L2DTL	AK001261	---
204916_at	15	RAMP1	NM_005855	2q36-q37.1
205779_at	15	RAMP2	NM_005854	17q12-q21.1
205326_at	14	RAMP3	NM_005856	7p13-p12
200749_at	15	RAN	BF112006	6p21
202483_s_at	15	RANBP1	NM_002882	22q11.21
53987_at	15	RANBP10	AL041852	16q22.1
201711_x_at	15	RANBP2	AI681120	2q12.3
201712_s_at	15	RANBP2	NM_006267	2q12.3
212842_x_at	15	RANBP2L1	AL043571	2q13
202640_s_at	13	RANBP3	NM_003624	19p13.3
211953_s_at	15	RANBP5	BC000947	13q32.2
211954_s_at	15	RANBP5	BC000947	13q32.2
211955_at	15	RANBP5	NM_002271	13q32.2
213019_at	15	RANBP6	AI123233	9p24.1
202583_s_at	15	RANBP9	NM_005493	6p23
213838_at	15	RANBP9	AA191426	6p23
202582_s_at	15	RANBPM	AF306510	6p23
218526_s_at	15	RANGNRF	NM_014185	17p13

209284_s_at	14	RAP140	AI922509	3p21.31
209285_s_at	15	RAP140	N38985	3p21.31
202362_at	15	RAP1A	NM_002884	1p13.3
200833_s_at	15	RAP1B	NM_015646	12q14
213280_at	11	RAP1GA3	AK000478	17p13.3
209444_at	15	RAP1GDS1	BC001851	4q23-q25
221830_at	15	RAP2A	AI302106	13q34
213923_at	15	RAP2B	AW005535	3q25.2
218668_s_at	13	RAP2C	NM_021183	Xq25
218669_at	15	RAP2C	NM_021183	Xq25
220746_s_at	15	RAP80	NM_016290	5q35.3
203096_s_at	15	RAPGEF2	BF439282	4q32.1
203097_s_at	15	RAPGEF2	NM_014247	4q32.1
205651_x_at	13	RAPGEF4	NM_007023	2q31-q32
204680_s_at	12	RAPGEF5	AI263837	7p15.3
204681_s_at	15	RAPGEF5	NM_012294	7p15.3
203749_s_at	15	RARA	AI806984	17q21
205080_at	15	RARB	NM_000965	3p24
209496_at	10	RARRES2	BC000069	7q36.1
204070_at	13	RARRES3	NM_004585	11q23
201330_at	14	RARS	NM_002887	5q35.1 /// 5q35.1
202677_at	15	RASA1	NM_002890	5q13.3
212706_at	14	RASA4	AI738591	7q11.22
205590_at	10	RASGRP1	NM_005739	15q15
214369_s_at	15	RASGRP2	AI688812	11q13
205801_s_at	15	RASGRP3	NM_015376	2p25.1-p24.1
220027_s_at	15	RASIP1	NM_017805	19q13.33
219142_at	14	RASL11B	NM_023940	4q12
219167_at	15	RASL12	NM_016563	15q11.2-q22.33
204346_s_at	15	RASSF1	NM_007182	3p21.3
203185_at	15	RASSF2	NM_014737	20pter-p12.1
49306_at	14	RASSF4	AI890191	10q11.21
209139_s_at	15	RAX	AF083033	2q31.3
203132_at	15	RB1	NM_000321	13q14.2
202033_s_at	15	RB1CC1	BG402105	8p22-q21.13
202034_x_at	15	RB1CC1	NM_014781	8p22-q21.13
211950_at	15	RBAF600	AB007931	1p36.13
219214_s_at	10	RBAK	NM_021163	7p22.2
211202_s_at	15	RBBP2H1	AF087481	1q32.1
210371_s_at	15	RBBP4	BC003092	1p34.3
217301_x_at	15	RBBP4	X71810	1p34.3
205178_s_at	15	RBBP6	NM_006910	16p12.2
201092_at	15	RBBP7	NM_002893	Xp22.22
203344_s_at	15	RBBP8	NM_002894	18q11.2
57540_at	13	RBKS	AI823980	2p23.3
212332_at	10	RBL2	BF110947	16q12.2
208984_x_at	15	RBM10	BC004181	Xp11.23
215089_s_at	15	RBM10	AW409974	Xp11.23
217221_x_at	13	RBM10	AL137421	Xp11.23
212168_at	15	RBM12	AL514547	20q11.23

211686_s_at	15	RBM13	AF251062	8p12 /// 8p12
219286_s_at	15	RBM15	NM_022768	1p13
202689_at	10	RBM15B	NM_013286	3p21.31
203250_at	15	RBM16	NM_014892	6q25.1-q25.3
218134_s_at	15	RBM22	NM_018047	5q33.1
219816_s_at	15	RBM23	NM_018107	14q11.2
212027_at	14	RBM25	AI925305	14q24.3
212030_at	15	RBM25	BG251218	14q24.3
212031_at	15	RBM25	AV757384	14q24.3
212033_at	15	RBM25	BF055107	14q24.3
218593_at	15	RBM28	NM_018077	7q32.2
208319_s_at	15	RBM3	NM_006743	Xp11.2
209497_s_at	15	RBM30	BC003503	11q13
200997_at	15	RBM4	NM_002896	11q13
201967_at	15	RBM6	NM_005777	3p21.3
218379_at	15	RBM7	NM_016090	11q23.1-q23.2
214113_s_at	15	RBM8A	AI738479	1q12
212104_s_at	15	RBM9	N95026	22q13.1
216215_s_at	15	RBM9	AL049748	22q13.1
203748_x_at	15	RBMS1	NM_016839	2q24.3
207266_x_at	15	RBMS1	NM_016837	2q24.3
215127_s_at	15	RBMS1	AL517946	2q24.3
206767_at	13	RBMS3	NM_014483	3p24-p23
213762_x_at	15	RBMX	AI452524	Xq26
204098_at	15	RBMX2	NM_016024	Xq26.1
203423_at	15	RBP1	NM_002899	3q23
2028_s_at	14	RBP3	M96577	20q11.2
207836_s_at	15	RBPMS	NM_006867	8p12-p11
209487_at	15	RBP-MS	D84109	8p12-p11
209488_s_at	15	RBP-MS	D84109	8p12-p11
207785_s_at	15	RBPSUH	NM_015874	4p15.2
211974_x_at	15	RBPSUH	AL513759	4p15.2
218117_at	15	RBX1	NM_014248	22q13.2
210751_s_at	15	RC; SMP30	D31815	Xp11.3
212820_at	15	RC3	AB020663	15q15.3
203941_at	15	RC74	NM_018250	8p21.1
218352_at	15	RCBTB1	NM_018191	13q14
204759_at	15	RCBTB2	NM_001268	13q14.3
206499_s_at	15	RCC1	NM_001269	1p36.1
202496_at	11	RCD-8	NM_014329	16q22.1
212749_s_at	15	RCHY1	AI096477	4q21.21
214281_s_at	15	RCHY1	AA524525	4q21.21
39817_s_at	15	Rcl	AF040105	6p21.1
201063_at	15	RCN1	NM_002901	11p13
201485_s_at	15	RCN2	BC004892	15q23
201486_at	15	RCN2	NM_002902	15q23
212612_at	15	RCOR	D31888	14q32.33
218344_s_at	15	RCOR3	AI807073	1q32.3
203898_at	12	RCP9	AU154853	7q11.21
203899_s_at	15	RCP9	NM_014478	7q11.21

209219_at	15	RD	L03411	6p21.3
216220_s_at	10	RDC7	X68485	1q32.1
217042_at	15	RDH11	AL096716	14q24.1
209605_at	14	RDS; MGC19578	D87292	22q13.1
212167_s_at	15	RDT	AK021419	22q11.23
204969_s_at	12	RDX	NM_002906	11q23
212397_at	15	RDX	AL137751	11q23
212398_at	15	RDX	AI057093	11q23
210216_x_at	15	REC1	AF084513	5p13.2
221531_at	15	REC14	AF309553	15q24.1
221532_s_at	15	REC14	AF309553	15q24.1
209571_at	14	recepin	U03644	2q31.1
205407_at	14	RECK	NM_021111	9p13-p12
205091_x_at	15	RECQL	NM_002907	12p12
210568_s_at	13	RECQL	BC001052	12p12
212917_x_at	15	RECQL	AI814728	12p12
213878_at	15	RECQL	AI685944	12p12
201783_s_at	15	RELA	NM_021975	11q13
205923_at	14	RELN	NM_005045	7q22
211168_s_at	11	RENT1	D86988	19p13.2-p13.11
219041_s_at	15	REPIN1	NM_014374	7q36.1
202296_s_at	15	RER1	NM_007033	1pter-q24
202297_s_at	15	RER1	AF157324	1pter-q24
200940_s_at	15	RERE	AI920976	1p36.1-p36.2
212920_at	14	REST	AV682285	---
210788_s_at	15	retDSR4; retSDR4	AF126782	14q23.1
218124_at	15	RetSat	NM_017750	2p11.2
218428_s_at	15	REV1L	NM_016316	2q11.1-q11.2
208070_s_at	15	REV3L	NM_002912	6q21
218194_at	15	REXO2	NM_015523	11q23.1-q23.2
208021_s_at	14	RFC1	NM_002913	4p14-p13 /// 4p14-p13
209084_s_at	15	RFC1	BE504689	4p14-p13
209085_x_at	13	RFC1	L14922	4p14-p13
204127_at	15	RFC3	BC000149	13q12.3-q13
204023_at	15	RFC4	NM_002916	3q27
203209_at	15	RFC5	BC001866	12q24.2-q24.3
203224_at	15	RFK	BF340123	9q21.31
203225_s_at	15	RFK	NM_018339	9q21.31
212968_at	15	RFNG	BF940276	17q25
212116_at	15	RFP	AL523814	6p22
212118_at	15	RFP	AL523814	6p22
203659_s_at	15	RFP2	NM_005798	13q14
207936_x_at	14	RFPL3	NM_006604	22q12.3
202963_at	14	RFX5	AW027312	1q21
202758_s_at	14	RFXANK	NM_003721	19p12
218723_s_at	15	RGC32	NM_014059	13q13.3
209568_s_at	15	RGL	AF186779	1q25.2
210676_x_at	15	RGP5	U64675	2q13
204316_at	10	RGS10	W19676	10q25

204319_s_at	15	RGS10	NM_002925	10q25
205823_at	14	RGS12	AI824113	4p16.3
204336_s_at	12	RGS19	NM_005873	20q13.3
202388_at	15	RGS2	NM_002923	1q31
203823_at	15	RGS3	NM_021106	9q32
204337_at	13	RGS4	AL514445	1q23.2
209070_s_at	15	RGS5	AI183997	1q23.1
209071_s_at	15	RGS5	AF159570	1q23.1
218353_at	15	RGS5	NM_025226	1q23.1 /// 1q23.1
206145_at	12	RH2	AF178841	6p21.1-p11
209709_s_at	15	RHAMM	U29343	5q33.2-qter
218686_s_at	15	RHBDF1	NM_022450	16p13.3
219489_s_at	15	RHBDL2	NM_017821	1p34.2
201453_x_at	15	RHEB	NM_005614	7q36
213404_s_at	15	RHEB	BF033683	---
213409_s_at	11	RHEB	BF593727	7q36
221780_s_at	13	RHLP	AF336851	20q13.13
215693_x_at	15	RHLP	AL512707	20q13.13
200059_s_at	15	RHOA	BC001360	3p21.3
212099_at	15	RHOB	AI263909	2pter-p12
212651_at	15	RHOBTB1	AB018283	10q21.3
202975_s_at	15	RHOBTB3	N21138	5q15
202976_s_at	15	RHOBTB3	NM_014899	5q15
216048_s_at	14	RHOBTB3	AK023621	5q15
216049_at	12	RHOBTB3	AK023621	5q15
209885_at	10	RHOD	BC001338	11q14.3
219045_at	15	RHOF	NM_019034	12q24.31
203175_at	15	RHOG	NM_001665	11p15.5-p15.4
212119_at	15	RHOQ	BF670447	2p21
214449_s_at	15	RHOQ	NM_012249	2p21
218323_at	15	RHOT1	NM_018307	17q12
222148_s_at	15	RHOT1	BF688108	17q12
65770_at	14	RHOT2	AI186666	16p13.3
49327_at	15	RIC8	AI492888	11p15.5
221647_s_at	15	RIC-8	AL136935	11p15.5
219446_at	15	RIC8B	NM_018157	12q24.11
208632_at	15	RIE2	AL578551	12q24.31
221127_s_at	14	RIG	NM_006394	11p15.1
36829_at	15	RIGUI	AF022991	17p13.1-17p12
205211_s_at	12	RIN1	NM_004292	11q13.1
209684_at	15	RIN2	AL136924	---
60471_at	12	RIN3	AA625133	14q32.13
208371_s_at	12	RING1	NM_002931	6p21.3
35685_at	15	RING1	AL031228	6p21.3
218598_at	14	RINT-1	NM_021930	7q22.2
218535_s_at	14	RIOK2	NM_018343	5q15
202129_s_at	12	RIOK3	AW006290	18q11.2
202130_at	15	RIOK3	NM_003831	18q11.2
202131_s_at	15	RIOK3	NM_003831	18q11.2
213939_s_at	15	RIPX	AI871641	4q21.1

210251_s_at	13	RIPX; KIAA0871	AF112221	4q21.1
91816_f_at	15	RKHD1	C18318	---
218247_s_at	15	RKHD2	NM_016626	18q21.1
204243_at	15	RLF	NM_012421	1p32
212467_at	15	RME8	AB014578	3q22.1
201785_at	15	RNASE1	NM_002933	14q11.2
206111_at	11	RNASE2	NM_002934	14q24-q31
205158_at	15	RNASE4	NM_002937	14q11.1
213397_x_at	14	RNASE4	AI761728	14q11.1
218497_s_at	15	RNASEH1	BG534527	2p25
203022_at	15	RNASEH2A	NM_006397	19p13.2
218269_at	15	RNASEN	NM_013235	5p13.3
217983_s_at	15	RNASET2	NM_003730	6q27
217984_at	15	RNASET2	NM_003730	6q27
207801_s_at	15	RNF10	NM_014868	12q24.31
202636_at	15	RNF103	NM_005667	2p11.2
218761_at	15	RNF111	NM_017610	15q21
209565_at	15	RNF113A	BC000832	Xq25-q26
219021_at	14	RNF121	NM_018320	11q13.3
215031_x_at	12	RNF126	BG420893	19p13.3
201779_s_at	15	RNF13	NM_007282	3q25.1
201780_s_at	15	RNF13	NM_007282	3q25.1
217865_at	15	RNF130	NM_018434	5q35.3
218738_s_at	15	RNF138	NM_016271	18q12.1
201823_s_at	15	RNF14	NM_004290	5q23.3-q31.1
204040_at	15	RNF144	NM_014746	2p25.2
221430_s_at	14	RNF146	NM_030963	6q22.1-q22.33
220985_s_at	10	RNF170	NM_030954	8p11.21 /// 8p11.21
210075_at	11	RNF172	AF151074	19p13.3
219035_s_at	15	RNF34	NM_025126	12q24.31
218528_s_at	15	RNF38	NM_022781	---
212696_s_at	15	RNF4	BF968633	4p16.3
201962_s_at	13	RNF41	NM_005785	12q13.13
203286_at	15	RNF44	NM_014901	5q35.3
209111_at	15	RNF5	BC004155	6p21.3
209682_at	15	RNF56	U26710	3q13.11-q13.12
203403_s_at	15	RNF6	NM_005977	13q12.2
209845_at	15	RNF61	AF117233	7q34
218286_s_at	15	RNF7	NM_014245	3q22-q24
210541_s_at	13	RNF76; TRIM27	AF230394	6p22
203160_s_at	15	RNF8	NM_003958	6p21.3
215945_s_at	13	RNF86; KIAA0517	BC005016	4q31.3
204208_at	13	RNGTT	NM_003800	6q16
206050_s_at	15	RNH1	NM_002939	11p15.5
202683_s_at	15	RNMT	NM_003799	18p11.22-p11.23
202684_s_at	10	RNMT	AB020966	18p11.22-p11.23
218993_at	13	RNMTL1	NM_018146	17p13.3
200087_s_at	15	RNP24	AK024976	12q24.31
212430_at	15	RNPC1	AL109955	20q13.31
207941_s_at	15	RNPC2	NM_004902	20q11.23

208270_s_at	15	RNPEP	NM_020216	1q32
218301_at	14	RNPEPL1	NM_018226	2q37.3
200060_s_at	15	RNPS1	BC001659	16p13.3
215011_at	13	RNU17D	AJ006835	1p36.1
207438_s_at	15	RNUT1	NM_005701	15q23
213194_at	15	ROBO1	BF059159	3p14.2
220758_s_at	14	ROBO4	NM_019055	11q24.2
209882_at	15	ROC1	AF084462	1q22
213044_at	15	ROCK1	N22548	18q11.2
214578_s_at	14	ROCK1	AV683882	18q11.2
202762_at	15	ROCK2	AL049383	2p24
211504_x_at	14	ROCK2	D87931	2p24
214697_s_at	12	ROD1	AW190873	9q33.1
210320_s_at	13	ROK1	AF077033	17q21.1
220425_x_at	10	ROPN1	NM_017578	3q21.2
205805_s_at	14	ROR1	NM_005012	1p32-p31
210479_s_at	15	ROR1	L14611	15q21-q22
36019_at	15	RP	L26260	6p21.3
214357_at	12	RP1-106H8.1	AL035295	1q24.1
214755_at	13	RP11-229P13.18	AK022632	9q34.3
205191_at	12	RP2	NM_006915	Xp11.4-p11.21
213058_at	15	RP3-477H23.1	AL033538	---
212774_at	15	RP58	AJ223321	1q44-qter
201528_at	15	RPA1	NM_002945	17p13.3
201529_s_at	15	RPA1	NM_002945	17p13.3
215088_s_at	15	RPA1	BG110532	---
201756_at	15	RPA2	NM_002946	1p35
209507_at	15	RPA3	BC005264	7p22
209317_at	15	RPA5	AF008442	6p21.1
218441_s_at	14	RPAP1	NM_015540	15q14
216282_x_at	14	RPB3	AJ224143	16q13-q21
209382_at	15	RPC62	U93867	1q21.2
221614_s_at	14	RPH3AL	BC005153	17p13.3
212973_at	15	RPIA	AI692341	2p11.2
200725_x_at	15	RPL10	NM_006013	Xq28
217379_at	15	RPL10	AL121934	---
221989_at	15	RPL10	AW057781	Xq28
200036_s_at	15	RPL10A	NM_007104	6p21.3-p21.2
217559_at	15	RPL10L	AI001784	14q13-q21
200010_at	15	RPL11	NM_000975	1p36.1-p35
200088_x_at	15	RPL12	AK026491	---
200809_x_at	15	RPL12	NM_000976	9q34
214271_x_at	15	RPL12	AA281332	9q34
208929_x_at	15	RPL13	BC004954	16q24.3
212191_x_at	15	RPL13	AW574664	16q24.3
212734_x_at	15	RPL13	AI186735	16q24.3
212933_x_at	15	RPL13	AA961748	16q24.3
214351_x_at	15	RPL13	AA789278	16q24.3
214976_at	14	RPL13	AI554467	16q24.3
200715_x_at	15	RPL13A	BC000514	19q13.3

200716_x_at	15	RPL13A	BC000514	19q13.3
210646_x_at	15	RPL13A	BC001675	19q13.3
211942_x_at	15	RPL13A	BF979419	19q13.3
212790_x_at	15	RPL13A	BF942308	19q13.3
200074_s_at	15	RPL14	U16738	3p22-p21.2
213588_x_at	15	RPL14	AA838274	3p22-p21.2
217266_at	15	RPL15	Z97353	---
200038_s_at	15	RPL17	NM_000985	18q21
212270_x_at	15	RPL17	BG168283	18q21
212537_x_at	15	RPL17	BE733979	18q21
222216_s_at	13	RPL17L	AK026857	11p15.5-p15.4
200022_at	15	RPL18	NM_000979	19q13
214335_at	11	RPL18	AI669349	19q13
222297_x_at	13	RPL18	AV738806	19q13
200869_at	15	RPL18A	NM_000980	19p13
216383_at	15	RPL18A	U52111	---
200029_at	15	RPL19	NM_000981	17q11.2-q12
200012_x_at	15	RPL21	NM_000982	13q12.2
217340_at	12	RPL21	AL024509	---
220960_x_at	15	RPL22	NM_000983	1p36.3-p36.2
221726_at	15	RPL22	BE250348	1p36.3-p36.2
200888_s_at	15	RPL23	NM_000978	17q
214744_s_at	10	RPL23	AK021960	---
203012_x_at	15	RPL23A	NM_000984	17q11
213084_x_at	15	RPL23A	BF125158	17q11
221634_at	11	RPL23AL1	BC000596	2q14
200013_at	15	RPL24	NM_000986	3q12
214143_x_at	15	RPL24	AI560573	3q12
222229_x_at	15	RPL26	AL121871	---
218830_at	15	RPL26L1	NM_016093	5q35.2
200025_s_at	15	RPL27	NM_000988	17q21.1-q21.2
213642_at	15	RPL27	BE312027	17q21.1-q21.2
203034_s_at	15	RPL27A	NM_000990	11p15
212044_s_at	14	RPL27A	BE737027	11p15
200003_s_at	15	RPL28	NM_000991	19q13.4
200823_x_at	15	RPL29	NM_000992	3p21.3-p21.2
213969_x_at	15	RPL29	BF683426	---
216177_at	12	RPL29	AW582267	---
216570_x_at	15	RPL29	AL096829	---
201217_x_at	15	RPL3	NM_000967	22q13
211073_x_at	15	RPL3	BC006483	22q13 /// 22q13
212039_x_at	15	RPL3	BG339228	22q13
215963_x_at	15	RPL3	Z98200	6q21
200062_s_at	15	RPL30	L05095	8q22
200962_at	15	RPL31	AI348010	2q12.1
200963_x_at	15	RPL31	NM_000993	2q12.1
200674_s_at	15	RPL32	NM_000994	3p25-p24
200026_at	15	RPL34	NM_000995	4q25
200002_at	15	RPL35	NM_007209	9q34.1
217347_at	15	RPL35	Z82202	---

213687_s_at	15	RPL35A	BE968801	3q29-qter
219762_s_at	15	RPL36	NM_015414	19p13.3
201406_at	15	RPL36A	NM_021029	Xq22.1
207585_s_at	15	RPL36AL	NM_001001	14q21
200092_s_at	15	RPL37	BF216701	---
201429_s_at	15	RPL37A	NM_000998	2q35
213459_at	15	RPL37A	AU155515	2q35
214041_x_at	15	RPL37A	BE857772	2q35
202028_s_at	15	RPL38	BC000603	17q23-q25
202029_x_at	15	RPL38	NM_000999	17q23-q25
221943_x_at	15	RPL38	AW303136	17q23-q25
208695_s_at	15	RPL39	BC001019	Xq22-q24
210115_at	15	RPL39L1	L05096	3q27
206768_at	15	RPL3L	NM_005061	16p13.3
200089_s_at	15	RPL4	AI953886	15q22
201154_x_at	15	RPL4	NM_000968	15q22
211710_x_at	15	RPL4	BC005817	15q22 /// 15q22
201492_s_at	15	RPL41	NM_021104	12q13
200937_s_at	15	RPL5	NM_000969	1p22.1
213080_x_at	15	RPL5	BF214492	1p22.1
200034_s_at	15	RPL6	NM_000970	12q24.1
200717_x_at	15	RPL7	NM_000971	8q13.3
212042_x_at	15	RPL7	BG389744	8q13.3
217092_x_at	15	RPL7	AL031589	---
217740_x_at	15	RPL7A	NM_000972	9q34
200936_at	15	RPL8	NM_000973	8q24.3
200032_s_at	15	RPL9	NM_000661	4p13
201033_x_at	15	RPLP0	NM_001002	12q24.2
208856_x_at	15	RPLP0	BC003655	12q24.2
211720_x_at	15	RPLP0	BC005863	12q24.2 /// 12q24.2
211972_x_at	15	RPLP0	AI953822	12q24.2
214167_s_at	15	RPLP0	AA555113	---
200763_s_at	15	RPLP1	NM_001003	15q22
200909_s_at	15	RPLP2	NM_001004	11p15.5-p15.4
201011_at	15	RPN1	NM_002950	3q21.3-q25.2
208689_s_at	15	RPN2	BC003560	20q12-q13.1
213399_x_at	15	RPN2	AI560720	20q12-q13.1
213491_x_at	15	RPN2	AL514285	20q12-q13.1
204245_s_at	15	RPP14	NM_007042	3p21.2
218836_at	15	RPP21	NM_024839	6p21.32
203436_at	15	RPP30	NM_006413	10q23.32-q23.33
215743_at	10	RPP38	AL134489	10p13
213427_at	14	RPP40	NM_006638	6p25.1
217943_s_at	15	RPRC1	NM_018067	1p34.3
200095_x_at	15	RPS10	AA320764	6p21.31
200817_x_at	15	RPS10	NM_001014	6p21.31
211542_x_at	15	RPS10	BC004334	---
214001_x_at	14	RPS10	AW302047	6p21.31
217336_at	14	RPS10	AL118510	---
200031_s_at	15	RPS11	NM_001015	19q13.3

213350_at	14	RPS11	BF680255	19q13.3
213377_x_at	15	RPS12	AI799007	6q23.1
200018_at	15	RPS13	NM_001017	11p15
208645_s_at	15	RPS14	AF116710	---
208646_at	15	RPS14	AF116710	---
200819_s_at	15	RPS15	NM_001018	19p13.3
200781_s_at	15	RPS15A	NM_001019	16p
201258_at	15	RPS16	NM_001020	19q13.1
213890_x_at	15	RPS16	AI200589	19q13.1
201665_x_at	15	RPS17	NM_001021	15q
211487_x_at	15	RPS17	BC004886	---
212578_x_at	15	RPS17	BF026595	22q12.3
216348_at	15	RPS17	AL049693	---
201049_s_at	15	RPS18	NM_022551	6p21.3
202649_x_at	15	RPS19	NM_001022	19q13.2
213414_s_at	15	RPS19	BE259729	19q13.2
203107_x_at	15	RPS2	NM_002952	16p13.3
212433_x_at	15	RPS2	AA630314	16p13.3
217466_x_at	14	RPS2	L48784	---
221798_x_at	15	RPS2	AI183766	16p13.3
200949_x_at	15	RPS20	NM_001023	8q12
214003_x_at	15	RPS20	BF184532	8q12
216246_at	13	RPS20	AF113008	8q12
216247_at	12	RPS20	AF113008	8q12
200834_s_at	15	RPS21	NM_001024	20q13.3
214097_at	15	RPS21	AW024383	20q13.3
200926_at	15	RPS23	NM_001025	5q14.1
200061_s_at	15	RPS24	BC000523	10q22-q23
200091_s_at	15	RPS25	AA888388	11q23.3
217753_s_at	15	RPS26	NM_001029	12q13
200741_s_at	15	RPS27	NM_001030	1q21
200017_at	15	RPS27A	NM_002954	2p16
218007_s_at	15	RPS27L	BC003667	15q22.1
201094_at	15	RPS29	NM_001032	14q
208692_at	15	rpS3	U14990	11q13.3-q13.5
200099_s_at	15	RPS3A	AL356115	---
201257_x_at	15	RPS3A	NM_001006	4q31.2-q31.3
212391_x_at	15	RPS3A	AI925635	---
200933_x_at	15	RPS4X	NM_001007	Xq13.1
213347_x_at	15	RPS4X	AW132023	Xq13.1
216342_x_at	15	RPS4X	AL121916	---
201909_at	10	RPS4Y1	NM_001008	Yp11.3
200024_at	15	RPS5	NM_001009	19q13.4
200081_s_at	15	RPS6	BE741754	9p21
201254_x_at	15	RPS6	NM_001010	9p21
209134_s_at	15	RPS6	BC000524	9p21
203379_at	11	RPS6KA1	NM_002953	3
204906_at	15	RPS6KA2	BC002363	6q27
212912_at	15	RPS6KA2	AI992251	6q27
203843_at	14	RPS6KA3	AA906056	Xp22.2-p22.1

204632_at	11	RPS6KA4	NM_003942	11q11-q13
204635_at	15	RPS6KA5	NM_004755	14q31-q32.1
218909_at	15	RPS6KC1	NM_012424	1q41
200082_s_at	15	RPS7	AI805587	2p25
213941_x_at	15	RPS7	AI970731	2p25
200858_s_at	15	RPS8	NM_001012	1p34.1-p32
214317_x_at	15	RPS9	BE348997	19q13.4
217747_s_at	15	RPS9	NM_001013	19q13.4
213179_at	15	RQCD1	BG289914	2q35
204802_at	15	RRAD	NM_004165	16q22
204803_s_at	15	RRAD	NM_004165	16q22
201628_s_at	15	RRAGA	NM_006570	9p22.1
221523_s_at	15	RRAGD	AL138717	6q15-q16
221524_s_at	15	RRAGD	AL138717	6q15-q16
212647_at	15	RRAS	NM_006270	19q13.3-qter
208456_s_at	13	RRAS2	NM_012250	11p15.2
212589_at	15	RRAS2	AI753792	11p15.2
212590_at	15	RRAS2	AI431643	11p15.2
201204_s_at	15	RRBP1	AA706065	20p12
203704_s_at	15	RREB1	AW118862	6p25
201476_s_at	15	RRM1	AI692974	11p15.5
201477_s_at	15	RRM1	NM_001033	11p15.5
201890_at	13	RRM2	BE966236	2p25-p24
209773_s_at	15	RRM2	BC001886	2p25-p24
216902_s_at	12	RRN3	AF001549	---
218307_at	15	RSAD1	NM_018346	17q21.33
218347_at	15	RSAFD1	NM_018264	7q11.22
213694_at	15	RSBN1	AW027347	1p13.1
214583_at	10	RSC1A1	AI268381	1p36.1
201975_at	13	RSN	NM_002956	12q24.3
214684_at	15	RSRFC4	X63381	15q26
210999_s_at	15	RSS	U66065	7p12-p11.2
201980_s_at	15	RSU1	NM_012425	10p13
203594_at	15	RTCD1	NM_003729	1p21.3
41037_at	15	RTEF1	U63824	12p13.2-p13.3
210222_s_at	14	RTN1	BC000314	14q21-q22
34408_at	15	RTN2	AF004222	19q13.32
219549_s_at	15	RTN3	NM_006054	11q13
218243_at	15	RUFY1	NM_025158	5q35.3
219957_at	14	RUFY2	NM_017987	10q22.1
203724_s_at	13	RUFY3	NM_014961	4q21.1
205529_s_at	15	RUNX1T1	NM_004349	8q22
206949_s_at	13	RUSC1	NM_014328	1q21-q22
203014_x_at	15	RUTBC3	NM_015705	22q13.1-q13.2
214779_s_at	15	RUTBC3	R51077	22q13.1-q13.2
201614_s_at	13	RUVBL1	NM_003707	3q21
201459_at	15	RUVBL2	NM_006666	19q13.3
212507_at	15	RW1	D87446	2q11.2
219598_s_at	15	RWDD1	NM_016104	6q13-q22.33
213555_at	10	RWDD2	AL049699	6q15

205087_at	15	RWDD3	NM_015485	1p22.1
202426_s_at	14	RXRA	BE675800	9q34.3
202449_s_at	15	RXRA	NM_002957	9q34.3
209148_at	15	RXRB	BC001167	6p21.3
212438_at	15	RY1	BG252325	2p13.3
212440_at	15	RY1	BG252325	2p13.3
201846_s_at	15	RYBP	NM_012234	3p14.2
202853_s_at	15	RYK	NM_002958	3q22
214172_x_at	15	RYK	BG032035	3q22
207557_s_at	15	RYR2	NM_001035	1q42.1-q43
214044_at	15	RYR2	BE968750	1q42.1-q43
206306_at	12	RYR3	NM_001036	15q14-q15
200872_at	15	S100A10	NM_002966	1q21
200660_at	15	S100A11	NM_005620	1q21
208540_x_at	15	S100A11	NM_021039	---
205863_at	14	S100A12	NM_005621	1q21
202598_at	15	S100A13	NM_005979	1q21 /// 1q21
218677_at	13	S100A14	NM_020672	1q21.1
203186_s_at	15	S100A4	NM_002961	1q21
217728_at	15	S100A6	NM_014624	1q21
202917_s_at	15	S100A8	NM_002964	1q21
218370_s_at	15	S100PBP	NM_022753	1p34.3
209503_s_at	15	S8	AF035309	17q23-q25 /// 17q23-q25
202797_at	15	SACM1L	NM_014016	3p21.3
213262_at	15	SACS	AI932370	13q12
217946_s_at	15	SAE1	NM_016402	19q13.33
201748_s_at	15	SAFB	NM_002967	19p13.3-p13.2
32099_at	15	SAFB2	D50928	19p13.3
213365_at	15	SAH	N64622	16p13.11
213283_s_at	15	SALL2	BG285616	14q11.1-q12
212845_at	15	SAMD4	AB028976	14q22.2
215495_s_at	15	SAMD4	AL117523	14q22.2
214967_at	11	SAMD4A	AU146983	---
201569_s_at	15	SAMM50	NM_015380	22q13.31
201570_at	15	SAMM50	NM_015380	22q13.31
220330_s_at	14	SAMSN1	NM_022136	21q11
37462_i_at	14	SAP 62	L21990	19p13.3-p13.2
220367_s_at	15	SAP130	NM_024545	2q21.1
208740_at	15	SAP18	BF593650	13q12.11
208741_at	13	SAP18	AW274856	13q12.11
208742_s_at	15	SAP18p	U78303	13q12.11
35820_at	15	SAP-3	M76477	5q31.3-q33.1 /// 5q31.3-q33.1
204899_s_at	11	SAP30	BF247098	4q34.1
204900_x_at	15	SAP30	NM_003864	4q34.1
219129_s_at	14	SAP30L	NM_024632	5q33.2
209229_s_at	14	SAPS1	BC002799	19q13.42
202791_s_at	15	SAPS2	NM_014678	22q13.33
217928_s_at	15	SAPS3	NM_018312	11q13

201542_at	15	SAR1	AY008268	10q22.2
201543_s_at	15	SAR1A	NM_020150	10q22.2
218254_s_at	15	SAR1B	NM_016103	5q31.2
202035_s_at	14	SARP2	AF017987	8p12-p11.1
202036_s_at	15	SARP2	AF017987	8p12-p11.1
202037_s_at	15	SARP2	AF017987	8p12-p11.1
200802_at	15	SARS	NM_006513	1p13.3-p13.1
200051_at	15	SART1	NM_005146	11q13.1
218854_at	15	SART2	NM_013352	6q22
200069_at	15	SART3	AI656011	12q24.1
209127_s_at	11	SART3	AW173076	12q24.1
209128_s_at	15	SART3	AI656011	12q24.1
203226_s_at	13	SAS	AL514076	12q13.3
203227_s_at	14	SAS	AL514076	12q13.3
209486_at	13	SAS10	BC004546	4q13.3
213236_at	13	SASH1	AK025495	6q24.3
41644_at	15	SASH1	AB018333	6q24.3
203455_s_at	15	SAT	NM_002970	Xp22.1
213988_s_at	15	SAT	BE971383	Xp22.1
203408_s_at	15	SATB1	NM_002971	3p23
213282_at	15	SATL1	BE501952	Xq21.1
213289_at	13	SATL1	BE221922	Xq21.1-q21.2
218276_s_at	15	SAV1	AI679398	14q13-q23
209820_s_at	12	SAZD	BC002361	16p13.3
39835_at	14	SBF1	U93181	22q13.33
211423_s_at	15	SC5DL; ERG3	D85181	11q23.3
206667_s_at	11	SCAMP1	AF005037	5q13.3-q14.1
206668_s_at	13	SCAMP1	NM_004866	5q13.3-q14.1
212416_at	15	SCAMP1	AV745949	5q13.3-q14.1
212417_at	14	SCAMP1	BF058944	5q13.3-q14.1
212425_at	15	SCAMP1	AL049223	5q13.3-q14.1
218143_s_at	15	SCAMP2	NM_005697	15q23-q25
201771_at	15	SCAMP3	NM_005698	1q21
212699_at	15	SCAMP5	BE222801	15q23
218206_x_at	15	SCAND1	NM_016558	20q11.1-q11.23
212329_at	15	SCAP	D83782	3p21.31
201819_at	13	SCARB1	NM_005505	12q24.31
201646_at	15	SCARB2	AA885297	---
201647_s_at	15	SCARB2	NM_005506	4q21.21-q21.22
213984_at	11	SCC-112	AW991219	4p14
201825_s_at	15	SCCPDH	NM_016002	1q44
201826_s_at	15	SCCPDH	NM_016002	1q44
200832_s_at	15	Scd	AB032261	10q23-q24
211569_s_at	15	SCHAD	AF001903	4q22-q26
204030_s_at	15	SCHIP1	NM_014575	3q25.33
214075_at	14	SCIRP10	AI984136	1q32.3
59705_at	15	SCLY	AA911739	2q37.3
221216_s_at	15	SCMH1	NM_012236	1p34
218793_s_at	13	SCML1	BF001786	Xp22.2-p22.1
207413_s_at	15	SCN5A	NM_000335	3p21

205241_at	13	SCO2	NM_005138	22q13.33
201339_s_at	15	SCP2	NM_002979	1p32
211733_x_at	15	SCP2	BC005911	1p32 /// 1p32
218217_at	15	SCPEP1	NM_021626	17q23.2
209868_s_at	15	scr2	D28482	2q24.3
205475_at	15	SCRG1	NM_007281	4q31-q32
201462_at	15	SCRN1	NM_014766	7p14.3-p14.1
219234_x_at	15	SCRN3	NM_024583	2q31.1
202541_at	15	SCYE1	BF589679	4q25
202542_s_at	15	SCYE1	NM_004757	4q25
205607_s_at	14	SCYL3	NM_020423	1q23.3
218607_s_at	15	SDAD1	NM_018115	4q21.21
212154_at	15	SDC2	AI380298	8q22-q23
212157_at	15	SDC2	AI380298	8q22-q23
212158_at	15	SDC2	AL577322	8q22-q23
202071_at	15	SDC4	NM_002999	20q12
200958_s_at	15	SDCBP	NM_005625	8q12
218649_x_at	15	SDCCAG1	NM_004713	14q22
218427_at	12	SDCCAG3	NM_006643	9q34.3
203090_at	15	SDF2	NM_006923	17q11.2
218681_s_at	13	SDF2L1	NM_022044	22q11.21
217855_x_at	15	SDF4	NM_016547	1p36.33
202228_s_at	15	SDFR1	NM_017455	15q22
201093_x_at	15	SDHA	NM_004168	5p15
222021_x_at	15	SDHA	AI348006	3q29
202675_at	15	SDHB	NM_003000	1p36.1-p35
214166_at	10	SDHB	AW294107	1p36.1-p35
202004_x_at	15	SDHC	NM_003001	1q21
202026_at	15	SDHD	NM_003002	11q23
218711_s_at	15	SDPR	NM_004657	2q32-q33
220180_at	12	SE57-1	NM_025214	18q21
201290_at	15	SEC11L1	NM_014300	15q25.2
216274_s_at	15	SEC11L1	N99438	15q25.2
207707_s_at	15	SEC13L1	NM_030673	3p25-p24
202084_s_at	15	SEC14L1	NM_003003	17q25.1-17q25.2
209206_at	15	SEC22L1	AV701283	1q21.2-q21.3
209207_s_at	11	SEC22L1	BC001364	1q21.2-q21.3
214257_s_at	14	SEC22L1	AA890010	1q21.2-q21.3
218703_at	14	SEC22L2	NM_012430	3q21.1
204344_s_at	11	SEC23A	NM_006364	14q13.3
212887_at	15	SEC23A	AI753659	14q13.3
201583_s_at	12	SEC23B	NM_006363	20p11.23
210293_s_at	15	SEC23B	BC005032	20p11.23
212900_at	15	SEC24A	AJ131244	5q31.2
212902_at	14	SEC24A	BE645231	5q31.2
202798_at	15	SEC24B	NM_006323	4q25
202361_at	15	SEC24C	NM_004922	10q22.3
202375_at	15	SEC24D	NM_014822	4q27
222127_s_at	15	SEC3	AK023461	4q12
200945_s_at	15	SEC31L1	NM_014933	4q21.3

215009_s_at	15	SEC31L1	U92014	4q21.3
219499_at	14	SEC61A2	NM_018144	10p14
203133_at	15	SEC61B	NM_006808	9q22.32-q31.3
203484_at	15	SEC61G	NM_014302	7p11.2
208942_s_at	15	Sec62	U93239	3q26.2-q27
208943_s_at	15	Sec62	U93239	3q26.2-q27
201914_s_at	12	SEC63	NM_007214	6q21
201915_at	11	SEC63	NM_007214	6q21
201916_s_at	15	SEC63	NM_007214	6q21
218265_at	15	SECISBP2	NM_024077	9q22.2
213492_at	13	SEDC; COL11A3	X06268	12q13.11-q13.2
221931_s_at	15	SEH1L	AV701173	18p11.21
206211_at	11	SELE	NM_000450	1q22-q25
214433_s_at	15	SELENBP1	NM_003944	1q21-q22
204563_at	12	SELL	NM_000655	1q23-q25
206049_at	12	SELP	NM_003005	1q22-q25
217811_at	15	SELT	NM_016275	3q25.1 /// 3q25.1
206805_at	10	SEMA3A	NM_006080	7p12.1
203788_s_at	14	SEMA3C	AI962897	7q21-q31
203789_s_at	15	SEMA3C	NM_006379	7q21-q31
219689_at	15	SEMA3G	NM_020163	3p21.31
35666_at	15	SEMA4	U38276	3p21.3
46665_at	15	SEMA4C	AI949392	2q11.2
219194_at	14	SEMA4G	NM_017893	10q24.32
205405_at	15	SEMA5A	NM_003966	5p15.2
213169_at	15	SEMA5A	BG109855	5p15.2
215028_at	10	SEMA6A	AB002438	5q23.1
215479_at	13	SEMA6A	AK000787	---
220454_s_at	11	SEMA6A	NM_020796	5q23.1
220574_at	12	SEMA6D	NM_024966	15q15.2
203871_at	15	SENP3	NM_015670	17p13
213184_at	12	SENP5	N48361	---
57703_at	15	SENP5	N32782	3q29
202319_at	14	SENP6	NM_015571	6q13-q14.3
200902_at	15	SEP15	NM_004261	1p31
213666_at	15	SEP2	AK026589	Xq25
208939_at	15	SEPHS1	AV682679	10p14
208941_s_at	13	SEPHS1	AV682679	10p14
200961_at	15	SEPHS2	NM_012248	16p12.1
201427_s_at	15	SEPP1	NM_005410	5q31
212698_s_at	15	SEPT10	BF966021	2q13
214720_x_at	15	SEPT10	BF981643	2q13
201307_at	15	SEPT11	AL534972	4q21.22
214293_at	15	SEPT11	AI539361	4q21.22
200015_s_at	15	SEPT2	AI191427	2q37
200778_s_at	15	SEPT2	AI191427	2q37
209767_s_at	14	SEPT5	AA702163	22q11.21
214298_x_at	12	SEPT6	AL568374	Xq25
213151_s_at	15	SEPT7	AU157515	7p14.3-p14.1
217721_at	15	SEPT7	NM_001788	7p14.3-p14.1

201194_at	15	SEPW1	NM_003009	19q13.3
209669_s_at	15	SERBP1	BC003049	1p31-p22
210466_s_at	15	SERBP1	BC002488	1p31-p22
219982_s_at	11	SERF1B	NM_022978	5q12.2-q13.3
217756_x_at	15	SERF2	NM_005770	15q15.1
211769_x_at	15	SERINC3	BC006088	20q13.1-13.3
212812_at	15	SERINC5	AI700633	---
200969_at	15	SERP1	NM_014445	3q25.1
200970_s_at	15	SERP1	AL136807	3q25.1
200971_s_at	15	SERP1	NM_014445	3q25.1
212268_at	14	SERPINB1	NM_030666	6p25
213572_s_at	14	SERPINB1	AI554300	6p25
211474_s_at	15	SERPINB6	BC004948	---
209723_at	12	SERPINB9	BC002538	6p25
212190_at	15	SERPINE2	AL541302	2q33-q35
202283_at	15	SERPINF1	NM_002615	17p13.1
200986_at	15	SERPING1	NM_000062	11q12-q13.1
207714_s_at	15	SERPINH1	NM_004353	11q13.5 /// 11q13.5
205352_at	15	SERPINI1	NM_005025	3q26.2
202656_s_at	15	SERTAD2	BG107456	2p15
202657_s_at	15	SERTAD2	NM_014755	2p15
219382_at	15	SERTAD3	NM_013368	19q13.2
218346_s_at	15	SESN1	NM_014454	6q21
200630_x_at	15	SET	AV702810	9q34
200631_s_at	15	SET	NM_003011	9q34
210231_x_at	15	SET	D45198	9q34
213047_x_at	15	SET	AI278616	9q34
213048_s_at	15	SET	W26593	9q34
215780_s_at	14	SET	Z95126	9q34
40189_at	15	SET	M93651	9q34
215373_x_at	13	SET07	AK022213	13q11-q13
220200_s_at	13	SET07	NM_020382	13q11-q13
203155_at	15	SETDB1	NM_012432	1q21
210755_at	13	SF	U46010	7q21.1
208313_s_at	15	SF1	NM_004630	11q13
201356_at	15	SF3A1	NM_005877	---
201357_s_at	14	SF3A1	NM_005877	22q12.2
216457_s_at	15	SF3A1	AK026080	---
203818_s_at	15	SF3A3	NM_006802	1p34.2
201070_x_at	13	SF3B1	AI739389	2q33.1
201071_x_at	15	SF3B1	NM_012433	2q33.1
214305_s_at	13	SF3B1	AW003030	2q33.1
200619_at	14	SF3B2	NM_006842	11q13.1 /// 11q13.1
200687_s_at	15	SF3B3	D13642	16q22.1 /// 16q22.1
200688_at	11	SF3B3	D13642	16q22.1
209044_x_at	15	SF3B4	BC004273	1q12-q21
221263_s_at	15	SF3B5	NM_031287	6q24.1 /// 6q24.1
201149_s_at	15	SFD	U67195	22q12.1-q13.2
221504_s_at	15	SFD	AF112204	8p22-q22.3
213370_s_at	13	SFMBT1	BF057298	3p21.31

33322_i_at	15	SFN	X57348	1p35.3
33323_r_at	10	SFN	X57348	1p35.3
201585_s_at	14	SFPQ	NM_005066	1p34.3
201586_s_at	15	SFPQ	NM_005066	1p34.3
214016_s_at	15	SFPQ	AL558875	1p34.3
221768_at	15	SFPQ	AV705803	1p34.3
204051_s_at	13	SFRP4	NM_003014	7p14.1
208863_s_at	15	SFRS1	M72709	17q21.3-q22
211784_s_at	15	SFRS1	BC006181	---
200892_s_at	15	SFRS10	BC000451	3q26.2-q27
200893_at	15	SFRS10	NM_004593	3q26.2-q27
200685_at	14	SFRS11	AU146237	1p31
200686_s_at	15	SFRS11	NM_004768	1p31
213742_at	13	SFRS11	AW241752	1p31
212721_at	15	SFRS12	AI810380	5q12.3
212001_at	15	SFRS14	AB002363	19p12
213505_s_at	13	SFRS14	BG252853	19p12
214092_x_at	15	SFRS14	AI928127	19p12
64371_at	12	SFRS14	AI978718	19p12
204978_at	13	SFRS16	NM_007056	19q13.3
200753_x_at	15	SFRS2	BE866585	17q25.3
200754_x_at	15	SFRS2	NM_003016	17q25.3
214882_s_at	15	SFRS2	BG254869	17q25.3
206989_s_at	15	SFRS2IP	NM_004719	12q13.11
213850_s_at	12	SFRS2IP	AI984932	12q13.11
202899_s_at	15	SFRS3	NM_003017	6p21
208672_s_at	15	SFRS3	BC000914	6p21
208673_s_at	15	SFRS3	AL514794	6p21
201696_at	15	SFRS4	NM_005626	1p35.2
203380_x_at	15	SFRS5	NM_006925	14q24
212266_s_at	15	SFRS5	AW084582	14q24
208804_s_at	15	SFRS6	AL031681	20q12-q13.1
213649_at	15	SFRS7	AA524053	2p22.1
214141_x_at	15	SFRS7	BF033354	2p22.1
202774_s_at	14	SFRS8	NM_004592	12q24.33
202775_s_at	15	SFRS8	NM_004592	12q24.33
200044_at	15	SFRS9	NM_003769	12q24.31
201698_s_at	15	SFRS9	NM_003769	12q24.31
213936_x_at	14	SFTPB	AW276646	2p12-p11.2
220974_x_at	15	SFXN3	NM_030971	10q24.32 /// 10q24.32
205120_s_at	15	SGCB	U29586	4q12
213543_at	14	SGCD	AA570453	5q33-q34
204688_at	15	SGCE	NM_003919	7q21-q22
207302_at	15	SGCG	NM_000231	13q12
201739_at	15	SGK	NM_005627	6q23
203889_at	15	SGNE1	NM_003020	15q13-q14
212321_at	15	SGPL1	BE999972	10q21
212322_at	15	SGPL1	BE999972	10q21
221268_s_at	12	SGPP1	NM_030791	14q23.2 /// 14q23.2

				/// 14q23.2
35626_at	15	SGSH	U30894	17q25.3
201396_s_at	11	SGTA	NM_003021	19p13
222140_s_at	14	SH120; AL844549.1	AK021758	1p36.13-q31.3
40149_at	15	SH2B	AL049924	16p12.1
202060_at	15	SH2BP1	NM_014633	11p15.3
204979_s_at	15	SH3BGR	NM_007341	21q22.3
201311_s_at	15	SH3BGRL	AL515318	Xq13.3
201312_s_at	15	SH3BGRL	NM_003022	Xq13.3
221269_s_at	14	SH3BGRL3	NM_031286	1p35-p34.3 /// 1p35-p34.3
209370_s_at	10	SH3BP2	BE502377	4p16.3
201810_s_at	12	SH3BP5	AL562152	3p24.3
201811_x_at	15	SH3BP5	NM_004844	3p24.3
209253_at	15	SH3D4	AF037261	8p21.2
201851_at	12	SH3GL1	NM_003025	19p13.3
205637_s_at	14	SH3GL3	NM_003027	15q24
209090_s_at	15	SH3GLB1	AL049597	1p22
218813_s_at	12	SH3GLB2	NM_020145	9q34
211819_s_at	11	SH3P12	AF136381	10q23.3-q24.1
204019_s_at	15	SH3YL1	NM_015677	2p25.3
213307_at	12	SHANK	AF131790	11q13.2
201469_s_at	11	SHC1	AI809967	1q21
214853_s_at	15	SHC1	AI091079	1q22
213464_at	15	SHC2	AV705938	19p13.3
219493_at	15	SHCBP1	NM_024745	16q11.2
202276_at	15	SHFM1	NM_006304	7q21.3-q22.1
214095_at	13	SHMT2	AW190316	12q13.2
214096_s_at	15	SHMT2	AW190316	12q13.2
214437_s_at	14	SHMT2	NM_005412	12q12-q14
202777_at	15	SHOC2	NM_007373	10q25
214527_s_at	15	SHS	AB041836	Xp11.23
209848_s_at	11	SI	U01874	12q13-q14
202980_s_at	15	SIAH1	AI953523	16q12
202981_x_at	15	SIAH1	NM_003031	16q12
221833_at	15	SIAH1	AV700132	16q12
221834_at	13	SIAH1	AV700132	16q12.1
201998_at	15	SIAT1	AI743792	3q27-q28
213355_at	13	SIAT10	AI989567	3q12.2
221551_x_at	11	SIAT7D	AW044319	9q34
208924_at	15	Sid1669	AB024703	1pter-p22.1
56256_at	15	SIDT2	AA150165	11q23.2
218921_at	15	SIGIRR	NM_021805	11p15.5
52940_at	14	SIGIRR	AA085764	11p15.5
213221_s_at	12	SIK2	AB018324	11q23.2
204665_at	15	SIKE	NM_025073	1p13.1
221705_s_at	12	SIKE	BC005934	1p13.1 /// 1p13.1
218046_s_at	15	SIL1	BE964789	10q22.1
200704_at	15	SIMPLE	AB034747	16p13.3-p12
210880_s_at	12	SIN	AB001467	14q11.2-q12

209352_s_at	11	SIN3B	AB014600	19p13.12
39705_at	14	SIN3B	AB014600	19p13.12
201381_x_at	15	SIP	AF057356	1q24-q25
210691_s_at	15	SIP	AF275803	1q24-q25
205063_at	14	SIP1	NM_003616	14q13
209376_x_at	12	SIP1	AF030234	12q13.11
210779_x_at	15	SIP1-beta	AB037701	14q13
211115_x_at	15	SIP1-delta	AB037703	14q13
211114_x_at	15	SIP1-gamma	AB037702	14q13
204164_at	10	SIPA1	NM_006747	11q13
202254_at	10	SIPA1L1	AB007900	14q24.1
202255_s_at	10	SIPA1L1	NM_015556	14q24.1
218878_s_at	15	SIRT1	NM_012238	10q22.1
220605_s_at	15	SIRT2	NM_012237	19q13
219185_at	15	SIRT5	NM_012241	6p23
221010_s_at	13	SIRT5	NM_031244	6p23 /// 6p23
218797_s_at	14	SIRT7	NM_016538	17q25
218225_at	15	SITPEC	NM_016581	19p13.2
203489_at	15	SIVA	NM_006427	14q32.33
222030_at	15	SIVA	AW024335	14q32.33
210792_x_at	15	SIVA; CD27BP	AF033111	14q32.33
209043_at	15	SK1	AF033026	4q24
217786_at	13	SKB1	NM_006109	14q11.2-q21
215424_s_at	15	SKIIP	AV689564	14q24.3
217591_at	12	SKIL	BF725121	3q26
202781_s_at	14	SKIP	AI806031	17p13.3
202782_s_at	12	SKIP	NM_016532	17p13.3
200711_s_at	15	SKP1A	BE964043	5q31
200718_s_at	15	SKP1A	AA927664	5q31
200719_at	13	SKP1A	BE964043	5q31
207974_s_at	15	SKP1A	NM_006930	5q31
203625_x_at	15	SKP2	BG105365	5p13
206052_s_at	15	SLBP	NM_006527	4p16.3
203123_s_at	15	SLC11A2	AU154469	12q13
204404_at	15	SLC12A2	NM_001046	5q23.3
215274_at	10	SLC12A3	AI627943	16q13
220740_s_at	15	SLC12A6	NM_005135	15q13-q15
218066_at	15	SLC12A7	NM_006598	5p15
220371_s_at	14	SLC12A9	NM_020246	7q22
205856_at	10	SLC14A1	NM_015865	18q11-q12
202234_s_at	15	SLC16A1	BF511091	1p12
202235_at	15	SLC16A1	BF511091	1p12
202236_s_at	15	SLC16A1	NM_003051	1p12
209900_s_at	15	SLC16A1	AL162079	1p12
202855_s_at	15	SLC16A3	AL513917	17q25
202856_s_at	15	SLC16A3	NM_004207	17q25
217691_x_at	15	SLC16A3	AA853175	17q25
213590_at	12	SLC16A5	AA705628	17q25.2
207057_at	12	SLC16A7	NM_004731	12q13
213549_at	13	SLC18A2	AI890972	10q25

213664_at	11	SLC1A1	AW235061	9p24
202800_at	15	SLC1A3	NM_004172	5p13
209610_s_at	14	SLC1A4	BF340083	2p15-p13
212810_s_at	15	SLC1A4	W72527	2p15-p13
212811_x_at	15	SLC1A4	AI889380	2p15-p13
208916_at	12	SLC1A5	AF105230	19q13.3
201920_at	15	SLC20A1	NM_005415	2q11-q14
207408_at	11	SLC22A14	NM_004803	3p21.3
218675_at	10	SLC22A17	NM_020372	14q11.2
204981_at	15	SLC22A18	NM_002555	11p15.5
205896_at	12	SLC22A4	NM_003059	5q31.1
209236_at	14	SLC23A2	AL389886	---
57588_at	11	SLC24A3	R62432	20p13
207088_s_at	15	SLC25A11	NM_003562	17p13.3
203339_at	15	SLC25A12	AI887457	2q24
203340_s_at	15	SLC25A12	AI887457	2q24
203775_at	15	SLC25A13	NM_014251	7q21.3
204587_at	13	SLC25A14	NM_003951	Xq24
218653_at	13	SLC25A15	NM_014252	13q14
210686_x_at	15	SLC25A16	BC001407	10q21.3
214140_at	13	SLC25A16	AI827990	10q21.3
211754_s_at	14	SLC25A17	BC005957	22q13.2 /// 22q13.2
203658_at	15	SLC25A20	BC001689	3p21.31
218725_at	10	SLC25A22	NM_024698	11p15.5
220182_at	10	SLC25A23	NM_024103	19p13.3
204342_at	13	SLC25A24	NM_013386	1p13.3
200030_s_at	15	SLC25A3	NM_002635	12q23
221020_s_at	15	SLC25A32	NM_030780	8q22.3 /// 8q22.3
201917_s_at	15	SLC25A36	NM_018155	3q23
202825_at	15	SLC25A4	NM_001151	4q35
214821_at	14	SLC25A4	AF052119	---
200657_at	15	SLC25A5	NM_001152	Xq24-q26
212085_at	15	SLC25A6	AA916851	Xp22.32 and Yp
212826_s_at	15	SLC25A6	AI961224	Xp22.32 and Yp
214951_at	15	SLC26A10	AL050358	12q13
205097_at	11	SLC26A2	AI025519	5q31-q34
222217_s_at	15	SLC27A3	BC003654	1q22
219932_at	15	SLC27A6	NM_014031	5q31.1
201801_s_at	15	SLC29A1	NM_004955	6p21.1-p21.2
201802_at	15	SLC29A1	NM_004955	6p21.1-p21.2
201250_s_at	15	SLC2A1	NM_006516	1p35-p31.3
221024_s_at	14	SLC2A10	NM_030777	20q13.1 /// 20q13.1
216236_s_at	12	SLC2A14	AL110298	12p13.31
222088_s_at	15	SLC2A14	AA778684	12p13.31
202497_x_at	13	SLC2A3	AI631159	12p13.3
202498_s_at	11	SLC2A3	BE550486	12p13.3
202499_s_at	15	SLC2A3	NM_006931	12p13.3
218494_s_at	15	SLC2A4RG	BE898559	20q13.33
218985_at	10	SLC2A8	NM_014580	9q34.13
212907_at	13	SLC30A1	AI972416	1q32-q41

218989_x_at	15	SLC30A5	NM_022902	5q12.1
202614_at	15	SLC30A9	NM_006345	4p13-p12
203971_at	11	SLC31A1	NM_001859	9q31-q32 /// 9q31-q32
204204_at	12	SLC31A2	NM_001860	9q31-q32
203164_at	15	SLC33A1	BE464756	3q25.31
203306_s_at	15	SLC35A1	NM_006416	6q15
207439_s_at	14	SLC35A2	NM_005660	Xp11.23-p11.22
207440_at	10	SLC35A2	NM_005660	Xp11.23-p11.22
206770_s_at	10	SLC35A3	NM_012243	1p21
218519_at	15	SLC35A5	NM_017945	3q13.2
202433_at	15	SLC35B1	NM_005827	17q21.33
209711_at	15	SLC35D1	N80922	1p32-p31
209712_at	13	SLC35D1	AI769637	1p32-p31
220796_x_at	15	SLC35E1	NM_024881	19p13.12
79005_at	13	SLC35E1	AA504646	19p13.12
217122_s_at	15	SLC35E2	AL031282	---
218988_at	14	SLC35E3	NM_018656	12q15
218826_at	14	SLC35F2	NM_017515	11q23.1
218237_s_at	15	SLC38A1	NM_030674	12q13.11
218041_x_at	15	SLC38A2	NM_018573	12q
220924_s_at	15	SLC38A2	NM_018976	12q
214830_at	10	SLC38A6	AI537540	14q23.1
217778_at	11	SLC39A1	NM_014437	1q21
212110_at	15	SLC39A14	D31887	8p21.2
202088_at	15	SLC39A6	AI635449	18q12.2
202089_s_at	15	SLC39A6	NM_012319	18q12.2
202667_s_at	14	SLC39A7	NM_006979	6p21.3
219869_s_at	11	SLC39A8	AW139759	4q22-q24
217859_s_at	13	SLC39A9	NM_018375	14q24.1
205799_s_at	11	SLC3A1	NM_000341	2p16.3
212216_at	15	SLC3A1	AW000954	2p22.1
212217_at	15	SLC3A1	AU154782	2p16.3
200924_s_at	13	SLC3A2	NM_002394	11q13
219175_s_at	15	SLC41A3	NM_017836	3q21.2
204394_at	14	SLC43A1	NM_003627	11p11.2-p11.1
213113_s_at	15	SLC43A3	AI630178	11q11
205592_at	14	SLC4A1	NM_000342	17q21-q22
221770_at	11	SLC4A10	BE964473	---
218682_s_at	10	SLC4A1AP	NM_018158	2p23.3
202111_at	15	SLC4A2	NM_003040	7q35-q36
205918_at	15	SLC4A3	NM_005070	2q36
203908_at	15	SLC4A4	NM_003759	4q21
207604_s_at	13	SLC4A7	NM_003615	3p22
209884_s_at	15	SLC4A7	AF047033	3p22
206628_at	15	SLC5A1	NM_000343	22q13.1
202219_at	15	SLC6A8	NM_005629	Xq28
213843_x_at	15	SLC6A8	AW276522	Xq28
212295_s_at	15	SLC7A1	AA148507	13q12-q14
203578_s_at	11	SLC7A6	NM_003983	16q22.1

203580_s_at	13	SLC7A6	NM_003983	16q22.1
204588_s_at	10	SLC7A7	NM_003982	14q11.2
216092_s_at	12	SLC7A8	AL365347	14q11.2
207053_at	10	SLC8A1	NM_021097	2p23-p22
210804_x_at	10	SLC8A1	AF128524	2p23-p22
208039_at	12	SLC9A2	NM_003048	2q11.2
203909_at	15	SLC9A6	NM_006359	Xq26.3
204368_at	15	SLCO2A1	NM_005630	3q21
203473_at	13	SLCO2B1	NM_007256	11q13
210542_s_at	15	SLCO3A1	BC000585	15q26
219229_at	12	SLCO3A1	NM_013272	15q26
209897_s_at	15	SLIL3; Slit-2	AF055585	4p15.2
206874_s_at	15	SLK	AL138761	10q25.1
206875_s_at	15	SLK	NM_014720	10q25.1
210105_s_at	15	SLK	M14333	6q21
216033_s_at	15	SLK	S74774	6q21
205374_at	14	SLN	NM_003063	11q22-q23
209948_at	13	SLO-BETA	U61536	5q34
33736_at	15	SLP-1	Y16522	15q24-q25
217828_at	15	SLTM	NM_024755	15q21.3
214850_at	11	SMA4	X75940	5q13
203075_at	15	SMAD2	AW151617	18q21.1
203077_s_at	15	SMAD2	NM_005901	18q21.1
218284_at	15	SMAD3	NM_015400	15q22.31
202527_s_at	15	SMAD4	NM_005359	18q21.1
207069_s_at	15	SMAD6	NM_005585	15q21-q22
204790_at	15	SMAD7	NM_005904	18q21.1
200084_at	15	SMAP	BE748698	11p15.2
201784_s_at	15	SMAP	NM_014267	11p15.2
218137_s_at	15	SMAP1	AU153608	6q12-q13
221509_at	15	smap-3	AB014731	12q24.31
203874_s_at	15	SMARCA1	M88163	Xq25
203875_at	15	SMARCA1	NM_003069	Xq25
206542_s_at	15	SMARCA2	AV725365	9p22.3
206544_x_at	15	SMARCA2	NM_003070	9p22.3
212257_s_at	14	SMARCA2	AW131754	9p22.3
217707_x_at	15	SMARCA2	AI535683	---
202983_at	15	SMARCA3	AI760760	3q25.1-q26.1
213720_s_at	15	SMARCA4	AI831675	19p13.2
202303_x_at	15	SMARCA5	NM_003601	4q31.1-q31.2
213251_at	15	SMARCA5	AV712064	4q31.1-q31.2
213859_x_at	13	SMARCA5	AI652586	4q31.1-q31.2
201073_s_at	15	SMARCC1	AL040633	3p23-p21
201074_at	15	SMARCC1	AA593983	3p23-p21
201075_s_at	15	SMARCC1	NM_003074	3p23-p21
201320_at	15	SMARCC2	BF663402	12q13.13
201321_s_at	15	SMARCC2	NM_003075	12q13-q14
203183_s_at	15	SMARCD1	NM_003076	12q13-q14
209518_at	10	SMARCD1	AI869240	12q13-q14
204099_at	15	SMARCD3	NM_003078	7q35-q36

211988_at	15	SMARCE1	BG289800	17q21.2
211989_at	15	SMARCE1	NM_003079	17q21.2
217758_s_at	15	SMBP	BG104571	10q24.2
31861_at	13	SMBP2	L14754	11q13.2-q13.4
201589_at	15	SMC1L1	D80000	Xp11.22-p11.21
204240_s_at	11	SMC2L1	NM_006444	9q31.2
213253_at	13	SMC2L1	AU154486	9q31.2
201663_s_at	15	SMC4L1	NM_005496	3q26.1
201664_at	15	SMC4L1	AL136877	3q26.1
212926_at	14	SMC5L1	AW183677	9q21.2
212927_at	14	SMC5L1	AW183677	9q21.2
218781_at	15	SMC6L1	NM_024624	2p24.3
202383_at	14	SMCX	NM_004187	Xp11.22-p11.21
206700_s_at	10	SMCY	NM_004653	Yq11
203852_s_at	15	SMN1	NM_000344	5q13
200071_at	15	SMNDC1	BF224259	10q23
210357_s_at	15	SMOX	BC000669	20p13
213624_at	15	SMPDL3A	AA873600	6q22.32
219772_s_at	15	SMPX	NM_014332	Xp22.1
202043_s_at	15	SMS	NM_004595	Xp22.1
207390_s_at	15	SMTN	NM_006932	22q12.2
209427_at	15	SMTN	AF064238	22q12.2
218685_s_at	12	SMUG1	NM_014311	12q13.11-q13.3
212666_at	15	SMURF1	AB046845	7q21.1-q31.1
205596_s_at	15	SMURF2	AY014180	17q22-q23
212921_at	12	SMYD2	AF070592	1q32.3
212922_s_at	15	SMYD2	AI809870	1q32.3
218788_s_at	15	SMYD3	NM_022743	1q44
44673_at	12	SN	N53555	20p13
213139_at	15	SNAI2	AI572079	8q11
209130_at	15	SNAP23	BC003686	15q14
214544_s_at	14	SNAP23	NM_003825	15q14
218098_at	15	SNAP23P	AL121903	20q13.13
218327_s_at	15	SNAP29	NM_004782	22q11.21
210465_s_at	14	SNAP50	U71300	9p22.2
205443_at	15	SNAPC1	NM_003082	14q22
204001_at	15	SNAPC3	NM_003084	9p22.2
213203_at	15	SNAPC5	AI633709	15q22.2
204466_s_at	15	SNCA	NM_000345	4q21
204467_s_at	15	SNCA	NM_000345	4q21
207827_x_at	15	SNCA	NM_007308	4q21
201622_at	15	SND1	NM_014390	7q31.3
218391_at	15	SNF8	NM_007241	17q21.33
218032_at	15	SNN	AF070673	16p13 /// 16p13
201221_s_at	15	SNRP70	NM_003089	19q13.3
201770_at	15	SNRPA	NM_004596	19q13.1
206055_s_at	15	SNRPA1	NM_003090	15q26.3
215722_s_at	15	SNRPA1	AJ130971	15q26.3
216977_x_at	14	SNRPA1	AJ130972	15q26.3
208821_at	15	SNRPB	J04564	20p13

213175_s_at	15	SNRPB	AL049650	20p13
202505_at	15	SNRPB2	NM_003092	20p12.2-p11.22
201342_at	15	SNRPC	NM_003093	6p21.31
202690_s_at	15	SNRPD1	BC001721	18q11.2
202691_at	14	SNRPD1	NM_006938	18q11.2
200826_at	15	SNRPD2	NM_004597	19q13.2
202567_at	15	SNRPD3	NM_004175	22q11.23
203316_s_at	15	SNRPE	NM_003094	1q32
215450_at	14	SNRPE	W87901	1q32
202886_s_at	12	SNRPEP1	M65254	11q23.2
203832_at	15	SNRPF	NM_003095	12q23.1
205644_s_at	15	SNRPG	NM_003096	2p13.3
201522_x_at	15	SNRPN	NM_003097	15q12
213447_at	15	SNRPN	AI672541	---
203516_at	15	SNTA1	NM_003098	20q11.2
205315_s_at	10	SNTB2	NM_006750	16q22-q23
206042_x_at	15	SNURF	NM_005678	15q12
201575_at	15	SNW1	NM_012245	14q24.3
210648_x_at	15	SNX 3A	AB047360	6q21
209860_s_at	15	SNX; ANX7	J04543	10q21.1-q21.2
201716_at	15	SNX1	NM_003099	15q22.1
213364_s_at	14	SNX1	AI052536	15q22.1
214531_s_at	12	SNX1	AF065484	15q22.1
53912_at	15	SNX11	AI668643	17q21.32
213292_s_at	15	SNX13	AA908770	7p21.1
205482_x_at	14	SNX15	NM_013306	11q12
219793_at	13	SNX16	NM_022133	8q21.13
202359_s_at	10	SNX19	NM_014758	11q25
202113_s_at	15	SNX2	AF043453	5q23
202114_at	15	SNX2	NM_003100	5q23
218705_s_at	13	SNX24	NM_014035	5q23.2
221006_s_at	11	SNX27	NM_030918	1q21.3 /// 1q21.3
200067_x_at	15	SNX3	AL078596	6q21
208781_x_at	15	SNX3	AL078596	6q21
213545_x_at	15	SNX3	BE962615	6q21
205329_s_at	15	SNX4	AF130078	3q21.2
212652_s_at	15	SNX4	AA524345	3q21.2
217792_at	15	SNX5	NM_014426	20p11
217789_at	14	SNX6	NM_021249	14q13.1
205573_s_at	15	SNX7	NM_015976	1p21.3
203372_s_at	15	SOCS2	NM_003877	12q
203373_at	15	SOCS2	NM_003877	12q
208127_s_at	15	SOCS5	NM_014011	2p21
209647_s_at	15	SOCS5	AW664421	2p21
209648_x_at	15	SOCS5	AL136896	2p21
206020_at	11	SOCS6	NM_016387	18q22.2
214462_at	11	SOCS6	NM_004232	18q22.2
200642_at	15	SOD1	NM_000454	21q22.1
215223_s_at	15	SOD2	W46388	6q25.3
204275_at	12	SOLH	AI796687	16p13.3

201086_x_at	15	SON	NM_003103	21q22.1-q22.2
213538_at	15	SON	AI936458	21q22.1-q22.2
207436_x_at	15	SORBS1	NM_014896	10q23.3-q24.1
218087_s_at	15	SORBS1	N21458	10q23.3-q24.1
204288_s_at	15	SORBS2	NM_021069	4q35.1
220858_at	12	SORBS2	NM_014133	4q35.1
201563_at	15	SORD1	L29008	15q15.3
212807_s_at	15	SORT1	BF447105	1p21.3-p13.1
212780_at	15	SOS1	AA700167	2p22-p21
211665_s_at	13	SOS2	L20686	14q21 /// 14q21
212870_at	15	SOS2	AI628605	14q22.1
213456_at	11	SOSTDC1	AI927000	7p21.2
204913_s_at	12	SOX11	AI360875	2p25
204914_s_at	13	sox11	AB028641	2p25
204915_s_at	14	sox11	AB028641	2p25
204432_at	13	SOX12	NM_006943	20p13
219993_at	15	SOX17	NM_022454	8q11.23
219568_x_at	15	SOX18	NM_018419	20q13.33
201416_at	15	SOX4	NM_003107	6p22.3
201417_at	15	SOX4	AL136179	6p22.3
201418_s_at	10	SOX4	NM_003107	6p22.3
202935_s_at	13	SOX9	AI382146	17q24.3-q25.1
202936_s_at	13	SOX9	NM_000346	17q24.3-q25.1
210195_s_at	12	SP1	M34715	19q13.2
202863_at	15	SP100	NM_003113	2q37.1
202864_s_at	15	SP100	NM_003113	2q37.1
208012_x_at	15	SP110	NM_004509	2q37.1
209761_s_at	15	SP110	AA969194	2q37.1
209762_x_at	15	SP110	AA969194	2q37.1
217877_s_at	15	SP192	AA741071	1p34.1
204367_at	13	SP2	D28588	17q21.32
213168_at	15	SP3	AU145005	2q31
205406_s_at	11	SPA17	NM_017425	11q24.2
219109_at	15	SPAG16	NM_024532	2q35
219888_at	11	SPAG4	NM_003116	20q11.21
203145_at	13	SPAG5	NM_006461	17q11.2
200053_at	15	SPAG7	NM_004890	17p13.2
212468_at	14	SPAG9	AK023512	17q21.33
212470_at	15	SPAG9	AB011088	17q21.33
200665_s_at	15	SPARC	NM_003118	5q31.3-q32
212667_at	15	SPARC	AL575922	5q31.3-q32
200795_at	15	SPARCL1	NM_004684	4q22.1
212526_at	15	SPARTIN	AK002207	13q13.2
207724_s_at	13	SPAST	NM_014946	2p24-p21
218164_at	14	SPATA20	NM_022827	17q21.33
218933_at	12	SPATA5L1	NM_024063	15q15.1
222163_s_at	13	SPATA5L1	BE890973	15q15.1
219583_s_at	14	SPATA7	NM_018418	14q31.3
218324_s_at	15	SPATS2	NM_023071	12q13.12
217927_at	15	SPCS1	NM_014041	3p21.31

201239_s_at	15	SPCS2	NM_014752	11q13.3
201240_s_at	15	SPCS2	NM_014752	11q13.3
218817_at	15	SPCS3	NM_021928	4q34.2
201996_s_at	11	SPEN	AL524033	1p36.33-p36.11
201997_s_at	15	SPEN	NM_015001	1p36.33-p36.11
210592_s_at	15	spermidine/spermine N1-acetyltransferase	M55580	Xp22.1
202441_at	15	SPFH1	AL568449	10q21-q22
216678_at	12	SPG	AK000773	3q21
215383_x_at	15	SPG21	AL137312	15q21-q22
217827_s_at	15	SPG21	NM_016630	15q21-q22
209748_at	11	SPG4	AB029006	2p24-p21
202104_s_at	13	SPG7	NM_003119	16q24.3
214494_s_at	15	SPG7	NM_005200	16q24.3 /// 16q24.3
206272_at	15	SPHAR	NM_006542	1q42.11-q42.3
219257_s_at	11	SPHK1	NM_021972	17q25.2
217813_s_at	14	SPIN	NM_006717	9q22.1-q22.3
211704_s_at	11	SPIN2	AF356353	Xp11.23-p11.21 /// Xp11.23-p11.21
32402_s_at	15	SPK; SYM	Y10931	19q13.3
202524_s_at	12	SPOCK2	AI952009	10pter-q25.3
209436_at	15	SPON1	AB018305	11p15.2
213994_s_at	13	SPON1	AI885290	11p15.2
218638_s_at	15	SPON2	NM_012445	4p16.3
204640_s_at	15	SPOP	NM_003563	17q21.33
208927_at	15	SPOP	BF673888	17q21.33
203458_at	11	SPR	AI951454	2p14-p12
212458_at	15	SPRED2	H97931	2p14
212558_at	15	SPRY1	BF508662	20q12
204011_at	15	SPRY2	NM_005842	13q22.2
221489_s_at	15	SPRY4	AF227517	5q31.3
206937_at	10	SPTA1	NM_003126	1q21
215235_at	15	SPTAN1	AL110273	9q33-q34
214145_s_at	10	SPTB	BG223341	14q23.3
200671_s_at	15	SPTBN1	N92501	2p21
200672_x_at	15	SPTBN1	NM_003128	2p21
207283_at	13	SPTBN1	NM_020217	2p21
212071_s_at	15	SPTBN1	BE968833	2p21
215918_s_at	15	SPTBN1	AA131826	2p21
202277_at	15	SPTLC1	AL568804	9q22.2
202278_s_at	10	SPTLC1	NM_006415	9q22.2
203127_s_at	13	SPTLC2	BC005123	14q24.3-q31
217995_at	13	SQRDL	NM_021199	15q15
201471_s_at	15	SQSTM1	NM_003900	5q35
212058_at	15	SR140	AI184562	3q23
212060_at	14	SR140	AU152088	3q23
212061_at	13	SR140	AB002330	3q23
209363_s_at	15	SRB7	U46837	12p12.1
204675_at	14	SRD5A1	NM_001047	5p15
201247_at	15	SREBF2	BE513151	22q13

201248_s_at	15	SREBF2	NM_004599	22q13
202401_s_at	15	SRF	NM_003131	6p21.1
208920_at	15	SRI	AV752215	7q21.1
208921_s_at	15	SRI	L12387	7q21.1
201516_at	14	SRM	NM_003132	1p36-p22
200007_at	15	SRP14	NM_003134	15q22
205335_s_at	15	SRP19	NM_003135	5q21-q22
213152_s_at	13	SRP46	AI343248	---
214789_x_at	15	SRP46	AA524274	11q22
203605_at	15	SRP54	NM_003136	14q13.2
208800_at	14	SRP72	AV702627	4q11
208801_at	15	SRP72	AV702627	4q11
208802_at	14	SRP72	AI493872	4q11
208803_s_at	15	SRP72	AV702627	4q11
201273_s_at	15	SRP9	NM_003133	1q42.13
202199_s_at	15	SRPK1	AW082913	6p21.3-p21.2
202200_s_at	15	SRPK1	NM_003137	6p21.3-p21.2
203181_x_at	14	SRPK2	AW149364	7q22-q31.1
203182_s_at	15	SRPK2	NM_003138	7q22-q31.1
214931_s_at	15	SRPK2	AC005070	7q22-q31.1
200918_s_at	15	SRPR	NM_003139	11q24.3
218140_x_at	15	SRPRB	NM_021203	3q22.1
204955_at	15	SRPX	NM_006307	Xp21.1
205499_at	12	SRPX2	NM_014467	Xq21.33-q23
219204_s_at	12	SRR	NM_021947	17p13
219205_at	14	SRR	NM_021947	17p13
201225_s_at	15	SRRM1	NM_005839	1p36.11
207435_s_at	15	SRRM2	NM_016333	16p13.3
208610_s_at	15	SRRM2	AI655799	16p13.3
216833_x_at	13	SS	U05255	4q31.1
202816_s_at	15	SS18	AW292882	18q11.2
202817_s_at	14	SS18	NM_005637	18q11.2
213140_s_at	15	SS18L1	AB014593	20q13.3
218283_at	15	SS18L2	NM_016305	3p21
210438_x_at	15	SSA2	M25077	1q31
212839_s_at	12	SSA2	AK024044	1q31
212852_s_at	15	SSA2	AL538601	1q31
213027_at	15	SSA2	AU146655	1q31
201138_s_at	15	SSB	NM_003142	2q31.1
201139_s_at	15	SSB	NM_003142	2q31.1
46256_at	15	SSB3	AA522670	---
202591_s_at	15	SSBP1	NM_003143	7q34
214060_at	14	SSBP1	BE220360	7q34
203787_at	15	SSBP2	NM_012446	5q14.1
217991_x_at	14	SSBP3	NM_018070	1p32.3
202506_at	15	SSFA2	NM_006751	2q32.1
221752_at	15	SSH1	AI651213	12q24.12
221753_at	15	SSH1	AI651213	12q24.12
210378_s_at	15	SSNA1	BC004118	9q34.3
202318_s_at	15	SSP1	AF306508	6q13-q14.3

214790_at	14	SSP1	AK001406	6q13-q14.3
204963_at	15	SSPN	AL136756	12p11.2
204964_s_at	13	SSPN	AL136756	12p11.2
200889_s_at	15	SSR1	NM_003144	6p24.3
200890_s_at	14	SSR1	NM_003144	6p24.3
200891_s_at	15	SSR1	NM_003144	6p24.3
200652_at	15	SSR2	NM_003145	1q21-q23
217790_s_at	12	SSR3	NM_007107	3q25.31
201004_at	15	SSR4	NM_006280	Xq28
200956_s_at	15	SSRP1	BE795648	11q12
200957_s_at	15	SSRP1	NM_003146	11q12
203015_s_at	15	SSX2IP	AW136988	---
203016_s_at	12	SSX2IP	NM_014021	---
203017_s_at	12	SSX2IP	R52678	---
207040_s_at	15	ST13	NM_003932	22q13.2
208666_s_at	15	ST13	BE866412	22q13.2
208667_s_at	15	ST13	U17714	22q13.2
210942_s_at	15	ST3Gal VI	AB022918	3q12.2
203759_at	15	ST3GAL4	NM_006278	11q23-q24
203217_s_at	15	ST3GAL5	NM_003896	2p11.2
202440_s_at	14	ST5	NM_005418	11p15
220937_s_at	15	ST6GALNAC4	NM_014403	9q34
207524_at	15	ST7	NM_021908	7q31.1-q31.3
207871_s_at	15	ST7	NM_018412	7q31.1-q31.3
38487_at	15	STAB1	D87433	3p21.31
201836_s_at	15	STAF65(gamma)	AU154740	2pter-p25.1
202294_at	15	STAG1	NM_005862	3q21-q22
207983_s_at	15	STAG2	NM_006603	Xq25
209022_at	15	STAG2	AK026678	Xq25
209023_s_at	15	STAG2	BC001765	Xq25
203544_s_at	15	STAM	NM_003473	10p14-p13
215044_s_at	14	STAM2	AI571996	2q24.1
202811_at	15	STAMBP	NM_006463	2p13.1
213103_at	14	STARD13	AA128023	13q12-q13
202991_at	10	STARD3	NM_006804	17q11-q12
200028_s_at	15	STARD7	NM_020151	2q11.2
206868_at	13	STARD8	NM_014725	Xq13.1
200887_s_at	15	STAT1	NM_007315	2q32.2
205170_at	13	STAT2	NM_005419	12q13.13
208991_at	15	STAT3	BC000627	17q21
206118_at	15	STAT4	NM_003151	2q32.2-q32.3
205026_at	13	STAT5B	NM_012448	17q11.2
212549_at	15	STAT5B	BE645861	17q11.2
212550_at	12	STAT5B	AI149535	17q11.2
201331_s_at	15	STAT6B	BC004973	12q13
208948_s_at	15	STAU	BC000830	20q13.1
211505_s_at	15	STAU	AL136601	20q13.1
207320_x_at	15	STAU1	NM_004602	20q13.1
204226_at	15	STAU2	NM_014393	8q13-q21.1
213037_x_at	15	staufen	AJ132258	20q13.1

204597_x_at	11	STC1	NM_003155	8p21-p11.2
203438_at	13	STC2	AI435828	5q35.2
203439_s_at	15	STC2	BC000658	5q35.2
202557_at	15	STCH	AI718418	21q11.1
202558_s_at	14	STCH	NM_006948	21q11.1
219934_s_at	15	STE	U55764	4q13.1
205339_at	13	STIL	NM_003035	1q32
202764_at	12	STIM1	NM_003156	11p15.5
213330_s_at	13	STIP1	BE886580	11q13
40420_at	15	STK10	AB015718	5q35.1
202693_s_at	14	STK17A	NM_004760	7p12-p14
206216_at	11	STK23	NM_014370	Xq28
208854_s_at	15	STK24	AA586774	13q31.2-q32.3
201314_at	15	STK25	NM_006374	2q37.3
204068_at	15	STK3	NM_006281	8q22.2
208855_s_at	15	STK3	AF083420	13q31.2-q32.3
202951_at	15	STK38	BE048506	6p21
212565_at	15	STK38L	BE302191	12p12.1
212572_at	15	STK38L	AW779556	12p12.1
202786_at	15	STK39	NM_013233	2q31.1
204092_s_at	13	STK6	NM_003600	20q13.2-q13.3
208079_s_at	15	STK6	NM_003158	20q13.2-q13.3 /// 20q13.2-q13.3
209607_x_at	15	STM	U08032	16p12.1
210580_x_at	15	STM	L25275	16p11.2
200783_s_at	15	STMN1	NM_005563	1p36.1-p35
217714_x_at	15	STMN1	AV756729	1p36.1-p35
203001_s_at	11	STMN2	NM_007029	8q21.11-q21.12
201060_x_at	15	STOM	AI537887	9q34.1
210825_s_at	15	STOM	AF130103	9q34.1 /// 9q34.1
201061_s_at	15	stomatin peptide	M81635	9q34.1
215416_s_at	15	STOML2	AC004472	9p13.1
215299_x_at	14	STP1	U37025	---
211385_x_at	14	STP2	U28169	16p12.1
200870_at	15	STRAP	NM_007178	12p13.1
204496_at	14	STRN3	NM_014574	14q13-q21
217903_at	15	STRN4	NM_013403	19q13.2
217934_x_at	15	STUB1	NM_005861	16p13.3
212625_at	15	STX10	NM_003765	19p13.13
212111_at	15	STX12	AA628051	1p35-p34.1
212112_s_at	15	STX12	AI816243	1p35-p34.1
218763_at	15	STX18	NM_016930	4p16.2
209238_at	15	STX3A	BE966922	11q12.2
203530_s_at	15	STX4A	NM_004604	16p11.2
203330_s_at	13	STX5A	NM_003164	11q12.3
212799_at	15	STX6	AI740832	1q25.1
212800_at	10	STX6	AI740832	1q25.1
203457_at	15	STX7	NM_003569	6q23.1
212631_at	15	STX7	AI566082	---
212632_at	15	STX7	N32035	---

204690_at	15	STX8	NM_004853	17p12
202260_s_at	15	STXBP1	NM_003165	9q34.1
203310_at	14	STXBP3	NM_007269	1p13.3
220994_s_at	14	STXBP6	NM_014178	14q11.2
218321_x_at	15	STYXL1	NM_016086	7q11.23
214512_s_at	15	SUB1	NM_006713	5p13.3
202930_s_at	15	SUCLA2	NM_003850	13q12.2-q13.3
217874_at	15	SUCLG1	NM_003849	2p11.2
212459_x_at	15	SUCLG2	BF593940	3p14.3
215772_x_at	15	SUCLG2	AV650470	3p14.3
221213_s_at	15	SUHW4	NM_017661	15q21.2
211956_s_at	15	SUI1	BF246436	17q21.31
212130_x_at	15	SUI1	AL537707	17q21.31
212227_x_at	15	SUI1	W67644	17q21.31
212344_at	13	SULF1	AW043713	8q13.2
212353_at	15	SULF1	AI479175	8q13.2
212354_at	15	SULF1	AI479175	8q13.2
203615_x_at	15	SULT1A1	NM_001055	16p12.1
207122_x_at	15	SULT1A2	NM_001054	16p12.1
205812_s_at	15	SULT1C2	NM_006588	2q11.1-q11.2
211069_s_at	15	SUMO1	BC006462	2q33 /// 2q33
213879_at	15	SUMO2	AV726646	17q25
213881_x_at	15	SUMO2	AI971724	17q25
215452_x_at	15	SUMO2	AL031133	---
200739_s_at	15	SUMO3	BG338532	21q22.3
200740_s_at	15	SUMO3	BG338532	21q22.3
217168_s_at	15	SUP	AF217990	16q12.2-q13
217815_at	15	SUPT16H	NM_007192	14q11.2
206506_s_at	15	SUPT3H	NM_003599	6p21.1-p21.3
211106_at	13	SUPT3H	AF064804	6p21.1-p21.3
201483_s_at	15	SUPT4H1	BC002802	17q21-q23
201484_at	15	SUPT4H1	NM_003168	17q21-q23
201480_s_at	15	SUPT5H	NM_003169	19q13
208420_x_at	14	SUPT6H	NM_003170	17q11.2
208830_s_at	13	SUPT6H	AI494567	17q11.2
208831_x_at	10	SUPT6H	D79984	17q11.2
212894_at	13	SUPV3L1	NM_003171	10q22.1
209362_at	15	SURB7	AI688580	12p12.1
204295_at	15	SURF1	NM_003172	9q34.2
206593_s_at	15	SURF5	NM_006752	9q34.2
218242_s_at	15	SUV420H1	AA056099	11q13.2
212287_at	15	SUZ12	BF382924	17q11.2
216086_at	15	SV2C	AB028977	5q13.3
202565_s_at	15	SVIL	NM_003174	10p11.2
202566_s_at	13	SVIL	AF051851	10p11.2
209898_x_at	15	SWA	U61167	2pter-p25.1
209306_s_at	14	SWAP70	BC000616	11p15
209307_at	15	SWAP70	AB014540	11p15
215294_s_at	15	SWI2	AK026426	Xq25
201296_s_at	15	SWIP1; WSB-1	AF106684	17q11.2

202829_s_at	15	SYBL1	NM_005638	Xq28
202553_s_at	15	SYF2	NM_015484	1p36.13-p35.1
212730_at	15	SYN; KIAA0353	AK026420	15q26.3
221499_s_at	15	SYN16	AK026970	20q13.32
221500_s_at	15	SYN16	AK026970	20q13.32
210247_at	11	SYN2	AW139618	3p25
221276_s_at	12	SYNC1	NM_030786	1p34.3-p33 /// 1p34.3-p33
209024_s_at	13	SYNCRIP	AI472757	6q14-q15
217832_at	15	SYNCRIP	AL520908	6q14-q15
217833_at	15	SYNCRIP	AL520908	6q14-q15
217834_s_at	14	SYNCRIP	AL520908	6q14-q15
202761_s_at	15	SYNE2	NM_015180	14q23.2
210613_s_at	15	SYNGR1	BC000731	22q13.1
201079_at	15	SYNGR2	NM_004710	17q25.3
212990_at	15	SYNJ1	AB020717	21q22.2
212828_at	13	SYNJ2	AA191573	6q25.3
219156_at	15	SYNJ2BP	NM_018373	14q24.1
202796_at	15	SYNPO	NM_007286	5q33.1
219804_at	15	SYNPO2L	NM_024875	10q22.3
201259_s_at	15	SYPL	AI768845	7q22.2
201260_s_at	15	SYPL1	NM_006754	7q22.2
203999_at	13	SYT1	AV731490	12cen-q21
209197_at	15	SYT11	AA626780	1q21.2
209198_s_at	14	SYT11	BC004291	1q21.2
56829_at	13	T1	H61826	8q24.3
221898_at	11	T1A-2	AU154455	1p36
200976_s_at	15	T6BP; TXBP151	AF090891	7p15
200977_s_at	15	T6BP; TXBP151	AF090891	7p15
200911_s_at	15	TACC1	NM_006283	8p11
202289_s_at	15	TACC2	NM_006997	10q26
218308_at	11	TACC3	NM_006342	4p16.3
201839_s_at	12	TACSTD1	NM_002354	2p21
200055_at	15	TAF10	NM_006284	11p15.3
202840_at	15	TAF15	NM_003487	17q11.1-q11.2
206613_s_at	12	TAF1A	NM_005681	1q42
203938_s_at	10	TAF1C	NM_005679	16q24
209523_at	15	TAF2B	AK001618	8q24.12
209358_at	15	TAF2I	AF118094	6p21.31
209463_s_at	11	TAF2J; TAFII20	D50544	1p35.2
213090_s_at	14	TAF4	AI744029	20q13.33
203572_s_at	15	TAF6	NM_005641	7q22.1
205955_at	11	TAF6L	NM_018336	11q12.3
213209_at	12	TAF6L	BF058726	11q12.3
201023_at	15	TAF7	NM_005642	5q31
202168_at	15	TAF9	NM_003187	5q11.2-q13.1
203893_at	15	TAF9	NM_016283	5q11.2-q13.1
209430_at	15	TAFII170	AJ001017	10q22-q23
205547_s_at	15	TAGLN	NM_003186	11q23.2
200916_at	15	TAGLN2	NM_003564	1q21-q25

210978_s_at	14	TAGLN2	BC002616	1q21-q25
211537_x_at	14	TAK1	AF218074	6q16.1-q16.3
211536_x_at	14	TAK1; TGF1a	AB009358	6q16.1-q16.3
206283_s_at	15	TAL1	NM_003189	1p32
201463_s_at	15	TALDO1	NM_006755	11p15.5-p15.4
207616_s_at	15	TANK	NM_004180	2q24-q31
220761_s_at	15	TAOK3	NM_016281	12q
221508_at	15	TAOK3	BC002756	12q
210276_s_at	15	TARA	AF281030	22q13.1
202813_at	15	TARBP1	NM_005646	1q42.3
203677_s_at	13	TARBP2	NM_004178	12q12-q13
211666_x_at	15	TARBP-b	L22453	22q13 /// 22q13
200020_at	15	TARDBP	NM_007375	1p36.22
221264_s_at	14	TARDBP	NM_031214	1p36.22 /// 1p36.22
201263_at	15	TARS	NM_003191	5p13.2 /// 5p13.2
221847_at	15	TAS2R14	BF665706	12p13
219443_at	14	TASP1	NM_017714	20p12.1
203648_at	14	TATDN2	NM_014760	3p25.3
212833_at	15	TB1	M74089	5q22.2
212350_at	15	TBC1D1	AB029031	4p14
220947_s_at	15	TBC1D10B	NM_015527	16p12.1
221858_at	15	TBC1D12	N34407	10q23.33
44696_at	15	TBC1D13	AA915989	9q34.13
218268_at	15	TBC1D15	NM_022771	12q15
218466_at	13	TBC1D17	NM_024682	19q13.33
209403_at	13	TBC1D3	AL136860	17q21.1
203386_at	15	TBC1D4	AI650848	13q21.33
203387_s_at	15	TBC1D4	NM_014832	13q21.33
201814_at	14	TBC1D5	NM_014744	3p24.3
204526_s_at	15	TBC1D8	NM_007063	2q12.1
203667_at	15	TBCA	NM_004607	5q14.1
216683_at	10	TBCA	AL353949	---
202495_at	13	TBCC	NM_003192	6pter-p12.1
201759_at	13	TBCD	AW247323	17q25.3
211052_s_at	14	TBCD	BC006364	17q25.3 /// 17q25.3
203714_s_at	15	TBCE	NM_003193	1q42.3
203715_at	15	TBCE	NM_003193	1q42.3
218520_at	15	TBK1	NM_013254	13q14.2-q14.3
221428_s_at	14	TBL1XR1	NM_030921	3q26.33 /// 3q26.33
212685_s_at	15	TBL2	AI608789	7q11.23
203135_at	15	TBP	NM_003194	6q27
208398_s_at	15	TBPL1	NM_004865	6q22.1-q22.3
205993_s_at	10	TBX2	NM_005994	17q23
40560_at	15	TBX2	U28049	17q23
207155_at	11	TBX5	NM_000192	12q24.1
211886_s_at	11	TBX5	U80987	12q24.1
207554_x_at	12	TBXA2R	NM_001060	19p13.3
209681_at	15	TC1	AF153330	1q23.3
200750_s_at	15	TC4; Gsp1; ARA24	AF054183	6p21
205766_at	15	TCAP	NM_003673	17q12

203919_at	15	TCEA2	NM_003195	20q13.33
204045_at	15	TCEAL1	NM_004780	Xq22.1
202371_at	15	TCEAL4	NM_024863	Xq22.2
202823_at	15	TCEB1	N89607	8q13.3
202824_s_at	15	TCEB1	NM_005648	8q13.3
200085_s_at	15	TCEB2	NM_007108	16p12.3
202818_s_at	14	TCEB3	AI344128	1p36.1
213604_at	14	TCEB3	AW451236	1p36.1
202396_at	15	TCERG1	NM_006706	5q31
208986_at	15	TCF12	AL559478	15q21
212931_at	13	TCF20	AB006630	22q13.3
215511_at	14	TCF20	U19345	22q13.3
204931_at	15	TCF21	NM_003206	6pter-qter
209152_s_at	12	TCF3	M31523	19p13.3
209153_s_at	15	TCF3	M31523	19p13.3
210776_x_at	14	TCF3	M31222	19p13.3
213730_x_at	14	TCF3	BE962186	19p13.3
213811_x_at	15	TCF3	BG393795	19p13.3
203753_at	15	TCF4	NM_003199	18q21.1
212382_at	15	TCF4	BF433429	18q21.1
212385_at	13	TCF4	AU118026	18q21.1
212386_at	15	TCF4	BF592782	18q21.1
212387_at	15	TCF4	BG495771	18q21.1
213891_s_at	15	TCF4	AI927067	18q21.1
205255_x_at	15	TCF7	NM_003202	5q31.1
221016_s_at	15	TCF7L1	NM_031283	2p11.2 /// 2p11.2
212761_at	13	TCF7L2	AI703074	10q25.3
212762_s_at	15	TCF7L2	AI375916	10q25.3
216035_x_at	12	TCF7L2	AV721430	10q25.3
216037_x_at	15	TCF7L2	AA664011	10q25.3
216511_s_at	15	TCF7L2	AJ270770	---
208078_s_at	15	TCF8	NM_030751	10p11.2 /// 10p11.2
212764_at	15	TCF8	AI373166	10p11.2
213708_s_at	15	TCFL4	N40555	17q12-q21
204849_at	13	TCFL5	NM_006602	20q13.3-qter
202384_s_at	12	TCOF1	AW167713	5q32-q33.1
208778_s_at	15	TCP1	BC000665	6q25-q27
203054_s_at	14	TCTA	NM_022171	3p21
208671_at	15	TDE2	AF164794	6q22.32
203742_s_at	10	TDG	NM_003211	12q24.1
203743_s_at	15	TDG	NM_003211	12q24.1
208089_s_at	14	TDRD3	NM_030794	13q21.1 /// 13q21.1
214028_x_at	13	TDRD3	AU156998	13q21.1
213361_at	13	TDRD7	AW129593	9q22.33
214600_at	13	TEAD1	AW771935	11p15.4
209454_s_at	15	TEF5	AF142482	6p21.2
200803_s_at	15	TEGT	NM_003217	12q12-q13
200804_at	15	TEGT	NM_003217	12q12-q13
206702_at	15	TEK	NM_000459	9p21
212494_at	15	TENC1	AB028998	12q13.13

204053_x_at	15	TEP1	U96180	10q23.3
220147_s_at	15	TERA	NM_021238	12p11
203448_s_at	15	TERF1	AI347136	8q13
203449_s_at	15	TERF1	NM_017489	8q13
203611_at	15	TERF2	NM_005652	16q22.1
201174_s_at	15	TERF2IP	NM_018975	16q23.1
202719_s_at	12	TES	NM_015641	7q31.2
202720_at	15	TES	NM_015641	7q31.2
204106_at	15	TESK1	NM_006285	9p13
209215_at	15	TETRAN; TETTRAN	L11669	4p16.3
218104_at	15	TEX10	NM_017746	9q31.1
218099_at	15	TEX2	NM_018469	17q24.2
212083_at	15	TEX261	AV759552	2p13.2
218548_x_at	14	TEX264	NM_015926	3p21.31
215758_x_at	15	TF34	AC007204	19p13.11
203176_s_at	13	TFAM	NM_003201	10q21
203177_x_at	15	TFAM	NM_003201	10q21
208541_x_at	14	TFAM	NM_012251	10q21
219169_s_at	15	TFB1M	NM_016020	6q25.1-q25.3
218605_at	15	TFB2M	NM_022366	1q44
207627_s_at	10	TFCP2	NM_005653	12q13
204147_s_at	12	TFDP1	NM_007111	13q34
212330_at	15	TFDP1	R60866	13q34
203588_s_at	15	TFDP2	BG034328	3q23
221866_at	14	TFEB	AL035588	6p21
50221_at	15	TFEB	AI524138	6p21
204623_at	14	TFF3	NM_003226	21q22.3
217839_at	15	TFG	NM_006070	3q11-q12
35671_at	15	TFIIIC	U02619	16p12
216241_s_at	15	TFIIS	X57198	3p22-p21.3
209676_at	14	TFPI	J03225	2q31-q32.1
213258_at	15	TFPI	BF511231	2q31-q32.1
209277_at	10	TFPI2	AL574096	7q22
210664_s_at	15	TFPIbeta	AF021834	2q31-q32.1
210665_at	14	TFPIbeta	AF021834	2q31-q32.1
218996_at	13	TFPT	NM_013342	19q13
207332_s_at	15	TFRC	NM_003234	3q26.2-qter
208691_at	15	TFRC	BC001188	3q26.2-qter
211573_x_at	13	TG2; TGC	M98478	20q12
208249_s_at	15	TGDS	NM_014305	13q32.1
205016_at	13	TGFA	NM_003236	2p13
209651_at	15	TGFB1I1	BC001830	16p11.2
209747_at	11	TGFB3	J03241	14q24
209591_s_at	15	tgf-beta	M60316	20q13
201506_at	15	TGFBI	NM_000358	5q31
208944_at	15	TGFBR2	D50683	3p22 /// 3p22
204731_at	15	TGFBR3	NM_003243	1p33-p32
221235_s_at	14	TGFBRAP1	NM_030825	---
203313_s_at	15	TGIF	NM_003244	18p11.3
216262_s_at	15	TGIF2	AL050318	20q11.2-q12

218724_s_at	14	TGIF2	AL050318	20q11.2-q12
201042_at	15	TGM2	AL031651	---
203833_s_at	15	TGOLN2	BF061845	2p11.2
212040_at	15	TGOLN2	BG249599	2p11.2
212043_at	15	TGOLN2	W72053	2p11.2
220607_x_at	15	TH1L	NM_016397	20q13
54632_at	14	THADA	AI286226	2p22.1
219596_at	14	THAP10	NM_020147	15q22.32
212910_at	15	THAP11	W19873	16q22.1
220417_s_at	15	THAP4	NM_015963	2q37.3
218492_s_at	15	THAP7	NM_030573	22q11.2
203887_s_at	15	THBD	NM_000361	20p12-cen
203888_at	13	THBD	NM_000361	20p12-cen
201110_s_at	11	THBS1	NM_003246	15q15
203083_at	15	THBS2	NM_003247	6q27
204776_at	15	THBS4	NM_003248	5q13
204565_at	15	THEM2	NM_018473	6p22.1
222122_s_at	15	THO2	AK001758	Xq25-q26.3
204064_at	15	THOC1	NM_005131	18p11.32
212994_at	14	THOC2	BE543527	Xq25-q26.3
203235_at	12	THOP1	NM_003249	19q13.3
1316_at	13	THRA	X55005	17q11.2
201987_at	15	THRAP1	AI984051	17q22-q23
212207_at	10	THRAP2	BG426689	12q24.22
213043_s_at	15	THRAP4	AI023317	17q21.2
221938_x_at	14	THRAP5	AW262690	19p13.3
43544_at	15	THRAP5	AA314406	19p13.3
219477_s_at	14	THSD1	NM_018676	13q14.2
218540_at	15	THTPA	NM_024328	---
206555_s_at	13	THUMPD1	NM_017736	16p13.11
213025_at	15	THUMPD1	AL134904	16p13.11
219248_at	15	THUMPD2	NM_025264	2p22.2
217744_s_at	15	THW	AJ251830	6q24
208850_s_at	15	THY1	AL558479	11q22.3-q23
208851_s_at	14	THY1	AL161958	11q22.3-q23
213869_x_at	15	THY1	AA218868	11q22.3-q23
218491_s_at	15	THYN1	NM_014174	11q25
201446_s_at	15	TIA1	BF692742	2p13
201447_at	15	TIA1	AL567227	2p13
201448_at	15	TIA1	AL567227	2p13
201449_at	15	TIA1	AL567227	2p13
201450_s_at	15	TIA1	NM_022037	2p13
217052_x_at	11	TIA1	AK024108	2p13
217164_at	15	TIA1	AK024108	2p13
202039_at	15	TIAF1	NM_004740	17q11.2
202405_at	15	TIAL1	BF432532	---
202406_s_at	15	TIAL1	NM_003252	10q
213135_at	14	TIAM1	U90902	21q22.1
202363_at	15	TIC1	AF231124	5q31
204468_s_at	15	TIE1	NM_005424	1p34-p33

204391_x_at	15	TIF1	AF009353	7q32-q34
213301_x_at	15	TIF1	AL538264	7q32-q34
222204_s_at	14	TIFIA	AL110238	16p12
215171_s_at	15	TIM17; TIM17A	AK023063	1q32.1
203046_s_at	15	TIMELESS	NM_003920	12q12-q13
218408_at	15	TIMM10	NM_012456	11q12.1-q12.3
218188_s_at	15	TIMM13	NM_012458	19p13.3
201821_s_at	15	TIMM17A	NM_006335	1q32.1
201822_at	10	TIMM17A	NM_006335	1q32.1
203342_at	15	TIMM17B	NM_005834	Xp11.23
218118_s_at	15	TIMM23	NM_006327	10q11.21-q11.23
218119_at	15	TIMM23	NM_006327	10q11.21-q11.23
203092_at	14	TIMM44	AF026030	19p13.3-p13.2
203093_s_at	15	TIMM44	NM_006351	19p13.3-p13.2
205217_at	12	TIMM8A	NM_004085	Xq22.1
218357_s_at	15	TIMM8B	NM_012459	11q23.1-q23.2
218316_at	15	TIMM9	NM_012460	14q21
201666_at	15	TIMP1	NM_003254	Xp11.3-p11.23
203167_at	10	TIMP2	NM_003255	17q25
201147_s_at	15	TIMP3	NM_000362	22q12.1-q13.2
201148_s_at	15	TIMP3	AW338933	22q12.1-q13.2
201150_s_at	15	TIMP3	NM_000362	22q12.1-q13.2
219058_x_at	11	TINAGL1	NM_022164	1p35.1
220052_s_at	12	TINF2	NM_012461	14q11.2
201922_at	15	TINP1	NM_014886	5q13.3
216248_s_at	11	TINUR	S77154	2q22-q23
209154_at	15	TIP-1	AF234997	17p13
215464_s_at	15	TIP-1	AK001327	17p13
208838_at	14	TIP120A	AB020636	12q14
208839_s_at	15	TIP120A	AL136810	12q14
213547_at	15	TIP120B	AB014567	3p25.2
212665_at	15	TIPARP	AL556438	3q25.31
217367_s_at	11	TIX1	AB007855	20q12
217547_x_at	11	TIZ	BF308250	19p12
202011_at	15	TJP1	NM_003257	15q13
214168_s_at	15	TJP1	AA813018	15q13
202085_at	15	TJP2	NM_004817	9q13-q21
47608_at	15	TJP4	AI697401	6p21.1
208700_s_at	15	tk	L12711	3p14.3
204276_at	14	TK2	BE895437	16q22-q23.1
214183_s_at	10	TKR; TKT2	X91817	Xq28
208699_x_at	12	TKT	BF696840	3p14.3
203221_at	15	TLE1	NM_005077	9q21.32
40837_at	15	TLE2	M99436	19p13.3
206472_s_at	11	TLE3	NM_005078	15q22
204872_at	15	TLE4	NM_007005	9q21.32
202606_s_at	15	TLK1	NM_012290	2q31.1
212997_s_at	15	TLK2	AU151689	17q23
215843_s_at	15	TLL2	AK026106	10q23-q24
203254_s_at	13	TLN1	NM_006289	9p13

212701_at	11	TLN2	AB002318	15q15-q21
212703_at	12	TLN2	AV728958	15q15-q21
209386_at	15	TM4SF1	AI346835	3q21-q25
215034_s_at	15	TM4SF1	AI189753	3q21-q25
209655_s_at	15	TM4SF10	AL136550	Xp11.4
209656_s_at	15	TM4SF10	AL136550	Xp11.4
209525_at	10	TM6SF1	BG285017	15q24-q26
219892_at	15	TM6SF1	NM_023003	15q24-q26
204137_at	15	TM7SF1	NM_003272	1q42-q43
210130_s_at	11	TM7SF2	AF096304	11q13
201078_at	15	TM9SF2	NM_004800	13q32.3
212194_s_at	15	TM9SF4	AI418892	20q11.21
212198_s_at	13	TM9SF4	AL515964	20q11.21
217730_at	15	TMBIM1	NM_022152	2p24.3-p24.1
219206_x_at	15	TMBIM4	NM_016056	12q14.1-q15
204328_at	15	TMC6	NM_007267	17q25.3
213349_at	14	TMCC1	AI934469	3q21.3
213096_at	12	TMCC2	T51252	1q32.1
220240_s_at	13	TMCO3	NM_017905	13q34
203679_at	15	TMED1	NM_006858	19p13.2
200929_at	15	TMED10	NM_006827	14q24.3
204426_at	13	TMED2	NM_006815	12q24.31
204427_s_at	15	TMED2	NM_006815	12q24.31
208837_at	15	TMED3	BC000027	15q24-q25
214658_at	14	TMED7	BG286537	5q23.1
208757_at	15	TMED9	BC001123	5q35.3
205122_at	14	TMEFF1	BF439316	9q31
219230_at	15	TMEM100	NM_018286	17q23.1
201764_at	15	TMEM106C	NM_024056	12q13.1
201361_at	15	TMEM109	NM_024092	11q12.2
203437_at	15	TMEM11	NM_003876	17p11.2
217882_at	15	TMEM111	NM_018447	3p25.3
218477_at	15	TMEM14A	NM_014051	6p12.3
221452_s_at	15	TMEM14B	NM_030969	6p25.1-p23
218804_at	15	TMEM16A	NM_018043	11q13.2
218910_at	10	TMEM16K	NM_018075	3p21.32
212989_at	15	TMEM23	AI377497	10q11.2
217743_s_at	15	TMEM30A	NM_018247	6q14.1
213285_at	10	TMEM30B	AV691491	14q23.1
218465_at	15	TMEM33	NM_018126	4p14
218772_x_at	15	TMEM38B	NM_018112	9q31.3
218615_s_at	15	TMEM39A	AA194996	3q13.33
218770_s_at	14	TMEM39B	NM_018056	1p34.3
202857_at	15	TMEM4	NM_014255	12q15
209796_s_at	15	TMEM4	BC001027	12q15
209797_at	15	TMEM4	BC001027	12q15
219503_s_at	10	TMEM40	AI087937	3p25.2
217795_s_at	15	TMEM43	NM_024334	3p25.1
219410_at	15	TMEM45A	NM_018004	3q12.3
218073_s_at	12	TMEM48	NM_018087	1p32.3

220990_s_at	15	TMEM49	NM_030938	17q23.2
204807_at	12	TMEM5	BF224146	12q14.1
204808_s_at	15	TMEM5	NM_014254	12q14.1
217766_s_at	15	TMEM50A	NM_014313	1p36.11
219600_s_at	15	TMEM50B	BC000569	21q22.11
200620_at	15	TMEM59	NM_004872	1p36-p31
218776_s_at	14	TMEM62	NM_024956	15q15.1
202700_s_at	12	TMEM63A	NM_014698	1q42.13
200847_s_at	15	TMEM66	NM_016127	8p12
219449_s_at	15	TMEM70	BC002748	8q13.3
221882_s_at	15	TMEM8	AI636233	16p13.3
221255_s_at	15	TMEM93	NM_031298	17p13.3
218065_s_at	15	TMEM9B	NM_020644	11p15.3
213024_at	14	TMF1	BF593908	3p21-p12
214948_s_at	15	TMF1	AL050136	---
203661_s_at	15	TMOD1	NM_003275	9q22.3
203662_s_at	15	TMOD1	NM_003275	9q22.3
213161_at	15	TMOD1	AI583393	---
212352_s_at	15	TMP21	BE780075	14q24.3
203432_at	15	TMPO	AW272611	12q22
210986_s_at	15	TMSA	Z24727	15q22.1
211921_x_at	15	TMSA; MGC104802	AF348514	--- /// ---
217733_s_at	15	TMSB10	NM_021103	2p11.2
216438_s_at	15	TMSB4X	AL133228	---
205347_s_at	15	TMSL8	NM_021992	Xq21.33-q22.3
201645_at	12	TNC	NM_002160	9q33
209904_at	15	TNC; TNNC	AF020769	3p21.3-p14.3
201207_at	15	TNFAIP1	NM_021137	17q22-q23
202510_s_at	15	TNFAIP2	NM_006291	14q32
202643_s_at	11	TNFAIP3	AI738896	6q23
202644_s_at	15	TNFAIP3	NM_006290	6q23
206025_s_at	15	TNFAIP6	AW188198	2q24.1
206026_s_at	15	TNFAIP6	NM_007115	2q24.1
208296_x_at	15	TNFAIP8	NM_014350	5q23.1
210260_s_at	15	TNFAIP8	BC005352	5q23.1
218368_s_at	14	TNFRSF12A	NM_016639	16p13.3
207643_s_at	15	TNFRSF1A	NM_001065	12p13.2
203508_at	14	TNFRSF1B	NM_001066	1p36.3-p36.2
218856_at	10	TNFRSF21	NM_016629	6p21.1-12.2
202687_s_at	10	TNFSF10	NM_003810	3q26
202688_at	15	TNFSF10	NM_003810	3q26
214329_x_at	13	TNFSF10	AW474434	3q26
218467_at	15	TNFSF5IP1	NM_020232	18p11.21
206907_at	11	TNFSF9	NM_003811	19p13.3
211828_s_at	11	TNIK	AF172268	3q26.31
213107_at	15	TNIK	R59093	3q26.31
213109_at	14	TNIK	N25621	3q26.31
207196_s_at	15	TNIP1	NM_006058	5q32-q33.1
48531_at	15	TNIP2	AA522816	4p16.3
217149_x_at	14	TNK1	AF097738	---

203839_s_at	14	TNK2	AI146308	3q29
202561_at	15	TNKS	AF070613	8p23.1
218228_s_at	15	TNKS2	BF060683	10q23.3
205177_at	15	TNNI1	NM_003281	1q31.3
205742_at	15	TNNI3	NM_000363	19q13.4
220415_at	15	TNNI3K	NM_015978	1p31.1
215389_s_at	15	TNNT2	X79857	1q32
205693_at	12	TNNT3	NM_006757	11p15.5
207657_x_at	15	TNPO1	NM_002270	5q13.2
209225_x_at	14	TNPO1	AI653355	5q13.2
212635_at	15	TNPO1	AW161626	5q13.2
221829_s_at	15	TNPO1	AI307759	5q13.2
221506_s_at	15	TNPO2	BG258639	19p13.2
221507_at	15	TNPO2	BG258639	19p13.2
212318_at	15	TNPO3	AK022910	7q32.3
211342_x_at	15	TNRC11	BC004354	---
212261_at	15	TNRC15	AB014542	2q37.1
217931_at	12	TNRC5	BC004423	6pter-p12.1
213254_at	13	TNRC6B	N64803	22q13.2
221747_at	15	TNS	AL046979	2q35-q36
221748_s_at	15	TNS	AL046979	2q35-q36
218864_at	14	TNS1	NM_022648	2q35-q36
217853_at	15	TNS3	NM_022748	7p12.3
206093_x_at	13	TNXB	NM_007116	6p21.3
208609_s_at	13	TNXB	NM_019105	6p21.3 /// 6p21.3
213451_x_at	15	TNXB	BE044614	6p21.3
202704_at	15	TOB1	AA675892	17q21
222243_s_at	15	TOB2	AB051450	22q13.2-q13.31
208880_s_at	14	TOM; ANT-1	AB019219	20q13.33
202807_s_at	14	TOM1	NM_005488	22q13.1
204485_s_at	15	TOM1L1	NM_005486	17q23.2
200662_s_at	15	TOMM20	NM_014765	1q42
212773_s_at	15	TOMM20	BG165094	1q42
217960_s_at	14	TOMM22	NM_020243	22q12-q13
201870_at	13	TOMM34	NM_006809	---
202264_s_at	15	TOMM40	NM_006114	19q13
201812_s_at	15	TOMM7	NM_019059	7p15.3
201512_s_at	15	TOMM70A	BC003633	3q12.3
201519_at	15	TOMM70A	NM_014820	3q12.3
208901_s_at	15	TOP1	J03250	20q12-q13.1
201291_s_at	14	TOP2A	AU159942	17q21-q22
201292_at	15	TOP2A	AL561834	17q21-q22
211987_at	15	TOP2B	NM_001068	3p24
202633_at	15	TOPBP1	NM_007027	3q22.1
204071_s_at	14	TOPORS	NM_005802	9p21
78383_at	15	TOPORS	AI150117	---
202348_s_at	15	TOR1A	BC000674	9q34
204529_s_at	15	TOX	AI961231	8q12.1
209753_s_at	15	TP	AF113682	12q22
203050_at	11	TP53BP1	NM_005657	15q15-q21

203120_at	15	TP53BP2	NM_005426	1q42.1
210609_s_at	14	TP53I3	BC000474	2p24.1
218095_s_at	15	TPARL	BC003545	4q12
203476_at	15	TPBG	NM_006670	6q14-q15
217914_at	11	TPCN1	NM_017901	12q24.21
201688_s_at	14	TPD52	BG389015	8q21
201689_s_at	15	TPD52	NM_005079	8q21
201690_s_at	15	TPD52	AA524023	8q21
201379_s_at	15	TPD52L2	NM_003288	20q13.2-q13.3
200822_x_at	15	TPI1	NM_000365	12p13
213011_s_at	15	TPI1	BF116254	---
221218_s_at	14	TPK1	NM_022445	7q34-q35
206116_s_at	15	TPM1	NM_000366	15q22.1
206117_at	15	TPM1	NM_000366	15q22.1
210987_x_at	15	TPM1	M19267	---
204083_s_at	15	TPM2	NM_003289	9p13.2-p13.1
212654_at	15	TPM2	AL566786	9p13.2-p13.1
212481_s_at	15	TPM3	AI214061	19p13.1
209344_at	15	TPM4	BC002827	19p13.1
203672_x_at	15	TPMT	U12387	6p22.3
200742_s_at	15	TPP1	BG231932	11p15
200743_s_at	15	TPP1	NM_000391	11p15
203374_s_at	10	TPP2	AW612376	13q32-q33
203375_s_at	15	TPP2	NM_003291	13q32-q33
201730_s_at	15	TPR	BF110993	1q25
201731_s_at	15	TPR	NM_003292	1q25
220865_s_at	15	TPRT	NM_014317	10p12.2
204140_at	14	TPST1	NM_003596	7q11.21
204079_at	14	TPST2	NM_003595	22q12.1
211943_x_at	15	TPT1	AL565449	13q12-q14
212284_x_at	15	TPT1	BG498776	13q12-q14
212869_x_at	15	TPT1	AI721229	13q12-q14
214327_x_at	15	TPT1	AI888178	13q12-q14
216520_s_at	15	TPT1	AF072098	---
216540_at	10	TRA@	X61072	---
200598_s_at	15	TRA1	AI582238	12q24.2-q24.3
200599_s_at	15	TRA1	NM_003299	12q24.2-q24.3
204658_at	15	TRA2A	NM_013293	7p15.3
213593_s_at	14	TRA2A	AW978896	7p15.3
210180_s_at	11	TRA2B	U87836	3q26.2-q27
209965_s_at	11	TRAD-d1	AB016223	17q11
209451_at	12	TRAF2; I-TRAF	U59863	2q24-q31
204352_at	15	TRAF5	NM_004619	1q32
202079_s_at	15	TRAK1	NM_014965	3p25.3-p24.1
202080_s_at	15	TRAK1	NM_014965	3p25.3-p24.1
202124_s_at	15	TRAK2	NM_015049	2q33
202125_s_at	15	TRAK2	NM_015049	2q33
201398_s_at	15	TRAM1	BC000687	8q13.2
201399_s_at	15	TRAM1	BC000687	8q13.2
202369_s_at	14	TRAM2	NM_012288	6p21.1-p12

201391_at	15	TRAP1	NM_016292	16p13.3
201986_at	15	TRAP240	AB011165	17q22-q23
219351_at	10	TRAPPC2	NM_014563	Xp22
203512_at	15	TRAPPC3	NM_014408	1p34.3
217958_at	15	TRAPPC4	NM_016146	11q23.3
217959_s_at	15	TRAPPC4	NM_016146	11q23.3
204985_s_at	15	TRAPPC6A	NM_024108	19q13.32
213193_x_at	12	TRBV19 /// TRBC1	AL559122	---
209510_at	15	TRC8	AF064801	8q24
213334_x_at	15	TREX2	BE676218	Xq28
218425_at	15	TRIAD3	BC000787	7p22.2
202241_at	14	TRIB1	NM_025195	8q24.13
202478_at	15	TRIB2	NM_021643	2p25.1
202479_s_at	15	TRIB2	BC002637	2p25.1
218145_at	13	TRIB3	NM_021158	20p13-p12.2
204341_at	14	TRIM16	NM_006470	17p11.2
202342_s_at	15	TRIM2	NM_015271	4q31.3
213293_s_at	15	TRIM22	AA083478	11p15
204732_s_at	15	TRIM23	AI021991	5q12.3
202702_at	13	TRIM26	NM_003449	6p21.3
200990_at	15	TRIM28	NM_005762	19q13.4
213885_at	14	TRIM3	AA114843	11p15.5
203846_at	15	TRIM32	BC003154	9q33.2
212435_at	15	TRIM33	AA205593	---
203568_s_at	10	TRIM38	NM_006355	6p21.3
203610_s_at	12	TRIM38	AI363270	6p21.3
217759_at	15	TRIM44	AA176780	11p13
217760_at	15	TRIM44	AA176780	11p13
219923_at	14	TRIM45	NM_025188	1p12
221897_at	15	TRIM52	AA205660	5q35.3
213748_at	12	TRIM66	AW271713	11p15.3
219405_at	13	TRIM68	NM_018073	11p15.4
221012_s_at	15	TRIM8	NM_030912	10q24.3 /// 10q24.3
209011_at	13	TRIO	BF223718	5p15.1-p14
209012_at	15	TRIO	AV718192	5p15.1-p14
202795_x_at	15	TRIOBP	NM_007032	22q13.1
208756_at	15	TRIP1	U36764	1p34.1
202734_at	15	TRIP10	NM_004240	19p13.3
201546_at	15	TRIP12	NM_004238	2q37.1
204033_at	15	TRIP13	NM_004237	5p15.33
209778_at	11	Trip230	AF007217	14q31-q32
212544_at	15	TRIP3	AI131008	17q21.1
203732_at	15	TRIP4	NM_016213	15q22.1
209377_s_at	15	TRIP7	AF274949	6q14.3
218617_at	15	TRIT1	NM_017646	1p35.3-p34.1
217033_x_at	12	trkC	S76475	15q25
213634_s_at	12	TRMT1	AL031588	22q13
219299_at	13	TRMT12	NM_017956	8q24.13
211700_s_at	15	TRO	AF349719	Xp11.22-p11.21
211701_s_at	15	TRO	AF349720	Xp11.22-p11.21

201713_s_at	14	TRP1	D42063	2q12.3
205802_at	15	TRPC1	NM_003304	3q22-q24
205803_s_at	15	TRPC1	NM_003304	3q22-q24
212059_s_at	15	TRPC4AP	AL117480	20q11.23
218502_s_at	11	TRPS1	NM_014112	8q24.12
219282_s_at	15	TRPV2	NM_015930	17p11.2 /// 17p11.2
202642_s_at	15	TRRAP	NM_003496	7q21.2-q22.1
218977_s_at	13	TRSPAP1	BC000680	1p35.2
208864_s_at	15	TRX; MGC61975	AF313911	9q31
210803_at	11	TRXR2A	AF201385	22q11.21
202061_s_at	15	TSA305	AB020335	14q24.3-q31
218872_at	15	TSC	NM_017899	12q24.23
209390_at	15	TSC1	AF013168	9q34
215735_s_at	15	TSC2	AC005600	16p13.3
215111_s_at	15	TSC22	AK027071	13q14
204094_s_at	15	TSC22D2	NM_014779	3q25.1
218132_s_at	15	TSEN34	NM_024075	19q13.4
201758_at	15	TSG101	NM_006292	11p15
201513_at	15	TSN	NM_004622	2q21.1
201515_s_at	15	TSN	NM_004622	2q21.1
203983_at	15	TSNAX	NM_005999	1q42.1
219274_at	15	TSPAN12	NM_012338	7q31.31
217979_at	15	TSPAN13	NM_014399	7p21.2
221002_s_at	15	TSPAN14	NM_030927	10q23.1 /// 10q23.1
218693_at	13	TSPAN15	NM_012339	10q22.1
214606_at	10	TSPAN2	BF129969	1p13.1
200972_at	15	TSPAN3	BC000704	15q24.1
200973_s_at	15	TSPAN3	NM_005724	15q24.1
220558_x_at	14	TSPAN32	NM_005705	11p15.5
209263_x_at	15	TSPAN4	BC000389	11p15.5
209264_s_at	13	TSPAN-4	AF054841	11p15.5
209108_at	15	TSPAN-6	AF053453	Xq22
209109_s_at	14	TSPAN-6	AF053453	Xq22
202242_at	15	TSPAN7	NM_004615	Xp11.4
220968_s_at	15	TSPAN9	NM_031285	12p13.3 /// 12p13.3
221493_at	15	TSPYL	AL136629	6q22-q23
218012_at	10	TSPYL2	NM_022117	Xp11.2
212928_at	15	TSPYL4	AL050331	6q22.31
213122_at	15	TSPYL5	AI096375	8q22.1
217968_at	10	TSSC1	NM_003310	2p25.3
209781_s_at	15	T-Star	AF069681	8q24.2
213922_at	14	TTBK2	AW294686	---
218034_at	15	TTC11	NM_016068	7q22.1
203122_at	12	TTC15	NM_016030	2p25.3
218972_at	14	TTC17	NM_018259	11p11.2
217964_at	15	TTC19	NM_017775	17p11.2
219838_at	13	TTC23	NM_022905	15q26.3
208073_x_at	15	TTC3	NM_003316	21q22.2
208662_s_at	15	TTC3	AI885338	21q22.2
208663_s_at	14	TTC3	AI652848	21q22.2

46167_at	15	TTC4	W22690	1p31.3
204771_s_at	15	TTF1	NM_007344	9q34.3
204772_s_at	13	TTF1	NM_007344	9q34.3
204822_at	15	TTK	NM_003318	6q13-q21
205652_s_at	15	TTLL1	NM_012263	22q13.1
208195_at	15	TTN	NM_003319	2q31
222258_s_at	15	TTP; BOG25	AF015043	2q37.1-q37.2
202266_at	15	TTRAP	NM_016614	6p22.3-p22.1
212242_at	15	TUBA1	AL565074	2q36.1
209118_s_at	15	TUBA3	AF141347	12q12-12q14.3
209251_x_at	15	TUBA6	BC004949	12q12-q14
211750_x_at	15	TUBA6	BC005946	12q12-q14 /// 12q12-q14
211714_x_at	15	TUBB	BC005838	6p21.32 /// 6p21.32
213726_x_at	15	TUBB2	AA515698	---
204141_at	15	TUBB2A	NM_001069	6p25
208977_x_at	15	TUBB2C	BC004188	---
202154_x_at	15	TUBB3	NM_006086	16q24.3
213476_x_at	15	TUBB3	AL565749	16q24.3
209191_at	15	TUBB6	BC002654	18p11.21
210389_x_at	15	TUBD1	BC000258	17q23.2
201714_at	15	TUBG1	NM_001070	17q21
203894_at	15	TUBG2	NM_016437	17q21
202477_s_at	15	TUBGCP2	NM_006659	10q26.3
203690_at	14	TUBGCP3	NM_006322	13q34
205854_at	15	TUBL3; MGC45295	AK024246	12p13.3
201113_at	15	TUFM	NM_003321	16p11.2
205807_s_at	15	TUFT1	NM_020127	1q21
222244_s_at	15	TUG1	AK000749	22q12.2
218184_at	12	TULP4	NM_020245	6q25-q26
203272_s_at	10	TUSC2	NM_007275	3p21.3
203273_s_at	10	TUSC2	NM_007275	3p21.3
209227_at	15	TUSC3	AU158251	8p22
213423_x_at	15	TUSC3	AI884858	8p22
203246_s_at	15	TUSC4	NM_006545	3p21.3
213943_at	15	TWIST1	X99268	7p21.2
209310_s_at	10	TX	U25804	11q22.2-q22.3
209077_at	15	TXN2	AL022313	22q13.1
208097_s_at	13	TXNDC	NM_030755	14q22.1 /// 14q22.1
209476_at	15	TXNDC	AL080080	14q22.1
201175_at	15	TXNDC14	NM_015959	11cen-q22.3
208957_at	14	TXNDC4	BF439241	9q31.1
208958_at	12	TXNDC4	AI827677	9q31.1
208959_s_at	15	TXNDC4	BC005374	9q31.1
221253_s_at	15	TXNDC5	NM_030810	6p24.3 /// 6p24.3
216640_s_at	15	TXNDC7	AK026926	---
203008_x_at	15	TXNDC9	NM_005783	2q11.2
211758_x_at	15	TXNDC9	BC005968	2q11.2 /// 2q11.2
201008_s_at	15	TXNIP	NM_006472	1q21.2
201009_s_at	15	TXNIP	NM_006472	1q21.2

201010_s_at	15	TXNIP	NM_006472	1q21.2
201588_at	15	TXNL1	NM_004786	18q21.31
216532_x_at	11	TXNL2	AL138831	---
202836_s_at	15	TXNL4A	BC001046	18q23
218794_s_at	10	TXNL4B	AW194729	16q22.2
201266_at	15	TXNRD1	NM_003330	12q23-q24.1
205546_s_at	15	TYK2	NM_003331	19p13.2
202589_at	15	TYMS	NM_001071	18p11.32
204122_at	15	TYROBP	NM_003332	19q13.1
205694_at	14	TYRP1	NM_000550	9p23
209422_at	15	TZP	AY027523	20q11.22-q11.23
205300_s_at	14	U1SNRNPBP	NM_022717	12q24.31
202858_at	15	U2AF1	NM_006758	21q22.3
208174_x_at	15	U2AF1L2	NM_005089	Xp22.1
213876_x_at	15	U2AF1L2	AW089584	Xp22.1
219690_at	10	U2AF1L4	NM_024660	19q13.13
218381_s_at	15	U2AF2	NM_007279	19q13.43
209340_at	15	UAP1	S73498	1q23.2
201177_s_at	15	UBA2	NM_005499	19q12
221700_s_at	15	UBA52	AF348700	19p13.1-p12 /// 19p13.1-p12
202151_s_at	15	UBADC1	NM_016172	9q34.3
46270_at	15	UBAP1	AL039447	9p22-p21
219192_at	12	UBAP2	NM_018449	9p13.2
221839_s_at	15	UBAP2	AK026088	9p13.2
201377_at	14	UBAP2L	NM_014847	1q22
201378_s_at	10	UBAP2L	NM_014847	1q22
209947_at	15	UBAP2L	BC003170	1q22
214695_at	13	UBAP2L	AW051361	1q22
200633_at	15	UBB	NM_018955	17p12-p11.2
217144_at	11	UBBP1	X04801	---
211296_x_at	15	UBC	AB009010	12q24.3
217823_s_at	15	Ubc6p	AF151039	6q16.1
217825_s_at	15	Ubc6p	AF151039	6q16.1
217826_s_at	15	Ubc6p	AF151039	6q16.1
210024_s_at	15	UbcH9	AB017644	2q32.1
200964_at	15	UBE1	NM_003334	Xp11.23
209115_at	15	UBE1C	AL117566	3p24.3-p13
218289_s_at	15	UBE1DC1	NM_024818	3q22.1
1294_at	15	UBE1L	L13852	3p21
201898_s_at	15	UBE2A	NM_003336	Xq24-q25
201899_s_at	15	UBE2A	NM_003336	Xq24-q25
202333_s_at	15	UBE2B	NM_003337	5q23-q31
202334_s_at	15	UBE2B	AI768723	5q23-q31
202335_s_at	10	UBE2B	NM_003337	5q23-q31
211763_s_at	15	UBE2B	BC005979	5q23-q31 /// 5q23-q31
202954_at	15	UBE2C	NM_007019	20q13.12
211764_s_at	15	UBE2D1	BC005980	10q11.2-q21 /// 10q11.2-q21
214590_s_at	12	UBE2D1	AL545760	10q11.2-q21

201343_at	15	UBE2D2	BE621259	5q31.3
201344_at	15	UBE2D2	BF196642	5q31.3
201345_s_at	15	UBE2D2	NM_003339	5q31.3
200667_at	15	UBE2D3	BF448062	4q24
200668_s_at	15	UBE2D3	BC003395	4q24
200669_s_at	15	UBE2D3	NM_003340	4q24
218837_s_at	13	UBE2D4	NM_015983	7p13
212519_at	15	UBE2E1	AL518159	3p24.2-p24.1
209141_at	15	UBE2G1	AW299555	1q42
209142_s_at	15	UBE2G1	BC002775	1q42
209041_s_at	11	UBE2G2	BG395660	21q22.3
209042_s_at	15	UBE2G2	BC001738	21q22.3
221962_s_at	15	UBE2H	AI829920	7q32
208760_at	15	UBE2I	AL031714	16p13.3
213535_s_at	15	UBE2I	AA910614	16p13.3
200676_s_at	15	UBE2L3	NM_003347	22q11.21
200682_s_at	15	UBE2L3	NM_003347	22q11.21
200683_s_at	14	UBE2L3	BE964689	22q11.21
200684_s_at	15	UBE2L3	NM_003347	22q11.21
201649_at	15	UBE2L6	NM_004223	11q12
203109_at	11	UBE2M	NM_003969	19q13.43
201523_x_at	15	UBE2N	BE262760	12q22
201524_x_at	15	UBE2N	NM_003348	12q22
212751_at	15	UBE2N	BG290646	12q22
217978_s_at	15	UBE2Q1	NM_017582	1q22
202779_s_at	15	UBE2S	NM_014501	19q13.43
201001_s_at	15	UBE2V1	BG164064	20q13.2
201002_s_at	15	UBE2V1	NM_003349	20q13.2
201003_x_at	15	UBE2V1	NM_003349	20q13.2
211285_s_at	15	UBE3A	U84404	15q11-q13
211575_s_at	15	UBE3A	AF116702	---
213128_s_at	15	UBE3A	AA527499	15q11-q13
213291_s_at	15	UBE3A	AA160522	15q11-q13
212403_at	15	UBE3B	AI749193	12q24.12
213822_s_at	15	UBE3B	BE856776	12q24.12
201817_at	15	UBE3C	NM_014671	7q36.3
202038_at	15	UBE4A	NM_004788	11q23.3
202317_s_at	15	UBE4B	NM_006048	1p36.3
201535_at	15	UBL3	NM_007106	13q12-q13
218011_at	15	UBL5	NM_024292	19p13.3
209088_s_at	15	UBN1	T70262	16p13.3
221654_s_at	15	UBP	AF077040	15q22.3
218082_s_at	15	UBP1	NM_014517	3p22.3
205687_at	15	UBPH	NM_019116	16p12
215884_s_at	15	UBQLN2	AK001029	---
212756_s_at	15	UBR2	AI761518	6p21.1
202692_s_at	15	UBTF	NM_014233	17q21.3
220757_s_at	15	UBXD1	NM_025241	19p13
212006_at	15	UBXD2	AU149908	2q21.3-q22.1
212007_at	13	UBXD2	AI927512	2q21.3-q22.1

212008_at	15	UBXD2	N29889	2q21.3-q22.1
201387_s_at	12	UCHL1	NM_004181	4p14
204616_at	15	UCHL3	NM_006002	13q21.33
219960_s_at	15	UCHL5	NM_015984	1q32
220083_x_at	11	UCHL5	NM_016017	1q32
218533_s_at	15	UCKL1	NM_017859	20q13.33
208998_at	15	UCPH	U94592	11q13 /// 11q13
218190_s_at	15	UCRC	NM_013387	22cen-q12.3
220775_s_at	11	UEV3	NM_018314	11p15.1
217797_at	15	UFC1	NM_016406	1q23.1
209103_s_at	15	UFD1L	BC001049	22q11.21
218050_at	15	UFM1	NM_016617	13q13.3
204881_s_at	15	UGCG	NM_003358	9q31
221765_at	10	UGCG	AI378044	9q31
218801_at	13	UGCGL2	NM_020121	13q32.1-q32.2
203343_at	14	UGDH	NM_003359	4p15.1
205480_s_at	15	UGP2	NM_006759	2p14-p13
209326_at	15	UGT	D84454	Xp11.23-p11.22
209333_at	11	ULK1	AB018265	12q24.3
204062_s_at	15	ULK2	BG526973	17p11.2
204063_s_at	14	ULK2	NM_014683	17p11.2
202706_s_at	15	UMPS	NM_000373	3q13
215165_x_at	15	UMPS	AL080099	3q13
202893_at	15	UNC13B	NM_006377	9p12-p11
221708_s_at	15	UNC45A	BC006214	15q26.1 /// 15q26.1
203583_at	15	UNC50	NM_014044	2q11.2
41856_at	15	UNC5B	AL049370	10q22.2
212074_at	15	UNC84A	BE972774	7p22.3
214169_at	15	UNC84A	BE615699	7p22.3
212144_at	15	UNC84B	AL021707	22q13.1
202330_s_at	15	UNG	NM_003362	12q23-q24.1
211800_s_at	15	UNP	AF017306	3p21.3
209475_at	15	UNPH4	AF106069	12q14
212305_s_at	13	UNQ6077; ARNT	AK025122	1q42.12
202646_s_at	15	UNR	AA167775	1p22
203519_s_at	15	UPF2	NM_015542	10p14-p13
206958_s_at	15	UPF3	AF318575	13q34
206959_s_at	13	UPF3	AF318575	13q34
214323_s_at	15	UPF3A	N36842	13q34
218757_s_at	14	UPF3B	NM_023010	Xq25-q26
210681_s_at	15	UPH	AF153604	12q14
222236_s_at	12	UPLC1	AK000253	1p36.11
203234_at	14	UPP1	NM_003364	7p12.3
209066_x_at	15	UQBP	M26700	8q22
202090_s_at	15	UQCR	NM_006830	19p13.3
205849_s_at	15	UQCRB	NM_006294	8q22
209065_at	15	UQCRB	BC005230	8q22
201903_at	15	UQCRC1	NM_003365	3p21.3
200883_at	15	UQCRC2	NM_003366	16p12
212600_s_at	15	UQCRC2	AV727381	16p12

208909_at	15	UQCRFS1	BC000649	19q12-q13.1
202233_s_at	15	UQCRH	NM_006004	1p34.1
201568_at	15	UQCRQ	NM_014402	5q31.1
208714_at	15	UQOR1	AF092131	11q13
208970_s_at	15	UROD	M14016	1p34
208971_at	15	UROD	M14016	1p34
203031_s_at	15	UROS	NM_000375	10q25.2-q26.3
202152_x_at	15	USF2	NM_003367	19q13
215737_x_at	15	USF2	X90824	19q13
202412_s_at	13	USP1	AW499935	1p32.1-p31.3
202413_s_at	15	USP1	AW499935	1p32.1-p31.3
209137_s_at	14	USP10	BC000263	16q24.1
211702_s_at	14	USP10	AF350251	17q23.3 /// 17q23.3
208723_at	15	USP11	BC000350	Xp11.23
213327_s_at	15	USP12	AI820101	13q12.2
205356_at	15	USP13	NM_003940	3q26.2-q26.3
201671_x_at	10	USP14	BC003556	18p11.32
201672_s_at	15	USP14	NM_005151	18p11.32
218386_x_at	15	USP16	AI806796	21q22.11
218367_x_at	14	USP21	NM_012475	1q22
200083_at	15	USP22	AA621731	17p11.2
212381_at	12	USP24	AB028980	1p32.3
212388_at	13	USP24	AB028980	1p32.3
220419_s_at	15	USP25	NM_013396	21q11.2
212513_s_at	15	USP33	AB029020	1p31.1
207365_x_at	14	USP34	NM_014709	2p15
212066_s_at	15	USP34	AB018272	2p15
212980_at	15	USP34	AL050376	2p16.1-p15
217829_s_at	13	USP39	NM_006590	2p11.2
202681_at	14	USP4	AI346043	3p21.31
202682_s_at	15	USP4	NM_003363	3p21.3
203869_at	13	USP46	AK024318	4q12
203870_at	15	USP46	BE856374	4q12
221518_s_at	15	USP47	BC000226	11p15.3
220078_at	13	USP48	NM_018391	1p36.12
220079_s_at	15	USP48	NM_018391	1p36.12
206031_s_at	12	USP5	NM_003481	12p13
203117_s_at	15	USP52	NM_014871	12q13.2-q13.3
206405_x_at	13	USP6	NM_004505	17q11
201498_at	15	USP7	AI160440	16p13.3
201499_s_at	15	USP7	NM_003470	16p13.3
202745_at	15	USP8	NM_005154	15q15.3
201099_at	15	USP9X	NM_004652	Xp11.4
201100_s_at	15	USP9X	NM_004652	Xp11.4
204190_at	15	USPL1	NM_005800	13q12-q14
205139_s_at	15	UST	NM_005715	6q24.3
218235_s_at	15	UTP11L	NM_016037	1p34.2
221514_at	12	UTP14A	BC001149	Xq26.1
203614_at	15	UTP14C	NM_021645	13q12.2-q13.3
213022_s_at	13	UTRN	NM_007124	6q24

203990_s_at	12	UTX	AI140752	Xp11.2
203991_s_at	15	UTX	NM_021140	Xp11.2
203992_s_at	15	UTX	AF000992	Xp11.2
203241_at	15	UVRAG	NM_003369	11q13.5
219675_s_at	15	UXS1	NM_025076	2q12.3
218495_at	15	UXT	NM_004182	Xp11.23-p11.22
208780_x_at	15	VAMP	AF154847	18p11.22
213326_at	13	VAMP1	AU150319	12p
201556_s_at	15	VAMP2	BC002737	17p13.1
201557_at	15	VAMP2	NM_014232	17p13.1
214792_x_at	15	VAMP2	AI955119	17p13.1
201336_at	15	VAMP3	BC003570	1p36.23
201337_s_at	10	VAMP3	NM_004781	1p36.23
211749_s_at	15	VAMP3	BC005941	1p36.23 /// 1p36.23
207350_s_at	14	VAMP4	NM_003762	1q24-q25
211760_s_at	13	VAMP4	BC005974	1q24-q25 /// 1q24-q25
213480_at	15	VAMP4	AF052100	1q24-q25
204929_s_at	15	VAMP5	NM_006634	2p11.2
202546_at	15	VAMP8	NM_003761	2p12-p11.2
219330_at	13	VANGL1	NM_024062	1p13.1
203940_s_at	15	VASH1	NM_014909	14q24.3
202205_at	15	VASP	NM_003370	19q13.2-q13.3
208626_s_at	15	VAT1	BC001913	17q21
208898_at	15	VATD	AF077614	---
208899_x_at	15	VATD	AF100741	---
214923_at	13	VATD	AK001155	14q24.1
218807_at	14	VAV3	NM_006113	1p13.3
201472_at	15	VBP1	NM_003372	Xq28
203868_s_at	15	VCAM1	NM_001078	1p32-p31
200931_s_at	15	VCL	NM_014000	10q22.1-q23
208648_at	15	VCP	W60953	9p13-p12
212038_s_at	15	VDAC1	AL515918	5q31
217140_s_at	15	VDAC1	AJ002428	---
211662_s_at	15	VDAC2	L08666	10q22 /// 10q22
208845_at	15	VDAC3	BC002456	---
201831_s_at	12	VDP	BE875592	4q21.21
201832_s_at	15	VDP	NM_003715	4q21.21
222316_at	14	VDP	AW973253	---
214843_s_at	15	VDU1	AK022864	1p31.1
212171_x_at	15	VEGF	H95344	6p12
203683_s_at	15	VEGFB	NM_003377	11q13
214004_s_at	15	VGLL4	AI806207	3p25.2
208622_s_at	14	VIL2	AA670344	6q25.2-q26
208623_s_at	15	VIL2	J05021	6q25.2-q26
201426_s_at	15	VIM	AI922599	10p13
217949_s_at	15	VKORC1	NM_024006	16p11.2
209822_s_at	15	VLDLRCH	L22431	9p24
210512_s_at	15	VEGF	AF022375	6p12
210513_s_at	15	VEGF	AF091352	6p12

211527_x_at	15	VEGF	M27281	6p12
213686_at	15	VPS13A	AI186145	---
218396_at	15	VPS13C	NM_017684	15q21.3
212323_s_at	15	VPS13D	AA524553	1p36.21
203459_s_at	15	VPS16	NM_022575	--- /// ---
217837_s_at	15	VPS24	NM_016079	2p24.3-p24.1
201807_at	15	VPS26A	NM_004896	10q21.1
218679_s_at	15	VPS28	NM_016208	8q24.3
44111_at	14	VPS33B	AI672363	15q26.1
217727_x_at	15	VPS35	NM_018206	16q12
221704_s_at	12	VPS37B	BC005882	12q24.31 /// 12q24.31
219053_s_at	15	VPS37C	NM_017966	11q12.3
218171_at	15	VPS42	AF195514	18q21.32-q21.33
209268_at	15	VPS45	AF165513	1q21-q22
217913_at	15	VPS4A	NM_013245	16q22.1
214585_s_at	13	VPS52	AL390171	6p21.3
218423_x_at	15	VPS54	NM_016516	2p13-p14
202261_at	15	VPS72	NM_005997	1q21
203856_at	11	VRK1	NM_003384	14q32
205126_at	14	VRK2	NM_006296	2p16-p15
218022_at	13	VRK3	NM_016440	19q13
221998_s_at	13	VRK3	BF062886	19q13
221999_at	15	VRK3	BF062886	19q13
209946_at	15	VRP; Flt4-L	U58111	4q34.1-q34.3
204787_at	10	VSIG4	NM_007268	Xq12-q13.3
203797_at	13	VSNL1	AF039555	2p24.3
203798_s_at	11	VSNL1	NM_003385	2p24.3
209452_s_at	15	VTI1	AF035824	14q24.1
202112_at	15	VWF	NM_000552	12p13.3
217742_s_at	15	WAC	NM_016628	---
219679_s_at	14	WAC	NM_018604	---
200629_at	15	WARS	NM_004184	14q32.31
221725_at	15	WASF2	AI962978	1p36.11-p34.3
204042_at	15	WASF3	AB020707	13q12
38964_r_at	14	WASP	U12707	Xp11.4-p11.21
202663_at	13	WASPIP	AI005043	2q31.2
202664_at	15	WASPIP	AW058622	2q31.2
202665_s_at	11	WASPIP	NM_003387	2q31.2
217821_s_at	13	WBP11	NM_016312	12p13.1
217822_at	15	WBP11	NM_016312	12p13.1
209117_at	15	WBP2	U79458	17q25
203599_s_at	15	WBP4	NM_007187	13q13.3
217975_at	15	WBP5	NM_016303	Xq22.2
206621_s_at	15	WBSCR1	NM_022170	7q11.23
221247_s_at	15	WBSCR16	NM_030798	7q11.23
213670_x_at	11	WBSCR20B	AI768378	7q11.23
214100_x_at	10	WBSCR20B	AI284845	7q11.23
213460_x_at	14	WBSCR20C	N29665	7q11.23
221927_s_at	15	WBSCR21	AI923458	7q11.23

207628_s_at	15	WBSCR22	NM_017528	---
212606_at	15	WDFY3	AL536319	4q22.1
200609_s_at	15	WDR1	NM_017491	4p16.1
218512_at	15	WDR12	NM_018256	2q33.3
220917_s_at	15	WDR19	NM_025132	4p14
201886_at	15	WDR23	NM_025230	14q11.2
218107_at	15	WDR26	NM_025160	1q42.12-q42.13
218882_s_at	14	WDR3	NM_006784	1p13-p12
219001_s_at	12	WDR32	NM_024345	9p13.1
218851_s_at	14	WDR33	NM_018383	2q21.1
203536_s_at	10	WDR39	NM_004804	2q11.2
217501_at	15	WDR39	AI339732	2q11.2
218055_s_at	15	WDR41	NM_018268	5q14.1
202249_s_at	14	WDR42A	AU146233	1q22-q23
202250_s_at	15	WDR42A	NM_015726	1q22-q23
219297_at	14	WDR44	NM_019045	Xq24
209216_at	15	WDR45	BC000464	Xp11.23
209217_s_at	15	WDR45	BC000464	Xp11.23
215553_x_at	14	WDR45	AK024315	---
209076_s_at	15	WDR45L	BC000974	17q25.3
203855_at	14	WDR47	NM_014969	1p13.3
221735_at	15	WDR48	H04342	---
56919_at	14	WDR48	AI806628	3p22-p21.33
65591_at	12	WDR48	N64681	3p21.33
203721_s_at	15	WDR50	NM_016001	17q21.33
219809_at	15	WDR55	NM_017706	5q31.3
218505_at	12	WDR59	NM_024673	16q22.3
219538_at	15	WDR5B	NM_019069	3q21.1
217734_s_at	15	WDR6	NM_018031	15q21
219251_s_at	10	WDR60	NM_018051	7q36.3
212880_at	15	WDR7	AB011113	18q21.1-q22
219193_at	10	WDR70	NM_018034	5p13.2
218957_s_at	15	WDR71	NM_025155	11q13.3
221712_s_at	15	WDR74	BC006351	11q12.3
201421_s_at	15	WDR77	NM_024102	1p13.2
40829_at	11	WDTC1	AB028960	1p35.3
215711_s_at	14	WEE1	AJ277546	11p15.3-p15.1
212533_at	15	Wee1 Hu	X62048	11p15.3-p15.1
203892_at	13	WFDC2	NM_006103	20q12-q13.2
202908_at	15	WFS1	NM_006005	4p16
209053_s_at	11	WHSC1	BE793789	4p16.3
209054_s_at	15	WHSC1	AF083389	4p16.3
218173_s_at	11	WHSC1L1	NM_017778	8p11.2
34225_at	15	WHSC2	AF101434	4p16.3
203827_at	15	WIPI1	NM_017983	17q24.3
202031_s_at	15	WIPI2	NM_015610	7p22.2
204710_s_at	15	WIPI2	NM_016003	7p22.2
213836_s_at	15	WIPI49	AW052084	17q24.3
212049_at	13	WIRE	BG230612	17q21.2
212051_at	11	WIRE	AA676803	17q21.2

212050_at	15	WIRE; WICH	AK026913	17q21.2
211992_at	15	WNK1	AI445745	12p13.3
211993_at	10	WNK1	AI768512	12p13.3
205990_s_at	13	WNT5A	NM_003392	3p21-p14
213425_at	10	WNT5A	AI968085	3p21-p14
71933_at	13	WNT6	AI218134	2q35
202749_at	15	WRB	NM_004627	21q22.3
201294_s_at	15	WSB1	N24643	17q11.2
201295_s_at	15	WSB1	BF111821	17q11.2
210561_s_at	15	WSB1	AL110243	17q11.2
201760_s_at	15	WSB2	NM_018639	12q24.23
213734_at	13	WSB2	BG260658	12q24.2-q24.3
206067_s_at	13	WT1	NM_024426	11p13
203137_at	15	WTAP	NM_004906	6q25-q27
210285_x_at	15	WTAP	BC000383	6q25-q27
214759_at	11	WTAP	AL583911	6q25-q27
215794_x_at	15	WUGSC:H_DJ0296G17.1	AC006144	Xq24-q25
216508_x_at	15	WUGSC:H_NH0244E06.1	AC007277	---
217249_x_at	15	WUGSC:H_RG162B04.1	AC004544	---
219077_s_at	13	WWOX	NM_016373	16q23.3-q24.1
212637_s_at	14	WWP1	AU155187	8q21
212638_s_at	15	WWP1	BF131791	8q21
204022_at	10	WWP2	AI668780	16q22.1
202132_at	14	WWTR1	AA081084	3q23-q24
202133_at	15	WWTR1	NM_015472	3q23-q24
213900_at	15	X123	AA524029	9q13-q21
209313_at	15	XAB1	AB044661	2p23.3
216333_x_at	15	TNXB1	M25813	6p21.3
210720_s_at	15	XB51	AB039947	20q11.22
200670_at	15	XBP1	NM_005080	22q12.1
206698_at	14	XK	NM_021083	Xp21.1
218753_at	15	XKR8	NM_018053	1p35.2
211858_x_at	15	XL	AF088184	20q13.2-q13.3
214548_x_at	15	XL	AF064092	20q13.2-q13.3
219059_s_at	14	XLKD1	NM_006691	11p15
220037_s_at	15	XLKD1	NM_016164	11p15 /// 11p15
205672_at	15	XPA	NM_000380	9q22.3
209375_at	14	XPCC	D21089	3p25
208619_at	15	XPE	L40326	11q12-q13
208453_s_at	15	XPNPEP1	NM_006523	10q25.3 /// 10q25.3
209045_at	15	XPNPEP1	AF195530	10q25.3
218479_s_at	15	XPO4	NM_022459	13q11
211982_x_at	15	XPO6	AL546600	16p12.1
208459_s_at	10	XPO7	NM_015024	8p21
212166_at	11	XPO7	H38643	8p21
212160_at	15	XPOT	AI984005	12q14.1
203655_at	15	XRCC1	NM_006297	19q13.2
208642_s_at	15	XRCC5	AA205834	2q35
200792_at	15	XRCC6	NM_001469	22q13.2-q13.31
211946_s_at	15	XTP2	AI359472	1q23.3

211948_x_at	13	XTP2	BG261071	1q23.3
214055_x_at	15	XTP2	AW238632	1q23.3
218069_at	15	XTP3TPA	NM_024096	16p12.1
203712_at	15	XTP5	NM_014878	9p24.2
206238_s_at	11	YAF2	NM_005748	12q12
213342_at	14	YAP1	AI745185	11q13
212048_s_at	15	YARS	AW245400	1p34.3
218470_at	12	YARS2	NM_015936	12p11.21
208628_s_at	15	YBX1	BC002411	1p34
201844_s_at	15	YEAF1	AB029551	3p14.2
201845_s_at	15	YEAF1	AB029551	3p14.2
221203_s_at	15	YEATS2	NM_018023	3q27.3
218911_at	11	YEATS4	NM_006530	12q13-q15
202932_at	15	YES1	NM_005433	18p11.31-p11.21
202933_s_at	15	YES1	NM_005433	18p11.31-p11.21
222180_at	10	YES1	AU147889	---
202418_at	15	YIF1A	NM_020470	11q13
209551_at	15	YIPF4	BC004875	2p23.2
221423_s_at	15	YIPF5	NM_030799	5q32 /// 5q32
212787_at	15	YLPM1	AI952986	14q24.3
201351_s_at	15	YME1L1	NM_014263	10p14
201352_at	15	YME1L1	NM_014263	10p14
213996_at	15	YPEL1	NM_013313	22q11.2
217783_s_at	15	YPEL5	NM_016061	2p23.3
212455_at	15	YT521	N36997	4q13.3
214814_at	12	YT521	BF592058	4q13.3
213077_at	15	YTHDC2	AL049305	5q22.3
221749_at	15	YTHDF3	AU157915	8q12.2
208743_s_at	15	YWHAB	BC001359	20q13.1
217717_s_at	15	YWHAB	NM_014052	20q13.1
217718_s_at	15	YWHAB	NM_014052	20q13.1
213655_at	15	YWHAE	AA502643	17p13.3
201020_at	15	YWHAH	NM_003405	22q12.3
200693_at	15	YWHAQ	NM_006826	2p25.2-p25.1
212426_s_at	15	YWHAQ	BF033313	2p25.2-p25.1
213699_s_at	15	YWHAQ	AA854017	---
200638_s_at	15	YWHAZ	BC003623	8q23.1
200639_s_at	15	YWHAZ	NM_003406	8q23.1
200640_at	15	YWHAZ	NM_003406	8q23.1
200641_s_at	15	YWHAZ	BC003623	8q23.1
217836_s_at	15	YY1AP1	NM_018253	1q22
217741_s_at	15	ZA20D2	NM_006007	9q13-q21
216887_s_at	15	ZASP	AJ133768	10q22.3-q23.2
203043_at	14	ZBED1	NM_004729	Xp22.33
213376_at	15	ZBTB1	AI656706	14q23.3
204847_at	15	ZBTB11	NM_014415	3q12.3
205883_at	14	ZBTB16	NM_006006	11q23.1
203602_s_at	15	ZBTB17	NM_003443	1p36.2-p36.1
205383_s_at	13	ZBTB20	NM_015642	3q13.2
214482_at	11	ZBTB25	NM_006977	14q23-q24 /// 14q23-

				q24
205256_at	11	ZBTB39	NM_014830	12q13.2
203026_at	15	ZBTB5	NM_014872	9p13.1
205788_s_at	15	ZC3H11A	NM_014827	1q32.1
218348_s_at	15	ZC3H7A	NM_014153	16p13-p12
213051_at	12	ZC3HAV1	AI133727	7q34
221193_s_at	15	ZCCHC10	NM_017665	5q31.1
212704_at	15	ZCCHC11	AI049962	1p32.3
219062_s_at	15	ZCCHC2	BE676543	18q21.33
220933_s_at	13	ZCCHC6	NM_024617	9q21
218478_s_at	15	ZCCHC8	NM_017612	12q24.31
212725_s_at	15	ZCWCC1	N37081	22q12.2
216863_s_at	13	ZCWCC1	AC004542	---
213000_at	14	ZCWCC3	AP000693	21q22.13
219296_at	15	ZDHHC13	NM_019028	11p15.1
212982_at	15	ZDHHC17	AI621223	12q21.1
212860_at	10	ZDHHC18	BG168720	1p35.3
218077_s_at	13	ZDHHC3	NM_016598	3p21.32
218078_s_at	15	ZDHHC3	NM_016598	3p21.32
220261_s_at	15	ZDHHC4	NM_018106	7p22.2
218249_at	15	ZDHHC6	NM_022494	10q25.3
218606_at	15	ZDHHC7	NM_017740	16q24.1
202978_s_at	11	ZF	NM_021212	11q14
202979_s_at	15	ZF	NM_021212	11q14
218919_at	15	ZFAND1	NM_024699	8q21.13
218020_s_at	15	ZFAND3	NM_021943	6pter-p22.3
211485_s_at	14	ZFGF5; FGF-18	AF211188	5q34
203603_s_at	15	ZFHX1B	NM_014795	2q22
211257_x_at	15	ZFML	AF273049	2p13.2-p13.1
217781_s_at	15	ZFP106	AI493587	15q14
201531_at	15	ZFP36	NM_003407	19q13.1
211962_s_at	15	ZFP36L1	BG250310	14q22-q24
211965_at	15	ZFP36L1	BE620915	14q22-q24
201368_at	15	ZFP36L2	AI356398	---
201369_s_at	15	ZFP36L2	NM_006887	2p22.3-p21
218968_s_at	15	ZFP64	NM_018197	20q13.13
203731_s_at	11	ZFP95	NM_014569	7q22
209428_s_at	10	ZFPL1	BG420865	11q13
219778_at	15	ZFPM2	NM_012082	8q23
201856_s_at	15	ZFR	BC000376	5p13.3
201857_at	15	ZFR	NM_016107	5p13.3
203651_at	15	ZFYVE16	NM_014733	5p15.2-q14.3
219929_s_at	14	ZFYVE21	NM_024071	14q32.33
213073_at	13	ZFYVE26	AB002319	14q24.1
37943_at	14	ZFYVE26	AB002319	14q24.1
204893_s_at	10	ZFYVE9	NM_004799	1p32.3
57539_at	15	ZGPAT	AA535065	20q13.3
203556_at	15	ZHX2	NM_014943	8q24.13
202939_at	15	ZMPSTE24	NM_005857	1p34
220206_at	11	ZMYM1	NM_024772	1p34.3

207559_s_at	15	ZMYM3	NM_005096	Xq13.1
202049_s_at	15	ZMYM4	NM_005095	1p32-p34
202051_s_at	15	ZMYM4	NM_005095	1p32-p34
219924_s_at	12	ZMYM6	NM_007167	1p34.2
202136_at	15	ZMYND11	BE250417	10p14
202137_s_at	12	ZMYND11	NM_006624	10p14
212798_s_at	15	ZMYND20	AK001389	7p21
207605_x_at	10	ZNF117	NM_024498	7q11.21
219571_s_at	15	ZNF12	NM_016265	7p22.2
221842_s_at	15	ZNF131	BE972394	5p12-p11
216960_s_at	15	ZNF133	AL049646	---
37254_at	15	ZNF133	U09366	20p11.23-20p11.22
206182_at	13	ZNF134	NM_003435	19q13.4
206240_s_at	15	ZNF136	NM_003437	19p13.2-p13.12
207394_at	12	ZNF137	NM_003438	19q13.4
219854_at	11	ZNF14	NM_021030	19p13.3-p13.2
204523_at	14	ZNF140	NM_003440	12q24.32-q24.33
221873_at	13	ZNF143	AW162015	11p15.4
200050_at	15	ZNF146	NM_007145	19q13.1
203318_s_at	14	ZNF148	NM_021964	3q21
219548_at	13	ZNF16	NM_006958	8q24
202171_at	15	ZNF161	AU146275	17q23.2
202172_at	15	ZNF161	BG035116	17q23.2
202173_s_at	15	ZNF161	NM_007146	17q23.2
206314_at	14	ZNF167	NM_018651	3p22.3-p21.1
210290_at	12	ZNF174	BC001161	16p13.3
207417_s_at	15	ZNF177	NM_003451	19p13.2
203585_at	15	ZNF185	NM_007150	Xq28
213218_at	13	ZNF187	AV705032	6p21.31
207513_s_at	15	ZNF189	NM_003452	9q22-q31
205181_at	14	ZNF193	NM_006299	6p21.3
204234_s_at	15	ZNF195	AI476267	11p15.5
205855_at	15	ZNF197	NM_006991	3p21
202778_s_at	15	ZNF198	NM_003453	13q11-q12
210282_at	10	ZNF198	AL136621	13q11-q12
213916_at	10	ZNF20	AU154474	19p13.3-p13.2
214706_at	14	ZNF200	AU149447	16p13.3
204327_s_at	14	ZNF202	NM_003455	11q23.3
200828_s_at	15	ZNF207	NM_003457	17q12
200829_x_at	15	ZNF207	NM_003457	17q12
205437_at	13	ZNF211	NM_006385	19q13.4
203985_at	15	ZNF212	NM_012256	7q36.1
210275_s_at	15	ZNF216	AF062347	9q13-q21
203739_at	12	ZNF217	NM_006526	20q13.2
219314_s_at	14	ZNF219	NM_016423	14q11
218005_at	15	ZNF22	AA744771	10q11
218006_s_at	15	ZNF22	AA744771	10q11
207128_s_at	14	ZNF223	NM_013361	19q13.2
219603_s_at	15	ZNF226	NM_015919	19q13.2
222237_s_at	14	ZNF228	AC084239	---

213934_s_at	10	ZNF23	AL567808	16q22
219123_at	15	ZNF232	NM_014519	17p13-p12
47571_at	13	ZNF236	AA004757	18q22-q23
215948_x_at	13	ZNF237	AI522311	13q12
203247_s_at	13	ZNF24	BC003566	18q12
212534_at	15	ZNF24	AU144066	18q12
213269_at	15	ZNF248	N21541	---
206900_x_at	14	ZNF253	NM_021047	19p13.11
213698_at	15	ZNF258	AI805560	1p34.3
200054_at	14	ZNF259	NM_003904	11q23.3
217185_s_at	10	ZNF259	Z95118	---
202050_s_at	15	ZNF262	AI650586	1p32-p34
203707_at	15	ZNF263	NM_005741	16p13.3
205917_at	14	ZNF264	NM_003417	19q13.4
214686_at	15	ZNF266	AA868898	---
219540_at	11	ZNF267	NM_003414	16p11.2
215239_x_at	14	ZNF273	AU132789	7q11.21
204937_s_at	15	ZNF274	NM_016325	19qter
218645_at	13	ZNF277	NM_021994	7q31.1
209494_s_at	14	ZNF278	AI807017	22q12.2
218401_s_at	15	ZNF281	AU150752	1q32.1 /// 1q32.1
211975_at	15	ZNF289	BE299671	11p11.2-p11.12
215848_at	10	ZNF291	AW139369	15q24
212366_at	15	ZNF292	BF223237	6q15
212368_at	15	ZNF292	AA972711	6q15
215596_s_at	15	ZNF294	AL163248	21q22.11
213081_at	12	ZNF297	AL523144	6p21.3
204180_s_at	14	ZNF297B	NM_014007	9p24.1-q22.33
204181_s_at	14	ZNF297B	T90308	9p24.1-q22.33
204182_s_at	13	ZNF297B	NM_014007	9p24.1-q22.33
218490_s_at	15	ZNF302	NM_018443	19q13.12
207753_at	12	ZNF304	NM_020657	19q13.4
200867_at	15	ZNF313	AL031685	20q13.13
200868_s_at	15	ZNF313	NM_018683	20q13.13
203521_s_at	14	ZNF318	NM_014345	6pter-p12.1
219376_at	15	ZNF322A	NM_024639	6p22.1
222016_s_at	15	ZNF323	AW086021	---
219765_at	12	ZNF329	NM_024620	19q13.43
209814_at	15	ZNF330	BC004421	---
219228_at	15	ZNF331	NM_018555	19q13.3-q13.4
78330_at	10	ZNF335	AA845577	20q11.21-q13.12
214760_at	10	ZNF337	AL049942	20p11.21
37860_at	15	ZNF337	AL049942	20p11.21
219801_at	15	ZNF34	NM_030580	8q24.3
206096_at	12	ZNF35	AI809774	3p22-p21
217715_x_at	15	ZNF354A	BE045142	---
219379_x_at	10	ZNF358	NM_018083	---
214670_at	15	ZNF36	AA653300	7q21.3-q22.1
212742_at	15	ZNF364	AL530462	1q21.2
212369_at	12	ZNF384	AI264312	---

218149_s_at	15	ZNF395	NM_017606	8p21.1
221123_x_at	12	ZNF395	NM_018660	8p21.1
221646_s_at	15	ZNF399	AF267859	5p15.33
218079_s_at	15	ZNF403	NM_024835	17q21.1
202010_s_at	15	ZNF410	NM_021188	14q24.3
205514_at	15	ZNF415	NM_018355	19q13.42
58367_s_at	15	ZNF419	AA429615	19q13.43
40569_at	15	ZNF42	M58297	---
214761_at	15	ZNF423	AW149417	16q12
206695_x_at	14	ZNF43	NM_003423	19p13.1-p12
206829_x_at	13	ZNF430	NM_025189	19p13.11
219848_s_at	11	ZNF432	NM_014650	19q13.41
218937_at	14	ZNF434	NM_017810	16p13.3
215359_x_at	15	ZNF44	AI758888	19p13.2
205928_at	13	ZNF443	NM_005815	19p13.2
50376_at	14	ZNF444	AI278629	19q13.43
218312_s_at	15	ZNF447	NM_023926	19q13.43
222028_at	15	ZNF45	AI967981	19q13.2
212557_at	15	ZNF451	AB011148	6p12.1
215012_at	11	ZNF451	AU144775	6p12.1
214746_s_at	15	ZNF467	BE549732	7q36.1
208119_s_at	14	ZNF505	NM_031218	19p13.11 /// 19p13.11
206053_at	11	ZNF510	NM_014930	9q22.33
203604_at	14	ZNF516	N38750	---
204291_at	14	ZNF518	NM_014803	10q24.1
215307_at	15	ZNF529	AL109722	---
220617_s_at	15	ZNF532	NM_018181	18q21.32
218735_s_at	15	ZNF544	NM_014480	19q13.43
219163_at	14	ZNF562	NM_017656	19p13.2
206648_at	12	ZNF571	NM_016536	19q13.13
217627_at	13	ZNF573	BE515346	19q13.13
219089_s_at	15	ZNF576	NM_024327	19q13.32
220748_s_at	14	ZNF580	NM_016202	19q13.43
219711_at	11	ZNF586	NM_017652	19q13.43
219981_x_at	15	ZNF587	NM_017961	---
204473_s_at	14	ZNF592	NM_014630	15q25.2
204175_at	15	ZNF593	NM_015871	1p35.3
219635_at	15	ZNF606	NM_025027	19q13.4
208137_x_at	10	ZNF611	NM_030972	19q13.42 /// 19q13.42
220721_at	14	ZNF614	NM_025040	19q13.41
213196_at	12	ZNF629	AI924293	---
213775_x_at	15	ZNF638	AI357871	2p13.2-p13.1
220760_x_at	15	ZNF665	NM_024733	19q13.42
218068_s_at	15	ZNF672	NM_024836	1q44
206583_at	15	ZNF673	NM_017776	Xp11.3
220661_s_at	15	ZNF692	NM_017865	1q44
205089_at	15	ZNF7	NM_003416	8q24
218059_at	15	ZNF706	NM_016096	8q22.3

213659_at	14	ZNF75	AA209420	Xq26.3
214813_at	13	ZNF75	W90796	Xq26.3
204453_at	15	ZNF84	NM_003428	12q24.33
206158_s_at	15	ZNF9	NM_003418	3q21
206059_at	13	ZNF91	NM_003430	19p13.1-p12
201541_s_at	15	ZNHIT1	NM_006349	7q22.1
213097_s_at	15	ZRF1	AI338837	7q22-q32
209431_s_at	12	ZSG	AF254083	22q12.2
204812_at	12	ZW10	NM_004724	11q23.3
204026_s_at	15	ZWINT	NM_007057	10q21-q22
202448_s_at	12	ZYG	BE675849	9q34.13
200808_s_at	15	ZYX	NM_003461	7q32
215706_x_at	15	ZYX	BC002323	7q32
