# Supplementary material for: Altered expression of mitochondrial and extracellular matrix genes in the heart of human fetuses with chromosome 21 trisomy
Source: BMC Genomics. 2007 Aug 7;8:268. doi: 10.1186/1471-2164-8-268 (PMC1964766; doi:10.1186/1471-2164-8-268)
Supplement: Additional file 2 — Hsa21 genes expressed in the human fetal heart at 18–22 weeks of gestation. Genes (probe sets) are sorted according to chromosomal location. Mitochondrial and ECM genes are in bold. [file 1471-2164-8-268-S2.pdf]

Table S2. Hsa21 genes expressed in the human fetal heart at 18-22 weeks of gestation.
Genes (probe sets) are sorted according to chromosomal location. Mitochondrial and ECM genes are in bold.

Probe ID	Gene Name	GenBank ID	Presence Call	Fold Change	p-value	Chromosomal localization
220330_s_at	SAMSN1	NM_022136	14	2.296	0.05930	21q11
202557_at	STCH	AI718418	15	2.011	0.05780	21q11.1
202558_s_at	STCH	NM_006948	14	1.564	0.05900	21q11.1
202599_s_at	NRIP1	NM_003489	15	1.451	0.14800	21q11.2
202600_s_at	NRIP1	AI824012	13	2.188	0.20700	21q11.2
220419_s_at	USP25	NM_013396	15	1.511	0.05530	21q11.2
202325_s_at	**ATP5J**	NM_001685	15	1.003	0.97100	21q21.1
213134_x_at	BTG3	AI765445	15	1.595	0.01340	21q21.1
205548_s_at	BTG3	NM_006806	15	1.685	0.00788	21q21.1
215425_at	BTG3	AL049332	14	1.236	0.39400	21q21.1
220941_s_at	C21orf91	NM_017447	10	1.343	0.13900	21q21.1
203917_at	CXADR	NM_001338	15	1.039	0.86900	21q21.1
205669_at	NCAM2	NM_004540	11	1.24	0.34800	21q21.1
222162_s_at	**ADAMTS1**	AK023795	15	1.93	0.01350	21q21.2
214953_s_at	**APP**	X06989	15	1.668	0.05310	21q21.2
211277_x_at	**APP**	BC004369	15	1.166	0.29500	21q21.2
200602_at	**APP**	NM_000484	15	1.479	0.20400	21q21.2
219213_at	JAM2	NM_021219	15	1.939	0.00165	21q21.2
220311_at	C21orf127	NM_013240	12	1.358	0.18900	21q21.3
221158_at	C21orf66	NM_013329	15	1.277	0.00464	21q21.3
218515_at	C21orf66	NM_016631	15	1.289	0.08500	21q21.3
219767_s_at	CRYZL1	NM_005111	15	1.512	0.00074	21q21.3
219935_at	HEMK2	NM_007038	15	1.5	0.02970	21q21.3
218558_s_at	**MRPL39**	NM_017446	15	1.318	0.04160	21q21.3
218123_at	C21orf59	NM_017835	15	1.242	0.24100	21q22.1
221677_s_at	DONSON	AF232674	15	1.2	0.07860	21q22.1
212378_at	GART	NM_000819	15	1.279	0.08630	21q22.1
210005_at	GART	D32051	12	0.847	0.24400	21q22.1
204786_s_at	IFNAR2	L41944	10	2.263	0.21400	21q22.1
200642_at	SOD1	NM_000454	15	1.163	0.14500	21q22.1
213135_at	TIAM1	U90902	14	1.968	0.04440	21q22.1
216954_x_at	**ATP5O**	S77356	15	1.344	0.07020	21q22.11
200818_at	**ATP5O**	NM_001697	15	1.03	0.68300	21q22.11
204194_at	BACH1	NM_001186	15	1.524	0.16100	21q22.11
212996_s_at	C21orf108	AI803485	11	1.169	0.43900	21q22.11
219004_s_at	C21orf45	NM_018944	15	1.227	0.30800	21q22.11
200873_s_at	CCT8	NM_006585	15	1.347	0.14900	21q22.11
201642_at	IFNGR2	NM_005534	15	1.476	0.00623	21q22.11
209575_at	IL10RB	BC001903	15	1.332	0.00233	21q22.11
209298_s_at	ITSN1	AF114488	13	1.535	0.13200	21q22.11
209297_at	ITSN1	AF114488	11	1.625	0.10500	21q22.11
35776_at	ITSN1	AF064243	15	1.722	0.00033	21q22.11
213164_at	**MRPS6**	AI867198	15	1.127	0.56600	21q22.11
212944_at	**MRPS6**	AK024896	15	1.663	0.21800	21q22.11
214988_s_at	SON	X63071	15	1.462	0.01210	21q22.11
213538_at	SON	AI936458	15	1.367	0.03200	21q22.11
201086_x_at	SON	NM_003103	15	1.423	0.03650	21q22.11
219600_s_at	TMEM50B	BC000569	15	1.25	0.11400	21q22.11
218386_x_at	USP16	AI806796	15	1.454	0.04300	21q22.11
215596_s_at	ZNF294	AL163248	15	1.328	0.16000	21q22.11
208370_s_at	DSCR1	NM_004414	15	1.245	0.49600	21q22.12
208514_at	KCNE1	NM_000219	12	1.224	0.14600	21q22.12
209033_s_at	DYRK1A	D86550	15	1.528	0.00454	21q22.13
213000_at	MORK3	AP000693	14	1.494	0.11000	21q22.13
213989_x_at	SETD4	AB004853	10	2.472	0.19500	21q22.13
203635_at	DSCR3	NM_006052	15	0.978	0.89300	21q22.2

205549_at	PCP4	NM_006198	15	1.195	0.38400	21q22.2
221689_s_at	PIGP	AB035745	15	1.475	0.00037	21q22.2
217269_s_at	PRSS7	AP001672	11	1.008	0.97300	21q22.2
212990_at	SINJ1	AB020717	15	1.147	0.28500	21q22.2
208662_s_at	TTC3	AI885338	15	1.541	0.01890	21q22.2
208661_s_at	TTC3	D84294	15	2.969	0.03570	21q22.2
208073_x_at	TTC3	NM_003316	15	1.635	0.00780	21q22.2
208663_s_at	TTC3	AI652848	14	2.754	0.06680	21q22.2
204567_s_at	ABCG1	NM_004915	15	1.613	0.06180	21q22.3
211113_s_at	ABCG1	U34919	14	1.503	0.01900	21q22.3
203865_s_at	ADARB1	NM_015833	11	1.541	0.01100	21q22.3
217867_x_at	BACE2	AF178532	15	1.61	0.02880	21q22.3
212875_s_at	C21orf25	AP001745	11	1.282	0.33000	21q22.3
212816_s_at	CBS	BE613178	12	1.01	0.96000	21q22.3
209082_s_at	**COL18A1**	AF018081	15	1.406	0.06460	21q22.3
209081_s_at	**COL18A1**	AF018081	15	1.57	0.00786	21q22.3
213428_s_at	**COL6A1**	AA292373	15	1.618	0.00371	21q22.3
212091_s_at	**COL6A1**	AI141603	10	1.927	0.04930	21q22.3
213290_at	**COL6A2**	AL531750	15	1.443	0.03480	21q22.3
209156_s_at	**COL6A2**	AY029208	15	2.391	0.00022	21q22.3
201201_at	CSTB	NM_000100	15	1.174	0.22900	21q22.3
215529_x_at	DIP2A	AI590053	12	1.29	0.53500	21q22.3
203405_at	DSCR2	NM_003720	15	1.15	0.35600	21q22.3
213541_s_at	ERG	AI351043	15	1.405	0.03210	21q22.3
201329_s_at	ETS2	NM_005239	15	1.089	0.45300	21q22.3
201328_at	ETS2	AL575509	15	1.563	0.11100	21q22.3
222303_at	ETS2	AV700891	14	1.571	0.09150	21q22.3
200944_s_at	HMGN1	NM_004965	15	1.347	0.00173	21q22.3
200943_at	HMGN1	NM_004965	15	1.558	0.00851	21q22.3
221564_at	HRMT1L1	AL570294	15	1.496	0.00233	21q22.3
202098_s_at	HRMT1L1	NM_001535	15	1.177	0.23900	21q22.3
208579_x_at	HSF2BP	NM_017445	13	1.642	0.00717	21q22.3
202803_s_at	**ITGB2**	NM_000211	13	1.052	0.59700	21q22.3
212846_at	KIAA0179	AA811192	14	1.428	0.01200	21q22.3
202217_at	**KNP-I**	NM_004649	15	1.407	0.18600	21q22.3
210667_s_at	**KNP-I**	D86062	15	1.086	0.79200	21q22.3
202245_at	LSS	AW084510	15	1.205	0.16700	21q22.3
212269_s_at	MCM3AP	AJ010089	15	1.369	0.06630	21q22.3
220459_at	MCM3APAS	NM_018118	14	0.874	0.41100	21q22.3
202086_at	MX1	NM_002462	12	1.04	0.91300	21q22.3
204994_at	MX2	NM_002463	15	0.84	0.30500	21q22.3
218019_s_at	PDXK	AW449022	15	1.119	0.49400	21q22.3
218018_at	PDXK	AW449022	15	0.758	0.52200	21q22.3
201102_s_at	PFKL	NM_002626	15	1.2	0.05060	21q22.3
211065_x_at	PFKL	BC006422	15	1.419	0.00882	21q22.3
54051_at	PKNOX1	H59033	13	1.201	0.20800	21q22.3
221883_at	PKNOX1	AA133342	12	1.321	0.22400	21q22.3
209578_s_at	POFUT2	BC000626	15	1.231	0.06320	21q22.3
200677_at	PTTG1IP	NM_004339	15	1.224	0.11300	21q22.3
204979_s_at	SH3BGR	NM_007341	15	1.278	0.22200	21q22.3
200740_s_at	SUMO3	BG338532	15	1.357	0.01830	21q22.3
200739_s_at	SUMO3	BG338532	15	1.215	0.33900	21q22.3
204623_at	TFF3	NM_003226	14	1.609	0.06070	21q22.3
202858_at	U2AF1	NM_006758	15	1.253	0.28100	21q22.3
209042_s_at	UBE2G2	BC001738	15	1.191	0.43400	21q22.3
209041_s_at	UBE2G2	BG395660	11	1.444	0.22100	21q22.3
202749_at	WRB	NM_004627	15	1.175	0.27900	21q22.3
