# Supplementary figures and images for: Altered expression of mitochondrial and extracellular matrix genes in the heart of human fetuses with chromosome 21 trisomy
Source: BMC Genomics. 2007 Aug 7;8:268. doi: 10.1186/1471-2164-8-268 (PMC1964766; doi:10.1186/1471-2164-8-268)

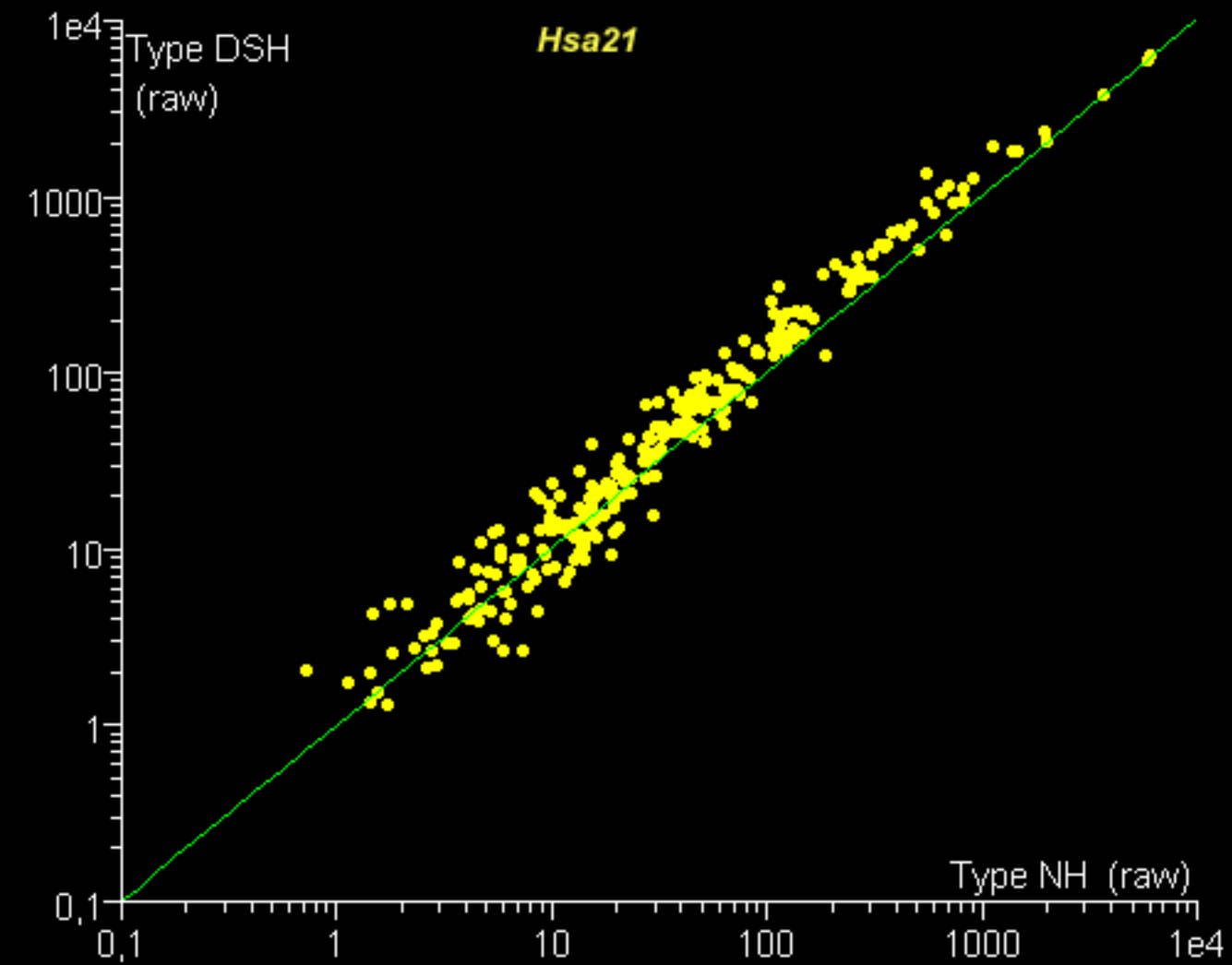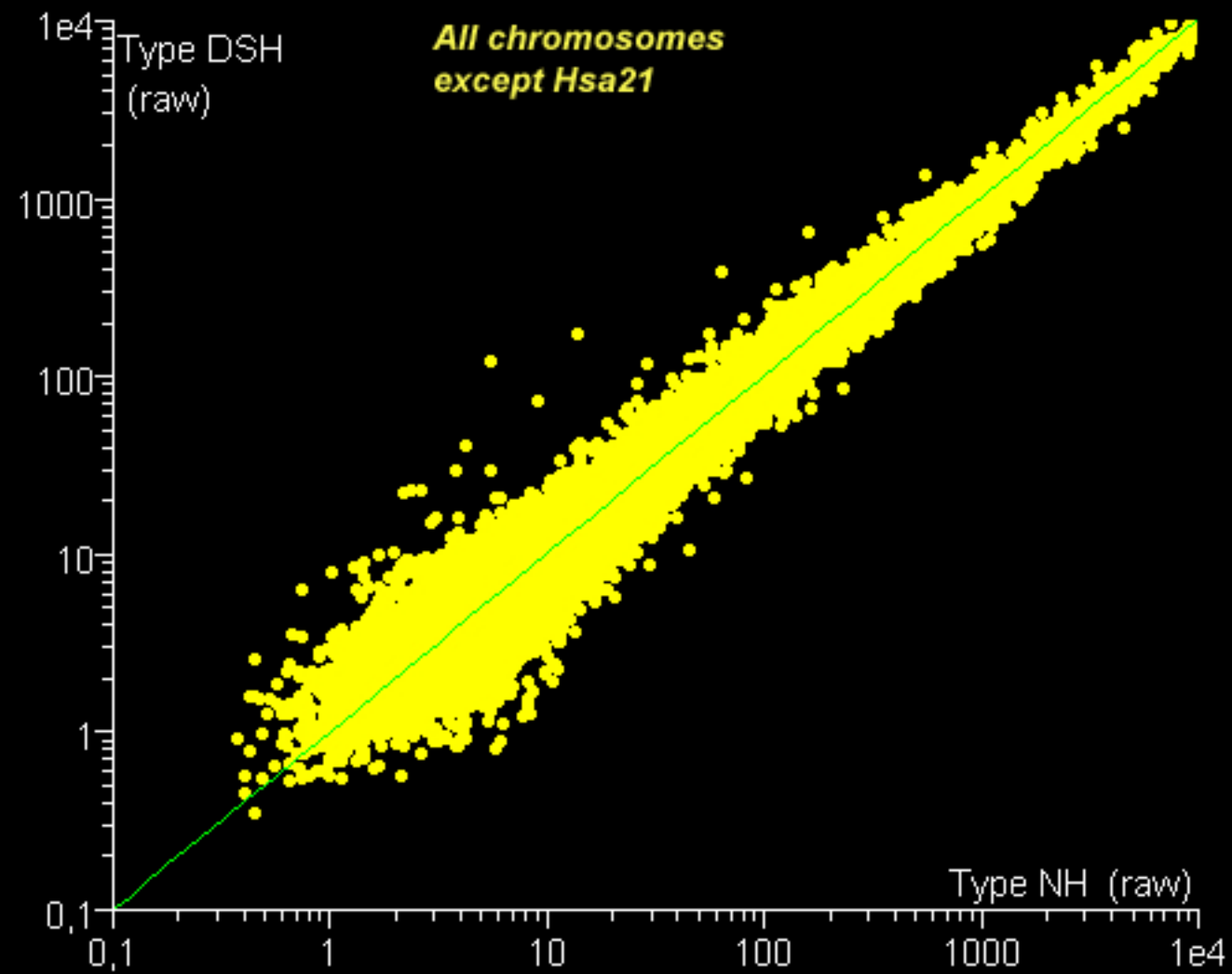

Supplement: Additional file 3 — Scatter plot of gene expression data of trisomic samples vs. control samples. Mean raw, log transformed, gene expression data from the 5 control samples (NH) were plotted on the x-axis and data from the 10 trisomic samples (DSH) were plotted on the y-axis. Plots are shown for Hsa21 genes and for genes of all chromosomes excluding Hsa21. In the plot of Hsa21 more than 75% of gene probe sets are above the line, whereas in the plot of all other chromosomes approximately the same number of gene probe sets is above and below the line. Abbreviations for DSH and NH are as in Figure 1. [file 1471-2164-8-268-S3.pdf]
