# Supplementary material for: Altered expression of mitochondrial and extracellular matrix genes in the heart of human fetuses with chromosome 21 trisomy
Source: BMC Genomics. 2007 Aug 7;8:268. doi: 10.1186/1471-2164-8-268 (PMC1964766; doi:10.1186/1471-2164-8-268)
Supplement: Additional file 4 — Genes differentially expressed between trisomic and control samples. The table includes genes with fold change > |1.2| and p < 0.05 (ANOVA test). Genes are sorted according to fold change. [file 1471-2164-8-268-S4.pdf]

Table S3. Genes differentially expressed between trisomic and control samples.

The table includes genes with fold change > |1.2| and p<0.05 (ANOVA test). Genes are sorted according to fold change,

Probe ID	Gene Name	GenBank ID	Fold Change	p-value	Chromosomal location
211560_s_at	ALAS2	AF130113	2.901	0.0445	Xp11.21
221795_at	NTRK2	AA707199	2.854	0.0283	9q22.1
222106_at	PRND	AL133396	2.831	0.0042	20pter-p12
205608_s_at	ANGPT1	U83508	2.467	0.004	8q22.3-q23
222288_at	PPP4R2	AI004009	2.44	0.0516	3p13
209535_s_at	AKAP13	AF127481	2.405	0.0153	15q24-q25
209156_s_at	COL6A2	AY029208	2.391	0.0002	21q22.3
204606_at	CCL21	NM_002989	2.263	0.0057	9p13
201668_x_at	MARCKS	AW163148	2.182	0.0162	6q22.2
201497_x_at	MYH11	NM_022844	2.168	0.0054	16p13.13-p13.12
205593_s_at	PDE9A	NM_002606	2.052	0.0067	21q22.3
201830_s_at	NET1	NM_005863	1.985	0.0435	10p15
205923_at	RELN	NM_005045	1.97	0.0029	7q22
213135_at	TIAM1	U90902	1.968	0.0444	21q22.1
214433_s_at	SELENBP1	NM_003944	1.965	0.0089	1q21-q22
212385_at	TCF4	AU118026	1.951	0.0481	18q21.1
217525_at	OLFML1	AW305097	1.942	0.0072	11p15.4
219213_at	JAM2	NM_021219	1.939	0.0017	21q21.2
222162_s_at	ADAMTS1	AK023795	1.93	0.0135	21q21.2
212091_s_at	COL6A1	AI141603	1.927	0.0493	21q22.3
209541_at	IGF1	AI972496	1.888	0.0336	12q22-q23
213094_at	GPR126	AL033377	1.881	0.02	6q24.1
205592_at	SLC4A1	NM_000342	1.853	0.0294	17q21-q22
219087_at	ASPN	NM_017680	1.829	0.0007	9q22
210198_s_at	PLP1	BC002665	1.787	0.0086	Xq22
205168_at	DDR2	NM_006182	1.769	0.04	1q12-q23
208037_s_at	MADCAM1	NM_007164	1.759	0.0079	19p13.3
209840_s_at	LRRN3	AI221950	1.754	0.0182	7q31.1
209242_at	PEG3	AL042588	1.752	0.0109	19q13.4
221582_at	HIST3H2A	BC001193	1.747	0.0001	1q42.13
206461_x_at	MT1H	NM_005951	1.741	0.0493	16q13
215735_s_at	TSC2	AC005600	1.734	0.0517	16p13.3
209335_at	DCN	BC005322	1.73	0.0165	12q13.2
203213_at	CDC2	AL524035	1.727	0.0422	10q21.1
35776_at	ITSN1	AF064243	1.722	0.0003	21q22.1-q22.2
202994_s_at	FBLN1	Z95331	1.716	0.0271	22q13.31
206023_at	NMU	NM_006681	1.715	0.0373	4q12
208850_s_at	THY1	AL558479	1.71	0.0263	11q22.3-q23
210510_s_at	NRP1	AF145712	1.701	0.0234	10p12
203502_at	BPGM	NM_001724	1.698	0.0147	7q31-q34
209121_x_at	NR2F	M64497	1.686	0.0224	15q26
213905_x_at	BGN	AA845258	1.685	0.0099	18q22.3
205548_s_at	BTG3	NM_006806	1.685	0.0079	21q21.1-q21.2
209220_at	GPC3	L47125	1.685	0.0032	Xq26.1
212692_s_at	LRBA	W60686	1.684	0.0153	4q31.23
203423_at	RBP1	NM_002899	1.677	0.0063	3q23
214953_s_at	APP	X06989	1.668	0.0531	21q21.2

203758_at	CTSO	AV729484	1.668	0.0362	4q31-q32
213125_at	OLFML2B	AW007573	1.666	0.0187	1q23.1
218162_at	OLFML3	NM_020190	1.659	0.0272	1p13.1
203868_s_at	VCAM1	NM_001078	1.652	0.0383	1p32-p31
214724_at	DIXDC1	AF070621	1.646	0.0236	11q23.1
208579_x_at	H2BFS	NM_017445	1.642	0.0072	21q22.3
208073_x_at	TTC3	NM_003316	1.635	0.0078	21q22.2
207173_x_at	CDH11	D21254	1.633	0.0147	16q22.1
200974_at	ACTA2	NM_001613	1.631	0.019	10q23.3
203883_s_at	RAB11FIP2	BG249608	1.63	0.0199	10q26.12
204745_x_at	MT1G	NM_005950	1.62	0.0423	16q13
204403_x_at	KIAA0738	NM_014719	1.619	0.0081	7q35
212312_at	COX4I2	AL117381	1.617	0.0102	20q11.21
201995_at	EXT1	NM_000127	1.615	0.0518	8q24.11-q24.13
217867_x_at	BACE2	AF178532	1.61	0.0288	21q22.3
221045_s_at	PER3	NM_016831	1.601	0.0157	1p36.23
202403_s_at	COL1A2	NM_000089	1.596	0.0192	7q22.1
212489_at	COL5A1	AI983428	1.595	0.0345	9q34.2-q34.3
202202_s_at	LAMA4	NM_002290	1.591	0.0488	6q21
202016_at	MEST	NM_002402	1.59	0.0385	7q32
209735_at	ABCG2	AF098951	1.587	0.0003	4q22
201069_at	MMP2	NM_004530	1.586	0.0069	16q13-q21
209081_s_at	COL18A1	AF018081	1.57	0.0079	21q22.3
201312_s_at	SH3BGRL	NM_003022	1.559	0.005	Xq13.3
200943_at	HMGN1	NM_004965	1.558	0.0085	21q22.3
205131_x_at	CLEC11A	NM_002975	1.556	0.0129	19q13.3
214439_x_at	BIN1	AF043899	1.556	0.0499	2q14
206101_at	ECM2	NM_001393	1.554	0.0079	9q22.3
205151_s_at	KIAA0644	AV724192	1.547	0.0182	7p15.1
202310_s_at	COL1A1	K01228	1.546	0.0333	17q21.3-q22.1
204785_x_at	IFNAR2	L41944	1.545	0.0002	21q22.1
203865_s_at	ADARB1	NM_015833	1.541	0.011	21q22.3
209656_s_at	TMEM47	AL136550	1.54	0.0171	Xp11.4
65588_at	LOC388796	AA827892	1.536	0.0098	20q12
209033_s_at	DYRK1A	D86550	1.528	0.0045	21q22.13
201525_at	APOD	NM_001647	1.526	0.0041	3q26.2-qter
204359_at	FLRT2	NM_013231	1.522	0.0295	14q24-q32
213661_at	DKFZP586H2123	AI671186	1.516	0.0451	11p13
205413_at	MPPED2	NM_001584	1.513	0.0012	11p13
219767_s_at	CRYZL1	NM_005111	1.512	0.0007	21q21.3
204505_s_at	EPB49	NM_001978	1.505	0.0078	8p21.1
211113_s_at	ABCG1	U34919	1.503	0.019	21q22.3
219935_at	ADAMTS5	NM_007038	1.5	0.0297	21q21.3
221564_at	PRMT2	AL570294	1.496	0.0023	21q22.3
201417_at	SOX4	AL136179	1.491	0.0346	6p22.3
213791_at	PENK	NM_006211	1.49	0.041	8q23-q24
201431_s_at	DPYSL3	NM_001387	1.487	0.0046	5q32
221447_s_at	GLT8D2	NM_031302	1.485	0.051	12q
204112_s_at	HNMT	NM_006895	1.483	0.0155	2q22.1
217764_s_at	RAB31	AF183421	1.482	0.0489	18p11.3
203896_s_at	PLCB4	AL535113	1.482	0.0175	20p12
202465_at	PCOLCE	NM_002593	1.482	0.0333	7q22

204462_s_at	SLC16A2	NM_006517	1.482	0	Xq13.2
203185_at	RASSF2	NM_014737	1.481	0.0546	20pter-p12.1
218585_s_at	RAMP	AK001261	1.48	0.0342	1q32.3
213296_at	PEX10	BF339133	1.48	0.0458	1pter-q24
201642_at	IFNGR2	NM_005534	1.476	0.0062	21q22.11
221689_s_at	PIGP	AB035745	1.475	0.0004	21q22.2
214730_s_at	GLG1	AK025457	1.471	0.0078	16q22-q23
221249_s_at	FAM117A	NM_030802	1.47	0.0083	17q21.33
204759_at	RCBTB2	NM_001268	1.462	0.0206	13q14.3
214988_s_at	SON	X63071	1.462	0.0121	21q22.1-q22.2
221814_at	GPR124	BF511315	1.462	0.0512	8p11.23
209560_s_at	DLK1	U15979	1.458	0.0472	14q32
204556_s_at	DZIP1	AL568422	1.456	0.0401	13q32.1
211546_x_at	NACP	L36674	1.456	0.0425	4q21
213392_at	IQCK	AW070229	1.455	0.0503	16p13.11
218386_x_at	USP16	AI806796	1.454	0.043	21q22.11
201427_s_at	SEPP1	NM_005410	1.452	0.0423	5q31
202242_at	TSPAN7	NM_004615	1.449	0.0137	Xp11.4
213074_at	IRAK1BP1	BG545769	1.444	0.007	6q14-q15
203454_s_at	ATOX1	NM_004045	1.441	0.0264	5q32
208818_s_at	COMT	BC000419	1.44	0.0357	22q11.21-q11.23
207827_x_at	SNCA	NM_007308	1.439	0.0364	4q21
210715_s_at	KOP	AF027205	1.436	0.0291	19q13.1
211161_s_at	COL3A1	AF130082	1.434	0.0423	2q31
203417_at	MFAP2	NM_017459	1.434	0.0491	1p36.1-p35
208407_s_at	CTNND1	NM_001331	1.433	0.0193	11q11
212846_at	KIAA0179	AA811192	1.428	0.012	21q22.3
203562_at	FEZ1	NM_005103	1.426	0.0324	11q24.2
213685_at	TCEB3	AA830143	1.426	0.0429	1p36.1
204037_at	EDG2	BF055366	1.426	0.0168	9q32
204354_at	POT1	NM_015450	1.425	0.0158	7q31.33
215157_x_at	PABPC1	AI734929	1.423	0.0127	8q22.2-q23
211065_x_at	PFKL	BC006422	1.419	0.0088	21q22.3
203477_at	COL15A1	NM_001855	1.413	0.0349	9q21-q22
211675_s_at	MDFIC	AF054589	1.41	0.0537	7q31.2
218609_s_at	NUDT2	NM_001161	1.41	0.0301	9p13
212762_s_at	TCF7L2	AI375916	1.409	0.0302	10q25.3
212970_at	APBB2	AI694303	1.409	0.0106	4p14
213541_s_at	ERG	AI351043	1.405	0.0321	21q22.3
203449_s_at	TERF1	NM_017489	1.402	0.0167	8q13
212337_at	TUG1	AI687738	1.393	0.0088	22q12.2
219049_at	ChGn	NM_018371	1.393	0.0109	8p21.3
205433_at	BCHE	NM_000055	1.388	0.049	3q26.1-q26.2
209897_s_at	SLIT2	AF055585	1.383	0.0424	4p15.2
206766_at	ITGA10	AF112345	1.378	0.0354	1q21
221476_s_at	EC45	AF279903	1.378	0.0316	3p24.1
205236_x_at	SOD3	NM_003102	1.372	0.0095	4p16.3-q21
200986_at	SERPING1	NM_000062	1.369	0.0466	11q12-q13.1
209707_at	PIGK	AF022913	1.363	0.0102	1p31.1
211955_at	RANBP5	NM_002271	1.357	0.011	13q32.2
200740_s_at	SUMO3	BG338532	1.357	0.0183	21q22.3
201814_at	TBC1D5	NM_014744	1.345	0.0236	3p24.3

214273_x_at	C16orf35	AV704353	1.343	0.0395	16p13.3
219025_at	CD248	NM_020404	1.342	0.0382	11q13
213750_at	CSIG	AA928506	1.338	0.0391	16p13.13
209575_at	IL10RB	BC001903	1.332	0.0023	21q22.1-q22.2
212698_s_at	SEPT10	BF966021	1.331	0.0411	2q13
209428_s_at	ZFPL1	BG420865	1.328	0.0031	11q13
213881_x_at	SUMO2	AI971724	1.325	0.0247	17q25
201719_s_at	EPB41L2	NM_001431	1.324	0.0525	6q23
201292_at	TOP2A	AL561834	1.322	0.0023	17q21-q22
214746_s_at	ZNF467	BE549732	1.32	0.0429	7q36.1
208779_x_at	DDR1	L20817	1.319	0.0071	6p21.3
218558_s_at	MRPL39	NM_017446	1.318	0.0416	21q21.3
204400_at	EFS	NM_005864	1.315	0.0436	14q11.2-q12
202643_s_at	TNFAIP3	AI738896	1.31	0.0275	6q23
205011_at	LOH11CR2A	NM_014622	1.306	0.0096	11q23
201778_s_at	KIAA0494	NM_014774	1.305	0.0205	1pter-p22.1
204450_x_at	APOA1	NM_000039	1.3	0.0308	11q23-q24
212372_at	NMMHCB	AK026977	1.3	0.0432	17p13
41047_at	C9orf16	AI885170	1.299	0.054	9q34.1
201965_s_at	SETX	NM_015046	1.299	0.0308	9q34.3
47069_at	PRR5	AA533284	1.292	0.0118	22q13.31
200003_s_at	RPL28	NM_000991	1.29	0.0448	19q13.4
201494_at	PRCP	NM_005040	1.289	0.0017	11q14
202075_s_at	PLTP	NM_006227	1.289	0.019	20q12-q13.1
202355_s_at	GTF2F1	BC000120	1.287	0.0366	19p13.3
202974_at	MPP1	NM_002436	1.287	0.0349	Xq28
201590_x_at	ANXA2	NM_004039	1.286	0.0502	15q21-q22
218017_s_at	FLJ32731	NM_025070	1.278	0.0348	8p11.1
212565_at	STK38L	BE302191	1.274	0.0467	12p12.1
200096_s_at	ATP6V0E	AV717561	1.27	0.0086	5q35.2
218870_at	ARHGAP15	NM_018460	1.263	0.0108	2q22.3
216381_x_at	AKR7A2	AL035413	1.256	0.0156	1p36.13
209263_x_at	TSPAN4	BC000389	1.252	0.0286	11p15.5
218927_s_at	CHST12	BC002918	1.251	0.0484	7p22
205176_s_at	ITGB3BP	NM_014288	1.25	0.0186	1p31.3
200023_s_at	EIF3S5	AI001896	1.246	0.0475	11p15.4
204568_at	KIAA0831	NM_014924	1.246	0.0296	14q22.2
203473_at	SLCO2B1	NM_007256	1.244	0.0068	11q13
209168_at	GPM6B	AW148844	1.242	0.0497	Xp22.2
203028_s_at	CYBA	NM_000101	1.231	0.0503	16q24
218253_s_at	LGTN	NM_006893	1.227	0.0274	1q31-q32
219175_s_at	SLC41A3	NM_017836	1.21	0.0126	3q21.2
212658_at	LHFPL2	N66633	1.203	0.0253	5q14.1
204571_x_at	PIN4	BE797213	-1.205	0.0272	Xq13
217774_s_at	HSPC152	NM_016404	-1.209	0.055	11q13.1
215416_s_at	STOML2	AC004472	-1.211	0.0525	9p13.1
215380_s_at	C7orf24	AK021779	-1.212	0.0119	7p15-p14
203669_s_at	DGAT1	NM_012079	-1.214	0.0445	8q24.3
212369_at	ZNF384	AI264312	-1.215	0.0322	12p13.31
202739_s_at	PHKB	NM_000293	-1.217	0.0516	16q12-q13
217950_at	NOSIP	NM_015953	-1.217	0.0501	19q13.33
206621_s_at	WBSCR1	NM_022170	-1.217	0.0497	7q11.23

218830_at	RPL26L1	NM_016093	-1.218	0.0394	5q35.2
202618_s_at	MeCP-2	L37298	-1.220	0.0381	Xq28
205277_at	PRDM2	NM_012231	-1.221	0.0321	1p36
201186_at	LRPAP1	NM_002337	-1.225	0.0237	4p16.3
204097_s_at	RBMX2	AF078865	-1.225	0.0262	Xq26.1
201584_s_at	DDX39	NM_005804	-1.229	0.0199	19p13.13
215088_s_at	RPA1	BG110532	-1.230	0.0456	17p13.3
209165_at	AATF	AF083208	-1.230	0.0168	17q11.2-q12
205963_s_at	DNAJA3	NM_005147	-1.232	0.0275	16p13.3
209080_x_at	TXNL2	AF118652	-1.232	0.0384	6p25.3
209075_s_at	ISCU	AY009128	-1.233	0.0035	12q24.1
209514_s_at	RAB27A	BE502030	-1.233	0.0186	15q21.3
219394_at	PGS1	NM_024419	-1.233	0.0387	17q25.3
207922_s_at	MAEA	NM_005882	-1.233	0.0476	4p16.3
209796_s_at	TMEM4	BC001027	-1.235	0.0499	12q15
218497_s_at	RNASEH1	BG534527	-1.238	0.0403	2p25
213702_x_at	ASAH1	AI934569	-1.242	0.0125	8p22-p21.3
214042_s_at	MDS1	AW071997	-1.244	0.0436	3q26.2
203103_s_at	PRPF19	NM_014502	-1.245	0.0326	11q12.2
221736_at	KIAA1219	BG236163	-1.245	0.0414	20q12
210273_at	PCDH7	AB006757	-1.245	0.0261	4p15
203406_at	MFAP1	NM_005926	-1.247	0.0194	15q15-q21
203517_at	MTX2	NM_006554	-1.247	0.0059	2q31.2
212852_s_at	TROVE2	AL538601	-1.248	0.0414	1q31
213398_s_at	HCDI	AI347090	-1.250	0.0362	14q11.2
209331_s_at	MAX	AA723514	-1.250	0.0485	14q23
200803_s_at	TEGT	NM_003217	-1.252	0.0505	12q12-q13
217795_s_at	TMEM43	NM_024334	-1.252	0.0273	3p25.1
209856_x_at	ABI2	U31089	-1.253	0.0137	2q33
205562_at	RPP38	NM_006414	-1.255	0.0359	10p13
219185_at	SIRT5	NM_012241	-1.255	0.029	6p23
208749_x_at	FLOT1	AA507012	-1.256	0.041	6p21.3
213011_s_at	TPI1	BF116254	-1.259	0.0091	12p13.31
202487_s_at	H2AFV	NM_012412	-1.259	0.0039	7p13
205412_at	ACAT1	NM_000019	-1.261	0.0357	11q22.3-q23.1
209389_x_at	DBI	M15887	-1.261	0.0262	2q12-q21
203663_s_at	COX5A	NM_004255	-1.263	0.04	15q25
204030_s_at	SCHIP1	NM_014575	-1.267	0.0381	3q25.33
218686_s_at	RHBDF1	NM_022450	-1.269	0.0159	16p13.3
212053_at	KIAA0251	AK025504	-1.271	0.0363	16p13.13
200947_s_at	GLUD1	NM_005271	-1.272	0.0269	10q23.3
203613_s_at	NDUFB6	NM_002493	-1.272	0.0318	9p13.3
204232_at	FCER1G	NM_004106	-1.274	0.0289	1q23
209345_s_at	PI4KII	AL561930	-1.276	0.0454	10q24
208687_x_at	HSC70	AF352832	-1.277	0.0247	11q24.1
218307_at	RSAD1	NM_018346	-1.277	0.0257	17q21.33
205633_s_at	ALAS1	NM_000688	-1.277	0.0113	3p21.1
211318_s_at	RAE1	U85943	-1.279	0.006	20q13.31
219244_s_at	MRPL46	NM_022163	-1.279	0.0388	15q24-q25
43977_at	TMEM161A	AI660497	-1.282	0.0414	19p13.11
210014_x_at	IDH3B	AF023266	-1.284	0.0154	20p13
202121_s_at	CHMP2A	NM_014453	-1.287	0.0155	19q

201937_s_at	DNPEP	NM_012100	-1.287	0.023	2q36.1
218970_s_at	CUTC	NM_015960	-1.289	0.0405	10q24.31
201517_at	NCBP2	BC001255	-1.289	0.0163	3q29
208909_at	UQCRFS1	BC000649	-1.292	0.0242	19q12-q13.1
218093_s_at	ANKRD10	NM_017664	-1.295	0.0356	13q34
50376_at	ZNF444	AI278629	-1.295	0.011	19q13.43
202427_s_at	BRP44	NM_015415	-1.295	0.0528	1q24
207515_s_at	POLR1C	NM_004875	-1.297	0.0126	6p21.1
219940_s_at	FLJ11305	NM_018386	-1.299	0.0331	13q34
209142_s_at	UBE2G1	BC002775	-1.299	0.0465	1q42
213838_at	NOL7	AA191426	-1.299	0.0258	6p23
218022_at	VRK3	NM_016440	-1.300	0.0317	19q13
221264_s_at	TARDBP	NM_031214	-1.300	0.0495	1p36.22
212825_at	PAXIP1	AI357401	-1.300	0.0404	7q36
211784_s_at	SFRS1	BC006181	-1.304	0.0263	17q22
218996_at	TFPT	NM_013342	-1.305	0.0017	19q13
218068_s_at	ZNF672	NM_024836	-1.305	0.0173	1q44
203858_s_at	COX10	NM_001303	-1.307	0.0101	17p12-17p11.2
201903_at	UQCRC1	NM_003365	-1.307	0.0142	3p21.3
202607_at	NDST1	AL526632	-1.307	0.0371	5q33.1
211366_x_at	IL1BCE	U13698	-1.309	0.0191	11q23
218190_s_at	UCRC	NM_013387	-1.309	0.0017	22cen-q12.3
53987_at	RANBP10	AL041852	-1.311	0.0145	16q22.1
218682_s_at	SLC4A1AP	NM_018158	-1.311	0.0128	2p23.3
221897_at	TRIM52	AA205660	-1.311	0.0134	5q35.3
201740_at	NDUFS3	NM_004551	-1.314	0.0294	11p11.11
216977_x_at	SNRPA1	AJ130972	-1.314	0.0464	15q26.3
201035_s_at	HADH	BC000306	-1.314	0.0273	4q22-q26
202757_at	COBRA1	NM_015456	-1.316	0.0107	9q34
200078_s_at	ATP6V0B	BC005876	-1.318	0.0542	1p32.3
203113_s_at	EEF1D	NM_001960	-1.321	0.0475	8q24.3
202154_x_at	TUBB3	NM_006086	-1.325	0.0521	16q24.3
221506_s_at	TNPO2	BG258639	-1.325	0.0338	19p13.2
222025_s_at	OPLAH	AI991887	-1.325	0.0335	8q24.3
207945_s_at	CSNK1D	NM_001893	-1.326	0.0135	17q25
217961_at	FLJ20551	NM_017875	-1.326	0.0423	3p21.33
207302_at	SGCG	NM_000231	-1.330	0.0325	13q12
220934_s_at	MGC3196	NM_024084	-1.333	0.0142	11q12.3
203781_at	MRPL33	NM_004891	-1.333	0.0318	2p21
209808_x_at	ING1	AW193656	-1.335	0.0058	13q34
212694_s_at	PCCB	NM_000532	-1.335	0.0462	3q21-q22
37549_g_at	PTHB1	U87408	-1.335	0.0514	7p14
217918_at	DYNLRB1	NM_014183	-1.337	0.0338	20q11.21
218024_at	BRP44L	NM_016098	-1.339	0.0129	6q27
219347_at	NUDT15	NM_018283	-1.342	0.0318	13q14.12
217722_s_at	NGRN	NM_016645	-1.342	0.0363	15q26.1
203606_at	NDUFS6	NM_004553	-1.342	0.0464	5p15.33
213133_s_at	GCSH	AW237404	-1.346	0.0209	1q23.3
219588_s_at	NCAPG2	NM_017760	-1.346	0.0404	7q36.3
203814_s_at	NQO2	NM_000904	-1.348	0.0216	6p25.2
203647_s_at	FDX1	NM_004109	-1.350	0.0178	11q22
221255_s_at	TMEM93	NM_031298	-1.350	0.0301	17p13.3

201239_s_at	SPCS2	NM_014752	-1.351	0.048	11q13.3
218661_at	FLJ14154	NM_024845	-1.351	0.046	16p13.3
203827_at	WIPI1	NM_017983	-1.351	0.046	17q24.3
200793_s_at	ACO2	NM_001098	-1.351	0.0231	22q11.2-q13.31
220966_x_at	ARPC5L	NM_030978	-1.351	0.0333	9q34.11
202734_at	TRIP10	NM_004240	-1.353	0.0476	19p13.3
209759_s_at	DCI	BC002746	-1.355	0.0485	16p13.3
212228_s_at	DKFZP434K046	AC004382	-1.355	0.0241	16q13
219946_x_at	MYH14	NM_024729	-1.355	0.0482	19q13.33
203205_at	JMJD2A	NM_014663	-1.355	0.031	1p34.1
205300_s_at	U1SNRNPBP	NM_022717	-1.357	0.0484	12q24.31
207253_s_at	UBN1	NM_016936	-1.357	0.0384	16p13.3
203847_s_at	AKAP8	NM_005858	-1.361	0.0104	19p13.1-q12
218809_at	PANK2	NM_024960	-1.361	0.0395	20p13
213699_s_at	YWHAQ	AA854017	-1.364	0.0483	2p25.1
212787_at	YLPM1	AI952986	-1.364	0.0239	14q24.3
221636_s_at	MOSC2	AL136931	-1.364	0.0005	1q42.11
204125_at	NDUFAF1	NM_016013	-1.366	0.0338	15q11.2-q21.3
200658_s_at	PHB	NM_002634	-1.366	0.0284	17q21
212877_at	KNS2	AA284075	-1.368	0.0201	14q32.3
209439_s_at	PHKA2	D38616	-1.368	0.01	Xp22.2-p22.1
221434_s_at	DC50	NM_031210	-1.370	0.0201	14q24.3
203511_s_at	TRAPPC3	AF041432	-1.370	0.0271	1p34.3
218027_at	MRPL15	NM_014175	-1.372	0.0522	8q11.2-q13
200976_s_at	TAX1BP1	AF090891	-1.374	0.03	7p15
218160_at	NDUFA8	NM_014222	-1.376	0.0445	9q33.2-q34.11
209311_at	BCL2L2	D87461	-1.377	0.0078	14q11.2-q12
202243_s_at	PSMB4	NM_002796	-1.377	0.0518	1q21
202560_s_at	C1orf77	NM_015607	-1.377	0.0124	1q22
36564_at	IBRDC3	W27419	-1.379	0.0245	1p34.3
212673_at	METAP1	D42084	-1.381	0.0455	4q23
203565_s_at	MNAT1	NM_002431	-1.383	0.0308	14q23
41037_at	RTEF1	U63824	-1.385	0.0277	12p13.2-p13.3
210036_s_at	HERG	AB044806	-1.385	0.0291	7q35-q36
202394_s_at	ABCF3	NM_018358	-1.391	0.0128	3q27.3
203193_at	ESRRA	NM_004451	-1.393	0.0111	11q13
218372_at	MED9	NM_018019	-1.393	0.0106	17p11.2
201275_at	FDPS	NM_002004	-1.397	0.0203	1q22
221821_s_at	C12orf41	AK022732	-1.399	0.0449	12q13.12
201032_at	BLCAP	NM_006698	-1.399	0.0022	20q11.2-q12
213346_at	C13orf27	BE748563	-1.401	0.0105	13q33.1
212293_at	HIPK1	BG111260	-1.401	0.052	1p13.1
200754_x_at	SFRS2	NM_003016	-1.403	0.0366	17q25.3
210896_s_at	ASPH	AF306765	-1.403	0.0468	8q12.1
200634_at	PFN1	NM_005022	-1.404	0.0499	17p13.3
218777_at	REEP4	NM_025232	-1.404	0.0421	8p21.3
202383_at	JARID1C	NM_004187	-1.404	0.043	Xp11.22-p11.21
214846_s_at	ALPK3	AB037751	-1.406	0.0207	15q25.2
221069_s_at	CCDC44	NM_016360	-1.406	0.0333	17q24.2
203340_s_at	SLC25A12	AI887457	-1.408	0.0088	2q24
200764_s_at	CTNNA1	AI826881	-1.408	0.0345	5q31
215424_s_at	SKIIP	AV689564	-1.410	0.0066	14q24.3

200070_at	C2orf24	BC001393	-1.410	0.0028	2q36.1
201323_at	EBNA1BP2	NM_006824	-1.412	0.0174	1p35-p33
1487_at	ESRRA	L38487	-1.416	0.0217	11q13
208728_s_at	CDC42	BC003682	-1.416	0.0047	1p36.1
218536_at	MRS2L	AF052167	-1.416	0.0054	6p22.3-p22.1
218714_at	MGC3121	NM_024031	-1.418	0.0191	16p11.2
218234_at	ING4	NM_016162	-1.420	0.0537	12p13.31
201192_s_at	PITPNA	NM_006224	-1.420	0.0045	17p13.3
204331_s_at	MRPS12	AA587905	-1.420	0.0298	19q13.1-q13.2
201489_at	PPIF	BC005020	-1.422	0.0398	10q22-q23
218934_s_at	HSPB7	NM_014424	-1.422	0.032	1p36.23-p34.3
203229_s_at	CLK2	NM_003993	-1.422	0.0385	1q21
205181_at	ZNF193	NM_006299	-1.422	0.0433	6p21.3
208424_s_at	CIAPIN1	NM_020313	-1.427	0.005	16q13-q21
200987_x_at	PSME3	AA758755	-1.427	0.0081	17q21
209488_s_at	RBP-MS	D84109	-1.427	0.0046	8p12-p11
202128_at	KIAA0317	NM_014821	-1.429	0.0361	14q24.2
213794_s_at	NGDN	AI269117	-1.431	0.0107	14q11.2
205356_at	USP13	NM_003940	-1.431	0.0317	3q26.2-q26.3
208827_at	PSMB6	BC000835	-1.433	0.0465	17p13
218442_at	TTC4	NM_004623	-1.437	0.0448	1p31.3
59625_at	NOL3	AI912351	-1.439	0.0075	16q22.1
204706_at	INPP5E	NM_019892	-1.439	0.0098	9q34.3
201157_s_at	NMT1	AF020500	-1.443	0.0081	17q21.31
219146_at	C17orf42	NM_024683	-1.445	0.0504	17q11.2
204288_s_at	SORBS2	NM_021069	-1.451	0.0364	4q35.1
219314_s_at	ZNF219	NM_016423	-1.453	0.0206	14q11
219032_x_at	CHML	NM_014322	-1.453	0.0055	1q43
201842_s_at	EFEMP1	AI826799	-1.453	0.0268	2p16
203243_s_at	PDLIM5	NM_006457	-1.456	0.0265	4q22
212456_at	KIAA0664	AB014564	-1.460	0.0192	17p13.3
204824_at	ENDOG	NM_004435	-1.466	0.0056	9q34.1
217249_x_at	WUGSC:H_RG162B04.1	AC004544	-1.471	0.0193	4q22.3
201422_at	IFI30	NM_006332	-1.473	0.0234	19p13.1
219142_at	RASL11B	NM_023940	-1.473	0.0287	4q12
204547_at	RAB40B	NM_006822	-1.475	0.0221	17q25.3
209248_at	GHITM	AL136713	-1.477	0.0233	10q23.2
206453_s_at	NDRG2	NM_016250	-1.477	0.0457	14q11.2
213213_at	DATF1	AL035669	-1.481	0.0056	20q13.33
204122_at	TYROBP	NM_003332	-1.481	0.0049	19q13.1
203275_at	IRF2	NM_002199	-1.481	0.0209	4q34.1-q35.1
200850_s_at	AHCYL1	NM_006621	-1.484	0.0343	1p12
212277_at	MTMR4	AB014547	-1.486	0.009	17q22-q23
209039_x_at	EHD1	AF001434	-1.488	0.0118	11q13
212872_s_at	TRFP	AK023092	-1.488	0.0046	6p21.1
206059_at	ZNF91	NM_003430	-1.490	0.0103	19p13.1-p12
215711_s_at	WEE1	AJ277546	-1.493	0.053	11p15.3-p15.1
205295_at	CKMT2	NM_001825	-1.497	0.0003	5q13.3
220688_s_at	MRT4	NM_016183	-1.499	0.0331	1p36.13
218938_at	FBXL15	NM_024326	-1.502	0.0253	10q24.32
219310_at	C20orf39	NM_024893	-1.502	0.0017	20p11.21
218869_at	MLYCD	NM_012213	-1.504	0.0408	16q23.3

201966_at	NDUFS2	NM_004550	-1.504	0.001	1q23
203880_at	COX17	NM_005694	-1.504	0.022	3q13.33
218425_at	TRIAD3	BC000787	-1.506	0.0185	7p22.2
218119_at	TIMM23	NM_006327	-1.508	0.0382	10q11.21-q11.23
209252_at	HARSL	U18937	-1.508	0.0161	5q31.3
221031_s_at	APOLD1	NM_030817	-1.511	0.0036	12p13.2
213510_x_at	FAM106A	AW194543	-1.511	0.046	17p11.2
221669_s_at	ACAD8	BC001964	-1.517	0.0249	11q25
203597_s_at	WBP4	AI734228	-1.517	0.0508	13q13.3
217935_s_at	C20orf44	NM_018244	-1.517	0.0119	20q11.23
201221_s_at	SNRP70	NM_003089	-1.522	0.0244	19q13.3
219214_s_at	RBAK	NM_021163	-1.524	0.0288	7p22.2
200980_s_at	MAP3K15	BF739979	-1.524	0.0088	Xp22.2-p22.1
214027_x_at	DES	AA889653	-1.529	0.0079	2q35
201226_at	NDUFB8	NM_005004	-1.536	0.016	10q23.2-q23.33
219045_at	RHOF	NM_019034	-1.536	0.0153	12q24.31
203487_s_at	ARMC8	NM_015396	-1.541	0.0463	3q22.3
217959_s_at	TRAPPC4	NM_016146	-1.548	0.0257	11q23.3
200875_s_at	NOL5A	NM_006392	-1.550	0.0082	20p13
202825_at	SLC25A4	NM_001151	-1.550	0.0263	4q35
218434_s_at	AACS	NM_023928	-1.553	0.0387	12q24.31
200894_s_at	FKBP4	NM_002014	-1.558	0.0549	12p13.33
205766_at	TCAP	NM_003673	-1.560	0.0532	17q12
217168_s_at	HERPUD1	AF217990	-1.565	0.0331	16q12.2-q13
59999_at	HIF1AN	W37897	-1.575	0.0093	10q24
215049_x_at	CD163	Z22969	-1.582	0.0268	12p13.3
201093_x_at	SDHA	NM_004168	-1.582	0.0145	5p15
203957_at	E2F6	NM_001952	-1.592	0.0328	2p25.1
213063_at	EML5	BF970253	-1.597	0.0522	14q31.3
201967_at	RBM6	NM_005777	-1.597	0.0499	3p21.3
210305_at	PDE4DIP	AB042557	-1.605	0.0172	1q12
204091_at	PDE6D	NM_002601	-1.608	0.0171	2q35-q36
212266_s_at	SFRS5	AW084582	-1.613	0.0541	14q24
208813_at	GOT1	BC000498	-1.616	0.0014	10q24.1-q25.1
219188_s_at	LRP16	BC000270	-1.616	0.0389	11q11
204676_at	C16orf51	NM_015421	-1.616	0.005	16p13.13-p12.3
200768_s_at	MAT2A	BC001686	-1.621	0.0417	2p11.2
204186_s_at	PPID	AI014573	-1.621	0.0324	4q31.3
204837_at	MTMR9	AL080178	-1.626	0.0287	8p23-p22
209008_x_at	KRT8	U76549	-1.629	0.047	12q13
214978_s_at	PPFIA4	AK023365	-1.629	0.0484	1q32.1
209312_x_at	HLA-DRB1	U65585	-1.634	0.0069	6p21.3
213648_at	EXOSC7	AW614427	-1.637	0.0001	3p21.32
203367_at	DUSP14	NM_007026	-1.645	0.0128	17q12
217759_at	TRIM44	AA176780	-1.664	0.0429	11p13
212891_s_at	GADD45GIP1	BF972185	-1.664	0.0402	19p13.2
217329_x_at	COX7BP1	AF042164	-1.669	0.0318	22q12.3
211150_s_at	DLAT	J03866	-1.678	0.0377	11q23.1
205738_s_at	FABP3	NM_004102	-1.692	0.017	1p33-p32
203252_at	CDK2AP2	NM_005851	-1.718	0.0211	11q13
215210_s_at	E2k	S72422	-1.724	0.0138	14q24.3
211715_s_at	BDH1	BC005844	-1.805	0.0201	3q29

212274_at	LPIN1	AV705559	-1.838	0.0317	2p25.1
200692_s_at	HSPA9	NM_004134	-1.873	0.0463	5q31.1
215795_at	MYH7B	AK000947	-1.923	0.0139	20q11.23
217356_s_at	PGK1	S81916	-1.949	0.0374	Xq13
218695_at	EXOSC4	NM_019037	-1.961	0.0498	8q24.3
213507_s_at	KPNB1	BG249565	-1.988	0.0012	17q21.32
202416_at	DNAJC7	NM_003315	-2.016	0.0034	17q11.2
204963_at	SSPN	AL136756	-2.132	0.0321	12p11.2
205493_s_at	DPYSL4	NM_006426	-2.404	0.0131	10q26
216834_at	BL34	S59049	-2.653	0.0263	1q31
