# Supplementary material for: Altered expression of mitochondrial and extracellular matrix genes in the heart of human fetuses with chromosome 21 trisomy
Source: BMC Genomics. 2007 Aug 7;8:268. doi: 10.1186/1471-2164-8-268 (PMC1964766; doi:10.1186/1471-2164-8-268)
Supplement: Additional file 5 — List of downregulated genes encoding mitochondrial proteins, and of upregulated genes encoding extracellular matrix proteins. Hsa21 genes are in bold. [file 1471-2164-8-268-S5.pdf]

Table S4: List of downregulated genes encoding mitochondrial proteins, and of upregulated genes encoding extracellular matrix proteins.
Hsa21 genes are in bold.

Downregulated genes

Probe ID	Fold Change (DSH/NH ratio)	Gene Name	Genbank ID
214274_s_at	0.647	ACAA1	AI860341
215210_s_at	0.548	ACADM	S72422
205412_at	0.756	ACAT1	NM_000019
208967_s_at	0.757	AK2	U39945
201322_at	0.739	ATP5B	NM_001686
208972_s_at	0.739	ATP5G1	AL080089
211715_s_at	0.554	BDH1	BC005844
205295_at	0.699	CKMT2	NM_001825
209746_s_at	0.721	COQ7	AF032900
203858_s_at	0.74	COX10	NM_001303
218057_x_at	0.738	COX4NB	BC001472
201597_at	0.764	COX7A2	NM_001865
201633_s_at	0.727	CYB5B	AW235051
208905_at	0.702	CYCS	BC005299
209759_s_at	0.729	DCI	BC002746
211150_s_at	0.568	DLAT	J03866
204824_at	0.638	ENDOG	NM_004435
201931_at	0.698	ETFA	NM_000126
202942_at	0.692	ETFB	NM_001985
213133_s_at	0.713	GCSH	AW237404
221415_s_at	0.545	GJA10	NM_030772
200947_s_at	0.758	GLUD1	NM_005271
208813_at	0.671	GOT1	BC000498
203745_at	0.678	HCCS	AI801013
200691_s_at	0.645	HSPA9B	NM_004134
210046_s_at	0.743	IDH2	U52144
202070_s_at	0.709	IDH3A	NM_005530
210418_s_at	0.751	IDH3B	AF023265
200955_at	0.704	IMMT	NM_006839
36830_at	0.711	MIPEP	U80034
219527_at	0.701	MOSC2	NM_017898
218027_at	0.716	MRPL15	NM_014175
203781_at	0.741	MRPL33	NM_004891
218890_x_at	0.745	MRPL35	NM_016622
204331_s_at	0.735	MRPS12	AA587905
220864_s_at	0.638	NDUFA13	NM_015965
202077_at	0.748	NDUFAB1	NM_005003
218201_at	0.669	NDUFB2	NM_004546
201226_at	0.663	NDUFB8	NM_005004
201966_at	0.62	NDUFS2	NM_004550
201740_at	0.72	NDUFS3	NM_004551
201757_at	0.763	NDUFS5	NM_004552
202941_at	0.76	NDUFV2	NM_021074
218455_at	0.712	NFS1	NM_021100

202780_at	0.705	OXCT1	NM_000436
200980_s_at	0.598	PDHA1	BF739979
214225_at	0.677	PIN4	BE674061
203649_s_at	0.657	PLA2G2A	NM_000300
205241_at	0.724	SCO2	NM_005138
201093_x_at	0.591	SDHA	NM_004168
202675_at	0.744	SDHB	NM_003000
202004_x_at	0.722	SDHC	NM_003001
203340_s_at	0.688	SLC25A12	AI887457
217961_at	0.705	SLC25A38	NM_017875
202825_at	0.607	SLC25A4	NM_001151
216841_s_at	0.734	SOD2	X15132
218119_at	0.634	TIMM23	NM_006327
203092_at	0.612	TIMM44	AF026030
220415_at	0.635	TNNI3K	NM_015978
209077_at	0.74	TXN2	AL022313
201903_at	0.75	UQCRC1	NM_003365
200883_at	0.716	UQCRC2	NM_003366
208909_at	0.744	UQCRFS1	BC000649
217140_s_at	0.637	VDAC1	AJ002428
217249_x_at	0.68	WUGSC:H_RG162B04.1	AC004544

Upregulated genes

Probe ID	Fold Change (DSH/NH ratio)	Gene name	GenBank ID
222162_s_at	1.93	**ADAMTS1**	AK023795
219935_at	1.5	**ADAMTS5**	NM_007038
220706_at	2.24	ADAMTS7	NM_014272
214953_s_at	1.668	**APP**	X06989
219087_at	1.829	ASPN	NM_017680
220988_s_at	1.466	C1QTNF3	NM_030945
219025_at	1.342	CD248	NM_020404
211809_x_at	1.721	COL13A1	M59217
203477_at	1.413	COL15A1	NM_001855
209081_s_at	1.57	COL18A1	AF018081
202310_s_at	1.546	COL1A1	K01228
202403_s_at	1.596	COL1A2	NM_000089
212489_at	1.595	COL5A1	AI983428
212091_s_at	1.927	**COL6A1**	AI141603
209156_s_at	2.391	**COL6A2**	AY029208
213622_at	1.434	COL9A2	AI733465
204724_s_at	1.6	COL9A3	NM_001853
207420_at	4.268	COLEC10	NM_006438
209335_at	1.73	DCN	BC005322
213661_at	1.516	DKFZP586H2123	AI671186
206101_at	1.554	ECM2	NM_001393
202994_s_at	1.716	FBLN1	Z95331
204359_at	1.522	FLRT2	NM_013231
209220_at	1.685	GPC3	L47125
206766_at	1.378	ITGA10	AF112345

216190_x_at	3.591	ITGB1	AA215854
204989_s_at	2.269	ITGB4	BF305661
221462_x_at	3.113	KLK15	NM_017509
202202_s_at	1.591	LAMA4	NM_002290
203417_at	1.434	MFAP2	NM_017459
203877_at	1.673	MMP11	NM_005940
201069_at	1.586	MMP2	NM_004530
205907_s_at	1.961	OMD	AI765819
202465_at	1.482	PCOLCE	NM_002593
218585_s_at	1.48	RAMP	AK001261
218452_at	1.859	SMARCAL1	NM_014140
205236_x_at	1.372	SOD3	NM_003102
208606_s_at	2.534	WNT4	NM_030761
