# Supplementary material for: Altered expression of mitochondrial and extracellular matrix genes in the heart of human fetuses with chromosome 21 trisomy
Source: BMC Genomics. 2007 Aug 7;8:268. doi: 10.1186/1471-2164-8-268 (PMC1964766; doi:10.1186/1471-2164-8-268)
Supplement: Additional file 6 — Genes differentially expressed between trisomic and control samples using gc-RMA preprocessed data. The table includes genes with fold change > |1.2| and p < 0.05 (ANOVA test). Genes are sorted according to fold change. Hsa21 genes are in bold. [file 1471-2164-8-268-S6.pdf]

Table S5. - Genes differentially expressed between trisomic and control samples using gc-RMA preprocessed data.
The table includes genes with fold change > |1.2| and p<0.05 (ANOVA test). Genes are sorted according to fold change. Hsa21 genes are in bold.

Probe ID	Fold change	Gene name	GenBank ID	Chromosomal location
205608_s_at	4.25	ANGPT1	U83508	8q22.3-q23
222106_at	3.67	PRND	AL133396	20pter-p12
211560_s_at	3.41	ALAS2	AF130113	Xp11.21
213135_at	3.40	**TIAM1**	U90902	21q22.1
209840_s_at	3.16	LRRN3	AI221950	7q31.1
201668_x_at	3.00	MARCKS	AW163148	6q22.2
205593_s_at	2.98	**PDE9A**	NM_002606	21q22.3
209156_s_at	2.81	**COL6A2**	AY029208	21q22.3
209541_at	2.66	IGF1	AI972496	12q22-q23
204606_at	2.51	CCL21	NM_002989	9p13
210746_s_at	2.46	EPB42	M30646	15q15-q21
202555_s_at	2.46	MYLK	NM_005965	3q21
205592_at	2.45	IL8	NM_000342	17q21-q22
221796_at	2.44	NTRK2	AA707199	9q22.1
205923_at	2.36	RELN	NM_005045	7q22
219213_at	2.34	**JAM2**	NM_021219	21q21.2
203758_at	2.29	CTSO	AV729484	4q31-q32
208850_s_at	2.28	THY1	AL558479	11q22.3-q23
210198_s_at	2.27	PLP1	BC002665	Xq22
213094_at	2.22	GPR126	AL033377	---
219179_at	2.19	DACT1	NM_016651	14q23.1
209735_at	2.18	BCRP	AF098951	4q22
222162_s_at	2.13	**ADAMTS1**	AK023795	21q21.2
204358_s_at	2.08	FLRT2	NM_013231	14q24-q32
214433_s_at	2.08	SELENBP1	NM_003944	1q21-q22
202242_at	2.06	TSPAN7	NM_004615	Xp11.4
206101_at	2.05	ECM2	NM_001393	9q22.3
210510_s_at	2.04	NRP1	AF145712	10p12
217867_x_at	2.04	**BACE2**	AF178532	21q22.3
204623_at	1.99	**TFF3**	NM_003226	21q22.3
208073_x_at	1.97	**TTC3**	NM_003316	21q22.2
202350_s_at	1.97	MATN2	NM_002380	8q22
217525_at	1.95	OLFML1	AW305097	11p15.4
202557_at	1.95	**STCH**	AI718418	21q11.1
209220_at	1.94	GPC3	L47125	Xq26.1
201387_s_at	1.93	UCHL1	NM_004181	4p14
213661_at	1.88	DKFZP586H2123	AI671186	11p13
209121_x_at	1.88	NR2F2	M64497	15q26
213125_at	1.88	OLFML2B	AW007573	1q23.1
209866_s_at	1.88	LPHN3	AF307080	4q13.1
213989_x_at	1.87	**SETD4**	AB004853	21q22.13
203865_s_at	1.87	**ADARB1**	NM_015833	21q22.3
207173_x_at	1.86	CDH11	D21254	16q22.1
213222_at	1.86	PLCB1	AL049593	20p12
47069_at	1.85	PRR5	AA533284	22q13.31
202994_s_at	1.83	FBLN1	Z95331	22q13.31
201497_x_at	1.82	MYH11	NM_022844	16p13.13-p13.12
203666_at	1.81	CXCL12	NM_000609	10q11.1
202310_s_at	1.81	COL1A1	K01228	17q21.3-q22.1
212846_at	1.81	**KIAA0179**	AA811192	21q22.3
203789_s_at	1.80	SEMA3C	NM_006379	7q21-q31
209335_at	1.79	DCN	BC005322	12q13.2
203185_at	1.79	RASSF2	NM_014737	20pter-p12.1
205548_s_at	1.78	**BTG3**	NM_006806	21q21.1-q21.2
213541_s_at	1.78	**ERG**	AI351043	21q22.3
201525_at	1.78	APOD	NM_001647	3q26.2-qter
203889_at	1.77	SCG5	NM_003020	15q13-q14
204785_x_at	1.77	**IFNAR2**	L41944	21q22.1
201642_at	1.77	**IFNGR2**	NM_005534	21q22.11

204442_x_at	1.76	LTBP4	NM_003573	19q13.1-q13.2
218377_s_at	1.76	**C21orf6**	NM_016940	21q22.11
209043_at	1.76	PAPSS1	AF033026	4q24
219087_at	1.76	ASPN	NM_017680	9q22
212713_at	1.75	MFAP4	R72286	17p11.2
202403_s_at	1.75	COL1A2	NM_000089	7q22.1
201860_s_at	1.75	PLAT	NM_000930	8p12
201830_s_at	1.74	NET1	NM_005863	10p15
200974_at	1.74	ACTA2	NM_001613	10q23.3
219767_s_at	1.74	**CRYZL1**	NM_005111	21q21.3
209578_s_at	1.73	**POFUT2**	BC000626	21q22.3
212190_at	1.73	SERPINE2	AL541302	2q33-q35
214439_x_at	1.72	BIN1	AF043899	2q14
212192_at	1.71	KCTD12	AI718937	13q22.1
213905_x_at	1.71	BGN	AA845258	18q22.3
203502_at	1.71	BPGM	NM_001724	7q31-q34
202016_at	1.71	MEST	NM_002402	7q32
202450_s_at	1.70	CTSK	NM_000396	1q21
202599_s_at	1.70	**NRIP1**	NM_003489	21q11.2
201995_at	1.70	EXT1	NM_000127	8q24.11-q24.13
211709_s_at	1.69	CLEC11A	BC005810	19q13.3 /// 19q13.3
214988_s_at	1.69	**SON**	X63071	21q22.1-q22.2
205151_s_at	1.69	KIAA0644	AV724192	7p15.1
217764_s_at	1.68	RAB31	AF183421	18p11.3
209033_s_at	1.68	**DYRK1A**	D86550	21q22.13
213428_s_at	1.68	**COL6A1**	AA292373	21q22.3
203325_s_at	1.68	COL5A1	AI130969	9q34.2-q34.3
209242_at	1.67	PEG3	AL042588	19q13.4
212269_s_at	1.67	**MCM3AP**	AJ010089	21q22.3
205168_at	1.66	DDR2	NM_006182	1q12-q23
218870_at	1.66	ARHGAP15	NM_018460	2q22.3
213629_x_at	1.65	MT1A	BF246115	16q13
202202_s_at	1.65	LAMA4	NM_002290	6q21
221447_s_at	1.64	GLT8D2	NM_031302	12q /// 12q
207191_s_at	1.64	ISLR	NM_005545	15q23-q24
221689_s_at	1.64	**PIGP**	AB035745	21q22.2
202749_at	1.64	**WRB**	NM_004627	21q22.3
212012_at	1.64	D2S448	BF342851	2p25
209897_s_at	1.64	SLIT2	AF055585	4p15.2
203116_s_at	1.63	FECH	NM_000140	18q21.3
208037_s_at	1.63	MADCAM1	NM_007164	19p13.3 /// 19p13.3
209583_s_at	1.63	CD200	AF063591	3q12-q13
212094_at	1.63	PEG10	BE858180	7q21
204745_x_at	1.62	MT1G	NM_005950	16q13
209297_at	1.62	**ITSN1**	AF114488	21q22.1-q22.2
215446_s_at	1.61	LOX	L16895	---
209082_s_at	1.61	**COL18A1**	AF018081	21q22.3
208817_at	1.61	COMT	BC000419	22q11.21-q11.23
214431_at	1.61	GMPS	NM_003875	3q24
209656_s_at	1.61	TM4SF10	AL136550	Xp11.4
208407_s_at	1.60	CTNND1	NM_001331	11q11
209758_s_at	1.60	MAGP2	U37283	12p13.1-p12.3
202465_at	1.60	PCOLCE	NM_002593	7q22
209560_s_at	1.59	DLK1	U15979	14q32
203423_at	1.59	RBP1	NM_002899	3q23
201976_s_at	1.59	MYO10	NM_012334	5p15.1-p14.3
201417_at	1.59	SOX4	AL136179	6p22.3
220750_s_at	1.58	LEPRE1	NM_022356	1p34.1
219935_at	1.58	**ADAMTS5**	NM_007038	21q21.3
221677_s_at	1.58	**DONSON**	AF232674	21q22.1
201427_s_at	1.58	SEPP1	NM_005410	5q31
213538_at	1.57	**SON**	AI936458	21q22.1-q22.2
214746_s_at	1.57	ZNF467	BE549732	7q36.1
201431_s_at	1.56	DPYSL3	NM_001387	5q32

221478_at	1.56	BNIP3L	AL132665	8p21
218162_at	1.55	OLFML3	NM_020190	1p13.1
213422_s_at	1.55	MXRA8	AW888223	1p36.33
200602_at	1.55	**APP**	NM_000484	21q21.2
206331_at	1.55	CALCRL	NM_005795	2q32.2
206766_at	1.54	ITGA10	AF112345	1q21
215157_x_at	1.54	PABPC1	AI734929	8q22.2-q23
204620_s_at	1.53	CSPG2	NM_004385	5q14.3
202283_at	1.52	SERPINF1	NM_002615	17p13.1
203417_at	1.52	MFAP2	NM_017459	1p36.1-p35
218386_x_at	1.52	**USP16**	AI806796	21q22.11
212233_at	1.52	MAP1B	AL523076	5q13
212761_at	1.51	TCF7L2	AI703074	10q25.3
219025_at	1.51	CD248	NM_020404	11q13
201050_at	1.51	PLD3	NM_012268	19q13.2
212653_s_at	1.51	EHBP1	AB020710	2p15
200986_at	1.50	SERPING1	NM_000062	11q12-q13.1
201069_at	1.50	MMP2	NM_004530	16q13-q21
213068_at	1.50	DPT	AI146848	1q12-q23
221564_at	1.50	**HRMT1L1**	AL570294	21q22.3
204112_s_at	1.50	HNMT	NM_006895	2q22.1
212658_at	1.50	LHFPL2	N66633	5q14.1
203562_at	1.49	FEZ1	NM_005103	11q24.2
201312_s_at	1.49	SH3BGRL	NM_003022	Xq13.3
215823_x_at	1.49	RNF12	U64661	Xq13-q21
200944_s_at	1.48	**HMGN1**	NM_004965	21q22.3
202671_s_at	1.48	**PDXK**	NM_003681	21q22.3
209570_s_at	1.48	D4S234E	BC001745	4p16.3
209506_s_at	1.48	NR2F1	BC004154	5q14
213592_at	1.47	HG11	X89271	11q12
204114_at	1.47	NID2	NM_007361	14q21-q22
200989_at	1.47	HIF1A	NM_001530	14q21-q24
202356_s_at	1.47	GTF2F1	NM_002096	19p13.3
209099_x_at	1.47	HJ1	U73936	20p12.1-p11.23
209575_at	1.47	**IL10RB**	BC001903	21q22.1-q22.2
212665_at	1.47	TIPARP	AL556438	3q25.31
205381_at	1.47	LRRC17	NM_005824	7q22.1
215222_x_at	1.46	MACF1	AK023406	1p32-p31
209022_at	1.46	STAG2	AK026678	Xq25
208113_x_at	1.45	PABPC3	NM_030979	13q12-q13
204556_s_at	1.45	DZIP1	AL568422	13q32.1
219561_at	1.45	COPZ2	NM_016429	17q21.32
201292_at	1.45	TOP2A	AL561834	17q21-q22
213000_at	1.45	**ZCWCC3**	AP000693	21q22.13
205020_s_at	1.45	ARL4A	NM_005738	7p21-p15.3
204505_s_at	1.45	EPB49	NM_001978	8p21.1
221773_at	1.44	ELK3	AW575374	12q23
212736_at	1.44	C16orf45	BE299456	16p13.13
204568_at	1.43	KIAA0831	NM_014924	14q22.2
215596_s_at	1.43	**ZNF294**	AL163248	21q22.11
200740_s_at	1.43	**SUMO3**	BG338532	21q22.3
221476_s_at	1.43	RPL15	AF279903	3p24.1
205433_at	1.43	BCHE	NM_000055	3q26.1-q26.2
203038_at	1.43	PTPRK	NM_002844	6q22.2-23.1
212205_at	1.43	H2AFV	AA534860	7p13
218930_s_at	1.43	TMEM106B	AV705186	7p21.3
204036_at	1.43	EDG2	AW269335	9q32
218558_s_at	1.42	**MRPL39**	NM_017446	21q21.3
203635_at	1.42	**DSCR3**	NM_006052	21q22.2
201201_at	1.42	**CSTB**	NM_000100	21q22.3
211065_x_at	1.42	**PFKL**	BC006422	21q22.3
213254_at	1.42	TNRC6B	N64803	22q13.2
205802_at	1.42	TRPC1	NM_003304	3q22-q24
212354_at	1.42	SULF1	AI479175	8q13.2

201215_at	1.42	PLS3	NM_005032	Xq24
212372_at	1.41	NMMHCB	AK026977	17p13
204985_s_at	1.41	TRAPPC6A	NM_024108	19q13.32
201778_s_at	1.41	ATPAF1	NM_014774	1pter-p22.1
219689_at	1.41	SEMA3G	NM_020163	3p21.31
201987_at	1.40	THRAP1	AI984051	17q22-q23
204154_at	1.40	CDO1	NM_001801	5q22-q23
204823_at	1.40	NAV3	NM_014903	chr12
202201_at	1.39	BLVRB	NM_000713	19q13.1-q13.2
200771_at	1.39	LAMC1	NM_002293	1q31
213075_at	1.39	OLFML2A	AL050002	9q34.11
201494_at	1.38	PRCP	NM_005040	11q14
203697_at	1.38	FRZB	U91903	2qter
218086_at	1.38	NPDC1	NM_015392	9q34.3
204450_x_at	1.37	APOA1	NM_000039	11q23-q24
208248_x_at	1.37	APLP2	NM_001642	11q23-q25
208986_at	1.37	TCF12	AL559478	15q21
203405_at	1.37	**DSCR2**	NM_003720	21q22.3
204979_s_at	1.37	**SH3BGR**	NM_007341	21q22.3
205801_s_at	1.37	RASGRP3	NM_015376	2p25.1-p24.1
206481_s_at	1.37	LDB2	NM_001290	4p16
221249_s_at	1.36	FAM117A	NM_030802	17q21.33
202732_at	1.36	PKIG	NM_007066	20q12-q13.1
212378_at	1.36	**GART**	NM_000819	21q22.1
212235_at	1.36	PLXND1	AL575403	3q21.3
208779_x_at	1.36	DDR1	L20817	6p21.3
218927_s_at	1.36	CHST12	BC002918	7p22
209821_at	1.36	IL33	AB024518	9p24.1
219884_at	1.36	LHX6	NM_014368	9q34.11
32625_at	1.35	NPR1	X15357	1q21-q22
218477_at	1.35	TMEM14A	NM_014051	6p12.3
213236_at	1.35	SASH1	AK025495	6q24.3
204457_s_at	1.35	GAS1	NM_002048	9q21.3-q22
203477_at	1.35	COL15A1	NM_001855	9q21-q22
213762_x_at	1.34	RBMX	AI452524	Xq26
208270_s_at	1.33	RNPEP	NM_020216	1q32
201003_x_at	1.33	UBE2V1	NM_003349	20q13.2
209272_at	1.33	NAB1	AF045451	2q32.3-q33
218452_at	1.33	SMARCAL1	NM_014140	2q34-q35
208614_s_at	1.33	FLNB	M62994	3p14.3
203049_s_at	1.33	KIAA0372	NM_014639	5q15
200023_s_at	1.32	EIF3S5	AI001896	11p15.4
208838_at	1.32	CAND1	AB020636	12q14
203167_at	1.32	TIMP2	NM_003255	17q25
202421_at	1.32	IGSF3	AB007935	1p13
202075_s_at	1.32	PLTP	NM_006227	20q12-q13.1
57715_at	1.31	FAM26B	W72694	10pter-q26.12
218326_s_at	1.31	LGR4	NM_018490	11p14-p13
212204_at	1.31	TMEM87A	AL049944	15q14
200677_at	1.31	**PTTG1IP**	NM_004339	21q22.3
202605_at	1.31	GUSB	NM_000181	7q21.11
41047_at	1.31	C9orf16	AI885170	9q34.1
201029_s_at	1.31	CD99	NM_002414	Xp22.32
213100_at	1.30	UNC5B	AA127885	10q22.2
201185_at	1.30	HTRA1	NM_002775	10q26.3
203211_s_at	1.30	MTMR2	NM_016156	11q22
221726_at	1.30	RPL22	BE250348	1p36.3-p36.2
212586_at	1.30	CAST	AA195244	5q15-q21
213074_at	1.30	PHIP	BG545769	6q14-q15
209337_at	1.30	PSIP2	AF063020	9p22.2
204462_s_at	1.30	SLC16A2	NM_006517	Xq13.2
204759_at	1.29	RCBTB2	NM_001268	13q14.3
217731_s_at	1.29	ITM2B	NM_021999	13q14.3
213392_at	1.29	IQCK	AW070229	16p13.11

218684_at	1.29	LRRC8D	NM_018103	1p22.2
218253_s_at	1.29	LGTN	NM_006893	1q31-q32
203919_at	1.29	TCEA2	NM_003195	20q13.33
211675_s_at	1.29	MDFIC	AF054589	7q31.2
219049_at	1.29	ChGn	NM_018371	8p21.3
202974_at	1.29	MPP1	NM_002436	Xq28
217890_s_at	1.28	PARVA	NM_018222	11p15.3
56256_at	1.28	SIDT2	AA150165	11q23.2
204428_s_at	1.28	LCAT	NM_000229	16q22.1
214259_s_at	1.28	AKR7A2	AI144075	1p35.1-p36.23
214043_at	1.28	PTPRD	BF062299	9p23-p24.3
200003_s_at	1.27	RPL28	NM_000991	19q13.4
217922_at	1.27	MAN1A2	BE543064	1p13
212294_at	1.27	GNG12	BG111761	1p31.2
205176_s_at	1.27	ITGB3BP	NM_014288	1p31.3
212725_s_at	1.27	TUG1	N37081	22q12.2
201121_s_at	1.27	PGRMC1	NM_006667	Xq22-q24
200642_at	1.26	**SOD1**	NM_000454	21q22.1
218575_at	1.26	ANAPC1	NM_022662	2q12.1
203799_at	1.25	CD302	NM_014880	2q24.2
208801_at	1.25	SRP72	AV702627	4q11
200795_at	1.25	SPARCL1	NM_004684	4q22.1
217025_s_at	1.25	DBN1	AL110225	5q35.3
200608_s_at	1.25	RAD21	NM_006265	8q24
211935_at	1.24	ARL6IP	D31885	16p12-p11.2
218353_at	1.24	RGS5	NM_025226	1q23.1
213037_x_at	1.24	STAU1	AJ132258	20q13.1
202606_s_at	1.24	TLK1	NM_012290	2q31.1
221934_s_at	1.24	DALRD3	BF941492	3p21.31
218109_s_at	1.24	MFSD1	NM_022736	3q25.32
218662_s_at	1.24	HCAP-G	NM_022346	4p16-p15
212731_at	1.24	ANKRD46	U79297	8q22.3
214749_s_at	1.24	ARMCX6	AK000818	Xq21.33-q22.3
212498_at	1.23	MARCH-VI	AF056433	---
160020_at	1.23	MMP14	Z48481	14q11-q12
201590_x_at	1.23	ANXA2	NM_004039	15q21-q22
201178_at	1.23	FBXO7	NM_012179	22q12-q13
212698_s_at	1.23	SEPT10	BF966021	2q13
65718_at	1.23	GPR124	AI655903	8p11.23
200016_x_at	1.22	HNRPA1	NM_002136	12q13.1
36907_at	1.22	MVK	M88468	12q24
221511_x_at	1.22	CPR8	AF212228	15q21.1
201217_x_at	1.22	RPL3	NM_000967	22q13
217783_s_at	1.22	YPEL5	NM_016061	2p23.3
205080_at	1.22	RARB	NM_000965	3p24
208858_s_at	1.21	MBC2	BC004998	12q13.13
201105_at	1.21	LGALS1	NM_002305	22q13.1
203150_at	1.21	RABEPK	NM_005833	9q34.11
211983_x_at	1.20	ACTG1	BE741683	17q25
202799_at	1.20	CLPP	NM_006012	19p13.3
215146_s_at	1.20	TTC28	AB028966	22q12.1
208667_s_at	1.20	ST13	U17714	22q13.2
210946_at	1.20	PPAP2A	AF014403	5q11
218938_at	-1.20	FBXL15	NM_024326	10q24.32
205423_at	-1.20	AP1B1	NM_001127	22q12
209389_x_at	-1.20	DBI	M15887	2q12-q21
217408_at	-1.20	HLA-E	AL050361	6p21.3
208398_s_at	-1.20	TBPL1	NM_004865	6q22.1-q22.3
209066_x_at	-1.20	UQBP	M26700	8q22
219283_at	-1.20	C1GALT1C1	NM_014158	Xq25
202298_at	-1.20	NDUFA1	NM_004541	Xq25-q26
201479_at	-1.20	DKC1	NM_001363	Xq28
218403_at	-1.21	TRIAP1	NM_016399	12q24.31
218074_at	-1.21	FAM96B	NM_016062	16q22.1-q22.3

202682_s_at	-1.21	USP4	NM_003363	3p21.3
218265_at	-1.21	SECISBP2	NM_024077	9q22.2
203406_at	-1.22	MFAP1	NM_005926	15q15-q21
212507_at	-1.22	RW1	D87446	2q11.2
201517_at	-1.22	NCBP2	BC001255	3q29
201186_at	-1.22	LRPAP1	NM_002337	4p16.3
212696_s_at	-1.22	C4orf8	BF968633	4p16.3
203218_at	-1.22	MAPK9	W37431	5q35
201597_at	-1.22	COX7A2	NM_001865	6q12
204045_at	-1.22	TCEAL1	NM_004780	Xq22.1
218780_at	-1.23	HOOK2	NM_013312	19p13.2
218996_at	-1.23	TFPT	NM_013342	19q13
213702_x_at	-1.23	ASAH1	AI934569	8p22-p21.3
203613_s_at	-1.23	NDUFB6	NM_002493	9p13.3
203459_s_at	-1.23	VPS16	NM_022575	chr20
205553_s_at	-1.24	CSRP3	NM_003476	11p15.1
202077_at	-1.24	NDUFAB1	NM_005003	16p12.3
201840_at	-1.25	NEDD8	NM_006156	14q11.2
203858_s_at	-1.25	COX10	NM_001303	17p12-17p11.2
219274_at	-1.25	TSPAN12	NM_012338	7q31.31
219449_s_at	-1.25	TMEM70	BC002748	8q13.3
218671_s_at	-1.26	ATPIF1	NM_016311	---
208687_x_at	-1.26	HSPA8	AF352832	11q24.1
209075_s_at	-1.26	ISCU	AY009128	12q24.1
203862_s_at	-1.26	ACTN2	H16245	1q42-q43
218254_s_at	-1.26	SAR1B	NM_016103	5q31.2
219129_s_at	-1.26	SAP30L	NM_024632	5q33.2
209080_x_at	-1.26	PICOT	AF118652	6p25.3
219433_at	-1.26	BCOR	NM_017745	Xp11.4
213149_at	-1.27	DLAT	AW299740	11q23.1
209233_at	-1.27	EMG1	U72514	12p13
218093_s_at	-1.27	ANKRD10	NM_017664	13q34
201192_s_at	-1.27	PITPNA	NM_006224	17p13.3
221636_s_at	-1.27	MOSC2	AL136931	1q42.11
207507_s_at	-1.27	ATP5G3	NM_001689	2q31.2
212441_at	-1.27	KIAA0232	D86985	4p16.1
204706_at	-1.27	INPP5E	NM_019892	9q34.3
220934_s_at	-1.28	MGC3196	NM_024084	---
218238_at	-1.28	GTPBP4	NM_012341	10p15-p14
203737_s_at	-1.28	PPRC1	NM_015062	10q24.32
217772_s_at	-1.28	MTCH2	NM_014342	11p11.2
220526_s_at	-1.28	MRPL20	NM_017971	1p36.3-p36.2
201460_at	-1.28	MAPKAPK2	AI141802	1q32
201937_s_at	-1.28	DNPEP	NM_012100	2q36.1
204842_x_at	-1.28	PRKAR2A	BC002763	3p21.3-p21.2
207335_x_at	-1.28	ATP5I	NM_007100	4p16.3
203606_at	-1.28	NDUFS6	NM_004553	5p15.33
206688_s_at	-1.28	CPSF4	NM_006693	7q22.1
213196_at	-1.28	ZNF629	AI924293	chr16
203051_at	-1.29	BAHD1	NM_014952	15q14
53987_at	-1.29	RANBP10	AL041852	16q22.1
208909_at	-1.29	UQCRFS1	BC000649	19q12-q13.1
209142_s_at	-1.29	UBE2G1	BC002775	1q42
217746_s_at	-1.29	PDCD6IP	NM_013374	3p22.3
222010_at	-1.29	ACAT2	BF224073	6q25.3-q26
209039_x_at	-1.30	EHD1	AF001434	11q13
221434_s_at	-1.30	C14orf156	NM_031210	14q24.3
200694_s_at	-1.30	DDX24	NM_020414	14q32
212505_s_at	-1.30	KIAA0892	AL110250	19p13.11
208967_s_at	-1.30	AK2	U39945	1p34
218716_x_at	-1.30	MTO1	NM_012123	6q14.1
213011_s_at	-1.30	TPI1	BF116254	chr12
209439_s_at	-1.30	PHKA2	D38616	Xp22.2-p22.1
203118_at	-1.31	PCSK7	NM_004716	11q23-q24

205963_s_at	-1.31	DNAJA3	NM_005147	16p13.3
202093_s_at	-1.31	PAF1	NM_019088	19q13.1
202263_at	-1.31	CYB5R1	NM_016243	1p36.13-q41
202427_s_at	-1.31	BRP44	NM_015415	1q24
205633_s_at	-1.31	ALAS1	NM_000688	3p21.1
200976_s_at	-1.31	TAX1BP1	AF090891	7p15
203391_at	-1.32	FKBP2	NM_004470	11q13.1-q13.3
203832_at	-1.32	SNRPF	NM_003095	12q23.1
219116_s_at	-1.32	DCUN1D2	NM_018185	13q34
214531_s_at	-1.32	SNX1	AF065484	15q22.1
221255_s_at	-1.32	TMEM93	NM_031298	17p13.3
218419_s_at	-1.32	TMUB2	NM_024107	17q21.31
218307_at	-1.32	RSAD1	NM_018346	17q21.33
201757_at	-1.32	NDUFS5	NM_004552	1p34.2-p33
200764_s_at	-1.32	CTNNA1	AI826881	5q31
218160_at	-1.32	NDUFA8	NM_014222	9q33.2-q34.11
201226_at	-1.33	NDUFB8	NM_005004	10q23.2-q23.33
215749_s_at	-1.33	GORASP1	AK001574	3p22-p21.33
207302_at	-1.34	SGCG	NM_000231	13q12
212783_at	-1.34	RBBP6	AK026954	16p12.2
202230_s_at	-1.34	CHERP	NM_006387	19p13.1
210574_s_at	-1.34	NUDC	AF241788	1p35-p34
214027_x_at	-1.34	DES	AA889653	2q35
217976_s_at	-1.34	DNCLI1	NM_016141	3p22.3
209084_s_at	-1.34	RFC1	BE504689	4p14-p13
201740_at	-1.35	NDUFS3	NM_004551	11p11.11
212228_s_at	-1.35	DKFZP434K046	AC004382	16q13
203230_at	-1.35	DVL1	AF006011	1p36
218682_s_at	-1.35	SLC4A1AP	NM_018158	2p23.3
221897_at	-1.35	TRIM52	AA205660	5q35.3
203647_s_at	-1.36	FDX1	NM_004109	11q22
200850_s_at	-1.36	AHCYL1	NM_006621	1p12
208918_s_at	-1.36	CDC2L2	BE674658	1p36.33-p36.21
201377_at	-1.36	UBAP2L	NM_014847	1q22
203517_at	-1.36	MTX2	NM_006554	2q31.2
200070_at	-1.36	C2orf24	BC001393	2q36.1
204824_at	-1.36	ENDOG	NM_004435	9q34.1
212088_at	-1.36	PMPCA	BF570122	9q34.3
208424_s_at	-1.37	CIAPIN1	NM_020313	16q13-q21
218771_at	-1.37	PANK4	NM_018216	1p36.32
202401_s_at	-1.37	SRF	NM_003131	6p21.1
219185_at	-1.37	SIRT5	NM_012241	6p23
205052_at	-1.37	AUH	NM_001698	9q22.31
221746_at	-1.37	UBL4	BE543027	Xq28
205562_at	-1.38	RPP38	NM_006414	10p13
213398_s_at	-1.38	C14orf124	AI347090	14q11.2
218506_x_at	-1.38	N-PAC	NM_018459	16p13.3
221692_s_at	-1.38	MRPL34	AB049652	19p13.1
31874_at	-1.38	GAS2L1	Y07846	22q12.2
202825_at	-1.38	SLC25A4	NM_001151	4q35
203814_s_at	-1.38	NQO2	NM_000904	6pter-q12
217722_s_at	-1.39	NGRN	NM_016645	15q26.1
221506_s_at	-1.39	TNPO2	BG258639	19p13.2
203511_s_at	-1.39	TRAPPC3	AF041432	1p34.3
213462_at	-1.39	NPAS2	AW000928	2q11.2
205300_s_at	-1.40	U1SNRNPBP	NM_022717	12q24.31
214679_x_at	-1.40	NCLN	AL110227	19p13.3
201966_at	-1.40	NDUFS2	NM_004550	1q23
212852_s_at	-1.40	SSA2	AL538601	1q31
205241_at	-1.40	SCO2	NM_005138	22q13.33
209248_at	-1.41	GHITM	AL136713	10q23.2
219045_at	-1.41	RHOF	NM_019034	12q24.31
214141_x_at	-1.41	SFRS7	BF033354	2p22.1
212872_s_at	-1.41	TRFP	AK023092	6p21.1

41037_at	-1.42	RTEF1	U63824	12p13.2-p13.3
205219_s_at	-1.42	GALK2	NM_002044	15q15.3
218916_at	-1.42	ZNF768	NM_024671	16p11.2
213104_at	-1.42	C16orf42	AI799802	16p13.3
208827_at	-1.42	PSMB6	BC000835	17p13
219310_at	-1.42	C20orf39	NM_024893	20p11.21
219142_at	-1.42	RASL11B	NM_023940	4q12
208772_at	-1.42	ANKHD1	AU160676	5q31.3
220966_x_at	-1.42	ARPC5L	NM_030978	9q34.11
203827_at	-1.43	WIPI1	NM_017983	17q24.3
204868_at	-1.43	ICT1	NM_001545	17q25.2
204122_at	-1.43	TYROBP	NM_003332	19q13.1
201032_at	-1.43	BLCAP	NM_006698	20q11.2-q12
203340_s_at	-1.43	SLC25A12	AI887457	2q24
203880_at	-1.43	COX17	NM_005694	3q13.33
205181_at	-1.43	ZNF193	NM_006299	6p21.3
216841_s_at	-1.43	SOD2	X15132	6q25.3
211784_s_at	-1.44	SFRS1	BC006181	---
1487_at	-1.44	ESRRA	L38487	11q13
201491_at	-1.44	AHSA1	NM_012111	14q23.3-31
212456_at	-1.44	KIAA0664	AB014564	17p13.3
204547_at	-1.44	RAB40B	NM_006822	17q25.3
202224_at	-1.45	CRK	NM_016823	---
219244_s_at	-1.45	MRPL46	NM_022163	15q24-q25
218372_at	-1.45	MED9	NM_018019	17p11.2
219212_at	-1.46	HSPA14	NM_016299	10p14
201489_at	-1.46	PPIF	BC005020	10q22-q23
204257_at	-1.46	FADS3	NM_021727	11q12-q13.1
218572_at	-1.46	CHMP4A	NM_014169	14q11.2
202070_s_at	-1.46	IDH3A	NM_005530	15q25.1-q25.2
217727_x_at	-1.46	VPS35	NM_018206	16q12
201157_s_at	-1.46	NMT1	AF020500	17q21.31
204937_s_at	-1.46	ZNF274	NM_016325	19qter
200980_s_at	-1.46	MAP3K15	BF739979	Xp22.2-p22.1
213507_s_at	-1.47	KPNB1	BG249565	17q21.32
208478_s_at	-1.47	BAX	NM_004324	19q13.3-q13.4
218548_x_at	-1.47	TEX264	NM_015926	3p21.31
204245_s_at	-1.48	RPP14	NM_007042	3p21.2
203203_s_at	-1.50	KRR1	NM_007043	12q21.1
218068_s_at	-1.50	ZNF672	NM_024836	1q44
201739_at	-1.50	SGK	NM_005627	6q23
218434_s_at	-1.52	AACS	NM_023928	12q24.31
219526_at	-1.52	C14orf169	NM_024644	14q24.2
214882_s_at	-1.52	SFRS2	BG254869	17q25.3
221031_s_at	-1.53	APOLD1	NM_030817	12p13.2
212877_at	-1.53	KNS2	AA284075	14q32.3
201967_at	-1.54	RBM6	NM_005777	3p21.3
205295_at	-1.55	CKMT2	NM_001825	5q13.3
221517_s_at	-1.56	CRSP6	AF105421	11q14
59625_at	-1.57	NOL3	AI912351	16q22.1
208498_s_at	-1.57	AMY1A	NM_004038	1p21
209252_at	-1.58	HARSL	U18937	5q31.3
217526_at	-1.59	NFATC2IP	AI478300	16p12.1
201422_at	-1.59	IFI30	NM_006332	19p13.1
201323_at	-1.59	EBNA1BP2	NM_006824	1p35-p33
203598_s_at	-1.60	WBP4	AK000979	13q13.3
212848_s_at	-1.60	C9orf3	BG036668	9q22.33
218744_s_at	-1.61	PACSIN3	NM_016223	11p12-p11.12
217959_s_at	-1.61	TRAPPC4	NM_016146	11q23.3
220225_at	-1.61	IRX4	NM_016358	5p15.3
205356_at	-1.62	USP13	NM_003940	3q26.2-q26.3
219006_at	-1.63	C6orf66	NM_014165	6q16.3
208813_at	-1.64	GOT1	BC000498	10q24.1-q25.1
206453_s_at	-1.64	NDRG2	NM_016250	14q11.2

204331_s_at	-1.64	MRPS12	AA587905	19q13.1-q13.2
200768_s_at	-1.64	MAT2A	BC001686	2p11.2
201248_s_at	-1.65	SREBF2	NM_004599	22q13
205738_s_at	-1.66	FABP3	NM_004102	1p33-p32
209700_x_at	-1.66	PDE4DIP	AB042555	1q12
222025_s_at	-1.66	OPLAH	AI991887	8q24.3
219269_at	-1.67	HMBOX1	NM_024567	8p21.1
219032_x_at	-1.68	CHML	NM_014322	1q43
203252_at	-1.69	CDK2AP2	NM_005851	11q13
200692_s_at	-1.70	HSPA9	NM_004134	5q31.1
203367_at	-1.72	DUSP14	NM_007026	17q12
207791_s_at	-1.72	RAB1A	NM_004161	2p14
204186_s_at	-1.72	PPID	AI014573	4q31.3
211990_at	-1.72	HLA-DPA1	M27487	6p21.3
217759_at	-1.76	TRIM44	AA176780	11p13
215193_x_at	-1.83	HLA-DRB1	AJ297586	6p21.3
215210_s_at	-1.86	DLST	S72422	14q24.3
217356_s_at	-1.89	PGK1	S81916	Xq13
220858_at	-1.93	PRO0618	NM_014133	4q35.1
204963_at	-2.04	SSPN	AL136756	12p11.2
215795_at	-2.16	MYH7B	AK000947	20q11.23
202416_at	-2.26	DNAJC7	NM_003315	17q11.2
209774_x_at	-2.26	CXCL2	M57731	4q21
205493_s_at	-2.37	DPYSL4	NM_006426	10q26
205805_s_at	-2.37	ROR1	NM_005012	1p32-p31
