# Supplementary material for: Altered expression of mitochondrial and extracellular matrix genes in the heart of human fetuses with chromosome 21 trisomy
Source: BMC Genomics. 2007 Aug 7;8:268. doi: 10.1186/1471-2164-8-268 (PMC1964766; doi:10.1186/1471-2164-8-268)
Supplement: Additional file 7 — Enrichment score plots of the five biologically informative sets correlated to the DS condition with an FDR value < 0.05. Extracellular matrix and Cell adhesion gene sets are positively correlated to DS condition whereas Mitochondria, Electron transport chain and Oxidative phosphorylation gene sets are negatively correlated. The enrichment score (ES) represents the degree to which a gene set is enriched at the top (positive ES) or at the bottom (negative ES) of our ranked list. The size indicated for each gene set is the dimension of the leading edge subset that is the subset of members of our list that contribute more to the enrichment score (ES). The nominal p-value and the False Discovery Rate (FDR) value estimate the probability that the enrichment score represents a false positive finding. [file 1471-2164-8-268-S7.pdf]

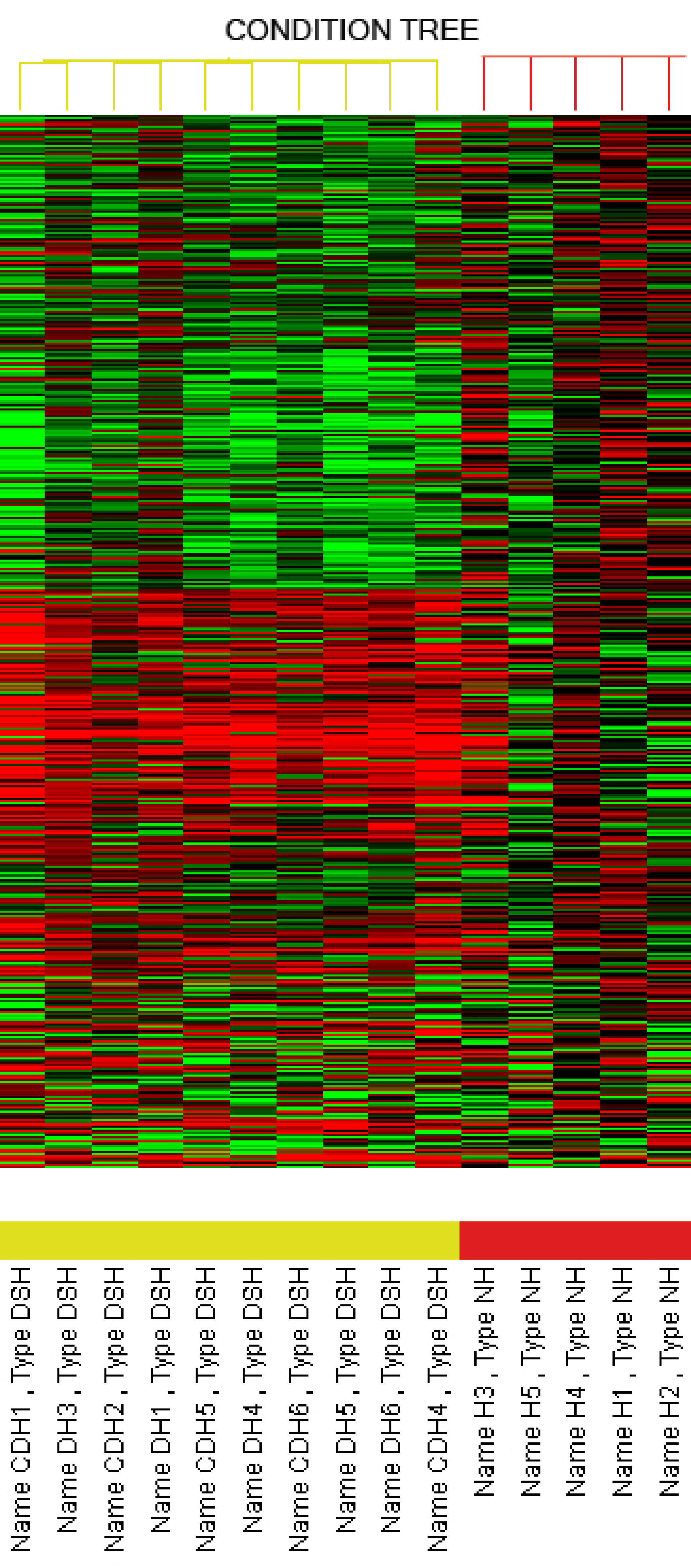
CONDITION TREE
Name CDH1 , Type DSH
Name DH3 , Type DSH
Name CDH2 , Type DSH
Name DH1 , Type DSH
Name CDH5 , Type DSH
Name DH4 , Type DSH
Name CDH6 , Type DSH
Name DH5 , Type DSH
Name DH6 , Type DSH
Name CDH4 , Type DSH
Name H3 , Type NH
Name H5 , Type NH
Name H4 , Type NH
Name H1 , Type NH
Name H2 , Type NH
