# Supplementary material for: Altered expression of mitochondrial and extracellular matrix genes in the heart of human fetuses with chromosome 21 trisomy
Source: BMC Genomics. 2007 Aug 7;8:268. doi: 10.1186/1471-2164-8-268 (PMC1964766; doi:10.1186/1471-2164-8-268)
Supplement: Additional file 8 — Condition tree generated using the hierarchical clustering approach. The tree groups samples together based on the similarity of their expression data across a gene list including ~1000 genes, not mapping to Hsa21, which encode mitochondrial and ECM proteins. The 15 DS samples are clustered together on the left whereas the five control samples are clustered on the right of the image demonstrating that the expression of genes in the specified list can be used to correctly separate DSH from NH samples. [file 1471-2164-8-268-S8.pdf]

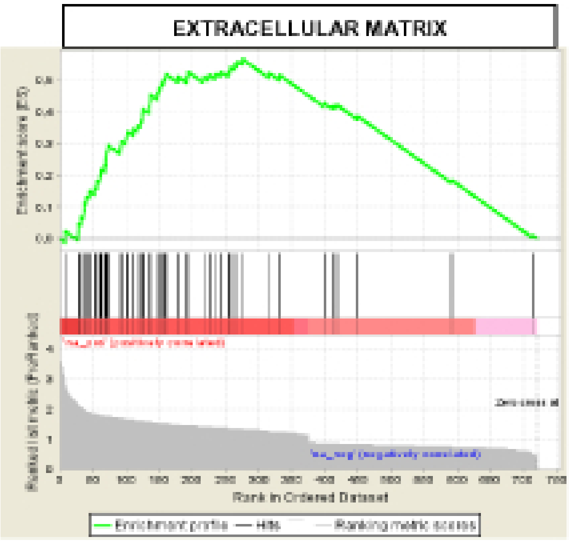

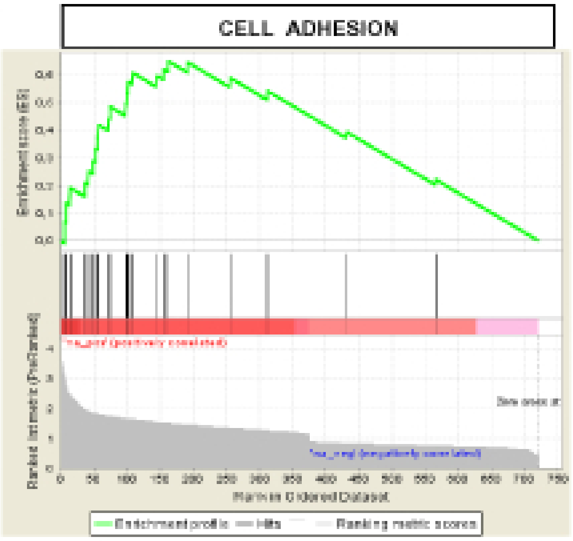

GSEA Report for Upregulated Genes in the DSH versus NH comparison

GENE SET NAME	SIZE	ES	Nom p-value	FDR
EXTRACELLULAR MATRIX GO:31012	53	0,5660	<0,001	<0,0001
CELL_ADHESION	15	0,5872	<0,001	0,0178

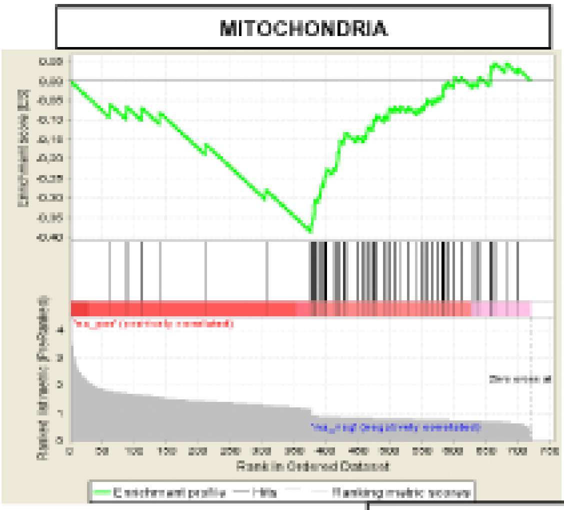

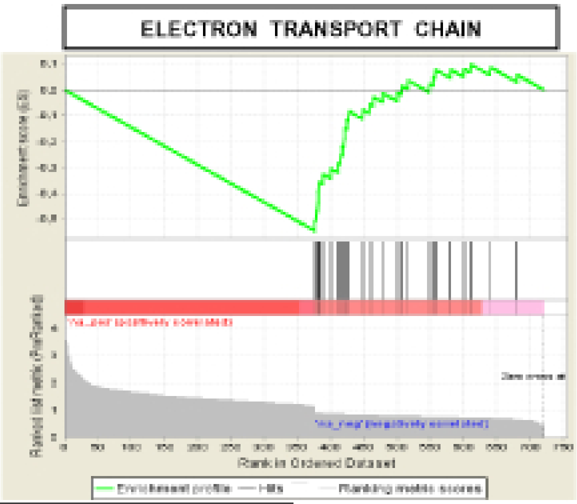

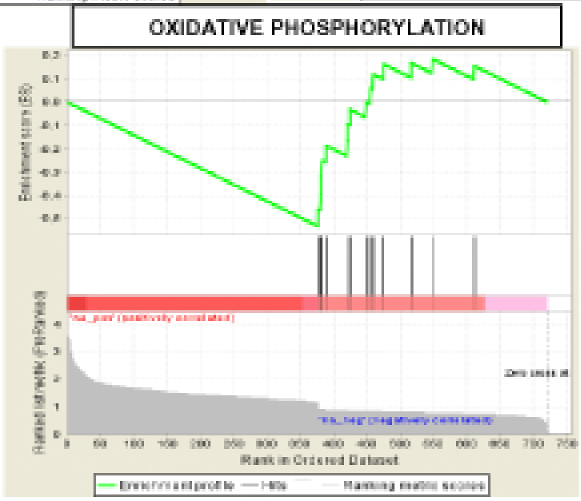

GSEA Report for Downregulated Genes in the DSH versus NH comparison

GENE SET NAME	SIZE	ES	Nom p-value	FDR
MITOCHONDRIA	62	-0,3865	<0,001	<0,0001
ELECTRON_TRANSPORT_CHAIN	29	-0,5448	<0,001	<0,0001
OXIDATIVE_PHOSPHORYLATION	15	-0,5340	<0,001	0,0004
